# Silver-Catalyzed (*Z*)-β-Fluoro-vinyl Iodonium Salts from Alkynes: Efficient and Selective Syntheses of *Z*-Monofluoroalkenes

**DOI:** 10.1021/jacs.4c03826

**Published:** 2024-06-03

**Authors:** Alexi T. Sedikides, Alastair J. J. Lennox

**Affiliations:** School of Chemistry, University of Bristol, Cantock’s Close, Bristol, BS8 1TS, United Kingdom

## Abstract

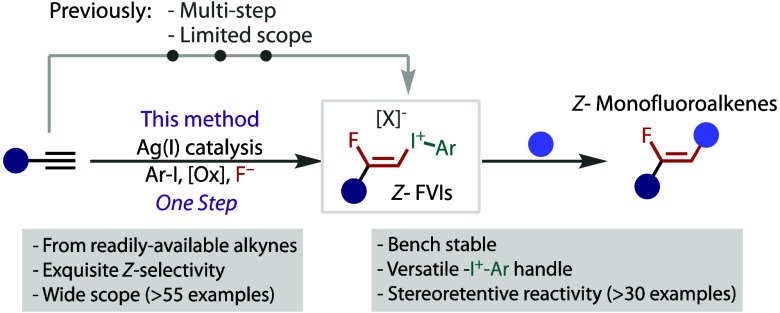

Monofluoroalkenes are stable and lipophilic amide bioisosteres used in medicinal chemistry. However, efficient and stereoselective methods for synthesizing *Z*-monofluoroalkenes are underdeveloped. We envisage *(Z)-*β-fluoro-vinyl iodonium salts (*Z*-FVIs) as coupling partners for the diverse and stereoselective synthesis of *Z*-monofluoroalkenes. Disclosed herein is the development and application of a silver(I)-catalyzed process for accessing a broad scope of (*Z*)-FVIs with exclusive *Z*-stereoselectivity and regioselectivity from alkynes in a single step. Experimental and computational studies provide insight into the mechanism of the catalytic cycle and the role of the silver(I) catalyst, and the reactivity of (*Z*)-FVIs is explored through several stereospecific derivatizations.

As effective bioisosteres of amides, monofluoroalkenes are privileged organofluorine structures.^1−4^ Specifically, internal trisubstituted *Z-*monofluoroalkenes bear the substitution pattern and stereochemistry that resembles secondary *s*-*trans*-amides that dominate in medicinal chemistry.^5^*Z-*monofluoroalkenes display close geometric and electronic alignment to these amides, including mimicry of polarity and dipole orientation (Figure 1A). This bioisosterism has been employed to improve hydrolytic stability,^6,7^ potency,^8−11^ lipophilicity,^12^ and conformational rigidity by preventing *E/Z* (*s-cis/s-trans*) interconversion.^13^ Furthermore, monofluoroalkenes are intermediates toward alkyl fluorides and fluoropolymers.^14−25^

**Figure 1:**
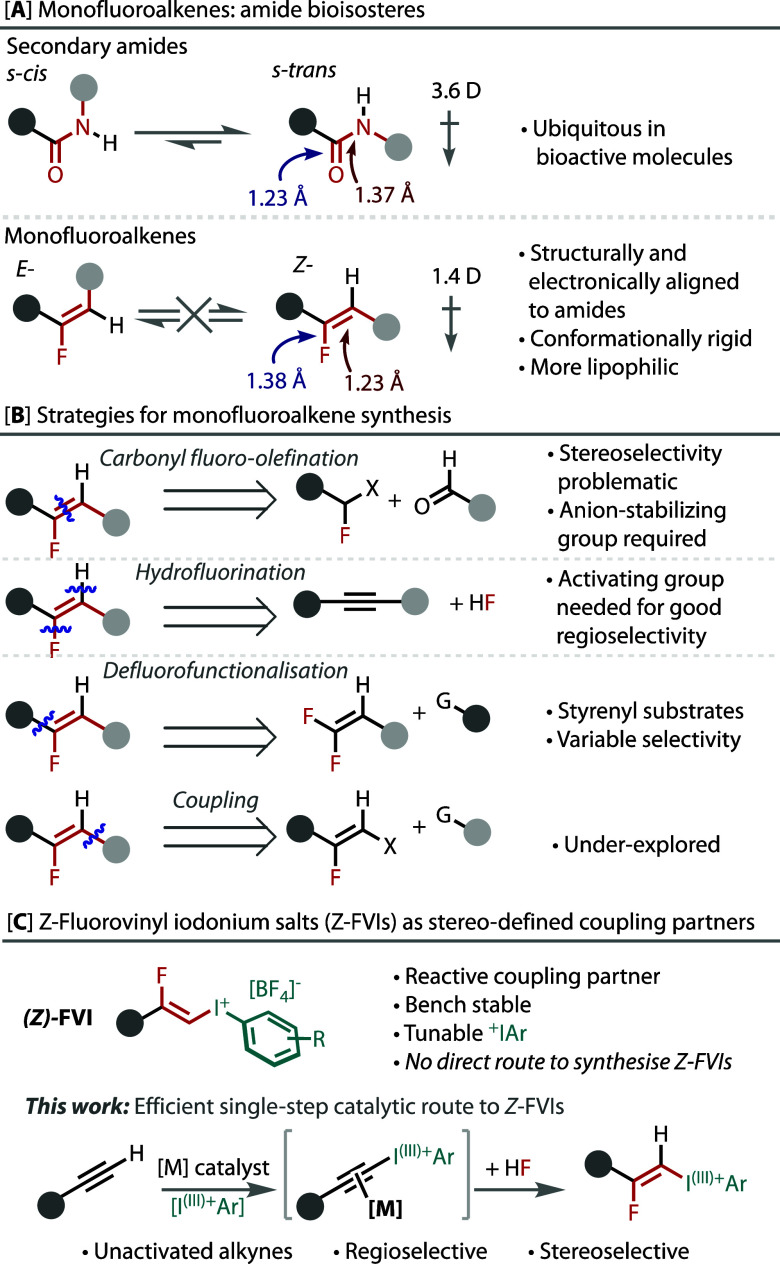
Fluoroalkenes as amide bioisosteres and their synthesis.

While this array of applications has attracted attention to their synthesis,^26,27^ there are several features of current strategies that limit their general utility (Figure 1B). For example, the fluoro-olefination of carbonyls contends with stereoselectivity issues and requires anion-stabilizing groups next to the C–F bond.^28−33^ The control of regioselectivity in direct alkyne hydrofluorination can be challenging unless a large electronic disparity exists on the alkyne.^34−40^ Finally, the defluorofunctionalization of *gem*-difluoroalkenes can occur via radical, carbanion, or migratory insertion pathways but are generally constrained to styrenyl substrates, and the stereoselectivity is variable without a directing group.^41−45^

An alternative approach is based on cross-coupling with a *Z-*β-fluorovinyl coupling partner (Figure 1B). Such a strategy relies on a stereodefined electrophilic fluoroalkene building block that can engage in stereoretentive coupling. However, this strategy has remained relatively elusive, possibly because of the difficulty in accessing suitably reactive *Z*-orientated coupling partners. With reactivity, stability and tunability in mind, we were drawn to *Z-*fluorovinyl iodonium salts (*Z-*FVIs) as potentially general linchpin building blocks (Figure 1C). The hypervalent (aryl)-iodonium group is a tunable functional handle that is more reactive and versatile than the corresponding bromo- or iodo-fluoroalkenes yet is still bench-stable.

Accessing *Z*-FVIs currently requires a multistep route that involves handling several unstable intermediates.^46−50^ As such, there are only limited reports of their use,^51−54^ which directly contrasts the extensive reports of related hypervalent iodine-based aryl, alkyl, or alkynyl transfer reagents for C–C and C–X bond formation.^55−63^ Hence, we proposed that a single-step method for their synthesis, with exclusive *Z*-selectivity, should invigorate their use as coupling partners.

The most efficient means to access diverse *Z*-FVIs should be the direct and selective fluoroiodanation, F/I(III), of readily available unactivated alkynes. However, the corresponding iodo-fluorination, F/I(I), of alkynes using an electrophilic iodine reagent gives *E*-selective iodofluoroalkenes.^64−70^ As metal salts can catalyze alkyne *anti-*hydrofluorination,^35−40^ we considered whether a strategy based on a metal-catalyzed *anti*-hydrofluorination of an *in situ* formed alkynyliodane could be feasible (Figure 1C). Herein, we describe the development of this catalytic strategy to achieve excellent regio- and stereoselectivity from unactivated alkynes.

Optimization was initiated by targeting the formation of mesityl-difluoro-λ^3^-iodane through the combination of 2-iodomesitylene, fluoride, and an oxidant^71−75^ (see the Supporting Information). Combining this with alkyne **1a** in MeCN did not lead to any observable product **3a** (Table 1, entry 1). Interestingly, when phenyl acetylene (**1b**) was subjected to these conditions, low yields of the *E*-isomer were observed (entry 2). We then conducted an extensive screening of oxidants (including mCPBA and oxone), solvents, sources of fluoride, and metal salts (see the Supporting Information). Ag_2_CO_3_ (1 equiv) was the only metal salt to yield moderate yields of **3a** and did so with exclusive stereo- and regioselectivity (entry 3).^39,76^ MeNO_2_ as the solvent gave the highest yield, although the greener dimethylcarbonate (DMC) also gave good yields (entries 4–6 and the Supporting Information).

**Table 1:**
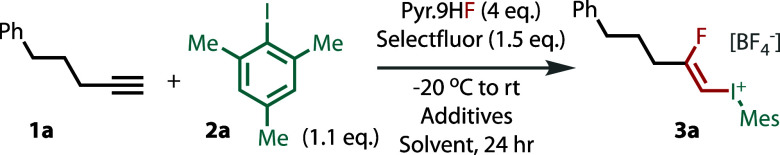
Reaction Optimization

entry	solvent	additives (equiv)	yield **3a**/%^a^
1	MeCN	none	0
2^b^	MeCN	none	0 (11% *E*-FVI)
3	MeCN	Ag_2_CO_3_ (1)	41
4	EtOAc	Ag_2_CO_3_ (1)	39
5	DMC	Ag_2_CO_3_ (1)	65
6	MeNO_2_	Ag_2_CO_3_ (1)	79
7	MeNO_2_	Ag_2_CO_3_ (0.5)	45
8	MeNO_2_	Ag_2_CO_3_ (0.1)	0
9	MeNO_2_	AgF (1)	22
10	MeNO_2_	AgOTf (1)	12
11	MeNO_2_	AgBF_4_ (1)	11
12	MeNO_2_	AgBF_4_ (1) + Cs_2_CO_3_ (1)	65
13	MeNO_*2*_	Ag_2_CO_3_ (0.1) + K_2_CO_3_ (3)	79
14	MeNO_2_	K_2_CO_3_ (3)	0
15^c^	MeNO_2_	Ag_2_CO_3_ (0.1) + K_2_CO_3_ (3)	45

a ^19^F NMR yield against internal standard.

b Phenylacetylene (**1b**) used instead of **1a**.

c HF/pyridine complex (pyr/9HF) (1 equiv) used. DMC = dimethylcarbonate.

When substoichiometric, catalytic quantities of Ag_2_CO_3_ were tested, a decline in yield was observed (entries 7 and 8). Other silver salts did not lead to improvements (entries 9–11 and the Supporting Information). However, we discovered that the addition of Cs_2_CO_3_ to AgBF_4_ greatly enhanced the yield compared with that without the base (entry 12 vs 11). Hence, other bases were tested with Ag_2_CO_3_ in which we found 3 equiv of K_2_CO_3_ switched on silver(I) catalysis (entries 13 and 14). A very good yield of **3a** with exclusive stereo- and regioselectivity were provided from 10 mol % Ag_2_CO_3_ and 3 equiv of K_2_CO_3_. As organic bases also showed this enhancement in the yield (see the Supporting Information), we propose that the reactivity of fluoride is fine-tuned by altering the hydrogen bonding environment.

The scope of the transformation was then explored in Figure 2. The reactions were performed in air using reagent-grade solvents and reagents without further drying or degassing. The FVIs were isolated and purified by trituration, filtration, and evaporation and all proved to be air- and moisture-stable at room temperature over a period of several months.

**Figure 2:**
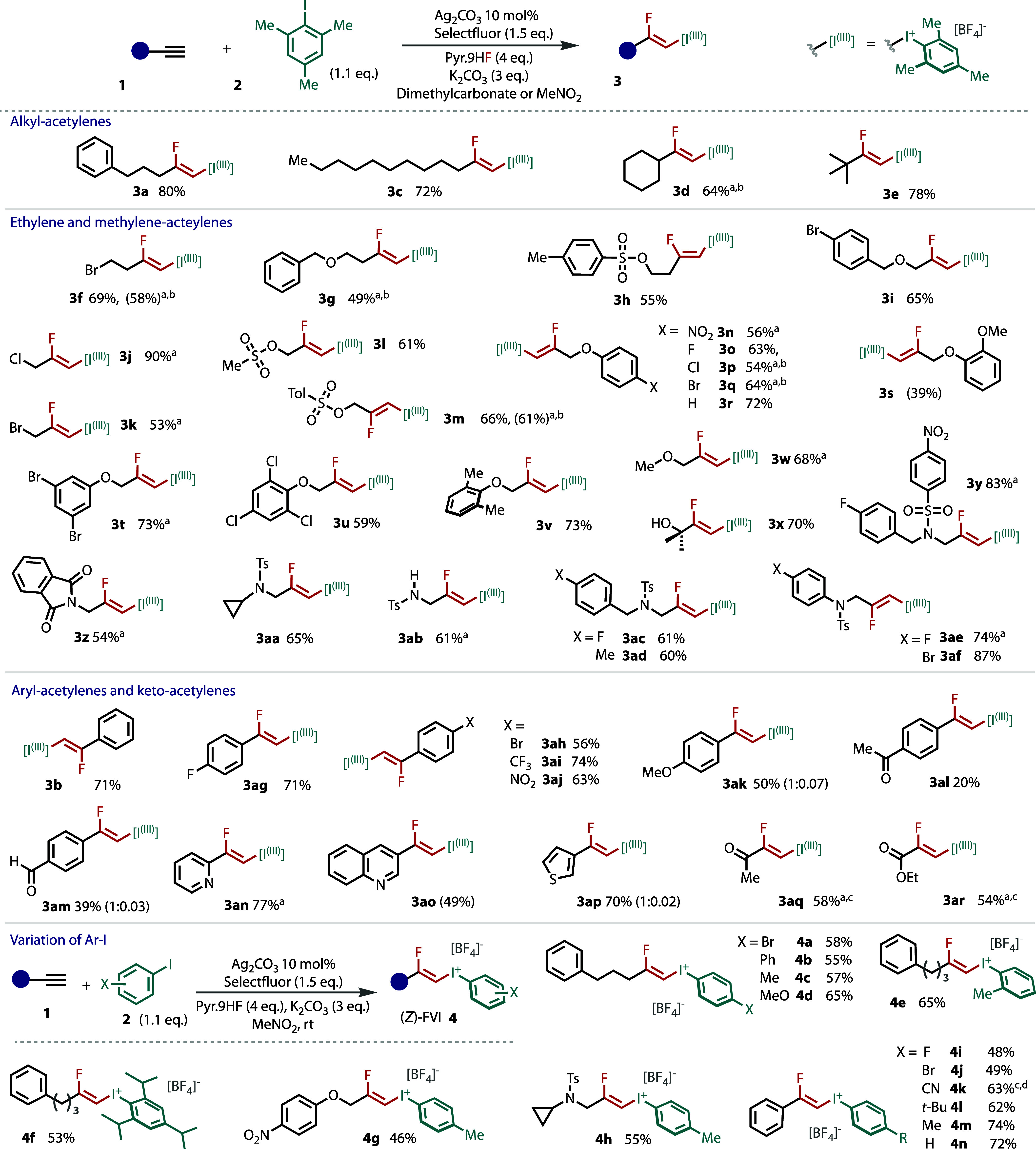
Isolated yields given and quantitative ^19^F NMR yields shown in parentheses. Ratio of (*Z*/*E*) given whenever the *E*-isomer was observed. ^a^No K_2_CO_3_ added; ^b^dimethylcarbonate (DMC) used in place of MeNO_2_; ^c^100 mol % Ag_2_CO_3_; and ^d^2.5 equiv of Selectfluor.

Unactivated alkyl-acetylenes performed well with primary (**3a**,**c**), secondary (**3d**), and tertiary alkyl groups (**3e**) on the alkyne (Figure 2). Alkynes with both ethylene (**3f**–**h**) and methylene (**3i–af**) units to heteroatoms were accommodated and transformed with exclusive selectivity. Tolerance to chloride (**3j**), bromide (**3k**), mesylate (**3l**), and tosylate (**3m,l**) leaving groups was observed, which is noteworthy considering the potential for substitution by fluoride. Ether (**3g**,**i**), alcohol (**3x**), and amine (**3z–ad**) functionalities were tolerated, as were phenolic aryl ethers (**3o**–**w**) and anilines (**3ae**,**af**) that are susceptible to oxidation. Only substrates that are highly sensitive to the oxidative and acidic conditions were not tolerated (see the Supporting Information).

Electron-rich, -neutral, and -deficient aryl-acetylenes (**3b**,**ag**–**am**) and keto-acetylenes (**3aq**–**ar**) were competent coupling partners (Figure 2). Without the addition of K_2_CO_3_ to aryl-acetylenes, the *E*-isomer was observed as a prominent product. This reactivity trend was not as rigid for alkyl-acetylenes where K_2_CO_3_ was not always necessary (e.g., **3f**). K_2_CO_3_ was most influential on the yield for substrates containing electron-rich, easily oxidized functionality. Finally, a variety of heterocyclic arylacetylene substrates were also successfully transformed (**3an–ap**).

Varying the aryl iodide component can tune the reactivity of *Z*-FVIs *vide infra*. Hence, various aryl iodides were used, which gave good yields (**4a**–**n**) of FVIs with broad tolerance of electronics and sterics, and exclusive *Z*-selectivity.

Key aspects of the reaction mechanism were investigated through experimental and computational studies (Figure 3). In the absence of silver(I), no *Z*-FVI was observed with alkyl-acetylenes, and only low amounts were observed with aryl-acetylenes. Hence, the role of Ag in the catalytic cycle and the origin of the stereo- and regioselectivity was investigated. These studies led to our proposed mechanism with alkyne coordination to Ag(I) (***I***) and the formation of alkynyl-Ag(I) ***II***, which then reacts with Mes-IF_2_ to give alkynyl-iodonium ***III*** followed by Ag(I)-mediated hydrofluorination via ***IV*** to yield the β-*Z*-FVI.

**Figure 3:**
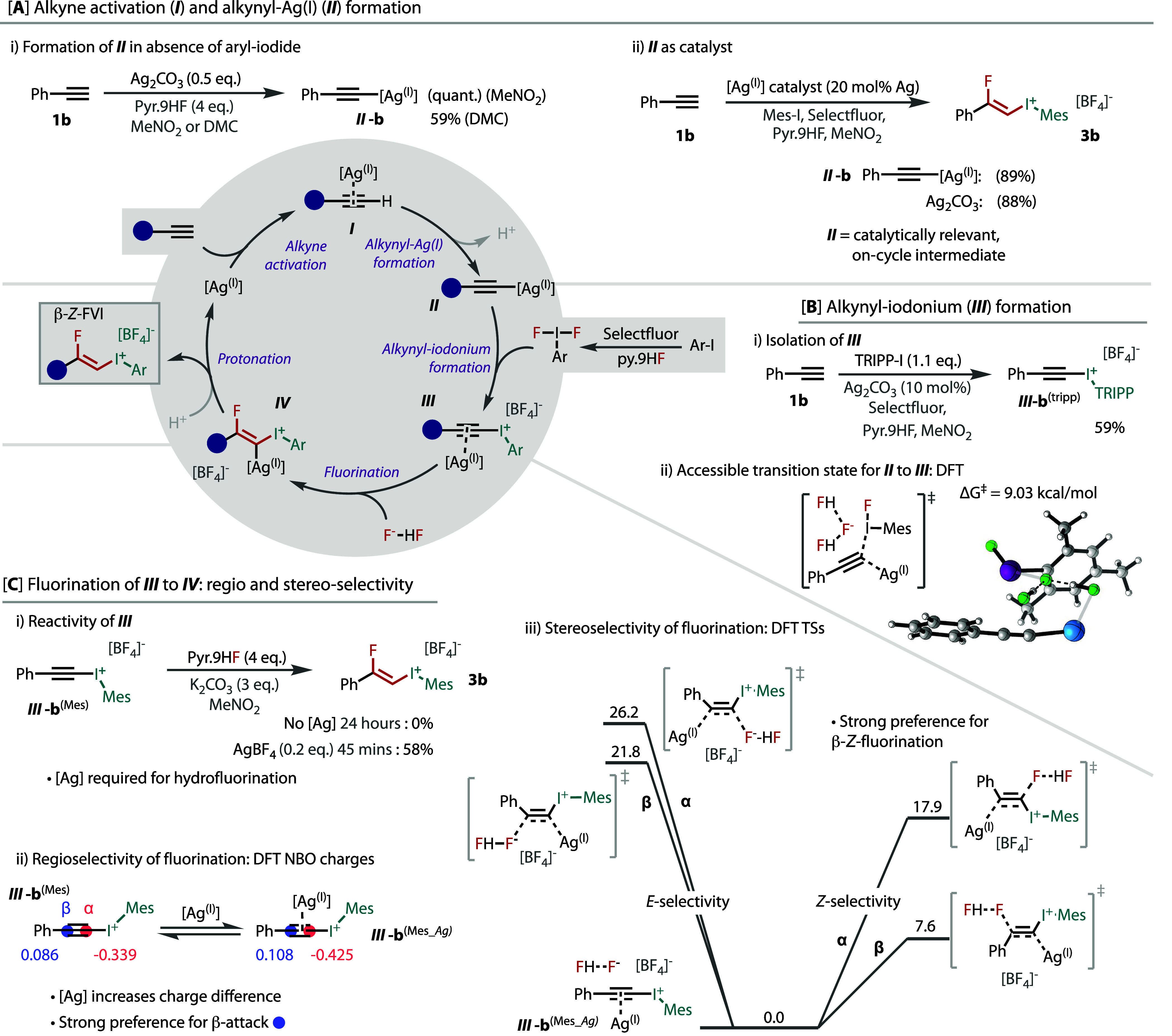
Mechanistic experimental and computational (M06-2X/def2-TZVP, IEFPCM(MeNO_2_)) studies of the Ag(I) catalytic cycle. TRIPP = 2,4,6-triisopropylphenyl.

Phenylethynyl-Ag(I) ***II*-b** was observed (^1^H NMR) when phenylacetylene (**1b**) was subjected to the conditions in the absence of aryl iodide or oxidant (Figure 3A,i). We isolated this species and confirmed its identity via an independent literature procedure.^77^ The reaction worked as efficiently with ***II*-b** as a source of silver catalyst, as shown in Figure 3A,ii. Hence, ***II*** should be a catalytically relevant, on-cycle species.

Alkynyl-iodonium [***III***^(Mes)^] could not be observed by NMR, likely because of rapid hydrofluorination. However, when using the more bulky and less reactive 2-iodo-1,3,5-triisopropylbenzene, alkynyl-iodonium ***III-*b**^(tripp)^ was observed (Figure 3B,i). Density functional theory (DFT) calculations revealed accessible transition states for transformation of alkynyl-silver(I) ***II*** to alkynyl-iodonium ***III*** (Figure 3B,ii).

To investigate the reactivity of alkynyl-iodonium ***III***, we prepared ***III*-b**^(Mes)^ (Figure 3C,i) and ***III*-b**^(Ph)^ (see the Supporting Information) via an independent literature procedure.^78^ In the absence of silver, both ***III*-b** compounds failed to react, even after 24 h. Conversely, rapid hydrofluorination took place in the presence of catalytic silver(I) tetrafluoroborate, which formed the *Z-*FVI in good yield after only 45 min, thus supporting the role of silver in this step. The hydrofluorination of the corresponding iodoalkyne was also tested, but no iodofluoroalkene was formed either in the presence or absence of Ag(I) (see the Supporting Information).

The fluorination of species ***III*** determines the regio- and stereoselectivity of the reaction. Natural bond orbital (NBO) calculations on alkynyl-iodonium ***III*-b** confirmed a partial positive charge where fluorination is observed at the β-position because of its relative positioning to an electron-withdrawing group (Figure 3C,ii). This charge difference is further enhanced (C_α_–C_β_) upon η^2^-coordination to Ag(I). Transition-state energies for fluorination are greatly decreased by the coordination of Ag(I) (see the Supporting Information), and show a strong preference for β-fluorination over α-fluorination (Figure 3C,iii).

To rationalize the *Z-*stereochemistry, we computed the *syn*- and *anti*-additions of Ag/F across alkynyl-iodonium ***III***, which correspond to pathways leading to *E* and *Z*-FVIs, respectively (Figure 3C,iii). Various silver coordination modes and anion/hydrogen-bonding environments were considered (see the Supporting Information), and in all cases, they supported the *anti*-(*Z-*)pathway in preference to the *syn*-(*E-*)-pathway. We propose the *anti*-Ag/F addition transition states are lower in energy because of a lower steric crowding between the forming cis-related substituents, as well as a possible Ag-fluoride interaction for *E*-selectivity transition state that serves to increase the barrier.

Collectively, these data suggest a dual role for Ag(I): first, alkyne activation toward alkynyl iodonium ***III*** formation, and second, mediation of *anti* β-fluorination to set the regio- and stereoselectivity.

To explore their reactivity and utility, we subjected a selection of *Z*-FVIs to a series of derivatizations. As there are several known reactions using vinyl- and diaryl-iodonium salts with copper catalysis,^58,59,79^ we began by investigating *Z-*FVIs for copper-catalyzed C–X bond formation. Copper can insert into either the vinyl–I(III) or the aryl–I(III) bond, as shown in Figure 4A. With the ability to alter the (aryl)-iodonium component, as shown in Figure 2, the reactivity of the *Z*-FVI can be tuned. Hence, we tested three different FVIs (**4c**, **4e**, and **3a**) in copper-catalyzed bromination (Figure 4A). With *p*-tolyl **4c**, insertion was favored in the aryl–I(III) bond. However, this reversed with ortho substitution, with mesityl **3a** giving complete selectivity for vinyl–I(III) insertion. This reactivity was extended to other *Z*-FVIs to give bromo-fluoro alkenes (**5b**–**5d**) in good yields and complete retention of *Z-*stereochemistry. There are few examples of *Z-*1,2-chlorofluoroalkenes reported in the literature,^80,81^ yet copper-mediated chlorination of **3a** gave **6a** in good yield.

**Figure 4:**
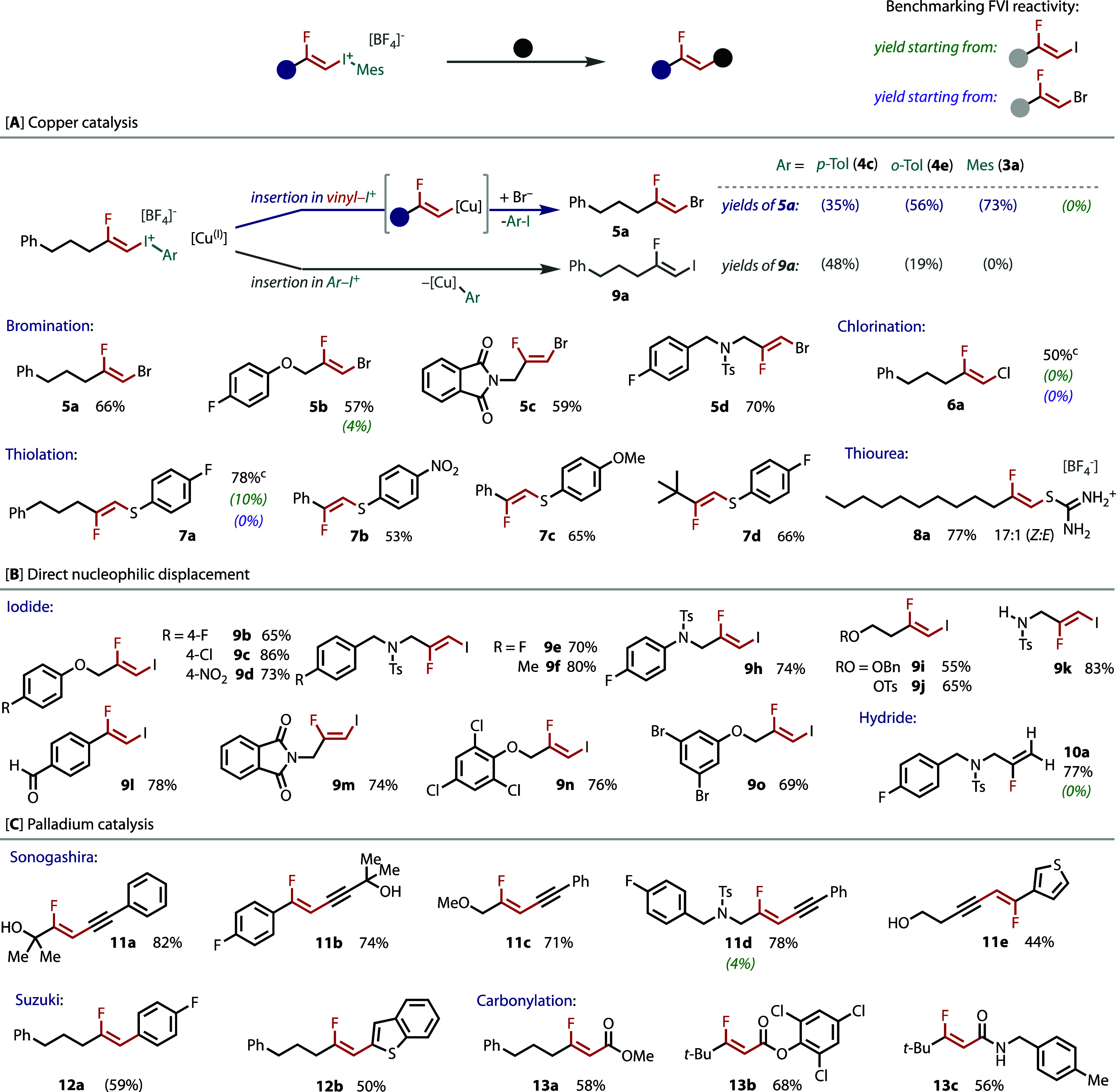
Reactivity studies and derivatization of *Z*-FVIs. Isolated yields are given with NMR yields in parentheses. See the Supporting Information for reaction conditions.

Vinyl sulfur compounds have demonstrated synthetic^82−85^ and biological^82−84^ utility, while the corresponding β-fluorovinyl sulfur compounds are very rare motifs.^40^ Hence, we developed a novel coupling reaction on the basis of copper catalysis between *Z*-FVIs with thiophenols and thioureas (see the Supporting Information for optimization) to give *Z*-thio-fluoro alkenes (**7a**–**d**) and a *Z-*fluorovinyl isothiouronium salt (**8a**). Vinyl isothiouronium salts serve as protected ene-thiol equivalents and precursors to heterocycles, sulfones, sulfoxides, and thioethers.^86,87^ However, the fluorovinyl-isothiouronium structure is unreported and, thus, should lead to fluorinated versions of these functional groups.

Direct vinylic substitution was explored by employing iodide as a nucleophile with heating to give *Z*-1,2-iodofluoro alkenes (Figure 4B). These products are underexplored organofluorine motifs, particularly when they are not styrenyl.^88^ The only reported method to these nonstyrenyl motifs involves a four-step synthesis via an FVI.^89,90^ Several iodofluoro alkenes (**9b**–**n**) were formed in high yields and could be telescoped from the FVI synthesis. Hydride displacement of iodomesitylene also furnished the hydrofluoroalkene (**10a**).

Palladium-catalyzed carbon–carbon bond-forming reactions with FVIs were tested to form internal monofluoroalkenes (Figure 4C). Sonogashira couplings with FVIs were explored to afford fluoro-enynes (**11a**–**e**). Suzuki–Miyaura arylation was also demonstrated from the FVIs. Notably, such anti-Markovnikov regioselectivity is inaccessible via hydrofluorination of the internal alkyne, which gives α-fluorostyrenes. Carbonylation reactions were conducted to access (Z)-β-fluoro-α,β-unsaturated carbonyl compounds, as highly stereoselective routes toward these compounds are underdeveloped.^91−94^ Either gaseous CO in methanol or 2,4,6-trichlorophenyl formate^95^ led to good yields of products (**13a**,**b**) with complete stereospecificity (see the Supporting Information for optimization). The trichlorophenyl ester can be derivatized, as demonstrated by one-pot carbonylation/transamination, to give amide **13c**.

The corresponding iodo(I)- or bromofluoroalkenes were tested in many of these reactions (Figure 4). They either failed or gave trace yield under the same conditions, thereby highlighting the increased reactivity and necessity of the I(III).

In conclusion, we have developed a regio- and stereoselective Ag(I)-catalyzed method for synthesizing a broad scope of *Z*-fluorovinyl iodonium (*Z*-FVIs) salts directly from unactivated alkynes. Mechanistic studies elucidate a dual role of silver to activate the alkyne and direct an *anti*-β-fluorination, which sets the regio- and stereochemistry. With an array of derivatizations, this method renders the use of *Z*-FVIs viable as versatile building blocks toward Z-monofluoroalkenes. Further explorations of their reactivity continue in our laboratory.

## Data Availability

The data underlying this study are available in the published article and its Supporting Information.

## Supporting Information Available

Experimental and computational procedures, characterization data for known and new compounds, and spectra for new compounds (PDF)
