# Supplementary material for: Silver-Catalyzed (Z)-β-Fluoro-vinyl Iodonium Salts from Alkynes: Efficient and Selective Syntheses of Z-Monofluoroalkenes
Source: J Am Chem Soc. 2024 Jun 3;146(23):15672–80. doi: 10.1021/jacs.4c03826 (PMC11177317; doi:10.1021/jacs.4c03826)
Supplement: Supplementary file 1 — ja4c03826_si_001.pdf [file ja4c03826_si_001.pdf]

Silver Catalyzed (Z)-β-fluoro-Vinyl Iodonium Salts from Alkynes: Efficient and Selective Syntheses of Z-Monofluoroalkenes

Alexi T. Sedikides, Alastair J. J. Lennox*

School of Chemistry, University of Bristol, Cantock's Close, Bristol, BS8 1TS (UK)

Corresponding author email: a.lennox@bristol.ac.uk

Contents

General Experimental Details 3
Literature Examples of Amide/Fluoroalkene Bioisosterism 5
Z-FVI Synthesis Optimisation Studies 6
Reaction Discovery and Optimisation using an 'alkyl-acetylene' substrate 6
Formation of Ar-IF_2 7
Examining the Effect of Added Base with an Aryl-acetylene Substrate 8
General Procedures for the Synthesis of Substrates 9
Amine Sulfonylation 9
Propargylation of Sulfonamides and Alcohols 9
Aryl-acetylene Synthesis 10
Synthetic Procedures and Characterisations of Substrates 11
Syntheses of Unsuccessful Alkynes 37
General Synthetic Procedure and Characterisations of Z-Fluorovinyl Iodonium Salts 44
Unsuccessful Akynes 103
Mechanistic Study (Figure 3) 104
Z-FVI Derivatisations: Reaction Procedures and Products 111
Bromination 111
Chlorination 115
Cu-Catalysed C-S Coupling 116
Thiol Coupling 116
Thiourea coupling 121
Iodination 122
Iodoalkyne Hydrofluorination Control Experiment 135
Hydride Displacement 136
Sonogashira Coupling 137
Pd-Catalysed Suzuki-Miyaura Arylation 142
Pd-Catalysed Carbonylation 144
FVI and 1,2-Halofluoroalkene Reactivity Comparison Experiments: 149
Effect of (aryl)-Iodane Structure 149
NMR Spectra of Novel Compounds 150
Computational Data 343
Natural Bond Order (NBO) Analyses 344
Alkynyl-iodonium formation 346
Stereo- and Regio-selectivity Study 366
Silver-free Fluorination of the Alkynyl-iodonium Species – Concerted Hydrofluorination 378
Fluoride Addition to the Alkynyl-iodonium - Iodonium Ylide Formation 391
References 411

General Experimental Details

Techniques

Manipulations involving air and moisture sensitive materials were conducted under a nitrogen atmosphere, employing standard glovebox and/or Schlenk-line techniques, using vacuum lines attached to a double manifold with greaseless J. Youngs valves equipped with an oil pump (0.1 mmHg) under an atmosphere of dry nitrogen. With reactions employing Schlenk lines, Youngs and Schlenk tubes were placed under a nitrogen atmosphere by 'cycling': placing the tube under vacuum and back-filling with nitrogen for three cycles. All glassware was dried by heating under vacuum to 250 °C for ~5 minutes, then allowed to cool (still under vacuum) at 0.1 mmHg. The removal of solvents under reduced pressure was achieved using a Büchi rotary evaporator (bath temperatures up to 40°C) at a pressure of 15 mmHg (diaphragm pump), or at 0.1 mmHg (oil pump) on a vacuum line at room temperature.

Solvents

For reactions employing anhydrous conditions, solvents were dried by storage over activated molecular sieves (3Å) under nitrogen, with THF (tetrahydrofuran), dichloromethane (DCM), acetonitrile (MeCN), hexane, and diethyl ether (Et_2O) dried using an Anhydrous Engineering alumina column drying system situated in the University of Bristol's chemistry department, and collected into Strauss flasks, using a gas-tight J. Youngs valve, containing activated molecular sieves. Molecular sieves were activated by heating to 300 °C under vacuum for 30 minutes, followed by cooling (still under vacuum). Deuterated solvents for NMR analysis were purchased from Sigma Aldrich.

Reagents

All reagents were purchased from TCI UK, Apollo Scientific, Sigma Aldrich, Alfa Aeser or Fluorochem and used as received.

Chromatography

TLC analysis was performed on Merck Silica gel 60F254 glass-backed plates. Visualisation was achieved by UV fluorescence (254 nm). Flash column chromatography was conducted using Fluorochem 60 silica: 230-400 mesh (40-63 µm).

Analysis

NMR spectra were recorded on Jeol ECS300 (300MHz), Bruker Nano 400 (400 MHz), Jeol ECS400 (400 MHz), Jeol ECZ400var (400 MHz), Jeol ECZ400 (400 MHz), Bruker Advance III HD 500 (500 MHz) cryo, or Varian 600 Cryo (600 MHz) spectrometers. Chemical shifts (δ) are quoted in parts per million (ppm), referenced to the residual solvent peak (^{1}H and ^{13}C NMR) and coupling constants (J) are given in Hz. Multiplicities are abbreviated as: s (singlet), d (doublet), t (triplet), q (quartet), m (multiplet) or combinations thereof. NMR shifts for novel compounds have been assigned with the use of the appropriate 2D NMR experiments, such as COSY, HSQC and HMBC. Novel compounds were also analysed by high-resolution mass spectrometry (HRMS). ^{19}F NMR measurements for the determination of NMR yields each used a 10 second relaxation delay with 16 scans. ^{19}F NMR purity assays were conducted by analysing the isolated product relative to a ^{19}F NMR standard, employing 4 scans with a 10 second relaxation delay.

Compound naming

Compound names were generated by ChemDraw Professional 20.0 (PerkinElmer) following IUPAC nomenclature.

Use of HF reagents (Warning)

The hazards of hydrogen fluoride solutions are well-categorised. Therefore, personal protection is of utmost importance. It is advised to wear two pairs of nitrile gloves when handling, and if the gloves come into contact with HF, they are removed immediately, and the area affected is washed thoroughly with water, then with Hexafluorine solutionTM. Calcium glucanoate gel is applied to the area and medical attention is sought. It is advised that Hexafluorine solutionTM and calcium glucanoate gel is kept nearby.

If HF has spilled on gloves:

Note: Time is of the essence as exposure to HF is a life-threatening emergency.

• Immediately remove gloves and wash area thoroughly with water

• If any HF has penetrated through gloves, then rub calcium glucanoate gel into the area for several minutes, reapplying once an hour for several hours.

If HF has been spilled on skin:

• Immediately wash the area with hexafluorine solution and large quantities of water for 5 minutes

• Then rub calcium gluconate gel into the area for several minutes

• Monitor area for 15 minutes and if redness and swelling develop, proceed to the closest A&E.

Literature Examples of Amide/Fluoroalkene Bioisosterism

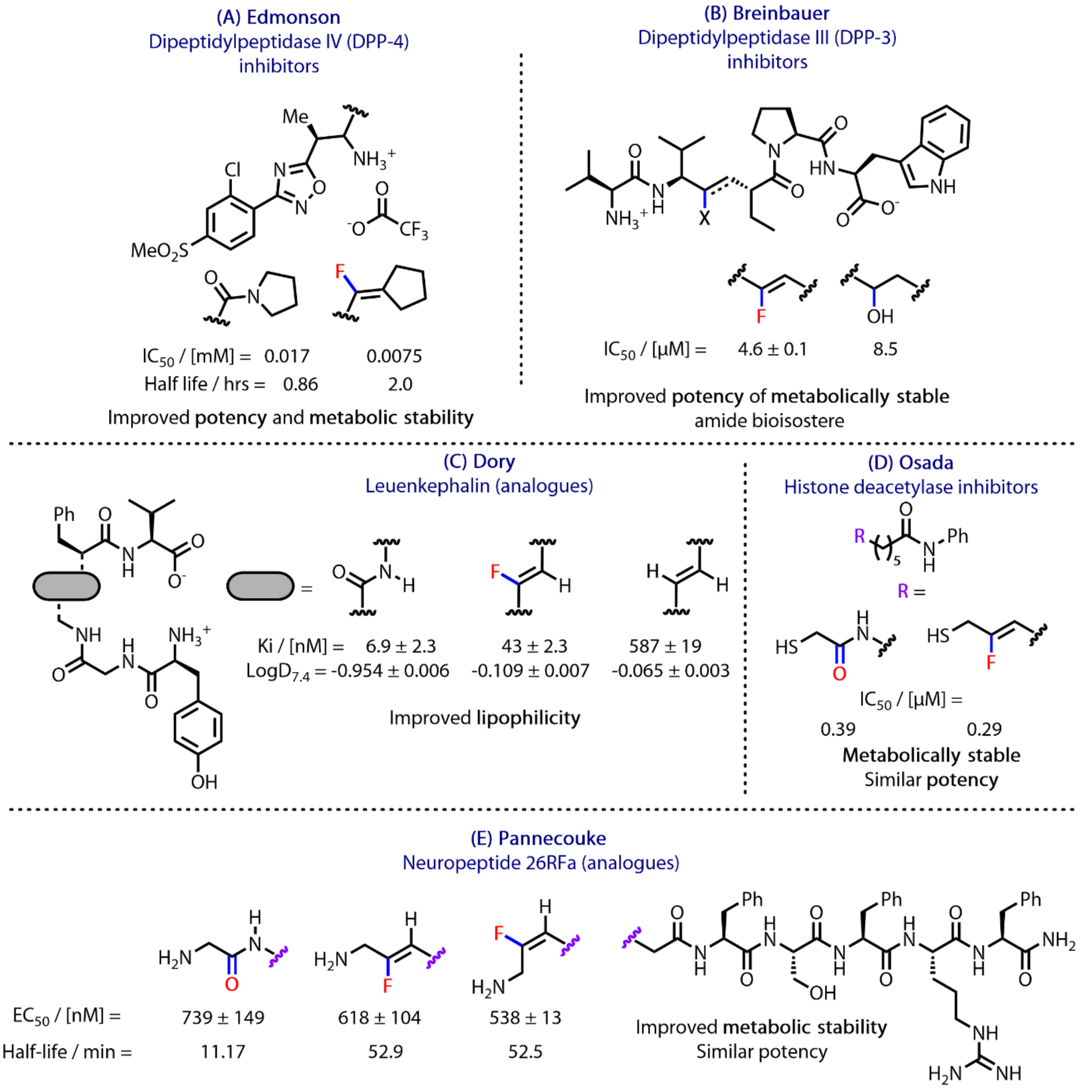

Figure S1 Amide/fluoroalkene biosisosterism examples, demonstrating improvements in lipophilicity and metabolic stability with respect to the parent amide.

Z-FVI Synthesis Optimisation Studies

Reaction Discovery and Optimisation using an 'alkyl-acetylene' substrate

To a high-density poly-ethylene (HDPE) vial equipped with a stirrer bar, was added the transition metal, 2-iodomesitylene (67 mg, 0.275 mmol, 1.1 equiv.), and oxidant (0.375 mmol, 1.5 equiv.). The vial was wrapped in aluminium foil before being cooled to 0°C. Solvent (1.77 mL) was added, followed by the drop-wise addition of pyridine.9HF (230 µL, 4 equiv./36 equiv. HF), and 5-phenyl-1-pentyne (38 µL, 0.25 mmol, 1 equiv.). The reaction mixture was allowed to warm to room temperature, and was left to stir for 24 hours. Distilled water (4 mL) and DCM (2 mL) were added to the reaction mixture, which was then transferred into a separating funnel, along with a further DCM rinse of the vial (4 mL). The aqueous and organic layers were separated, and the aqueous phase was extracted with DCM (3 x). The combined organic layers were evaporated to ~1-2 mL volume, and hexafluorobenzene was added. The mixture was analysed by ^{19}F NMR to determine NMR yields by integration relative to hexafluorobenzene.

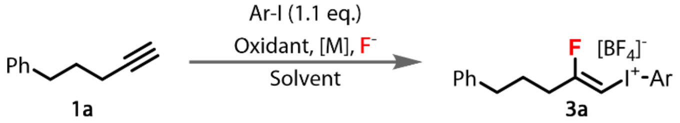

Table S1

Entry	Solvent	Additives (eq.)	F^- source (eq.)	Oxidant (eq.)	Yield / %
1	MeCN	CuTC (0.2)	Pyr.9HF (4)	Selectfluor (1.5)	0
2	DCM	CuTC (0.2)	Pyr.9HF (4)	Selectfluor (1.5)	0
3	MeCN	$CuBr_2$ (0.2)	Pyr.9HF (4)	Selectfluor (1.5)	0
4	MeCN	Cu turnings (1)	NEt_3.3HF (5.7)	Selectfluor (1.5)	0
5	MeCN	$PtBr_2$ (0.2)	Pyr.9HF (4)	Selectfluor (1.5)	0
6	DCM	$PtBr_2$ (0.2)	Pyr.9HF (4)	Selectfluor (1.5)	0
7	MeCN	$K[PtCl_4]$ (0.2)	NEt_3.3HF (3.7)	Selectfluor (1.5)	0
8	DCM	$K[PtCl_4]$ (0.2)	NEt_3.3HF (3.7)	Selectfluor (1.5)	0
9	MeCN	$FeBr_2$ (0.2)	NEt_3.3HF (3.7)	Selectfluor (1.5)	0
10	MeCN	$FeBr_2$ (0.2)	Pyr.9HF (4)	Selectfluor (1.5)	0
11	MeCN	$Fe(acac)_3$ (0.2)	Pyr.9HF (4)	*m*-CPBA (1.5)	0
12	$CHCl_3$	$[(PhP)_2CoCl_2]$ (0.1)	Pyr.9HF (4)	*m*-CPBA (1.5)	0
13	DCM	$(PPh_3)AuCl$ (0.2)	NEt_3.3HF (5.7)	Selectfluor (1.5)	0
14	DCM	$(PPh_3)AuCl$ (0.2)	Pyr.9HF (4)	Selectfluor (1.5)	0
15	MeCN	$(PPh_3)AuCl$ (0.2)	Pyr.9HF (4)	Selectfluor (1.5)	0
16	DCM	$KAuCl_4$ (0.2)	NEt_3.3HF (5.7)	Selectfluor (1.5)	0
17	DCM	Ag_2CO_3 (1)	Pyr.9HF (4)	Selectfluor (1.5)	24
18	Toluene	Ag_2CO_3 (1)	Pyr.9HF (4)	Selectfluor (1.5)	22
19	THF	Ag_2CO_3 (1)	Pyr.9HF (4)	Selectfluor (1.5)	13
20	2-MeTHF	Ag_2CO_3 (1)	Pyr.9HF (4)	Selectfluor (1.5)	11
21	TBME	Ag_2CO_3 (1)	Pyr.9HF (4)	Selectfluor (1.5)	0
22	Anisole	Ag_2CO_3 (1)	Pyr.9HF (4)	Selectfluor (1.5)	0
23	Propan-2-ol	Ag_2CO_3 (1)	Pyr.9HF (4)	Selectfluor (1.5)	0
24	Ethanol	Ag_2CO_3 (1)	Pyr.9HF (4)	Selectfluor (1.5)	3
25	*t*-Butanol	Ag_2CO_3 (1)	Pyr.9HF (4)	Selectfluor (1.5)	10
26	EtOAc	Ag_2CO_3 (1)	Pyr.9HF (4)	Selectfluor (1.5)	39
27	BuOAc	Ag_2CO_3 (1)	Pyr.9HF (4)	Selectfluor (1.5)	13
**28**	**$MeNO_2$**	**Ag_2CO_3 (1)**	**Pyr.9HF (4)**	**Selectfluor (1.5)**	**79**
29	Acetone	Ag_2CO_3 (1)	Pyr.9HF (4)	Selectfluor (1.5)	23
**30**	**DMC**	**Ag_2CO_3 (1)**	**Pyr.9HF (4)**	**Selectfluor (1.5)**	**65**
31	Water	Ag_2CO_3 (1)	Pyr.9HF (4)	Selectfluor (1.5)	0
32	$MeNO_2$	Ag_2CO_3 (0.5)	Pyr.9HF (4)	Selectfluor (1.5)	45
33	$MeNO_2$	$AgBF_4$ (1)	Pyr.9HF (4)	Selectfluor (1.5)	11
34	$MeNO_2$	$AgBF_4$ (1), Cs_2CO_3 (1)	Pyr.9HF (4)	Selectfluor (1.5)	65
35	$MeNO_2$	AgF (1)	Pyr.9HF (4)	Selectfluor (1.5)	22
36	$MeNO_2$	AgF (1), Cs_2CO_3 (1)	Pyr.9HF (4)	Selectfluor (1.5)	30
37	$MeNO_2$	AgOTf (1)	Pyr.9HF (4)	Selectfluor (1.5)	12
38	$MeNO_2$	Ag(I) trifluoroacetate (1)	Pyr.9HF (4)	Selectfluor (1.5)	25
39	$MeNO_2$	$AgNO_3$ (1)	Pyr.9HF (4)	Selectfluor (1.5)	1
40	$MeNO_2$	$AgPF_6$ (1)	Pyr.9HF (4)	Selectfluor (1.5)	7
41	$MeNO_2$	None	Pyr.9HF (4)	Selectfluor (1.5)	0
42	$MeNO_2$	K_2CO_3 (1)	Pyr.9HF (4)	Selectfluor (1.5)	0
**43**	**$MeNO_2$**	**Ag_2CO_3 (0.1), K_2CO_3 (3)**	**Pyr.9HF (4)**	**Selectfluor (1.5)**	**79**
**44**	**$MeNO_2$**	**Ag_2CO_3 (0.1)**	**Pyr.9HF (4)**	**Selectfluor (1.5)**	**0**
45	$MeNO_2$	Ag_2CO_3 (0.1), K_2CO_3 (3)	Pyr.9HF (4)	NFSI (1.5)	0
46	$MeNO_2$	Ag_2CO_3 (0.1), K_2CO_3 (3)	Pyr.9HF (4)	*N*-Fluoro pyridinium BF_4 (1.5)	0

47	$MeNO_2$	Ag_2CO_3 (0.1), K_2CO_3 (3)	Pyr.9HF (4)	*Tert*-Butyl peroxide (1.5)	0
48	$MeNO_2$	Ag_2CO_3 (0.1), K_2CO_3 (3)	Pyr.9HF (4)	Oxone (1.5)	0
49	$MeNO_2$	Ag_2CO_3 (0.1), K_2CO_3 (3)	Pyr.9HF (4)	*m*-CPBA (1.5)	68
50	$MeNO_2$	Ag_2CO_3 (0.1), K_2CO_3 (3)	Pyr.9HF (1)	Selectfluor (1.5)	45

Formation of Ar-IF$_2$

To an HDPE vial, was added Ag_2CO_3 (35.4 mg, 0.125 mmol, 0.1 equiv.), 2-iodomesitylene (338 mg, 1.375 mmol, 1.1 equiv.), and MeCN (8.82 mL). The mixture was cooled to 0°C, and pyridine.9HF (1.18 mL) was added dropwise, and an aliquot of the reaction was analysed by ^{1}H NMR. Selectfluor (664 mg, 1.875 mmol, 1.5 equiv.) was added, the mixture was stirred further for 30 minutes, allowing warming to room temperature, and another ^{1}H NMR aliquot was taken.

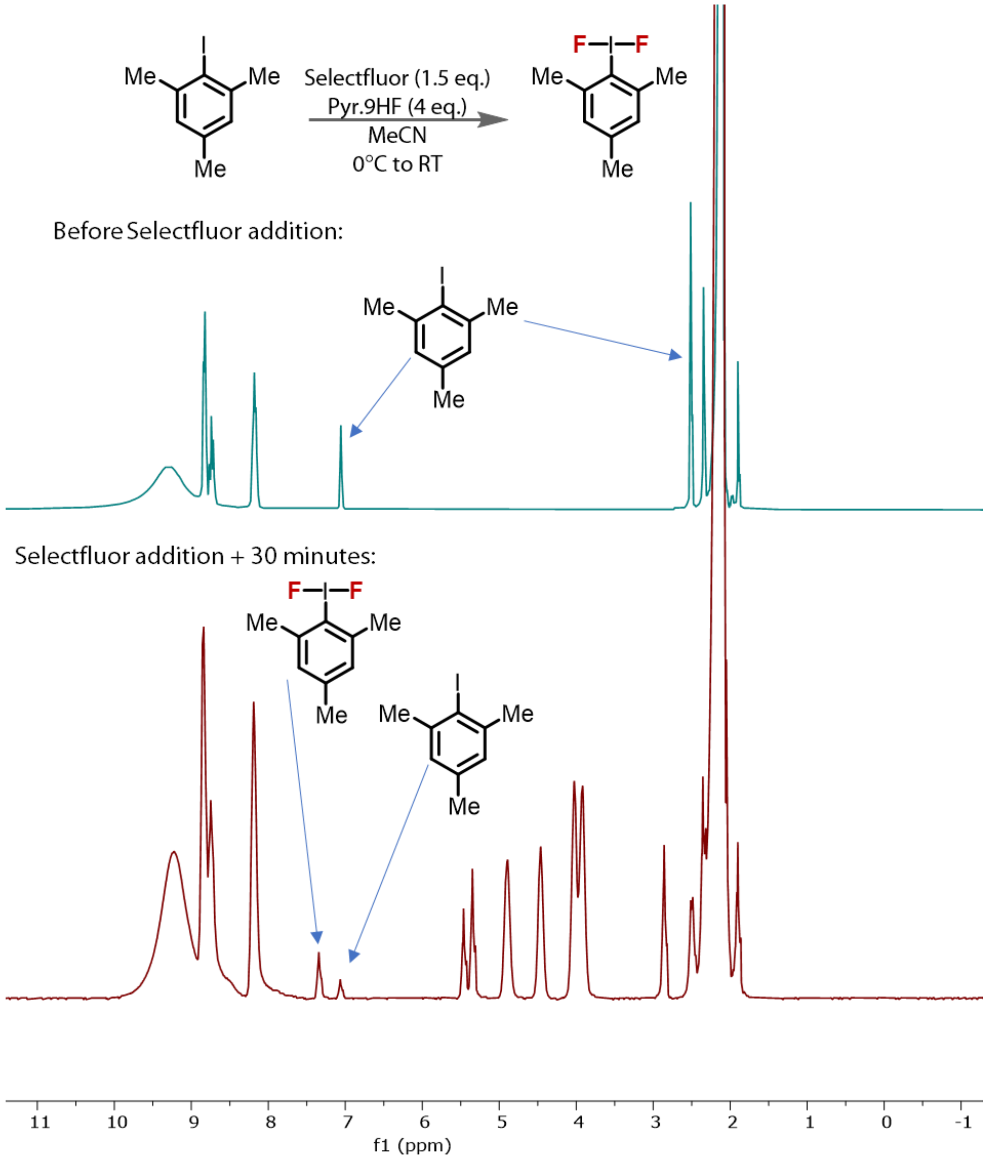

Examining the Effect of Added Base with an Aryl-acetylene Substrate

To an HDPE vial equipped with a stirrer bar, was added the silver salt (0.025 mmol, 1 equiv.), 2-iodomesitylene (67 mg, 0.275 mmol, 1.1 equiv.), oxidant (1.375 mmol, 1.5 equiv.), and additive (x equiv.) (liquid additives were added immediately after $MeNO_2$). The vial was wrapped in aluminium foil before being cooled to 0◦C. Nitromethane (1.77 mL) was added, followed by the drop-wise addition of pyridine.9HF (230 μL, 4 equiv. 36 equiv.). The reaction mixture was stirred for 5 minutes before the addition of 4-fluorophenylacetylene (28.7 μL, 0.25 mmol, 1 equiv.). The reaction mixture was allowed to warm to room temperature and was left to stir for 24 hours. Distilled water (4 mL) and dimethyl carbonate (2 mL) was added to the reaction mixture, which was then transferred into a separating funnel along with a further rinse of the vial with dimethyl carbonate (4 mL). The aqueous and organic layers were separated, and the aqueous phase was extracted with dimethyl carbonate (2 x). The combined organic layers were evaporated under reduced pressure to 1-2 mL volume, and hexafluorobenzene was added. The mixture was then analysed by ^{19}F NMR to determine NMR yields and remaining starting material by integration relative to hexafluorobenzene.

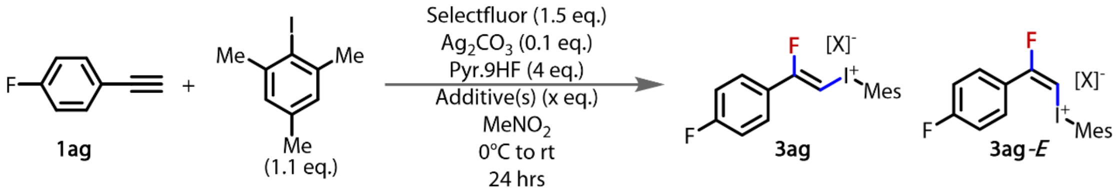

Table S2

Entry	Variation(s)	RSM / %	Yield (NMR) / \% (3ag, 3ag-*E*)
1	None (36 eq. HF)	0	8, 10
2	No [Ag]	52	1, 6
3	K_2CO_3 (1 eq.)	1	42, 14
4[A]	Pyridine (2 eq.)	0	44, 13
-[B]	-	0	42, 11
5	NEt_3 (2 eq.)	3	59, 19
6	K_2CO_3 (2 eq.)	1	89, 9
7	Pyr.9HF (30 eq. HF)	0	<1, 9
8	Pyr.9HF (30 eq. HF), KF (6 eq.)	0	49, 15
9	Oxalic acid (4 eq.)	11	5,6
10	TFA (4 eq.)	0	5, 8

[A]NMR yield measured directly from the reaction mixture, without conducting the above-described workup. [B] Results from the workup of the reaction in entry 4, to indicate minimal loss of product/mass balance.

General Procedures for the Synthesis of Substrates

Amine Sulfonylation

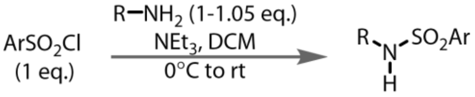

To a stirring solution of the sulfonyl chloride (1 equiv.) in DCM at 0°C, was added the amine (1 - 1.05 equiv.) slowly. Triethylamine (1.2-1.5 equiv.) was added drop-wise, and the reaction was warmed to room temperature. When the sulfonyl chloride had been fully consumed (as indicated by TLC), water was added. The aqueous and organic layers were separated, and the aqueous layer was extracted with DCM (3 x). The combined organic extracts were washed with 1M HCl (aqueous) and brine, before being dried over $MgSO_4$, filtered, and concentrated under reduced pressure. Unless otherwise specified, no further purification was required.

Propargylation of Sulfonamides and Alcohols

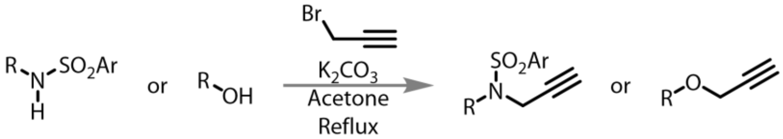

Procedure 1A

To a stirring solution of amine or alcohol and propargyl bromide (80% in toluene) in either MeCN or acetone, was added K_2CO_3 .The suspension was stirred under reflux for 24 hours, before being cooled to room temperature and filtered through cotton. The filtrate was concentrated under reduced pressure. The resulting crude mixture was dissolved in DCM and washed with water. The aqueous phase was back-extracted with DCM (3 x). The combined organic extracts were dried over $MgSO_4$, filtered, and concentrated under reduced pressure. The resulting crude mixture was subjected to flash-column chromatography.

Procedure 1B

To a stirring solution of amine or alcohol and propargyl bromide (80% in toluene) in acetone, was added K_2CO_3. The suspension was stirred under reflux for 24 hours, then cooled to room temperature and evaporated under reduced pressure. Water and DCM were added, and the mixture was transferred to a separating funnel. The organic and aqueous layers were separated, and the aqueous phase was extracted further with DCM (2 x). The combined organic extracts were dried over $MgSO_4$, filtered, and concentrated under reduced pressure. The resulting crude mixture was subjected to flash-column chromatography.

Aryl-acetylene Synthesis

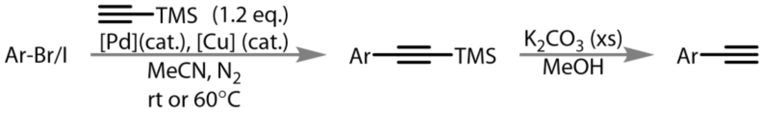

To a Schlenk tube equipped with a stirrer bar, was added CuI, aryl-halide and a palladium source (either bis(triphenylphosphine)palladium chloride, or $Pd(OAc)_2$ with triphenylphospine (0.06 equiv.). The tube was placed under vacuum and back-filled with nitrogen for three cycles. A degassed mixture of acetonitrile and NEt_3 (3:1) were added, followed immediately by ethynyltrimethylsilane. The reaction was stirred at room temperature when using an aryl iodide, or heated to 60 °C when using an aryl bromide. Upon complete consumption of the aryl-halide (monitoring by TLC), the mixture was poured into a saturated aqueous solution of ammonium chloride, along with a DCM rinse of the Schlenk tube, and the mixture was extracted with DCM (3 x). The combined organic extracts were washed with brine, filtered, and concentrated under reduced pressure. The resulting crude mixture was either passed through a short pad of silica and used directly in the subsequent step, or subjected to flash-column chromatography. The trimethylsilyl-alkyne from the previous step was added to a stirring suspension of K_2CO_3 (2 equiv.) in methanol. After full consumption of the trimethylsilyl-alkyne (monitoring by TLC), the solvent was evaporated under reduced pressure. Water was added and the mixture was extraced with DCM (3 x). The combined organic extracts were dried over $MgSO_4$, filtered, and concentrated under reduced pressure. Purification by flash-column chromatography was employed where specified.

Synthetic Procedures and Characterisations of Substrates

((But-3-yn-1-yloxy)methyl)benzene **(1g)**

This substrate was synthesised according to a modified literature procedure.[1] To an oven-dried two-neck round-bottom flask equipped with a stirrer bar, under nitrogen, was added sodium hydride (400 mg, 60% in mineral oil, 10 mmol, 2 equiv.) and dry THF (20 mL). The mixture was cooled to 0°C, 3-butyn-1-ol (378 µL, 5 mmol, 1 equiv.) was added, and the mixture stirred for 20 minutes. Tetrabutylammonium iodide (185 mg, 0.5 mmol, 0.1 equiv.) and benzyl bromide (654 µL, 5.5 mmol, 1.1 equiv.) were added. The mixture was allowed to warm to room temperature and stirred for 18 hours. Brine was added (10 mL), and the mixture was extracted with DCM (3 x 15 mL). The combined organic extracts were filtered and concentrated under reduced pressure. The resulting crude mixture was subjected to flash-column chromatography (0 to 10% EtOAc in pentane) to afford the product as a colourless oil (730 mg, 91%).

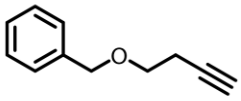

^{1}H NMR (500 MHz, $CDCl_3$): δ 8.14 – 7.99 (2H, m), 7.60 – 7.53 (1H, m), 7.49 – 7.40 (2H, m), 4.43 (2H, t, *J* = 6.8 Hz), 2.67 (2H, td, *J* = 6.8, 2.7 Hz), 2.03 (1H, t, *J* = 2.7 Hz)

^{13}C NMR (126 MHz, $CDCl_3$): δ 166.4, 133.2, 130.1, 129.8, 128.5, 80.2, 70.1, 62.7, 19.2.

These data are consistent with those previously reported.[2]

But-3-yn-1-yl 4-methylbenzenesulfonate **(1h)**

This substrate was synthesised according to an adapted literature procedure.[3] To a round-bottom flask equipped with a stirrer bar, was added 3-butynol (3.24 mL, 42.8 mmol, 1.0 equiv), DMAP (51.3 mg, 0.42 mmol, 0.01 equiv.), NEt_3 (7.7 mL, 55.4 mmol, 1.3 equiv.), and DCM (15 ml). The solution was cooled to 0°C, and *p*-TsCl (8.97 g, 47.1 mmol, 1.1 equiv.) was added as a solution in DCM (15 mL). The reaction mixture warmed to room temperature and left to stir for 18 hours before adding water (30 mL). The aqueous and organic layers were separated, and the aqueous phase was extracted with DCM (3 x 40 mL). The combined organic layers were washed with brine (60 mL), dried over $MgSO_4$, and concentrated under reduced pressure to afford a crude mixture as a dark red oil. The resulting crude mixture was subjected to flash-column chromatography (20 to 30% Et_2O in pentane) to yield a colourless oil (2.6 g, 27%).

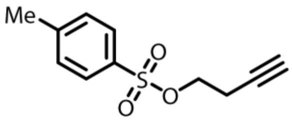

^{1}H NMR (500 MHz, $CDCl_3$): δ 7.81 (2H, d, *J* = 8.4 Hz), 7.35 (2H, d, *J* = 8.1 Hz), 4.10 (2H, t, *J* = 7.1 Hz), 2.56 (2H, td, *J* = 7.1, 2.7 Hz), 2.45 (3H, s), 1.97 (1H, t, *J* = 2.7 Hz).

^{13}C NMR (126 MHz, $CDCl_3$): δ 145.1, 133.0, 130.0, 128.1, 78.5, 70.9, 67.6, 21.8, 19.6.

These data are consistent with those previously reported. [3]

1-Bromo-4-((prop-2-yn-1-yloxy)methyl)benzene **(1i)**

To an oven-dried Schlenk tube equipped with a stirrer bar with a stirrer bar, under nitrogen, was added sodium hydride (800 mg, 60% in mineral oil, 20 mmol) and dry THF (40 mL). The mixture was cooled to 0°C. (4-Bromophenyl)methanol (1.87 g, 10 mmol, 1 equiv.) was added, and left to stir for 20 minutes. Propargyl bromide (1.42 mL, 13.2 mmol, 1.32 equiv., 80% wt in toluene) was added, the reaction was allowed to warm to room temperature, and stirred for a further 18 hours. Brine was added (10 mL), and the mixture was extracted with DCM (3 x 15 mL). The combined organic extracts were filtered and concentrated under reduced pressure. The resulting crude mixture was subjected to flash-column chromatography (0 to 20% EtOAc in pentane) to afford the product as a yellow oil (1.56 g, 69%).

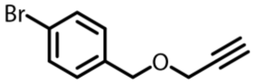

^{1}H NMR (500 MHz, $CDCl_3$): δ 7.51 – 7.45 (2H, m), 7.25 – 7.21 (2H, m), 4.56 (2H, s), 4.17 (2H, d, *J* = 2.4 Hz), 2.48 (1H, t, *J* = 2.4 Hz).

^{13}C NMR (126 MHz, $CDCl_3$): δ 136.5, 131.7, 129.8, 122.0, 79.5, 75.0, 70.9, 57.4.

These data are consistent with those previously reported.[4]

Prop-2-yn-1-yl methanesulfonate **(1l)**

To a stirring solution of prop-2-yn-1-ol (1.16 mL, 20 mmol, 1 equiv.) in DCM (40 mL), in a round-bottom flask cooled to 0°C, was added methanesulfonyl chloride (2.29 mL, 29 mmol, 1 equiv.) and NEt_3 (2.79 mL, 20 mmol, 1 equiv.). After stirring for one hour, 1M HCl (aq.) (20 mL) was added. The aqueous and organic layers were separated, and the aqueous phase was extracted with DCM (2 x 15 mL). The combined organic extracts were washed with brine (20 mL), before being dried over $MgSO_4$, filtered, and concentrated under reduced pressure. The resulting crude mixture was subjected to flash-column chromatography (20% EtOAc in pentane) to afford the product as a colourless oil (1.88 g, 70%).

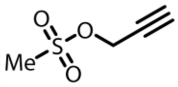

^{1}H NMR (500 MHz, $CDCl_3$): δ 4.85 (2H, d, *J* = 2.5 Hz), 3.13 (3H, s), 2.70 (1H, t, *J* = 2.5 Hz).

^{13}C NMR (500 MHz, $CDCl_3$): δ 78.0, 75.9, 57.3, 39.2.

These data are consistent with those previously reported.[5]

Prop-2-yn-1-yl 4-methylbenzenesulfonate **(1m)**

This substrate was synthesised according to a modified literature procedure.[6] To a stirring solution of prop-2-yn-1-ol (3.5 mL, 60 mmol, 1 equiv.) and *p*-TsCl (13.8 g, 72 mmol, 1.2 equiv) in Et_2O (60 mL), in a round-bottom flask cooled to 0°C, was added potassium hydroxide (16.8 g, 300 mmol, 5 equiv.). The suspension was slowly warmed to room-temperature and left to stir for 18 hours, followed by the addition of water (30 mL). The aqueous and organic layers were separated. The aqueous phase was extracted with Et_2O (2 x 30 mL). The combined organic extracts were washed with brine (50 mL), before being dried over $MgSO_4$, filtered, and concentrated under reduced pressure to afford the product as a colourless oil (8.0 g, 63%).

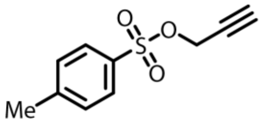

^{1}H NMR (500 MHz, $CDCl_3$): δ 7.96 – 7.70 (2H, m), 7.46 – 7.30 (2H, m), 4.69 (2H, d, J = 2.5 Hz), 2.47 (1H, t, J = 2.5 Hz), 2.45 (3H, s).

^{13}C NMR (126 MHz, $CDCl_3$): δ 145.4, 133.0, 130.0, 128.3, 77.4, 75.5, 57.5, 21.8.

These data are consistent with those previously reported. [6]

1-Nitro-4-(prop-2-yn-1-yloxy)benzene **(1n)**

Synthesised according to Propargylation Procedure 2 using 4-nitrophenol (696 mg, 5 mmol, 1 equiv.), propargyl bromide (1.12 mL, 80% in toluene, 10.5 mmol, 2.1 equiv.), and K_2CO_3 (1.38 g, 10 mmol, 2 equiv.), in acetone (25 mL). The product was obtained after workup, without the need for column chromatography (770 mg, 87%).

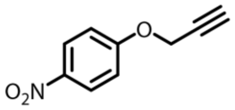

^{1}H NMR (500 MHz, CDCl$_3$): δ 8.28 – 8.17 (2H, m), 7.10 – 7.01 (2H, m), 4.80 (2H, d, *J* = 2.4 Hz), 2.58 (1H, t, *J* = 2.4 Hz).

^{13}C NMR (126 MHz, CDCl$_3$): δ 162.3, 142.2, 125.9, 115.0, 77.2, 77.1, 56.3.

These data are consistent with those previously reported.[7]

1-Fluoro-4-(prop-2-yn-1-yloxy)benzene **(1o)**

Synthesised according to a modified version of Propargylation Procedure 1 using 4-fluorophenol (3.36 g, 30 mmol, 1 equiv.), propargyl bromide (3.45 mL, 80% in toluene, 36 mmol, 1.21 equiv.), and K_2CO_3 (10 g, 72.4 mmol, 2.42 equiv.), in acetone (150 mL). Purified by flash-column chromatography (10 to 20% EtOAc in pentane) to yield a light-yellow oil (3.03 g, 67% yield).

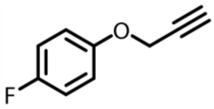

^{1}H NMR (500 MHz, $CDCl_3$): δ 7.05 – 6.96 (2H, m), 6.96 – 6.86 (2H, m), 4.66 (2H, d, *J* = 2.6 Hz), 2.52 (1H, t, *J* = 2.5 Hz).

^{13}C NMR (126 MHz, $CDCl_3$): δ 157.9 (d, *J* = 239.3 Hz), 153.8 (d, *J* = 2.2 Hz), 116.3 (d, *J* = 8.0 Hz), 116.0 (d, *J* = 23.2 Hz), 78.6, 75.8, 56.6.

^{19}F NMR (376 MHz, $CDCl_3$): δ -122.73 (1F, m, *J* = 8.2, 4.4 Hz).

These data are consistent with those previously reported.[8]

1-Chloro-4-(prop-2-yn-1-yloxy)benzene **(1p)**

Synthesised according to Propargylation Procedure 2 using 4-chlorophenol (1.29 g, 10 mmol, 1 equiv.), propargyl bromide (2.23 mL, 80% in toluene, 20.8 mmol, 2.1 equiv.), and K_2CO_3 (2.76 g, 20 equiv.), in acetone (50 mL). Purified by flash-column chromatography (4% EtOAc in pentane) to yield a colourless oil (1.18 g, 71% yield).

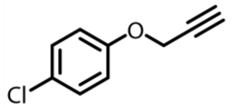

^{1}H NMR (500 MHz, $CDCl_3$): δ 7.31 – 7.24 (2H, m), 6.96 – 6.88 (2H, m), 4.68 (2H, d, *J* = 2.4 Hz), 2.54 (1H, t, *J* = 2.4 Hz).

^{13}C NMR (126 MHz, $CDCl_3$): δ 156.2, 129.5, 126.7, 116.4, 78.3, 76.0, 56.2

These data are consistent with those previously reported.[9]

1-Bromo-4-(prop-2-yn-1-yloxy)benzene **(1q)**

Synthesised according to Propargylation Procedure 2 using 4-bromophenol (865 mg, 5 mmol, 1 equiv.), propargyl bromide (1.12 mL, 80% in toluene, 10.5 mmol, 2.1 equiv.), and K_2CO_3 (1.38 g, 10 mmol, 2 equiv.), in acetone (25 mL). The product was obtained after workup, without the need for column chromatography, as a yellow oil (1.05 g, quant.).

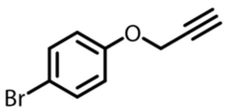

^{1}H NMR (500 MHz, $CDCl_3$): δ 7.44 – 7.37 (2H, m), 6.90 – 6.84 (2H, m), 4.67 (2H, d, *J* = 2.4 Hz), 2.53 (1H, t, *J* = 2.4 Hz).

^{13}C NMR (126 MHz, $CDCl_3$): δ 156.6, 132.3, 116.8, 113.9, 78.1, 75.9, 56.0.

These data are consistent with those previously reported.[10]

1,3-Dibromo-5-(prop-2-yn-1-yloxy)benzene ***(1t)***

Synthesised according to Propargylation Procedure 2 using 3,5-dibromophenol (1.26 g, 5 mmol, 1 equiv.), propargyl bromide (1.12 mL, 80% in toluene, 10.5 mmol, 2.1 equiv), and K_2CO_3 (1.38 g, 10 mmol, 2 equiv.), in acetone (25 mL). The product was obtained after workup, without the need for column chromatography, as a brown solid (1.38 g, 95%).

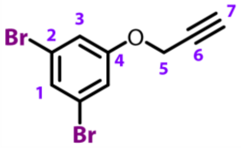

^{1}H NMR (500 MHz, $CDCl_3$): δ 7.30 (1H, t, *J* = 1.6 Hz, H^1), 7.08 (2H, d, *J* = 1.6 Hz, H^3), 4.67 (2H, d, *J* = 2.4 Hz, H^5), 2.57 (1H, t, *J* = 2.4 Hz, H^7).

^{13}C NMR (126 MHz, $CDCl_3$): δ 158.7 (1C, s, C^4), 127.5 (1C, s, C^1), 123.3 (2C, s, C^2), 117.6 (2C, s, C^3), 77.5 (1C, s, C^6), 76.7 (1C, d, *J* = 1.8 Hz, C^7), 56.4 (1C, s, C^5).

HRMS (EI) calc: $[M – H]^+$ ($C_9H_6OBr_2$) 286.8702; measured: 286.8700 = 0.70 ppm difference.

IR (neat) v_{max}**/cm^{-1}:** 3680, 3270, 3079, 1583, 1559, 1436, 1418, 1379, 1256, 1230, 867, 828, 746, 704, 687, 665.

1,3,5-Trichloro-2-(prop-2-yn-1-yloxy)benzene ***(1u)***

Synthesised according to Propargylation Procedure 2 using 2,4,6-trichlorophenol (1.97 g, 10 mmol, 1 equiv.), propargyl bromide (2.24 mL, 80% in toluene, 21 mmol, 2.1 equiv.), and K_2CO_3 (2.76 g, 20 mmol, 2 equiv.), in acetone (50 mL). The product was obtained after workup, without the need for column chromatography, as a white solid (1.71 g, 73%).

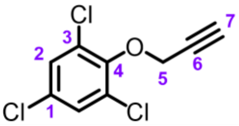

^{1}H NMR (500 MHz, $CDCl_3$): δ 7.32 (2H, s, H^2), 4.77 (2H, d, J = 2.5 Hz, H^5), 2.54 (1H, t, J = 2.5 Hz, H^7).

^{13}C NMR (126 MHz, $CDCl_3$): δ 149.2 (1C, s, C^4), 130.7 (2C, s, C^3), 130.4 (1C, s, C^1), 128.9 (2C, s, C^2), 77.6 (1C, s, C^6), 76.7 (1C, s, C^7), 60.7 (1C, s, C^5).

HRMS (EI) calc: $[M]^+$ ($C_9H_5OCl_3$) 233.9400; measured: 233.9399 = 0.43 ppm difference.

IR (neat) ν_{max}/cm^{-1}: 3289, 1553, 1456, 1431, 1371, 1255, 1138, 984, 955, 860, 800, 765, 683, 651, 575, 428.

1,3-Dimethyl-2-(prop-2-yn-1-yloxy)benzene **(3v)**

Synthesised according to Propargylation Procedure 2 using 2,6-dimethylphenol (611 mg, 5 mmol, 1 equiv.), propargyl bromide (1.12 mL, 80% in toluene, 10.5 mmol, 2.1 equiv.), and K_2CO_3 (1.38 g, 10 mmol, 2 equiv.), in acetone (25 mL). The procedure was repeated, and both crude mixtures obtained after work-up were combined and subjected to flash-column chromatography (2% EtOAc in pentane) to obtain the product as colourless oil (498 mg, 62% yield).

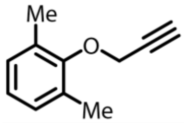

^{1}H NMR (500 MHz, CDCl$_3$): δ 7.02 (2H, dq, *J* = 7.2, 0.8 Hz), 6.95 (1H, dd, *J* = 8.3, 6.6 Hz), 4.51 (2H, d, *J* = 2.4 Hz, 1H), 2.51 (1H, t, *J* = 2.5 Hz), 2.33 (6H, s)

^{13}C NMR (126 MHz, CDCl$_3$): δ 155.4, 131.3, 129.0, 124.6, 79.5, 75.0, 59.9, 16.6

These data are consistent with those previously reported.[11]

N-(4-fluorobenzyl)-4-nitro-N-(prop-2-yn-1-yl)aniline ***(1y)***

N-(4-fluorobenzyl)-4-nitrobenzenesulfonamide: Synthesised according to Amine Sulfonylation Procedure using 4-fluorobenzylamine (1.71 mL, 15 mmol, 1.05 equiv.), *p*-nitrobenzenesulfonyl chloride (3.16, 14.25 mmol, 1 equiv.), and NEt_3 (2.3 mL, 16.5 mmol, 1.16 equiv.). The product was obtained after workup, without further purification, as a light yellow solid (3.87 g, 87%).

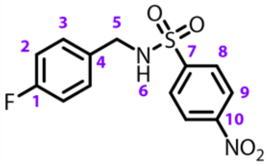

^{1}H NMR (500 MHz, CD_3CN): δ 8.29 (2H, d, *J* = 8.9 Hz, H^9), 7.96 (2H, d, *J* = 8.8 Hz, H^8), 7.20 (2H, dd, *J* = 8.5, 5.5 Hz, H^2), 6.99 (2H, t, *J* = 8.8 Hz, H^3), 6.31 (1H, t, *J* = 6.5 Hz, H^6), 4.12 (2H, d, *J* = 6.1 Hz, H^5).

^{13}C NMR (126 MHz, CD_3CN): δ 163.1 (1C, d, *J* = 243.8 Hz, C^1), 151.1 (1C, s, C^{10}), 147.3 (1C, s, C^7), 134.0 (d, *J* = 3.1 Hz, C^4), 130.9 (2C, d, *J* = 8.3 Hz, C^3), 129.2 (2C, s, C^8), 125.3 (2C, s, C^9), 116.1 (2C, d, *J* = 21.7 Hz, C^2), 46.9 (1C, s, C^5).

^{19}F NMR (376 MHz, CD_3CN): δ -116.48 – -116.66 (1F, m, F^1).

HRMS (ESI) calc: $[M]^-$ ($C_{13}H_{12}N_2O_4SF$) 310.0429; measured: 310.0424 = 1.61 ppm difference.

IR (neat) v_{max}/cm^{-1}: 3270, 1523, 1509, 1411, 1350, 1308, 1219, 1160, 1092, 1048, 923, 858, 836, 769, 738, 685, 569, 524, 504, 465.

N-(4-fluorobenzyl)-4-nitro-N-(prop-2-yn-1-yl)aniline: Synthesised according to Propargylation Procedure 1 using *N*-(4-fluorobenzyl)-4-nitrobenzenesulfonamide (1.63 g, 5.24 mmol, 1 equiv.), propargyl bromide (1 mL, 80% in toluene, 9.34 mmol, 1.78 equiv.), and K_2CO_3 (1.45 g, 10.48, equiv.), in acetone (50 mL). Purified by flash-column chromatography (10 to 30 % Et_2O in pentane) to yield a yellow solid (900 mg, 49% yield).

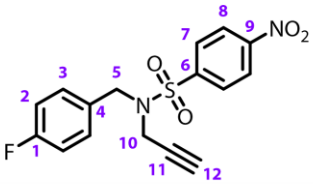

^{1}H NMR (500 MHz, CD_3CN): δ 8.38 (2H, d, *J* = 8.9 Hz, H^7), 8.24 – 7.96 (2H, m, H^8), 7.46 – 7.25 (2H, m, H^3), 7.15 – 7.06 (2H, m, H^2), 4.40 (2H, s, H^5), 3.97 (2H, d, *J* = 2.4 Hz, H^{10}), 2.33 (1H, t, *J* = 2.5 Hz, H^{12}).

^{13}C NMR (126 MHz, CD_3CN): δ 163.5 (d, *J* = 244.3 Hz, C^1), 151.6 (1C, s, C^9), 145.3 (1C, s, C^6), 132.1 (d, *J* = 3.2 Hz, C^4), 131.6 (d, *J* = 8.4 Hz, C^3), 130.1 (2C, s, C^8), 125.4 (2C, s, C^7), 116.5 (d, *J* = 21.8 Hz, C^2), 76.5 (1C, s, C^{12}), 76.2 (1C, s, C^{11}), 50.5 (1C, s, C^5), 37.0 (1C, s, C^{10}).

^{19}F NMR (376 MHz, CD_3CN): δ -115.67 – -115.82 (1F, m, F^1).

HRMS (ESI) calc: $[M+Na]^+$ ($C_{16}H_{13}FN_2NaO_4S$) 371.0472; measured: 371.0464 = 2.2 ppm difference.

IR (neat) v_{max}/cm^{-1}: 3680, 3275, 2980, 2902, 1608, 1529, 1512, 1351, 1307, 1223, 1164, 1090.

2-(Prop-2-yn-1-yl)isoindoline-1,3-dione **(1z)**

This substrate was synthesised according to a literature procedure.[12] To a round-bottomed flask, was added phthalimide (3.04 g, 20.6 mmol, 1 equiv.), K_2CO_3 (4.28 g, 31 mmol, 1.5 equiv.), CH_3CN (50 mL), and propargyl bromide (4.6 mL, 80% wt in toluene, 43.0 mmol, 2.09 equiv.), and a stirrer bar. After stirring for 24 hours at reflux, the hot reaction mixture was filtered through Celite and washed with CH_3CN (3 x 15 mL). The filtrate was evaporated under reduced pressure, re-dissolved in a minimum volume of DCM, and precipitated with pentane. The solid was washed with pentane (3 x), affording a yellow solid (3.19 g, 84% yield).

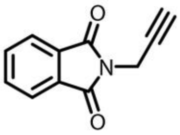

^{1}H NMR (500 MHz, $CDCl_3$): δ 7.89 (2H, dd, *J* = 5.4, 3.1 Hz), 7.74 (2H, dd, *J* = 5.5, 3.0 Hz), 4.46 (2H, d, *J* = 2.5 Hz), 2.22 (1H, t, *J* = 2.5 Hz).

^{13}C NMR (126 MHz, $CDCl_3$): δ 167.1, 134.4, 132.2, 123.7, 77.3, 71.6, 27.2.

These data are consistent with those previously reported.[12]

N-cyclopropyl-4-methyl-N-(prop-2-yn-1-yl)benzenesulfonamide ***(1aa)***

Synthesised according to Propargylation Procedure 2 using *N*-tosyl cyclopropylamine (1.06 g, 5 mmol, 1 equiv.), propargyl bromide (1.12 mL, 80% in toluene, 10.5 mmol, 2.1 equiv.), and K_2CO_3 (1.38 g, 10 mmol, 2 equiv.), in acetone (25 mL). The product was obtained after workup, without the need for column chromatography, as a cream solid (1.24 g, quant.).

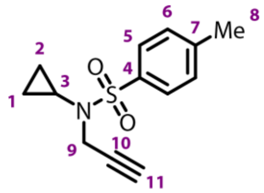

^{1}H NMR (500 MHz, CDCl$_3$): δ 7.81 (2H, d, *J* = 8.3 Hz, H^5), 7.34 – 7.29 (2H, m, H^6), 4.12 (2H, d, *J* = 2.5 Hz, H^9), 2.43 (3H, s, H^8), 2.20 (1H, tt, *J* = 7.0, 3.6 Hz, H^3), 2.00 (1H, t, *J* = 2.5 Hz, H^{11}), 1.02 – 0.88 (2H, m, H^{1+2}), 0.84 – 0.70 (2H, m, H^{1+2}).

^{13}C NMR (126 MHz, CDCl$_3$): δ 143.8 (1C, s, C^7), 135.1 (1C, s, C^4), 129.4 (2C, s, C^6)), 128.6 (2C, s, C^5), 77.2 (1C, s, C^{10}), 73.6 (1C, s, C^{11}), 39.9 (1C, s, C^9), 29.4 (1C, s, C^3), 21.7 (1C, s, C^8), 7.9 (2C, s, C^{1+2}).

HRMS (ESI) calc: [M+H]+ ($C_{13}H_{15}FO_2S$) 250.0896; measured: 250.0893 = 1.2 ppm difference.

IR (neat) v_{max}**/cm^{-1}:** 3255, 1596, 1422, 1367, 1342, 1331, 1299, 1222, 1160, 1103, 1033, 943, 918, 850, 831, 808, 773, 737, 714, 696, 654, 600, 550, 539.

4-Methyl-N-(prop-2-yn-1-yl)benzenesulfonamide **(1ab)**

Synthesised according to Amine Sulfonylation Procedure using prop-2-yn-1-amine (350 µL, 5.5 mmol, 1.1 equiv.), p-TsCl (953 mg, 5 mmol, 1 equiv.), and NEt_3 (1.05 mL, 7.5 mmol, 1.5 equiv.), in DCM (30 mL). No further purification was required after workup, yielding the product as a light yellow solid (531 mg, 51%).

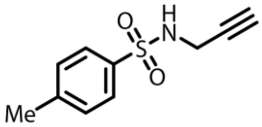

^{1}H NMR (500 MHz, CD_3CN): δ 7.80 – 7.60 (2H, m), 7.38 (2H, d, *J* = 8.1 Hz), 5.86 (1H, d, *J* = 6.4 Hz), 3.72 (2H, dd, *J* = 6.2, 2.5 Hz), 2.42 (3H, s), 2.34 (1H, t, *J* = 2.5 Hz).

^{13}C NMR (126 MHz, CD_3CN): δ 144.9, 138.2, 130.6, 128.1, 79.7, 73.5, 33.2, 21.5.

These data are consistent with those previously reported.[13]

N-(4-fluorobenzyl)-4-methyl-N-(prop-2-yn-1-yl)benzenesulfonamide ***(1ac)***

Synthesised according to the Amine Sulfonylation Procedure using 4-fluoro benzylamine (3.43 mL, 30 mmol, 1.05 equiv.), *p*-TsCl (5.43 g, 28.5 mmol, 1 equiv.), NEt_3 (4.6 mL, 33 mmol, 1.16 equiv.), in DCM (60 mL). The white solid obtained after workup was subjected to the propargylation step (Procedure 1A), using propargyl bromide (3.41 mL, 80% in toluene, 31.8 mmol, 1.12 equiv.), K_2CO_3 (5.52 g, 40 mmol, 1.33 equiv.), in acetone (75 mL). Purified by flash-column chromatography (10 to 30% EtOAc in pentane) to afford a white solid (4.7 g, 52% over two steps).

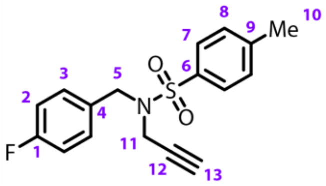

^{1}H NMR (500 MHz, CD_3CN): δ 7.78 (2H, d, *J* = 8.2 Hz, H^7), 7.41 (2H, d, *J* = 8.0 Hz, H^8), 7.35 (2H, dd, *J* = 8.4, 5.6 Hz, H^3), 7.10 (2H, t, *J* = 8.8 Hz, H^2), 4.33 (2H, s, H^5), 3.92 (2H, d, *J* = 2.4 Hz, H^{11}), 2.44 (3H, s, H^{10}), 2.32 (1H, t, *J* = 2.5 Hz, H^{13}).

^{13}C NMR (126 MHz, CD_3CN): δ 163.4 (1C, d, *J* = 243.9 Hz, C^1), 145.2 (1C, s, C^9), 136.9 (1C, s, C^6), 132.7 (d, *J* = 3.1 Hz, C^4), 131.5 (d, *J* = 8.4 Hz, C^3), 130.7 (2C, s, C^8), 128.6 (2C, s, C^7), 116.3 (2C, d, *J* = 21.7 Hz, C^2), 77.3 (1C, s, C^{12}), 75.5 (1C, s, C^{13}), 50.3 (1C, s, C^5), 36.8 (1C, s, C^{11}), 21.6 (1C, s, C^{10}).

^{19}F NMR (376 MHz, $CDCl_3$): δ -113.79 – -113.90 (1F, m, F^1).

HRMS (APCI) calc: $[M+H]^+$ ($C_{16}H_{14}FNO_2S$) 304.0802; measured: 304.0804 = 0.66 ppm difference.

IR (neat) v_{max}**/cm^{-1}:** 3315, 2988, 2919, 1601, 1507, 1425, 1351, 1215, 1164, 1092, 1071.

4-Methyl-N-(4-methylbenzyl)-N-(prop-2-yn-1-yl)benzenesulfonamide ***(1ad)***

4-Methyl-N-(4-methylbenzyl)benzenesulfonamide: Synthesised according to Amine Sulfonylation Procedure using 4-methylbenzylamine (2.55 mL, 20 mmol, 1 equiv.), *p*-TsCl (3.81 g, 20 mmol, 1 equiv.), and NEt_3 (4.18 mL, 30 mmol, 1.5 equiv.), in DCM (30 mL). Purified by flash-column chromatography (5 to 50% EtOAc in pentane) to yield a white solid (3.48 g, 63%).

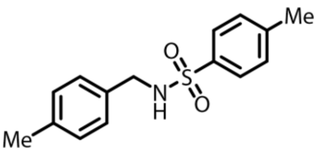

^{1}H NMR (500 MHz, $CDCl_3$): δ 7.76 (2H, d, *J* = 8.3 Hz), 7.37 – 7.29 (2H, m), 7.08 (4H, s), 4.56 (1H, t, *J* = 6.2 Hz), 4.08 (2H, d, *J* = 6.0 Hz), 2.44 (3H, s), 2.31 (3H, s).

^{13}C NMR (126 MHz, $CDCl_3$): δ 143.6, 137.9, 137.0, 133.3, 129.9, 129.5, 128.0, 127.3, 47.2, 21.7, 21.2.

These data are consistent with those previously reported.[14]

4-Methyl-N-(4-methylbenzyl)-N-(prop-2-yn-1-yl)benzenesulfonamide: Synthesised according to Propargylation Procedure 1 using *N*-(4-fluorobenzyl)-4-methylbenzenesulfonamide (3.3 g, 12 mmol, 1 equiv.), propargyl bromide (1.55 mL, 80% in toluene, 14.5 mmol, 1.21 equiv.), K_2CO_3 (3.31 g, 24 mmol, 1.33 equiv.), in acetone (30 mL). Purified by flash-column chromatography (10 to 20% EtOAc in pentane) to yield a white solid (2.61 g, 69%).

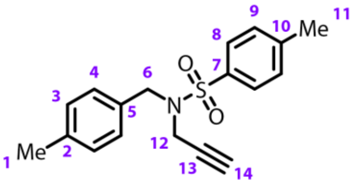

^{1}H NMR (500 MHz, $CDCl_3$): δ 7.79 (2H, d, *J* = 8.3 Hz, H^8), 7.36 – 7.30 (2H, m, H^9), 7.24 (2H, d, *J* = 8.0 Hz, H^4), 7.14 (2H. d, *J* = 7.8 Hz, H^3), 4.31 (2H, s, H^6), 3.94 (2H, d, *J* = 2.5 Hz, H^{12}), 2.45 (3H, s, H^{11}), 2.34 (3H, s, H^1), 2.00 (1H, t, *J* = 2.5 Hz, H^{14}).

^{13}C NMR (126 MHz, $CDCl_3$): δ 143.7 (1C, s, C^{10}), 138.0 (1C, s, C^2), 136.2 (1C, s, C^7), 131.9 (1C, s, C^5),129.6 (2C, s, C^9), 129.5 (2C, s, C^3), 128.9 (2C, s, C^4), 128.0 (2C, s, C^8), 76.5 (1C, s, C^{13}), 74.1 (1C, s, C^{14}),49.6 (1C, s, C^6), 35.5 (1C, s, C^{12}), 21.7 (1C, s, C^{11}), 21.3 (1C, s, C^1).

HRMS (ESI) calc: $[M]^+$ ($C_{18}H_{20}NO_2S$) 314.1209; measured: 314.1213 = 1.3 ppm difference.

IR (neat) v_{max}/cm^{-1}: 3293, 1598, 1517, 1457, 1426, 1328, 1167, 1118, 1091, 1055.

N-(4-fluorophenyl)-4-methyl-N-(prop-2-yn-1-yl)benzenesulfonamide ***(1ae)***

N-(4-fluorophenyl)-4-methylbenzenesulfonamide:_Synthesised according to Amine Sulfonylation Procedure using 4-fluoro aniline (2.84 mL, 30 mmol, 1.05 equiv.), *p*-TsCl (5.43 g, 28.5 mmol, 1 equiv.), NEt_3 (4.6 mL, 33 mmol, 1.16 equiv.), in DCM (60 mL). The product was obtained after workup without any further purification as a white solid (3.1 g, 41%).

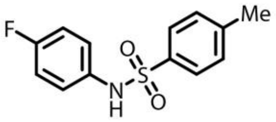

^{1}H NMR (500 MHz, $CDCl_3$): δ 7.62 – 7.56 (2H, m), 7.24 – 7.17 (2H, m), 7.09 – 6.99 (2H, m), 6.97 – 6.86 (2H, m), 6.59 (1H, s), 2.39 (3H, s).

^{13}C NMR (126 MHz, $CDCl_3$): δ 160.9 (d, *J* = 245.6 Hz), 144.2, 135.9, 132.4 (d, *J* = 2.9 Hz), 129.8, 127.4, 125.0 (d, *J* = 8.4 Hz), 116.3 (d, *J* = 22.8 Hz), 21.7.

^{19}F NMR (376 MHz, CD_3CN): δ -115.94 – -116.09 (m).

These data are consistent with those previously reported.[15]

N-(4-fluorophenyl)-4-methyl-N-(prop-2-yn-1-yl)benzenesulfonamide: Synthesised according to propargylation procedure 1A using N-(4-fluorophenyl)-4-methylbenzenesulfonamide (1.42 g, 5 mmol, 1 equiv.), propargyl bromide (670 µL, 80% solution in toluene, 6.26 mmol, 1.25 equiv.), and K_2CO_3 (1.38 g, 10 mmol, 2 equiv.), in acetone (50 mL). The product was obtained after workup without any further purification as a yellow solid (1.22 g, 80% yield).

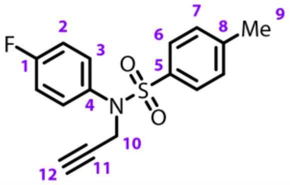

^{1}H NMR (500 MHz, CD_3CN): δ 7.61 – 7.44 (2H, m, *H*^{6}), 7.35 (2H, d, *J* = 7.8 Hz, *H*^{7}), 7.25 – 7.14 (2H, m, *H*^{3}), 7.15 – 7.05 (2H, m, *H*^{2}), 4.41 (2H, d, *J* = 2.6 Hz, *H*^{10}), 2.48 (1H, t, *J* = 2.5 Hz, *H*^{12}), 2.42 (3H, s, *H*^{9}).

^{13}C NMR (126 MHz, CD_3CN): δ 163.1 (d, *J* = 246.0 Hz, *C*^{1}), 145.5 (1C, s, *C*^{8}), 136.3 (d, *J* = 3.1 Hz, *C*^{4}),136.1 (1C, s, *C*^{5}), 131.7 (2C, d, *J* = 9.0 Hz, *C*^{3}), 130.6 (2C, s, *C*^{7}), 128.8 (2C, s, *C*^{6}), 116.7 (2C, d, *J* = 22.9 Hz, *C*^{2}), 79.0 (1C, s, *C*^{11}), 75.2 (1C, s, *C*^{12}), 41.8 (1C, s, *C*^{10}), 21.6 (1C, s, *C*^{9}).

^{19}F NMR (376 MHz, $CDCl_3$): δ -112.47 (1F, tt, *J* = 8.2, 4.9 Hz, *F*^{1}).

HRMS (APCI) calc: $[M+H]^+$ ($C_{16}H_{14}FNO_2SF$) 304.0802; measured: 304.0804 = 0.66 ppm difference.

IR (neat) ν_{max}**/cm^{-1}:** 3289, 1598, 1504, 1452, 1346, 1306, 1291, 1237, 1210, 1186, 1160, 1091, 1015, 920, 867, 846, 810, 727, 707, 688, 652, 588, 547, 523, 441.

N-(4-bromophenyl)-4-methyl-N-(prop-2-yn-1-yl)benzenesulfonamide ***(1af)***

Synthesised according to Amine Sulfonylation Procedure using 4-bromo aniline (3.44 g, 20 mmol, 2 equiv.), *p*-TsCl (1.9 g, 10 mmol, 1 equiv.), pyridine (1.53 mL, 19 mmol, 1.9 equiv.), in DCM (40 mL). The crude product was subjected to the next step without purification, according to propargylation procedure 1A using using propargyl bromide (1.34 mL, 80% solution in toluene, 12.5 mmol, 1.2 equiv.), and K_2CO_3 (2.76 g, 20 mmol, 2 equiv.), in acetone (50 mL). After workup, the crude material was subjected to flash-column chromatography (0 to 20% EtOAc) to obtain the product as a cream solid (1.18 g, 33% yield over two steps).

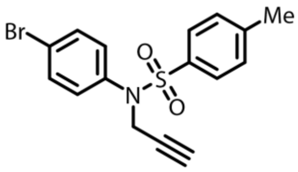

^{1}H NMR (500 MHz, $CDCl_3$): δ 7.56 – 7.51 (2H, m), 7.46 – 7.42 (2H, m), 7.26 – 7.22 (2H, m), 7.13 – 7.09 (2H, m), 4.41 (2H, d, *J* = 2.5 Hz), 2.42 (3H, s), 2.17 (1H, t, *J* = 2.5 Hz).

^{13}C NMR (126 MHz, $CDCl_3$): δ 144.1, 138.5, 135.3, 132.4, 130.1, 129.6, 128.2, 122.3, 77.8, 74.3, 41.0, 21.8.

These data are consistent with those previously reported.[16]

1-Ethynyl-4-nitrobenzene **(1aj)**

Synthesised according to the aryl-acetylene synthesis procedure, using 1-iodo-4-nitrobenzene (2 g, 8 mmol, 1 equiv.), trimethylsilylacetylene (1.34 mL, 9.6 mmol, 1.2 equiv.) $Pd(PPh_3)_2Cl_2$ (464 mg, 0.66 mmol, 0.083 equiv.), CuI (76.6 mg, 0.4 mmol, 0.05 equiv.). After workup, the resulting crude was subjected to the subsequent desilylation step without purification, using K_2CO_3 (2.2 g, 16 mmol, 2 equiv.) as a suspension in methanol (30 mL). Purification by flash-column chromatography (5% EtOAc in pentane) afforded the product (839 mg, 71%).

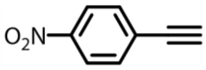

^{1}H NMR (500 MHz, CDCl$_3$): δ 8.24 – 8.17 (2H, m), 7.68 – 7.61 (2H, m), 3.36 (1H, s).

^{13}C NMR (126 MHz, CDCl$_3$): δ 147.7, 133.1, 129.1, 123.7, 82.5, 81.8.

These data are consistent with those previously reported.[17]

1-Ethynyl-4-methoxybenzene **(1ak)**

((4-Methoxyphenyl)ethynyl)trimethylsilane:_Synthesised according to the aryl-acetylene synthesis procedure, using 1-iodo-4-methoxybenzene (3.74 g, 16 mmol, 1 equiv.), trimethylsilylacetylene (2.68 mL, 18.8 mmol, 1.18 equiv.) $Pd(PPh_3)_2Cl_2$ (562 mg, 0.8 mmol, 0.05 equiv.), CuI (152 mg, 0.8 mmol, 0.05 equiv.). Purification by flash-column chromatography (5 to 20% EtOAc in pentane) afforded the product (2.51 g, 77% yield).

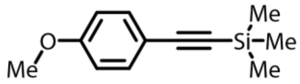

^{1}H NMR (500 MHz, $CDCl_3$): δ 7.69 – 7.24 (2H, m), 7.01 – 6.59 (2H, m), 3.80 (3H, d, *J* = 0.7 Hz), 0.24 (9H, d, *J* = 0.7 Hz).

NMR (126 MHz, $CDCl_3$): δ 159.9, 133.6, 115.4, 113.9, 105.3, 92.6, 55.4, 0.2.

These data are consistent with those previously reported.[18]

1-Ethynyl-4-methoxybenzene**:** ((4-methoxyphenyl)ethynyl)trimethylsilane (2.51 g, 12.3 mmol, 1 equiv.) was subjected to the desilylation step using K_2CO_3 (2.2 g, 16 mmol, 1.3 equiv.) as a suspension in methanol (50 mL). The product was obtained after workup, without further purification (1.54 g, 95%).

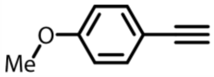

^{1}H NMR (500 MHz, $CDCl_3$): δ 7.60 – 7.36 (2H, m), 6.94 – 6.66 (2H, m), 3.81 (3H, s), 3.00 (1H, s).

^{13}C NMR (126 MHz, $CDCl_3$): δ 159.9, 133.6, 115.4, 113.9, 105.3, 92.6, 55.4, 0.2.

These data are consistent with those previously reported.[19]

1-(4-Ethynylphenyl)ethan-1-one **(1al)**

1-(4-((Trimethylsilyl)ethynyl)phenyl)ethan-1-one: Synthesised according to the aryl-acetylene synthesis procedure, using 1-(4-iodophenyl)ethan-1-one (1.97 g, 8 mmol, 1 equiv.), trimethylsilylacetylene (1.34 mL, 9.6 mmol, 1.2 equiv.) $Pd(PPh_3)_2Cl_2$ (281 mg, 0.4 mmol, 0.05 equiv.), CuI (77 mg, 0.4 mmol, 0.05 equiv.). Purification by flash-column chromatography (10% EtOAc in pentane) afforded the product (1.495 g, 86%)

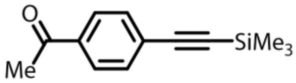

^{1}H NMR (500 MHz, $CDCl_3$): δ 7.95 – 7.80 (2H, m), 7.60 – 7.46 (2H, m), 2.59 (3H, s), 0.26 (9H, s).

^{13}C NMR (126 MHz, $CDCl_3$): δ 197.5, 136.5, 132.2, 128.3, 128.1, 104.1, 98.3, 26.8, -0.03.

These data are consistent with those previously reported.[20]

1-(4-Ethynylphenyl)ethan-1-one: 1-(4-((trimethylsilyl)ethynyl)phenyl)ethan-1-one (1.36 g, 6.3 mmol, 1 equiv.) was subjected to the desilylation step using K_2CO_3 (1.73 g, 12.5 mmol, 2 equiv.) as a suspension in methanol (20 mL). The product was obtained after workup, without further purification (851 mg, 94%).

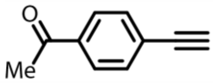

^{1}H NMR (500 MHz, $CDCl_3$): δ 7.97 – 7.86 (2H, m), 7.64 – 7.52 (2H, m), 3.25 (1H, s), 2.60 (3H, s).

^{13}C NMR (126 MHz, $CDCl_3$): δ 197.4, 136.9, 132.5, 128.3, 127.1, 82.9, 80.5, 26.8.

These data are consistent with those previously reported.[21]

4-Ethynylbenzaldehyde **(1am)**

Synthesised according to the aryl-acetylene synthesis procedure, using 4-iodobenzaldehyde (1.86 g, 8 mmol, 1 equiv.), trimethylsilylacetylene (1.34 mL, 9.6 mmol, 1.2 equiv.), $Pd(PPh_3)_2Cl_2$ (281 mg, 0.4 mmol, 0.05 equiv.), and CuI (77 mg, 0.4 mmol, 0.05 equiv.). After workup, the resulting crude was subjected to the subsequent desilylation step without purification, using K_2CO_3 (2.2 g, 16 mmol, 2 equiv.) as a suspension in methanol (20 mL). Purification by flash-column chromatography (7.5% EtOAc in pentane) afforded the product (593 mg, 57% over two steps).

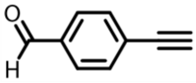

^{1}H NMR (500 MHz, $CDCl_3$): δ 10.02 (1H, s), 7.90 – 7.81 (2H, m), 7.69 – 7.59 (2H, m), 3.29 (1H, s).

^{13}C NMR (126 MHz, $CDCl_3$): δ 191.5, 136.1, 132.9, 129.6, 128.4, 82.8, 81.2.

These data are consistent with those previously reported.[22]

3-Ethynylquinoline **(1ao)**

3-((Trimethylsilyl)ethynyl)quinoline: Synthesised according to the aryl-acetylene synthesis procedure, using 3-bromoquinoline (2.17 mL, 16 mmol, 1 equiv.), trimethylsilylacetylene (2.8 mL, 19.2 mmol, 1.2 equiv.) $Pd(PPh_3)_2Cl_2$ (562 mg, 0.8 mmol, 0.054 equiv.), CuI (152 mg, 0.8 mmol, 0.05 equiv.). Purification by flash-column chromatography (5% EtOAc in pentane) afforded the product (2.04 g, 56% yield).

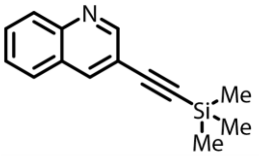

^{1}H NMR (500 MHz, $CDCl_3$): δ 8.91 (1H, d, *J* = 2.1 Hz), 8.25 (1H, dd, *J* = 2.1, 0.9 Hz), 8.08 (1H dq, *J* = 8.5, 1.0 Hz), 7.76 (1H, ddt, *J* = 8.2, 1.3, 0.6 Hz), 7.71 (1H, ddd, *J* = 8.4, 6.9, 1.5 Hz), 7.55 (1H, ddd, *J* = 8.2, 6.9, 1.2 Hz), 0.30 (9H, s).

^{13}C NMR (126 MHz, $CDCl_3$): δ 152.5, 147.0, 139.1, 130.3, 129.5, 127.7, 127.4, 127.2, 117.4, 102.2, 98.4, 0.0.

These data are consistent with those previously reported.[23]

3-Ethynylquinoline: 3-((trimethylsilyl)ethynyl)quinoline (2.04 g, 9.05 mmol, 1 equiv.) was subjected to the desilylation step using K_2CO_3 (4.42 g, 32 mmol, 3.56 equiv.) as a suspension in methanol (30 mL). The product was obtained after workup, without further purification (1.326 g, 96%).

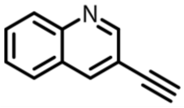

^{1}H NMR (500 MHz, $CDCl_3$): δ 8.95 (1H, d, *J* = 2.1 Hz), 8.29 (1H, dd, *J* = 2.2, 0.8 Hz), 8.10 (1H, dq, *J* = 8.6, 0.9 Hz), 7.81 – 7.77 (1H, m), 7.74 (1H, ddd, *J* = 8.4, 6.9, 1.4 Hz), 7.58 (1H, ddd, *J* = 8.1, 6.9, 1.2 Hz), 3.28 (1H, s).

^{13}C NMR (126 MHz, $CDCl_3$): δ 152.4, 147.3, 139.5, 130.5, 129.6, 127.8, 127.5, 127.2, 116.4, 81.1, 80.6.

These data are consistent with those previously reported.[24]

2-Iodo-1,3,5-triisopropylbenzene **(2f)**

Synthesised according to a modified literature procedure.[25] To a solution of 2-iodo-1,3,5-triisopropylbenzene (12.2 g, 60 mmol, 1 equiv.) in HFIP (120 mL) was added NIS (14.85 g, 66 mmol, 1.1 equiv.). The reaction was stirred for 3 hours before being evaporated under reduce pressure. A saturated aqueous solution of sodium thiosulfate (100 mL) was added, and extracted with DCM (3 x 50 mL). The combined organic extracts were filtered and evaporated under reduced pressure. The crude product was subjected to flash-column chromatography (pentane) to afford the aryl-iodide as a colourless oil (17.4 g, 88%).

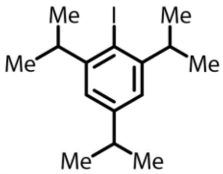

^{1}H NMR (500 MHz, $CDCl_3$): δ 6.96 (2H, s), 3.40 (2H, hept, *J* = 6.8 Hz), 2.88 (1H, hept, *J* = 6.9 Hz), 1.25 (18H, dd, *J* = 6.9, 5.3 Hz).

^{13}C NMR (126 MHz, $CDCl_3$): δ 150.9, 149.0, 122.2, 105.9, 39.4, 34.0, 24.1, 23.6.

These data are consistent with those previously reported.[26]

Syntheses of Unsuccessful Alkynes

<u>1-(Prop-2-yn-1-yl)-1H-indole</u>

To a stirring solution of indole (2.34 g, 20 mmol, 1 equiv.) in THF (50 mL), at 0◦C, was added sodium hydride (1.6 g, 60% in mineral oil, 40 mmol, 2 equiv.). Propargyl bromide (3.78 mL, 35.3 mmol, 80% in toluene, 1.77 equiv.) was added drop-wise and the stirring suspension was allowed to warm to room temperature. After stirring for 18 hours, saturated aqueous ammonium chloride as added slowly (20 mL) and the mixture was extracted with DCM (3 x 20 mL). The combined organic extracts were filtered and evaporated under reduced pressure. The crude product was subjected to flash-column chromatography (2.5% EtOAc in pentane) to accord the product as a brown oil that slowly solidified (1.6 g, 52 %).

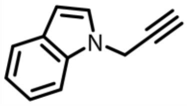

^{1}H NMR (500 MHz, $CDCl_3$): δ 7.64 (1H, dt, *J* = 7.9, 1.0 Hz), 7.41 (1H, dt, *J* = 8.2, 0.9 Hz), 7.29 – 7.23 (1H, m), 7.21 (1H, d, *J* = 3.2 Hz), 7.14 (1H, ddd, *J* = 8.0, 7.0, 1.0 Hz), 6.54 (1H, dd, *J* = 3.2, 0.9 Hz), 4.87 (2H, d, *J* = 2.6 Hz), 2.39 (1H, t, *J* = 2.6 Hz).

^{13}C NMR (126 MHz, $CDCl_3$): δ 135.9, 129.0, 127.4, 122.0, 121.3, 120.0, 109.4, 102.2, 77.9, 73.6, 35.9.

These data are consistent with those previously reported.[27]

Phenyl(prop-2-yn-1-yl)sulfane

Synthesised according to propargylation procedure 1B using benzenethiol (2.04 mL, 20 mmol, 1 equiv.), propargyl bromide (4.46 mL, 80% solution in toluene, 41.7 mmol, 2.1 equiv.), and K_2CO_3 (4.15 g, 30 mmol, 1.5 equiv.), in acetone (50 mL). Purified by flash-column chromatography (pentane) to obtain the product as an orange oil (1.48 g, 50% yield).

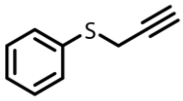

^{1}H NMR (500 MHz, $CDCl_3$): δ 7.50 – 7.41 (2H, m), 7.38 – 7.29 (2H, m), 7.28 – 7.20 (1H, m), 3.61 (2H, d, *J* = 2.6 Hz), 2.35 – 2.13 (1H, m).

^{13}C NMR (126 MHz, $CDCl_3$): δ 135.1, 130.2, 129.1, 127.1, 80.0, 71.7, 22.7.

These data are consistent with those previously reported.[28]

1-Phenyl-5-(prop-2-yn-1-ylthio)-1H-tetrazole

Synthesised according to the propargylation procedure, using 1-phenyl-1H-tetrazole-5-thiol (1.78 g, 10 mmol, 1 equiv.), propargyl bromide (1.89 mL, 80% wt in toluene, 17.7 mmol, 1.77 equiv.), and K_2CO_3 (2.76 g, 20 mmol, equiv.), in acetone (50 mL). The product was obtained after workup, without further purification as a light cream solid (1.01 g, 47%).

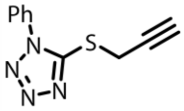

^{1}H NMR (500 MHz, $CDCl_3$): δ 7.65 – 7.51 (5H, m), 4.18 (2H, d, *J* = 2.7 Hz), 2.32 (1H, t, *J* = 2.7 Hz).

^{13}C NMR (126 MHz, $CDCl_3$): δ 153.0, 133.6, 130.5, 130.1, 124.0, 77.3, 73.3, 22.0.

These data are consistent with those previously reported.[29]

1-(Prop-2-yn-1-yloxy)naphthalene

Synthesised according to propargylation procedure 1B using naphthalen-1-ol (1.44 g, 10 mmol, 1 equiv.), propargyl bromide (2.23 mL, 80% solution in toluene, 20.84 mmol, 2.08 equiv.), and K_2CO_3 (2.76 g, 20 mmol, 1.5 equiv.), in acetone (30 mL). Purified by flash-column chromatography (2% EtOAc in pentane) to obtain the product as a colourless oil (1.26 g, 69% yield).

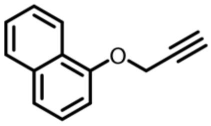

^{1}H NMR (500 MHz, $CDCl_3$): δ 8.32 – 8.25 (1H, m), 7.85 – 7.76 (1H, m), 7.52 – 7.45 (3H, m), 7.40 (1H, dd, *J* = 8.3, 7.6 Hz), 6.96 (1H, dd, *J* = 7.6, 0.9 Hz), 4.91 (2H, d, *J* = 2.4 Hz), 2.56 (1H, t, *J* = 2.4 Hz).

^{13}C NMR (126 MHz, $CDCl_3$): δ 153.5, 134.7, 127.6, 126.7, 125.8, 125.7, 125.5, 122.2, 121.3, 105.6, 78.8, 75.7, 56.3.

These data are consistent with those previously reported.[4]

2-((Prop-2-yn-1-yloxy)methyl)furan

To a round-bottom flask equipped with a stirrer bar, at 0°C, was added sodium hydride (3 g, 60% in mineral oil, 75 mmol, 2.5 equiv.) and THF (75 mL). Furan-2-ylmethanol (2.6 mL, 30 mmol, 1 equiv.) was added, and the suspension was left to stir for 30 minutes. Propargyl bromide (5.7 mL, 80% wt in toluene, 83.22 mmol, 2.77 equiv.) was added, and the mixture was warmed to room temperature. After completion of the reaction (TLC monitoring), the suspension was cooled to 0°C, at which point water (40 mL) was added slowly. The aqueous and organic layers were separated, and the aqueous layer was extracted with DCM (3 x). The combined organic extracts were washed with brine, before being dried over $MgSO_4$, filtered, and concentrated under reduced pressure.The resulting crude mixture was subjected to flash-column chromatography (0 to 20% EtOAc) to obtain the product as a yellow oil (2.89 g, 71%).

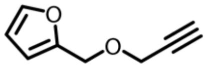

^{1}H NMR (500 MHz, $CDCl_3$): δ 7.42 (1H, dd, *J* = 2.0, 1.0 Hz), 6.53 – 6.26 (2H, m), 4.57 (2H, s), 4.16 (2H, d, *J* = 2.4 Hz), 2.46 (1H, t, *J* = 2.4 Hz).

^{13}C NMR (126 MHz, $CDCl_3$): δ 150.9, 143.2, 110.5, 110.2, 79.4, 74.9, 63.2, 56.9.

These data are consistent with those previously reported.[30]

2-((Prop-2-yn-1-yloxy)methyl)tetrahydro-2H-pyran

Synthesised according to a modified literature procedure.[31] To a stirring solution of propargyl alcohol (2.6 mL, 44.6 mmol, 1 equiv.) and *p*-TsOH.H_2O (90 mg, 0.47 mmol, 0.01 equiv.) in DCM (50 mL), in a round-bottom flask cooled to 0°C, was added DHP (4.3 mL, 46.8 mmol) dropwise. The reaction mixture was warmed to room temperature and was stirred for 1 hour. Saturated aqueous $NaHCO_3$ (30 mL) was added, and the aqueous layer was extracted with DCM (45 mL). The combined organic layer dried over $MgSO_4$, filtered, and evaporated under reduced pressure to give the compound as a colourless oil (5.45 g, 79%).

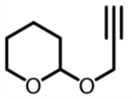

^{1}H NMR (500 MHz, $CDCl_3$): δ 4.81 (1H, t, *J* = 3.4 Hz), 4.44 – 4.14 (2H, m), 3.83 (1H, ddd, *J* = 11.5, 9.0, 3.2 Hz), 3.53 (1H, dtd, *J* = 11.3, 4.4, 1.9 Hz), 2.41 (2H, t, *J* = 2.4 Hz), 1.91 – 1.67 (2H, m), 1.67 – 1.35 (3H, m).

^{13}C NMR (126 MHz, $CDCl_3$): δ 97.0, 79.9, 74.1, 62.1, 54.1, 30.3, 25.4, 19.1.

These data are consistent with those previously reported.[31]

Tert-butyl prop-2-yn-1-yl carbonate

Synthesised according to a modified literature procedure.[32] To a solution of propargyl alcohol (700 μL, 12 mmol, 1 equiv.), DMAP (148 mg, 1.2 mmol, 0.1 equiv.) and NEt_3 (1.7 mL, 12 mmol, 1 equiv.), in DCM (20 mL), was added di-tert-butyl pyrocarbonate (2.8 mL, 12 mmol, 1 equiv.). After stirring for 24 hours at room temperature, the mixture was poured into EtOAc (30 mL), washed with 1 M HCl (20 mL), a saturated aqueous solution of sodium bicarbonate (20 mL), and brine (10 mL). The organic phase was then dried over $MgSO_4$, filtered, and evaporated under reduced pressure to afford the product as a yellow oil (1.7 g, 91%).

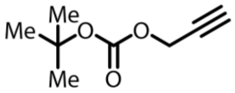

^{1}H NMR (500 MHz, $CDCl_3$): δ 4.66 (2H, d, *J* = 2.5 Hz), 2.49 (1H, t, *J* = 2.5 Hz), 1.49 (9H, s).

^{13}C NMR (126 MHz, $CDCl_3$): δ 152.9, 83.2, 77.5, 75.4, 54.5, 27.9, 14.4.

These data are consistent with those previously reported.[32]

General Synthetic Procedure and Characterisations of Z-Fluorovinyl Iodonium Salts

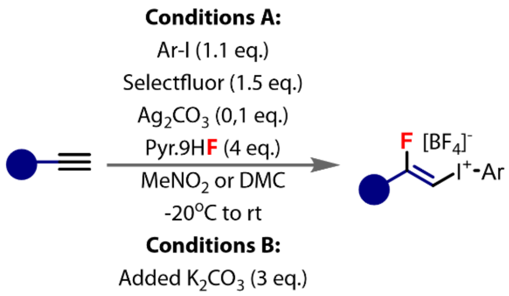

Conditions A

To an HDPE vial equipped with a stirrer bar, was added aryl iodide (1.1 equiv.), Selectfluor (1.5 equiv.), and Ag_2CO_3 (0.1 equiv.). The vial was wrapped in aluminium foil before being placed in a -20 °C cooling bath. Solvent ($MeNO_2$ or dimethyl carbonate) was added in the amount specified to give a 0.125 M concentration of alkyne. Pyridine.9HF (4 equiv.) was added drop-wise, and the reaction was left to stir for 5 minutes. Alkyne (1 equiv.) was added slowly, and the reaction mixture was allowed to warm to room temperature, at which it was stirred for 24 hours. Distilled water (half the reaction volume) was added to the reaction mixture, which was then transferred into a separating funnel, along with a further rinse of the vial with dimethyl carbonate. The aqueous and organic layers were separated, and the aqueous phase was extracted with dimethyl carbonate (2 x). The aqueous phase was slowly poured into a saturated aqueous solution of $NaHCO_3$. The combined organic extracts were washed with a saturated aqueous solution of NaBF4, and twice with distilled water, then evaporated under reduced pressure. To the resulting crude product was added either Et_2O or pentane (10-15 mL per mmol of alkyne added at the start of the reaction). The suspension was sonicated for 10-15 minutes, then carefully decanted (leaving behind the (Z)-FVI). This trituration process was typically repeated 1-3 times. Evaporation of the residual solvent yielded the Z-fluorovinyl iodonium Salt.

Conditions B

To an HDPE vial equipped with a stirrer bar, was added aryl iodide (1.1 equiv.), Selectfluor (1.5 equiv.), K_2CO_3 (3 equiv.), and Ag_2CO_3 (0.1 equiv.). The vial was wrapped in aluminium foil before being placed in a -20 ◦C cooling bath. Nitromethane was added in the amount specified to give a 0.125 M concentration of alkyne. Pyridine.9HF (4 equiv.) was added drop-wise, and the reaction was left to stir for 5-10 minutes. Alkyne (1 equiv.) was added slowly, and the reaction mixture was allowed to warm to room temperature. The procedure from this point onwards is identical to that under "Conditions A".

(Z)-(2-fluoro-5-phenylpent-1-en-1-yl)(mesityl)iodonium BF_4 ***(3a)***

Synthesised according to conditions B, using 5-phenyl-1-pentyne (190 μL, 1.25 mmol, 1 equiv.), Ag_2CO_3 (35.4 mg, 0.125 mmol, 0.1 equiv.), 2-iodomesitylene (338 mg, 1.375 mmol, 1.1 equiv.), Selectfluor (664 mg, 1.875 mmol, 1.5 equiv.), K_2CO_3 (518 mg, 3.75 mmol, 3 equiv.), and pyridine.9HF (1.178 mL), in $MeNO_2$ (8.82 mL). Trituration with Et_2O (3 x 13 mL) yielded a cream solid (552 mg, 80% yield based on a purity of 90% determined by quantitative ^{19}F NMR assay. The compound was spectroscopically pure by ^{1}H, ^{13}C, and ^{19}F NMR.

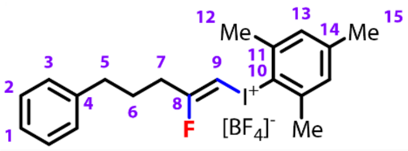

^{1}H NMR (500 MHz, CD_3CN): δ 7.28 (2H, t, *J* = 7.4 Hz, *H*^{2}), 7.21 (2H, s, *H*^{13}), 7.19 (1H, t, *J* = 7.3 Hz, *H*^{1}), 7.13 (2H, d, *J* = 7.0 Hz, *H*^{3}), 6.29 (1H, d, *J* = 34.8 Hz, *H*^{9}), 2.71 – 2.50 (10H, m, *H*$^{5+7+12}$), 2.34 (3H, s, *H*^{15}), 1.83 (2H, p, *J* = 7.5 Hz, *H*^{6}).

^{13}C NMR (126 MHz, CD_3CN): δ 174.1 (1C, d, *J* = 276.9 Hz,*C*^{8}), 145.8 (1C, s, *C*^{14}), 143.4 (2C, s, *C*^{11}) , 142.1 (1C, s, *C*^{4}), 131.2 (2C, s, *C*^{13}), 129.4 (2C, s, *C*^{2}), 129.3 (2C, s, *C*^{3}), 127.1 (1C, s, *C*^{1}), 120.4 (1C, s, *C*^{10}), 74.5 (d, *J* = 22.1 Hz, *C*^{9}), 35.0 (1C, s, *C*^{5}), 32.1 (d, *J* = 24.1 Hz, *C*^{7}), 27.9 (1C, s, *C*^{6}), 27.2 (2C, s, *C*^{12}), 21.0 (1C, s, *C*^{15}).

^{19}F NMR (376 MHz, CD_3CN): δ -66.07 (1F, dt, *J* = 33.8, 18.5 Hz, *F*^{8}), -151.48 + -151.53 (*BF_4*).

HRMS (ESI) calc: $[M – BF_4]^+$ ($C_{20}H_{23}FI$) 409.0823; measured: 409.0847 = 5.8 ppm difference.

IR (neat) v_{max}**/cm^{-1}:** 3097, 3026, 2922, 2852, 1651, 1454, 1300, 1050, 1027, 755, 701.

(Z)-(2-fluoro-2-phenylvinyl)(mesityl)iodonium BF_4 ***(3b)***

Synthesised according to conditions B using phenylacetylene (275 μL, 2.5 mmol, 1 equiv.), Ag_2CO_3 (69 mg, 0.25 mmol, 0.1 equiv.), 2-iodomesitylene (676.5 mg, 2.75 mmol, 1.1 equiv.), Selectfluor (1.33 g, 3.75 mmol, 1.5 equiv.), and pyridine.9HF (2.36 mL), in $MeNO_2$ (17.6 mL). Trituration with Et_2O (3 x 15 mL) and evaporation of the residual solvent under reduced pressure yielded a yellow solid (935 mg, 71% yield based on a purity of 86% determined by quantitative ^{19}F NMR assay). The compound was spectroscopically pure by ^{1}H, ^{13}C, and ^{19}F NMR. This reaction was repeated on several occasions during our studies, and sometimes yielded the *E* isomer as a very minor side product (1: ≤0.01 *Z:E*).

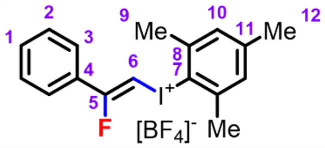

^{1}H NMR (500 MHz, CD_3CN): δ 7.68 (2H, d, *J* = 7.4 Hz, H^3), 7.59 (1H, t, *J* = 7.4 Hz, H^1), 7.52 (2H, *t*, *J* = 7.9 Hz, H^2), 7.23 (2H, s, H^{10}), 7.05 (1H, d, *J* = 35.4 Hz, H^6), 2.67 (6H, s, H^9), 2.35 (3H, s, H^{12}).

^{13}C NMR (126 MHz, CD_3CN): δ 168.2 (1C, d, *J* = 265.5 Hz, C^5), 145.9 (1C, s, C^{11}), 143.6 (2C, s C^8),133.9 (1C, s, C^1), 131.2 (2C, s, C^{10}), 130.2 (2C, d, *J* = 1.9 Hz, C^2), 128.3 (1C, d, *J* = 27.2 Hz, C^4), 127.3 (2C, d, *J* = 7.1 Hz, C^3), 120.5 (1C, s, C^7), 74.6 (1C, d, *J* = 23.9 Hz, C^6), 27.3 (2C, s, C^9), 21.0 (1C, s, C^{12}).

^{19}F NMR (376 MHz, CD_3CN): δ -79.76 (1F, d, *J* = 35.3 Hz, F^5), -151.54 + -151.59 (BF_4).

HRMS (ESI) calc: $[M – BF_4]^+$ ($C_{17}H_{17}FI$) 367.0354; measured: 367.0361 = 2.0 ppm difference.

IR (neat) v_{max}/cm^{-1}: 3099, 2981, 2902, 1620, 1575, 1447, 1282, 1028, 1020, 776, 743, 688, 634, 518.

Melting point: 182 – 184 °C (decomposition).

(Z)-(2-fluorododec-1-en-1-yl)(mesityl)iodonium BF_4 ***(3c)***

Synthesised according to conditions B, but at a higher concentration, using dodec-1-yne (534 μL, 2.5 mmol, 1 equiv.), Ag_2CO_3 (69 mg, 0.25 mmol, 0.1 equiv.), 2-iodomesitylene (676.5 mg, 2.75 mmol, 1.1 equiv.), Selectfluor (1.33 g, 3.75 mmol, 1.5 equiv.), K_2CO_3 (1.04 g, 3.75 mmol, 3 equiv.), and pyridine.9HF (2.36 mL), in $MeNO_2$ (17.64 mL). Trituration with pentane (2 x 25 mL) yielded an off-white solid (1.07 g, 72% yield based on a purity of 87% determined by quantitative ^{19}F NMR assay). The compound was spectroscopically pure by ^{1}H, ^{13}C, and ^{19}F NMR.

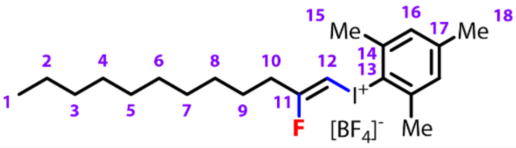

^{1}H NMR (500 MHz, $CDCl_3$): δ 7.08 (2H, s, H^{16}), 6.20 (1H, d, J = 33.8 Hz, H^{13}), 2.66 (6H, *s*, H^{15}), 2.52 (2H, dt, J = 17.4, 7.6 Hz, H^{10}), 2.35 (3H, s, H^{18}), 1.53 (2H, t, J = 7.3 Hz, H^{9}), 1.28 (2H, q, J = 7.2 Hz, H^{8}), 1.24 (12H, m, H^{2-7}), 0.88 (3H, t, J = 6.9 Hz, H^{1}).

^{13}C NMR (126 MHz, $CDCl_3$): δ 173.4 (1C, d, J = 278.1 Hz, C^{11}), 144.3, 142.5, 130.3 (2C, s, C^{16}), 119.6, 73.1 (1C, d, J = 23.0 Hz, C^{13}), 32.5 (1C, d, J = 23.8 Hz, C^{11}), 32.0, 29.6, 29.5, 29.4, 29.2, 28.8 (C^{2-8}), 27.2 (d, J = 1.3 Hz), 25.6, 22.8, 21.2, 14.3 (1C, s, C^{1}).

^{19}F NMR (377 MHz, $CDCl_3$): δ -65.12 (1F, dt, J = 35.2, 17.9 Hz, F^{11}), -147.55 – -147.73 (BF_4) .

HRMS (Nanospray) calc: $[M – BF_4]^+$ ($C_{21}H_{33}FI$) 431.1611; measured: 431.1620 = 2.1 ppm difference.

IR (neat) v_{max}/cm^{-1}: 1648, 1468, 1304, 878, 849, 758, 522.

Melting point: 73 - 76 °C

(Z)-(2-cyclohexyl-2-fluorovinyl)(mesityl)iodonium BF_4 ***(3d)***

Synthesised according to conditions B, using ethynylcyclohexane (653 µL, 5 mmol, 1 equiv.), Ag_2CO_3 (136 mg, 0.5 mmol, 0.1 equiv.), 2-iodomesitylene (1.35 g, 5.5 mmol, 1.1 equiv.), Selectfluor (2.66 g, 7.25 mmol, 1.5 equiv.), and pyridine.9HF (4.71 mL), in dimethylcarbonate (35.3 mL). Trituration with Et_2O (2 x 50 mL) yielded a white solid (1.56 g, 64% yield based on a purity of 94% determined by quantitative ^{19}F NMR assay). The compound was spectroscopically pure by ^{1}H, ^{13}C, and ^{19}F NMR.

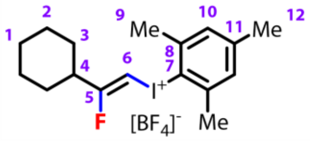

^{1}H NMR (500 MHz, CD_3CN): δ 7.21 (2H, s, H^{10}), 6.25 (1H, d, J = 35.5 Hz, H^{6}), 2.59 (7H, m, H^{9+4}), 2.57 – 2.59 (1H, m, H^{4}), 2.35 (3H, s, H^{12}), 1.89 – 1.70 + 1.66 (5H, m, H^{1-3}), 1.38 – 1.11 (5H, m, H^{1-3}).

^{13}C NMR (126 MHz, CD_3CN): δ 177.4 (1C, d, J = 278.2 Hz, C^{5}), 145.7 (1C, s, C^{11}), 143.5 (2C, s, C^{8}), 131.2 (2C, s, C^{10}), 120.0 (1C, s, C^{7}), 74.0 (1C, d, J = 22.8 Hz, C^{6}), 42.1 (1C, d, J = 22.2 Hz, C^{4}), 28.5 (2C, d, J = 2.0 Hz, C^{3}), 27.1 (2C, s, C^{9}), 26.1 *(CH2)*, 25.9 *(CH2)*, 21.0 (1C, s, C^{12}).

^{19}F NMR (377 MHz, $CDCl_3$): δ -70.46 (1F, dd, J = 35.7, 15.1 Hz, F^{5}), -151.572 - 151.62 (*BF_4*).

HRMS (ESI) calc: $[M – BF_4]^+$ ($C_{17}H_{23}FI$) 373.0823; measured: 373.0833 = 2.6 ppm difference.

IR (neat) v_{max}/cm^{-1}: 3103, 2931, 2858, 1640, 1452, 1303, 1249, 1012.

Melting point: 137 – 142 °C

(Z)-(2-fluoro-3,3-dimethylbut-1-en-1-yl)(mesityl)iodonium BF_4 ***(3e)***

Synthesised according to conditions A, using 3,3-dimethylbut-1-yne (154 µL, 1.25 mmol, 1 equiv.), Ag_2CO_3 (35.4 mg, 0.125 mmol, 0.1 equiv.), 2-iodomesitylene (338 mg, 1.375 mmol, 1.1 equiv.), Selectfluor (664.5 mg, 1.875 mmol, 1.5 equiv.), K_2CO_3 (518 mg, 3.75 mmol, 3 equiv.), and pyridine.9HF (1.18 mL), in $MeNO_2$ (8.82 mL). Trituration with Et_2O (1 x 50 mL) yielded a white solid (424 mg, 78%) The compound was spectroscopically pure by ^{1}H, ^{13}C, and ^{19}F NMR, with a purity of >97% determined by quantitative ^{19}F NMR assay.

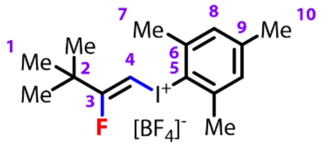

^{1}H NMR (500 MHz, CD_3CN): δ 7.22 (2H, s, H^8), 6.35 (1H, d, J = 35.7 Hz, H^4), 2.59 (6H, s, H^7), 2.35 (3H, s, H^{10}), 1.18 (9H, s, H^1).

^{13}C NMR (126 MHz, CD_3CN): δ 180.0 (1C, d, J = 279.4 Hz, C^3), 145.7 (1C, s, C^9), 143.6 (2C, s, C^6), 131.2 (2C, s, C^8), 119.6 (1C, s, C^5), 74.2 (1C, d, J = 23.4 Hz, C^4), 38.3 (1C, d, J = 21.7 Hz, C^2), 27.14 (2C, s, C^7), 26.82 (3C, d, J = 2.4 Hz, C^1), 21.0 (1C, s, C^{10}).

^{19}F NMR (376 MHz, CD_3CN): δ -73.37 (1F, d, J = 35.7 Hz, F^3), -151.49 (BF_4).

HRMS (ESI) calc: $[M – BF_4]^+$ ($C_{15}H_{21}FI$) 347.0667; measured: 347.0676 = 2.6 ppm difference.

IR (neat) v_{max}/cm^{-1}: 3119, 2982, 1630, 1459, 1374, 1281, 1033, 1002, 867, 767, 520.

Melting point: 126 - 127 °C

(Z)-(4-bromo-2-fluorobut-1-en-1-yl)(mesityl)iodonium BF_4 ***(3f)***

Synthesised according to conditions B, using 4-bromo-1-butyne (235 µL, 2.5 mmol, 1 equiv.), Ag_2CO_3 (69 mg, 0.25 mmol, 0.1 equiv.), 2-iodomesitylene (677 mg, 2.75 mmol, 1.1 equiv.), Selectfluor (1.33 g, 3.75 mmol, 1.5 equiv.), K_2CO_3 (1.04 g, 3.75 mmol, 3 equiv.), and pyridine.9HF (2.36 mL), in either $MeNO_2$ or dimethyl carbonate (17.6 mL). Trituration with Et_2O (3 x 15 mL) and evaporation of the residual solvent under reduced pressure yielded a yellow solid (874 mg, 69% % yield based on a purity of 95% determined by quantitative ^{19}F NMR assay. The compound was spectroscopically pure by ^{1}H, ^{13}C, and ^{19}F NMR.

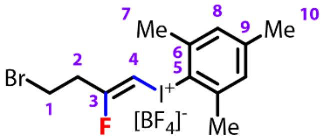

^{1}H NMR (500 MHz, CD_3CN): δ 7.22 (2H, s, H^8), 6.45 (1H, d, J = 34.7 Hz, H^4), 3.56 (2H, t, J = 6.3 Hz, H^1), 3.13 (2H, dt, J = 17.4, 6.3 Hz, H^2), 2.62 (6H, s, H^7), 2.35 (3H, s, H^{10}).

^{13}C NMR (126 MHz, CD_3CN): δ 170.5 (1C, d, J = 276.2 Hz, C^3), 145.8 (1C, s, C^9), 143.5 (2C, s, C^6), 131.2 (2C, s, C^8), 120.3 (1C, s, C^5) , 76.5 (1C, d, J = 21.1 Hz, C^4), 35.7 (1C, d, J = 24.7 Hz, C^2), 27.7 (1C, d, J = 1.5 Hz, C^1), 27.2 (2C, s, C^7), 21.0 (1C, s, C^{10}).

^{19}F NMR (376 MHz, CD_3CN): δ -71.82 (1F, dt, J = 34.8, 17.5 Hz, F^3), -151.38 (BF_4).

HRMS (ESI) calc: $[M – BF_4]^+$ ($C_{13}H_{16}BrFI$) 396.9459; measured: 396.9472 = 3.5 ppm difference.

IR (neat) v_{max}/cm^{-1}: 3110.8, 2980.4, 1650.4, 1443.9, 1390.8, 1285.4, 1252.5, 1057.9, 1014.4, 942.8, 883.6, 849.6, 759.8, 678.8, 568.4, 536.5, 520.5, 503.1.

Melting point: 129 – 133 °C.

(Z)-(4-(benzyloxy)-2-fluorobut-1-en-1-yl)(mesityl)iodonium BF_4 **(3g)**

Synthesised according to conditions A, using ((prop-2-yn-1-yloxy)methyl)benzene (366 mg, 2.5 mmol, 1 equiv.), Ag_2CO_3 (69 mg, 0.25 mmol, 0.1 equiv.), 2-iodomesitylene (677 mg, 2.75 mmol, 1.1 equiv.), Selectfluor (1.33 g, 3.75 mmol, 1.5 equiv.), and pyridine.9HF (2.36 mL), in dimethyl carbonate (17.6 mL). Trituration with Et_2O (2 x 25 mL) and evaporation of the residual solvent under reduced pressure yielded a white solid (747 mg, 48% yield based on a purity of 82% determined by quantitative ^{19}F NMR assay). The compound was spectroscopically pure by ^{1}H, ^{13}C, and ^{19}F NMR.

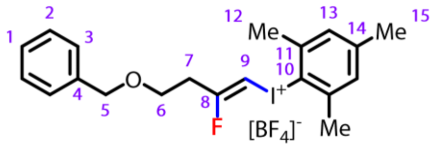

^{1}H NMR (500 MHz, CD_3CN): δ 7.35 – 7.27 (3H, m, H^{1+2}), 7.23 – 7.19 (2H, m, H^{3}), 7.18 – 7.15 (2H, m, $H^{\mathbf{13}}$), 6.34 (1H, dt, J = 34.8, 0.9 Hz, H^{9}), 4.42 (2H, s, H^{5}), 3.64 (2H, t, J = 5.8 Hz, H^{6}), 2.86 (2H, dtd, J =16.8, 5.8, 0.9 Hz, H^{7}), 2.57 (6H, s, H^{12}), 2.34 (3H, s, H^{15}).

^{13}C NMR (126 MHz, CD_3CN): δ 172.2 (1C, d, J = 276.8 Hz, C^{8}), 145.7 (1C, s, C^{14}), 143.4 (2C, s, C^{11}), 139.1 (1C, s, C^{4}), 131.2 (1C, s, C^{8}), 129.3 (1C, s, C^{1}), 128.63 (2C, s, C^{2}), 128.61 (2C, s, C^{3}), 120.2 (1C, s, C^{10}), 75.1 (1C, d, J = 21.5 Hz, C^{9}), 73.4 (1C, s, C^{5}), 65.8 (1C, d, J = 2.4 Hz, C^{6}), 33.5 (1C, d, J = 24.3 Hz, C^{7}), 27.1 (2C, d, J = 1.2 Hz, C^{12}), 21.0 (1C, s, C^{15}).

^{19}F NMR (376 MHz, CD_3CN): -66.84 (1F, dt, J = 34.1, 16.7 Hz, F^{8}), -150.65 + -150.71 (BF_4).

HRMS (ESI) calc: $[M-BF_4]^+$ ($C_{20}H_{23}OFI$) 425.0772; measured: 425.0775 = 1.41 ppm difference.

IR (neat) v_{max}**/cm^{-1}:** 3099, 1648, 1453, 1388, 1263, 1199, 1089, 1055, 1012, 985, 958, 865, 836, 755, 740, 703, 522.

Melting point: 128 – 133 °C (decomposition).

(Z)-(2-fluoro-4-(tosyloxy)but-1-en-1-yl)(mesityl)iodonium BF_4 ***(3h)***

Synthesised according to conditions B, using but-3-yn-1-yl 4-methylbenzenesulfonate (561 mg,1.25 mmol, 1 equiv.), Ag_2CO_3 (34.8 mg, 0.125 mmol, 0.1 equiv.), 2-iodomesitylene (338 mg, 1.375 mmol, 1.1 equiv.), Selectfluor (665 mg, 1.875 mmol, 1.5 equiv.), K_2CO_3 (518 mg, 3.75 mmol, 3 equiv.), and pyridine.9HF (1.18 mL), in $MeNO_2$ (8.8 mL). Trituration with Et_2O (3 x 15 mL) and evaporation of the residual solvent under reduced pressure yielded a light-yellow oil (506 mg, 55% yield based on a purity of 79% determined by quantitative ^{19}F NMR assay). The compound was spectroscopically pure by ^{1}H, ^{13}C, and ^{19}F NMR.

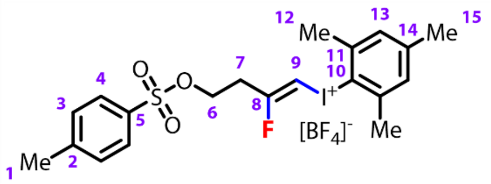

^{1}H NMR (500 MHz, CD_3CN): δ 7.72 – 7.68 (2H, m, H^4), 7.45 – 7.42 (2H, m, H^3), 7.24 – 7.19 (2H, m, H^{13}), 6.35 (1H, dt, J = 34.6, 0.8 Hz, H^9), 4.15 (2H, t, J = 5.7 Hz, H^6), 2.91 (2H, dt, J = 17.2, 5.8 Hz, H^7), 2.58 (6H, s, H^{12}), 2.45 (3H, s, H^1), 2.36 (3H, s, H^{15}).

^{13}C NMR (126 MHz, CD_3CN): δ 169.5 (1C, d, J = 276.2 Hz, C^8), 146.8 (1C, s, C^2), 145.8 (1C, s, C^{14}), 143.5 (2C, s, C^{11}), 133.2 (1C, s, C^2), 131.22 (2C, s, C^{13}), 131.15 (2C, s, C^3), 128.8 (2C, s, C^4), 120.3 (1C, s, C^{10}), 76.6 (1C, d, J = 21.0 Hz, C^9), 66.2 (1C, d, J = 1.5 Hz, C^6), 32.7 (1C, d, J = 25.1 Hz, C^7), 27.2 (2C, d, J = 1.1 Hz, C^{12}), 21.7 (1C, s, C^1), 21.0 (1C, s, C^{15}).

^{19}F NMR (376 MHz, $C(CD_3)_2O$): δ -69.95 (1F, dt, J = 34.6, 17.0 Hz, F^8), -146.50 – -152.30 (BF_4).

HRMS (ESI) calc: $[M – BF_4]^+$ ($C_{20}H_{23}FIO_3S^+$) 489.0391; measured: 489.0375 = 3.27 ppm difference.

IR (neat) v_{max}/cm^{-1}: 3681, 2991, 2901, 1652, 1386, 1335, 1175, 1058, 985, 956, 898, 750, 689, 571, 541, 521.

(Z)-(3-((4-bromobenzyl)oxy)-2-fluoroprop-1-en-1-yl)(mesityl)iodonium BF_4 ***(3i)***

Synthesised according to conditions B, using 1-bromo-4-((prop-2-yn-1-yloxy)methyl)benzene (562 mg, 2.5 mmol, 1 equiv.), Ag_2CO_3 (69 mg, 0.25 mmol, 0.1 equiv.), 2-iodomesitylene (677 mg, 2.75 mmol, 1.1 equiv.), Selectfluor (1.33 g, 3.75 mmol, 1.5 equiv.), K_2CO_3 (1.04 g, 7.5 mmol, 3 equiv.), and pyridine.9HF (2.33 mL), in $MeNO_2$ (17.6 mL). Trituration with Et_2O (3 x 30 mL). Trituration with Et_2O (2 x 25 mL) and evaporation of the residual solvent under reduced pressure yielded a cream solid (1.09 g, 65% yield based on a purity of 86% determined by quantitative ^{19}F NMR assay). The compound was spectroscopically pure by ^{1}H, ^{13}C, and ^{19}F NMR.

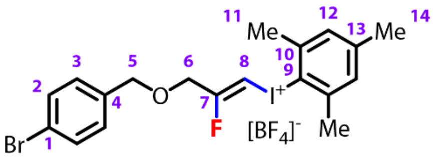

^{1}H NMR (500 MHz, $C(CD_3)_2O$): δ 7.56 – 7.47 (2H, m, H^{2}), 7.28 (2H, s, H^{12}) 7.28 (2H, d, J = 8.4Hz, H^{3}), 7.16 (1H, d, J = 35.0 Hz, H^{8}), 4.58 (2H, s, H^{5}), 4.51 (2H, d, J = 11.3 Hz, H^{6}), 2.72 (6H, s, H^{11}), 2.37 (3H, s, H^{14}).

^{13}C NMR (126 MHz, $C(CD_3)_2O$): δ 169.2 (1C, d, J = 276.8 Hz, C^{7}), 145.3 (1C, s, C^{13}), 143.4 (2C, s, C^{10}),137.7 (1C, s, C^{4}), 132.3 (2C, s, C^{2}), 131.0 (2C, s, C^{12}), 130.7 (2C, s, C^{3}), 122.2 (1C, s, C^{1}), 120.6 (1C, s, C^{9}), 78.1 (1C, d, J = 18.5 Hz, C^{8}), 72.8 (1C, s, C^{5}), 67.2 (1C, d, J = 31.2 Hz, C^{6}), 30.6 (1C, s,), 27.0 (2C, s, C^{11}), 20.9 (1C, s, C^{14}).

^{19}F NMR (376 MHz, $C(CD_3)_2O$): δ -79.36 (1F, dt, J = 35.0, 11.3 Hz, F^{6}), -150.73 + -150.78 (BF_4).

IR (neat) v_{max}/cm^{-1}: 3105, 2971, 1651, 1589, 1485, 1450, 1380, 1364, 1300, 1286, 1234, 1056, 1032, 1008, 941, 869, 808, 774, 709, 677, 639, 542, 496, 481.

HRMS (ESI) calc: $[M-BF_4]^+$ ($C_{19}H_{20}BrFIO$) 488.9721; measured: 488.9723 = 0.40 ppm difference.

Melting point: 107 – 112 °C

(Z)-(3-chloro-2-fluoroprop-1-en-1-yl)(mesityl)iodonium BF_4 ***(3j)***

Synthesised according to conditions A, using propargyl chloride (181 μL, 2.5 mmol, 1 equiv.), Ag_2CO_3 (69 mg, 0.25 mmol, 0.1 equiv.), 2-iodomesitylene (677 mg, 2.75 mmol, 1.1 equiv.), Selectfluor (1.33 g, 3.75 mmol, 1.5 equiv.), and pyridine.9HF (2.36 mL), in $MeNO_2$ (17.6 mL). Trituration with Et_2O (3 x 15 mL) and evaporation of the residual solvent under reduced pressure yielded a solid (1.01 g, 86% yield based on a purity of 90% determined by quantitative ^{19}F NMR assay). The compound was spectroscopically pure by ^1H, ^{13}C, and ^{19}F NMR.

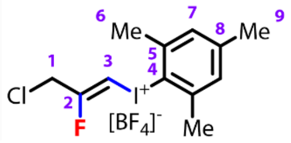

^1H NMR (500 MHz, $C(CD_3)_2O$): δ 7.36 (1H, d, J = 33.7 Hz, H^3), 7.33 – 7.31 (2H, m, H^7), 4.71 (2H d, J = 17.2 Hz, H^1), 2.75 (6H, s, H^6), 2.40 (3H, s, H^9).

^{13}C NMR (126 MHz, $C(CD_3)_2O$): δ 166.8 (1C, d, J = 272.7 Hz, C^2), 145.6 (1C, s, C^8), 143.6 (2C, s, C^5), 131.3 (2C, s, C^7), 120.5 (1C, s, C^4), 80.1 (1C, d, J = 20.1 Hz, C^3), 40.0 (1C, d, J = 29.8 Hz, C^1), 27.1 (2C, d, J = 1.1 Hz, C^6), 21.1 (1C, s, C^9).

^{19}F NMR (376 MHz, $C(CD_3)_2O$): δ -76.69 (1F, dt, J = 34.0, 17.3 Hz), -149.76 – -149.87 (BF_4).

HRMS (ESI) calc: $[M – BF_4]^+$ ($C_{12}H_{14}ClFI$) 338.9807; measured: 338.9802 = 1.74 ppm difference.

IR (neat) v_{max}/cm^{-1}: 1666, 1454, 1219, 1050, 1005, 909, 858, 765.

(Z)-(3-bromo-2-fluoroprop-1-en-1-yl)(mesityl)iodonium BF_4 ***(3k)***

Synthesised according to conditions A, using propargyl bromide (214 µL, 80% wt in toluene, 2.5 mmol, 0.1 equiv.), Ag_2CO_3 (69 mg, 0.25 mmol, 0.1 equiv.), 2-iodomesitylene (677 mg, 2.75 mmol,1.1 equiv.), Selectfluor (1.33 g, 3.75 mmol, 1.5 equiv.), and pyridine.9HF (2.36 mL), in $MeNO_2$ (17.6 mL). Trituration with Et_2O (3 x 5 mL) and evaporation of the residual solvent under reduced pressure yielded a light-yellow solid (730 mg, 53% yield based on a purity of 86% determined by quantitative ^{19}F NMR assay). The compound was spectroscopically pure by ^{1}H, ^{13}C, and ^{19}F NMR.

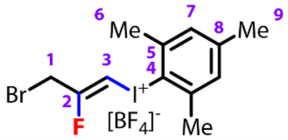

^{1}H NMR (500 MHz, $C(CD_3)_2O$): δ 7.23 (2H, s, H^7), 6.74 (1H, d, J = 33.3 Hz, H^3), 4.28 (2H, d, J = 19.2 Hz, H^1), 2.6 (6H, s, H^6), 2.36 (3H, s, H^9).

^{13}C NMR (126 MHz, $C(CD_3)_2O$): δ 166.75 (1C, d, J = 270.4 Hz, C^2), 145.40 (1C, s, C^8), 143.45 (2C, s, C^5), 131.08 (2C, s, C^7), 120.35 (1C, s, C^4), 79.18 (1C, d, J = 20.7 Hz, C^3), 26.97 (d, J = 1.1 Hz, C^1), 24.95 (d, J = 29.3 Hz, C^6), 20.93 (1C, s, C^9).

^{19}F NMR (376 MHz, $C(CD_3)_2O$): δ -74.76 (1F, dt, J = 33.4, 19.5 Hz, F^2, -149.34 – -149.41 (BF_4).

HRMS (ESI) calc: $[M – BF_4]^+$ ($C_{12}H_{14}FBrI$) 382.9308; measured: 382.9301 = -1.8 ppm difference.

IR (neat) v_{max}/cm^{-1}: 3095, 2981, 1698, 1649, 1587, 1452, 1382, 1365, 1283, 1227, 1049, 1027, 942, 897, 871, 854, 763, 695, 683, 542, 520, 488.

(Z)-(2-fluoro-3-((methylsulfonyl)oxy)prop-1-en-1-yl)(mesityl)iodonium BF_4 ***(3l)***

Synthesised according to conditions B using prop-2-yn-1-yl methanesulfonate (168 mg, 1.25 mmol, 1 equiv.), Ag_2CO_3 (35.4 mg, 0.125 mmol, 0.1 equiv.), 2-iodomesitylene (338 mg, 1.375 mmol, 1.1 equiv.), Selectfluor (664 mg, 1.875 mmol, 1.5 equiv.), K_2CO_3 (517 mg, 3.75 mmol, 3 equiv.), and pyridine.9HF (1.8 mL), in $MeNO_2$ (8.8 mL). Trituration with Et_2O (3 x 15 mL) and evaporation of the residual solvent under reduced pressure yielded (444 mg, 61% yield based on a purity of 83% determined by quantitative ^{19}F NMR assay). The compound was spectroscopically pure by ^{1}H, ^{13}C, and ^{19}F NMR.

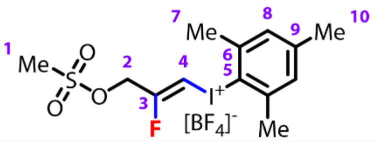

^{1}H NMR (400 MHz, $C(CD_3)_2O$): δ 7.38 (1H, d, J = 34.2 Hz, H^4), 7.30 (2H, s, H^8), 5.21 (2H, dd, J =13.3, 0.9 Hz, H^2), 3.21 (3H, s, H^1), 2.74 (6H, s, H^7), 2.38 (3H, s, H^{10}).

^{13}C NMR (126 MHz, $C(CD_3)_2O$): δ 165.2 (1C, d, J = 274.7 Hz, C^3), 145.5 (1C, s, C^9), 143.5 (2C, s, C^6), 131.1 (2C, s, C^8), 120.5 (1C, s,C^5), 81.0 (1C, d, J = 18.5 Hz, C^4), 65.2 (1C, d, J = 32.1 Hz, C^2), 37.9 (1C, s,C^1), 27.1 (2C, s, C^7), 20.9 (1C, s, C^{10}).

^{19}F NMR (376 MHz, $C(CD_3)_2O$): δ -81.05 (1F, dt, J = 34.1, 13.4 Hz, F^3), -150.85 + -150.90 (BF_4).

HRMS (ESI) calc: $[M\text{-}BF_4]^+$ ($C_{13}H_{17}O_3FSI$) 398.9922; measured: 398.9913 = 2.25 ppm difference.

IR (neat) v_{max}/cm^{-1}: 3108, 2966, 1659, 1453, 1359, 1297, 1174, 1057, 965, 895, 813, 764, 701, 686, 627, 521, 493, 463.

Melting point: 98 - 103°C

(Z)-(2-fluoro-3-(tosyloxy)prop-1-en-1-yl)(mesityl)iodonium BF_4 **(3m)**

Synthesised according to conditions A using 4-methylbenzenesulfonate (263 mg, 1.25 mmol, 1 equiv.), Ag_2CO_3 (35.4 mg, 0.125 mmol, 0.1 equiv.), 2-iodomesitylene (338 mg, 1.375 mmol, 1.1 equiv.), Selectfluor (664 mg, 1.875 mmol, 1.5 equiv.), and pyridine.9HF (1.18 mL), in $MeNO_2$ (8.8 mL). Trituration with Et_2O (3 x 15 mL) and evaporation of the residual solvent under reduced pressure yielded a light-brown solid (466 mg, 53% yield based on a purity of 80% determined by quantitative ^{19}F NMR assay). The compound was spectroscopically pure by ^{1}H, ^{13}C, and ^{19}F NMR.

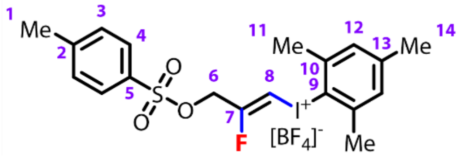

^{1}H NMR (400 MHz, $C(CD_3)_2O$): δ 7.83 – 7.77 (2H, m, H^4), 7.52 – 7.46 (2H, m, H^3), 7.28 (1H, d, J= 34.1 Hz, H^8), 7.28-7.26 (2H, m, H^{12}), 5.06 (2H, d, J = 13.9 Hz, H^6), 2.68 (6H, s, H^{11}), 2.46 (3H, s, H^1), 2.37 (3H, s, H^{14}).

^{13}C NMR (126 MHz, $C(CD_3)_2O$): δ 164.7 (1C, d, J = 275.0 Hz, C^7), 146.9 (1C, s, C^5), 145.5 (1C, s, C^{13}), 143.5 (2C, s, C^{10}), 133.1 C^2), 131.2 (1C, s, C^3), 131.1 (2C, s, C^{12}), 128.9 (2C, s, C^3), 120.3 (1C, s, C^9), 81.0 (1C, d, J = 18.4 Hz, C^6), 65.5 (1C, d, J = 31.3 Hz, C^6), 27.0 (2C, s, C^{11}), 21.6 (1C, s, C^1), 20.9 (1C, s, C^{14}).

^{19}F NMR (376 MHz, $C(CD_3)_2O$): δ -81.25 (1F, dt, J = 34.0, 13.9 Hz, F^7), -150.60 + -150.66 (BF_4).

HRMS (ESI) calc: $[M – BF_4]^+$ ($C_{19}H_{21}FIO_3S$) 475.0235; measured: 475.0240 = 1.05 ppm difference.

IR (neat) ν_{max}**/cm^{-1}:** 3664, 1651, 1479, 1394, 1186, 897, 829, 589.

Melting point: 100 – 106 °C

(Z)-(2-fluoro-3-(4-nitrophenoxy)prop-1-en-1-yl)(mesityl)iodonium BF_4 ***(3n)***

Synthesised according to conditions A, using 1-nitro-4-(prop-2-yn-1-yloxy)benzene (89 mg, 0.5 mmol, 1 equiv.), Ag_2CO_3 (13.8 mg, 0.05 mmol, 0.1 equiv.), 2-iodomesitylene (135 mg, 0.55 mmol, 1.1 equiv.), Selectfluor (266 mg, 0.75 mmol, 1.5 equiv.), and pyridine.9HF (470 µL), in dimethyl carbonate (3.53 mL). Trituration with Et_2O (3 x 15 mL) and evaporation of the residual solvent under reduced pressure yielded a white solid (195 mg, 56% yield based on a purity of 76% determined by quantitative ^{19}F NMR assay). The compound was spectroscopically pure by ^{1}H, ^{13}C, and ^{19}F NMR.

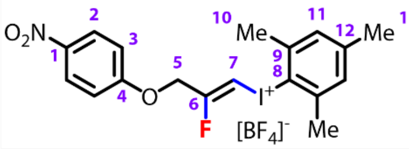

^{1}H NMR (500 MHz, CD_3CN): δ 8.21 – 8.16 (2H, m, H^2), 7.22 – 7.19 (2H, m, H^{11}), 7.10 – 7.05 (2H, m, H^3), 6.78 (1H, d, J = 34.5 Hz, H^7), 5.02 (2H, dd, J = 9.2, 1.1 Hz, H^5), 2.59 (6H, s, H^{10}), 2.35 (3H, s, H^{13}).

^{13}C NMR (126 MHz, CD_3CN): δ 167.0 (1C, d, J = 275.1 Hz, C^6), 162.9 (1C, s, C^1), 146.0 (1C, s, C^{12}),143.6 (2C, d, J = 7.8 Hz, C^9), 143.5 (1C, s, C^4), 131.3 (2C, s, C^{11}), 126.9 (2C, s, C^2), 120.4 (1C, s, C^8), 116.3 (2C, s, C^3), 78.8 (1C, d, J = 18.6 Hz, C^7), 65.7 (1C, d, J = 33.9 Hz, C^5), 27.2 (2C, d, J = 1.1 Hz, C^{10}), 21.0 (1C, s, C^{13}).

^{19}F NMR (376 MHz, $C(CD_3)_2O$): δ -79.62 (1F, dt, J = 34.4, 9.0 Hz, F^6), -151.34 + -151.40 (BF_4).

HRMS (ESI) calc: $[M\text{-}BF_4]^+$ ($C_{18}H_{18}NO_3FI$) 442.0315; measured: 442.0319 = 0.9 ppm difference.

IR (neat) v_{max}/cm^{-1}: 3108, 2987, 2927, 1654 1612, 1591, 1506, 1494, 1451, 1339, 1302, 1265, 1223, 1180, 1026, 945, 879, 861, 847, 764, 750, 708, 690, 630, 615, 539, 521, 492.

Melting point: 170 – 173 °C

(Z)-(2-fluoro-3-(4-fluorophenoxy)prop-1-en-1-yl)(mesityl)iodonium BF_4 ***(3o)***

Synthesised according to conditions B, using 1-fluoro-4-(prop-2-yn-1-yloxy)benzene (188 mg, 1.25 mmol, 1 equiv.), Ag_2CO_3 (35.4 mg, 0.125 mmol, 1 equiv.), 2-iodomesitylene (338 mg, 1.375 mmol,1.1 equiv.), Selectfluor (664 mg, 1.875 mmol, 1.5 equiv.), K_2CO_3 (518 mg, 3.75 mmol, 3 equiv.), and pyridine.9HF (1.18 mL), in $MeNO_2$ (8.8 mL). Trituration with Et_2O (3 x 15 mL) and evaporation of the residual solvent under reduced pressure yielded a dark red waxy solid. (468 mg, 63% yield based on a purity of 84%, as determined by ^{19}F NMR, relative to an internal standard). The compound was spectroscopically pure by ^{1}H, ^{13}C, and ^{19}F NMR.

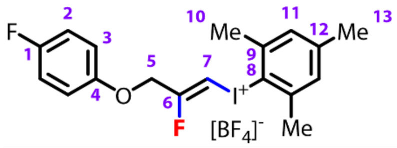

^{1}H NMR (500 MHz, $C(CD_3)_2O$): δ 7.45 – 7.41 (2H, m, H^{2}), 7.31 (1H, d, J = 34.8 Hz, H^{7}), 7.28 (2H, s, H^{11}), 6.99 – 6.94 (4H, m, H^{3}), 5.13 (2H, dd, J = 10.3, 1.1 Hz, H^{5}), 2.70 (6H, s, H^{10}), 2.38 (3H, s, H^{13}).

^{13}C NMR (126 MHz, $C(CD_3)_2O$): δ 167.9 (1C, d, J = 275.9 Hz, C^{6}), 158.9 (1C, d, J = 238.4 Hz, C^{1}),154.4 (1C, d, J = 2.3 Hz, C^{4}), 145.4 (1C, s, C^{12}), 143.4 (2C, s, C^{9}), 131.1 (2C, s, C^{11}), 120.5, (1C, s, C^{8}),117.6 (2C, d, J = 8.3 Hz, C^{3}), 116.8 (2C, d, J = 23.5 Hz, C^{2}), 78.8 (1C, d, J = 18.2 Hz, C^{7}), 66.0 (1C, d, J =32.6 Hz, C^{5}), 27.0 (2C, d, J = 1.3 Hz, C^{10}), 20.9 (1C, s, C^{13}).

^{19}F NMR (376 MHz, $C(CD_3)_2O$): δ -80.06 (1F, dt, J = 34.7, 10.3 Hz, F^{6}), -123.34 (1F, tt, J = 8.5, 4.5 Hz, F^{1}), -151.69 + -150.75 (BF_4).

HRMS (ESI) calc: $[M-BF_4]^+$ ($C_{18}H_{18}OF_2I$) 415.0365; measured: 415.0367 = 0.48 ppm difference.

IR (neat) v_{max}**/cm^{-1}:** 1627, 1602, 1509, 1456, 1283, 1239, 1165, 944, 846, 765, 556, 521.

Melting point: 98 – 103 °C.

(Z)-(3-(4-chlorophenoxy)-2-fluoroprop-1-en-1-yl)(mesityl)iodonium BF_4 **(3p)**

Synthesised according to conditions A, using 1-chloro-4-(prop-2-yn-1-yloxy)benzene (83 mg, 0.5 mmol, 1 equiv.), Ag_2CO_3 (13.8 mg, 0.05 mmol, 0.1 equiv.), 2-iodomesitylene (135 mg, 0.55 mmol, 1.1 equiv.), Selectfluor (266 mg, 0.75 mmol, 1.5 equiv.), and pyridine.9HF (470 µL), in dimethyl carbonate (3.53 mL). Trituration with Et_2O (3 x 15 mL) and evaporation of the residual solvent under reduced pressure yielded a cream solid (177 mg, 54% yield based on a purity of 79% determined by ^{19}F NMR, relative to an internal standard). The compound was spectroscopically pure by ^{1}H, ^{13}C, and ^{19}F NMR.

The procedure was repeated according to conditions B on a 1.25, using nitromethane as the solvent mmol scale, affording the same product in 56% yield based on a purity of 91% determined by ^{19}F NMR, relative to an internal standard). The compound was spectroscopically pure by ^{1}H, ^{13}C, and ^{19}F NMR.

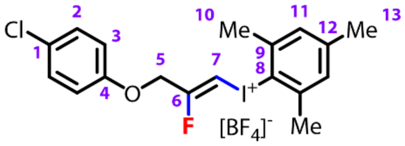

^{1}H NMR (500 MHz, $C(CD_3)_2O$): δ 7.31 (1H, d, J = 31.3 Hz, H^7), 7.31 – 7.28 (2H, m, H^2), 7.28 – 7.26 (2H, m, H^3), 7.04 – 6.99 (2H, s, H^{11}), 5.12 (2H, dd, J = 10.6, 0.9 Hz, H^5), 2.70 (6H, s, H^{10}), 2.38 (3H, s, H^{13}).

^{13}C NMR (126 MHz, $C(CD_3)_2O$): δ 167.5 (1C, d, J = 275.7 Hz, C^6), 157.0 (1C, s, C^4), 145.4 (1C, s, C^{12}),143.4 (2C, s, C^9), 131.0 (2C, s, C^{11}), 130.3 (2C, s, C^2), 127.5 (1C, s, C^1), 120.5 (1C, s, C^8), 117.7 (2C, s, C^3), 79.1 (1C, d, J = 18.3 Hz, C^7), 65.5 (1C, d, J = 32.6 Hz, C^5), 27.0 (2C, s, C^{10}), 20.9 (1C, s, C^{13}).

^{19}F NMR (283 MHz, $C(CD_3)_2O$): δ -80.55 (dt, J = 35.0, 10.7 Hz, F^6), -151.05 + -151.10 (BF_4).

HRMS (ESI) calc: $[M-BF_4]^+$ ($C_{18}H_{18}ClFIO$) 431.0069; measured: 431.0082 = 3.01 ppm difference.

IR (neat) v_{max}/cm^{-1}: 3114, 2923, 1663, 1582, 1490, 1453, 1385, 1281, 1242, 1043, 908, 858, 829, 720, 685, 521.

Melting points: 110 - 116 °C

(Z)-(3-(4-bromophenoxy)-2-fluoroprop-1-en-1-yl)(mesityl)iodonium BF_4 ***(3q)***

Synthesised according to conditions A, using 1-bromo-4-(prop-2-yn-1-yloxy)benzene (106 mg, 0.5 mmol, 1 equiv.), Ag_2CO_3 (13.8 mg, 0.05 mmol, 0.1 equiv.), 2-iodomesitylene (135 mg, 0.55 mmol, 1.1 equiv.), Selectfluor (266 mg, 0.75 mmol, 1.5 equiv.), and pyridine.9HF (470 µL), in dimethyl carbonate (3.53 mL). Trituration with Et_2O (3 x 15 mL) and evaporation of the residual solvent under reduced pressure yielded a white solid (200 mg, 64% based on a purity of 90% determined by quantitative ^{19}F NMR assay). The compound was spectroscopically pure by ^1H, ^{13}C, and ^{19}F NMR.

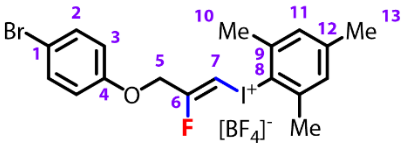

^{1}H NMR (500 MHz, $C(CD_3)_2O$): δ 7.45 – 7.41 (2H, m, H^2), 7.30 (1H, d, J = 34.7 Hz, H^7), 7.29 – 7.25 (2H, m, H^{11}), 6.99 – 6.93 (2H, m, H^3), 5.12 (2H, dd, J = 10.6, 1.0 Hz, H^5), 2.69 (6H, s, H^{10}), 2.38 (3H, s, H^{13}).

^{13}C NMR (126 MHz, $C(CD_3)_2O$): δ 167.5 (1C, d, J = 275.7 Hz, C^6), 157.4 (1C, s, C^4), 145.3 (1C, s, C^{12}), 143.4 (2C, s, C^9), 133.3 (1C, s, C^2), 131.0 (2C, s, C^{11}), 120.6 (1C, s, C^8), 118.2 (2C, s, C^3), 114.8 (1C, s, C^1), 79.1 (1C, d, J = 18.3 Hz, C^7), 65.4 (1C, d, J = 32.5 Hz, C^5), 27.0 (2C, s, C^{10}), 21.0 (1C, s, C^{13}).

^{19}F NMR (376 MHz, $C(CD_3)_2O$): δ -80.25 (1F, d, J = 36.1 Hz, F^6), -150.61 + -151.67 (BF_4).

HRMS (ESI) calc: $[M-BF_4]^+$ ($C_{18}H_{18}OBrFI$) 474.9564 ; measured: 474.9571 = 1.47 ppm difference.

IR (neat) v_{max}/cm^{-1}: 1406, 1394, 1241, 1066, 892, 879.

Melting point: 125 – 130 °C.

(Z)-(2-fluoro-3-phenoxyprop-1-en-1-yl)(mesityl)iodonium BF_4 ***(3r)***

Synthesised according to conditions B, using (prop-2-yn-1-yloxy)benzene (330 mg, 2.5 mmol, 1 equiv.), Ag_2CO_3 (69 mg, 0.125 mmol, 1 equiv.), 2-iodomesitylene (676 mg, 1.375 mmol,1.1 equiv.), Selectfluor (1.33 g, 1.875 mmol, 1.5 equiv.), K_2CO_3 (1.04 g, 3.75 mmol, 3 equiv.), and pyridine.9HF (2.33 mL), in $MeNO_2$ (17.7 mL). Trituration with Et_2O (2 x 50 mL) and evaporation of the residual solvent under reduced pressure yielded a white solid (983 mg, 72% yield based on a purity of 88% determined by quantitative ^{19}F NMR assay). The compound was spectroscopically pure by ^1H, ^{13}C, and ^{19}F NMR.

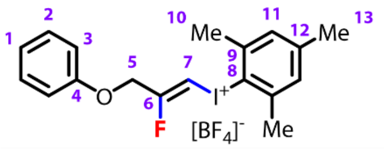

^{1}H NMR (600 MHz, $C(CD_3)_2O$): δ 7.29 (1H, dt, *J* = 34.9, 1.1 Hz, H^7), 7.31 – 7.28 (2H, m, H^2), 7.28 – 7.26 (2H, m, H^{11}), 7.05 – 6.98 (1H, m, H^1), 7.01 (2H, tt, *J* = 7.4, 1.0 Hz, H^3), 6.99 – 6.96 (2H, m, H^3), 5.09 (2H, dd, *J* = 10.2, 1.0 Hz, H^6), 2.70 (6H, s, H^{10}), 2.37 (3H, s, H^{13}).

^{13}C NMR (151 MHz, $C(CD_3)_2O$): δ 168.1 (1C, d, *J* = 275.6 Hz, C^6), 158.2 (1C, s, C^4) , 145.3 (1C, s, C^{13}),143.4 (2C, s, C^9), 131.0 (2C, s, C^{11}), 130.5 (2C, s, C^2), 123.0 (1C, s, C^1), 120.5 (1C, s, C^8), 115.9 (2C, s, C^3), 78.6 (d, *J* = 18.5 Hz C^5), 65.2 (d, *J* = 32.5 Hz, C^7), 27.0 (2C, s, C^{10}), 20.9 (1C, s, C^{13}).

^{19}F NMR (376 MHz, $C(CD_3)_2O$): δ -79.88 (1F, dt, *J* = 34.8, 9.9 Hz), -150.78 + -150.83 (*BF_4*).

HRMS (ESI) calc: $[M – BF_4]^+$ ($C_{18}H_{19}FIO$) 397.0459; measured: 397.0468 = 2.1 ppm difference.

IR (neat) v_{max}**/cm^{-1}:** 3106, 1666, 1588, 1490, 1445, 1381, 1292, 1231, 1194, 1018, 941, 915, 875, 819, 786, 764, 696, 520, 493.

Melting point: 105 – 110 °C

(Z)-(2-fluoro-3-(2-methoxyphenoxy)prop-1-en-1-yl)(mesityl)iodonium ***(3s)***

The Z-FVI formation step was conducted by employing conditions B, using 1-methoxy-2-(prop-2-yn-1-yloxy)benzene (203 mg, 1.25 mmol, 1 equiv.), Ag_2CO_3 (35.4 mg, 0.125 mmol, 0.1 equiv.), 2-iodomesitylene (338 mg, 1.375 mmol, 1.1 equiv.), Selectfluor (664 mg, 1.875 mmol, 1.5 equiv.), K_2CO_3 (518 mg, 3.75 mmol, 3 equiv.), and pyridine.9HF (1.18 mL), in $MeNO_2$ (8.82 mL), affording the compound in 39% NMR yield after work-up. The ^{19}F NMR and HRMS data of the crude mixture was in alignment with *Z*-FVI formation.

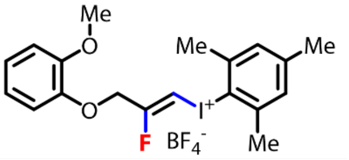

^{19}F NMR (376 MHz, CH_3CN): δ -77.94 (1F, dt, *J* = 34.1, 11.1 Hz).

HRMS (ESI) calc: $[M – BF_4]^+$ ($C_{19}H_{21}FIO_2$) 427.0565; measured: 427.0568 = 0.7 ppm difference.

(Z)-1-((2-fluoro-3-iodoallyl)oxy)-2-methoxybenzene: The crude product was dissolved in MeCN (10 mL), transferred to a glass vial with a stirrer bar, and TEAI (354 mg, 1.38 mmol, 1.1 equiv. w.r.t the alkyne from the first step) was added. The mixture was stirred at 60 °C for 18 hours, and the solvent was subsequently evaporated under reduced pressure. The resulting crude mixture was subjected to flash-column chromatography (0 to 30 % Et_2O in pentane) to afford the product as a colourless oil (121 mg, 31% over two steps).

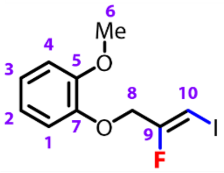

^{1}H NMR (500 MHz, $CDCl_3$): δ 7.01 (1H, ddd, *J* = 8.2, 6.7, 2.2 Hz, H^3), 6.94 – 6.87 (3H, m, H1-3), 5.78 (1H, d, *J* = 33.9 Hz, H^{10}), 4.67 (2H, dd, *J* = 10.3, 0.9 Hz, H^8), 3.87 (3H, s, H^6).

^{13}C NMR (126 MHz, $CDCl_3$): δ 161.4 (1C, d, *J* = 261.1 Hz, C^9), 150.3 (1C, s, C^5), 147.1 (1C, s, C^7), 123.2 (1C, s, C^3), 121.0 (1C, s, C^2), 115.9 (1C, s, C^1), 112.4 (1C, s, C^4), 67.3 (d, *J* = 35.1 Hz, C^8), 56.7 (d, *J* = 23.3 Hz, C^{10}), 56.0 (1C, s, C^6)

^{19}F NMR (376 MHz, $C(CD_3)_2O$): δ -90.05 (1F, dt, *J* = 33.9, 10.4 Hz, F^9).

HRMS (EI) calc: $[M]^+$ ($C_{10}H_{10}O_2FI$) 307.9704; measured: 307.9705 = 0.32 ppm difference.

IR (neat) ν_{max}**/cm^{-1}:** 3086, 2937, 2836, 1660, 1592, 1500, 1454, 1379, 1328, 1251, 1217, 1177, 1116, 1044, 1024, 893, 870, 835, 741, 650, 579, 536, 487, 460

(Z)-(3-(3,5-dibromophenoxy)-2-fluoroprop-1-en-1-yl)(mesityl)iodonium BF_4 ***(3t)***

Synthesised according to conditions B, using 1,3-dibromo-5-(prop-2-yn-1-yloxy)benzene (325 mg, 1.25 mmol, 1 equiv.), Ag_2CO_3 (35.4 mg, 0.125 mmol, 0.1 equiv.), 2-iodomesitylene (338 mg, 1.375 mmol, 1.1 equiv.), Selectfluor (664 mg, 1.875 mmol, 1.5 equiv.), K_2CO_3 (518 mg, 3.75 mmol, 3 equiv.), and pyridine.9HF (1.18 mL), in $MeNO_2$ (8.8 mL). Trituration with Et_2O (3 x 15 mL) and evaporation of the residual solvent under reduced pressure yielded a white solid (686 mg, 73% yield based on a purity of 85% determined by ^{19}F NMR, relative to an internal standard). The compound was spectroscopically pure by ^{1}H, ^{13}C, and ^{19}F NMR.

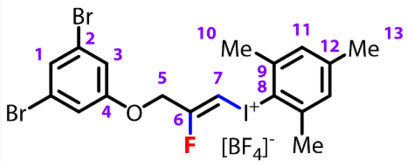

^{1}H NMR (600 MHz, $C(CD_3)_2O$): δ 7.39 (1H, t, *J* = 1.6 Hz, H^1), 7.35 (1H, dt, *J* = 34.9, 1.0 Hz, H^7), 7.27 (2H, s, H^{11}), 7.22 2H, d, *J* = 1.6 Hz, H^3), 5.21 (2H, dd, *J* = 10.1, 1.0 Hz), 2.70 (6H, s, H^{10}), 2.37 (3H, s, H^{13}).

^{13}C NMR (151 MHz, $C(CD_3)_2O$): δ 167.1 (1C, d, *J* = 274.9 Hz, C^6), 159.5 (1C, s, C^4), 145.3 (1C, s, C^{12}),143.3 (2C, s, C^9), 131.1 (2C, s, C^{11}), 128.4 (1C, s, C^1), 123.9 (1C, s, C^2) , 120.6 (2C, s, C^8) , 118.6 (1C, s, C^3), 79.4 (1C, d, *J* = 18.3 Hz, C^7), 65.6 (1C, d, *J* = 33.5 Hz, C^5), 27.0 (2C,s, C^{10}), 21.0 (1C, s, C^{13}).

^{19}F NMR (376 MHz, $C(CD_3)_2O$): δ -80.33 (1F, dt, *J* = 34.9, 9.7 Hz), -150.77 + -150.83 (*BF_4*).

HRMS (ESI) calc: $[M\text{-}BF_4]^+$ ($C_{18}H_{17}OBr_2FI$) 552.8675 ; measured: 552.8669 = 1.1 ppm difference.

IR (neat) v_{max}/cm^{-1}: 1662, 1582, 1562, 1395, 1250, 1230, 892, 745, 667, 520.

Melting point: 124 – 128 °C.

(Z)-(2-fluoro-3-(2,4,6-trichlorophenoxy)prop-1-en-1-yl)(mesityl)iodonium BF_4 **(3u)**

Synthesised according to conditions B, using 1,3,5-trichloro-2-(prop-2-yn-1-yloxy)benzene (294 mg, 1.25 mmol, 1 equiv.), Ag_2CO_3 (35.4 mg, 0.125 mmol, 0.1 equiv.), 2-iodomesitylene (338 mg, 1.375 mmol, 1.1 equiv.), Selectfluor (664 mg, 1.875 mmol, 1.5 equiv.), K_2CO_3 (518 mg, 3.75 mmol, 3 equiv.), and pyridine.9HF (1.178 mL), in $MeNO_2$ (8.82 mL). Trituration with Et_2O (3 x 30 mL) and evaporation of the residual solvent under reduced pressure yielded a cream solid (524 mg, 59% yield based on a purity of 83% determined by ^{19}F NMR, relative to an internal standard). The compound was spectroscopically pure by ^1H, ^{13}C, and ^{19}F NMR.

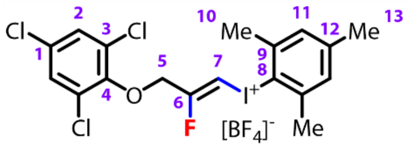

^1H NMR (500 MHz, $C(CD_3)_2O$): δ 7.51 (2H, s, H^2), 7.39 (1H, dt, J = 34.0, 0.7 Hz, H^7), 7.31 (2H, s, H^{11}), 5.11 (2H, d, J = 15.2 Hz, H^5), 2.73 (6H, s, H^{10}), 2.40 (3H, s, H^{13}).

^{13}C NMR (126 MHz, $C(CD_3)_2O$): δ 166.7 (1C, d, J = 276.8 Hz, C^6), 150.0 (1C, s, C^4), 145.5 (1C, s, C^{12}), 143.5 (2C, s, C^9), 131.4 (1C, s, C^1), 131.1 (2C, s, C^{11}), 130.7 (2C, s, C^3), 129.9 (2C, s, C^2), 120.5 (1C, s, C^8), 80.7 (1C, d, J = 18.6 Hz, C^7), 69.8 (1C, d, J = 30.3 Hz, C^5), 27.1 (2C, d, J = 1.3 Hz, C^{10}), 21.0 (1C, s, C^{13}).

^{19}F NMR (376 MHz, $C(CD_3)_2O$): δ -73.41 (1F, dt, J = 33.9, 15.2 Hz), -145.68 + -145.73 (BF_4).

HRMS (ESI) calc: $[M-BF_4]^+$ ($C_{18}H_{16}Cl_3FIO$) 498.9290 ; measured: 498.9282 = 1.6 ppm difference.

IR (neat) v_{max}/cm^{-1}: 3099, 2938, 1653, 1556, 1450, 1437, 1382, 1296, 1246, 1056, 1006, 987, 938, 898, 864, 801, 775, 725, 694, 579, 540, 523, 427.

Melting point: 152 – 157 °C.

(Z)-(3-(2,6-dimethylphenoxy)-2-fluoroprop-1-en-1-yl)(mesityl)iodonium BF_4 ***(3v)***

Synthesised according to conditions B, using 1,3-dimethyl-2-(prop-2-yn-1-yloxy)benzene (80 mg, 0.5 mmol, 1 equiv.), Ag_2CO_3 (13.8 mg, 0.05 mmol, 0.1 equiv.), 2-iodomesitylene (135 mg, 0.55 mmol, 1.1 equiv.), Selectfluor (266 mg, 0.75 mmol, 1.5 equiv.), K_2CO_3 (518 mg, 3.75 mmol, 3 equiv.), and pyridine.9HF (470 µL), in $MeNO_2$ (3.53 mL). Trituration with Et_2O (3 x 15 mL) and evaporation of the residual solvent under reduced pressure yielded a yellow solid (207 mg, 73% yield based on a purity of 90% determined by quantitative ^{19}F NMR assay.). The compound was spectroscopically pure by ^{1}H, ^{13}C, and ^{19}F NMR.

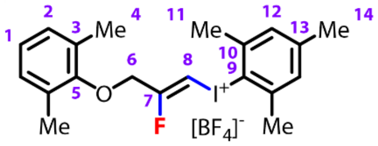

^{1}H NMR (500 MHz, $C(CD_3)_2O$): δ 7.34 (1H, d, *J* = 34.5 Hz, *H*^{8}), 7.32 (2H, s, *H*^{12}) 7.00 (2H, d, *J = 7.4 Hz*, *H*^{2}), 6.97 – 6.92 (1H, m, *H*^{1}), 4.85 (2H, dd, *J* = 13.2, 0.9 Hz, *H*^{6}), 2.74 (6H, s, *H*^{11}), 2.40 (3H, s, *H*^{14}), 2.17 (6H, s, *H*^{4}).

^{13}C NMR (126 MHz, $C(CD_3)_2O$): δ 168.5 (d, *J* = 276.4 Hz, *C*^{7}), 155.6 (1C, s, *C*^{5}), 145.4 (1C, s, *C*^{13}), 143.4 (2C, s, *C*^{10}), 131.4 (2C, s, *C*^{3}), 131.1 (2C, s, *C*^{12}), 129.9 (2C, s, *C*^{2}), 125.7 (1C, s, *H*^{1}), 120.5 (1C, s, *C*^{9}), 78.9 (d, *J* = 18.5 Hz, *C*^{8}), 68.7 (d, *J* = 30.6 Hz, *H*^{6}), 27.0 (d, *J* = 1.3 Hz, *C*^{11}), 21.0 (1C, s, *C*^{14}), 16.2 (2C, s, *C*^{4}).

^{19}F NMR (376 MHz, $C(CD_3)_2O$): δ -79.03 (1F, dt, *J* = 34.6, 13.1 Hz, *F*^{7}), -150.97 + -151.02(*BF_4*).

HRMS (ESI) calc: $[M\text{-}BF_4]^+$ ($C_{20}H_{23}OFI$) 425.0772; measured: 425.0775 = 0.71 ppm difference.

IR (neat) ν_{max}**/cm^{-1}:** 1662, 1478, 1452, 1381, 1292, 1192, 883, 780, 521.

Melting point: 133 – 137 °C.

(Z)-(2-fluoro-3-methoxyprop-1-en-1-yl)(mesityl)iodonium BF_4 ***(3w)***

Synthesised according to conditions A, using methyl propargyl ether (211 µL, 2.5 mmol, 1 equiv.), Ag_2CO_3 (69 mg, 0.25 mmol, 0.1 equiv.), 2-iodomesitylene (677 mg, 2.75 mmol, 1.1 equiv.), Selectfluor (1.33 g, 3.75 mmol, 1.5 equiv.), and pyridine.9HF (2.36 mL), in $MeNO_2$ (17.6 mL). Trituration with Et_2O (3 x 15 mL) and evaporation of the residual solvent under reduced pressure yielded a yellow solid (791 mg, 68% yield based on a purity of 90% determined by quantitative ^{19}F NMR assay). The compound was spectroscopically pure by ^{1}H, ^{13}C, and ^{19}F NMR.

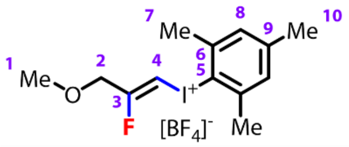

^{1}H NMR (400 MHz, $C(CD_3)_2O$): δ 7.22 (2H, s, H^8), 6.54 (1H, d, J = 34.5 Hz, H^4), 4.22 (2H, d, J = 10.7 Hz, H^2), 3.33 (3H, s, H^1), 2.61 (6H, s, H^7), 2.35 (3H, s, H^{10}).

^{13}C NMR (126 MHz, CD_3CN): δ 169.7 (1C, d, J = 278.4 Hz, C^3), 145.9 (1C, s, C^9), 143.5 (2C, s, C^6), 131.2 (2C, s C^8), 120.2 (1C, s, C^5), 77.1 (1C, d, J = 18.9 Hz, C^4), 69.3 (1C, d, J = 30.8 Hz, C^2), 59.4 (1C, s, C^1), 27.2 (2C, s, C^7), 21.0 (1C, s, C^{10}).

^{19}F NMR (376 MHz, CD_3CN): δ -78.53 (1F, dt, J = 34.7, 10.8 Hz, F^3), -151.56 + 151.62 (BF_4).

HRMS (ESI) calc: $[M – BF_4]^+$ ($C_{13}H_{17}FIO$) 335.0303; measured: 335.0298 = 1.4 ppm difference.

IR (neat) v_{max}**/cm^{-1}:** 3669, 3108, 2978, 2899, 1655, 1393, 1253, 1065, 898, 872, 765.

Melting point: 142 – 145 °C.

(Z)-(2-fluoro-3-hydroxy-3-methylbut-1-en-1-yl)(mesityl)iodonium BF4 ***(3x)***

Synthesised according to conditions B using 2-methyl-3-butyn-2-ol (120 μL, 1.25 mmol, 1 equiv.), Ag_2CO_3 (35.4 mg, 0.125 mmol, 0.1 equiv.), 2-iodomesitylene (338 mg, 1.375 mmol, 1.1 equiv.), Selectfluor (664 mg, 1.875 mmol, 1.5 equiv.), K_2CO_3 (518 mg, 3.75 mmol, 3 equiv.), and pyridine.9HF (1.18 mL), in $MeNO_2$ (8.8 mL). Trituration with Et_2O (3 x 15 mL) and evaporation of the residual solvent under reduced pressure yielded a cream solid (424 mg, 68% yield based on a purity of 87% determined by quantitative ^{19}F NMR assay). The compound was spectroscopically pure by ^{1}H, ^{13}C, and ^{19}F NMR.

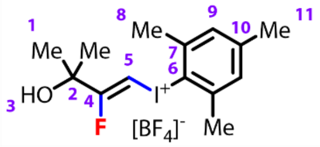

^{1}H NMR (500 MHz, C(CD$_3$)$_2$O): δ 7.29 (2H, s, H^9), 7.05 (1H, d, J = 34.8 Hz, H^5), 4.99 (1H, s, H^3), 2.72 (6H, s, H^8), 2.38 (3H, s, H^{11}), 1.43 (6H, t, J = 1.1 Hz, H^1).

^{13}C NMR (126 MHz, C(CD$_3$)$_2$O): δ 177.7 (1C, d, J = 283.4 Hz, C^4), 145.2 (1C, s, C^{10}), 143.4 (2C, s, C^7),131.0 (2C, s, C^9), 120.0 (1C, s, C^6), 75.4 (1C, d, J = 19.9 Hz, C^2), 72.1 (1C, d, J = 27.3 Hz, C^5), 27.5 (2C, d, J = 7.8 Hz, C^1), 27.0 (2C, d, J = 1.2 Hz, C^8), 20.9 (1C, s, C^{11}).

^{19}F NMR (377 MHz, C(CD$_3$)$_2$O): δ -80.16 (1F, d, J = 34.7 Hz, F^4), 151.09 + -151.14 (BF_4).

HRMS (ESI) calc: $[M – BF_4]^+$ ($C_{14}H_{19}FIO$) 349.0449; measured: 349.0459 = 2.73 ppm difference.

IR (neat) v_{max}/cm^{-1}: 3488, 3117, 1634, 1460, 1381, 1272, 1198, 1033, 850, 780, 742, 567.

Melting point: 165 – 168 °C.

(Z)-(2-fluoro-3-((N-(4-fluorobenzyl)-4-nitrophenyl)sulfonamido)prop-1-en-1-yl)(mesityl)iodonium BF4 ***(3y)***

Synthesised according to conditions A, using *N*-(4-fluorobenzyl)-4-nitro-*N*-(prop-2-yn-1-yl)benzenesulfonamide (436 mg, 1.25 mmol, 1 equiv.), Ag_2CO_3 (35.4 mg, 0.125 mmol, 0.1 equiv.), 2-iodomesitylene (338 mg,1.375 mmol, 1.1 equiv.), Selectfluor (664 mg, 1.875 mmol, 1.5 equiv.), and pyridine.9HF (1.18 mL), in $MeNO_2$ (8.82 mL). Trituration with Et_2O (3 x 15 mL) yielded a yellow solid (797 mg, 83% yield based on a purity of 92%, as determined by quantitative ^{19}F NMR assay). The compound was spectroscopically pure by ^{1}H, ^{13}C, and ^{19}F NMR.

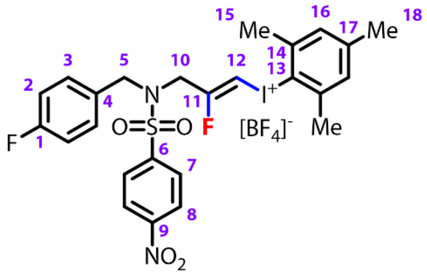

^{1}H NMR (500 MHz, CD_3CN): δ 8.35 (2H, d, *J* = 8.9 Hz, H^8), 8.02 (2H, d, *J* = 8.9 Hz, H^7), 7.20 (2H, s, H^{16}), 7.19 – 7.17 (2H, m, H^3), 6.94 (2H, t, J = 8.9 Hz H^2), 6.34 (1H, d, *J* = 33.9 Hz, H^{12}), 4.32 (2H, s, H^5), 4.22 (2H, d, *J* = 12.0 Hz, H^{10}), 2.49 (6H, s, H^{15}), 2.38 (3H, s, H^{18}).

^{13}C NMR (126 MHz, CD_3CN): δ 167.7 (d, *J* = 278.1 Hz, C^{11}), 163.4 (d, *J* = 245.4 Hz, C^1), 151.7 (1C, s, C^6), 146.0 (1C, s, C^9), 144.9 (1C, s, C^{17}), 143.5 (2C, s, C^{14}), 131.9 (d, *J* = 3.1 Hz, C^4), 131.6 (d, *J* = 8.4 Hz, C^3), 131.3 (2C, s, C^{16}), 129.8 (2C , s, C^7), 125.7 (2C, s, C^8), 120.2 (1C, s, C^{13}), 116.4 (d, *J* = 21.7 Hz,C^2), 78.3 (1C, d, *J* = 19.1 Hz, C^{12}), 53.0 (1C, s, C^5), 48.4 (d, *J* = 30.3 Hz, C^{10}), 27.2 (2C, s, C^{15}), 21.1 (1C, s, C^{18}).

^{19}F NMR (376 MHz, CD_3CN): δ -74.41 – 74.94 (1F, m, F^{11}), -113.51 – 113.78 (1F, m, F^1), -149.43 + -150.35 (*BF_4*).

HRMS (ESI) calc: $[M – BF_4]^+$ ($C_{25}H_{24}N_2O_4SF_2I$) 613.0464; measured: 613.0462 = 0.33 ppm difference.

IR (neat) v_{max}/cm^{-1}: 3095, 1652, 1605, 1532, 1509, 1449, 1366, 1351, 1314, 1226, 1172, 1062, 1001, 940, 905, 876, 858, 836, 782, 762, 744, 735, 684, 633, 599, 576, 519, 495, 464, 423.

Melting point: 160 – 165 °C.

(Z)-(3-(1,3-dioxoisoindolin-2-yl)-2-fluoroprop-1-en-1-yl)(mesityl)iodonium BF_4 ***(3z)***

Synthesised according to conditions A using 2-(prop-2-yn-1-yl)isoindoline-1,3-dione (232 mg, 1.25 mmol, 1 equiv.), Ag_2CO_3 (35.4 mg, 0.125 mmol, 0.1 equiv.), 2-iodomesitylene (338 mg, 1.375 mmol,1.1 equiv.), Selectfluor (664 mg, 1.875 mmol, 1.5 equiv.), and pyridine.9HF (1.18 mL), in $MeNO_2$ (8.8 mL). Trituration with Et_2O (3 x 15 mL) yielded a light brown solid (410 mg, 54% yield based on a purity of 89% as determined by quantitative ^{19}F NMR assay). The compound was spectroscopically pure by ^{1}H, ^{13}C, and ^{19}F NMR.

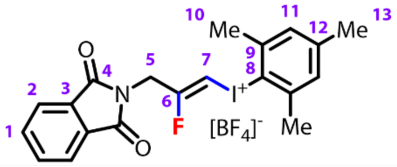

^{1}H NMR (500 MHz, CD_3CN): δ 7.90 – 7.80 (4H, m, H^{1+2}), 7.21 (2H, s, H^{11}), 6.64 (1H, d, J = 34.2 Hz, H^7), 4.64 (2H, d, J = 9.8 Hz, H^5), 2.58 (6H, s, H^{10}), 2.34 (3H, s, H^{13}).

^{13}C NMR (126 MHz, CD_3CN): δ 168.1 (2C, s, C^4), 166.9 (1F, d, J = 277.4 Hz, C^6), 145.9 (1C, s, C^{12}), 143.6 (2C, s, C^9), 135.8 (2C, s, C^{1+2}), 132.8 (2C, s, C^3), 131.2 (2C, s, C^{11}), 124.4 (2C, s, C^{1+2}), 120.2 (1C, s, C^8), 77.5 (1C, d, J = 19.1 Hz, C^7), 38.3 (1C, d, J = 33.8 Hz, C^5), 27.2 (2C, s, C^{10}), 21.0 (1C, s, C^{13}).

^{19}F NMR (376 MHz, CD_3CN): δ -75.12 (dt, J = 34.2, 9.7 Hz), -151.57 + -151.62 (*BF_4*).

HRMS (ESI) calc: $[M – BF_4]^+$ ($C_{20}H_{18}FINO_2$) 450.0361; measured: 450.0383 = 4.9 ppm difference.

IR (neat) ν_{max}**/cm^{-1}:** 1775, 1725, 1662, 1406, 1389, 1307, 1049, 939, 735, 715, 530.

(Z)-(3-((N-cyclopropyl-4-methylphenyl)sulfonamido)-2-fluoroprop-1-en-1-yl)(mesityl)iodonium BF₄ **(3aa)**

Synthesised according to the general synthetic procedure B using N-cyclopropyl-4-methyl-N-(prop-2-yn-1-yl)benzenesulfonamide (312 mg, 1.25 mmol, 1 equiv.), Ag_2CO_3 (35.4 mg, 0.125 mmol, 0.1 equiv.), 2-iodomesitylene (338 mg, 1.375 mmol, 1.1 equiv.), Selectfluor (664 mg, 1.875 mmol, 1.5 equiv.), K_2CO_3 (518 mg, 3.75 mmol, 3 equiv.), and pyridine.9HF (1.18 mL), in $MeNO_2$ (8.8 mL). Trituration with Et_2O (3 x 15 mL) and evaporation of the residual solvent under reduced pressure yielded a cream solid (635 mg, 65% yield based on a purity of 78% determined by quantitative ^{19}F NMR assay). The compound was spectroscopically pure by ^{1}H, ^{13}C, and ^{19}F NMR.

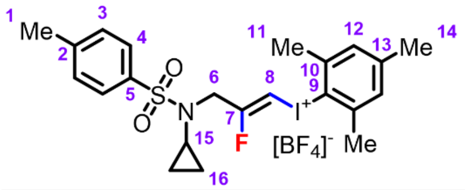

^{1}H NMR (500 MHz, $C(CD_3)_2O$): δ 7.75 (2H, d, *J* = 8.4 Hz, H^4), 7.48 – 7.40 (2H, m, H^3), 7.32 – 7.25 (2H, m, H^{12}), 7.15 (1H, d, *J* = 34.3, H^8), 4.37 (2H, dd, *J* = 13.4, 0.9 Hz, H^6), 2.71 (6H, s, H^{11}), 2.45 (3H, s, H^1), 2.38 (3H, s, H^{14}), 0.84 – 0.70 (2H, m, H^{16}), 0.68 – 0.57 (2H, m, H^{16}).

^{13}C NMR (126 MHz, $C(CD_3)_2O$): δ 168.9 (1C, d, *J* = 278.0 Hz, C^7), 145.3 (1C, s, C^{13}), 145.2 (1C, s, C^5), 143.4 (2C, s, C^{10}), 135.5 (1C, s, C^2), 131.0 (2C, s, C^{12}), 130.7 (2C, s, C^3), 128.7 (2C, s, C^4), 120.5 (1C, s, C^9), 78.6 (1C, d, *J* = 19.4 Hz, C^8), 51.2 (d, *J* = 29.4 Hz, C^6), 32.1, 30.6, 27.0 (2C, s, C^{11}), 21.46 (2C, s, C^1), 20.9 (1C, s, C^{14}), 7.8 (2C, s, C^{16}).

^{19}F NMR (376 MHz, $C(CD_3)_2O$): δ -75.16 (1F, dt, *J* = 34.2, 13.2 Hz, F^7), -151.03 (BF_4).

HRMS (ESI) calc: $[M – BF_4]^+$ ($C_{22}H_{26}NO_2SFI$) 514.0707; measured: 514.0709 = 0.39 ppm difference.

IR (neat) v_{max}/cm^{-1}: 3092, 2979, 2909, 1646, 1452, 1381, 1349, 1337, 1300, 1160, 1058, 1005, 933, 873, 853, 837, 819, 772, 726, 706, 667, 619, 542, 522, 490, 434.

Melting point: 127 – 133 °C

(Z)-(2-fluoro-3-((4-methylphenyl)sulfonamido)prop-1-en-1-yl)(mesityl)iodonium BF_4 ***(3ab)***

Synthesised according to conditions A, using 4-methyl-N-(prop-2-yn-1-yl)benzenesulfonamide (261 mg, 1.25 mmol, 1 equiv.), Ag_2CO_3 (35.4 mg, 0.125 mmol, 0.1 equiv.), 2-iodomesitylene (338 mg, 1.375 mmol, 1.1 equiv.), Selectfluor (664 mg, 1.875 mmol, 1.5 equiv.), and pyridine.9HF (1.18 mL), in either $MeNO_2$ (8.8 mL). Trituration with Et_2O (3 x 15 mL) and evaporation of the residual solvent under reduced pressure yielded a yellow oil (584 mg, 61% yield based on a purity of 73% determined by quantitative ^{19}F NMR assay). The compound was spectroscopically pure by ^{1}H, ^{13}C, and ^{19}F NMR.

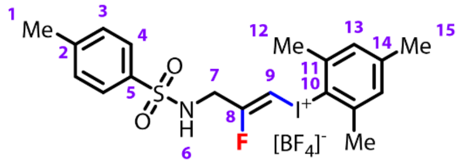

^{1}H NMR (500 MHz, $C(CD_3)_2O$): δ 7.72 (2H, d, *J* = 8.3 Hz, *H*^{4}), 7.40 – 7.36 (2H, m, *H*^{3}), 7.28 – 7.27 (2H, m, *H*^{13}), 7.20 (1H, t, *J* = 6.5 Hz, *H*^{6}), 7.02 (1H, d, *J*= 34.3, *H*^{9}), 4.10 (2H, ddd, *J* = 10.4, 6.3, 1.1 Hz, *H*^{7}), 2.69 (6H, s, *C*^{12}), 2.41 (3H, s, *H*^{1}), 2.37 (3H, s, *H*^{15}).

^{13}C NMR (126 MHz, $C(CD_3)_2O$): δ 169.5 (1C, d, *J* = 278.2 Hz, *C*^{8}), 145.4 (2C, s, *C*^{14}), 144.6 (d, *J* = 2.7 Hz, *H*^{2}), 143.5 (1C, s, *C*^{11}), 138.3 (1C, d, *J* = 8.6 Hz, *C*^{5}), 131.1 (2C, s, *C*^{13}), 130.7 (2C, s, *H*^{3}), 127.8 (2C, d, *J* = 2.0 Hz, *C*^{4}), 120.3 (1C, s, *C*^{10}), 77.3 (1C, d, *J* = 19.2 Hz, *C*^{9}), 43.4 (2H, dd, *J* = 32.3, 11.3 Hz, *C*^{7}), 27.0 (1C, s, *C*^{12}), 21.4 (1C, s, *C*^{1}), 20.9 (1C, s, *C*^{15}).

^{19}F NMR (376 MHz, $C(CD_3)_2O$): δ -76.48 (1F, d, *J* = 34.5 Hz, *F*^{8}), -151.12 (*BF_4*).

HRMS (ESI) calc: $[M – BF_4]^+$ ($C_{19}H_{22}NO_2SFI$) 474.0394; measured: 474.0385 = 1.90 ppm difference.

IR (neat) ν_{max}**/cm^{-1}:** 3541, 3295, 3099, 2925, 1655, 1597, 1450, 1381, 1327, 1304, 1289, 1230, 1156, 1051, 1032, 815, 706, 664, 549, 520.

(Z)-(2-fluoro-3-((N-(4-fluorobenzyl)-4-methylphenyl)sulfonamido)prop-1-en-1-yl)(mesityl)iodonium BF_4 ***(3ac)***

Synthesised according to the general synthetic procedure B using N-(4-fluorobenzyl)- 4-methyl-N-(prop-2-yn-1-yl)benzenesulfonamide (397 mg, 1.25 mmol, 1 equiv.), Ag_2CO_3 (35.4 mg, 0.125 mmol, 0.1 equiv.), 2-iodomesitylene (338 mg, 1.375 mmol, 1.1 equiv.), Selectfluor (664 mg, 1.875 mmol, 1.5 equiv.), K_2CO_3 (518 mg, 3.75 mmol, 3 equiv.), and pyridine.9HF (1.178 mL), in $MeNO_2$ (8.82 mL). Trituration with Et_2O (2 x 25 mL) yielded a light- yellow solid (595 mg, 61% yield based on a purity of 85% determined by ^{19}F NMR assay). The compound was spectroscopically pure by ^{1}H, ^{13}C, and ^{19}F NMR.

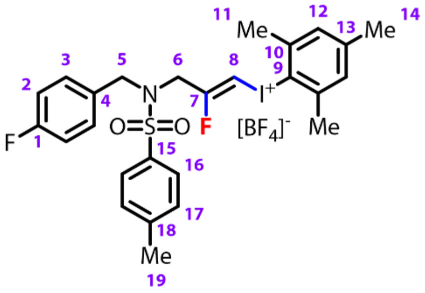

^{1}H NMR (500 MHz, CD_3CN): δ 7.73 – 7.70 (2H, m, H^{16}), 7.41 (2H, d, J = 7.9 Hz, H^{17}), 7.21 (2H, s, H^{3}) 7.20 – 7.14 (2H, m, H^{3}), 6.94 – 6.88 (2H, m, H^{2}), 6.30 (1H, d, J = 34.3 Hz, H^{8}), 4.22 (2H, s, H^{5}), 4.12 (2H, d, J = 11.2 Hz, H^{6}), 2.48 (6H, s, H^{11}), 2.45 (3H, s, $H^{\mathbf{19}}$), 2.38 (3H, s, H^{14}).

^{13}C NMR (126 MHz, CD_3CN): δ 168.6 (1C, d, J = 279.0 Hz, C^{7}), 163.4 (1C, d, J = 244.8 Hz, C^{1}), 145.9 (1C, s, C^{13}), 145.7 (1C, s, C^{18}), 143.5 (2C, s, C^{10}), 136.3, 132.5 (d, J = 3.1 Hz, C^{4}), 131.5 (d, J = 8.4 Hz, C^{3}), 131.3, 131.0, 128.4, 120.0 (1C, s, C^{9}), 116.3 (1C, d, J = 21.7 Hz, C^{2}), 77.7 (1C, d, J = 19.7 Hz, C^{8}), 53.1 (1C, s, C^{5}), 48.5 (d, J = 31.0 Hz, C^{6}), 27.2 (2C, s, C^{11}), 21.6 (1C, s, $C^{\mathbf{19}}$), 21.1 (1C, s, C^{14}).

^{19}F NMR (376 MHz, CD_3CN): δ -74.36 (1F, dt, J = 33.6, 10.8 Hz, F^{7}), -114.74 – -114.85 (1F, m, F^{1}), -151.52 + -151.57 (*BF_4*).

HRMS (ESI) calc: $[M – BF4]^+$ ($C_{26}H_{27}F_2INO_2S$) 582.0775; measured: 582.0761 = 2.4 ppm difference.

IR (neat) v_{max}/cm^{-1}: 3662, 3100, 2980, 2925, 1651, 1600, 1510, 1450, 1340, 1223, 1158, 1055, 908, 815, 756, 664, 579, 547, 519.

Melting point: 91-96°C

*(Z)-(2-fluoro-3-((4-methyl-N-(4-methylbenzyl)phenyl)sulfonamido)prop-1-en-1-yl)(mesityl)iodonium BF_4, **(3ad)***

Synthesised according to the general synthetic procedure employing B using N-(4-fluorobenzyl)-4-methyl-N-(prop-2-yn-1-yl)benzenesulfonamide (786 mg, 2.5 mmol, 1 equiv.), Ag_2CO_3 (69 mg, 0.25 mmol, 0.1 equiv.), 2-iodomesitylene (677 mg, 2.75 mmol, 1.1 equiv.), Selectfluor (1.33 g, 3.75 mmol, 1.5 equiv.), and pyridine.9HF (2.36 mL), in $MeNO_2$ (17.7 mL). Trituration with Et_2O (3 x 15 mL) and evaporation of the residual solvent under reduced pressure yielded a yellow solid (1.21 g, 60% yield based on a purity of 83% determined by quantitative ^{19}F NMR assay). The compound was spectroscopically pure by ^{1}H, ^{13}C, and ^{19}F NMR.

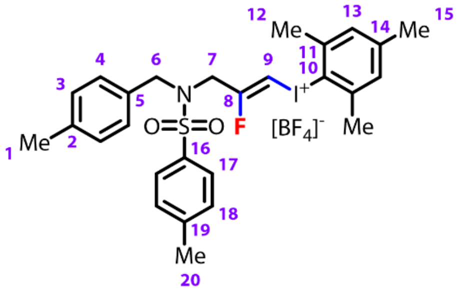

^{1}H NMR (500 MHz, CD_3CN): δ 7.71 (2H, d, J = 8.3 Hz, H^{17}), 7.40 (2H, d, J = 8.0 Hz, H^{18}), 7.21 (2H, s, H^{13}), 7.04 (4H, s, H^{3+4}), 6.28 (1H, d, J = 33.9 Hz), 4.20 (2H, s, H^6), 4.09 (2H, d, J = 11.6 Hz, H^7), 2.49 (6H, s, H^{12}), 2.44 (3H, s, H^{20}), 2.37 (3H, s, H^{15}), 2.27 (3H, s, H^1).

^{13}C NMR (126 MHz, CD_3CN): δ 168.6 (1C, d, J = 279.1 Hz, C^8), 145.9 (1C, s, C^{14}), 145.6 (1C, s, C^{19}), 143.5 (1C, s, C^{11}), 139.1 (1C, s, C^2), 136.4, 133.1, 131.2 (2C, s, C^{18}), 131.0 (2C, s, C^{13}), 130.2 (1C, s, C^{3or4}), 129.6 (2C, s, C^{3or4}), 128.3 (2C, s, C^{17}), 120.0 (1C, s, C^{10}), 77.6 (1C, d, J = 19.7 Hz, C^9), 53.4 (1C, s, C^6), 48.1 (1C, d, J = 30.6 Hz, C^7), 27.2 (2C, s, C^{12}), 21.6 (1C, s, C^{20}), 21.2 (1C, s, C^1), 21.1 (1C, s, C^{15}).

^{19}F NMR (376 MHz, CD_3CN): δ -74.33 (1F, dt, J = 33.9, 11.5 Hz, F^8), -151.78 + -151.83 (BF_4).

HRMS (ESI) calc: $[M – BF_4]^+$ ($C_{27}H_{30}FINO_2S$) 578.1021; measured: 578.1018 = 0.5 ppm difference.

IR (neat) v_{max}/cm^{-1}: 2973, 1647, 1451, 1331, 1158, 1055, 905, 866, 817, 763, 661, 580, 518, 545, 518.

Melting point: 129 - 133°C.

(Z)-(2-fluoro-3-((N-(4-fluorophenyl)-4-methylphenyl)sulfonamido)prop-1-en-1-yl)(mesityl)iodonium BF_4 ***(3ae)***

Synthesised according to conditions A, using N-(4-fluorophenyl)-4-methyl-N-(prop-2-yn-1yl)benzenesulfonamide (379 mg, 1.25 mmol, 1 equiv.), Ag_2CO_3 (35.4 mg, 0.125 mmol, 0.1 equiv.), 2-iodomesitylene (338 mg, 1.375 mmol, 1.1 equiv.), Selectfluor (664 mg, 1.875 mmol, 1.5 equiv.), and pyridine.9HF (1.18 mL), in $MeNO_2$ (8.8 mL). Trituration with Et_2O (3 x 15 mL) yielded a brown oil (705 mg, 74% yield based on a purity of 86% determined by quantitative ^{19}F NMR assay). The compound was spectroscopically pure by ^1H, ^{13}C, and ^{19}F NMR.

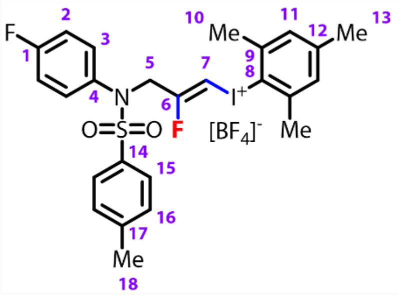

^1H NMR (500 MHz, $C(CD_3)_2O$): δ 7.47 (2H, d, *J* = 8.3 Hz, H^{16}), 7.38 (2H, d, *J* = Hz, H^{15}), 7.23 (2H, s, H^{11}), 7.08 (1H, d, *J* = 34.4 Hz, H^7), 7.05 – 6.98 (4H, m, H^{2+3}), 4.83 (2H, d, *J* = 14.1 Hz, H^5), 2.58 (6H, s, H^{10}), 2.42 (3H, s, H^{18}), 2.39 (3H, s, H^{13}).

^{13}C NMR (126 MHz, $C(CD_3)_2O$): δ 167.0 (1C, d, *J* = 278.9 Hz, C^6), 162.9 (1C, d, *J* = 247.0 Hz, C^1),145.4 (1C, s, C^{17}), 145.2 (1C, s, C^{12}), 143.1 (2C, s, C^9), 135.5 (1C, s, C^{14}), 135.2 (1C, d, *J* = 3.0 Hz, C^4), 131.6 (2C, d, *J* = 9.1 Hz, C^3), 131.0 (2C, s, C^{11}), 130.7 (2C, s, C^{15}), 128.6 (2C, s, C^{16}), 120.9 (1C, s, C^8), 116.7 (1C, d, *J* = 23.1 Hz, C^2), 80.1 (1C, d, *J* = 20.5 Hz, C^7), 51.3 (1C, d, *J* = 28.6 Hz, C^5), 26.9 (1C, s), 21.5 (2C, s, C^{10}), 21.0 (1C, s, C^{13}).

^{19}F NMR (376 MHz, $C(CD_3)_2O$): δ -77.29 (1F, dt, *J* = 34.3, 14.1 Hz, F^6), -113.72 – 113.79 (1F, tt, *J* = 7.9, 5.3 Hz, F^1), -151.05 – -151.11 (*BF_4*).

HRMS (ESI) calc: $[M – BF_4]^+$ ($C_{25}H_{25}NO_2SF_2I$) 568.0619; measured: 568.0613 = 1.1 ppm difference.

IR (neat) v_{max}**/cm^{-1}:** 3085, 1654, 1598, 1556, 1505, 1449, 1403, 1379, 1343, 1303, 1211, 1158, 1052, 1012, 942, 884, 847, 819, 770, 733, 707, 668, 618, 586, 552, 520, 494, 481.

Melting point: 143 – 148 °C.

(Z)-(3-((N-(4-bromophenyl)-4-methylphenyl)sulfonamido)-2-fluoroprop-1-en-1-yl)(mesityl)iodonium *BF_4* ***(3af)***

Synthesised according to conditions B, using N-(4-bromophenyl)-4-methyl-N-(prop-2-yn-1yl)benzenesulfonamide (455 mg, 1.25 mmol, 1 equiv.), Ag_2CO_3 (35.4 mg, 0.125 mmol, 0.1 equiv.), 2-iodomesitylene (338 mg, 1.375 mmol, 1.1 equiv.), Selectfluor (664 mg, 1.875 mmol, 1.5 equiv.), K_2CO_3 (518 mg, 3.75 mmol, 3 equiv.), and pyridine.9HF (1.18 mL), in $MeNO_2$ (8.8 mL). Trituration with Et_2O (3 x 15 mL) and evaporation of the residual solvent under reduced pressure yielded a yellow solid (894 mg, 87% yield based on a purity of 87% determined by quantitative ^{19}F NMR assay). The compound was spectroscopically pure by ^{1}H, ^{13}C, and ^{19}F NMR.

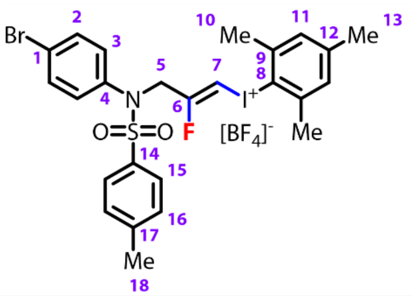

^{1}H NMR (600 MHz, $C(CD_3)_2O$): δ 7.50 – 7.47 (2H, m, H^{15}), 7.42 – 7.39 (2H, m, J = 8.7 Hz, H^{2}), 7.39 – 7.36 (2H, m, H^{16}), 7.24 – 7.22 (2H, m, H^{11}), 7.06 (1H, dt, J = 34.3, 0.8 Hz, H^{7}), 6.97 – 6.94 (2H, m, H^{3}), 4.84 (2H, dd, J = 14.6, 0.8 Hz,), 2.55 (6H, s, H^{10}), 2.42 (3H, s, H^{18}), 2.41 (3H, s, H^{4}).

^{13}C NMR (151 MHz, $C(CD_3)_2O$): δ 166.9 (1C, d, J = 277.6 Hz, C^{6}), 145.5 (1C, s, C^{17}), 145.3 (1C, s, C^{12}), 143.1 (2C, s, C^{9}), 138.4 (1C, s, C^{4}), 135.4 (1C, s, C^{14}), 133.1 (2C, s, C^{2}), 131.2 (2C, s, C^{3}), 131.0 (2C, s, C^{11}), 130.7 (2C, s, C^{16}), 128.6 (2C, s, C^{15}), 122.8 (1C, s, C^{1}), 120.7 (1C, s, C^{8}), 80.0 (1C, d, J = 19.1 Hz, C^{7}), 51.0 (1C, d, J = 28.7 Hz, C^{5}), 26.9 (1C, d, J = 1.3 Hz, C^{18}), 21.3 (1C, d, J = 52.8 Hz, C^{13}).

^{19}F NMR (376 MHz, $C(CD_3)_2O$): δ -77.16 (1F, dt, J = 33.7, 13.3 Hz, F^{6}), -150.69 – -151.17 (BF_4).

HRMS (ESI) calc: $[M – BF_4]^+$ ($C_{25}H_{25}NO_2SFBrI$) 627.9818; measured: 627.9821 = 0.5 ppm difference.

IR (neat) v_{max}**/cm^{-1}:** 3090, 2980, 2924, 1657, 1484, 1348, 1162, 1054, 876, 815, 751, 712, 663, 591, 544.

Melting point: 146 – 152 °C.

(Z)-(2-fluoro-2-(4-fluorophenyl)vinyl)(mesityl)iodonium BF_4 ***(3ag)***

Synthesised according to the general synthetic procedure using 4-fluoro phenylacetylene (725 μL, 5 mmol, 1 equiv.), Ag_2CO_3 (138 mg, 0.5 mmol, 1 equiv.), 2-iodomesitylene (1.35 g, 5.5 mmol, 1.1 equiv.), Selectfluor (4.43 g, 12.5 mmol, 2.5 equiv.) and pyridine.9HF (4.71 mL, 180 mmol, 36 equiv. HF), in $MeNO_2$ (35.3 mL). Trituration with Et_2O (3 x 50 mL) and evaporation of the residual solvent under reduced pressure yielded a white solid (1.91 g, 71%, based on a purity of 88% determined by quantitative ^{19}F NMR assay). The compound was spectroscopically pure by ^{1}H, ^{13}C, and ^{19}F NMR.

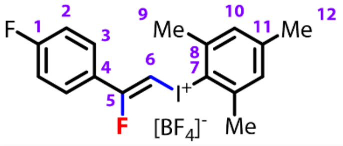

^{1}H NMR (500 MHz, CD_3CN): δ 7.75 – 7.69 (2H, m, H^3), 7.26 (2H, dd, J = 8.7 Hz, H^2), 7.23 (2H, s, H^{10}), 7.00 (1H, d, J = 35.5 Hz, H^6), 2.66 (6H, s, H^9), 2.35 (3H, s, H^{12}).

^{13}C NMR (126 MHz, CD_3CN): δ 167.3 (d, J = 265.1 Hz, C^5), 166.2 (d, J = 252.9 Hz, C^1), 145.9 (1C, s, C^{11}), 143.6 (2C, s, C^8), 131.3 (2C, s, C^{10}), 130.2 (2C, dd, J = 9.4, 7.1 Hz, C^3), 124.8 (1C, d, J = 27.9 Hz, C^4), 120.6 (1C, s, C^7), 117.4 (2C, dd, J = 22.9, 1.8 Hz, C^2), 74.3 (1C, d, J = 24.2 Hz, C^6), 27.3 (2C, s, C^9), 21.0 (1C, s, C^{12}).

^{19}F NMR (376 MHz, CD_3CN): δ -79.12 (1F, d, J = 35.4 Hz, F^5), -106.92 – -107.07 (1F, m, F^1), -151.52 + -151.71 (BF_4).

HRMS (ESI) calc: $[M – BF_4]^+$ ($C_{17}H_{16}F_2I$) 385.0259; measured: 367.0272 = 3.4 ppm difference.

IR (neat) v_{max}**/cm^{-1}:** 3668, 3099, 2984, 2901, 1627, 1602, 1509, 1239, 1049, 847.

Melting point: 207 - 210°C

(Z)-(2-(4-bromophenyl)-2-fluorovinyl)(mesityl)iodonium BF_4 ***(3ah)***

Synthesised according to conditions B using 1-bromo-4-ethynylbenzene (226 mg, 1.25 mmol, 1 equiv.), Ag_2CO_3 (35.4 mg, 0.125 mmol, 0.1 equiv.), 2-iodomesitylene (338 mg, 1.375 mmol, 1.1 equiv.), Selectfluor (664 mg, 1.875 mmol, 1.5 equiv.), K_2CO_3 (518 mg, 3.75 mmol, 3 equiv.), and pyridine.9HF (1.18 mL), in $MeNO_2$ (8.8 mL). Trituration with Et_2O (3 x 15 mL) and evaporation of the residual solvent under reduced pressure yielded a light-yellow solid (412 mg, 56% based on a purity of 91% determined by quantitative ^{19}F NMR assay). The compound was spectroscopically pure by ^{1}H, ^{13}C, and ^{19}F NMR.

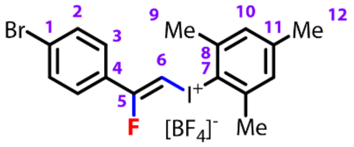

^{1}H NMR (500 MHz, $C(CD_3)_2O$): δ 7.84 (1H, d, *J* = 36.0 Hz, H^{6}), 7.76 (4H, s, H^{2+3}), 7.30 (2H, m, H^{10}), 2.78 (6H, s, H^{9}), 2.37 (3H, s, H^{12}).

^{13}C NMR (126 MHz, $C(CD_3)_2O$): δ 166.9 (1C, d, *J* = 263.9 Hz, C^{5}), 145.4 (1C, s, C^{11}), 143.5 (2C, s, C^{8}),133.3 (1C, d, *J* = 1.9 Hz, C^{2}), 131.1 (2C, s, C^{10}), 129.0 (2C, d, *J* = 7.0 Hz, C^{3}), 127.8 (1C, s, C^{1}), 127.6 (1C, d, *J* = 29.1 Hz, C^{4}), 120.8 (1C, s, C^{7}), 76.0 (1C, d, *J* = 22.9 Hz, C^{6}), 27.1 (2C, d, *J* = 1.2 Hz, C^{9}), 20.9 (1C, s, C^{12}).

^{19}F NMR (376 MHz, $C(CD_3)_2O$): δ -80.46 (1F, d, *J* = 35.3 Hz F^{5}), -151.59 + -151.64 (BF_4).

HRMS (ESI) calc: $[M – BF_4]^+$ ($C_{17}H_{16}FBrI$) 446.9434; measured: 446.9438 = 0.89 ppm difference.

IR (neat) v_{max}/cm^{-1}: 3105, 1616, 1586, 1488, 1454, 1400, 1285, 1027, 1005, 942, 841, 820, 803, 748, 626, 540, 521, 462.

Melting point: 182 – 192 °C.

(Z)-(2-fluoro-2-(4-(trifluoromethyl)phenyl)vinyl)(mesityl)iodonium BF_4 **(3ai)**

Synthesised according to the general synthetic procedure using 1-ethynyl-4-(trifluoromethyl)benzene (179 µL, 1.25 mmol, 1 equiv.), Ag_2CO_3 (35.4 mg, 0.125 mmol, 0.1 equiv.), 2-iodomesitylene (338 mg, 1.375 mmol, 1.1 equiv.), Selectfluor (664 mg, 1.875 mmol, 1.5 equiv.), K_2CO_3 (518 mg, 3.75 mmol, 3 equiv.), and pyridine.9HF (1.18 mL), in $MeNO_2$ (8.8 mL). Trituration with Et_2O (3 x 15 mL) and evaporation of the residual solvent under reduced pressure yielded a light-yellow solid (558 mg 74%, based on a purity of 86% determined by quantitative ^{19}F NMR assay). The compound was spectroscopically pure by ^{1}H, ^{13}C, and ^{19}F NMR.

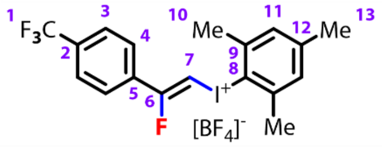

^{1}H NMR (500 MHz, $C(CD_3)_2O$): δ 8.04 (2H, d, *J* = 8.4 Hz, H^3), 7.96 (1H, d, *J* = 35.9 Hz, H^7), 7.90 (2H, d, *J* = 8.0 Hz H^4), 7.31 (2H, s, H^{11}), 2.80 (6H, s, H^{10}), 2.37 (3H, s, C^{13}).

^{13}C NMR (126 MHz, $C(CD_3)_2O$): δ 166.1 (1C, d, *J* = 264.4 Hz, C^6), 145.5 (1C, s, C^{12}), 143.6 (2C, s, C^9), 134.0 (1C, q, *J* = 32.7 Hz, C^2), 132.2 (1C, q, *J* = 28.6 Hz), 131.1(2C, s, C^{11}), 128.0 (2C, d, *J* = 7.0 Hz, C^3),127.1 (2C, dd, *J* = 4.0, 2.2 Hz, C^4), 124.6 (1C, q, *J* = 271.9 Hz, C^1), 120.7 (1C, s, C^8), 78.1 (1C, d, *J* = 22.6 Hz, C^7), 27.1 (2C, d, *J* = 1.4 Hz, C^{10}), 20.9 (1C, s, C^{13}).

^{19}F NMR (376 MHz, $C(CD_3)_2O$): δ -63.77 (3F, s, F^1), -82.05 (1F, d, *J* = 35.8 Hz, F^6), -150.84 + - 150.90 (BF_4).

HRMS (ESI) calc: $[M – BF_4]^+$ ($C_{18}H_{16}F_4I$) 435.0227; measured: 435.0227 = 0 ppm difference.

IR (neat) v_{max}**/cm^{-1}:** 3100, 1627, 1576, 1454, 1412, 1384, 1302, 1323, 1290, 1185, 1118, 1068, 1013, 943, 850, 816, 758, 737, 631, 521.

Melting point: 175 – 181 °C.

(Z)-(2-fluoro-2-(4-nitrophenyl)vinyl)(mesityl)iodonium BF_4 ***(3aj)***

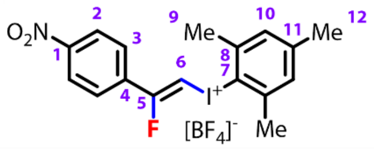

Synthesised according to the general synthetic procedure using 1-ethynyl-4-nitrobenzene (223 mg, 1.25 mmol, 1 equiv.), Ag_2CO_3 (35.4 mg, 0.125 mmol, 0.1 equiv.), 2-iodomesitylene (338 mg, 1.375 mmol, 1.1 equiv.), Selectfluor (664 mg, 1.875 mmol, 1.5 equiv.), K_2CO_3 (518 mg, 3.75 mmol, 3 equiv.), and pyridine.9HF (1.18 mL), in $MeNO_2$ (8.8 mL). Trituration with Et_2O (3 x 15 mL) and evaporation of the residual solvent under reduced pressure yielded a yellow solid (518 mg, 63% yield, based on a purity of 76%, as determined by quantitative ^{19}F NMR assay). The compound was spectroscopically pure by ^{1}H, ^{13}C, and ^{19}F NMR.

^{1}H NMR (500 MHz, $C(CD_3)_2O$): δ 8.39 – 8.34 (2H, m, H^2), 8.12 – 8.08 (2H, m, H^3), 8.04 (1H, d, J = 35.8 Hz, H^6), 7.30 (2H, s, H^{10}), 2.80 (6H, s, H^9), 2.37 (3H, s, H^{12}).

^{13}C NMR (126 MHz, $C(CD_3)_2O$): δ 165.6 (1C, d, J = 264.5 Hz, C^5), 150.9 (1C, s, C^1), 145.6 (1C, s, C^{11}), 143.6 (2C, s, C^8), 134.2 (d, J = 28.6 Hz, C^4), 131.1 (2C, s, C^{10}), 128.6 (2C, d, J = 6.9 Hz, C^3), 125.1 (2C, d, J = 1.9 Hz, C^2), 120.8 (1C, s, C^7), 79.4 (1C, d, J = 22.6 Hz, C^6), 27.2 (2C, s, C^9), 21.0 (1C, s, C^{12}).

^{19}F NMR (376 MHz, $C(CD_3)_2O$): δ -82.08 (1F, d, J = 35.9 Hz, F^5), -150.69 + -150.75 (BF_4).

HRMS (ESI) calc: $[M – BF_4]^+$ ($C_{17}H_{16}FINO_2$) 412.0204; measured: 412.0196 = 1.94 ppm difference.

IR (neat) v_{max}/cm^{-1}: 3111, 1630, 1594, 1525, 1450, 1346, 1296, 1000, 856, 940, 856, 796, 742, 742, 691, 636, 620, 518.

(Z)-(2-fluoro-2-(4-methoxyphenyl)vinyl)(mesityl)iodonium BF_4 ***(3ak)***

Synthesised according to conditions B using 1-ethynyl-4-methoxybenzene (165 μL, 1.25 mmol, 1 equiv.), Ag_2CO_3 (35.4 mg, 0.125 mmol, 0.1 equiv.), 2-iodomesitylene (338 mg, 1.375 mmol, 1.1 equiv.), Selectfluor (664 mg, 1.875 mmol, 1.5 equiv.), K_2CO_3 (518 mg, 3.75 mmol, 3 equiv.), and pyridine.9HF (1.18 mL), in $MeNO_2$ (8.82 mL). Trituration with Et_2O (3 x 15 mL) and evaporation of the residual solvent under reduced pressure yielded a white solid (434 mg, 50% based on a purity of 70% determined by quantitative ^{19}F NMR assay). The compound was spectroscopically pure by ^{1}H, ^{13}C, and ^{19}F NMR.

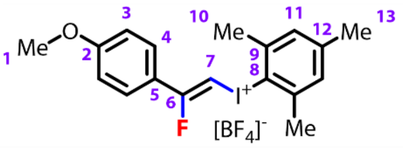

^{1}H NMR (500 MHz, $C(CD_3)_2O$): δ 7.80 – 7.72 (2H, m, *H*^{4}), 7.53 (1H, d, *J* = 36.2 Hz, *H*^{7}), 7.28 (2H, s, *H*^{11}), 7.10 – 7.06 (2H, m, *H*^{3}), 3.88 (3H, s, *H*^{1}), 2.78 (6H, s, *H*^{10}), 2.36 (3H, s, *H*$^{\mathbf{13}}$).

^{13}C NMR (126 MHz, $C(CD_3)_2O$): δ 167.2 (1C, d, *J* = 263.1 Hz, *C*^{6}), 164.3 (1C, s, *C*^{2}), 145.2, 143.3, 131.0, 129.2 (1C, d, *J* = 7.5 Hz,), 121.1, 120.6, 120.4, 115.6 (2C, s, *C*^{3}), 72.0 (1C, dd, *J* = 23.6, 2.0 Hz, *C*^{7}), 56.1 (1C, d, *J* = 5.3 Hz, *C*^{1}), 27.1 (2C, d, *J* = 4.0 Hz, *C*^{10}), 20.9 (d, *J* = 2.5 Hz, *C*^{13}).

^{19}F NMR (377 MHz, $C(CD_3)_2O$): δ 80.59 (d, *J* = 36.1 Hz), -150.99 + -151.05 (*BF_4*). A minor peak was observed at -63.47 (d, *J* = 14.3 Hz), which was assigned as the *(E)*-isomer.

HRMS (ESI) calc: $[M – BF_4]^+$ ($C_{18}H_{19}FIO$) 397.0459; measured: 397.0457 = 0.50 ppm difference.

IR (neat) v_{max}/cm^{-1}: 3101, 1602, 1570, 1512, 1456, 1382, 1290, 1261, 1181, 1012, 943, 839, 757, 739, 686, 637, 576, 561, 520.

(Z)-(2-(4-acetylphenyl)-2-fluorovinyl)(mesityl)iodonium BF_4 ***(3al)***

Synthesised according to conditions B using 1-(4-ethynylphenyl)ethan-1-one (180 mg, 1.25 mmol, 1 equiv.), Ag_2CO_3 (35.4 mg, 0.125 mmol, 0.1 equiv.), 2-iodomesitylene (338 mg, 1.375 mmol, 1.1 equiv.), Selectfluor (664 mg, 1.875 mmol, 1.5 equiv.), K_2CO_3 (518 mg, 3.75 mmol, 3 equiv.), and pyridine.9HF (1.178 mL), in $MeNO_2$ (8.82 mL). Trituration with Et_2O (3 x 15 mL) and evaporation of the residual solvent under reduced pressure yielded a yellow solid (149 mg, 20%, based on a purity of 83% determined by quantitative ^{19}F NMR assay). The compound was spectroscopically pure by ^{1}H, ^{13}C, and ^{19}F NMR.

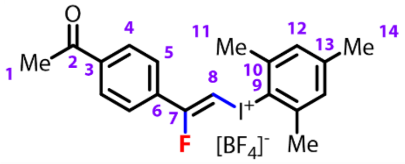

^{1}H NMR (500 MHz, $C(CD_3)_2O$): δ 8.11 (2H, d, *J* = 8.0 Hz, *C*^{4}), 7.94 (2H, d, *J* = 8.6 Hz, *H*^{5}), 7.90 (1H, d, *J* = 35.9 Hz, *H*^{8}), 7.32 – 7.27 (2H, m, *H*^{12}), 2.80 (6H, s, *H*^{11}), 2.62 (3H, s, *H*^{1}), 2.37 (3H, s, *H*^{14}).

^{13}C NMR (126 MHz, $C(CD_3)_2O$): δ 197.3 (1C, s, *C*^{2}), 166.8 (1C, d, *J* = 264.5 Hz, *C*^{7}), 145.5 (1C, s, *C*^{13}), 143.5 (2C, s, *C*^{10}), 140.9 (1C, s, *C*^{3}), 132.2 (1C, d, *J* = 27.8 Hz, *C*^{6}), 131.1 (2C, s, *C*^{12}), 129.7 (2C, d, *J* = 1.9 Hz, *C*^{4}), 127.5 (2C, d, *J* = 6.9 Hz, *C*^{5}), 120.8 (1C, s, *C*^{9}), 77.6 (1C, d, *J* = 22.9 Hz, *C*^{8}), 27.1 (2C, d, *J* = 1.2 Hz, *C*^{11}), 26.9 (1C, s, *C*^{1}), 20.9 (1C, s, *C*^{14}).

^{19}F NMR (376 MHz, $C(CD_3)_2O$): δ -81.90 (1F, d, *J* = 36.0 Hz, *F*^{7}), -150.77 + -150.82 (*BF_4*).

HRMS (ESI) calc: $[M – BF_4]^+$ ($C_{19}H_{19}FIO$) 409.0459; measured: 409.0465 = 1.46 ppm difference.

IR (neat) v_{max}/cm^{-1}: 3104, 1670, 1619, 1410, 1360, 1267, 1023, 956, 847, 838, 813, 754, 727, 685, 635, 594, 522, 480.

(Z)-(2-fluoro-2-(4-formylphenyl)vinyl)(mesityl)iodonium BF_4 ***(3am)***

Synthesised according to conditions B using (163 mg, 1.25 mmol, 1 equiv.), Ag_2CO_3 (35.4 mg, 0.125 mmol, 0.1 equiv.), 2-iodomesitylene (338 mg, 1.375 mmol, 1.1 equiv.), Selectfluor (664 mg, 1.875 mmol, 1.5 equiv.), K_2CO_3 (518 mg, 3.75 mmol, 3 equiv.), and pyridine.9HF (1.18 mL), in $MeNO_2$ (8.8 mL). Trituration with Et_2O (3 x 15 mL) and evaporation of the residual solvent under reduced pressure yielded a yellow solid (282 mg, 39% yield based on a purity of 83% determined by quantitative ^{19}F NMR assay). The compound was spectroscopically pure by ^1H, ^{13}C, and ^{19}F NMR.

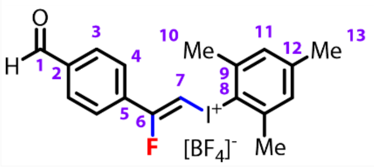

^1H NMR (500 MHz, $C(CD_3)_2O$): δ 10.12 (1H, s, H^1), 8.04 (4H, q, J = 8.5 Hz, H^{3+4}), 7.95 (1H, d, J = 35.9 Hz, H^7), 7.31 – 7.29 (2H, dd, J = 1.4, 0.8 Hz, H^{11}), 2.80 (6H, s, H^{10}), 2.37 (3H, s, H^{13}).

^{13}C NMR (126 MHz, $C(CD_3)_2O$): δ 192.3 (1C, s, C^1), 166.6 (d, J = 264.6 Hz, C^6), 145.5 (1C, s, C^{12}), 143.6 (2C, s, C^9), 140.0 (1C, s, C^2), 133.2 (1C, d, J = 27.8 Hz, C^5), 131.1 (2C, s, C^{11}), 130.8 (2C, d, J = 2.1 Hz, C^3), 127.9 (2C, d, J = 7.0 Hz, C^4), 120.8 (1C, s, C^8), 78.2 (d, J = 22.9 Hz), 27.2 (d, J = 1.2 Hz, C^{10}), 21.0 (3H, s, C^{13}).

^{19}F NMR (376 MHz, $C(CD_3)_2O$): δ -81.81 (1F, d, J = 35.9 Hz, F^6), -150.92 + -150.97 (BF_4). A minor peak was observed at -64.42 (d, J = 15.2 Hz), which was assigned as the *(E)*-isomer.

HRMS (ESI) calc: $[M – BF_4]^+$ ($C_{18}H_{17}FIO$) 395.0303; measured: 393.0313 = 2.53 ppm difference.

IR (neat) v_{max}/cm^{-1}: 3098, 1703, 1622, 1568, 1452, 1384, 1285, 1213, 1175, 1027, 838, 778, 628, 520.

Melting point: 167 – 170 °C.

(Z)-(2-fluoro-2-(pyridin-2-yl)vinyl)(mesityl)iodonium BF_4 ***(3an)***

Synthesised according to conditions A using 2-ethynylpyridine (500 µL, 5 mmol, 1 equiv.), Ag_2CO_3 (136 mg, 0.5 mmol, 0.1 equiv.), 2-iodomesitylene (1.35 g, 5.5 mmol, 1.1 equiv.), Selectfluor (2.66 g, 7.25 mmol, 1.5 equiv.), and pyridine.9HF (4.7 mL), in $MeNO_2$ (35.3 mL). Trituration with Et_2O (2 x 50 mL) and evaporation of the residual solvent under reduced pressure yielded a brown solid (2.12 g, 77% yield based on a purity of 83%, as determined by quantitative ^{19}F NMR assay). The compound was spectroscopically pure by ^{1}H, ^{13}C, and ^{19}F NMR.

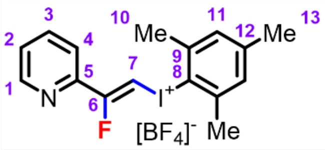

^{1}H NMR (500 MHz, CD_3CN): δ 8.64 (1H, d, *J* = 3.0 Hz, H^1), 7.93 (1H, td, *J* = 8.2, 7.8, 1.8 Hz, H^3), 7.71 (1H, d, *J* = 7.6 Hz, H^4) , 7.54 – 7.49 (1H, m, H^2), 7.44 (1H, d, *J* = 34.2 Hz, H^7), 7.23 (2H, s, H^{11}), 2.66 (6H, s, H^{10}), 2.35 (3H, s, H^{13}).

^{13}C NMR (126 MHz, CD_3CN): δ 166.8 (1C, d, *J* = 265.7 Hz, C^6), 151.5 (1C, d, *J* = 4.7 Hz, C^1), 146.9 (d, *J* = 34.5 Hz, C^5), 146.0 (1C, s, C^{12}), 143.7 (2C, s, C^9), 138.8 (1C, s, C^3), 131.3 (2C, s, C^{11}), 128.0 (1C, s, C^2), 122.0 (1C, d, *J* = 3.4 Hz, C^4), 120.4 (1C, s, C^8), 78.1 (d, *J* = 20.4 Hz, C^7), 27.3 (2C, s, C^{10}), 21.0 (1C, s, C^{13}).

^{19}F NMR (376 MHz, $CDCl_3$): δ -88.81 (1F, d, *J* = 34.6 Hz, F^6), -149.33 + -149.38 (BF_4).

HRMS (ESI) calc: $[M – BF_4]^+$ ($C_{16}H_{16}FIN$) 368.0306; measured: 368.0299 = 1.90 ppm difference.

IR (neat) v_{max}**/cm^{-1}:** 3098, 2992, 2925, 1634, 1578, 1464, 1381, 1316, 1303, 1019, 796, 763, 627, 607, 521.

(Z)-(2-fluoro-2-(quinolin-3-yl)vinyl)(mesityl)iodonium ***(3ao)***

The Z-FVI formation step was conducted by employing conditions B, using 3-ethynylquinoline (191 mg, 1.25 mmol, 1 equiv.), Ag_2CO_3 (35.4 mg, 0.125 mmol, 0.1 equiv.), 2-iodomesitylene (338 mg, 1.375 mmol, 1.1 equiv.), Selectfluor (664 mg, 1.875 mmol, 1.5 equiv.), K_2CO_3 (518 mg, 3.75 mmol, 3 equiv.), and pyridine.9HF (1.18 mL), in $MeNO_2$ (8.82 mL), affording the compound in 49% NMR yield after work-up. The ^{19}F NMR and HRMS data of the crude mixture was in alignment with *Z*-FVI formation.

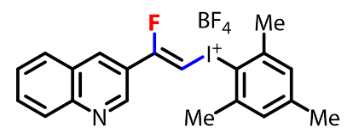

^{19}F NMR (376 MHz, CH_3CN): -81.00 (1F, d, *J* = 35.9 Hz)

HRMS (ESI) calc: $[M – BF_4]^+$ ($C_{20}H_{18}NFI$) 418.0468 ; measured: 418.0464 = 1.0 ppm difference

(Z)-3-(1-fluoro-2-iodovinyl)quinoline: The crude product was dissolved in MeCN (10 mL), transferred to a glass vial with a stirrer bar, and TEAI (354 mg, 1.38 mmol, 1.1 equiv. w.r.t the alkyne from the first step) was added. The mixture was stirred at 60 °C for 18 hours, and the solvent was subsequently evaporated under reduced pressure. The resulting crude mixture was subjected to flash-column chromatography (0 to 35% Et_2O in pentane) to afford the product as a white solid (153 mg, 41% over two steps).

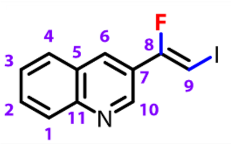

^1H NMR (500 MHz, $CDCl_3$): δ 9.02 (s, 1H), 8.26 (d, *J* = 2.3 Hz, 2H), 8.10 (d, *J* = 8.4 Hz, 2H), 7.86 (dd, *J* = 8.1, 1.4 Hz, 2H), 7.76 (ddd, *J* = 8.4, 6.9, 1.4 Hz, 3H), 7.59 (ddd, *J* = 8.2, 6.9, 1.2 Hz, 3H), 6.39 (d, *J* = 34.7 Hz, 2H).

^{13}C NMR (126 MHz, $CDCl_3$): δ 161.1 (1C, d, *J* = 251.9 Hz, C^8), 148.1 (1C, s, C^{11}), 146.7 (d, *J* = 5.9 Hz, C^{10}), 131.4 (d, *J* = 5.3 Hz), 130.8(1C, s, C^2),, 129.6 (1C, s, C^1),, 128.6 (1C, s, C^4),, 127.9 (1C, s, C^3), 127.1 (1C, s, C^5), 124.1 (d, *J* = 28.7 Hz, C^7), 55.9 (d, *J* = 27.9 Hz, C^9).

^{19}F (376 MHz, $CDCl_3$): δ -91.14 (1F, d, *J* = 34.7 Hz).

HRMS (ESI) calc: $[M+H]^+$ ($C_{11}H_7FIN$) 299.9680; measured: 299.9673 = 2.33 ppm difference.

IR (neat) v_{max}/cm^{-1}: 3087, 3026, 2927, 2857, 1655, 1603, 1496, 1453, 1427, 1247, 1114m 1080, 1030, 976, 883, 847, 804, 744, 698, 584, 563, 509, 485

(Z)-(2-fluoro-2-(thiophen-3-yl)vinyl)(mesityl)iodonium BF_4 ***(3ap)***

Synthesised according to conditions B, using 3-ethynylthiophene (270 mg, 1.25 mmol, 1 equiv.), Ag_2CO_3 (35.4 mg, 0.125 mmol, 0.1 equiv.), 2-iodomesitylene (338 mg, 1.375 mmol, 1.1 equiv.), Selectfluor (664 mg, 1.875 mmol, 1.5 equiv.), K_2CO_3 (518 mg, 3.75 mmol, 3 equiv.), and pyridine.9HF (1.18 mL), in $MeNO_2$ (8.8 mL). Trituration with Et_2O (3 x 15 mL) and evaporation of the residual solvent under reduced pressure yielded a brown solid (465 mg, 70% based on a purity of 87% determined by quantitative ^{19}F NMR assay). The compound was spectroscopically pure by ^{1}H, ^{13}C, and ^{19}F NMR.

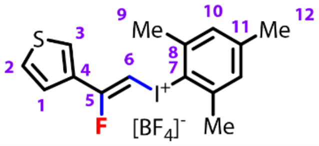

^{1}H NMR (500 MHz, $C(CD_3)_2O$): δ 8.14 (1H, dd, *J* = 3.0, 1.3 Hz, H^1), 7.70 (1H, dt, *J*= 5.2, 2.9 Hz, H^2), 7.58 (1H, d, *J* = 36.0 Hz, H^6), 7.52 (1H, dt, *J* = 5.2, 1.2 Hz, H^3), 7.28 (2H, dd, *J* = 1.4, 0.8 Hz H^{10}), 2.77 (6H, s, H^9), 2.36 (3H, s, H^{12}).

^{13}C NMR (126 MHz, $C(CD_3)_2O$): δ 164.2 (1C, d, *J* = 261.8 Hz, C^5), 145.3 (1C, s, C^{11}), 143.4 (2C, s, C^8), 131.0 (2C, s, C^{10}), 130.1 (d, *J* = 29.8 Hz, C^4), 129.9 (d, *J* = 4.7 Hz, C^1), 129.7 (d, *J* = 1.9 Hz, C^2), 125.9 (d, *J* = 6.6 Hz, C^3), 121.0 (1C, s, C^7), 73.8 (d, *J* = 22.1 Hz, C^6), 27.1 (2C, d, *J* = 1.3 Hz, C^9), 20.9 (1C, s, C^{12}).

^{19}F NMR (376 MHz, $C(CD_3)_2O$): δ -79.37 (1F, d, *J* = 35.9, F^5), -150.71 (BF_4). A minor peak was observed at -65.70 (d, *J* = 15.2 Hz), which was assigned as the *(E)*-isomer.

HRMS (ESI) calc: $[M – BF_4]^+$ ($C_{15}H_{15}FIS$) 372.9918; measured: 372.9920 = 0.80 ppm difference.

IR (neat) v_{max}/cm^{-1}: 3094, 1622, 1452, 1283, 1037, 943, 914, 876, 854, 802, 730, 672, 541, 521.

Melting point: 173 – 176 °C

(Z)-(2-fluoro-3-oxobut-1-en-1-yl)(mesityl)iodonium BF_4 ***(3aq)***

Synthesised according to conditions A using butynone (340 mg, 2.5 mmol, 1 equiv.), Ag_2CO_3 (689 mg, 2.5 mmol, 1 equiv.), 2-iodomesitylene (677 mg, 2.75 mmol, 1.1 equiv.), Selectfluor (1.33 g, 3.75 mmol, 1.5 equiv.), and pyridine.9HF (2.33 mL), in $MeNO_2$ (17.7 mL). Trituration with pentane (3 x 30 mL) and evaporation of the residual solvent under reduced pressure yielded (671 mg, 58% based on a purity of 90% determined by quantitative ^{19}F NMR assay). The compound was spectroscopically pure by ^{1}H, ^{13}C, and ^{19}F NMR.

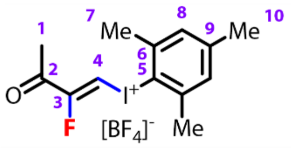

^{1}H NMR (500 MHz, CD_3CN): 7.48 (1H, d, J = 33.0 Hz, H^4), 7.25 (2H, s, H^8), 2.62 (6H, s, H^7), 2.38 – 2.34f (6H, m, H^{1+10}).

^{13}C NMR (126 MHz, CD_3CN): δ 188.0 (1C, d, J = 29.0 Hz, C^2), 161.4 (1C, d, J = 282.3 Hz, C^3), 146.3 (1C, s, C^9), 144.0 (2C, s, C^8), 131.4 (2C, s, C^8), 119.7 (1C, s, C^5), 90.8 (1C, d, J = 19.8 Hz, C^4), 27.4 (2C, s, C^7), 26.6 (1C, s, C^1), 21.1 (1C s, C^{10}).

^{19}F NMR (376 MHz, CD_3CN): δ -90.88 (1F, dt, J = 33.1, 2.1 Hz, F^4), -151.47 + -151.53 (BF_4).

HRMS (ESI) calc: $[M – BF_4]^+$ ($C_{13}H_{15}FIO$) 333.0146; measured: 333.0162 = 4.6 ppm difference.

IR (neat) v_{max}**/cm^{-1}:** 3095.4, 2920.3, 1713.8, 1620.8, 1449.8, 1371.8, 1288.2, 1196.7, 1002.6, 974.8, 940.9, 848.7, 796.3, 763.7, 682.3, 605.9, 551.4, 537.9, 520.1, 468.8.

(Z)-(3-ethoxy-2-fluoro-3-oxoprop-1-en-1-yl)(mesityl)iodonium BF_4 ***(3ar)***

Synthesised according to conditions A but with stoichiometric Ag_2CO_3, using ethyl propiolate (253 μL, 2.5 mmol, 1 equiv.), Selectfluor (1.33 g, 7.5 mmol, 1.5 equiv.) and pyridine.9HF (2.33 mL), in $MeNO_2$ (17.7 mL). Trituration with pentane (2 x 30 mL) and evaporation of the residual solvent under reduced pressure yielded a light-yellow solid (703 mg, 54% yield based on a purity of 87% determined by quantitative ^{19}F NMR assay). The compound was spectroscopically pure by ^{1}H, ^{13}C, and ^{19}F NMR.

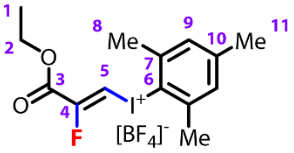

^{1}H NMR (500 MHz, CD_3CN): δ 7.50 (1H, d, J = 31.7 Hz, H^5), 7.25 (2H, s, H^9), 4.30 (2H, q, J = 7.1 Hz, H^2), 2.61 (6H, s, H^8), 2.36 (3H, s, H^{11}), 1.28 (3H, t, J = 7.1 Hz, H^1).

^{13}C NMR (126 MHz, CD_3CN): δ 157.0 (1C, d, J = 275.2 Hz, C^4), 156.9 (1C. d, J = 36.6 Hz, C^3), 146.3 (1C, s, C^{10}), 144.0 (1C, s, C^6), 131.4 (2C, s, C^9), 120.0 (1C, s, C^7), 89.7 (1C, d, J = 18.6 Hz, C^5), 64.7 (1C, s, C^2), 27.4 (2C, s, C^8), 21.1 (1C, s, C^{11}), 14.1 (1C, s, C^1).

^{19}F NMR (376 MHz, CD_3CN): δ -91.18 (1F, d, J = 31.9 Hz, F^4), -151.21 (BF_4).

HRMS (ESI) calc: $[M-BF_4]^+$ ($C_{14}H_{17}O_2FI$) 363.0252; measured: 363.0245 = 1.93 ppm difference.

IR (neat) v_{max}/cm^{-1}: 3535, 3098, 2982, 1738, 1638, 1588, 1451, 1373, 1316, 1302, 1220, 1031, 1008, 940, 864, 761, 734, 710, 685, 645, 520.

Melting point: 82 – 86 °C.

(Z)-(4-bromophenyl)(2-fluoro-5-phenylpent-1-en-1-yl)iodonium BF_4 ***(4a)***

Synthesised according to conditions B, using pent-4-yn-1-ylbenzene (270 mg, 1.25 mmol, 1 equiv.), Ag_2CO_3 (35.4 mg, 0.125 mmol, 0.1 equiv.), 1-bromo-4-iodobenzene (389 mg, 1.375 mmol, 1.1 equiv.), Selectfluor (664 mg, 1.875 mmol, 1.5 equiv.), K_2CO_3 (518 mg, 3.75 mmol, 3 equiv.), and pyridine.9HF (1.18 mL), in $MeNO_2$ (8.82 mL). Trituration with Et_2O (3 x 15 mL) and evaporation of the residual solvent under reduced pressure yielded a light-yellow oil (428 mg, 58% yield based on a purity of 91% determined by quantitative ^{19}F NMR assay). The compound was spectroscopically pure by ^{1}H, ^{13}C, and ^{19}F NMR.

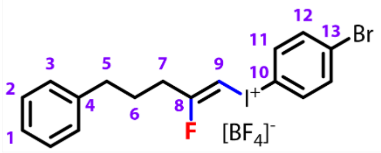

^{1}H NMR (400 MHz, $C(CD_3)_2O$): δ 8.22 – 8.18 (2H, m, H^{11}), 7.83 – 7.80 (2H, m, H^{12}), 7.30 – 7.24 (2H, t, J = 7.4 Hz, H^{2}), 7.21 – 7.14 (3H, m, H^{1+3}), 7.04 (1H, dt, J = 34.3, 0.8 Hz, H^{9}), 2.77 (2H, dt, J = 17.8, 7.4 Hz, H^{7}), 2.69 – 2.61 (2H, m, H^{5}), 1.95 – 1.87 (2H, m, H^{6})

^{13}C NMR (126 MHz, $C(CD_3)_2O$): δ 174.1 (1C, d, J = 277.5 Hz, C^{8}), 141.9 (1C, s, C^{4}), 138.2 (2C, s, C^{11}), 136.1 (2C, s, C^{12}), 129.3 (2C, s, C^{2}), 129.3 (2C, s, C^{3}), 128.1 (1C, s, C^{13}), 127.0 (2C, s, C^{1}), 111.9 (1C, s, C^{10}), 77.6 (1C, d, J = 21.1 Hz, C^{9}), 35.0 (1C, s, C^{5}), 32.0 (1C, d, J = 24.0 Hz, C^{7}), 28.1 (1C, s, C^{6})

^{19}F NMR (376 MHz, $C(CD_3)_2O$): δ -66.22 (1F, dt, J = 35.0, 17.8 Hz, F^{8}), -150.97 – -151.20 (BF_4)

HRMS (ESI) calc: $[M – BF_4]^+$ ($C_{17}H_{16}BrFI$) 446.9438; measured: 446.9429 = 2.01 ppm difference

IR (neat) v_{max}/cm^{-1}: 3095, 1635, 1473, 1385, 1261, 1116, 1054, 1010, 988, 852, 815, 745, 704, 580, 520,499, 487, 474

(Z)-[1,1'-biphenyl]-4-yl(2-fluoro-5-phenylpent-1-en-1-yl)iodonium BF_4 **(4b)**

Synthesised according to conditions B, using pent-4-yn-1-ylbenzene (270 mg, 1.25 mmol, 1 equiv.), Ag_2CO_3 (35.4 mg, 0.125 mmol, 0.1 equiv.), 4-iodo-1,1'-biphenyl (385 mg, 1.375 mmol, 1.1 equiv.), Selectfluor (664 mg, 1.875 mmol, 1.5 equiv.), K_2CO_3 (518 mg, 3.75 mmol, 3 equiv.), and pyridine.9HF (1.18 mL), in $MeNO_2$ (8.82 mL). Trituration with Et_2O (3 x 15 mL) and evaporation of the residual solvent under reduced pressure yielded a red oil (411 mg, 55% yield based on a purity of 88% determined by quantitative ^{19}F NMR assay). The compound was spectroscopically pure by ^{1}H, ^{13}C, and ^{19}F NMR.

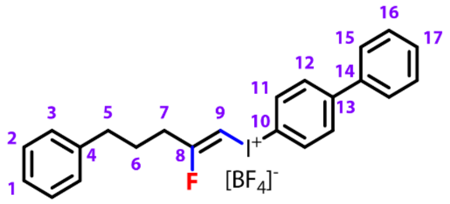

^{1}H NMR (400 MHz, $C(CD_3)_2O$): δ 8.35 – 8.31 (2H, m, H^{11}), 7.92 – 7.88 (2H, m, H^{12}), 7.76 – 7.70 (2H, m, H^{15}), 7.55 – 7.50 (2H, m, H^{16}), 7.49 – 7.44 (1H, m, H^{17}), 7.29 – 7.22 (2H, m, H^{2}), 7.20 – 7.13 (3H, m, H^{1+3}), 7.06 (1H, dt, J = 34.3, 0.9 Hz, H^{9}), 2.77 (2H, J = 18.0, 7.4 Hz, H^{7}), 2.70 – 2.62 (2H, m, H^{5}), 1.99 – 1.92 (2H, dt, J = 14.8, 7.6 Hz, H^{6}).

^{13}C NMR (126 MHz, $C(CD_3)_2O$): δ 173.0 (1C, d, J = 277.0 Hz, C^{8}), 146.2 (1C, s, C^{13}), 141.9 (1C, s, C^{4}), 139.3 (1C, s, C^{14}), 137.0 (2C, s, C^{11}), 131.3 (2C, s, C^{12}), 130.1 (2C, s, C^{16}), 129.7 (1C, s, C^{17}), 129.3 (2C, s, C^{2}), 129.2 (2C, s, C^{3}), 128.1 (2C, s, C^{15}), 126.9 (1C, s, C^{1}), 111.9 (1C, s, C^{10}), 77.5 (1H, d, J = 21.2 Hz, H^{9}), 35.0 (1C, s, C^{5}), 31.1 (1C, d, J = 24.1 Hz, C^{7}), 28.2 (1C, s, C^{6}).

^{19}F NMR (376 MHz, $C(CD_3)_2O$): δ -66.85 (1F, dt, J = 35.3, 17.8 Hz), -150.84 + -150.89 (BF_4).

HRMS (ESI) calc: $[M – BF_4]^+$ ($C_{23}H_{21}FI$) 443.0666; measured: 443.0666 = 0 ppm difference.

IR (neat) v_{max}/cm^{-1}: 3569, 3102, 3028, 2934, 2863, 1642, 1602, 1579, 1496, 1475, 1453, 1428, 1391, 1283, 1050, 1029, 989, 891, 833, 758, 745, 698, 582, 543, 520, 486.

(Z)-(2-fluoro-5-phenylpent-1-en-1-yl)(p-tolyl)iodonium BF_4 ***(4c)***

Synthesised according to conditions B, using pent-4-yn-1-ylbenzene (270 mg, 1.25 mmol, 1 equiv.), Ag_2CO_3 (35.4 mg, 0.125 mmol, 0.1 equiv.), 1-iodo-4-methylbenzene (300 mg, 1.375 mmol, 1.1 equiv.), Selectfluor (664 mg, 1.875 mmol, 1.5 equiv.), K_2CO_3 (518 mg, 3.75 mmol, 3 equiv.), and pyridine.9HF (1.18 mL), in $MeNO_2$ (8.8 mL). Trituration with Et_2O (3 x 15 mL) and evaporation of the residual solvent under reduced pressure yielded a light brown solid (367 mg, 57% yield based on a purity of 90% determined by quantitative ^{19}F NMR assay). The compound was spectroscopically pure by ^1H, ^{13}C, and ^{19}F NMR.

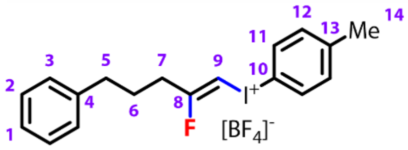

^{1}H NMR (400 MHz, $C(CD_3)_2O$): δ 814 – 8.10 (2H, m, H^{11}), 7.47 – 7.42 (2H, d, J = 8.2 Hz, H^{12}), 7.30 – 7.24 (2H, t, J = 7.5 Hz, H^2), 7.21 – 7.14 (3H, m, H^{1+3}), 6.99 (1H, dt, J = 34.5, 0.9 Hz, H^9), 2.74 (2H, dt, J = 17.7, 7.3 Hz, H^7), 2.67 – 2.58 (2H, m, H^5), 2.44 (3H, s, H^{14}), 1.90 (2H, dt, J = 14.8, 7.6 Hz, H^6).

^{13}C NMR (126 MHz, CD_3CN): δ 174.2 (1C, d, J = 277.7 Hz, C^8), 145.5 (1C, s, C^{13}), 142.1 (1C, s, C^4), 136.5 (2C, s, C^{11}), 134.1 (2C, s, C^{12}), 129.5 (2C, s, C^2), 129.4 (2C, s, C^3), 127.1 (1C, s, C^1), 109.4 (1C, s, C^{10}), 76.9 (1C, d, J = 21.7 Hz, C^9), 34.9 (1C, s, C^5), 32.0 (1C, d, J = 23.8 Hz, C^7), 28.0 (1C, s, C^6), 21.4 (1C, s, C^{14}).

^{19}F NMR (377 MHz, CD_3CN): δ -66.06 (1F, dt, J = 35.2, 17.8 Hz, F^8), -151.55 + -151.61 (BF_4).

HRMS (ESI) calc: $[M – BF_4]^+$ ($C_{18}H_{19}FI$) 381.051003; measured: 381.051471 = 1.2 ppm difference.

IR (neat) v_{max}/cm^{-1}: 3098, 3027, 2922, 2852, 1651, 1454, 1027, 755, 701.

Melting point: 83-87°C

(Z)-(2-fluoro-5-phenylpent-1-en-1-yl)(4-methoxyphenyl)iodonium BF_4 ***(4d)***

Synthesised according to conditions B, using pent-4-yn-1-ylbenzene (270 mg, 1.25 mmol, 1 equiv.), Ag_2CO_3 (35.4 mg, 0.125 mmol, 0.1 equiv.), 1-iodo-4-methoxybenzene (321 mg, 1.375 mmol, 1.1 equiv.), Selectfluor (664 mg, 1.875 mmol, 1.5 equiv.), K_2CO_3 (518 mg, 3.75 mmol, 3 equiv.), and pyridine.9HF (1.18 mL), in $MeNO_2$ (8.82 mL). Trituration with Et_2O (3 x 15 mL) and evaporation of the residual solvent under reduced pressure yielded a red oil (411 mg, 65% yield based on a purity of 96% determined by quantitative ^{19}F NMR assay). The compound was spectroscopically pure by ^{1}H, ^{13}C, and ^{19}F NMR.

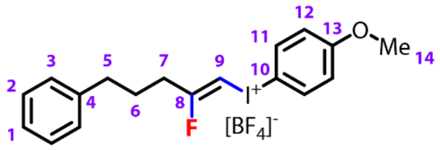

^{1}H NMR (500 MHz, $C(CD_3)_2O$): δ 8.18 – 8.14 (2H, m, H^{11}), 7.27 (2H, t, J = 7.3 Hz, H^{12}), 7.20 – 7.12 (5H, m, H^{1-3}), 6.97 (1H, d, J = 34.6, H^{9}), 3.90 (3H, s, H^{14}), 2.73 (2H, dt, J = 17.7, 7.4 Hz, H^{7}), 2.65 – 2.62 (2H, m, H^{5}), 1.90 (2H, dt, J = 14.9, 7.7 Hz, H^{6}).

^{13}C NMR (126 MHz, $C(CD_3)_2O$): δ 173.3 (1C, d, J = 276.2 Hz, C^{8}), 164.1 (1C, s, C^{13}), 141.9 (1C, s, C^{4}), 138.6 (2C, s, C^{11}), 129.3 (2C, s, C^{2}), 129.2 (2C, s, C^{3}), 126.9 (1C, s, C^{1}), 118.8 (2C, s, C^{12}), 101.9 (1C, s, C^{10}), 78.0 (1C, dd, J = 21.3, 4.4 Hz, C^{9}), 56.3 (d, J = 5.8 Hz, C^{14}), 35.0 (1C, C^{5}), 32.0 (1C, d, J = 24.4 Hz, C^{7}), 28.2 (1C, s, C^{6}).

^{19}F NMR (376 MHz, $C(CD_3)_2O$): δ -67.96 (1F, dt, J = 35.2, 17.8 Hz, F^{8}), -150.60 + -150.66 (BF_4).

HRMS (ESI) calc: $[M – BF_4]^+$ ($C_{18}H_{19}FIO$) 397.0459; measured: 397.0480 = 5.2 ppm difference.

IR (neat) v_{max}/cm^{-1}: 3096, 1638, 1572, 1487, 1460, 1441, 1301, 1253, 1187, 1017, 854, 829, 786, 750, 704, 622, 591, 511, 489.

Melting point: 97 – 100 °C

(Z)-(2-fluoro-5-phenylpent-1-en-1-yl)(o-tolyl)iodonium BF_4 ***(4e)***

Synthesised according to conditions B, using pent-4-yn-1-ylbenzene (270 mg, 1.25 mmol, 1 equiv.), Ag_2CO_3 (35.4 mg, 0.125 mmol, 0.1 equiv.), 1-iodo-2-methylbenzene (175 μL, 1.375 mmol, 1.1 equiv.), Selectfluor (664 mg, 1.875 mmol, 1.5 equiv.), K_2CO_3 (518 mg, 3.75 mmol, 3 equiv.), and pyridine.9HF (1.18 mL), in $MeNO_2$ (8.8 mL). Trituration with Et_2O (3 x 15 mL) and evaporation of the residual solvent under reduced pressure yielded a red oil (439 mg, 65% yield based on a purity of 86% determined by quantitative ^{19}F NMR assay). The compound was spectroscopically pure by ^{1}H, ^{13}C, and ^{19}F NMR.

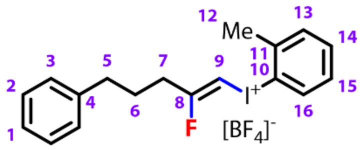

^{1}H NMR (500 MHz, $CDCl_3$): δ 8.07 (1H, d, *J* = 8.2 Hz, H^{16}), 7.50 (1H, td, *J* = 7.5, 1.2 Hz, H^{14}),7.43 (1H, dd, *J* = 7.8, 1.8 Hz, H^{13}), 7.25 (2H, dd, *J* = 6.7, 5.4 Hz, H^2), 7.23 – 7.15 (2H, m, H^{1+15}), 7.11 – 7.06 (2H, m, H^3), 6.41 (1H, d, *J* = 34.0 Hz, H^9), 2.63 (3H, s, H^{12}), 2.58 (2H, t, *J* = 7.6 Hz, H^5), 2.52 (2H, dt, *J* = 17.4, 7.5 Hz, H^7), 1.86 (2H, p, *J* = 7.5 Hz, H^6).

^{13}C NMR (126 MHz, $CDCl_3$): δ 172.8 (1C, d, *J* = 277.9 Hz, C^8), 141.5 (1C, s, C^{11}), 140.6 (1C, s, C^4), 137.7 (1C, s, C^{16}), 133.4 (1C, s, C^{14}), 131.9 (1C, s, C^{13}), 129.8 (1C, s, C^{15}), 128.7 (2C, s, C^2), 128.6 (2C, s, C^3), 126.4 (1C, s, C^1), 118.5 (1C, s, C^{10}), 75.7 (d, *J* = 22.3 Hz, C^9), 34.6 (1C, s, C^5), 31.7 (d, *J* = 24.1 Hz, C^7), 27.1 (1C, s, C^6), 25.8 (1C, s, C^{12}).

^{19}F NMR (376 MHz, $C(CD_3)_2O$): δ -67.12 (1F, dt, *J* = 35.2, 17.8 Hz, F^8), -150.78 + -150.83 (*BF_4*).

HRMS (ESI) calc: $[M – BF_4]^+$ ($C_{18}H_{19}FI$) 381.0510; measured: 381.0528 = 4.6 ppm difference.

IR (neat) v_{max}/cm^{-1}: 3545, 3103, 2934, 1644, 1496, 1455, 1275, 1211, 1026, 994, 890, 853, 750, 702, 642, 520, 486, 434.

(Z)-(2-fluoro-5-phenylpent-1-en-1-yl)(2,4,6-triisopropylphenyl)iodonium BF_4 ***(4f)***

Synthesised according to conditions B, using pent-4-yn-1-ylbenzene (270 mg, 1.25 mmol, 1 equiv.),Ag_2CO_3 (35.4 mg, 0.125 mmol, 0.1 equiv.), 2-iodo-1,3,5-triisopropylbenzene (454 mg, 1.375 mmol, 1.1 equiv.), Selectfluor (664 mg, 1.875 mmol, 1.5 equiv.), K_2CO_3 (518 mg, 3.75 mmol, 3 equiv.), and pyridine.9HF (1.18 mL), in $MeNO_2$ (8.8 mL). Trituration with Et_2O (3 x 15 mL) and evaporation of the residual solvent under reduced pressure yielded a light-yellow oil (427 mg, 53% based on a purity of 90% determined by quantitative ^{19}F NMR assay). The compound was spectroscopically pure by ^1H, ^{13}C, and ^{19}F NMR.

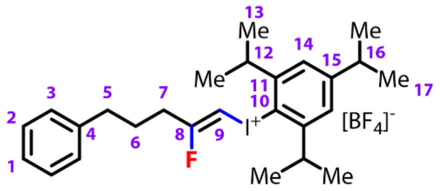

^1H NMR (500 MHz, $C(CD_3)_2O$): δ 7.43 (2H, s, H^{14}), 7.30 – 7.23 (2H, H^2), 7.20 – 7.16 (1H, m, H^1), 7.16–7.12 (2H, m, H^3), 6.87 (1H, d, J = 35.1 Hz, H^9), 3.47 (2H, hept, J = 6.6 Hz, H^{12}), 3.06 (1H, dt, J = 13.9, 7.0 Hz, H^{16}), 2.78 – 2.69 (2H, m, H^7), 2.66 – 2.60 (2H, m, H^5), 1.95 – 1.86 (2H, m, H^6), 1.35 (12H, d, J = 6.8 Hz, H^{13}), 1.28 (6H, d, J = 6.9 Hz, H^{17}).

^{13}C NMR (126 MHz, $C(CD_3)_2O$): δ 172.9 (1C, d, J = 276.4 Hz, C^8), 155.3 (1C, s, C^{15}), 152.4 (2C, s, C^{11}), 140.5 (1C, s, C^4), 128.7 (2C, s, C^2), 128.6 (2C, s, C^3), 126.4 (2C, s, C^1), 125.2 (2C, s, C^{14}), 121.4 (1C, s, C^{10}), 74.0 (1C, d, J = 21.5 Hz, C^9), 39.9 (2C, s, C^{12}), 34.7 (1C, s, C^5), 34.3 (1C, s, C^{16}), 31.8 (d, J = 24.1 Hz, C^7), 27.1 (1C, s, C^6), 24.5 (4C, s, C^{13}), 23.8 (2C, s, C^{17}).

^{19}F NMR (376 MHz, $C(CD_3)_2O$): δ -67.51 (1F, dt, J = 35.8, 18.2 Hz, F^8), -150.98 – -151.18 (BF_4).

HRMS (ESI) calc: $[M – BF_4]^+$ ($C_{26}H_{35}FI$) 493.1762; measured: 493.1760 = 0.4 ppm difference.

IR (neat) ν_{max}**/cm^{-1}:** 3092, 2964, 2933, 2872, 1706, 1646, 1584, 1497, 1462, 1388, 1262, 1223, 1053, 1032, 983, 879, 853, 749, 701, 651, 592, 530, 520, 488.

(Z)-(2-fluoro-3-(4-nitrophenoxy)prop-1-en-1-yl)(p-tolyl)iodonium BF_4 ***(4g)***

Synthesised according to conditions B, using (885 mg, 5 mmol, 1 equiv.), Ag_2CO_3 (276 mg, 1 mmol, 0.2 equiv.), 4-Iodotoluene (1.12 g, 5.5 mmol, 5.5 equiv.), Selectfluor (2.66 g, 7.5 mmol, 1.5 equiv.), K_2CO_3 (2.07 g, 15 mmol, 3 equiv.), and pyridine.9HF (4.72 mL), in $MeNO_2$ (35.3 mL). Trituration with Et_2O (2 x 50 mL) and evaporation of the residual solvent under reduced pressure yielded a light-yellow solid (1.30 g, 46% based on a purity of 93% determined by quantitative ^{19}F NMR assay). The compound was spectroscopically pure by ^{1}H, ^{13}C, and ^{19}F NMR.

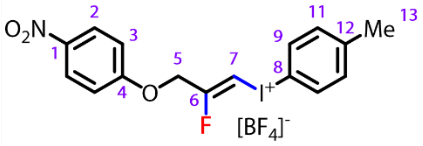

^{1}H NMR (400 MHz, $C(CD_3)_2O$): δ 8.25 – 8.17 (2H, m, *H^2*), 8.17 – 8.10 (2H, m, *H^9*), 7.47 (1H, dt, *J* = 34.2, 1.1 Hz, *H^7*), 7.45 – 7.40 (2H, m, *H^3*), 5.33 (2H, dd, *J* = 10.3, 1.0 Hz, *H^5*), 2.44 (3H, s, *H^{13}*).

^{13}C NMR (126 MHz, $C(CD_3)_2O$): δ 166.7 (1C, d, *J* = 275.6 Hz, *C^6*), 163.0 (1C, s, *C^1*), 145.0 (1C, s, *C^{12}*), 143.4 (1C, s, *C^4*), 136.7 (2C, s, *C^9*), 133.9 (2C, s, *C^{11}*), 126.6 (2C, s, *C^2*), 116.3 (2C, s, *C^3*), 109.8 (1C, s, *C^8*), 81.8 (1C, d, *J* = 17.8 Hz, *C^5*), 65.5 (1C, d, *J* = 33.0 Hz, *C^7*), 21.3 (1C, s, *C^{13}*).

^{19}F NMR (377 MHz, $C(CD_3)_2O$): δ -80.58 (1F, dt, *J* = 34.0, 10.3 Hz, *F^6*), 150.20 + -150.25 (*BF_4*).

HRMS (ESI) calc: $[M – BF_4]^+$ ($C_{16}H_{14}FINO_3$) 413.9997; measured: 413.9983 = 3.38 ppm difference.

IR (neat) v_{max}/cm^{-1}: 3106, 1650, 1589, 1495, 1331, 1302, 1271, 1224, 1181, 1112, 1028, 995, 875, 862, 849, 795, 752, 689, 631, 615, 520, 505, 469.

Melting point: 119 – 123 °C

(Z)-(3-((N-cyclopropyl-4-methylphenyl)sulfonamido)-2-fluoroprop-1-en-1-yl)(p-tolyl)iodonium BF_4 ***(4h)***

Synthesised according to conditions B, using (722 mg, 2.9 mmol, 1 equiv.), Ag_2CO_3 (80 mg, 0.29 mmol, 0.1 equiv.), 4-Iodotoluene (696 mg, 3.2 mmol, equiv.), Selectfluor (1.54 g, 4.35 mmol, 1.5 equiv.), K_2CO_3 (1.2 g, 8.7 mmol, 3 equiv.), and pyridine.9HF (2.74 mL), in $MeNO_2$ (20.6 mL). Trituration with Et_2O (2 x 20 mL) and evaporation of the residual solvent under reduced pressure yielded a light-yellow waxy solid (1.08 g, 55% based on a purity of 85% determined by quantitative ^{19}F NMR assay). The compound was spectroscopically pure by ^{1}H, ^{13}C, and ^{19}F NMR.

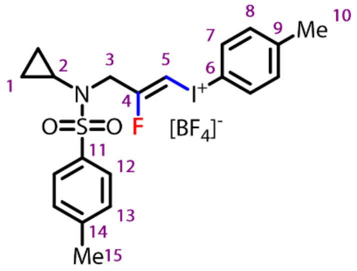

^{1}H NMR (500 MHz, $C(CD_3)_2O$): δ 8.18 – 8.01 (2H, m, H^{7}), 7.85 – 7.64 (2H, m, H^{12}), 7.48 – 7.43 (2H, m, H^{13}), 7.45 – 7.42 (2H, m, H^{8}), 7.19 (1H, d, J = 33.5 Hz, H^{5}), 4.40 (2H, dd, J = 13.3, 0.9 Hz, H^{3}), 2.45 (3H, s, H^{10}), 2.44 (3H, s, H^{15}), 2.11 – 2.05 (1H, H^{2}), 0.79 – 0.71 (2H, m, H^{1}), 0.68 – 0.57 (2H, m, H^{1}).

^{13}C NMR (126 MHz, $C(CD_3)_2O$): δ 168.7 (1C, d, J = 278.4 Hz, C^{4}), 145.2 (1C, s, C^{9}), 144.8 (1C, s, C^{14}), 136.5 (1C, s, C^{7}), 135.5 (1C, s, C^{11}), 133.8 (2C, s, C^{8}), 130.7 (2C, s, C^{13}), 128.7 (2C, s, C^{12}), 110.0 (1C, s, C^{6}), 80.9 (1C, d, J = 19.2 Hz, C^{3}), 51.1 (1C, d, J = 29.3 Hz, C^{5}), 32.1 (1C, s, C^{2}), 21.5 (1C, s, C^{10+14}), 21.3 (1C, s, C^{10+14}), 7.8 (2C, s, C^{1}).

^{19}F NMR (471 MHz, $C(CD_3)_2O$): δ -75.58 (1F, dt, J = 33.6, 13.4 Hz, F^{4}), -150.41 + -150.46 (*BF_4*)

HRMS (ESI) calc: $[M – BF_4]^+$ ($C_{20}H_{22}FINO_2S$) 486.0394; measured: 486.0372 = 4.53 ppm difference

IR (neat) v_{max}**/cm^{-1}:** 3560, 3098, 2925, 1703, 1652, 1597, 1479, 1454, 1423, 1398, 1381, 1352, 1338, 1307, 1210, 1159, 1051, 1030, 928, 875, 851, 816, 801, 777, 733, 708, 665, 616, 578, 541, 520, 476.

(Z)-(2-fluoro-2-phenylvinyl)(4-fluorophenyl)iodonium BF_4 ***(4i)***

Synthesised according to conditions B, using phenylacetylene (137 μL, 1.25 mmol, 1 equiv.), Ag_2CO_3 (35.4 mg, 0.125 mmol, 0.1 equiv.), 1-fluoro-4-iodobenzene (163 μL, 1.38 mmol, 1.1 equiv.), Selectfluor (664 mg, 1.875 mmol, 1.5 equiv.), K_2CO_3 (518 mg, 3.75 mmol, 3 equiv.), and pyridine.9HF (1.18 mL), in $MeNO_2$ (8.8 mL). Trituration with Et_2O (3 x 20 mL) and evaporation of the residual solvent under reduced pressure yielded a light-yellow solid (291 mg, 48% yield based on a purity of 88% determined by quantitative ^{19}F NMR assay). The compound was spectroscopically pure by ^{1}H, ^{13}C, and ^{19}F NMR.

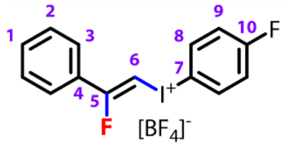

^{1}H NMR (400 MHz, $C(CD_3)_2O$): δ 8.44 – 8.37 (2H, m, H^8), 7.84 (1H, d, J = 35.2 Hz, H^6), 7.83 – 7.79 (2H, m, H^3), 7.65 – 7.60 (1H, m, H^1), 7.59 – 7.53 (2H, t, J = 7.6 Hz, H^2), 7.46 – 7.39 (2H, m, H^9).

^{13}C NMR (126 MHz, CD_3CN): δ 168.5 (1C, d, J = 266.9 Hz, C^5), 166.2 (1C, d, J = 253.8 Hz, C^{10}), 139.6 (2C, d, J = 9.5 Hz, C^8), 134.2 (1C, s, C^1), 130.3 (2C, d, J = 1.9 Hz, C^2), 128.1 (1C, d, J = 27.0 Hz, C^4), 127.5 (2C, d, J = 7.1 Hz, C^3), 120.8 (2C, d, J = 23.6 Hz, C^9), 106.7 (1C, s, C^7), 77.0 (1C, d, J = 23.4 Hz, C^6).

^{19}F NMR (376 MHz, $C(CD_3)_2O$): δ -80.75 (1F, d, J = 35.0 Hz, F^5), -106.64 (1F, tt, J = 8.8, 4.8 Hz, F^{10}), -150.43 + -150.48 (BF_4).

HRMS (ESI) calc: $[M – BF_4]^+$ ($C_{14}H_{10}F_2I$) 342.9790; measured: 342.9786 = 1.17 ppm difference.

IR (neat) v_{max}/cm^{-1}: 3109, 1622, 1575, 1481, 1448, 1399, 1290, 1236, 1166, 1008, 829, 769, 738, 682, 636, 605, 576, 520, 502.

Melting point: 128 – 132 °C

(Z)-(4-bromophenyl)(2-fluoro-2-phenylvinyl)iodonium BF_4 **(4j)**

Synthesised according to conditions B, using phenylacetylene (137 μL, 1.25 mmol, 1 equiv.), Ag_2CO_3 (35.4 mg, 0.125 mmol, 0.1 equiv.), 1-bromo-4-iodobenzene (354 mg, 1.375 mmol, 1.1 equiv.), Selectfluor (664 mg, 1.875 mmol, 1.5 equiv.), K_2CO_3 (518 mg, 3.75 mmol, 3 equiv.), and pyridine.9HF (1.178 mL), in $MeNO_2$ (8.82 mL). Trituration with Et_2O (3 x 20 mL) and evaporation of the residual solvent under reduced pressure yielded a light-yellow solid (300 mg, 49% yield based on a purity of >97% determined by quantitative ^{19}F NMR assay). The compound was spectroscopically pure by ^{1}H, ^{13}C, and ^{19}F NMR.

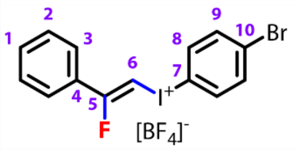

^{1}H NMR (400 MHz, $C(CD_3)_2O$): δ 8.29 – 8.24 (2H, m, H^8), 7.84 (1H, d, J = 35.0 Hz, H^6), 7.83 – 7.78 (4H, m, H^{3+9}), 7.65 – 7.59 (1H, m, H^1), 7.58 – 7.53 (2H, t, J = 7.8 Hz, H^2).

^{13}C NMR (126 MHz, CD_3CN): δ 168.6 (1C, d, J = 267.3 Hz, C^5), 138.2 (2C, s, C^8), 136.4 (2C, s, C^9), 134.2 (1C, s, C^1), 130.3 (d, J = 1.9 Hz, C^2), 128.7 (1C, s, C^{10}), 128.1 (1C, d, J = 26.9 Hz, C^4), 127.5 (2C, d, J = 7.1 Hz, C^3), 111.4 (1C, s, C^7), 76.7 (1C, d, J = 23.3 Hz, C^6).

^{19}F NMR (376 MHz, $C(CD_3)_2O$): δ -80.79 (1F, d, J = 35.1 Hz, F^5), -150.55 + -150.61 (BF_4).

HRMS (ESI) calc: $[M – BF_4]^+$ ($C_{14}H_{10}FBrI$) 402.8995; measured: 402.8990 = 1.2 ppm difference.

IR (neat) v_{max}**/cm^{-1}:** 3112, 1620, 1574, 1495, 1471, 1447, 1384, 1287, 1185, 1064, 1019, 988, 808, 774, 733, 685, 637, 601, 475.

Melting point: 173-176°C

(Z)-(4-cyanophenyl)(2-fluoro-2-phenylvinyl)iodonium BF_4 ***(4k)***

Synthesised according to conditions A but with stoichiometric Ag_2CO_3 (1.38 g, 5 mmol, 1 equiv.), using phenylacetylene (549 μL, 5 mmol, 1 equiv.), 4-iodobenzonitrile (1.26 g, 5.5 mmol, 1.1 equiv.), Selectfluor (4.43 g, 12.5 mmol, 2.5 equiv.), and pyridine.9HF (4.71 mL), in $MeNO_2$ (35.5 mL). Trituration with Et_2O (3 x 50 mL) and evaporation of the residual solvent under reduced pressure yielded a light-yellow solid (1.38 g, 63% yield based on a purity of >97%, as determined by quantitative ^{19}F NMR assay). The compound was spectroscopically pure by ^{1}H, ^{13}C, and ^{19}F NMR.

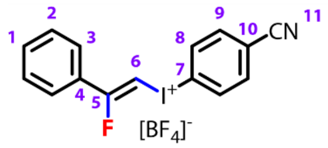

^{1}H NMR (500 MHz, CD_3CN): δ 8.22 (2H, d, *J* = 8.6 Hz, *H*^{8}), 7.88 (2H, dt, *J* = 8.6 Hz, *H*^{9}), 7.73 (2H, d, *J* = 7.6 Hz, *H*^{3}), 7.66 – 7.60 (1H, m, *H*^{1}), 7.55 (2H, t, *J* = 7.7 Hz, *H*^{2}), 7.26 (1H, d, *J* = 34.2 Hz, *H*^{6}).

^{13}C NMR (126 MHz, CD_3CN): δ 169.1 (1C, d, *J* = 268.1 Hz, *C*^{5}), 137.0 (2C, s, *C*^{8}), 136.5 (2C, s, *C*^{9}), 134.4 (1C, s, *C*^{1}), 130.3 (d, *J* = 1.9 Hz, *C*^{2}), 128.0 (d, *J* = 26.8 Hz, *C*^{4}), 127.6 (d, *J* = 7.2 Hz, *C*^{3}), 117.9 (1C, s, *C*^{7}), 117.6 (1C, s, *C*^{11}), 117.5 (1C, s, *C*^{10}), 76.7 (d, *J* = 23.2 Hz, *C*^{6}).

^{19}F NMR (376 MHz, CD_3CN): δ -78.21 (1F, d, *J* = 34.8 Hz, *F*^{5}), -149.30 + -149.36 (*BF_4*).

HRMS (ESI) calc: $[M – BF_4]^+$ ($C_{15}H_{10}NFI$) 349.9836; measured: 349.9832 = 1.14 ppm difference.

IR (neat) v_{max}/cm^{-1}: 3126, 2238, 1624, 1575, 1495, 1478, 1447, 1398, 1288, 1193, 1017, 822, 798, 774, 724, 687, 638, 604, 541, 518, 418.

(Z)-(4-(tert-butyl)phenyl)(2-fluoro-2-phenylvinyl)iodonium BF_4 ***(4l)***

Synthesised according to conditions B, using phenylacetylene (137 μL, 1.25 mmol, 1 equiv.), Ag_2CO_3 (35.4 mg, 0.125 mmol, 0.1 equiv.), 1-(tert-butyl)-4-iodobenzene (244 μL, 1.375 mmol, 1.1 equiv.), Selectfluor (664 mg, 1.875 mmol, 1.5 equiv.), K_2CO_3 (518 mg, 3.75 mmol, 3 equiv.), and pyridine.9HF (1.18 mL), in $MeNO_2$ (8.8 mL). Trituration with Et_2O (3 x 20 mL) and evaporation of the residual solvent under reduced pressure yielded a white solid (377 mg, 62% yield based on a purity of 96% determined by quantitative ^{19}F NMR assay). The compound was spectroscopically pure by ^{1}H, ^{13}C, and ^{19}F NMR.

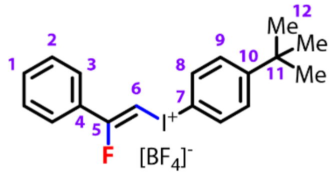

^{1}H NMR (500 MHz, $(CD_3)_2CO$): δ 8.23 (2H, d, J = 8.8 Hz, C^8), 7.83 (1H, d, J = 35.2 Hz, H^6), 7.83 – 7.79 (2H, m, H^3), 7.69 – 7.65 (2H, m, H^9), 7.65 – 7.60 (1H, m, H^1), 7.59 – 7.52 (2H, m, H^2), 1.33 (9H, s, H^{12}).

^{13}C NMR (126 MHz, $C(CD_3)_2O$): δ 167.8 (1C, d, J = 265.2 Hz, C^5), 157.5 (1C, s, C^{10}), 136.3 (2C, s, C^8), 133.8 (1C, s, C^1), 130.5 (2C, s, C^9), 130.2 (1C, d, J = 2.1 Hz, C^2), 128.3 (1C, d, J = 27.3 Hz, C^4), 127.2 (2C, d, J = 7.2 Hz, C^3), 110.3 (1C, s, C^7), 77.3 (1C, d, J = 22.9 Hz, C^6), 35.9 (1C, C^{11}), 31.2 (3C, s, C^{12}).

^{19}F NMR (376 MHz, $C(CD_3)_2O$): δ -79.81 (1F, d, J = 34.6 Hz, F^5), -151.49 + -151.54 (BF_4).

HRMS (ESI) calc: $[M – BF_4]^+$ ($C_{18}H_{19}FI$) 381.0510; measured: 381.0504 = 1.57 ppm difference.

IR (neat) v_{max}/cm^{-1}: 3100, 2967, 1618, 1574, 1481, 1448, 1395, 1286, 1200, 1019, 992, 821, 798, 772, 740, 716, 682, 634, 602, 540, 522.

Melting point: 151-155°C

(Z)-(2-fluoro-2-phenylvinyl)(*p*-tolyl)iodonium BF_4 **(4m)**

Synthesised according to conditions B, using phenylacetylene (137 *μ*L, 1.25 mmol, 1 equiv.), Ag_2CO_3 (35.4 mg, 0.125 mmol, 0.1 equiv.), 1-iodo-4-methylbenzene (299 mg, 1.375 mmol, 1.1 equiv.), Selectfluor (664 mg, 1.875 mmol, 1.5 equiv.), K_2CO_3 (518 mg, 3.75 mmol, 3 equiv.), and pyridine.9HF (1.18 mL), in $MeNO_2$ (8.8 mL). Trituration with Et_2O (3 x 20 mL) and evaporation of the residual solvent under reduced pressure yielded a white solid (409 mg, 74% yield based on a purity of 96% determined by quantitative ^{19}F NMR assay). The compound was spectroscopically pure by ^1H, ^{13}C, and ^{19}F NMR.

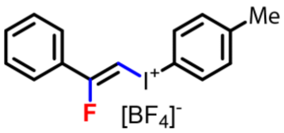

^1H NMR (500 MHz, $(CD_3)_2SO$): *δ* 8.03 (2H, d, *J* = 8.4 Hz), 7.89 (1H, d, *J* = 37.6 Hz), 7.78 – 7.69 (2H, m), 7.62 – 7.47 (4H, m), 7.37 (2H, dd, *J* = 7.8, 1.1 Hz,), 2.37 (3H, s).

^{13}C NMR (126 MHz, $(CD_3)_2SO$): *δ* 164.4 (d, *J* = 262.0 Hz), 142.5, 135.1, 132.4, 129.3, 127.4 (d, *J* = 28.1 Hz), 125.9 (d, *J* = 7.0 Hz), 111.8, 80.6 (d, *J* = 21.5 Hz), 30.7, 20.9.

^{19}F NMR (376 MHz, $C(CD_3)_2O$): *δ* -81.24 (d, *J* = 35.3 Hz), -150.62 (*BF_4*).

These data are consistent with those previously reported.[33]

(Z)-(2-fluoro-2-phenylvinyl)(phenyl)iodonium BF_4 **(4n)**

Synthesised according to conditions B, using phenylacetylene (137 *μ*L, 1.25 mmol, 1 equiv.), Ag_2CO_3 (35.4 mg, 0.125 mmol, 0.1 equiv.), iodobenzene (153 *μ*L, 1.375 mmol, 1.1 equiv.), Selectfluor (664 mg, 1.875 mmol, 1.5 equiv.), K_2CO_3 (518 mg, 3.75 mmol, 3 equiv.), and pyridine.9HF (1.18 mL), in $MeNO_2$ (8.8 mL). Trituration with Et_2O (3 x 20 mL) and evaporation of the residual solvent under reduced pressure yielded a white solid (382 mg, 72% yield based on a purity of 97% determined by quantitative ^{19}F NMR assay). The compound was spectroscopically pure by ^{1}H, ^{13}C, and ^{19}F NMR.

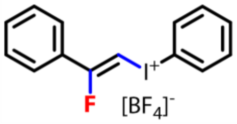

^{1}H NMR (500 MHz, $(CD_3)_2SO$): *δ* 8.23 – 8.08 (2H, m), 7.93 (1H, d, *J* = 37.6 Hz), 7.79 – 7.72 (2H, m), 7.73 – 7.66 (1H, m), 7.63 – 7.47 (6H, m).

^{13}C NMR (126 MHz, $(CD_3)_2SO$): *δ* 164.6 (d, *J* = 262.2 Hz), 135.1, 132.5, 132.1, 131.9, 129.3 (d, *J* = 1.9 Hz), 127.4 (d, *J* = 28.0 Hz), 126.0 (d, *J* = 7.0 Hz), 115.5, 80.5 (d, *J* = 21.6 Hz).

^{19}F NMR (376 MHz, $C(CD_3)_2O$): *δ* -84.48 (d, *J* = 37.6 Hz), -146.73 – -150.38 (*BF_4*).

These data are consistent with those previously reported.[34]

Unsuccessful Akynes

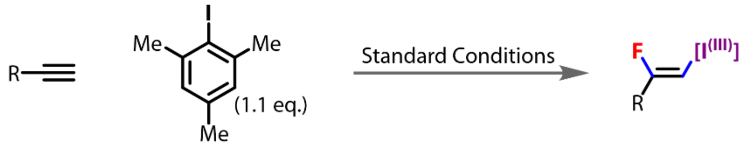

Too electron-rich/easily oxidized

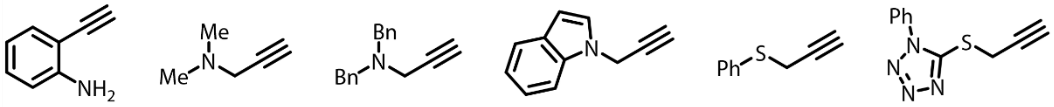

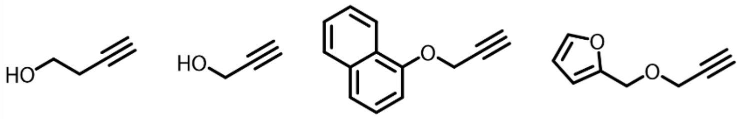

Acid-labile groups not tolerated

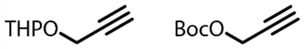

Mechanistic Study (Figure 3)

Preparation of (phenylethynyl)silver

Synthesised according to a literature procedure.[35] To round-bottom flask, equipped with a stirrer bar and wrapped in aluminium foil, was added silver nitrate (1.04 g, 6.15 mmol, 1 equiv.), acetonitrile (70 mL), phenylacetylene (2.03 mL, 18.45 mmol, 3 equiv.), and NEt_3 (2.56 mL, 18.45 mmol, 3 equiv.). A white precipitate formed immediately upon the addition of NEt_3, and suspension was stirred for 48 hours. The white solid was filtered and washed with acetonitrile (3 x) and methanol (3 x), before being evaporated under high-vacuum to afford the product (307 mg, 31%).

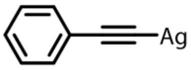

^{1}H NMR (400 MHz, C_5D_5N): δ 7.70 – 7.58 (2H, m), 7.21 – 7.16 (1H, m), 7.16 – 7.08 (2H, m)

^{13}C NMR (101 MHz, C_5D_5N): δ 133.0, 129.0, 128.2, 125.6, 122.9, 102.2

HRMS (MALDI) calc: $[M]^-$ ($C_{16}H_{10}Ag$) 308.9839; measured: 308.9845 = 1.94 ppm difference

These data are consistent with those previously reported.[36]

Observation of (Phenylethynyl)silver Formation Under Conditions A/$MeNO_2$

(A) Phenylacetylene (44 μL, 0.4 mmol) was dissolved in a mixture of $MeNO_2$ (1.6 mL) and pyridine.9HF (374 μL), and ^{1}H NMR **(A)** was measured after 5 minutes.

(B): To an HDPE vial equipped with a stirrer bar, was added Ag_2CO_3 (110 mg, 0.4 mmol, 1 equiv.), $MeNO_2$ (1.6 mL), Pyridine.9HF (374 μL), followed by phenylacetylene (44 μL, 0.4 mmol, 1 equiv.). ^{1}H NMR **(B)** was measured after stirring for 5 minutes.

(C): To an HDPE vial, was added the 'independently-prepared' (phenylethynyl)silver prepared by the above literature procedure (83.6 mg, 0.4 mmol), $MeNO_2$ (1.6 mL), and pyridine.9HF (374 μL). When the (phenylethynyl)silver appeared to be fully dissolved, the ^{1}H NMR **(C)** was measured.

(D): Reference ^{1}H NMR measurement of $MeNO_2$.

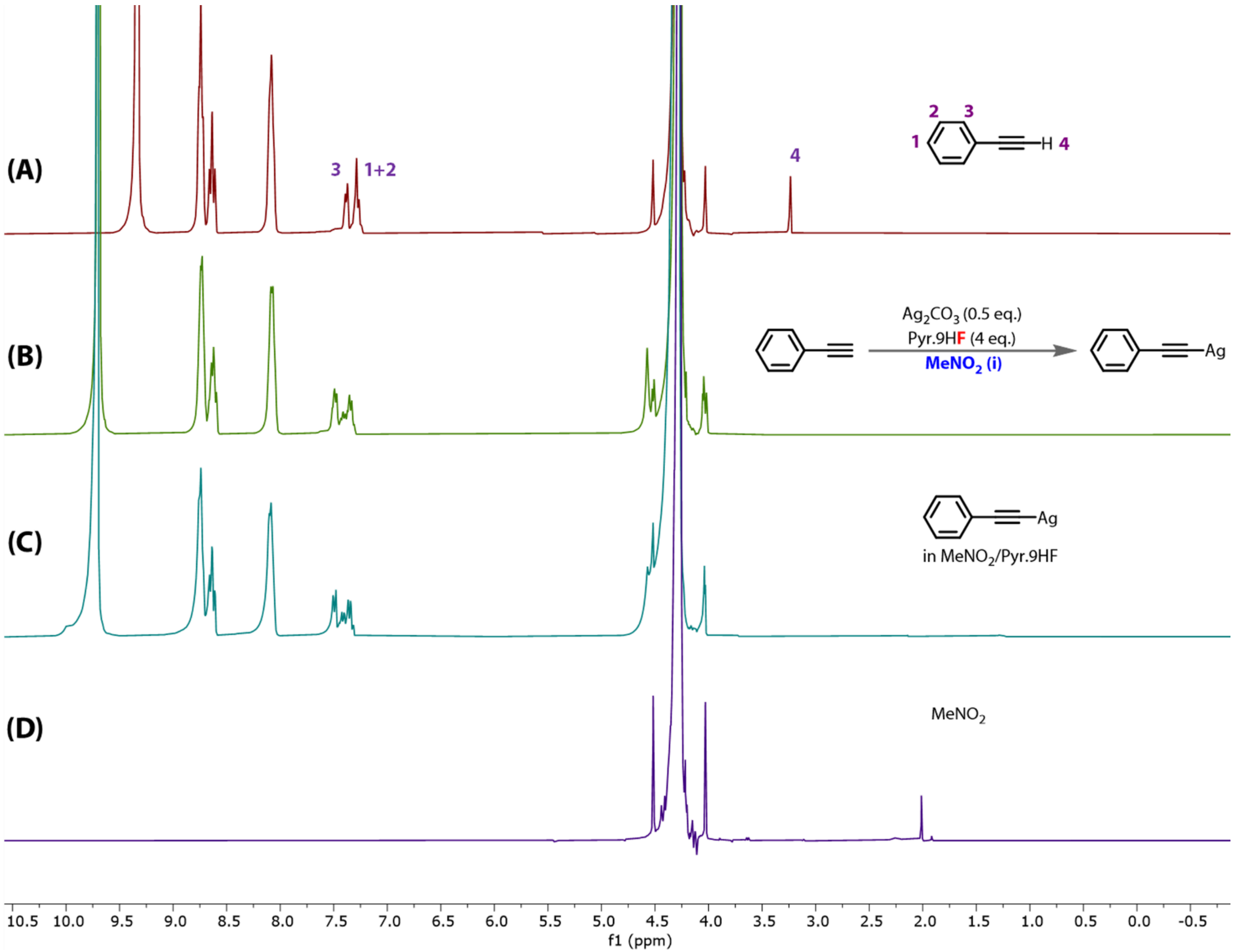
(A)
(B)
(C)
(D)
3
1+2
4
1
2
3
H 4
Ag$_2$CO$_3$ (0.5 eq.)
Pyr.9HF (4 eq.)
MeNO$_2$ (i)
Ag
Ag
in MeNO$_2$/Pyr.9HF
MeNO$_2$
10.5 10.0 9.5 9.0 8.5 8.0 7.5 7.0 6.5 6.0 5.5 5.0 4.5 4.0 3.5 3.0 2.5 2.0 1.5 1.0 0.5 0.0 -0.5
f1 (ppm)

Observation of (Phenylethynyl)silver Formation using Dimethyl Carbonate Solvent

To an HDPE vial equipped with a stirrer bar, was added Ag_2CO_3 (1.38 g, 5 mmol, 1 equiv.), dimethyl carbonate (35.3 mL), phenylacetylene (550 μL, 5 mmol, 1 equiv.), and pyridine.9HF drop-wise (4.71 mL, 180 mmol, 36 equiv. HF). The resulting white precipitate was filtered. The solid was analysed by ^{1}H NMR and HRMS, matching the above-mentioned data.

Subjection of an Internal Alkyne to Conditions B

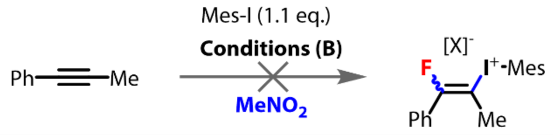

Following conditions B, using 1-phenyl-1-propyne (62.6 μL, 0.5 mmol, 1 equiv.), Ag_2CO_3 (13.8 mg, 0.05 mmol, 1 equiv.), 2-iodomesitylene (135 mg, 1.375 mmol, 1.1 equiv.), Selectfluor (266 mg, 0.75 mmol, 1.5 equiv.), K_2CO_3 (207 mg, 1.5 mmol, 3 equiv.), and pyridine.9HF (470 µL), in $MeNO_2$ (3.5 mL). No FVI formation was detected by NMR analysis.

Use of Phenylethynyl-silver(I) as the Catalyst/Pre-catalyst

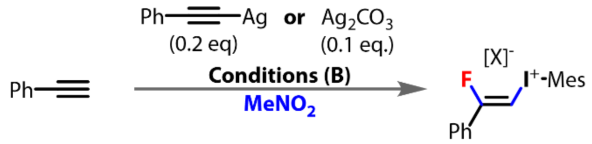

Following the procedure analogous to conditions B, using phenylacetylene (55 μL, 0.5 mmol, 1 equiv.), (phenylethynyl)silver(I) (40.8 mg, 0.2 mmol, 0.2 equiv.), 2-iodomesitylene (135 mg, 0.55 mmol, 1.1 equiv.), Selectfluor (266 mg, 0.75 mmol, 1.5 equiv.), K_2CO_3 (207 mg, 1.5 mmol, 3 equiv.), and pyridine.9HF (470 µL), in $MeNO_2$ (3.5 mL). The yield was determined by ^{19}F NMR relative to hexafluorobenzene.

The following reaction was set up in parallel with the above. Phenylacetylene (55 μL, 0.5 mmol, 1 equiv.) Ag_2CO_3 (13.8 mg, 0.05 mmol, 0.1 equiv.), 2-iodomesitylene (135 mg, 1.375 mmol, 1.1 equiv.), Selectfluor (266 mg, 0.75 mmol, 1.5 equiv.), K_2CO_3 (207 mg, 1.5 mmol, 3 equiv.), and pyridine.9HF (470 µL), in $MeNO_2$ (3.5 mL). The yield was determined by ^{19}F NMR relative to hexafluorobenzene.

Observation of an Alkynyl-iodonium Intermediate under Reaction Conditions

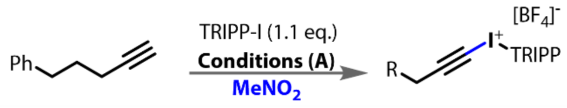

(5-Phenylpent-1-yn-1-yl)(2,4,6-triisopropylphenyl)iodonium BF_4 ***(III-b)***

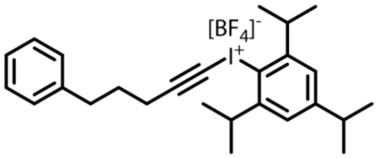

Synthesised according to conditions A, using pent-4-yn-1-ylbenzene (270 mg, 1.25 mmol, 1 equiv.), Ag_2CO_3 (35.4 mg, 0.125 mmol, 0.1 equiv.), 2-iodo-1,3,5-triisopropylbenzene (454 mg, 1.375 mmol, 1.1 equiv.), Selectfluor (664 mg, 1.875 mmol, 1.5 equiv.), and pyridine.9HF (1.18 mL), in $MeNO_2$ (8.82 mL). Trituration with Et_2O (2 x 30 mL) and evaporation of the residual solvent under reduced pressure yielded cream solid (413 mg, 59%).

^{1}H NMR (500 MHz, $C(CD_3)_2O$): δ 7.75 – 7.68 (1H, m), 7.59 (1H, t, *J* = 1.5 Hz), 7.43 (2H, s), 7.35 (1H, td, *J* = 7.5, 1.3 Hz), 7.26 (1H, dq, *J* = 7.8, 0.7 Hz), 7.24 – 7.18 (1H, m), 3.50 (2H, hept, *J* = 6.7 Hz), 3.11 – 3.00 (3H, m), 2.91 (2H, t, *J* = 6.2 Hz), 2.00 (2H, p, *J* = 6.3 Hz), 1.37 (12H, d, *J* = 6.8 Hz), 1.28 (6H, d, *J* = 6.9 Hz)

^{13}C NMR (126 MHz, $C(CD_3)_2O$): δ 156.1, 154.4, 153.0, 140.4, 132.4, 131.7, 130.3, 127.6, 126.8, 126.1, 121.0, 96.4, 40.5, 36.0, 34.8, 30.6, 30.4, 24.5, 23.93, 23.92.

HRMS (ESI) calc: $[M – BF_4]^+$ ($C_{26}H_{34}I$) 473.1700; measured: 473.1699 = 0.21 ppm difference

IR (neat) v_{max}**/cm^{-1}:** 2966, 2934, 2877, 1464, 1056, 1012, 889, 757, 519

The Role of Silver(I) in Alkynyl-iodonium Fluorination

Preparation of Alkynyl-iodonium Salts

mesityl(phenylethynyl)iodonium – tosylate and *-BF_4*

Synthesised according to an adapted literature procedure.[37] To a round-bottom flask, equipped with a stirrer bar, was added 2-iodomesitylene (2.46 g, 10 mmol, 1 equiv.), DCM (100 mL), *m*-CPBA (2.24 g, assuming 77% active oxidant, 10 mmol, 1 equiv.), and by *p*-TsOH·H_2O (1.9 g, 10 mmol, 1 equiv.). The suspension was stirred at room temperature until it became homogeneous, at which point phenylacetylene (1.1 mL, 10 mmol, 1 equiv.) was added and the solution was stirred for a further 24 hours. The solvent was evaporated under reduced pressure, and the resulting crude mixture was triturated by sonication in Et_2O (2 x 100 mL). The ether was decanted and the trace solvent was evaporated under reduced pressure to afford the compound as a light cream solid (3.17 g, 73%). The compound underwent decomposition over a timescale that precluded characterisation by ^{13}C NMR. Nevertheless, the ^{1}H NMR and HRMS data are consistent with the structure, and indicate adequate purity by ^{1}H NMR immediately after purification. For this reason, portions of the solid were crystallised slowly out of a DCM/pentane mixture while kept in a freezer (~-5°C). The solid was quickly dissolved in DCM and washed with saturated aq. $NaBF_4$. After solvent removal and confirmation by ^{1}H NMR, the solid was used immediately in the subsequent experiment.

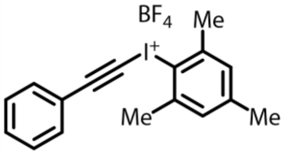

^{1}H NMR (300 MHz, $C(CD_3)_2O$): δ 7.63 – 7.50 (3H, m), 7.50 – 7.41 (2H, m), 7.32 (2H, s), 2.82 (6H, s), 2.39 (3H, s).

HRMS (ESI) calc: $[M]^+$ ($C_{17}H_{16}I$) 347.0291; measured: 347.0289 = 0.57 ppm difference.

phenyl(phenylethynyl)iodonium OTs

The same procedure and scale for the mesityl(phenylethynyl)iodonium salt was employed, but on a 5 mmol scale, using iodobenzene instead of 2-iodomesitylene, and with precipitation out of MeCN/Et_2O. (white solid, 810 mg, 34% yield). The compound was slightly unstable in solution ($CDCl_3$).

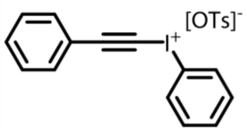

^{1}H NMR (400 MHz, $CDCl_3$): δ 8.21 – 8.03 (2H, m), 7.71 – 7.60 (2H, m), 7.60 – 7.53 (1H, m), 7.48 – 7.39 (5H, m), 7.38 – 7.31 (2H, m), 7.11 – 7.01 (2H, m), 2.31 (3H, s).

^{13}C NMR (101 MHz, $CDCl_3$): δ 141.6, 140.3, 134.0, 133.1, 132.1, 131.9, 131.0, 128.9, 128.7, 126.2, 120.3, 119.2, 105.4, 39.3, 21.5.

These data are consistent with those previously reported. [38]

Fluorination of Mesityl(phenylethynyl)iodonium: Silver versus Silver-free Conditions

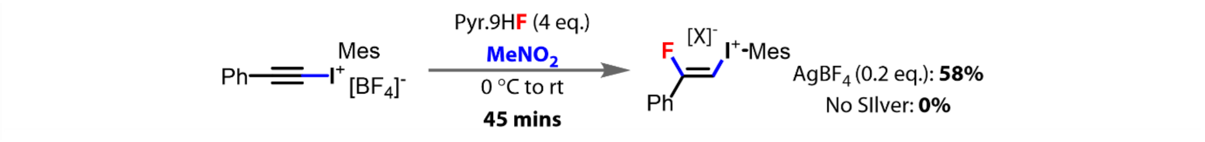

To an HDPE vial equipped with a stirrer bar, in a nitrogen-filled glovebox, was added $AgBF_4$ (2 mg, 0.1 mmol, 0.2 equiv.). The vial was removed from the glovebox and K_2CO_3 (20.7 mg, 0.15 mmol, 3 equiv.) was added, followed by MeNO2 (450 µL). The vial was placed in an ice bath, and pyridine.9HF (47 µL) was added drop-wise while stirring. After 5 minutes, mesityl(phenylethynyl)iodonium BF_4 (21.7 mg, 0.05 mmol, 1 equiv.) was added, and the vial was left to stir while warming to room temperature. The yield was determined by ^{19}F NMR of an aliquot from the reaction mixture, relative to hexafluorobenzene. As a direct comparison, a reaction following the same procedure was set up in parallel with the above, but without silver.

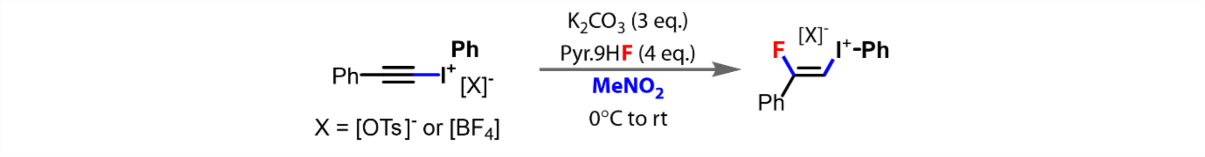

Table S3

Entry	Counterion ('[X]⁻')	$AgBF_4$ eq.	Reaction time / hrs	NMR Yield / %
**1**	OTs	0.2	1	55
**2**	OTs	0	18	0
**3**	BF_4	0.2	1.5	44
**4**	BF_4	0	1.5	0

To an HDPE vial equipped with a stirrer bar, in a nitrogen-filled glovebox, was added $AgBF_4$ (3.9 mg, 0.02 mmol, 0.2 equiv.). The vial was removed from the glovebox and K_2CO_3 (41 mg, 0.3 mmol, 3 equiv.) was added, followed by $MeNO_2$ (900 µL). The vial was placed in an ice bath, and pyridine.9HF (94 µL) was added drop-wise while stirring. After 5 minutes, phenyl(phenylethynyl)iodonium (47.6 mg, 0.1 mmol, 1 equiv.), and the vial was left to stir while warming to room temperature. After the specified time, 4,4'-difluorobiphenyl was added, and into a small vial, was added an aliquot (300 µL) a further 300 µL $MeNO_2$, and distilled water (300 µL). The vial was shaken thoroughly, and the organic layer was analysed by ^{19}F NMR to determine the yield of the reaction (integration relative to 4,4'difluorobiphenyl). As a direct comparison, a reaction following the same procedure was set up in parallel with the above,

but without silver. For reactions employing the BF_4 counterion, the tosylate iodonium (0.1 mmol weighed out) was washed with saturated aqueous $NaBF_4$ immediately before use.

Z-FVI Derivatisations: Reaction Procedures and Products

Bromination

(Z)-(5-bromo-4-fluoropent-4-en-1-yl)benzene ***(5a)***

To a Schlenk tube equipped with a stirrer bar, was added the *Z*-FVI (248 mg, 0.5 mmol, 1 equiv.), TBAB (177 mg, 0.55 mmol, 1.1 equiv.), CuTC (19 mg, 0.1 mmol, 0.2 equiv.), and 2,2'-bipyridyl (15.6 mg, 0.1 mmol, 0.2 equiv.). The tube was sealed with a septum and placed under a nitrogen atmosphere by cycling onto a Schlenk line. Degassed MeCN (5 mL) was added and the mixture was stirred briefly at room temperature, then heated to 60 °C. After stirring at this temperature for 18 hours, the reaction mixture was evaporated under reduced pressure, and the resulting crude mixture was subjected to flash-column chromatography (0 to 20% Et_2O in pentane) to afford the product as a colourless oil (81 mg, 67% yield, as a 1:0.04 mixture of the 1,2-bromofluoroalkene:1,2-iodofluoroalkene).

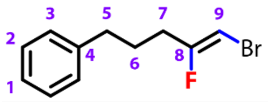

^{1}H NMR (400 MHz, $CDCl_3$): δ 7.33 – 7.27 (2H, m, H^2), 7.24 – 7.15 (3H, m, H^{1+3}), 5.32 (1H, dt, *J* = 28.1, 0.9 Hz, H^9), 2.66 (2H, t, J = 7.6 Hz, H^5), 2.42 – 2.12 (2H, m, H^7), 2.05 – 1.74 (2H, m, H^6).

^{13}C NMR (126 MHz, $CDCl_3$): δ 162.4 (1C, d, *J* = 261.7 Hz, C^8), 141.2 (1C, s, C^4), 128.59 (2C, s, C^2), 128.54 (2C, d, *J* = 6.8 Hz, C^3), 126.2 (1C, s, C^1), 82.8 (1C, d, *J* = 22.1 Hz, C^9), 34.8 (1C, s, C^5), 31.4 (1C, d, *J* = 25.5 Hz, C^7), 27.3 (1C, s, C^6).

^{19}F NMR (376 MHz, $CDCl_3$): δ -94.93 (1F, dt, *J* = 28.1, 17.5 Hz, F^8). A minor peak corresponding to the *Z*-1,2-Iodofluoroalkene was observed at -79.43 (dt, *J* = 34.7, 16.6 Hz).

HRMS (EI) calc: $[M]^+$ ($C_{11}H_{12}FI$) 242.0101; measured: 242.0097 = 1.65 ppm difference.

IR (neat) v_{max}**/cm^{-1}:** 3103, 3027, 2939, 2863, 1671, 1603, 1496, 1453, 1428, 1259, 1138, 1080, 1030, 979, 889, 852, 805, 746, 698, 643, 563, 520, 489

.

(Z)-1-((3-bromo-2-fluoroallyl)oxy)-4-fluorobenzene ***(5b)***

To a Schlenk tube equipped with a stirrer bar, was added the *Z*-FVI (251 mg, 0.5 mmol, 1 equiv.), TBAB (177 mg, 0.55 mmol, 1.1 equiv.), CuTC (19 mg, 0.1 mmol, 0.2 equiv.), and 2,2'-bipyridyl (15.6 mg, 0.1 mmol, 0.2 equiv.). The tube was sealed with a septum and placed under a nitrogen atmosphere by cycling onto a Schlenk line. Degassed MeCN (5 mL) was added and the mixture was stirred briefly at room temperature, then heated to 60 °C. After stirring at this temperature for 18 hours, the reaction mixture was evaporated under reduced pressure, and the resulting crude mixture was subjected to flash-column chromatography (0 to 20% Et_2O in pentane) to afford the product as a white solid (71 mg, 57% yield).

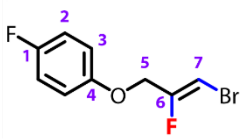

^{1}H NMR (500 MHz, $CDCl_3$): δ 7.02 – 6.97 (2H, m, H^2), 6.89 – 6.85 (2H, m, H^3), 5.82 (1H, d, *J* = 27.1 Hz, H^7), 4.55 (2H, dd, *J* = 11.8, 0.9 Hz, H^5).

^{13}C NMR (126 MHz, $CDCl_3$): δ 158.11 (1C, d, *J* = 240.1 Hz, C^1), 157.32 (1C, d, *J* = 261.8 Hz, C^6), 153.9 (1C, d, *J* = 2.3 Hz, C^4), 116.5 (2C, d, *J* = 8.2 Hz, C^3), 116.3 (2C, d, *J* = 23.3 Hz, C^2), 88.1 (1C, d, *J* = 19.1 Hz, C^7), 66.4 (d, *J* = 32.3 Hz, C^5).

^{19}F NMR (376 MHz, $CDCl_3$): δ -104.65 (1F, dt, *J* = 27.3, 11.7 Hz, F^6), -121.98 – -122.05 (1F, m, F^1).

HRMS (EI) calc: $[M]^+$ ($C_9H_7OF_2Br$) 247.9643; measured: 247.9639 = 1.61 ppm difference

(Z)-2-(3-bromo-2-fluoroallyl)isoindoline-1,3-dione **(5c)**

To a Schlenk tube equipped with a stirrer bar, was added the *Z*-FVI (269 mg, 0.5 mmol, 1 equiv.), TBAB (177 mg, 0.55 mmol, 1.1 equiv.), CuTC (19 mg, 0.1 mmol, 0.2 equiv.), and 2,2'-bipyridyl (15.6 mg, 0.1 mmol, 0.2 equiv.). The tube was sealed with a septum and placed under a nitrogen atmosphere by cycling onto a Schlenk line. Degassed MeCN (5 mL) was added and the mixture was stirred briefly at room temperature, then heated to 60 °C. After stirring at this temperature for 18 hours, the reaction mixture was evaporated under reduced pressure, and the resulting crude mixture was subjected to flash-column chromatography (25% EtOAc in pentane) to afford the product as a white solid (84 mg, 59% yield).

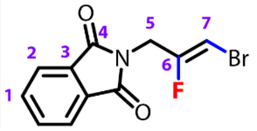

^{1}H NMR (500 MHz, CDCl$_3$): δ 7.91 - 7.86 (2H, m, *H*^{2}), 7.78 - 7.74 (2H, *m*, *H*^{1}), 5.81 (1H, d, *J* = 26.8, *H*^{7}), 4.46 (2H, d, *J* = 14.8 Hz, *H*^{5}).

^{13}C NMR (126 MHz, CDCl$_3$): δ 167.3 (2C, s, *C*^{4}), 156.1 (d, *J* = 263.8 Hz, *C*^{6}), 134.5 (2C, s, *C*^{1}), 131.9 (2C, s, *C*^{3}), 123.8 (2C, s, *C*^{2}), 88.1 (1C, d, *J* = 18.9 Hz, *C*^{7}), 37.8 (1C, d, *J* = 33.5 Hz, *C*^{5}).

^{19}F NMR (376 MHz, CDCl$_3$): δ -102.42 (1F, dt, *J* = 26.6, 14.8 Hz, *F*^{6}).

HRMS (ESI) calc: [M-Br]$^+$ ($C_{11}H_7NO_2FBr$) 204.0455; measured: 204.0454 = 0.49 ppm difference.

IR (neat) v_{max}**/cm^{-1}:** 3103, 2922, 1777, 1707, 1467, 1422, 1395, 1314, 1246, 1296, 1144, 1111, 1090, 942, 905, 791, 767, 722, 711, 630, 531, 497.

(Z)-N-(3-bromo-2-fluoroallyl)-N-(4-fluorobenzyl)-4-methylbenzenesulfonamide **(5d)**

In a nitrogen-filled glovebox, to a Schlenk tube equipped with a stirrer bar, was added the *Z*-FVI (582 mg, 1 mmol, 1 equiv.), TBAB (355 mg, 1.1 mmol, 1.1 equiv.), $(CuOTf)_2$.toluene (38.8 mg, 0.075 mmol, 0.15 equiv $Cu^{(I)}$), and 2,2'-bipyridyl (31 mg, 0.2 mmol, 0.2 equiv.). The tube was sealed with a septum, removed from the glovebox, and cycled onto a Schlenk line to maintain the nitrogen atmosphere. Degassed MeCN (12 mL) was added and the mixture was stirred briefly at room temperature before being heated to 60 °C. After stirring at this temperature for 18 hours, the reaction mixture was evaporated under reduced pressure, and the resulting crude mixture was subjected to flash-column chromatography (10 to 20% EtOAc in pentane) to afford the product as a white solid (291 mg, 70% yield).

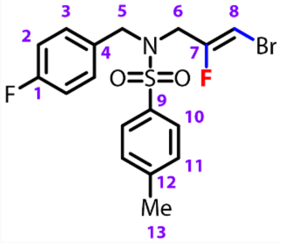

^{1}H NMR (500 MHz, CDCl$_3$): δ 7.78 – 7.66 (2H, m, H^{10}), 7.33 (2H, d, *J* = 8.0 Hz, H^{11}), 7.31 – 7.22 (2H, m, H^3), 7.02 (2H, t, *J* = 8.6 Hz, H^2), 5.44 (1H, d, *J* = 27.0 Hz, H^8), 4.35 (2H, s, H^5), 3.91 (2H, d, *J* = 16.9 Hz, H^6), 2.45 (3H, s, H^{13}).

^{13}C NMR (126 MHz, CDCl$_3$): δ 162.7 (1C, d, *J* = 247.0 Hz, C^1), 156.9 (1C, d, *J* = 265.6 Hz, C^7), 144.0 (1C, s, C^{12}), 136.8 (1C, s, C^9), 130.9 (1C, d, *J* = 3.2 Hz, C^4), 130.4 (2C, d, *J* = 8.3 Hz, C^3), 129.9 (2C, s,C^{11}), 127.4 (2C, s, C^{10}), 115.9 (2C, d, *J* = 21.7 Hz, C^2), 88.4 (1C, d, *J* = 19.6 Hz, C^8), 50.5 (1C, s, C^5), 46.0 (1C, d, *J* = 27.1 Hz, C^6), 21.7 (1C, s, C^{13}).

^{19}F NMR (376 MHz, CDCl$_3$): δ -100.67 (1F, dt, *J* = 27.0, 17.0 Hz, F^7), -113.63 (1F, tt, *J* = 8.3, 5.2 Hz, F^7).

HRMS (APCI) calc: $[M+H]^+$ ($C_{17}H_{16}NO_2F_2SBr$) 416.0126; measured: 416.0129 = 0.72 ppm difference.

IR (neat) ν_{max}**/cm^{-1}:** 3662, 2990, 2991, 1517, 1338, 1241, 1220, 1159, 1066, 878, 767, 672, 529, 551.

Chlorination

(Z)-(5-chloro-4-fluoropent-4-en-1-yl)benzene ***(6a)***

To a Schlenk tube equipped with a stirrer bar, was added (Z)-(2-fluoro-5-phenylpent-1-en-1-yl)(mesityl)iodonium BF_4 (496 mg, 1 mmol, 1 equiv.), benzyltriethylammonium chloride (125 mg, 0.55 mmol, 0.5 equiv.), and 2,2'-bipyridyl (156 mg, 1 mmol, 1 equiv.). The Schlenk tube was then transferred into a nitrogen-filled glovebox, and CuCl (99 mg, 1 mmol, 1 equiv.) was added. The tube was sealed with a septum, removed from the glovebox, and cycled onto a Schlenk line to maintain the nitrogen atmosphere. Degassed MeCN (5 mL) was added and the mixture was stirred briefly at room temperature before being heated to 55 °C. After stirring at this temperature for 18 hours, the reaction mixture was evaporated under reduced pressure, and the resulting crude mixture was subjected to flash-column chromatography (0 to 20% EtOAc in pentane) to afford the product as a colourless oil (99 mg, 50% yield, as a 1:0.1 mixture of the 1,2-chlorofluoroalkene:1,2-iodofluoroalkene).

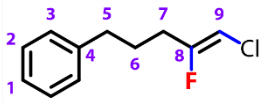

^{1}H NMR (500 MHz, $CDCl_3$) δ 7.31 (2H, m, H^2), 7.24 – 7.20 (1H, m, H^1), 7.20 – 7.17 (2H, m, H^3), 5.34 (1H, d, J = 24.6 Hz, H^9), 2.67 (2H, t, J = 7.6 Hz, H^5), 2.33 – 2.13 (2H, m, H^7), 1.88 (2H, ddd, J = 15.0, 8.2, 6.9 Hz, H^6).

^{13}C NMR (126 MHz, $CDCl_3$) δ 160.9 (1C, d, J = 262.1 Hz, C^8), 141.3 (1C, s, C^4), 128.60 (2C, s, C^2), 128.56 (2C, s, C^3), 126.3 (1C, s, C^1), 96.3 (1C, d, J = 18.7 Hz, C^9), 34.9 (1C, s, C^5), 30.5 (1C, d, J = 24.5 Hz, C^7), 27.3 (1C, s, C^6).

^{19}F (376 MHz, $CDCl_3$) δ -104.08 (1F, dt, J = 24.6, 18.0 Hz, F^8).

HRMS (EI) calc: [M]+ ($C_{11}H_{12}ClF$) 198.0606; measured: 198.0602 = 2.02 ppm difference.

IR (neat) v_{max}**/cm^{-1}:** 3102, 3063, 3027, 2941, 2865, 1680, 1603, 1496, 1453, 1429, 1309, 1286, 1267, 1163, 1116, 1080, 1030, 980, 899, 862, 845, 820, 749, 698, 491.

Cu-Catalysed C-S Coupling

Thiol Coupling

Reaction Optimization

To a Schlenk tube equipped with a stirrer bar, was added (Z)-(2-fluoro-3,3-dimethylbut-1-en-1-yl)(mesityl)iodonium BF4 (21.7 mg, 0.05 mmol, 1 equiv.), CuTC (either 0.95 mg for 0.005 mmol (0.1 equiv.), or 0.48 mg, for 0.0025 mmol (0.05 equiv.)), and ligand (in the same number of equivalents as the copper). The vessel was sealed and placed under a nitrogen atmosphere by cycling onto a Schlenk line. Degassed solvent (500 μL) and by 4-fluorothiophenol (5.9 μL , 0.055 mmol, 1.1 equiv.) were added and the mixture was stirred for 24 hours at the specified temperature. NMR yields were calculated relative to hexafluorobenzene.

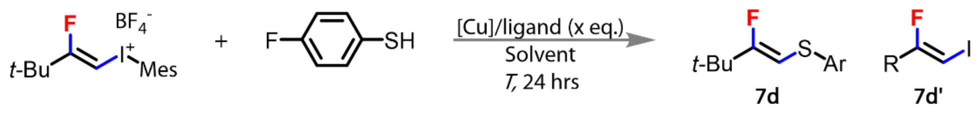

Table S4

Entry	Catalyst / (eq.)	Solvent	T / °C	RSM /%	Yield / % (7d,7d’)
1	CuTC/Bipy (0.1)	EtOAc	65	21	29, 11
2	CuTC/Bipy (0.1)	Acetone	65	34	22, 9
3	CuTC/Bipy (0.1)	PC	65	55	18, 7
4	CuTC/Bipy (0.1)	DMI	65	19	27, 13
5	CuTC/Bipy (0.1)	DMSO	65	31	17, 6
6	CuTC/Bipy (0.1)	DMF	65	9	39, 5
7	CuTC/L-Proline (0.05)	DMF	55	35	14, 11
8	CuTC/L1 (0.05)	DMF	55	13	48, 11
9	CuTC/L2 (0.05)	DMF	55	5	49, 8
10	CuTC/L3 (0.05)	DMF	55	7	51, 10
11	CuTC/2-Picolinic acid (0.05)	DMF	55	32	15, 10
12	CuTC/Xantphos (0.05)	DMF	55	85	4, 10
13	CuTC/DPPF (0.05)	DMF	55	66	23, 16
14	CuTC/L1 (0.05)	DMA	55	12	38, 7
15	CuTC/L1 (0.05)	DMPU	55	11	30, 11
16	CuTC/L1 (0.05)	DMEU	55	50	25, 9
17	CuTC/L1 (0.05)	2-Propanol	55	7	49, 18
18	CuTC/L1 (0.05)	Isobutanol	55	18	47, 17
19	CuTC/L1 (0.05)	t-BuOH	55	23	44, 14
20	CuTC/L4 (0.05)	Isobutanol	55	37	29, 16
21	CuTC/L5 (0.05)	Isobutanol	55	63	21, 13
22	CuTC/L6 (0.05)	Isobutanol	55	4	53, 24
23	CuTC/L7 (0.05)	Isobutanol	55	37	34, 21
24	CuTC/L8 (0.05)	Isobutanol	55	42	28, 16
25[a]	CuTC/Bipy (0.2)	Isobutanol	55	0	70, 15

^{19}F NMR Yields and conversions were determined by relative integration to hexafluorobenzene. All reactions were performed under a nitrogen atmosphere. RSM = Remaining starting material (FVI). DMI = Dimethyl isosorbide. DMEU = 1,3-Dimethyl-2-imidazolidinone. [a] Reaction conducted on a 0.5 mmol scale

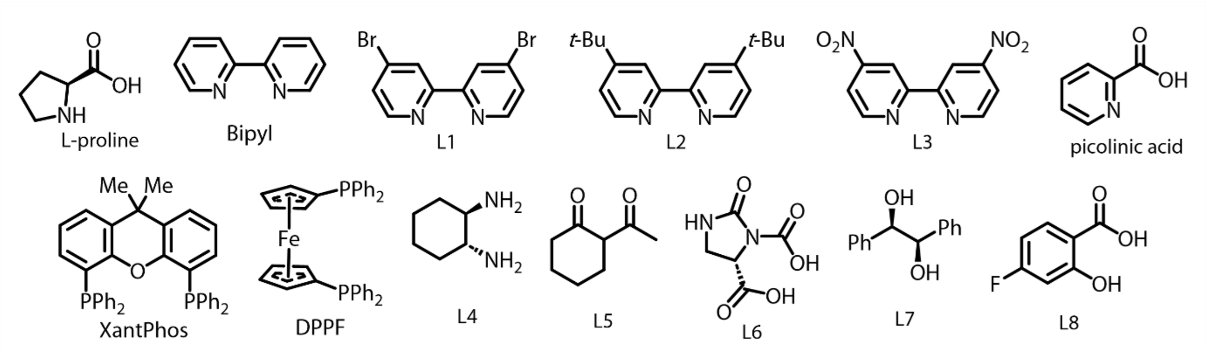

(Z)-(2-fluoro-5-phenylpent-1-en-1-yl)(4-fluorophenyl)sulfane ***(7a)***

To a Schlenk tube equipped with a stirrer bar, was added *(Z)*-(2-fluoro-5-phenylpent-1-en-1-yl)(mesityl)iodonium BF_4 (248 mg, 0.5 mmol, 1 equiv.), CuTC (19 mg, 0.1 mmol, 0.2 equiv.), and 2,2'-bipyridine (15.6 mg, 0.1 mmol, 0.2 equiv.). The vessel was sealed and placed under a nitrogen atmosphere by cycling onto a Schlenk line. Degassed isobutanol (2.5 mL) and 4-fluoro thiophenol (59 uL, 0.55 mmol, 1.1 equiv.) were added and the mixture was stirred for 24 hours at 55°C. The solvent was evaporated under reduced pressure and the resulting crude was subjected to flash-column chromatography to yield a yellow solid (113 mg, 78% yield as a mixture of 1:0.02 thioether:*Z*-1,2-iodofluoroalkene).

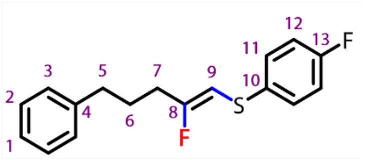

^{1}H NMR (500 MHz, $CDCl_3$): δ 7.36 – 7.27 (4H, m, H^{2+11}), 7.24 – 7.13 (3H, m, H^{1+3}), 7.07 – 6.95 (2H, m, H^{12}), 5.33 (1H, d, J = 33.2 Hz, H^{9}), 2.68 (2H, t, J = 7.6 Hz, H^{5}), 2.33 (dt, J = 17.3, 7.5 Hz, H^{7}), 1.90 (2H, p, J = 7.6 Hz, H^{6}).

^{13}C NMR (126 MHz, $CDCl_3$): δ 162.3 (1C, d, J = 260.6 Hz, C^{8}), 161.8 (1C, d, J = 246.6 Hz, C^{13}), 141.3 (1C, s, C^{4}), 131.1 (2C, d, J = 8.2 Hz, C^{11}), 130.8 (1C, s, C^{10}), 128.47 (1C, s, C^{2}), 128.45 (2C, s, C^{3}), 126.1 (1C, s, C^{1}), 116.2 (2C, d, J = 22.2 Hz, C^{12}), 99.2 (1C, d, J = 17.5 Hz, C^{9}), 34.9 (1C, s, C^{5}), 31.8 (1C, d, J = 25.8 Hz, C^{7}), 27.6 (1C, s, C^{6}).

^{19}F NMR (376 MHz, $CDCl_3$): δ -95.41 (1F, dt, J = 33.1, 17.2 Hz, F^{8}), -112.49 – -119.26 (1F, m, F^{13}). A minor peak was observed at -79.44 (dt, J = 34.7, 16.5 Hz), corresponding to the *Z*-1,2-iodofluoroalkene.

HRMS (EI) calc: [M]+ ($C_{17}H_{16}F_2S$) 290.0935; measured: 290.0930 = 1.72 ppm difference.

IR (neat) v_{max}**/cm^{-1}:** 3675, 3063, 3026, 2924, 2865, 1877, 1658, 1590, 1489, 1453, 1227, 1156, 1136, 1090, 1030, 893, 823, 788, 698, 628, 502, 428.

(Z)-(2-fluoro-2-phenylvinyl)(4-nitrophenyl)sulfane ***(7b)***

To a Schlenk tube equipped with a stirrer bar, was added *(Z)*-(2-fluoro-2-phenylvinyl)(mesityl)iodonium BF_4 (227 mg, 0.5 mmol, 1 equiv.), CuTC (19 mg, 0.1 mmol, 0.2 equiv.), and 2,2'-bipyridine (15.6 mg, 0.1 mmol, 0.2 equiv.). The vessel was sealed and placed under a nitrogen atmosphere by cycling onto a Schlenk line. Degassed isobutanol (2.5 mL) and 4-nitrothiophenol (85 mg, 0.55 mmol, 1.1 equiv.) were added and the mixture was stirred for 24 hours at 55°C. The solvent was evaporated under reduced pressure and the resulting crude was subjected to flash-column chromatography to yield a yellow solid (79 mg, 57% yield).

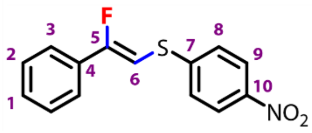

^{1}H NMR (500 MHz, $CDCl_3$): δ 8.18 – 8.15 (2H, m, H^9), 7.63 – 7.58 (2H, m, H^3), 7.48 – 7.45 (2H, m, H^8), 7.45 – 7.42 (3H, m, H^{1+2}), 6.24 (1H, d, J = 31.9 Hz, H^6).

^{13}C NMR (126 MHz, $CDCl_3$): δ 161.3 (1C, d, J = 255.7 Hz, C^5), 145.9 (1C, s, C^7), 145.8 (1C, d, J = 1.5 Hz, C^1), 130.8 (1C, d, J = 27.1 Hz, C^4), 130.3 (1C, s, C^1), 129.0 (2C, d, J = 2.1 Hz, C^2), 127.1 (2C, s, C^8), 124.6 (2C, d, J = 6.8 Hz, C^3), 124.3 (2C, s, C^9), 96.0 (1C, d, J = 19.4 Hz, C^6).

^{19}F NMR (376 MHz, $CDCl_3$): δ -103.25 (1F, d, J = 32.0 Hz, F^5).

HRMS (EI) calc: [M]+ ($C_{14}H_{10}SNO_2F$) 275.0411; measured: 275.0406 = 1.82 ppm difference.

IR (neat) v_{max}**/cm^{-1}:** 3068, 2918, 1626, 1595, 1577, 1510, 1446, 1334, 1282, 1182, 1111, 1037, 1091, 1060, 1037, 853, 841, 809.

(Z)-(2-fluoro-2-phenylvinyl)(4-methoxyphenyl)sulfane **(7c)**

To a Schlenk tube equipped with a stirrer bar, was added *(Z)*-(2-fluoro-2-phenylvinyl)(mesityl)iodonium BF_4 (227 mg, 0.5 mmol, 1 equiv.), CuTC (19 mg, 0.1 mmol, 0.2 equiv.), and 2,2'-bipyridine (15.6 mg, 0.1 mmol, 0.2 equiv.). The vessel was sealed and placed under a nitrogen atmosphere by cycling onto a Schlenk line. Degassed isobutanol (2.5 mL) and 4-methoxythiophenol (67.6 µL, 0.55 mmol, 1.1 equiv.) were added and the mixture was stirred for 24 hours at 55°C. The solvent was evaporated under reduced pressure and the resulting crude was subjected to flash-column chromatography to yield a colourless oil (85 mg, 65% yield).

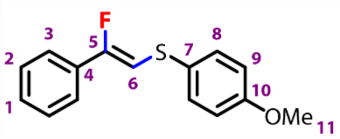

^{1}H NMR (600 MHz, $CDCl_3$): δ 7.48 (2H, d, *J* = 7.6 Hz, *H^3*), 7.44 – 7.41 (2H, m, *H^8*), 7.36 (2H, t, *J* = 7.4 Hz, *H^2*), 7.33 – 7.29 (1H, m, *H^1*), 6.89 (2H, m, *H^9*), 6.13 (1H, d, *J* = 34.8 Hz, *H^6*), 3.82 (3H, s, *H^{11}*).

^{13}C NMR (151 MHz, $CDCl_3$): δ 159.6 (1C, s, *C^{10}*), 155.7 (1C, d, *J* = 247.0 Hz, *C^5*), 132.8 (2C, s, *C^8*), 131.7 (1C, d, *J* = 27.3 Hz, *C^4*), 128.8 (2C, d, *J* = 19.0 Hz, *C^2*), 128.7 (1C, s, *C^1*), 125.5 (2C, s, *C^7*), 123.7 (2C, d, *J* = 6.7 Hz, *C^3*), 115.0 (2C, s, *C^9*), 103.8 (1C, d, *J* = 19.6 Hz, *C^6*), 55.6 (1C, s, *C^{11}*)

^{19}F NMR (376 MHz, $CDCl_3$): δ -103.59 (1F, dt, *J* = 32.7, 17.9 Hz, *F^5*).

HRMS (EI) calc: [M]+ ($C_{15}H_{13}SOF$) 260.0666; measured: 260.0661 = 1.92 ppm difference.

IR (neat) v_{max}/cm^{-1}: 3064, 3002, 2939, 2836, 2043, 1880, 1803, 1708, 1623, 1592, 1572, 1492, 1461, 1441, 1406, 1377, 1313, 1286, 1244, 1173, 1104, 1096, 1075, 1031, 1014, 913, 837**.**

(Z)-(2-fluoro-3,3-dimethylbut-1-en-1-yl)(4-fluorophenyl)sulfane ***(7d)***

To a Schlenk tube equipped with a stirrer bar, was added *(Z)*-(2-fluoro-3,3-dimethylbut-1-en-1-yl)(mesityl)iodonium BF_4 (217 mg, 0.5 mmol, 1 equiv.), CuTC (19 mg, 0.1 mmol, 0.2 equiv.), and 2,2'-bipyridine (15.6 mg, 0.1 mmol, 0.2 equiv.). The vessel was sealed and placed under a nitrogen atmosphere by cycling onto a Schlenk line. Degassed isobutanol (2.5 mL) and 4-fluorothiophenol (59 µL, 0.55 mmol, 1.1 equiv.) were added and the mixture was stirred for 24 hours at 55°C. The solvent was evaporated under reduced pressure and the resulting crude was subjected to flash-column chromatography to yield a colourless oil (75 mg, 66% yield).

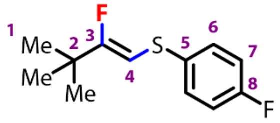

^{1}H NMR (500 MHz, $CDCl_3$): δ 7.39 – 7.30 (2H, m, H^6), 7.08 – 6.95 (2H, m, H^7), 5.37 (1H, d, J = 34.3Hz, H^4), 1.18 (9H, d, J = 0.9 Hz, H^1).

^{13}C NMR (126 MHz, $CDCl_3$): δ 169.0 (1C, d, J = 262.8 Hz, C^3), 161.9 (1C, d, J = 246.5 Hz, C^8), 131.2 (2C, d, J = 7.9 Hz, C^6), 116.3 (2C, d, J = 22.1 Hz, C^7), 96.7 (1C, d, J = 18.7 Hz, C^4), 35.8 (1C, d, J =23.3 Hz, C^2), 27.32 (3C, d, J = 2.4 Hz, C^1).

^{19}F NMR (376 MHz, $CDCl_3$): δ -103.09 (1F, d, J = 34.4 Hz, F^3), -115.48 (1F, tt, J = 8.4, 5.1 Hz, F^8).

HRMS (EI) calc: [M]+ ($C_{12}H_{14}SF_2$) 228.0779; measured: 228.0779 = 0 ppm difference.

IR (neat) ν_{max}**/cm^{-1}:** 3675, 2970, 2908, 1634, 1590, 1489, 1461, 1396, 1365, 1287, 1228, 1156, 1077, 1028, 1013, 874, 822, 789, 767, 630, 546, 509, 426.

Thiourea coupling

(Z)-2-(2-fluorododec-1-en-1-yl)isothiouronium BF$_4$ ***(8a)***

To a Schlenk tube, equipped with a stirrer bar, was added CuTC (38 mg, 0.2 mmol, 0.2 equiv.), 2,2'-bipyrine (38 mg, 0.22 mmol, 0.22 equiv.), *(Z)*-(2-fluorododec-1-en-1-yl)(mesityl)iodonium BF_4 (518 mg, 1 mmol, 1 eq.), and thiourea (84 mg, 1.1 mmol, 1.1 equiv.). The tube was placed under a nitrogen atmosphere, and degassed acetonitrile (2.5 mL) was added. After stirring at toom temperature for ~20 seconds, the tube was placed in a heating block pre-heated to 60 °C. The reaction as stirred at this temperature for 18 hours. The mixture was allowed to cool to room temperature, and the solvent was evaporated under reduced pressure. Water (5 mL) was added, and extracted with DCM (3 x 5 mL). The combined organic extracts were evaporated under reduced pressure and the resulting crude mixture was triturated with pentane (30 mL). Decantation of the pentane and evaporation of the residual solvent under reduced pressure yielded a dark red waxy solid (387 mg, 77% yield based on a purity of 69% with respect to the *Z* isomer, as determined by quantitative ^{19}F NMR assay).

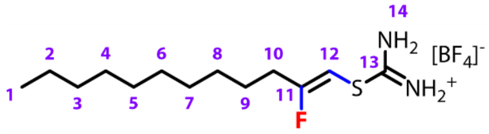

^{1}H NMR (500 MHz, C(CD$_3$)$_2$O): δ 8.72 (4H, brs, H^{14}), 5.88 (1H, d, J = 30.2 Hz, H^{12}), 2.51 (2H, dt, J = 17.5, 7.6 Hz, H^{10}), 1.69 – 1.52 (2H, m, H^9), 0.87 (3H, t, J = 6.9 Hz, H^1).

^{13}C NMR (126 MHz, C(CD$_3$)$_2$O): δ 174.7 (1C, d, J = 274.6 Hz, C^{11}), 171.8 (1C, s, C^{13}), 87.5 (1C, d, J = 15.4 Hz, C^{12}), 33.1 (1C, d, J = 23.5 Hz, C^{10}), 32.6 (6C, s, C^{2-7}) , 26.1 (1C, s, C^9), 23.3 (1C, s, C^{2-7}), 14.3 (1C, s C^1).

^{19}F NMR (376 MHz, C(CD$_3$)$_2$O): δ -79.94 (1F, dt, J = 30.3, 17.4 Hz, F^{11}), 150.11 + 150.16 (*BF$_4$*). A minor peak was observed at -72.48 (td, J = 23.1, 14.4 Hz), which was assigned as the *E*-isomer, in a 1:0.02 *Z:E* ratio.

HRMS (EI) calc: [M – BF_4]+ ($C_{13}H_{26}FN_2S$) 261.1795; measured: 261.1808 = 5.00 ppm difference

IR (neat) v_{max}**/cm^{-1}:** 3426, 3345, 3257, 2923, 2853, 1651, 1467, 1435, 1377, 1283, 1022, 917, 798, 785, 769, 722, 688, 610, 520, 469, 419.

Iodination

(Z)-1-fluoro-4-((2-fluoro-3-iodoallyl)oxy)benzene ***(9b)***

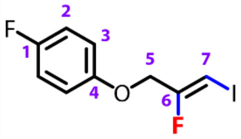

To a glass vial equipped with a stirrer bar, was added the *Z*-FVI (251 mg, 0.5 mmol, 1 equiv.), TBAI (203 mg, 1.1 mmol, 1.1 equiv.), and MeCN (2.5 mL). The mixture was heated to 65 °C. After stirring at this temperature for 18 hours, the reaction mixture was evaporated under reduced pressure, and the resulting crude mixture was subjected to flash-column chromatography (0 to 30% EtOAc in pentane) to afford the product as a colourless oil (96 mg, 65% yield).

1**H NMR (500 MHz, $CDCl_3$):** δ 7.03 – 6.96 (2H, m, H^2), 6.92 – 6.84 (2H, m, H^3), 5.78 (2H, d, *J* = 33.8 Hz, H^7), 4.58 (2H, dd, *J* = 10.0, 0.9 Hz, H^5).

13**C NMR (126 MHz, $CDCl_3$):)** δ 161.1 (1C, d, *J* = 260.8 Hz, C^7), 158.1 (1C, d, *J* = 240.0 Hz, C^1), 153.9 (1C, d, *J* = 2.5 Hz, C^4), 116.4 (2C, d, *J* = 8.1 Hz, C^3), 116.3 (2C, d, *J* = 23.4 Hz, C^2), 66.6 (1C, d, *J* = 35.4, Hz, C^5), 56.6 (1C, d, *J* = 23.2 Hz, C^7).

19**F NMR (376 MHz, $CDCl_3$):** δ -90.22 (1F, dt, *J* = 33.8, 9.9 Hz, F^6), -116.36 – -124.86 (1F, m, F^1).

HRMS (EI) calc: [M]+ (C9H7OF2I) 295.9504; measured: 295.9507 = 1.01 ppm difference.

IR (neat) *v*max/**cm−1:** 3090, 2864, 1663, 1502, 1453, 1379, 1283, 1249, 1197, 1154, 1119, 1097, 1064, 1029, 1007, 917, 895, 871, 825, 778, 767, 730, 711, 510, 483, 458.

(Z)-1-chloro-4-((2-fluoro-3-iodoallyl)oxy)benzene ***(9c)***

To a glass vial equipped with a stirrer bar, was added the *Z*-FVI (259 mg, 0.5 mmol, 1 equiv.), TBAI (203 mg, 0.55 mmol, 1.1 equiv.), and MeCN (5 mL). The mixture was heated to 65 °C. After stirring at this temperature for 18 hours, the reaction mixture was evaporated under reduced pressure, and the resulting crude mixture was subjected to flash-column chromatography (0 to 30% Et_2O in pentane) to afford the product as a white solid (134 mg, 86% yield).

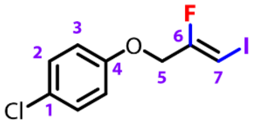

^{1}H NMR (500 MHz, $CDCl_3$): δ 7.28 – 7.24 (2H, m, H^2), 6.87 – 6.83 (2H, m, H^3), 5.79 (1H, d, J = 33.8 Hz, H^7), 4.59 (2H, dd, J = 9.9, 0.9 Hz, H^5).

^{13}C NMR (126 MHz, $CDCl_3$): δ 160.8 (1C, d, J = 260.4 Hz, C^6), 156.4 (1C, s, C^4), 129.7 (1C, s, C^1), 127.1 (2C, s, C^2), 116.4 (2C, s, H^3), 66.0 (1C, d, J = 35.6 Hz, C^7), 56.8 (d, J = 23.3 Hz, C^5).

^{19}F NMR (376 MHz, $CDCl_3$): δ -90.14 (1F, dt, J = 33.8, 9.9 Hz, F^6).

HRMS (EI) calc: $[M]^+$ (C_9H_7OFClI) 311.9209; measured: 311.9203 = 1.92 ppm difference.

IR (neat) v_{max}/cm^{-1}: 3095, 1665, 1583, 1488, 1375, 1273, 1170, 1119, 1086, 1026, 1003, 887, 823, 807, 763, 713, 684, 591, 526, 503, 461

(Z)-1-((2-fluoro-3-iodoallyl)oxy)-4-nitrobenzene ***(9d)***

To a glass vial equipped with a stirrer bar, was added the *Z*-FVI (159 mg, 0.3 mmol, 1 equiv.), TBAI (122 mg, 0.33 mmol, 1.1 equiv.), and MeCN (3 mL). The mixture was heated to 65 °C. After stirring at this temperature for 18 hours, the reaction mixture was evaporated under reduced pressure, and the resulting crude mixture was subjected to flash-column chromatography (0 to 30% Et_2O in pentane) to afford the product as a white solid (71 mg, 73% yield).

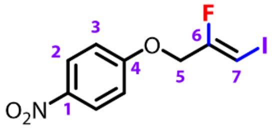

^{1}H NMR (500 MHz, $CDCl_3$): δ 8.25-8.21 (2H, m, H^2), 7.02-6.98 (2H, m, H^3) 5.89 (1H, d, J = 33.4 Hz, H^7), 4.72 (2H, d, J = 10.8 Hz, H^5).

^{13}C NMR (126 MHz, $CDCl_3$): δ 162.5 (1C, s, C^1), 159.9 (1C, d, J = 260.2 Hz, C^6), 142.5 (1C, s, C^4), 126.2 (2C, s, C^3), 114.9 (2C, s, C^2), 65.9 (1C, d, J = 35.3 Hz, C^7), 58.0 (1C, d, J = 23.5 Hz, C^5).

^{19}F NMR (471 MHz, $CDCl_3$): δ -90.13 (1F, dt, J = 33.3, 10.7 Hz, F^6).

HRMS (EI) calc: $[M]^+$ ($C_9H_7NO_3FI$) 322.9449; measured: 322.9445 = 1.24 ppm difference.

IR (neat) v_{max}**/cm^{-1}:** 3102, 2862, 1877, 1609, 1593, 1504, 1452, 1340., 1338, 1262, 1231, 1180, 1063, 916, 862, 844, 820, 771, 752, 690, 658, 528, 499

(Z)-N-(2-fluoro-3-iodoallyl)-N-(4-fluorobenzyl)-4-methylbenzenesulfonamide **(9e)**

In a nitrogen-filled glovebox, to a Schlenk tube equipped with a stirrer bar, was added the *Z*-FVI (669 mg, 1 mmol, 1 equiv.), TBAI (406 mg, 1.1 mmol, 1.1 equiv.), $(CuOTf)_2$.toluene (38.8 mg, 0.075 mmol, 0.15 equiv Cu$^{(I)}$), and 2,2'-bipyridyl (31 mg, 0.2 mmol, 0.2 equiv.). The tube was sealed with a septum, removed from the glovebox, and cycled onto a Schlenk line to maintain the nitrogen atmosphere. Degassed MeCN (12 mL) was added and the mixture was stirred briefly at room temperature before being heated to 60 °C. After stirring at this temperature for 18 hours, the reaction mixture was evaporated under reduced pressure, and the resulting crude mixture was subjected to flash-column chromatography (10 to 20% EtOAc in pentane) to afford the product as a white solid (325 mg, 70% yield).

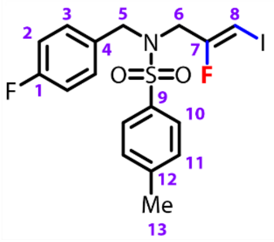

^{1}H NMR (500 MHz, CDCl$_3$): δ 7.72 (2H, d, *J* = 8.2 Hz, H^{10}), 7.34 (2H, d, *J* = 8.0 Hz, H^{11}), 7.28 – 7.23 (2H, m, H^3), 7.02 (2H, t, *J* = 8.6 Hz, H^2), 5.40 (1H, d, *J* = 33.5 Hz, H^8), 4.34 (2H, s, H^5), 3.95 (2H, d, *J* = 15.8 Hz, H^6), 2.45 (3H, s, H^{13}).

^{13}C NMR (126 MHz, CDCl$_3$):) δ 162.7 (1C, d, *J* = 247.0 Hz, C^1), 160.6 (1C, d, *J* = 264.9 Hz, C^7), 144.0 (1C, s, C^{12}), 136.8 (1C, s, C^9), 130.9 (d, *J* = 3.2 Hz, C^4), 130.4 (2C, d, *J* = 8.2 Hz, C^3), 129.9 (2C, s, C^{11}), 127.5 (2C, s, C^{10}), 115.9 (2C, d, *J* = 21.6 Hz, C^2), 57.5 (1C, d, *J* = 24.1 Hz, H^8), 50.6 (1C, s, C^5), 46.5 (1C, d, *J* = 29.2 Hz, C^6), 21.7 (1C, s, C^{13}).

^{19}F NMR (376 MHz, CDCl$_3$): δ -86.19 (1F, dt, *J* = 32.6, 16.0 Hz, F^7), -113.66 (1F, td, *J* = 8.8, 4.6 Hz, F^1)

HRMS (APCI) calc: $[M+H]^+$ ($C_{17}H_{16}NO_2F_2SI$) 463.9987; measured: 463.9982 = 1.08 ppm difference.

IR (neat) v_{max}**/cm^{-1}:** 3679, 2989, 2901, 1663, 1600, 1508, 1334, 1225, 1160, 1086, 1066.

(Z)-N-(2-fluoro-3-iodoallyl)-4-methyl-N-(4-methylbenzyl)benzenesulfonamide ***(9f)***

To a glass vial equipped with a stirrer bar, was added the *Z*-FVI (332 mg, 0.5 mmol, 1 equiv.), TBAI (203 mg, 0.55 mmol, 1.1 equiv.), and MeCN (5 mL). The mixture was heated to 65 °C. After stirring at this temperature for 18 hours, the reaction mixture was evaporated under reduced pressure, and the resulting crude mixture was subjected to flash-column chromatography (0 to 40% Et_2O in pentane) to afford the product as a white solid (184 mg, 80% yield).

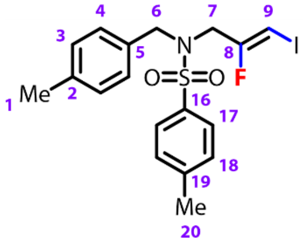

^{1}H NMR (500 MHz, $CDCl_3$): δ 7.75 – 7.71 (2H, m, H^{17}), 7.35 – 7.32 (2H, m, H^{18}), 7.16 – 7.11 (4H, m, H^{3+4}), 5.37 (1H, d, J = 33.6 Hz, H^9), 4.34 (2H, s, H^6), 3.95 (2H, d, J = 15.6 Hz, H^7), 2.45 (3H, s, H^{20}), 2.33 (3H, s, H^1).

^{13}C NMR (126 MHz, $CDCl_3$): δ 160.7 (1C, d, J = 264.9 Hz, C^8), 143.7 (1C, s, C^{19}), 138.1 (1C, s, C^2), 136.9 (1C, s, C^{16}), 131.9 (1C, s, C^5), 129.8 (2C, s, C^{18}), 129.5 (2C, s, C^{3+4}), 128.7 (2C, s, C^{3+4}), 127.4 (2C, s, C^{17}), 57.2 (1C, d, J = 24.0 Hz, C^9), 50.9 (1C, s, C^6), 46.2 (1C, d, J = 29.5 Hz, C^7), 21.7 (1C, s, C^{20}), 21.2 (1C, s, C^1).

^{19}F NMR (471 MHz, $CDCl_3$): δ -86.30 (1F, dt, J = 33.5, 15.6 Hz, F^8).

HRMS (Nanospray) calc: $[M+Na]^+$ ($C_{18}H_{19}NO_2FNaIS$) 482.0063 ; measured: 482.0049 = 2.9 ppm difference.

IR (neat) ν_{max}**/cm^{-1}:** 3088, 2921, 2864, 1657, 1598, 1514, 1495, 1444, 1333, 1289, 1156, 1118, 1090, 1067, 1019, 937, 910, 868, 842, 747, 769, 706, 577, 546, 513, 476, 706

(Z)-N-(2-fluoro-3-iodoallyl)-N-(4-fluorophenyl)-4-methylbenzenesulfonamide ***(9h)***

To a glass vial equipped with a stirrer bar, was added the *Z*-FVI (177 mg, 0.27 mmol, 1 equiv.), TBAI (122 mg, 0.33 mmol, 1.1 equiv.), and MeCN (5 mL). The mixture was heated to 65 °C. After stirring at this temperature for 18 hours, the reaction mixture was evaporated under reduced pressure, and the resulting crude mixture was subjected to flash-column chromatography (0 to 30% Et_2O in pentane) to afford the product as a white solid (90 mg, 74% yield).

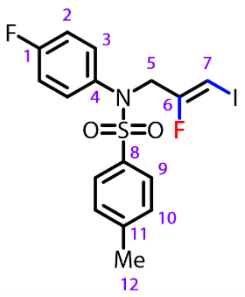

^{1}H NMR (500 MHz, $CDCl_3$): δ 7.51 – 7.44 (2H, m, H^9), 7.26 (2H, d, J = 7.9 Hz, H^{10}), 7.06 – 6.93 (m, H^{2+3}), 5.53 (1H, d, J = 33.2 Hz, H^7), 4.37 (2H, dd, J = 13.9, 0.8 Hz, H^5), 2.42 (3H, s, H^{12}).

^{13}C NMR (126 MHz, $CDCl_3$): δ 162.3 (1C, d, J = 249.2 Hz, C^1), 160.4 (1C, d, J = 262.7 Hz, C^6), 144.2 (1C, s, C^{11}), 135.4 (1C, s, C^8), 134.6 (d, J = 3.2 Hz, C^4), 131.2 (d, J = 8.8 Hz, C^3), 129.7 (2C, s, C^{10}), 127.8 (2C, s, C^9), 116.4 (d, J = 22.8 Hz, C^2), 57.5 (d, J = 24.4 Hz, C^7), 52.0 (1C, d, J = 31.2 Hz, C^5), 21.7 (1C, s, C^{12}).

^{19}F NMR (376 MHz, $CDCl_3$): δ -87.01 (1F, dt, J = 33.1, 13.8 Hz, F^6), -111.69 (1F, dt, J = 12.8, 7.2 Hz, F^1).

HRMS (EI) calc: $[M]^+$ ($C_{16}H_{14}NO_2F_2IS$) 448.9753 ; measured: 448.9743 = 2.23 ppm difference.

IR (neat) v_{max}/cm^{-1}: 3089, 2923, 1659, 1598, 1505, 1347, 1210, 1236, 1210, 1150, 1123, 1090, 1013, 876, 874, 810, 768, 731, 707, 665, 516, 548, 492, 445

(Z)-(((3-fluoro-4-iodobut-3-en-1-yl)oxy)methyl)benzene ***(9i)***

To a glass vial equipped with a stirrer bar, was added the *Z*-FVI (256 mg, 0.5 mmol, 1 equiv.), TBAI (203 mg, 0.5 mmol, 1.1 equiv.), and MeCN (3 mL). The mixture was heated to 65 °C. After stirring at this temperature for 18 hours, the reaction mixture was evaporated under reduced pressure, and the resulting crude mixture was subjected to flash-column chromatography (0 to 20% Et_2O in pentane) to afford the product as a colourless oil (84 mg, 55% yield).

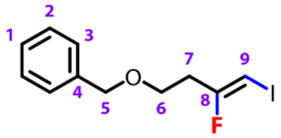

^{1}H NMR (500 MHz, $CDCl_3$): δ 7.38 – 7.28 (5H, m, H^{1-3}), 5.33 (1H, dt, J = 34.8, 0.9 Hz, H^9), 4.53 (2H, s, H^5), 3.63 (2H, t, J = 6.4 Hz, H^6), 2.65 (2H, dtd, J = 16.1, 6.4, 0.9 Hz, H^7).

^{13}C NMR (126 MHz, $CDCl_3$): δ 163.8 (1C, d, J = 260.5 Hz, C^8), 138.0 (1C, s, C^4), 128.6 (2C, s, C^2), 127.9 (1C, s, C^1), 127.8 (2C, s, C^3), 73.3 (1C, s, C^5), 65.9 (1C, s, C^6), 52.8 (d, J = 25.8 Hz, C^9), 33.7 (1C, d, J = 27.2 Hz, C^7).

^{19}F NMR (471 MHz, $CDCl_3$): δ -80.11 (1F, dt, J = 34.7, 15.9 Hz, F^8).

HRMS (Nanospray) calc: $[M+Na]^+$ ($C_{11}H_{12}OFNaI$) 328.9815; measured: 328.9807 = 2.4 ppm difference.

IR (neat) v_{max}/cm^{-1}: 3089, 3030, 2863, 1657, 1495, 1454, 1416, 1362, 1255, 1199, 1099, 1028, 908, 877, 831, 735, 697, 643, 606, 525, 484, 455

(Z)-3-fluoro-4-iodobut-3-en-1-yl 4-methylbenzenesulfonate ***(9j)***

To a glass vial equipped with a stirrer bar, was added the *Z*-FVI (288 mg, 0.5 mmol, 1 equiv.), TBAI (203 mg, 0.55 mmol, 1.1 equiv.), and MeCN (3 mL). The mixture was heated to 45 °C for 18 hours. After stirring at this temperature for 18 hours, the reaction mixture was evaporated under reduced pressure, and the resulting crude mixture was subjected to flash-column chromatography (0 to 30% Et_2O in pentane) to afford the product as a colourless oil (120 mg, 65% yield).

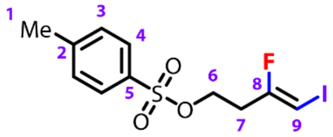

^{1}H NMR (600 MHz, $CDCl_3$): δ 7.76 (2H, d, *J* = 8.3 Hz, *H^4*), 7.36 (2H, d, *J* = 8.3 Hz, *H^3*), 5.33 (1H, d, *J* = 34.3 Hz, *H^9*), 4.14 (td, *J* = 6.2, 1.5 Hz, *H^6*), 2.68 (2H, dt, *J* = 16.2, 6.3 Hz, *H^7*).

^{13}C NMR (151 MHz, $CDCl_3$): δ 161.1 (1C, d, *J* = 260.0 Hz, *C^8*), 145.3 (1C, s, *C^2*), 132.6 (1C, s, *C^5*), 130.1 (2C, s, *C^3*), 128.0 (2C, s, *C^4*), 65.3 (1C, s, *C^9*), 54.6 (1C, d, *J* = 25.4 Hz, *C^6*), 32.8 (1C, d, *J* = 28.2 Hz, *C^7*), 21.8 (1C, s, *C^1*).

^{19}F NMR (376 MHz, $CDCl_3$): δ -82.61 (1F, dt, *J* = 33.4, 16.3 Hz, *F^8*).

HRMS (ESI) calc: $[M+Na]^+$ ($C_{11}H_{12}O_3NaSIF$) 392.9434; measured: 392.9429 = 1.3 ppm difference.

IR (neat) v_{max}**/cm^{-1}:** 3016, 2970, ,1738, 1435, 1366, 1229, 1217, 1092, 897, 772, 528

(Z)-N-(2-fluoro-3-iodoallyl)-4-methylbenzenesulfonamide (9k)

To a glass vial equipped with a stirrer bar, was added the *Z*-FVI (281 mg, 0.5 mmol, 1 equiv.), TBAI (203 mg, 0.55 mmol, 1.1 equiv.), and MeCN (3 mL). The mixture was heated to 70 °C. After stirring at this temperature for 18 hours, the reaction mixture was evaporated under reduced pressure, and the resulting crude mixture was subjected to flash-column chromatography (25% EtOAc in pentane) to afford the product as a white solid (mg, % yield).

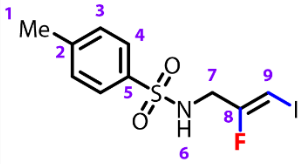

^{1}H NMR (400 MHz, CDCl$_3$): δ 7.72 (2H, d, *J* = 8.3 Hz, *H*^{4}), 7.32 (2H, d, *J* = 8.0 Hz, *H*^{3}), 5.49 (1H, d, *J* = 33.7, *H*^{9}), 4.90 (1H, t, *J* = 6.5 Hz, *H*^{6}), 3.85 (2H, ddd, *J* = 12.8, 6.5, 0.8 Hz, *H*^{7}), 2.44 (3H, s, *H*^{1}).

^{13}C NMR (126 MHz, CDCl$_3$): δ 160.3 (1C, d, *J* = 260.4 Hz*C*^{8}), 144.2 (1C, s, *C*^{5}), 136.9 (1C, s, *C*^{2}), 130.0 (2C, s, *C*^{3}), 127.3 (2C, s, *C*^{4}), 56.1 (1C, d, *J* = 24.2 Hz, *C*^{9}), 43.8 (1C, d, *J* = 32.3 Hz, *C*^{7}), 21.7 (1C, s, *C*^{1}).

^{19}F NMR (376 MHz, CDCl$_3$): δ -88.81 (1F, dt, *J* = 33.7, 12.5 Hz, *F*^{8}).

HRMS (ESI) calc: $[M-I]^+$ ($C_{10}H_{11}NO_2FSI$) 228.0489; measured: 228.0488 = 0.44 ppm difference.

IR (neat) ν_{max}**/cm^{-1}:** 3260 1664, 1600, 1440, 1322, 1156, 1125, 1095, 1056, 873, 808, 670, 547, 507.

(Z)-4-(1-fluoro-2-iodovinyl)benzaldehyde ***(9l)***

To a glass vial equipped with a stirrer bar, was added the *Z*-FVI (193 mg, 0.4 mmol, 1 equiv.), TBAI (162 mg, 0.44 mmol, 1.1 equiv.), and MeCN (4 mL). The mixture was heated to 65 °C. After stirring at this temperature for 18 hours, the reaction mixture was evaporated under reduced pressure, and the resulting crude mixture was subjected to flash-column chromatography (0 to 30% Et_2O in pentane) to afford the product as a white solid (86 mg, 78% yield).

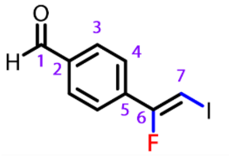

^{1}H NMR (500 MHz, $CDCl_3$): δ 10.04 (1H, s, *H^1*), 7.92 – 7.89 (2H, m, *H^3*), 7.70 – 7.67 (2H, m, *H^4*), 6.39 (1H, d, *J* = 34.0 Hz, *H^7*).

^{13}C NMR (126 MHz, $CDCl_3$): δ 191.4 (1C, s, *C^1*), 162.1 (1C, d, *J* = 252.1 Hz, *C^6*), 137.0 (1C, s, *C^2*), 136.0 (1C, d, *J* = 28.8 Hz, *C^5*), 130.2 (2C, d, *J* = 2.0 Hz, *C^3*), 125.2 (2C, d, *J* = 6.1 Hz, *C^4*), 57.8 (1C, d, *J* = 28.6 Hz, *C^5*).

^{19}F NMR (376 MHz, $CDCl_3$): δ -90.74 (1F, d, *J* = 34.1 Hz, *F^6*).

HRMS (EI) calc: $[M]^+$ (C_9H_6OFI) 275.9442 ; measured: 275.9427 = 1.81 ppm difference.

IR (neat) ν_{max}**/cm^{-1}:** 3081, 2836, 2743, 1696, 1677, 1602, 1417, 1385, 1307, 1275, 1215, 1171, 1007, 1019, 845, 829, 758, 702, 610, 463

(Z)-2-(2-fluoro-3-iodoallyl)isoindoline-1,3-dione **(9m)**

To a glass vial equipped with a stirrer bar, was added the *Z*-FVI (269 mg, 0.5 mmol, 1 equiv.), TBAI (203 mg, 0.55 mmol, 1.1 equiv.), and MeCN (3 mL). The mixture was heated to 70 °C. After stirring at this temperature for 18 hours, the reaction mixture was evaporated under reduced pressure, and the resulting crude mixture was subjected to flash-column chromatography (25% EtOAc in pentane) to afford the product as a white solid (129 mg, 78% yield).

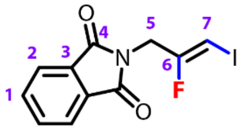

^{1}H NMR (400 MHz, $CDCl_3$): δ 7.93 – 7.84 (2H, m, H^1), 7.81 – 7.71 (2H, m, H^2), 5.73 (1H, d, J = 33.2 Hz, H^7), 4.50 (2H, dd, J = 13.2, 0.8 Hz, H^5).

^{13}C NMR (126 MHz, $C(CD_3)_2O$): δ 167.9 (2C, s, C^4), 161.1 (d, J = 260.2 Hz, C^6), 135.4 (2C, s, C^2), 133.0 (2C, s, C^3), 124.1 (2C, s, C^1), 56.4 (d, J = 22.7 Hz, C^7), 38.6 (d, J = 34.6 Hz, C^5).

^{19}F NMR (376 MHz, $C(CD_3)_2O$): δ -89.60 (1F, dt, J = 35.1, 11.6 Hz, F^4).

HRMS (ESI) calc: $[M-I]^+$ ($C_{11}H_7NO_2F$) 204.0455; measured: 204.0455 = 0 ppm difference.

IR (neat) v_{max}/cm^{-1}: 3093, 2921, 1775, 1705, 1668, 1469, 1415, 1394, 1311, 1227, 1196, 1119, 1106, 942, 897, 799, 783, 738, 716, 626, 559, 530, 476, 415.

(Z)-1,3,5-trichloro-2-((2-fluoro-3-iodoallyl)oxy)benzene ***(9n)***

To a glass vial equipped with a stirrer bar, was added the *Z*-FVI (294 mg, 0.5 mmol, 1 equiv.), TBAI (203 mg, 0.55 mmol, 1.1 equiv.), and MeCN (5 mL). The mixture was heated to 65 °C. After stirring at this temperature for 18 hours, the reaction mixture was evaporated under reduced pressure, and the resulting crude mixture was subjected to flash-column chromatography (0 to 30 % Et_2O in pentane) to afford the product as a white solid (144 mg, 76% yield).

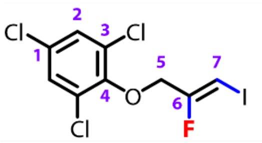

^{1}H NMR (500 MHz, $CDCl_3$): δ 7.32 (2H, s, H^2), 5.89 (1H, dd, J = 32.9, 0.7 Hz, H^7), 4.62 (2H, dd, J = 13.9, 0.6 Hz, H^5).

^{13}C NMR (126 MHz, $CDCl_3$): δ 160.7 (1C, d, J = 261.1 Hz, C^6), 149.3 (1C, s, C^4), 130.6 (1C, s, C^1), 130.2 (2C, s, C^3), 129.1 (2C, s, C^2), 70.0 (1C, d, J = 33.6 Hz, C^7), 58.9 (1C, d, J = 23.6 Hz, C^5).

^{19}F NMR (471 MHz, $CDCl_3$): δ -88.87 (1F, dt, J = 32.9, 13.8 Hz, F^6).

HRMS (Nanospray) calc: $[M+Na]^+$ ($C_9H_5OCl_3NaIF$) 402.8332; measured: 402.8329 = 0.7 ppm difference.

IR (neat) ν_{max}**/cm^{-1}:** 3083, 1662, 1553, 1446, 1429, 1377, 1278, 1253, 1190, 1135, 1122, 967, 895, 817, 801, 779, 766, 721, 618, 576, 509, 425.

(Z)-1,3-dibromo-5-((2-fluoro-3-iodoallyl)oxy)benzene ***(9o)***

To a glass vial equipped with a stirrer bar, was added the *Z*-FVI (71 mg, 0.11 mmol, 1 equiv.), TBAI (41 mg, 0.11 mmol, 1.0 equiv.), and MeCN (1 mL). The mixture was heated to 65 °C. After stirring at this temperature for 18 hours, the reaction mixture was evaporated under reduced pressure, and the resulting crude mixture was subjected to flash-column chromatography (0 to 30% Et_2O in pentane) to afford the product as a white solid (33 mg, 69% yield).

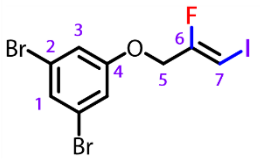

^{1}H NMR (500 MHz, $CDCl_3$): δ 7.32 (1H, t, *J* = 1.6 Hz, H^1), 7.02 (2H, d, *J* = 1.7 Hz, H^3), 5.83 (1H, d, *J* = 33.5 Hz, H^7), 4.58 (2H, dd, *J* = 10.5, 0.9 Hz, H^5).

^{13}C NMR (126 MHz, $CDCl_3$): δ 160.1 (1C, d, *J* = 260.2 Hz, C^5), 158.8 (1C, s, C^4), 127.9 (1C, s, C^1), 123.5 (2C, s, C^2), 117.4 (2C, s, C^3), 66.0 (d, *J* = 35.3 Hz, C^5), 57.5 (d, *J* = 23.4 Hz, C^7).

^{19}F NMR (471 MHz, $CDCl_3$): δ -90.14 (1F, dt, *J* = 33.5, 10.5 Hz, F^6).

HRMS (EI) calc: $[M]^+$ ($C_9H_6OFBr_2I$) 433.7809 ; measured: 433.7803 = 1.38 ppm difference.

IR (neat) v_{max}**/cm^{-1}:** 3083, 2922, 2863, 1662, 1581, 1559, 1435, 1418, 1374, 1297, 1254, 1218, 1108, 1025, 1005, 906, 888, 766, 744, 665, 505, 465.

Iodoalkyne Hydrofluorination Control Experiment

Synthesis of (iodoethynyl)benzene

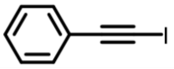

This substrate was synthesised according to an adapted literature procedure.[39]

To a round-bottom flask wrapped in foil and equipped with a stir bar, under air, was added NIS (1.24 g, 5.5 mmol, 1.1 equiv), $AgNO_3$ (170 mg, 1.0 mmol, 20 mol %), acetone (70 mL), and phenylacetylene (550 µL, 5 mmol, 1 equiv.). The mixture was stirred for 18 hours before being quenched with saturated NaHCO3(aq). The mixture was extracted with DCM (2× 40 mL). The combined organic extracts were filtered through cotton. The filtrate was evaporated under reduced pressure and subjected to flash-column chromatography to afford the product as an orange oil (683 g, 30% yield).

^{1}H NMR (500 MHz, $CDCl_3$): δ 7.48 – 7.38 (2H, m), 7.36 – 7.29 (3H, m).

^{13}C NMR (126 MHz, $CDCl_3$): δ 132.5, 128.9, 128.4, 123.5, 94.3, 6.3.

These data are consistent with those previously reported.[40]

(iodoethynyl)benzene Hydrofluorination Test

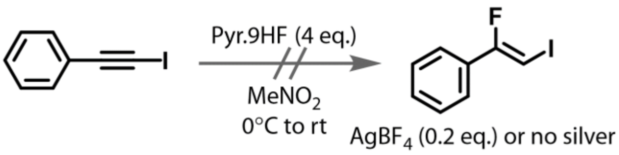

An HDPE vial equipped ,with a stirrer bar, was taken into a nitrogen-filled glovebox. $AgBF_4$ (3.9 mg, 0.02 mmol, 0.2 equiv.) was added. The vial was removed from the glovebox, and a solution of (iodoethynyl)benzene (22.8 mg, 0.1 mmol, 1 equiv.) in $MeNO_2$ (700 µL) was added. The mixture was then cooled to 0°C before adding pyr.9HF (93 µL). The mixture was allowed to warm to room temperature and left to stir for 18 hours. Hexafluorobenzene was added, and an aliquot of the reaction mixture was analysed by ^{19}F NMR to determine the NMR yield. The same procedure was carried immediately after but without the addition of silver. In both cases, no hydrofluorination was observed.

Hydride Displacement

N-(2-fluoroallyl)-N-(4-fluorobenzyl)-4-methylbenzenesulfonamide ***(10a)***

To a glass vial equipped with a stirrer bar, was added (Z)-(2-fluoro-3-((4-methyl-N-(4methylbenzyl)phenyl)sulfonamido)prop-1-en-1-yl)(mesityl)iodonium BF_4 (335 mg, 0.5 mmol, 1 equiv.) and methanol (5 mL). The solution was cooled to 0 °C and sodium borohydride (38 mg, 1 mmol, 2 equiv.) was added. The mixture was allowed to stir for 5 hours before adding water (5 mL) and DCM (10 mL). The aqueous and organic layers were separated, and the aqueous phase was extracted with DCM (3 x). The combined organic extracts were filtered and evaporated under reduced pressure. The resulting crude was subjected to flash-column chromatography (0 to 25% EtOAc in pentane) to afford the product as a white solid (130 mg, 77%).

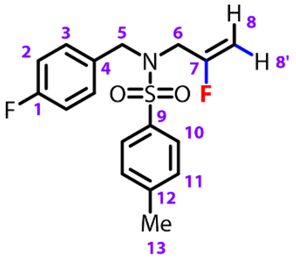

^{1}H NMR (500 MHz, $CDCl_3$): δ 7.76 (2H, d, *J* = 8.3 Hz, H^{10}), 7.33 (2H, d, *J* = 8.2 Hz, H^{11}), 7.31 – 7.27 (2H, m, H^3), 7.02 (2H,j t, *J* = 8.6 Hz, H2), 4.65 (1H, dd, *J* = 16.2, 3.3 Hz, H8′), 4.38 (2H, s, H5), 4.32 (1H, dd, *J* = 47.9, 3.4 Hz, H8), 3.89 (2H, d, *J* = 15.9 Hz, H6), 2.46 (3H, s, H13).

^{13}C NMR (126 MHz, $CDCl_3$): δ 162.6 (1C, d, *J* = 246.5 Hz, C^1), 160.3 (1C, d, *J* = 262.0 Hz, C^7), 143.7 (1C, s, C^{12}), 137.2 (1C, s, C^9), 131.2 (1C, d, *J* = 3.2 Hz, C^4), 130.4 (2C, d, *J* = 8.2 Hz, C^3), 129.7 (2C, s, C^{11}), 127.4 (2C, s, *J* = 1.0 Hz, C^{10}), 115.7 (2C, d, *J* = 21.5 Hz, C^2), 94.8 (d, *J* = 17.4 Hz, C^8), 49.9 (1C, s, C^5), 46.3 (1C, d, *J* = 30.5 Hz, C^6), 21.6 (1C, s, C^{13}).

^{19}F NMR (376 MHz, $CDCl_3$): δ -101.29 (1F, dq, *J* = 47.8, 16.0 Hz, F^1), -114.06 (1F, tt, *J* = 8.6, 5.3 Hz, F^7).

HRMS (ESI) calc: $[M+H]^+$ ($C_{17}H_{17}NO_2SF_2$) 338.1021; measured: 338.1017 = 1.18 ppm difference.

IR (neat) v_{max}**/cm^{-1}:** 2925, 1678, 1608, 1509, 1445, 1395, 1360, 1339, 1325, 1310, 1287, 1237, 1224, 1189, 1157, 1122, 1092, 1015, 970, 936, 905, 889, 852, 816, 789, 753, 707, 667, 636, 608, 574, 549, 505, 479, 415.

Sonogashira Coupling

(Z)-3-fluoro-2-methyl-6-phenylhex-3-en-5-yn-2-ol ***(11a)***

To a Schlenk tube equipped with a stirrer bar, was added [Pd(OAc)$_2$] (5.6 mg, 0.025 mmol, 0.05 equiv.), triphenylphosphine (7.9 mg, 0.03 mmol, 0.06 equiv.), CuI (7.6 mg, 0.04 mmol, 0.08 equiv.), and the *Z*-FVI (218 mg, 0.5 mmol, 1 equiv.). The tube was placed under vacuum and back-filled with nitrogen for three cycles. Degassed MeCN (2.5 mL), NEt$_3$ (140 *µ*L, 1 mmol, 2 equiv.), and phenylacetylene (110 *µ*L, 1 mmol, 2 equiv.) were added in short succession. The mixture was stirred for 45 minutes, and a saturated aqueous solution of ammonium chloride was added. The aqueous and organic layers were separated, and the aqueous phase was extracted with DCM (3 x). The combined organic layers were washed with water (2 x) and brine (3 x), then evaporation under reduced pressure. The resulting crude mixture was subjected to flash-column chromatography (0 to 50% Et$_2$O in pentane) to afford the product as an orange solid (84 mg, 82% yield).

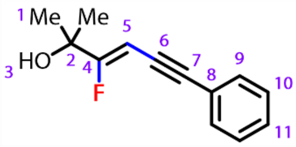

^{1}H NMR (500 MHz, CDCl$_3$): δ 7.49 – 7.43 (2H, m, *H^9*), 7.36 – 7.31 (3H, m, *H^{10+11}*), 5.48 (1H, d, *J* = 33.4 Hz, *H^5*), 2.15 (1H, brs, *H^3*), 1.49 (6H, d, *J* = 1.4 Hz, *H^1*).

^{13}C NMR (126 MHz, CDCl$_3$): δ 172.4 (1C, d, *J* = 276.5 Hz, *C^4*), 131.6 (1C, s, *C^9*), 128.4 (3C, s, *C^{10+11}*), 123.3 (1C, s, *C^8*), 94.4 (1C, d, *J* = 6.1 Hz, *C^6*), 86.0 (1C, d, *J* = 13.0 Hz, *C^5*), 81.2 (1C, d, *J* = 3.5 Hz, *C^7*), 70.8 (1C, d, *J* = 27.5 Hz, *C^2*), 27.4 (2C, s, *C^1*).

^{19}F NMR (471 MHz, CDCl$_3$): δ -102.32 (1F, d, *J* = 33.5 Hz, *F^4*).

HRMS (EI) calc: [M]$^+$ ($C_{13}H_{13}OF$); 204.0945 ; measured: 204.0941 = 1.96 ppm difference.

IR (neat) v_{max}**/cm^{-1}:** 3383, 3066, 2983, 2937, 1738, 1664, 1597, 1490, 1442, 1365, 1307, 1230, 1191, 1173, 1089, 1032, 957, 879, 807, 754, 689, 593, 528, 440

(Z)-6-fluoro-6-(4-fluorophenyl)-2-methylhex-5-en-3-yn-2-ol ***(11b)***

To a Schlenk tube equipped with a stirrer bar, was added [Pd(OAc)$_2$] (11.2 mg, 0.05 mmol, 0.05 equiv.), triphenylphosphine (15.7 mg, 0.06 mmol, 0.06 equiv.), CuI (15.2 mg, 0.08 mmol, 0.08 equiv.), and *(Z)-(*2-fluoro-2-(4-fluorophenyl)vinyl)(mesityl)iodonium BF_4 (472 mg, 1 mmol, 1 equiv.). The tube was placed under vacuum and back-filled with nitrogen for three cycles. Degassed MeCN (5 mL), NEt_3 (280 μL, 2 mmol, 2 equiv.), and 2-methyl-3-butyn-2-ol (194 μL, 2 mmol, 2 equiv.) were added in short succession. The mixture was stirred for 45 minutes and a saturated aqueous solution of ammonium chloride was added. The aqueous and organic layers were separated, and the aqueous phase was extracted with DCM (3 x). The combined organic layers were evaporated under reduced pressure, and the resulting crude mixture was subjected to flash-column chromatography (5 to 20% EtOAc in pentane) to afford the product as a colourless oil (169 mg, 76% yield).

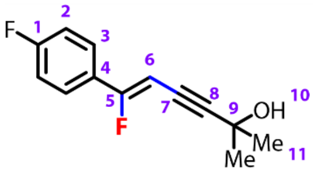

^{1}H NMR (500 MHz, CDCl$_3$): δ 7.54 – 7.49 (2H, m, H^3), 7.08 (t, J = 8.6 Hz, H^2), 5.51 (1H, d, J = 33.2 Hz, H^6), 2.02 (1H, brs, H^{10}), 1.60 (6H, s, H^{11}).

^{13}C NMR (126 MHz, CDCl$_3$): δ 164.2 (1C, d, J = 261.3 Hz, C^5), 163.9 (1C, d, J = 251.2 Hz, C^1), 127.4 (dd, J = 27.2, 3.4 Hz, C^4), 126.4 (dd, J = 8.5, 7.1 Hz, C^3), 116.1 (dd, J = 22.2, 2.1 Hz, C^2), 100.8 (1C, d, J = 6.3 Hz, C^7), 86.5 (1C, dd, J = 16.9, 2.1 Hz, C^6), 74.9 (1C, d, J = 3.2 Hz, C^8), 66.0 (1C, s, C^9), 31.5 (2C, s, C^{11}).

^{19}F NMR (376 MHz, CDCl$_3$): δ -102.46 (1F, d, J = 33.0 Hz, F^5), -109.71 (1F, m, F^1).

HRMS (ESI) calc: $[M+H-H_2O]^+$ ($C_{13}H_{12}OF_2$) 205.0823; measured: 205.0824 = 0.49 ppm difference.

IR (neat) ν_{max}**/cm^{-1}:** 3395, 2979, 2927, 1683, 1600, 1509, 1233, 1159, 839, 511..

(Z)-(4-fluoro-5-methoxypent-3-en-1-yn-1-yl)benzene ***(11c)***

To a Schlenk tube equipped with a stirrer bar, was added [$Pd(OAc)_2$] (5.4 mg, 0.025 mmol, 0.05 equiv.), triphenylphosphine (7.9 mg, 0.03 mmol, 0.06 equiv.), CuI (7.6 mg, 0.04 mmol, 0.08 equiv.), and the *Z*-FVI (211 mg, 0.5 mmol, 1 equiv.). The tube was placed under vacuum and back-filled with nitrogen for three cycles. Degassed DMF (2.5 mL), NEt_3 (140 μL, 1 mmol, 2 equiv.), and phenylacetylene (110 μL, 1 mmol, 2 equiv.) were added in short succession. The mixture was stirred for 45 minutes, and a saturated aqueous solution of ammonium chloride was added. The aqueous and organic layers were separated, and the aqueous phase was extracted with DCM (3 x). The combined organic layers were washed with water (2 x) and brine (3 x), then evaporation under reduced pressure. The resulting crude mixture was subjected to flash-column chromatography (5 to 20% EtOAc in pentane) to afford the product as an orange solid (68 mg, 71% yield).

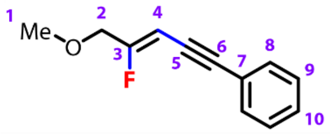

^{1}H NMR (500 MHz, $CDCl_3$): δ 7.49 – 7.44 (2H, m, H^8), 7.34 – 7.30 (3H, m, H^{9+10}), 5.33 (1H, d, *J* = 32.7, H^4), 4.03 (2H, d, *J* = 12.4 Hz, H^2), 3.42 (3H, s, H^1).

^{13}C NMR (126 MHz, $CDCl_3$): δ 164.4 (1C, d, *J* = 273.7 Hz, C^3), 131.7 (2C, s, C^8), 128.6 (1C, s, C^{10}),128.4 (2C, s, C^9), 123.1 (1C, s, C^7), 94.9 (1C, d, *J* = 5.9 Hz, C^6), 90.7 (1C, d, *J* = 11.8 Hz, C^5), 80.7 (1C, s, C^4), 69.8 (1C, d, *J* = 30.1 Hz, C^2), 58.7 (1C, s, C^1).

^{19}F NMR (376 MHz, $C(CD_3)_2O$): δ -99.14 (1F, dt, *J* = 32.7, 12.3 Hz, F^3).

HRMS (EI) calc: $[M]^+$ ($C_{12}H_{11}OF$) 190.0788; measured: 190.0787 = 0.53 ppm difference.

IR (neat) ν_{max}**/cm^{-1}:** 2927, 2206, 1597, 1490, 1443, 1385, 1340, 1187, 1102, 979, 914, 873, 816, 757, 691, 528.

(Z)-N-(2-fluoro-5-phenylpent-2-en-4-yn-1-yl)-N-(4-fluorobenzyl)-4-methylbenzenesulfonamide ***(11d)***

To a Schlenk tube equipped with a stirrer bar, was added [Pd(OAc)$_2$] (5.4 mg, 0.025 mmol, 0.05 equiv.), triphenylphosphine (7.9 mg, 0.03 mmol, 0.06 equiv.), CuI (7.6 mg, 0.04 mmol, 0.08 equiv.), and the *Z*-FVI (335 mg, 0.5 mmol, 1 equiv.). The tube was placed under vacuum and back-filled with nitrogen for three cycles. Degassed DMF (2.5 mL), NEt$_3$ (140 μL, 1 mmol, 2 equiv.), and phenylacetylene (110 μL, 1 mmol, 2 equiv.) were added in short succession. The mixture was stirred for 45 minutes, and a saturated aqueous solution of ammonium chloride was added. The aqueous and organic layers were separated, and the aqueous phase was extracted with DCM (3 x). The combined organic layers were evaporated under reduced pressure, and the resulting crude mixture was subjected to flash-column chromatography (5 to 20% EtOAc in pentane) to afford the product as an orange solid (170 mg, 78% yield).

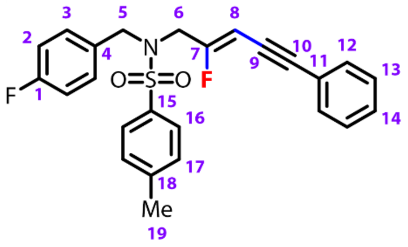

^{1}H NMR (500 MHz, CDCl$_3$): δ 7.78 – 7.75 (2H, m, H^{16}), 7.44 – 7.40 (2H, m, J = 7.8 Hz, H^{17}), 7.33 – 7.27 (5H, m, H^{12-14}), 7.06 – 7.01 (2H, m, H^2), 5.05 (1H, d, J = 32.0 Hz, H^8), 4.37 (2H, s, H^5), 3.94 (2H, d, J = 16.3 Hz, H^6), 2.44 (3H, s, H^{19}).

^{13}C NMR (126 MHz, CDCl$_3$):) δ 162.7 (1C, d, J = 246.9 Hz, C^1), 162.30 (1C, d, J = 275.6 Hz, C^7), 143.9 (1C, s, C^{18}), 137.0 (1C, s, C^{15}), 131.7 (2C, s, C^{12}), 131.0 (1C, d, J = 3.2 Hz, C^4), 130.5 (2C, d, J = 8.3 Hz, C^3), 129.9 (2C, s, C^{17}), 128.8, (1C, s, C^{14}) 128.5, (2C, s, C^{13}) 127.5 (2C, s, C^{16}), 122.8 (1C, s, C^{11}), 115.9 (2C, d, J = 21.5 Hz, C^2), 95.4 (d, J = 5.7 Hz, C^9), 92.7 (1C, d, J = 12.3 Hz, C^8), 80.3 (1C, d, J = 2.3 Hz, C^{10}), 50.2 (1C, s, C^5), 46.3 (1C, d, J = 27.0 Hz, C^6), 21.7 (1C, s, C^{19}).

^{19}F NMR (376 MHz, CDCl$_3$): δ -95.85 (1F, dt, J = 32.4, 16.4 Hz, F^7), -113.77 – -113.98 (1F, m, F^1)

HRMS (ESI) calc: $[M+Na]^+$ ($C_{25}H_{21}F_2NO_2S$) 460.1154; measured: 460.1149 = 0.86 ppm difference.

IR (neat) ν_{max}**/cm^{-1}:** 2983, 2901, 1674, 1603, 1507, 1358, 1339, 1219, 1154, 1089, 1055, 935, 899, 876, 811, 774, 749, 691, 677, 654, 577, 548, 516, 484, 452.

(Z)-6-fluoro-6-(thiophen-3-yl)hex-5-en-3-yn-1-ol ***(11e)***

To a Schlenk tube equipped with a stirrer bar, was added [$(PPh_3)_2PdCl_2$] (10.5 mg, 0.015 mmol, 0.05 equiv.), CuI (4.6 mg, 0.024 mmol, 0.08 equiv.), and the *Z*-FVI (138 mg, 0.3 mmol, 1 equiv.). The tube was placed under vacuum and back-filled with nitrogen for three cycles. Degassed MeCN (1 mL), NEt_3 (84 μL, 0.6 mmol, 2 equiv.), and 3-Butyn-1-ol (50 μL, 0.66 mmol, 2.2 equiv.) were added in short succession. The mixture was stirred for 45 minutes, and a saturated aqueous solution of ammonium chloride was added. The aqueous and organic layers were separated, and the aqueous phase was extracted with DCM (3 x). The combined organic layers were washed with water (2 x) and brine (3 x), then evaporation under reduced pressure. The resulting crude mixture was subjected to flash-column chromatography (0 to 50% Et_2O in pentane) to afford the product as an orange solid (26 mg, 44 % yield).

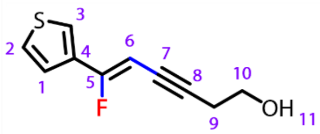

^{1}H NMR (500 MHz, $CDCl_3$): δ 7.49 (1H, d, *J* = 3.0 Hz, H^1), 7.31 (1H, dt, *J* = 5.2, 2.9 Hz, H^2), 7.13 (1H, dt, *J* = 5.1, 1.2 Hz, H^3), 5.37 (1H, dt, *J* = 33.3, 2.3 Hz, H^6), 3.79 (2H, t, *J* = 6.2 Hz, H^{10}), 2.68 (2H, tdd, *J* = 6.3, 2.3, 1.1 Hz, H^9).

^{13}C NMR (126 MHz, $CDCl_3$): δ 161.8 (1C, d, *J* = 257.6 Hz, C^5), 133.4 (1C, d, *J* = 28.9 Hz, C^4), 127.1 (d, *J* = 2.1 Hz, C^2), 123.9 (1C, d, *J* = 6.7 Hz, C^3), 123.0 (1C, d, *J* = 4.6 Hz, C^1), 93.8 (1C, d, *J* = 5.7 Hz, C^7), 86.8 (1C, d, *J* = 15.6 Hz, C^6), 75.0 (1C, d, *J* = 3.1 Hz, C^8), 61.2 (1C, s, C^{10}), 24.4 (1C, s, C^9).

^{19}F NMR (471 MHz, $CDCl_3$): δ -101.81 (1F, d, *J* = 33.6 Hz, F^5).

HRMS (EI) calc: $[M]^+$ ($C_{10}H_9OFS$); 196.0353 ; measured: 196.0349 = 2.04 ppm difference.

IR (neat) ν_{max}**/cm^{-1}:** 3344, 3110, 3054, 2952, 2888, 2220, 1645, 1521, 1420, 1399, 1299, 1256, 1198, 1155, 1039, 904, 871, 832, 780, 735, 695, 617, 575, 544.

Pd-Catalysed Suzuki-Miyaura Arylation

(Z)-1-fluoro-4-(2-fluoro-5-phenylpent-1-en-1-yl)benzene ***(12a)***

To a glass vial equipped with a stirrer bar, was added *(Z)*-(2-fluoro-5-phenylpent-1-en-1yl)(mesityl)iodonium BF_4 (297.6 mg, 0.6 mmol, 1 equiv.), tetraethylammonium iodide (170 mg, 0.66 mmol, 1.1 equiv.), and MeCN (5 mL). The suspension was stirred at 80°C for 6 hours and the solvent was evaporated under reduced pressure. [$(Ph_3P)_2PdCl_2$] (31.6 mg, 0.045 mmol, 0.075 equiv.), 4-fluorophenyl boronic acid (210 mg, 1.5 mmol, 2.5 equiv.), and K_2CO_3 (207 mg, 1.5, 2.5 equiv.) were added. The vial was resealed with a septum and flushed with nitrogen before adding degassed toluene:water (1:0.06) (5.3 mL). The mixture was stirred at 80 °C for 18 hours and saturated aqueous solution of ammonium chloride was added. The aqueous and organic layers were separated, and the aqueous phase was extracted with DCM (3 x). The combined organic layers were evaporated under reduced pressure, and the resulting crude mixture was subjected to flash-column chromatography (0 to 20% Et_2O in pentane) to afford the product as a colourless oil (82 mg, 53%).

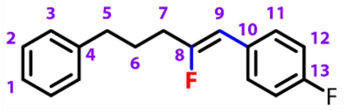

^{1}H NMR (600 MHz, $CDCl_3$): δ 7.46 – 7.42 (2H, m, H^{12}), 7.33 – 7.29 (2H, m, H^3), 7.23 – 7.19 (3H, m, H^{1+2}), 7.03 – 6.98 (2H, m, H^{11}), 5.44 (1H, d, J = 39.1 Hz, H^9), 2.71 (2H, t, J = 7.6 Hz, H^5), 2.35 (2H, dt, J = 18.1, 7.4 Hz, H^7), 1.94 (2H, p, J = 7.6 Hz, H^6).

^{13}C NMR (151 MHz, $CDCl_3$): δ 161.6 (1C, dd, J = 246.3, 3.4 Hz, C^{13}), 160.5 (1C, dd, J = 265.9, 2.3 Hz, C^8), 141.7, (1C, s, C^4), 130.0 (1C, appt, J = 7.7 Hz, C^{11+10}), 128.61 (2C, s, C^3), 128.56 (2C, s, C^2), 126.1 (1C, s, C^1), 115.4 (2C, d, J = 21.4 Hz, C^{12}), 105.2 (1C, d, J = 8.9 Hz, C^9), 35.1 (1C, s, H^5), 32.6 (1C, d, J = 26.5 Hz, C^7), 28.0 (1C, s, C^6).

^{19}F NMR (283 MHz, $CDCl_3$): δ -103.60 (1F, dt, J = 38.3, 18.0 Hz, F^8), -116.27 – -116.41 (1F, m, F^{13}).

HRMS (EI) calc: $[M]^+$ ($C_{17}H_{16}F_2$) 258.1215; measured: 258.1214 = 0.39 ppm difference.

IR (neat) v_{max}/cm^{-1}: 3027, 2938, 1690, 1604, 1508, 1454, 1413, 1340, 1161, 1135, 1098, 1080, 1030, 1015, 976, 892, 846, 822, 783, 744, 698, 622, 654, 520, 474, 415.

Direct reaction from the FVI:

To a glass vial equipped with a stirrer bar, was added [(DPPF)$PdCl_2$].DCM (4mg, 0.005 mmol, 5 mol%), 4-flurophenyl boronic acid (21 mg, 0.15 mmol, 1.5 equiv.), $NaHCO_3$ (34 mg, 0.4 mmol, 4 eq.), and (Z)-(2-fluoro-5-phenylpent-1-en-1-yl)(mesityl)iodonium BF_4 (49.6 mg, 0.1 mmol, 1 equiv.). The vial was sealed with a septum and placed under a nitrogen atmosphere. DMF:water (1:1), which was sparged under nitrogen, was then added (1 mL). The reaction was stirred at 40 °C 24 hours, and hexafluorobenzene was added to determine the ^{19}F NMR yield.

(Z)-2-(2-fluoro-5-phenylpent-1-en-1-yl)benzo[b]thiophene ***(12b)***

To a Schlenk tube equipped with a stirrer bar, was added *(Z)*-(2-fluoro-5-phenylpent-1-en-1yl)(mesityl)iodonium BF_4 (248 mg, 0.5 mmol, 1 equiv.),.[(DPPF)$PdCl_2$] (36.5 mg, 0.05 mmol, 0.1 equiv.), Benzo[b]thiophene-2-boronic acid (133 mg, 0.75 mmol, 1.5 equiv.), and $NaHCO_3$ (147 mg, 1.75 mmol, 3.5 equiv.) were added. The Schlenk tube was placed under a nitrogen atmosphere, and to it was added degassed NMP:water (2:1) (5 mL). The mixture was stirred at room temperature for 24 hours and DCM was added (5 mL). The aqueous and organic layers were separated, and the aqueous phase was extracted with DCM (2 x). The combined organic layers washed with brine (2 x), then evaporated under reduced pressure. The resulting crude mixture was subjected to flash-column chromatography (0 to 10% Et_2O in pentane) to afford the product as a white solid (74 mg, 50%).

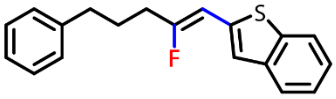

^{1}H NMR (500 MHz, $CDCl_3$): δ 7.52 (1H, d, *J* = 7.7 Hz), 7.44 (1H, d, *J* = 7.3 Hz), 7.07 – 6.99 (4H, m), 6.97 – 6.93 (4H, m), 5.61 (1H, d, *J* = 38.0 Hz), 2.46 (2H, t, *J* = 7.6 Hz), 2.15 (2H, dt, *J* = 17.6, 7.5 Hz), 1.84 – 1.65 1.72 (2H, q, *J* = 7.7 Hz)

^{13}C NMR (126 MHz, $CDCl_3$): δ 161.3 (d, *J* = 268.6 Hz), 141.6, 140.0 (d, *J* = 7.8 Hz), 139.5, 136.4 (d, *J* = 4.2 Hz), 128.6 (d, *J* = 3.1 Hz), 126.2, 124.4, 124.3, 123.2 (d, *J* = 1.5 Hz), 122.19, 122.16, 122.1, 101.5 (d, *J* = 11.9 Hz), 35.1, 32.1 (d, *J* = 25.4 Hz), 27.9.

^{19}F NMR (376 MHz, $CDCl_3$) δ -95.46 (1F, dt, *J* = 38.2, 17.7 Hz).

HRMS (EI) calc: $[M]^+$ ($C_{19}H_{17}FS$) 296.1030; measured: 296.1025 = 1.69 ppm difference.

IR (neat) v_{max}/cm^{-1}: 3661.0, 3056.4, 2956.3, 1684.1, 1601.5, 1495.0, 1454.5, 1433.8, 1317.9, 1226.7, 1155.5, 1127.2, 1066.0, 1011.9, 939.7, 891.6, 866.5, 853.8, 819.9, 744.6, 725.8, 697.3, 685.8, 666.6, 579.2, 494.6, 473.9, 437.3

Pd-Catalysed Carbonylation

Methyl (Z)-3-fluoro-6-phenylhex-2-enoate ***(13a)***

To glass vial equipped, with a stirrer bar and placed inside an autoclave vessel, was added *(Z)*-(2-fluoro-5-phenylpent-1-en-1-yl)(mesityl)iodonium BF_4 (496 mg, 1 mmol, 1 equiv.), $PdCl_2$ (3.5 mg, 0.02 mmol) and triethylamine (140 μL, 1 mmol, 1 equiv.) in wet methanol (10 mL). The vessel was pressurized to 50 psig with carbon monoxide and stirred for 18 hours. The reaction mixture was evaporated udner reduced pressure, and the resulting crude was subjected to flash-column chromatography (0 to 30% EtOAc in pentane) to afford the product as a colourless oil (128 mg, 58%).

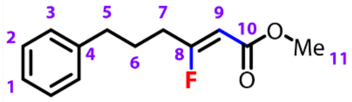

^{1}H NMR (500 MHz, CDCl$_3$): δ 7.32 – 7.27 (2H, m, *H*^{2}), 7.23 – 7.19 (1H, m, *H*^{1}), 7.19 – 7.15 (2H, m, *H*^{3}), 5.19 (1H, dd, *J* = 33.3, 0.7 Hz, *H*^{9}), 3.72 (3H, s, *H*^{11}), 2.68 (2H, t, *J* = 7.6 Hz, *H*^{5}), 2.29 (2H, dt, *J* = 17.2, 7.5 Hz, *H*^{7}), 1.91 (2H, p, *J* = 7.6 Hz, *H*^{6}).

^{13}C NMR (126 MHz, CDCl$_3$): δ 172.0 (1C, d, *J* = 286.7 Hz, *C*^{8}), 164.3 (1C, d, *J* = 1.9 Hz, *C*^{10}), 141.0 (1C, s, *C*^{4}), 128.7 (2C, s, *C*^{2}), 128.6 (2C, s, *C*^{3}), 126.3 (1C, s, *C*^{1}), 98.9 (1C, d, *J* = 5.3 Hz, *C*^{9}), 51.5 (1C, s, *C*^{11}), 34.9 (1C, s, *C*^{5}), 32.5 (1C, d, *J* = 24.0 Hz, *C*^{7}), 27.2 (1C, d, *J* = 1.5 Hz, *C*^{6}).

^{19}F NMR (376 MHz, CDCl$_3$): δ -79.24 (1F, dt, *J* = 33.3, 17.2 Hz, *F*^{8}).

IR (neat) v_{max}/cm^{-1}: 3027, 2945, 1729, 1717, 1683, 1604, 1497, 1453, 1347, 1277, 1217, 1135, 1081, 1035, 978, 894, 833, 746, 699, 588, 564, 533, 493.

HRMS (EI) calc: $[M]^+$ ($C_{13}H_{15}O_2F$) 202.0988; measured: 202.0988 = 0 ppm difference.

Synthesis of *2,4,6-trichlorophenyl formate* CO Surrogate

This substrate was synthesised according to a literature procedure.[41] To a round-bottom flask, equipped with a stirrer bar, was added acetic anhydride (19 mL, 200 mmol, 4.0 equiv.) and formic acid (9.5 mL, 250 mmol, 5.0 equiv.). The stirring mixture was subsequently heated to 60 °C for 1 hour before being poured into a separate round-bottom flask, submerged in a (room-temperature) water bath containing the 2,4,6-trichlorophenol (9.87 g, 50 mmol, 2 equiv.), and sodium acetate (2.05 g, 25 mmol, 1 equiv.). After stirring the latter mixture for 5 hours, toluene was added and washed with water (3 x). The organic phase was evaporated under reduced pressure to afford the product as a white solid (1.71 g, 30% yield).

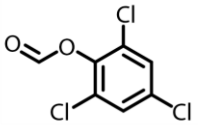

^{1}H NMR (500 MHz, CDCl$_3$): δ 8.28 (1H, s), 7.41 (2H, s).

^{13}C NMR (126 MHz, CDCl$_3$): δ 156.4, 142.0, 132.8, 129.4, 128.9.

These data are consistent with those previously reported.[41]

Optimisation Experiments for the Reaction Employing the CO Surrogate

To a Schlenk tube equipped with a stirrer bar, was added *(Z)*-(2-fluoro-3,3-dimethylbut-1-en-1yl)(mesityl)iodonium BF_4 (21.7 mg, 0.05 mmol, 1 equiv.), 2,4,6-trichlorophenyl formate (22.6 mg, 0.1 mmol, 2 equiv.), ligand (0.005 mmol, 0.05 equiv.), and bis(dibenzylideneacetone)palladium(0) (14.4 mg, 0.0025 mmol, 0.025 equiv.). The tube was placed under vacuum and back-filled with nitrogen for three cycles. Degassed solvent (500 µL) was added, followed by triethylamine (14 µL, 0.1 mmol, 2 equiv.), and the reaction was stirred for 18 hours at room temperature before determining the yield by ^{19}F NMR relative to hexafluorobenzene.

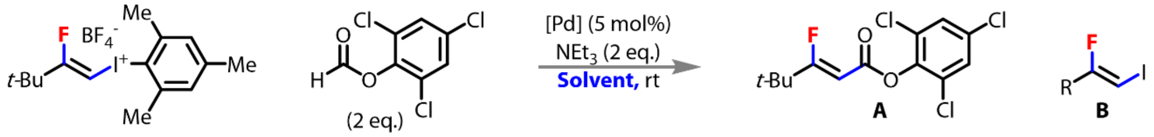

Table S5

Entry	[Pd]	Solvent	RSM (NMR) / %	Yield (NMR) / % (A,B)
1	$[(Ph_3P)_2PdCl_2]$	EtOAc	55	0,31
2	$[(Ph_3P)_2PdCl_2]$	2-Propanol	53	0,24
3	$[(Ph_3P)_2PdCl_2]$	MeCN	37	4,12
4	$[(Ph_3P)_2PdCl_2]$	DMF	55	9,20
5	$[(Ph_3P)_2PdCl_2]$	DMC	54	5,25
6	$[(Ph_3P)_2PdCl_2]$	DCM	50	10,21
7	$[(Ph_3P)_2PdCl_2]$	$PhCF_3$	46	10,25
8	$[(Ph_3P)_2PdCl_2]$	PhMe	30	2,55
9	$[(Ph_3P)_2PdCl_2]$	THF	15	24,37
10	$Pd_2(dba)_3$/XPhos	THF	41	20,11
11	$Pd_2(dba)_3$/CyJohnPhos	THF	0	58,13
12	$Pd_2(dba)_3$/DavePhos	THF	0	51,14
13	$Pd_2(dba)_3$/SPhos	THF	68	3,8
14	$Pd_2(dba)_3$/Xantphos	THF	22	68,11
15	$Pd_2(dba)_3$/BINAP	THF	0	76,4
16	$Pd_2(dba)_3$/DPPB	THF	27	19,13
**17**	**Pd2(dba)$_3$/DPPF**	**THF**	**0**	**82,6**

^{19}F NMR Yields and conversions were determined by relative integration to hexafluorobenzene. RSM = Remaining starting material (FVI).

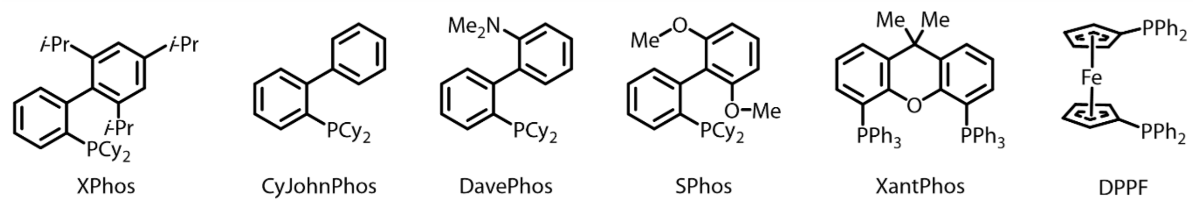

2,4,6-Trichlorophenyl (Z)-3-fluoro-4,4-dimethylpent-2-enoate ***(13b)***

To a Schlenk tube equipped with a stirrer bar, was added *(Z)*-(2-fluoro-3,3-dimethylbut-1-en1-yl)(mesityl)iodonium BF_4 (434 mg, 1 mmol, 1 equiv.), 2,4,6-trichlorophenyl formate (452 mg, 2 mmol, 2 equiv.), DPPF (27.8 mg, 0.05 mmol, 0.05 equiv.), bis(dibenzylideneacetone)palladium(0) (14.4 mg, 0.025 mmol, 0.025 equiv.). The tube was placed under vacuum and back-filled with nitrogen for three cycles. Degassed THF (5 mL), followed by triethylamine (280 µL, 2 mmol, 2 equiv.) was added and the reaction was stirred for 18 hours at room temperature. The reaction mixture was evaporated under reduced pressure, and the resulting crude mixture was subjected to flash-column chromatography (0 to 30% EtOAc in pentane) to afford a colourless oil that slowly solidified (222 mg, 68%).

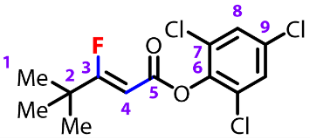

^{1}H NMR (500 MHz, CDCl$_3$): δ 7.38 (2H, s, H^8), 5.55 (1H, d, J = 32.8 Hz, H^4), 1.26 (9H, s, H^1).

^{13}C NMR (126 MHz, CDCl$_3$):) δ 182.6 (1C, d, J = 295.8 Hz, C^3), 160.0 (1C, s, C^5), 143.0 (1C, s, C^6) , 132.0 (1C, s, C^9), 129.9 (2C, s, C^7), 128.7 (2C, s, C^8), 93.6 (d, J = 5.6 Hz, C^4), 36.6 (d, J = 20.6 Hz, C^2), 26.9 (d, J = 2.8 Hz, C^1).

^{19}F NMR (376 MHz, CDCl$_3$): δ -80.83 (1F, d, J = 32.8 Hz, F^3).

HRMS (APCI) calc: $[M+H]^+$ ($C_{13}H_{12}O_2Cl_3F$) 324.9960; measured: 324.9959 = 0.31 ppm difference.

IR (neat) v_{max}**/cm^{-1}:** 3083, 2973, 2876, 1765, 1747, 1664, 1563, 1481, 1447, 1386, 1372, 1322, 1278, 1238, 1216, 1188, 1116, 1096, 1029, 932, 898, 854, 819, 784, 698, 675, 651, 563.

(Z)-3-fluoro-4,4-dimethyl-N-(4-methylbenzyl)pent-2-enamide ***(13c)***

To a Schlenk tube equipped with a stirrer bar, was added *(Z)*-(2-fluoro-3,3-dimethylbut-1-en-1yl)(mesityl)iodonium BF_4 (217 mg, 0.5 mmol, 1 equiv.), 2,4,6-trichlorophenyl formate (226 mg, 1 mmol, 2 equiv.), DPPF (13.9 mg, 0.025 mmol, 0.05 equiv.), bis(dibenzylideneacetone)palladium(0) (7.2 mg, 0.0125 mmol, 0.025 equiv.). The tube was placed under vacuum and back-filled with nitrogen for three cycles. Degassed THF (2.5 mL) was added, and the reaction was stirred for 18 hours at room temperature. THF (2 mL) was added, followed by 4-methyl benzylamine (95.5 *µ*L, 0.75 mmol, 1 equiv.), DMAP (3 mg, 0.025 mmol, 0.05 equiv.), and NEt_3 (139 *µ*L, 1 mmol, 2.0 equiv.). The mixture was heated to 45 °C, and stirred for 18 hours. After cooling to room temperature, the crude material was subjected to flash column chromatography (0 to 30% EtOAc in pentane) to yield a colourless solid (70 mg, 56% over two steps).

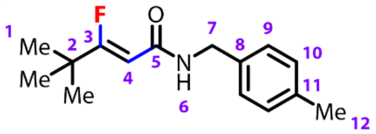

^{1}H NMR (500 MHz, $CDCl_3$): δ 7.21 (2H, d, *J* = 8.0 Hz, H^9), 7.15 (2H, d, *J* = 7.8 Hz, H^{10}), 6.55 (1H, brs, H^6), 5.25 (1H, d, *J* = 40.5 Hz, H^4), 4.48 (2H, dd, *J* = 5.7, 1.3 Hz, H^7), 2.34 (3H, s, H^{12}), 1.16 (9H, d, *J* = 0.9 Hz, H^1).

^{13}C NMR (126 MHz, $CDCl_3$): δ 174.1 (1C, d, *J* = 274.3 Hz, C^3), 164.0 (1C, s, C^5), 137.3 (2C, s, C^{10}), 135.4 (2C, s, C^9), 129.5 (1C, s, C^{11}), 127.9 (1C, s, C^8), 100.3 (1C, d, *J* = 9.0 Hz, C^4), 43.5 (1C, s, C^7), 35.7 (1C, d, *J* = 23.6 Hz, C^2), 27.0 (3C, d, *J* = 3.0 Hz, C^1), 21.2 (1C, s, C^{12}).

^{19}F NMR (376 MHz, $CDCl_3$): δ -98.93 (1F, dd, *J* = 40.5, 11.0 Hz, F^3).

HRMS (ESI) calc: $[M]^+$ ($C_{15}H_{21}NOF$) 250.1607; measured: 250.1604 = 1.2 ppm difference.

IR (neat) ν_{max}**/cm^{-1}:** 3276, 2973, 2922, 2870, 1682, 1630, 1540, 1362, 1282, 1213, 1096, 1033, 877, 853, 808, 757, 697, 660, 560, 540, 480.

FVI and 1,2-Halofluoroalkene Reactivity Comparison Experiments:

Effect of (aryl)-Iodane Structure

The specified FVI or iodofluoroalkene (0.1 mmol) was subjected to the previously-outlined procedure for copper-catalysed bromination, and the yield/conversion was measured by **^{19}**F NMR, relative to a 4,4'-difluorobiphenyl standard, after stirring at 60 °C for the specified reaction time.

(Z)-(4-fluoro-5-iodopent-4-en-1-yl)benzene

For characterisation purposes, the combined crude reaction mixtures were subjected to flash-column chromatography (0 to 20% EtOAc in pentane), isolating the *Z*-1,2-iodofluoroalkene as a colourless oil.

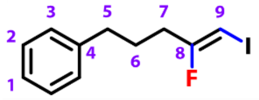

^{1}H NMR (500 MHz, CDCl$_3$): δ 7.31 – 7.27 (2H, m, *H*^{2}), 7.22 – 7.18 (1H, m, *H*^{1}), 7.18 – 7.15 (2H, m, *H*^{3}), 5.20 (1H, d, *J* = 34.6 Hz, *H*^{9}), 2.65 (2H, t, *J* = 7.6 Hz, *H*^{5}), 2.36 (2H, dt, *J* = 16.6, 7.5 Hz 3H), 1.96 – 1.77 (2H, m, *H*^{6}).j

^{13}C NMR (126 MHz, CDCl$_3$): δ 166.3 (1C, d, *J* = 261.3 Hz, *C*^{8}), 141.3, *C*^{4}, 128.60 (2C, s, *C*^{2}), 128.57 (2C, s, *C*^{3}), 126.2 (1C, s, *C*^{1}), 51.3 (1C, d, *J* = 26.8 Hz, *C*^{9}), 34.9 (1C, s, *C*^{5}), 32.3 (1C, d, *J* = 26.9 Hz, *C*^{7}), 27.5 (1C, s, *C*^{6}).

^{19}F NMR (376 MHz, CDCl$_3$): δ -79.44 (1F, dt, *J* = 34.7, 16.5 Hz, *F*^{8}).

HRMS (EI) calc: [M]$^+$ ($C_{11}H_{12}FI$) 289.9964; measured: 289.9962 = 0.69 ppm difference.

.

NMR Spectra of Novel Compounds

1,3-Dibromo-5-(prop-2-yn-1-yloxy)benzene ***(1t)***

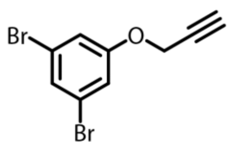

^{1}H NMR (500 MHz, $CDCl_3$):

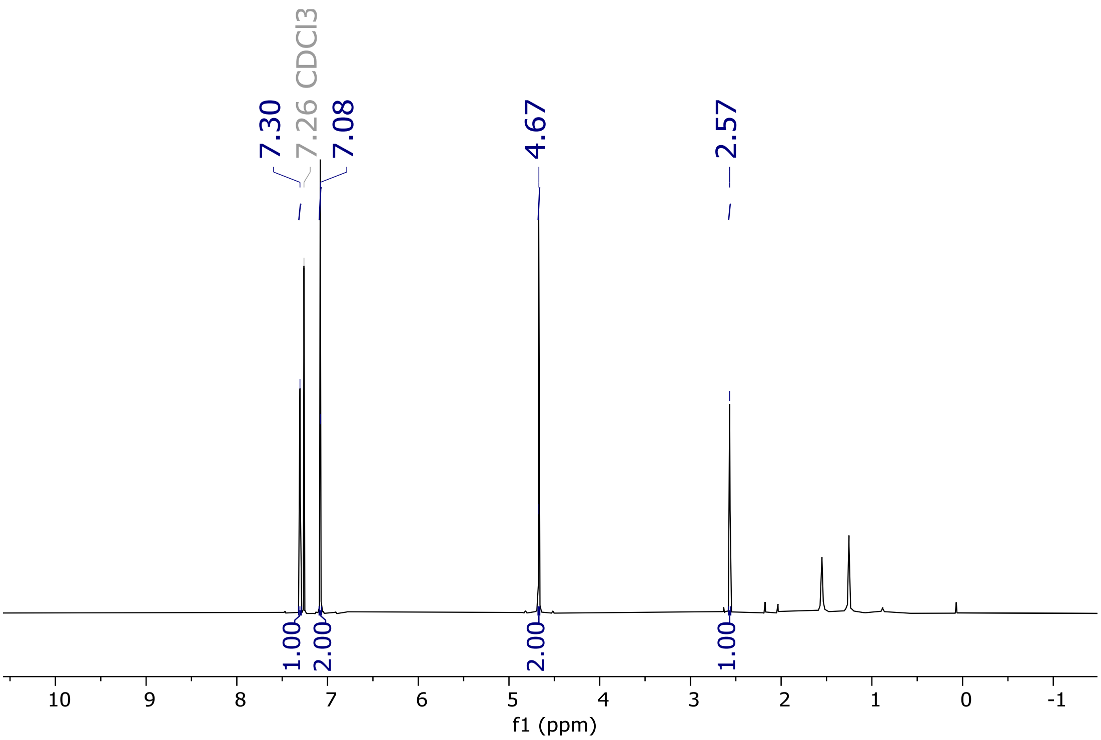

^{13}C NMR (126 MHz, $CDCl_3$):

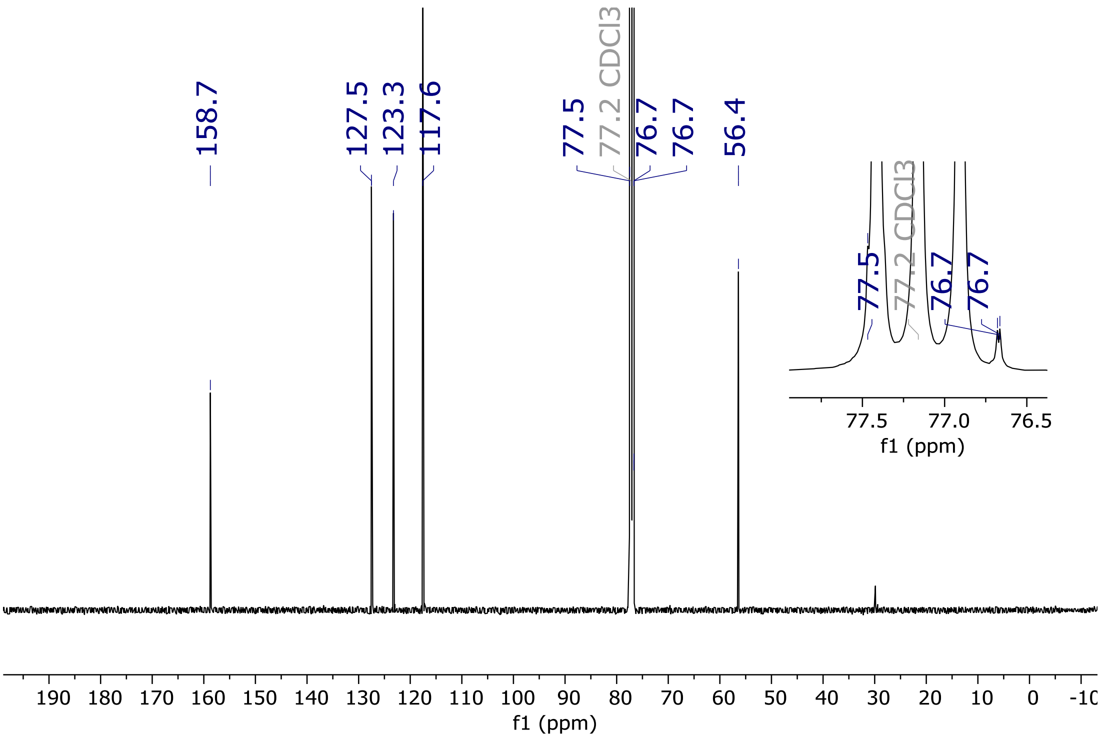

1,3,5-Trichloro-2-(prop-2-yn-1-yloxy)benzene ***(1u)***

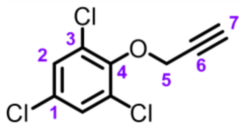

^{1}H NMR (500 MHz, CDCl$_3$):

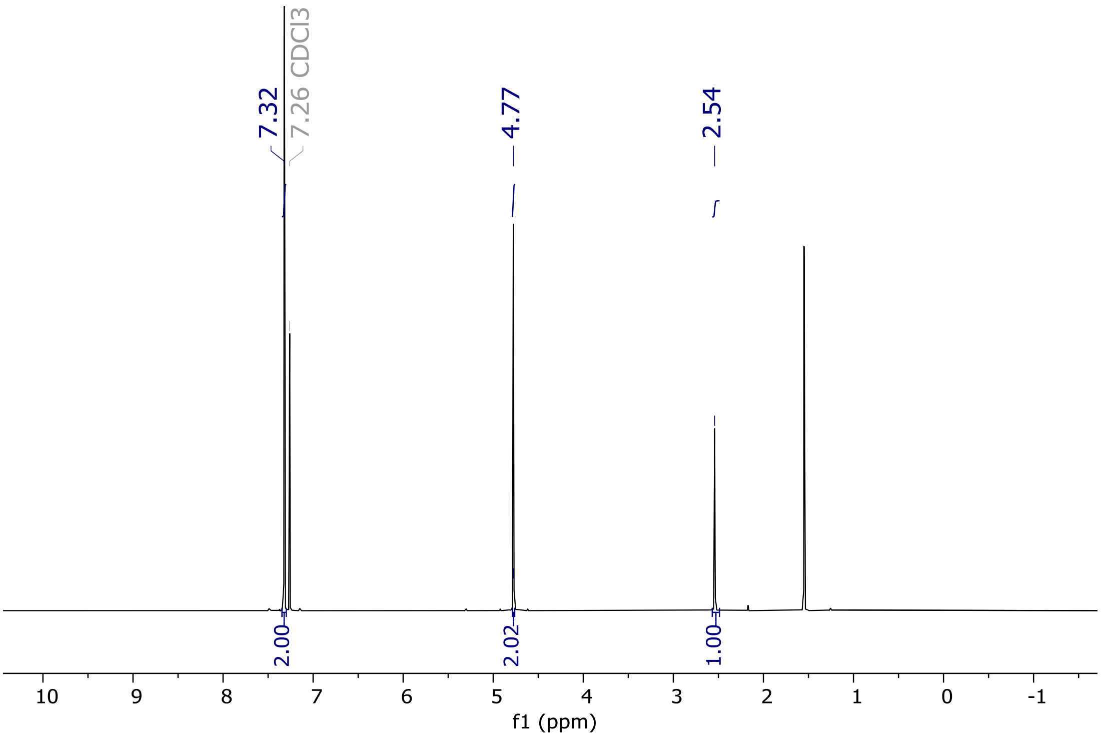

^{13}C NMR (126 MHz, CDCl$_3$):

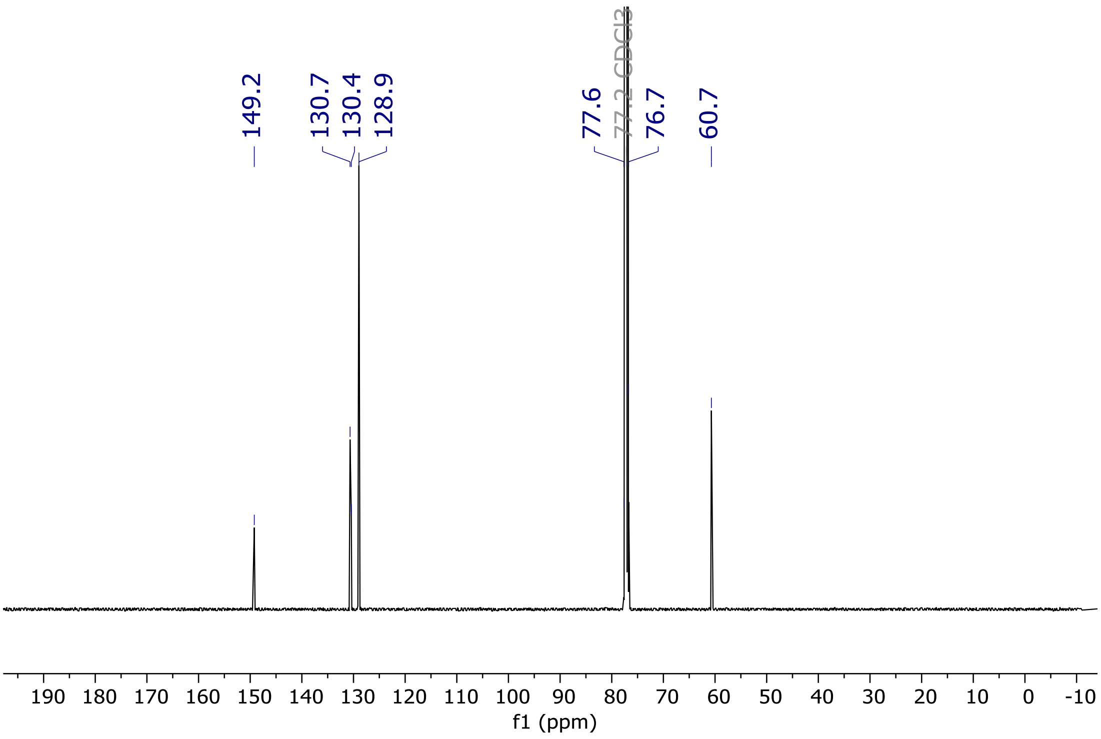

N-(4-fluorobenzyl)-4-nitrobenzenesulfonamide

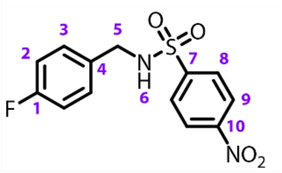

^{1}H NMR (500 MHz, CD_3CN):

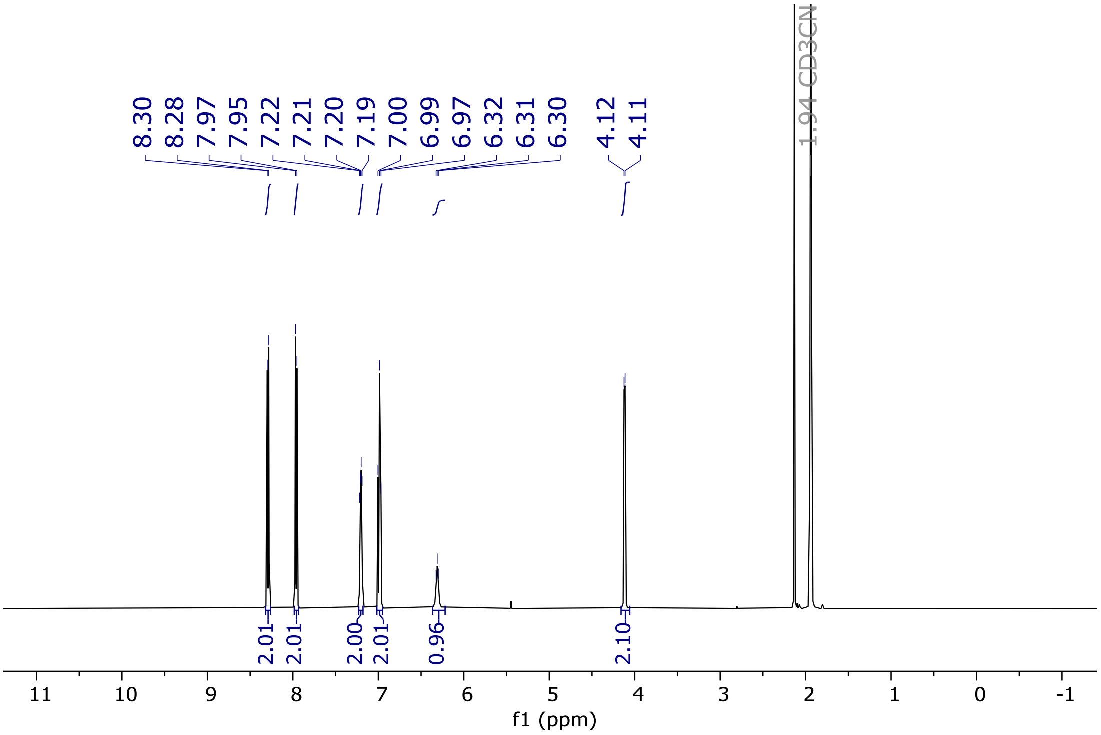

^{13}C NMR (126 MHz, CD_3CN):

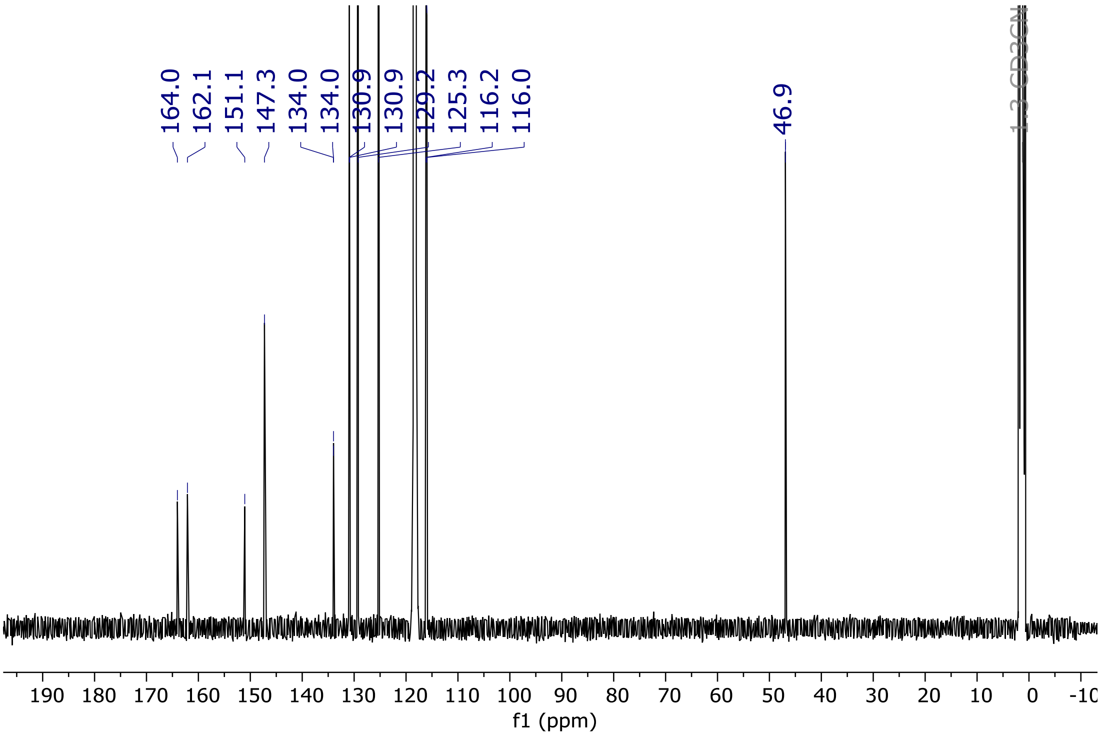

^{19}F NMR (376 MHz, CD_3CN):

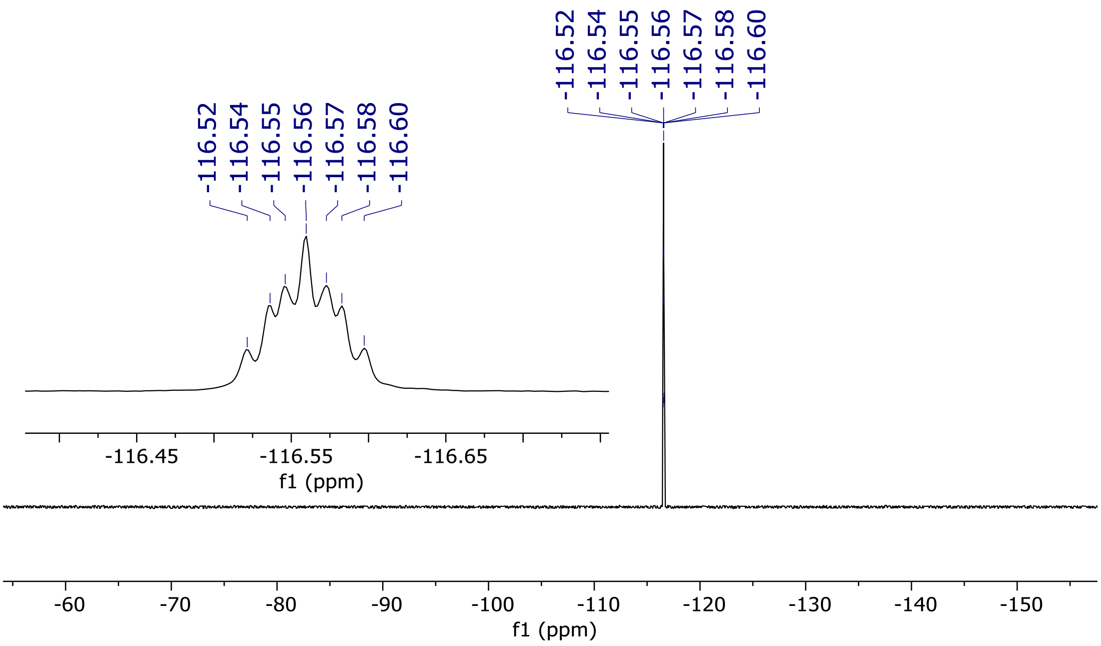

N-(4-fluorobenzyl)-4-nitro-N-(prop-2-yn-1-yl)benzenesulfonamide **(1y)**

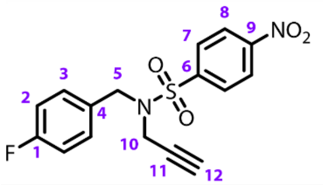

^{1}H NMR (500 MHz, CD_3CN):

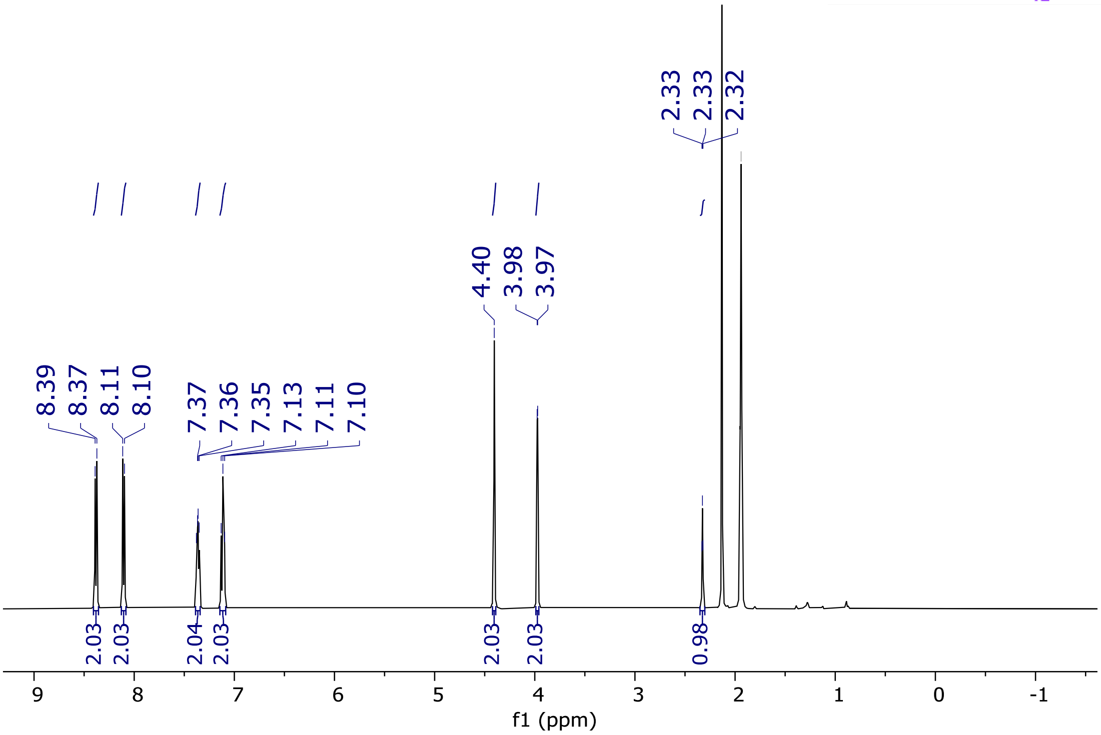

^{13}C NMR (126 MHz, CD_3CN):

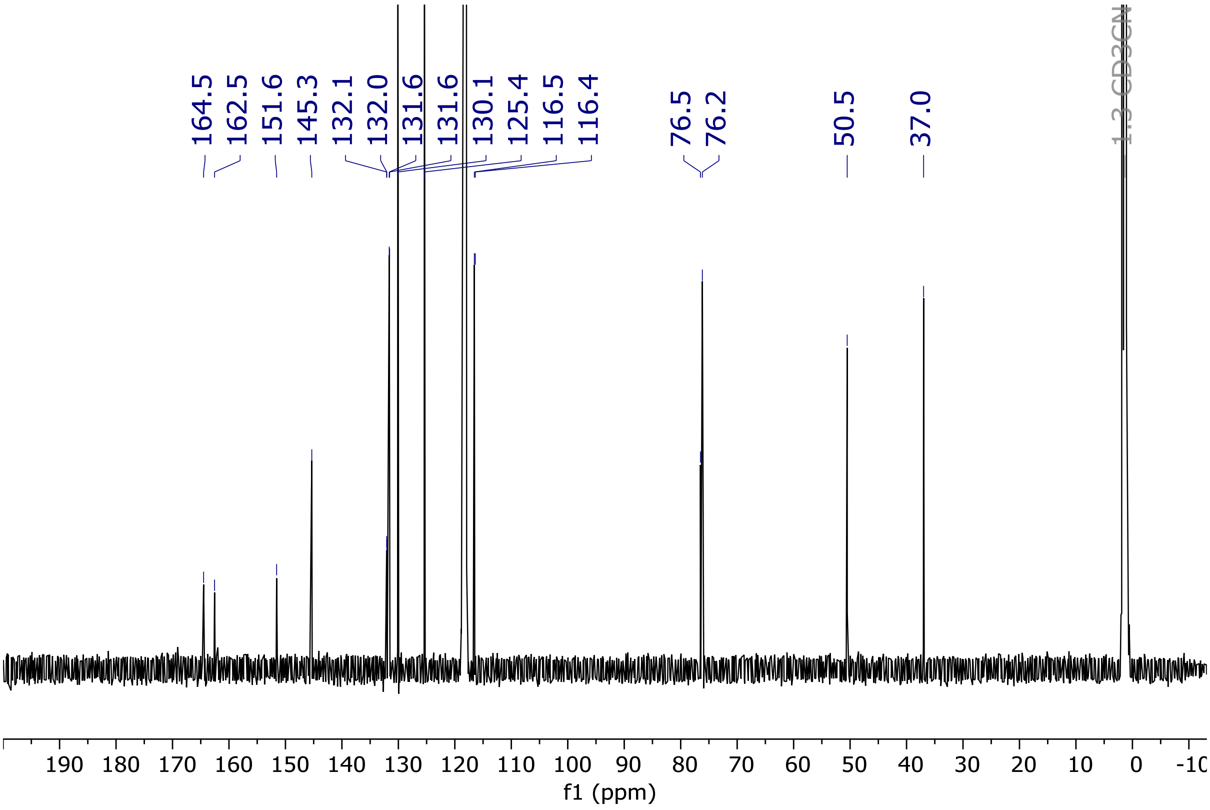

^{19}F NMR (376 MHz, CD_3CN):

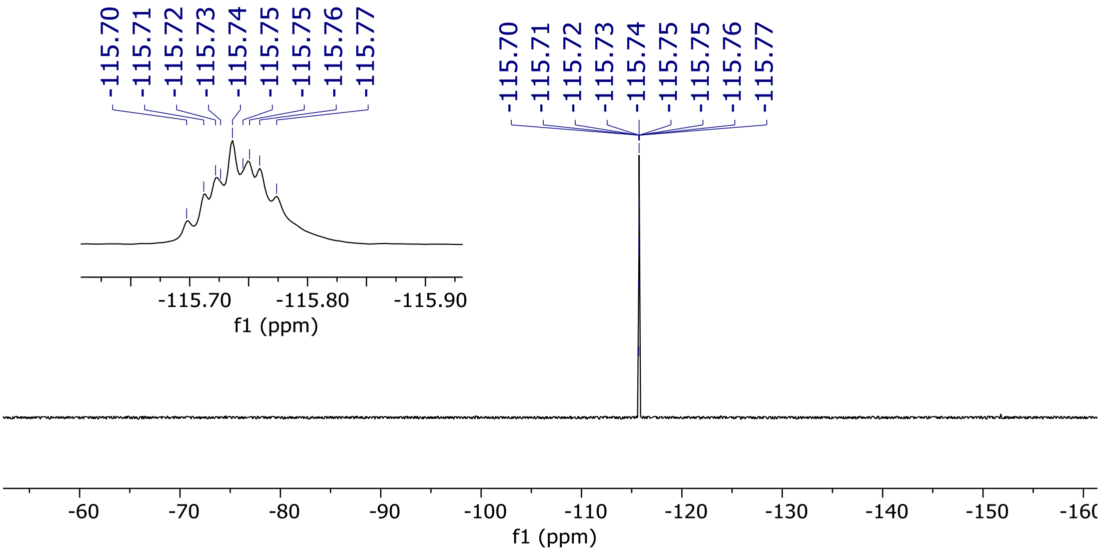

N-cyclopropyl-4-methyl-N-(prop-2-yn-1-yl)benzenesulfonamide ***(1aa)***

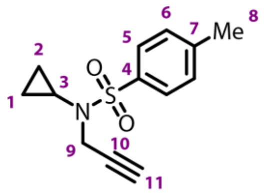

^{1}H NMR (500 MHz, CDCl$_3$):

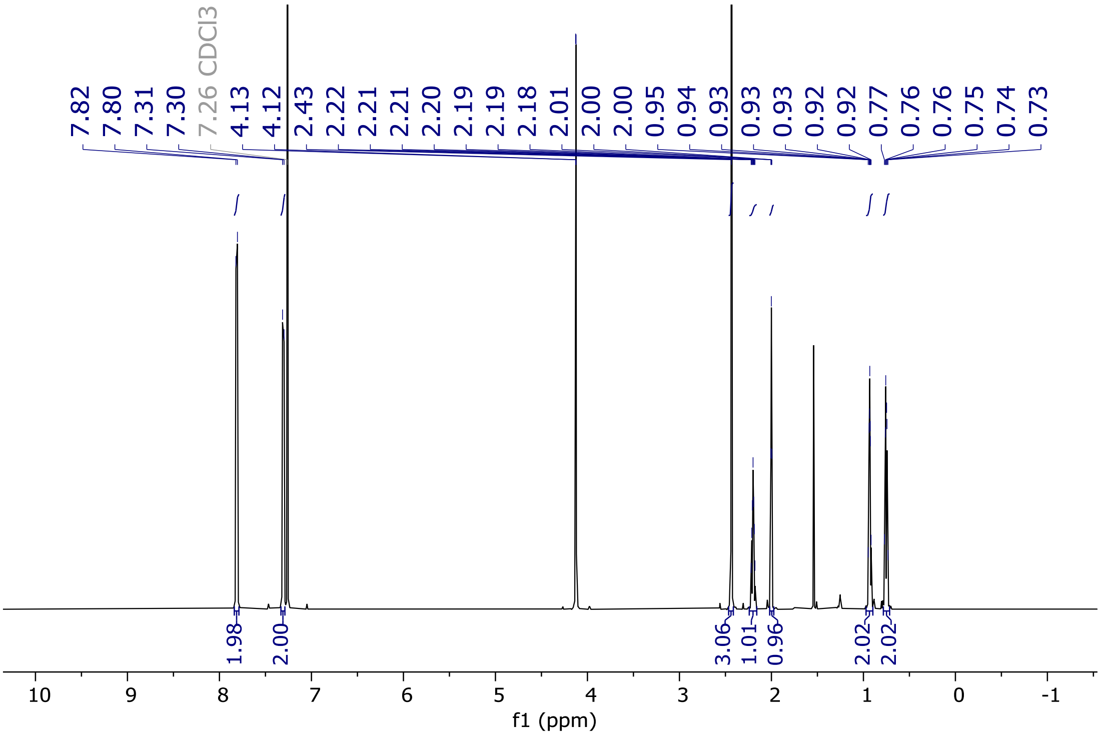

^{13}C NMR (126 MHz, CDCl$_3$):

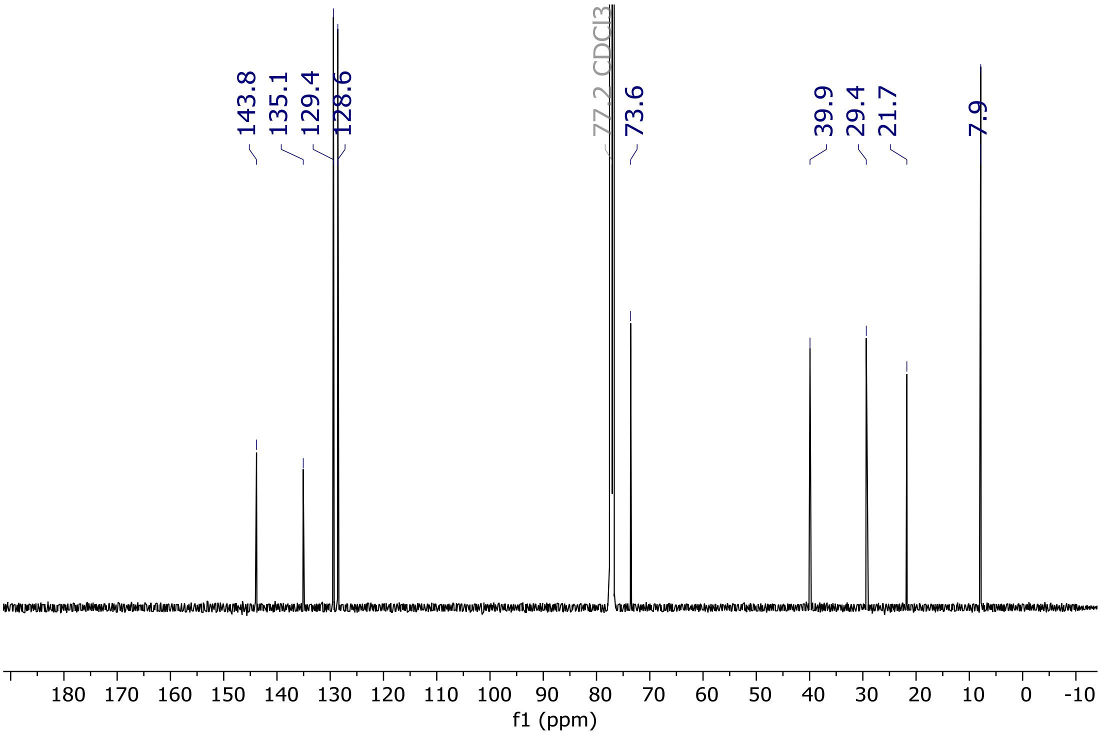

N-(4-fluorobenzyl)-4-methyl-N-(prop-2-yn-1-yl)benzenesulfonamide
(1ac)

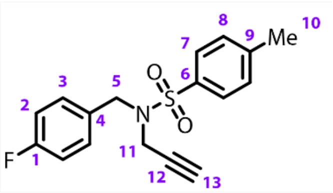

^{1}H NMR (500 MHz, CD_3CN):

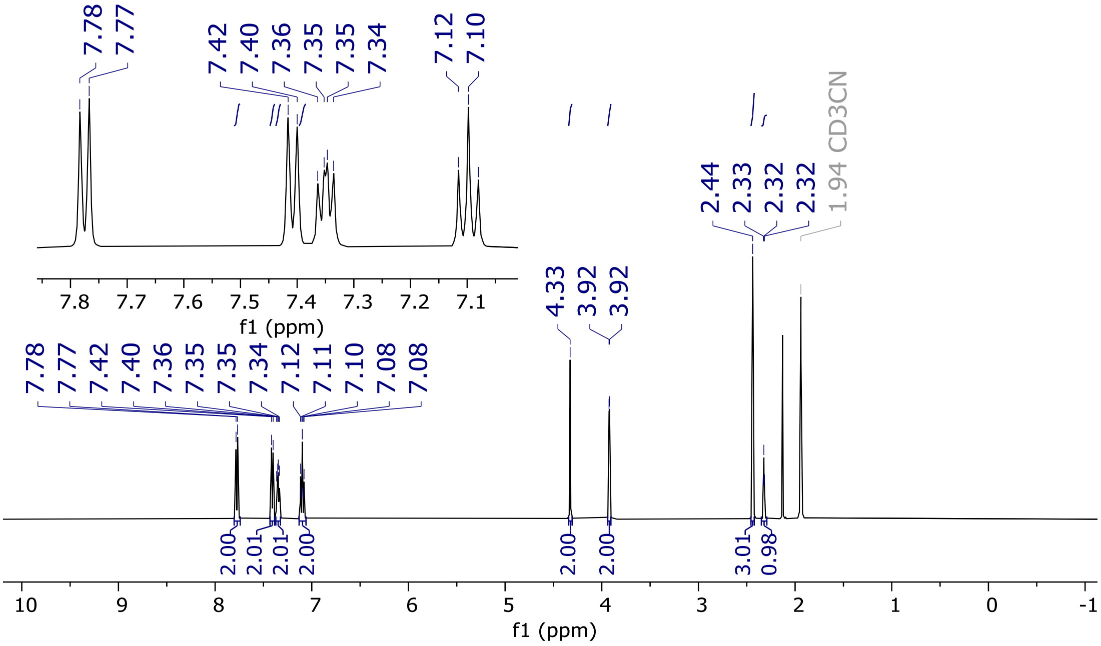

^{13}C NMR (126 MHz, CD_3CN):

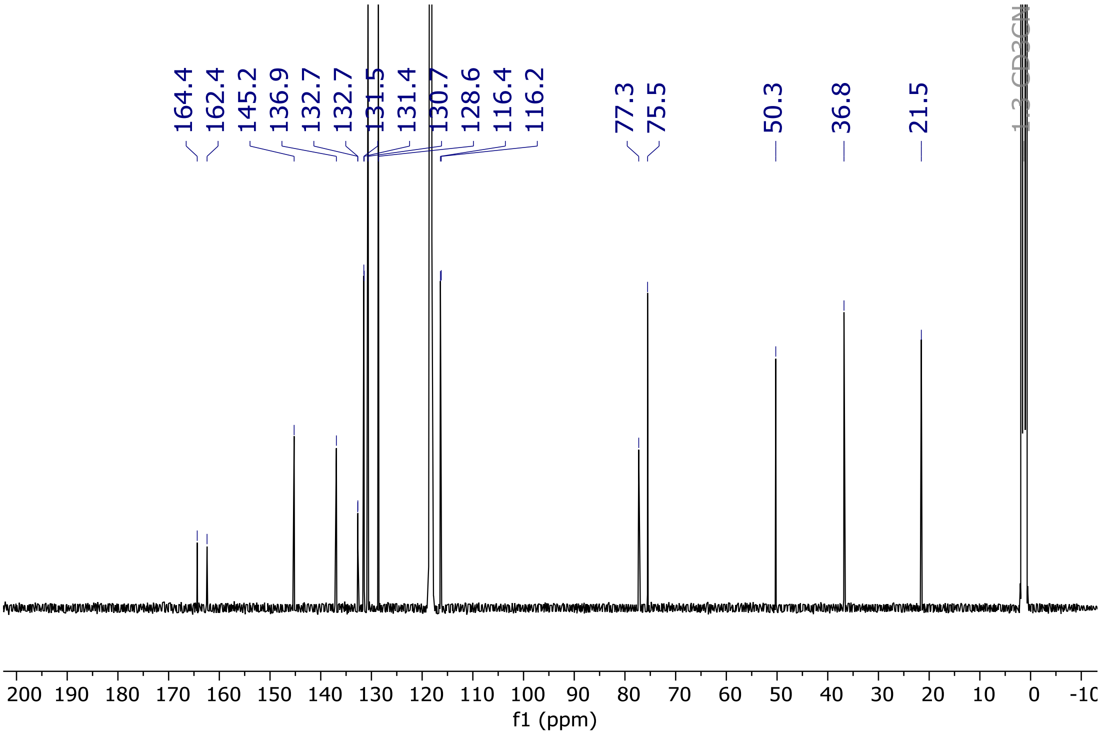

^{19}F NMR (376 MHz, CD_3CN):

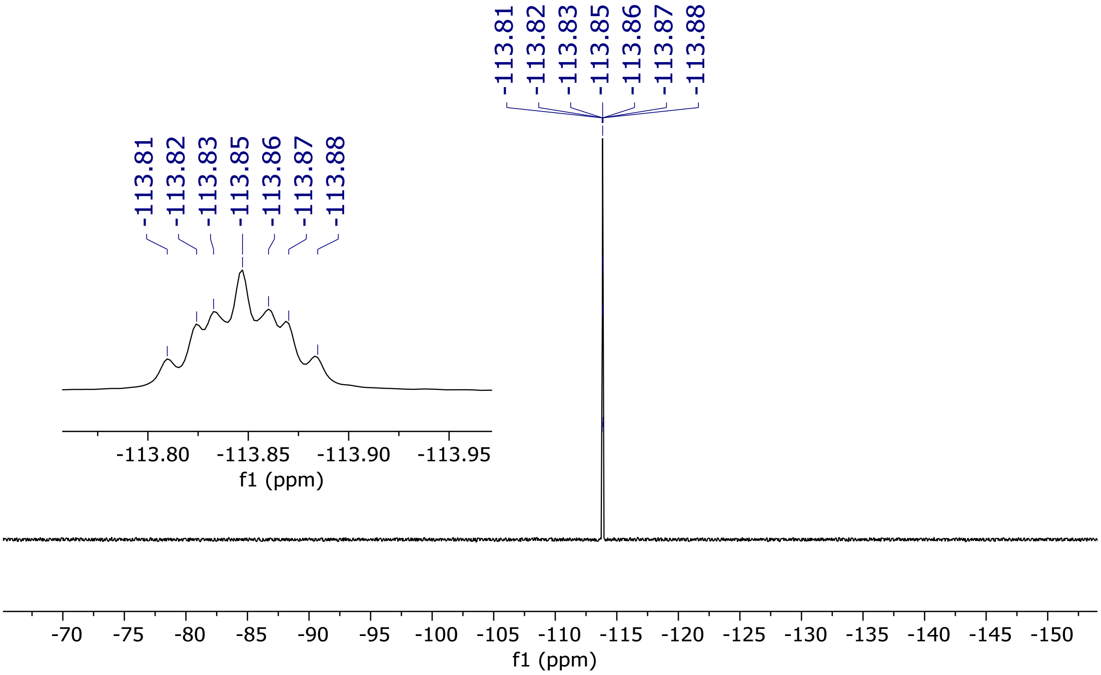

4-Methyl-N-(4-methylbenzyl)-N-(prop-2-yn-1-yl)benzenesulfonamide ***(1ad)***

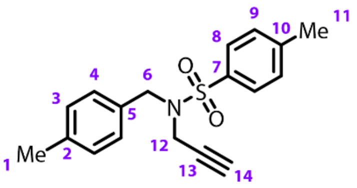

^{1}H NMR (500 MHz, $CDCl_3$):

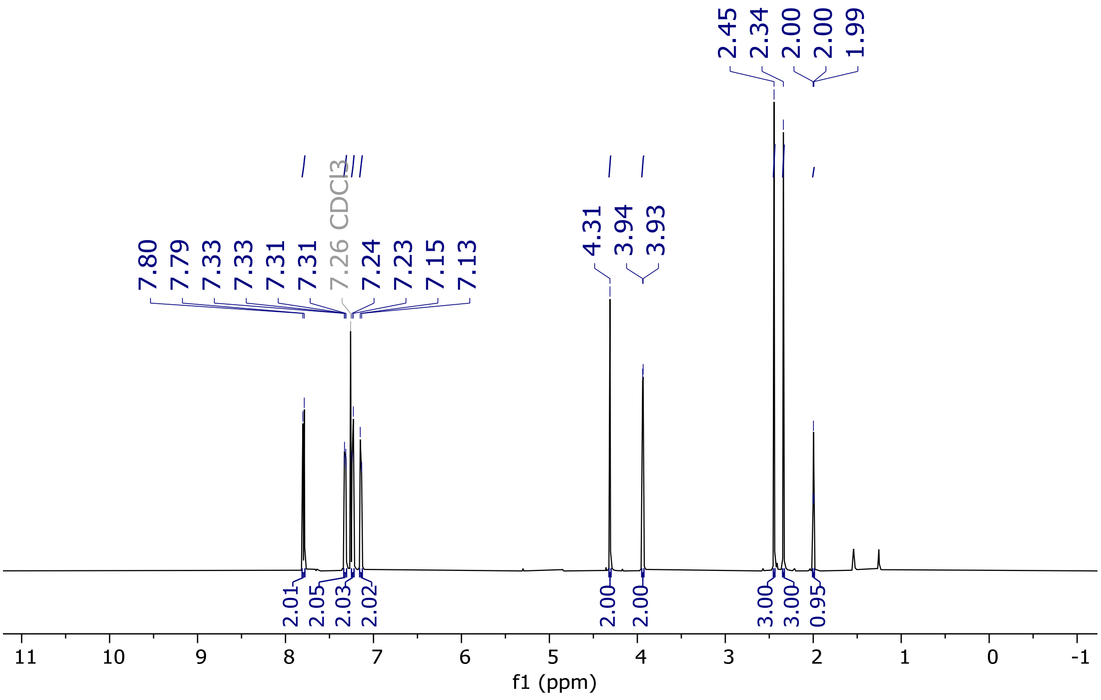

^{13}C NMR (126 MHz, $CDCl_3$):

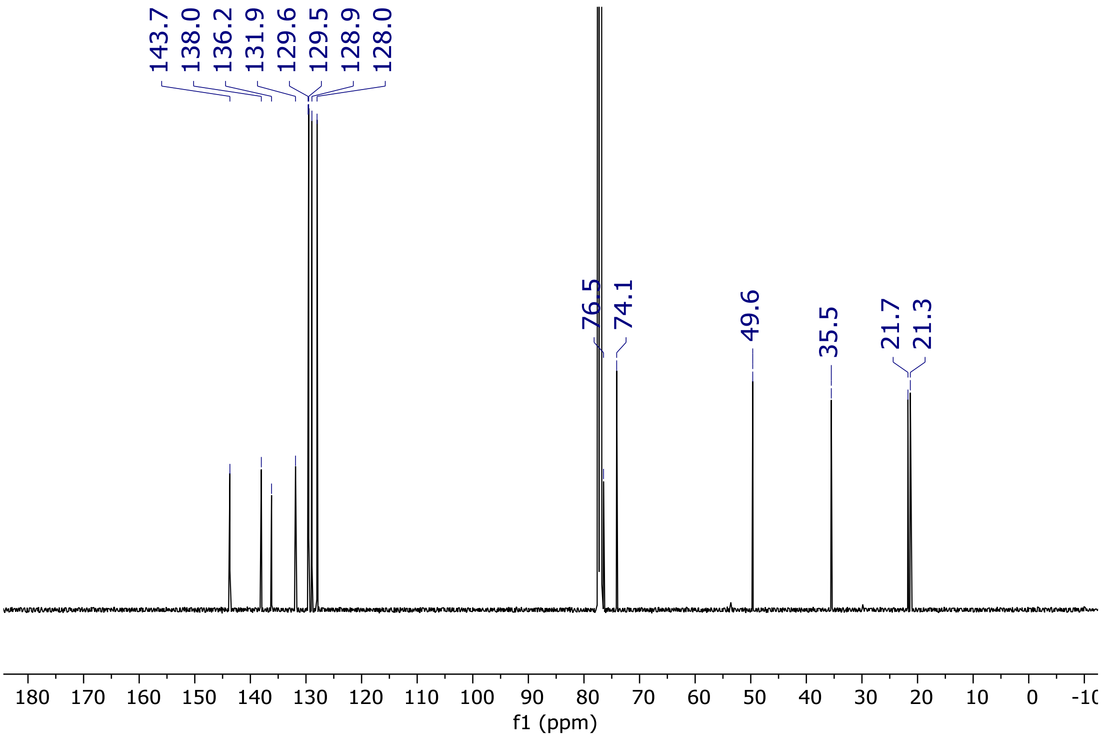

*N-(4-fluorophenyl)-4-methyl-N-(prop-2-yn-1-yl)benzenesulfonamide**(1ae)***

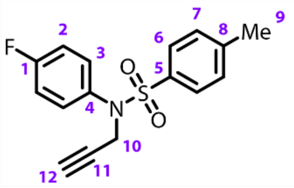

^{1}H NMR (500 MHz, CD$_3$CN):

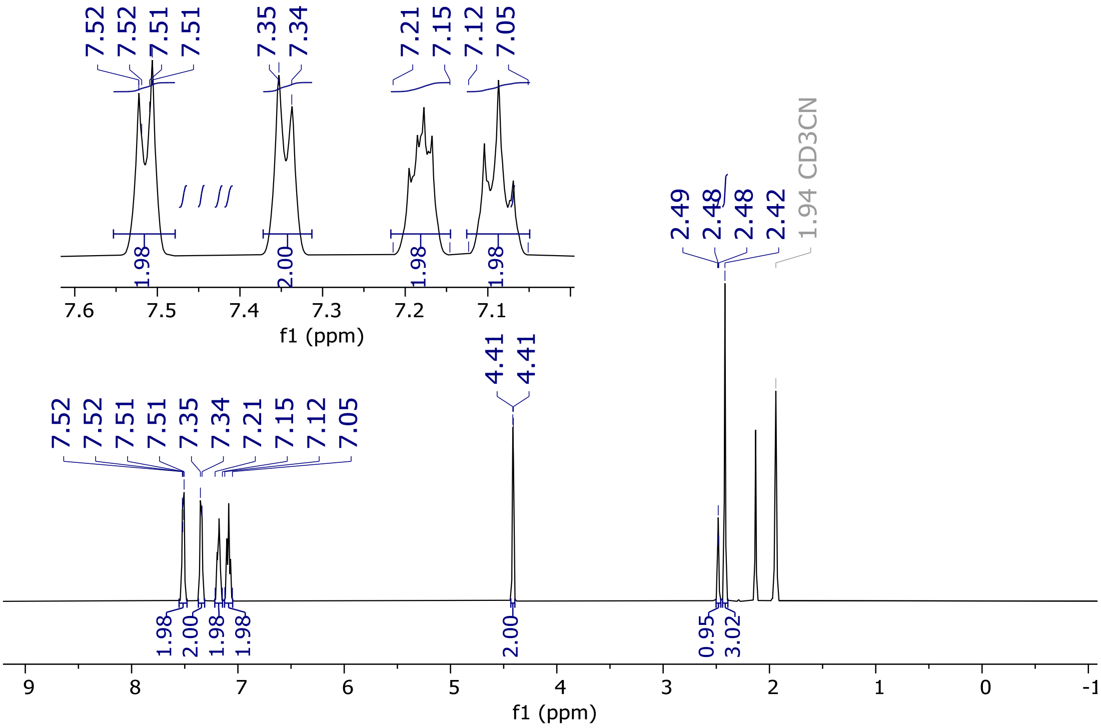

^{13}C NMR (126 MHz, CD$_3$CN):

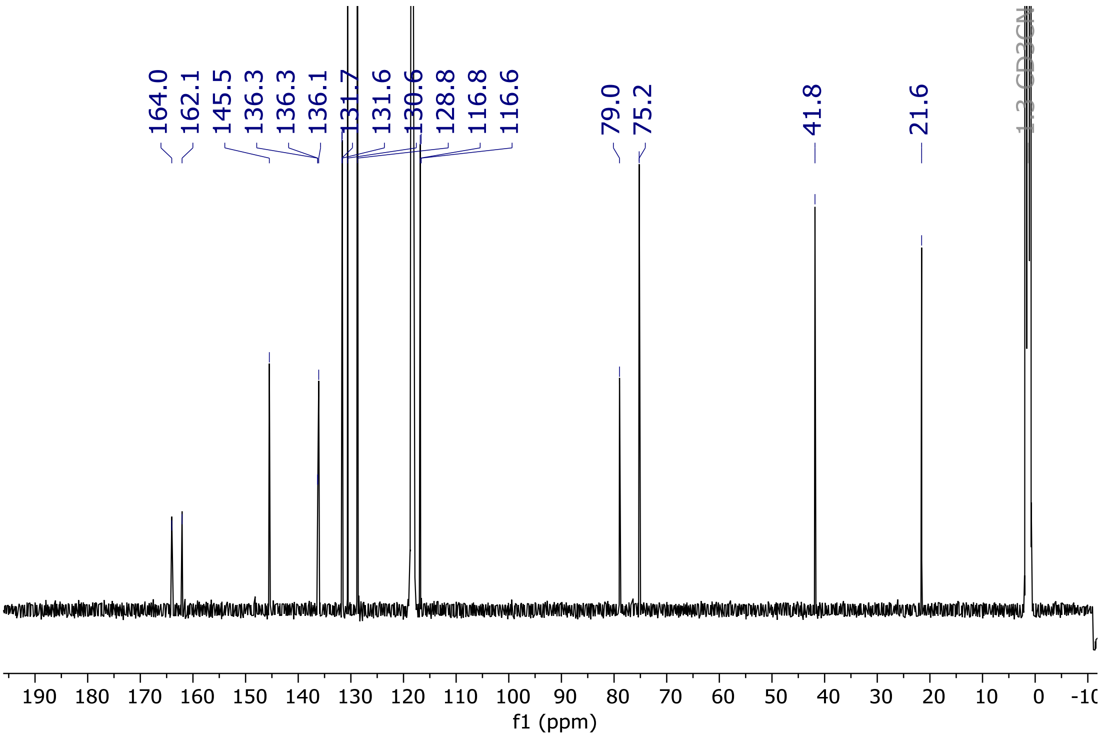

^{19}F (376 MHz, $CDCl_3$):

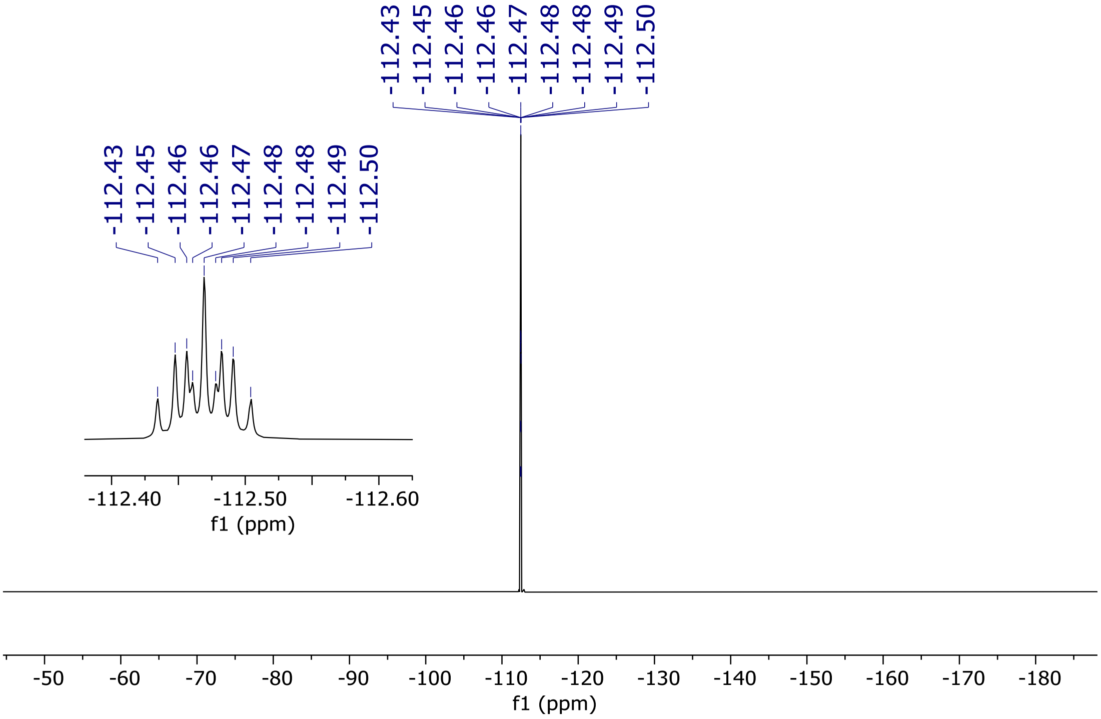

(Z)-(2-fluoro-5-phenylpent-1-en-1-yl)(mesityl)iodonium BF_4 **(3a)**

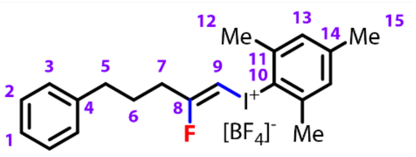

^{1}H NMR (500 MHz, CD_3CN):

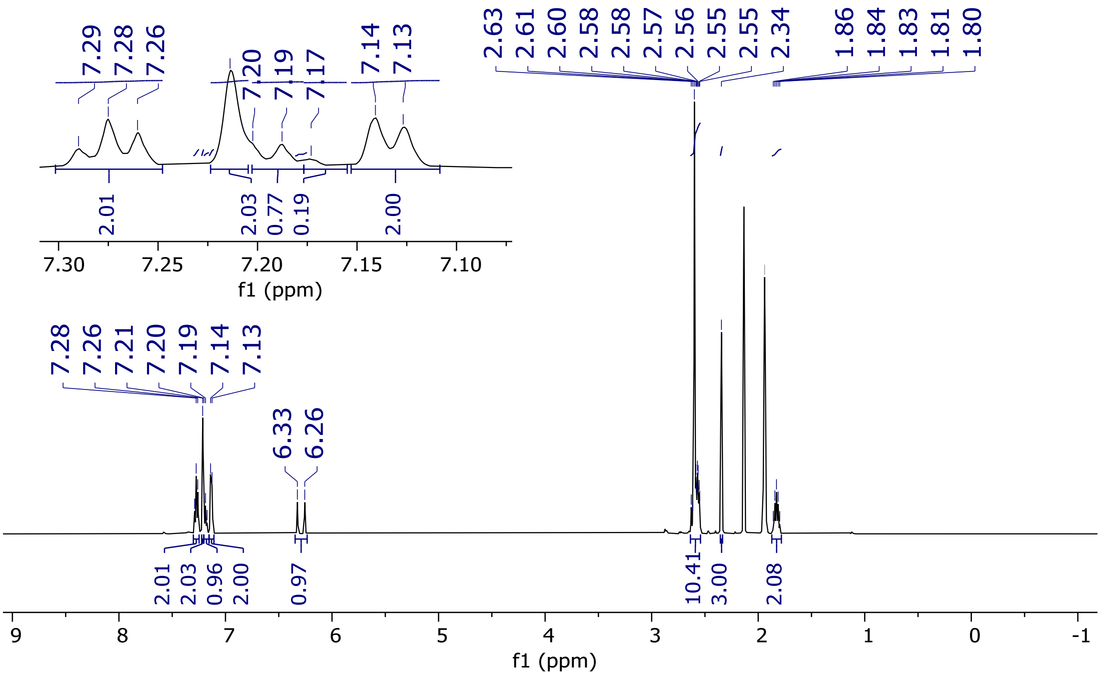

^{13}C NMR (126 MHz, CD_3CN):

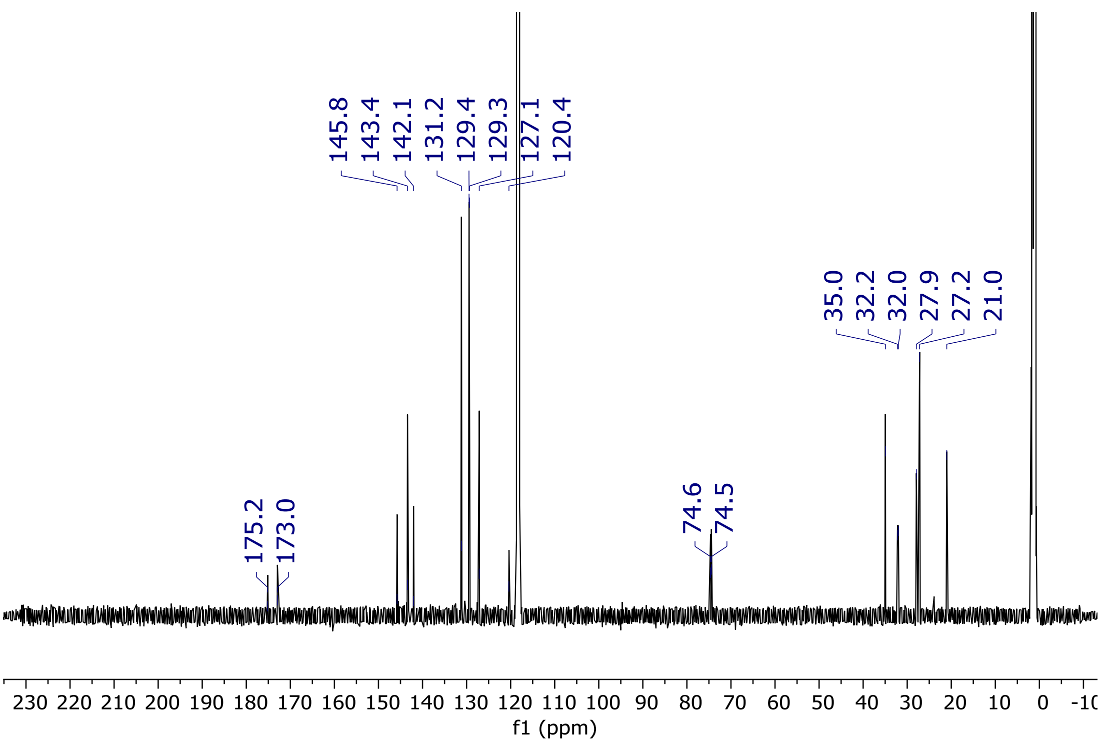

^{19}F NMR (376 MHz, CD_3CN):

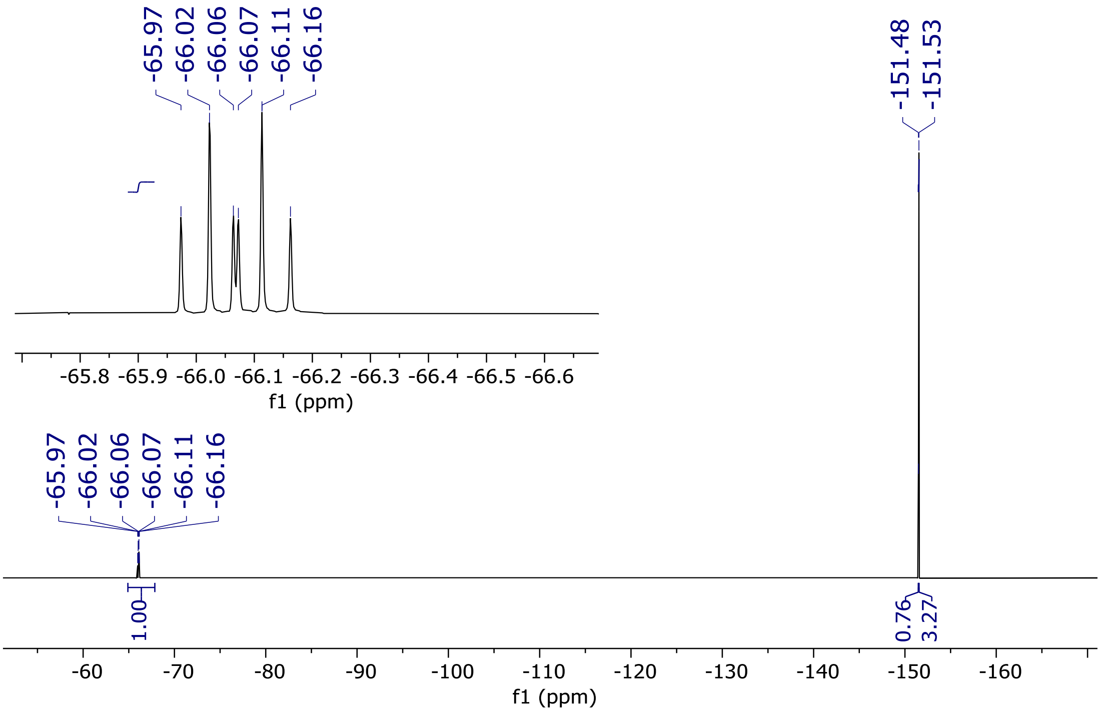

(Z)-(2-fluoro-2-phenylvinyl)(mesityl)iodonium BF_4 **(3b)**

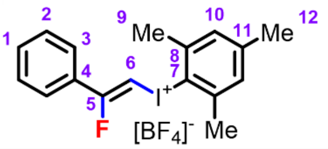

^{1}H NMR (500 MHz, CD_3CN):

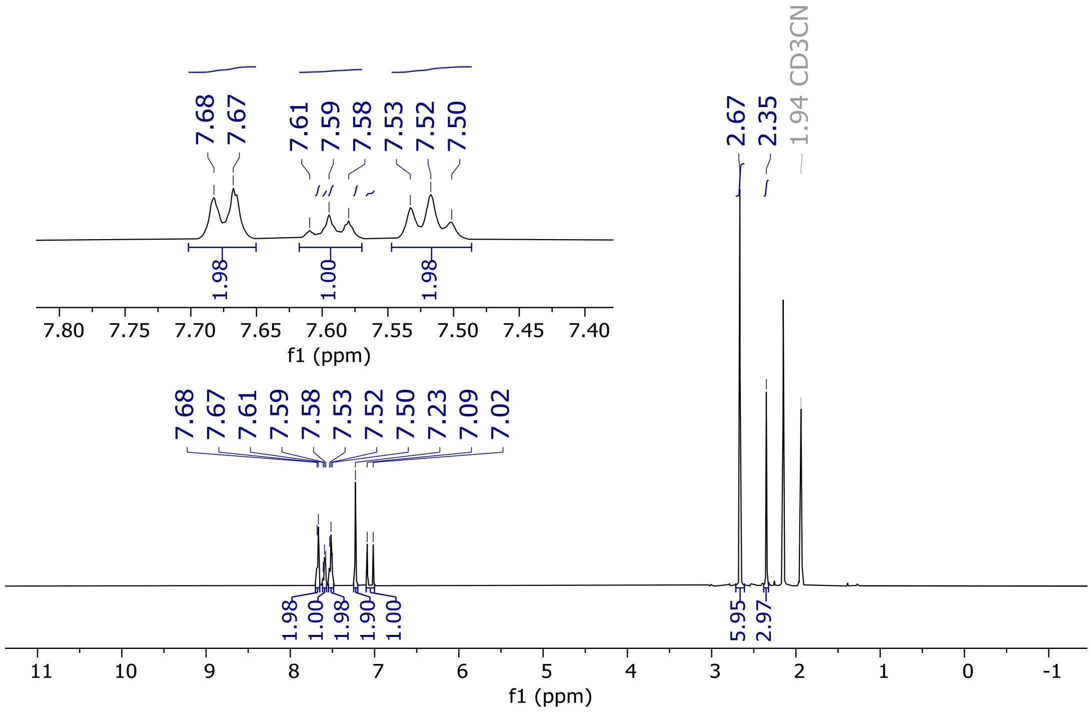

^{13}C NMR (126 MHz, CD_3CN):

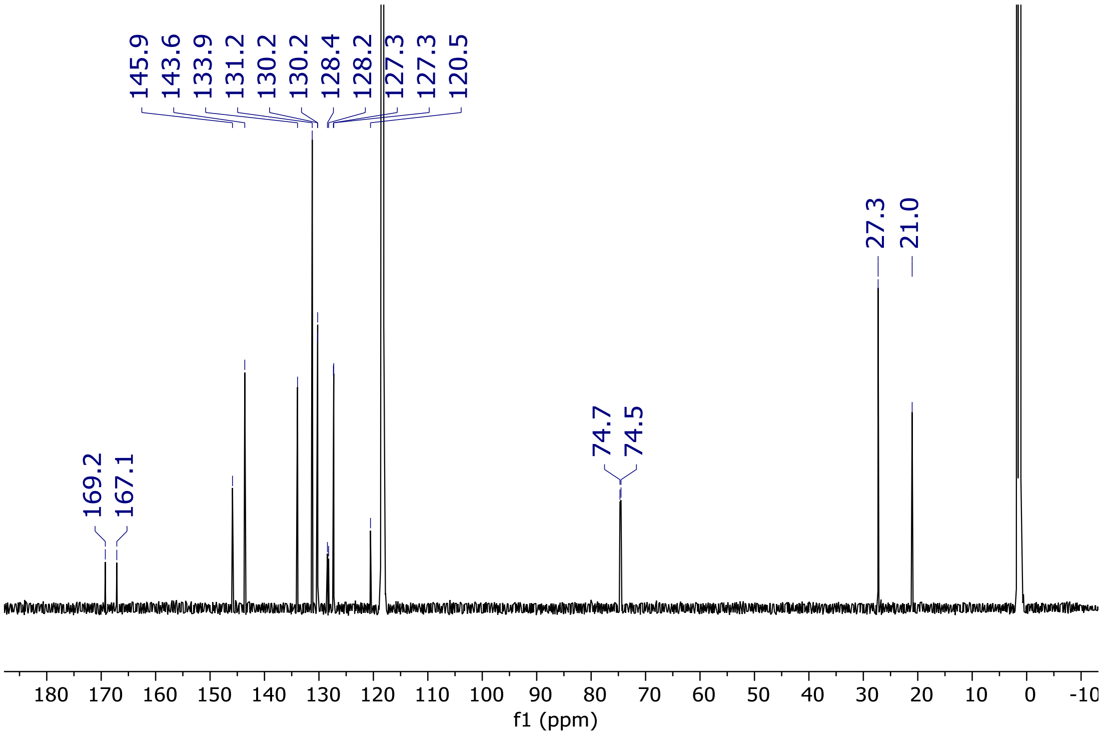

^{19}F NMR (376 MHz, CD_3CN):

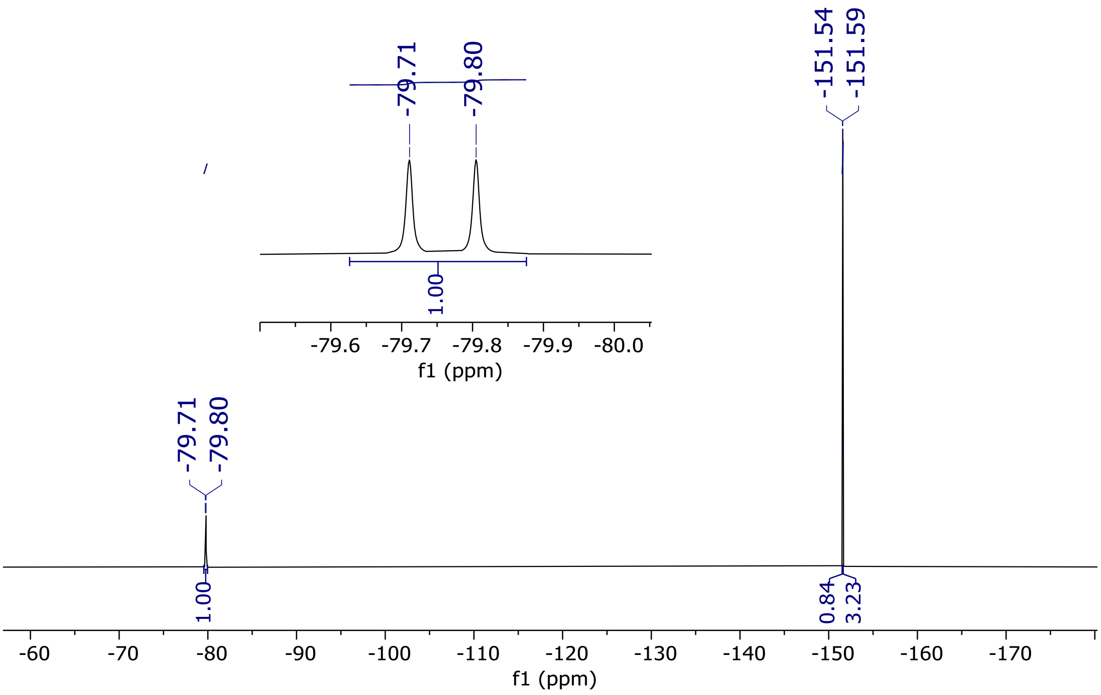

(Z)-(2-fluorododec-1-en-1-yl)(mesityl)iodonium BF_4 ***(3c)***

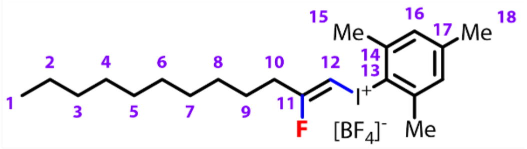

^{1}H NMR (500 MHz, $CDCl_3$):

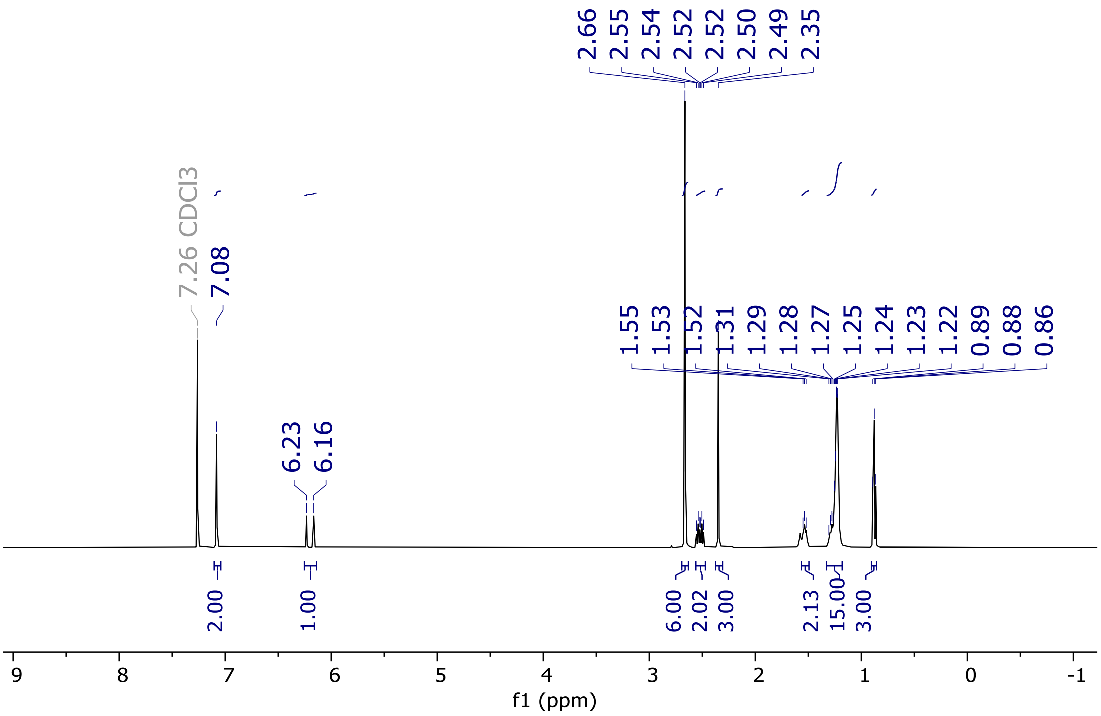

^{13}C NMR (126 MHz, $CDCl_3$):

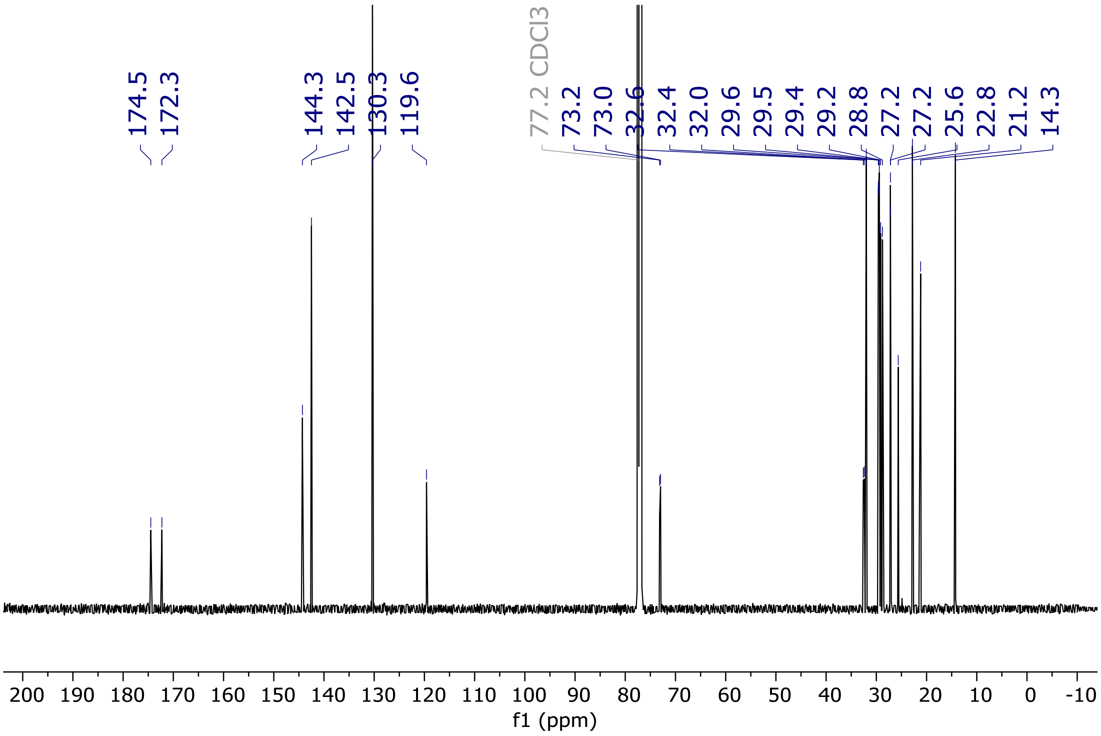

^{19}F NMR (377 MHz, $CDCl_3$):

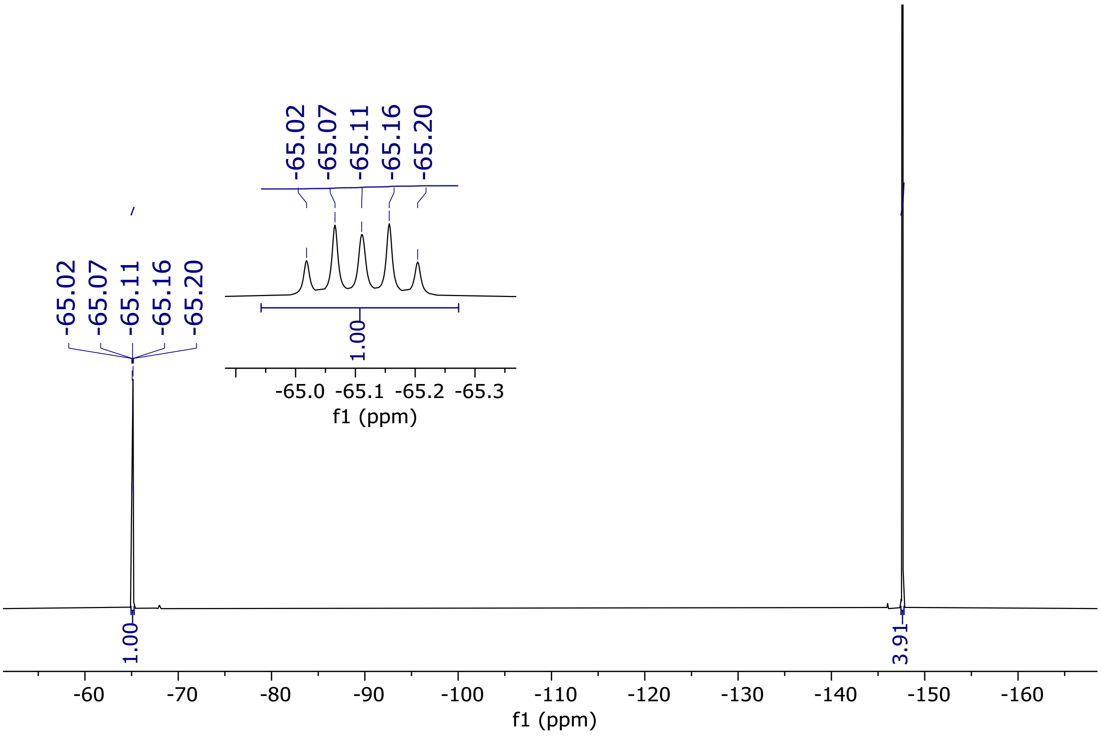

(Z)-(2-cyclohexyl-2-fluorovinyl)(mesityl)iodonium BF_4 **(3d)**

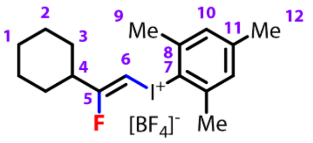

¹H NMR (500 MHz, CDCl₃):

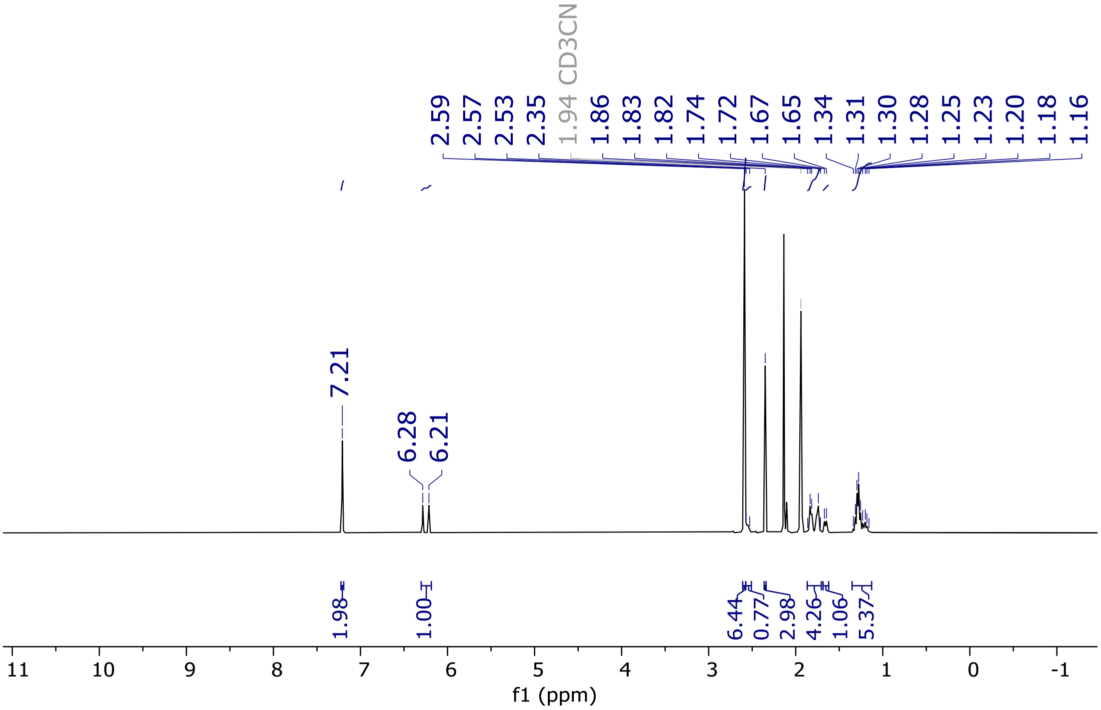

¹³C NMR (126 MHz, CDCl₃):

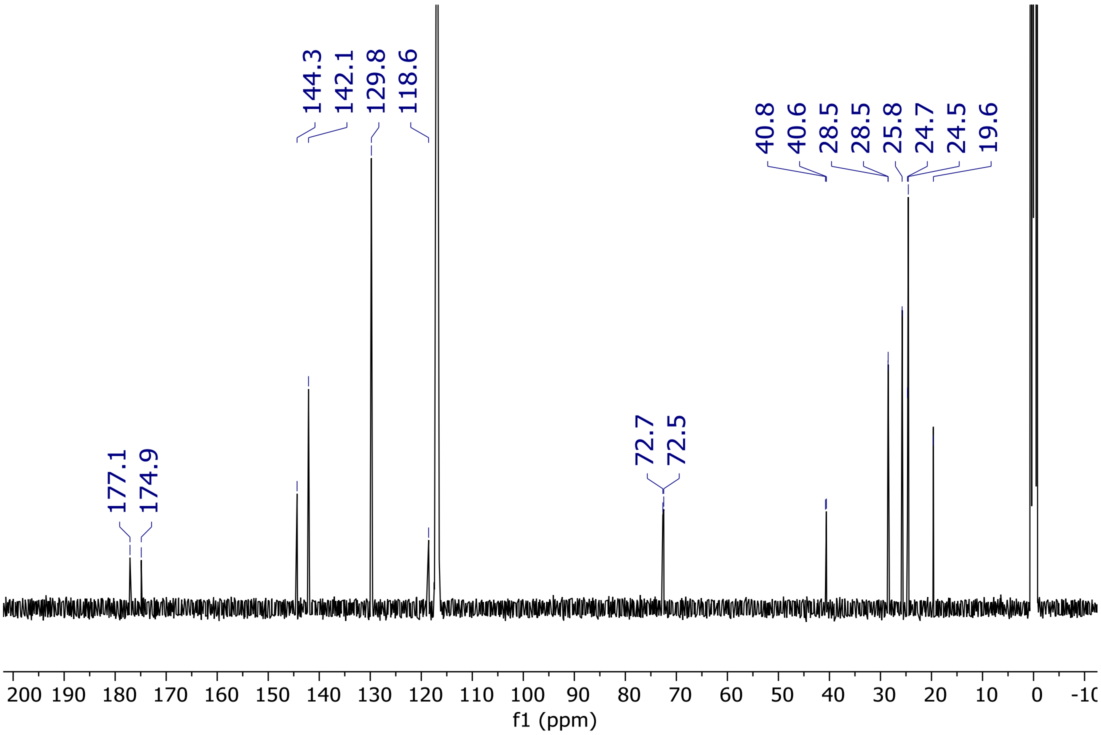

^{19}F NMR (377 MHz, $CDCl_3$):

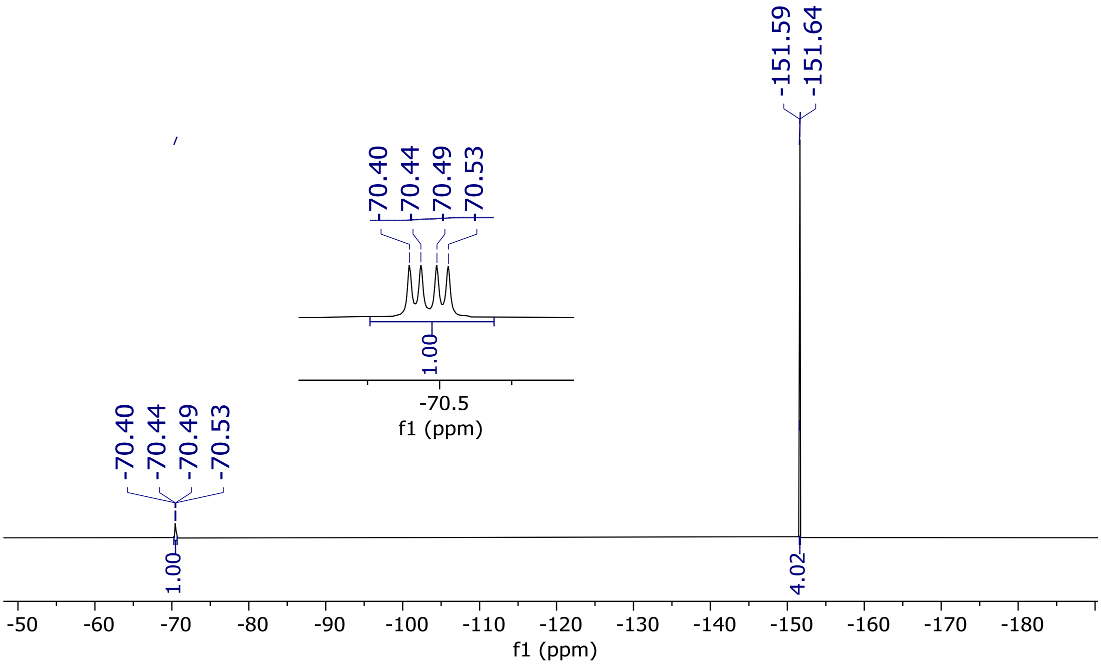

(Z)-(2-fluoro-3,3-dimethylbut-1-en-1-yl)(mesityl)iodonium BF_4 ***(3e)***

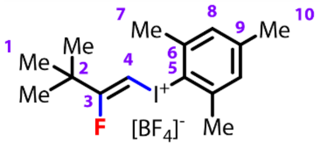

^{1}H NMR (500 MHz, CD_3CN):

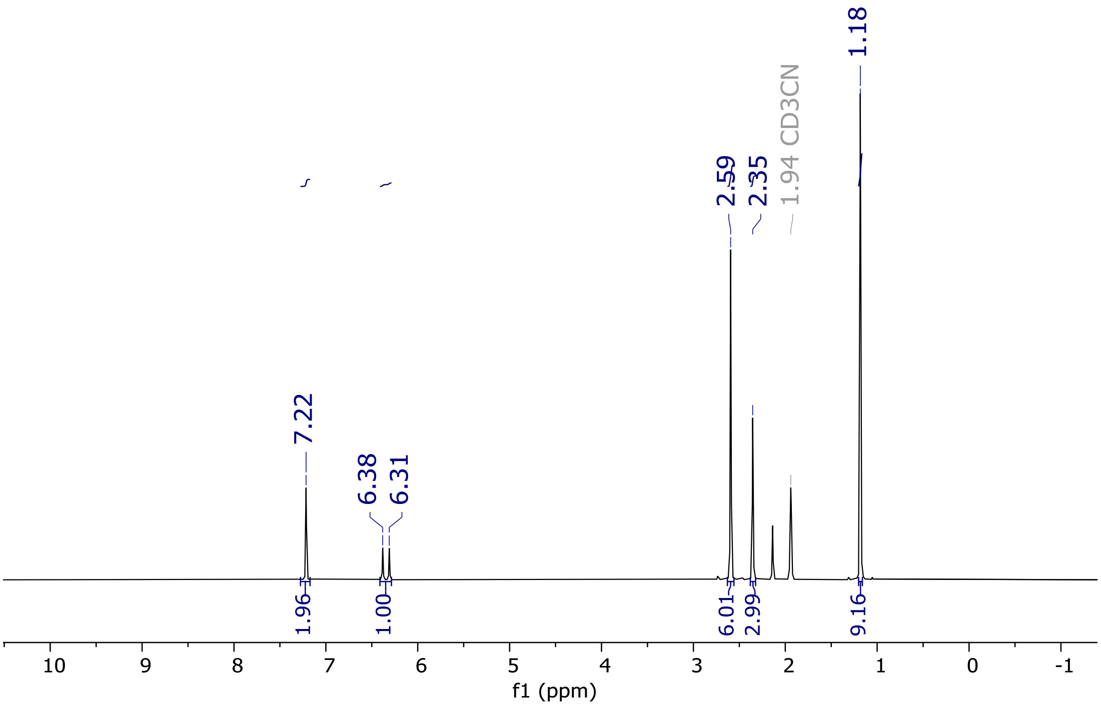

^{13}C NMR (126 MHz, CD_3CN):

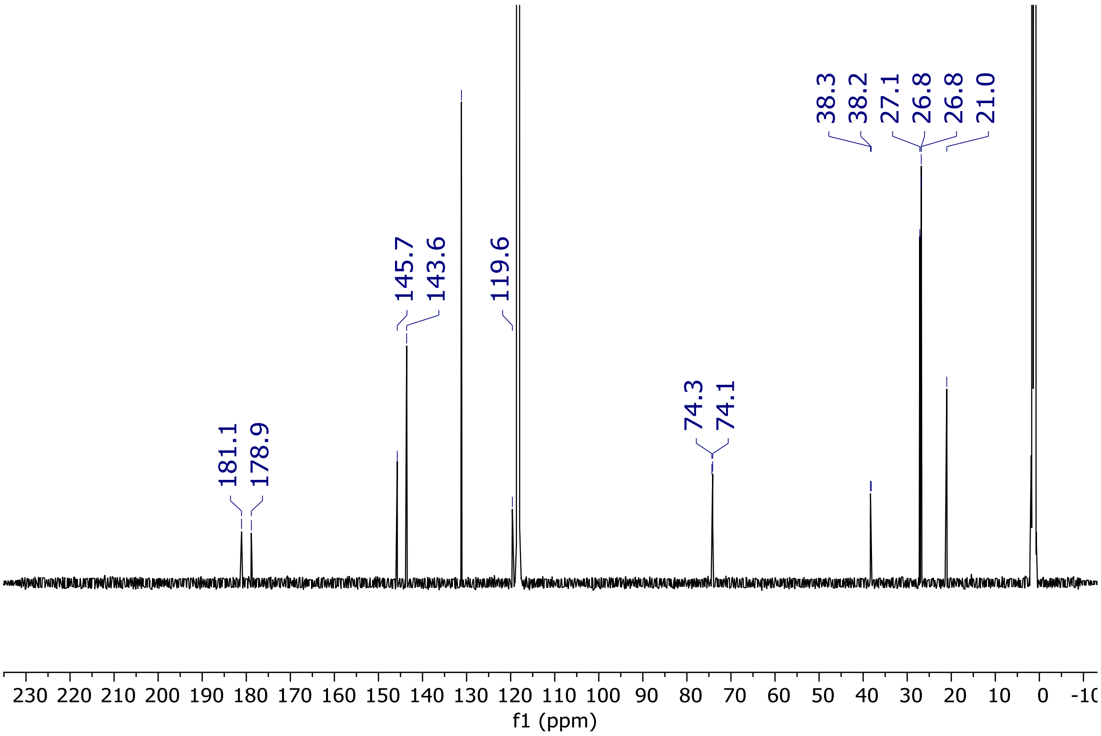

^{19}F NMR (376 MHz, CD_3CN):

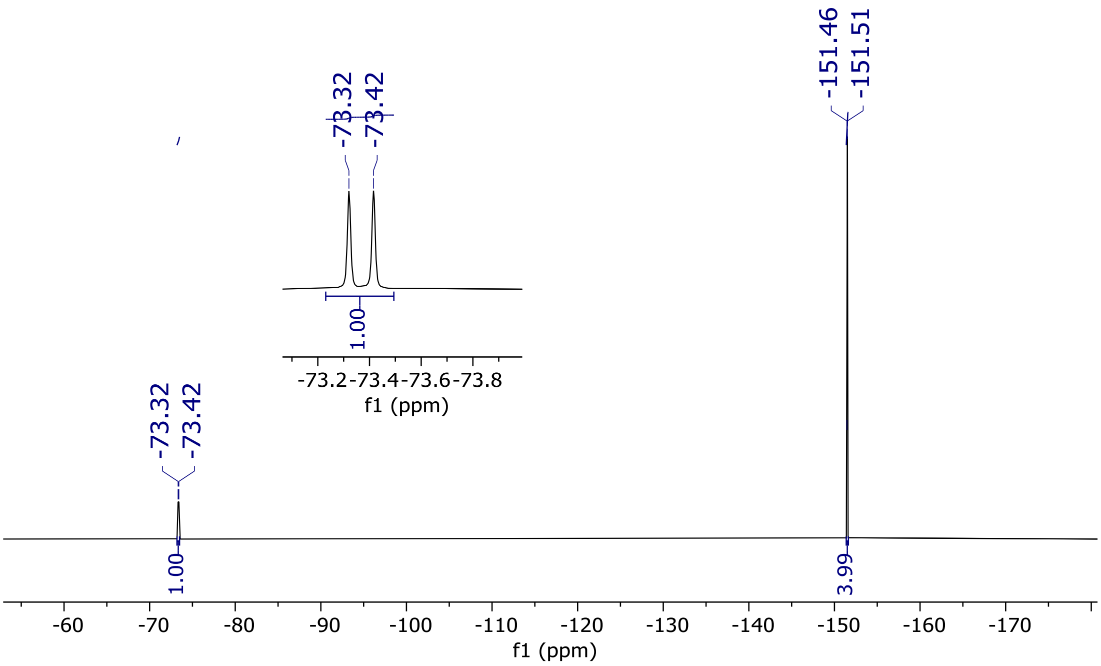

(Z)-(4-bromo-2-fluorobut-1-en-1-yl)(mesityl)iodonium BF_4 ***(3f)***

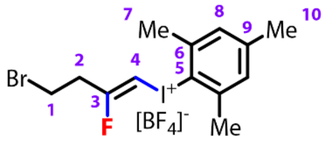

^{1}H NMR (500 MHz, CD_3CN):

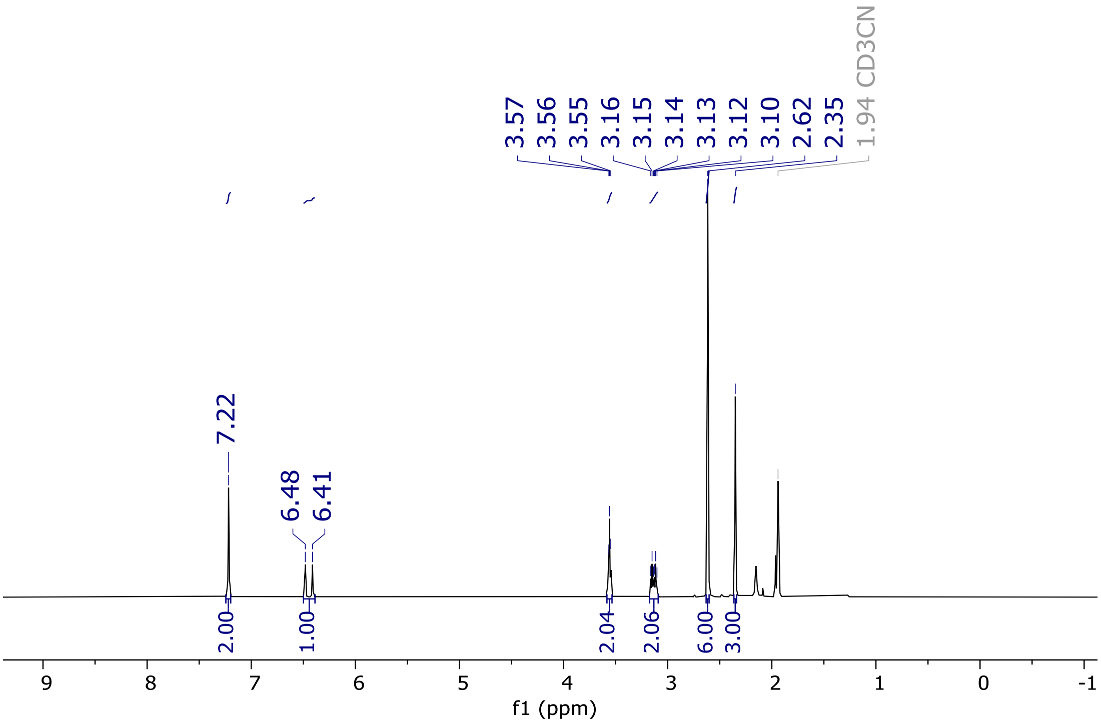

^{13}C NMR (126 MHz, CD_3CN):

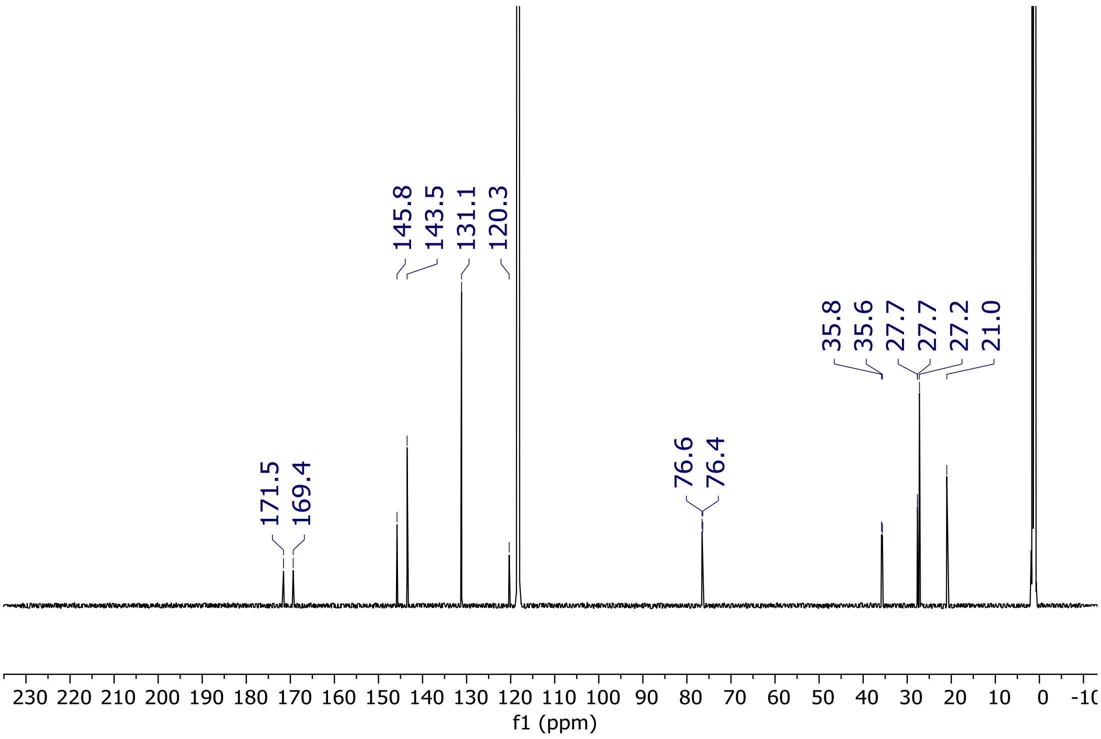

^{19}F NMR (376 MHz, CD_3CN):

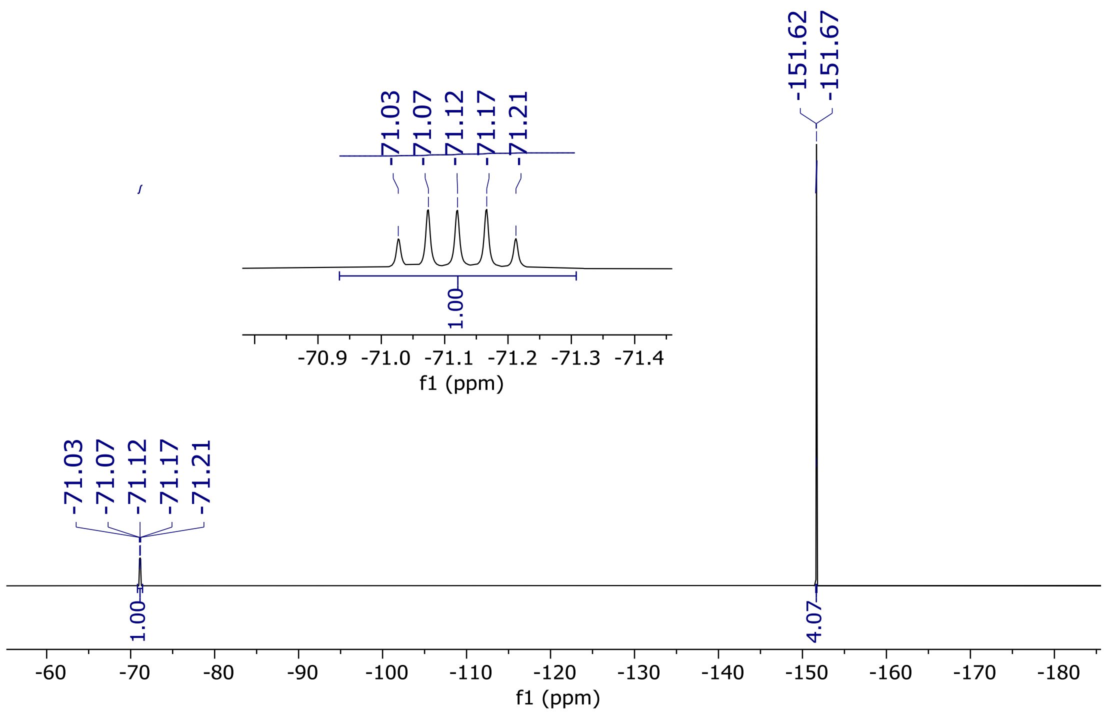

(Z)-(4-(benzyloxy)-2-fluorobut-1-en-1-yl)(mesityl)iodonium BF₄ ***(3g)***

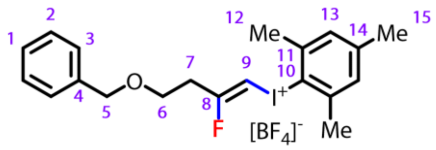

^{1}H NMR (500 MHz, CD3CN):

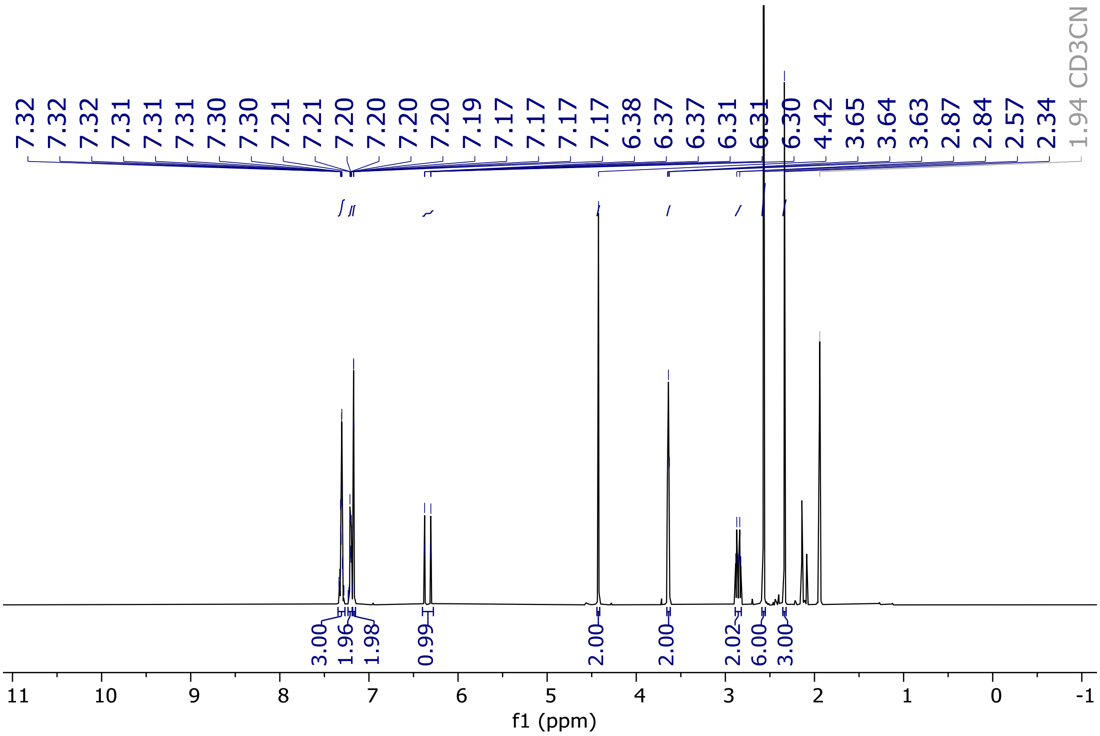

^{13}C NMR (126 MHz, CD3CN):

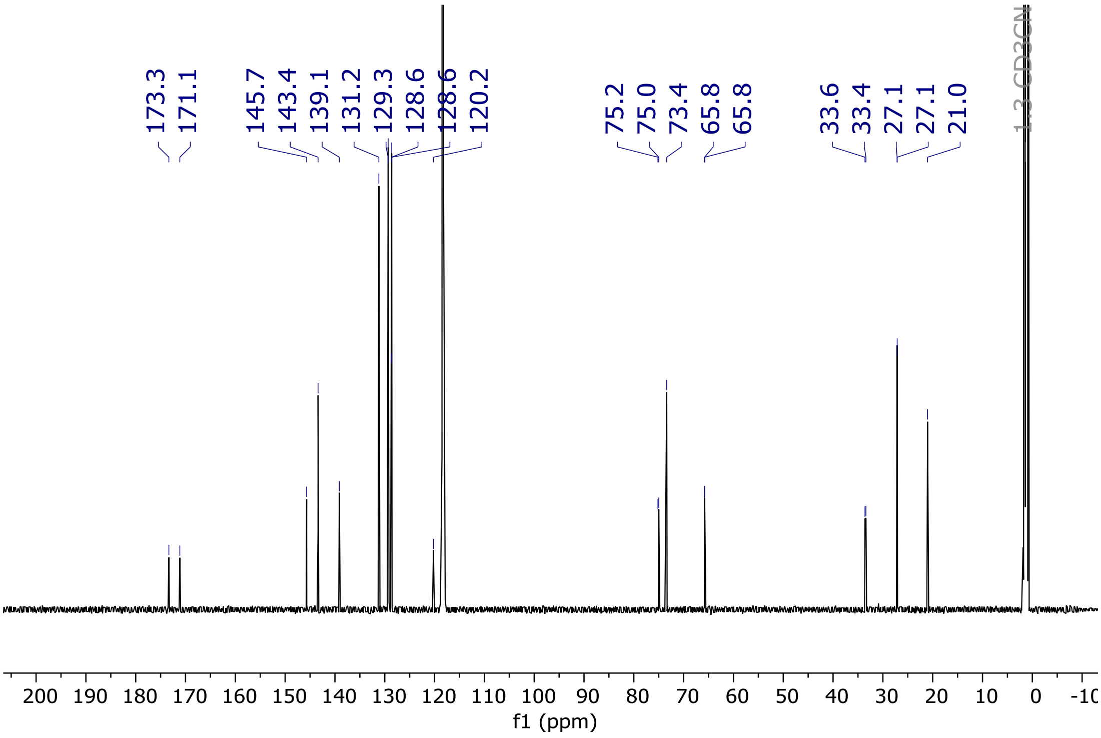

^{19}F NMR (376 MHz, CD_3CN):

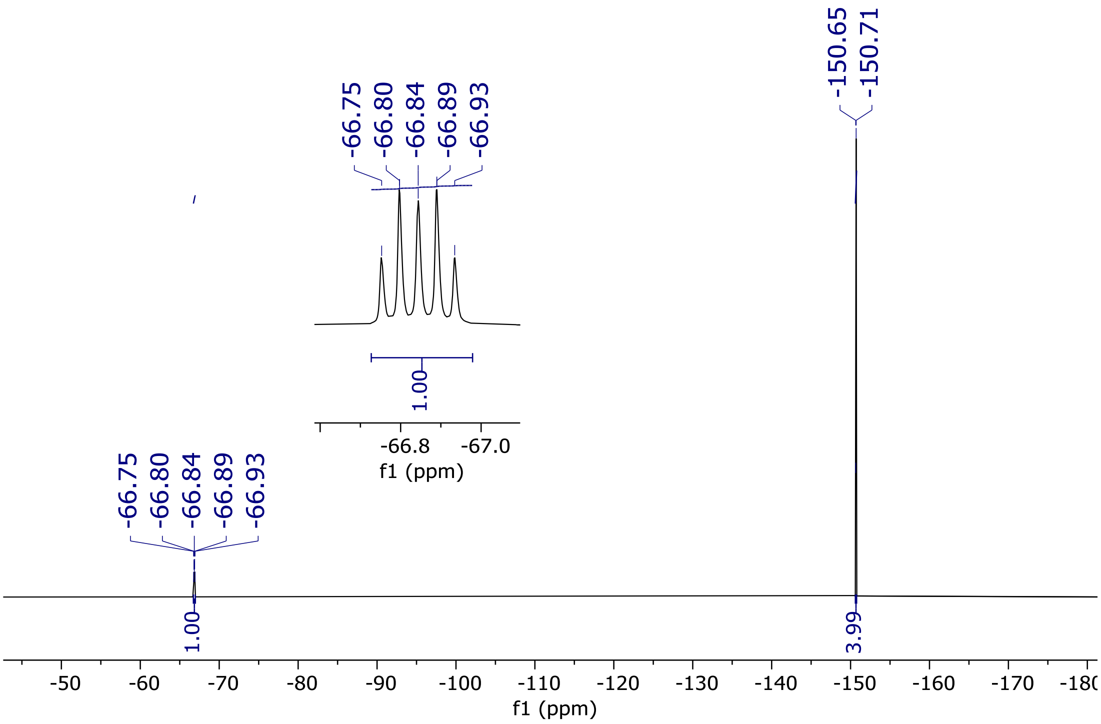

(Z)-(2-fluoro-4-(tosyloxy)but-1-en-1-yl)(mesityl)iodonium BF_4 ***(3h)***

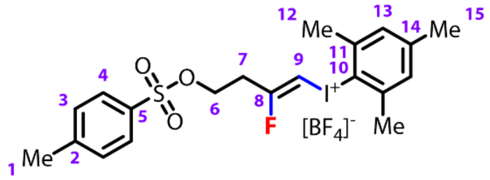

^{1}H NMR (500 MHz, CD_3CN):

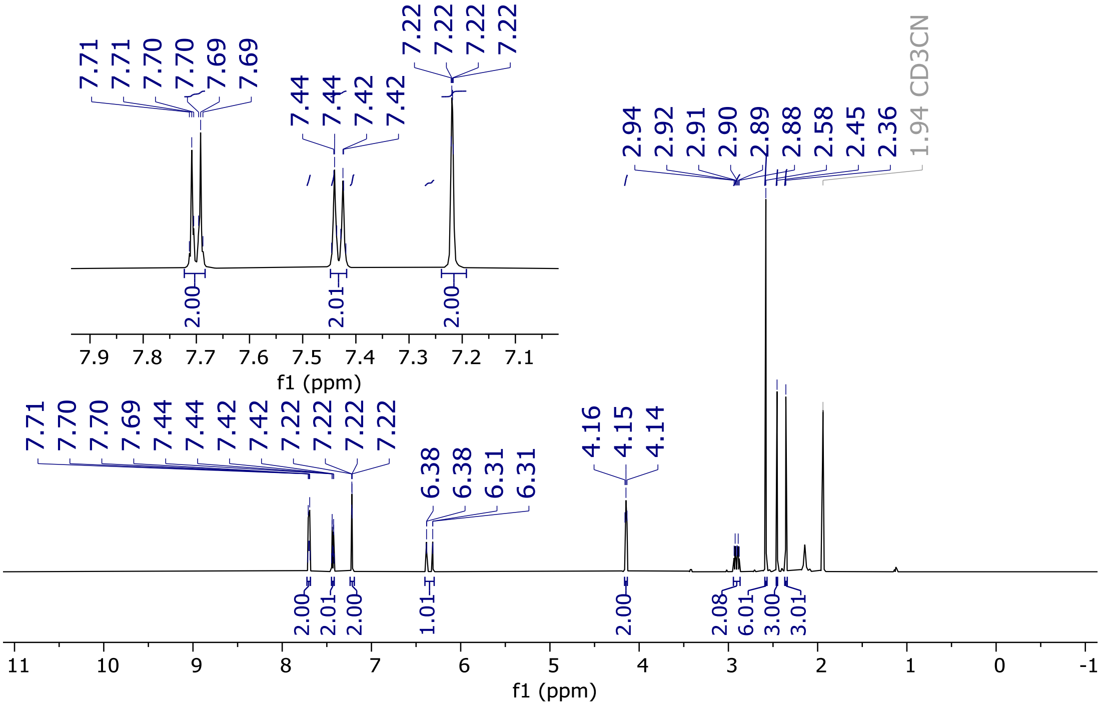

^{13}C NMR (126 MHz, CD_3CN):

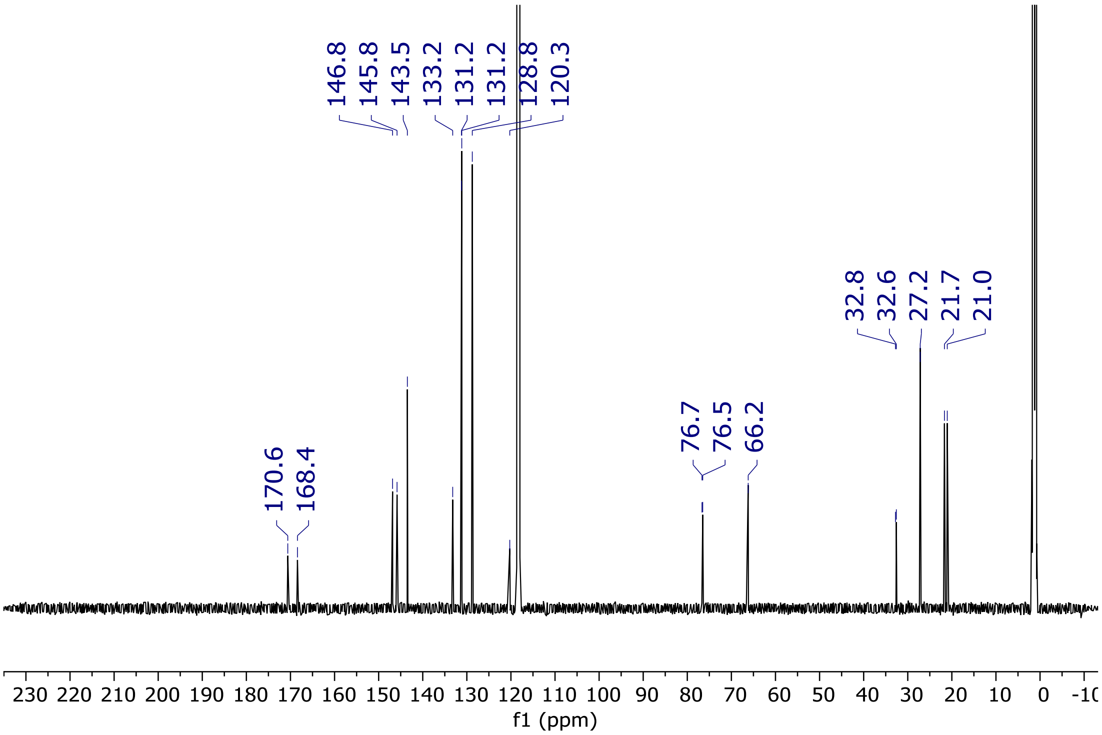

^{19}F NMR (376 MHz, $C(CD_3)_2O$):

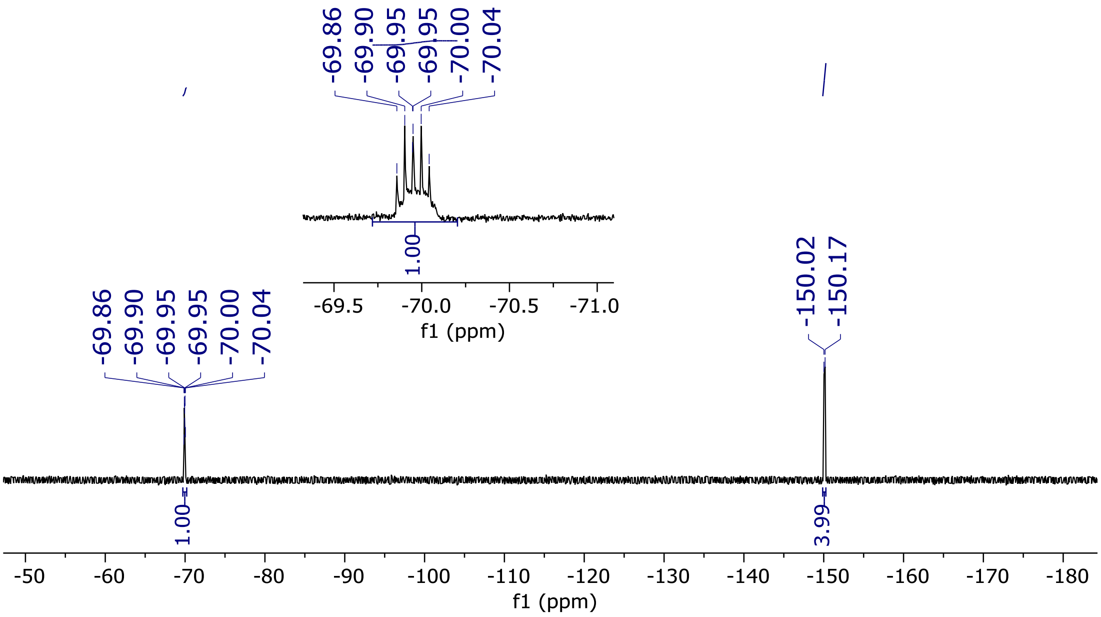

Z)-(3-((4-bromobenzyl)oxy)-2-fluoroprop-1-en-1-yl)(mesityl)iodonium BF_4 **(3i)**

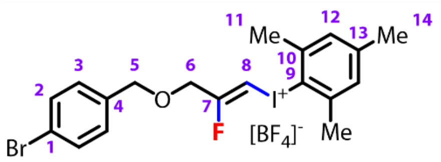

^{1}H NMR (500 MHz, $C(CD_3)_2O$):

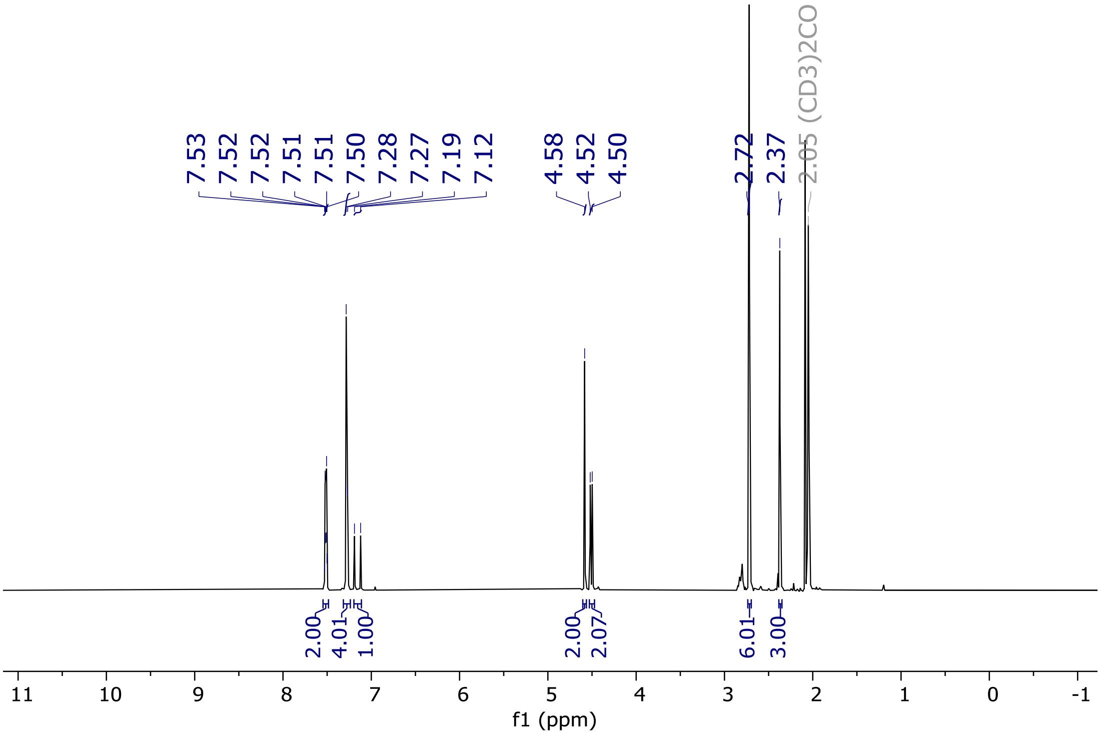

^{13}C NMR (126 MHz, $C(CD_3)_2O$):

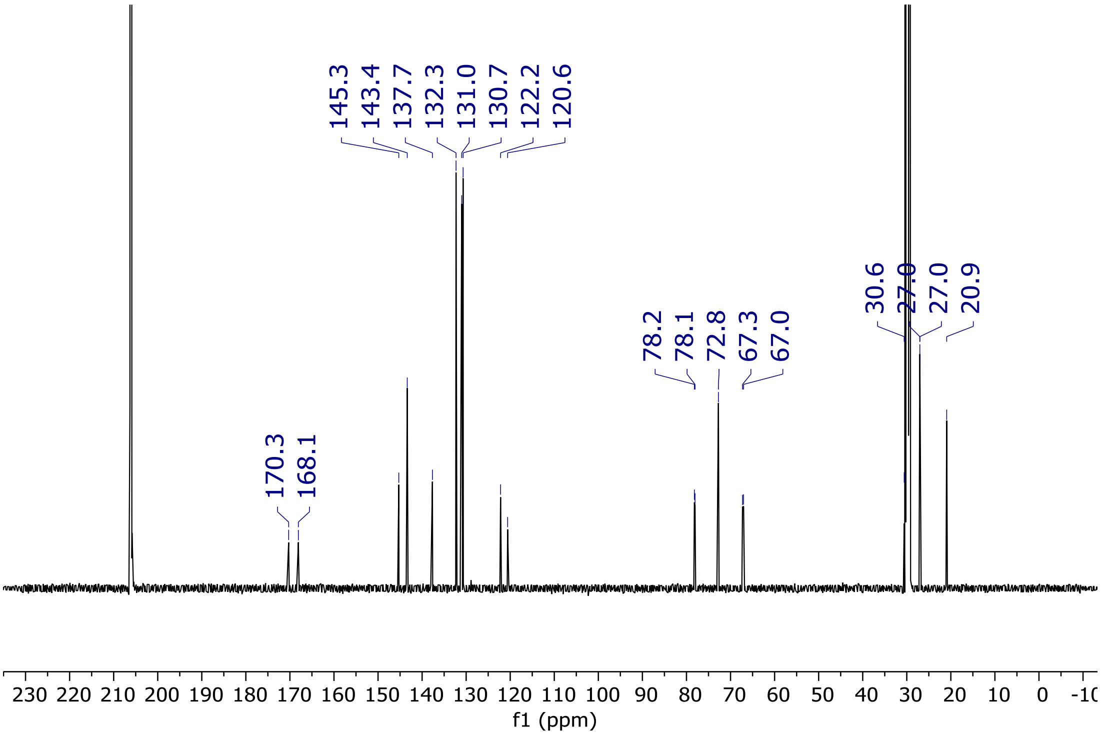

^{19}F NMR (376 MHz, $C(CD_3)_2O$):

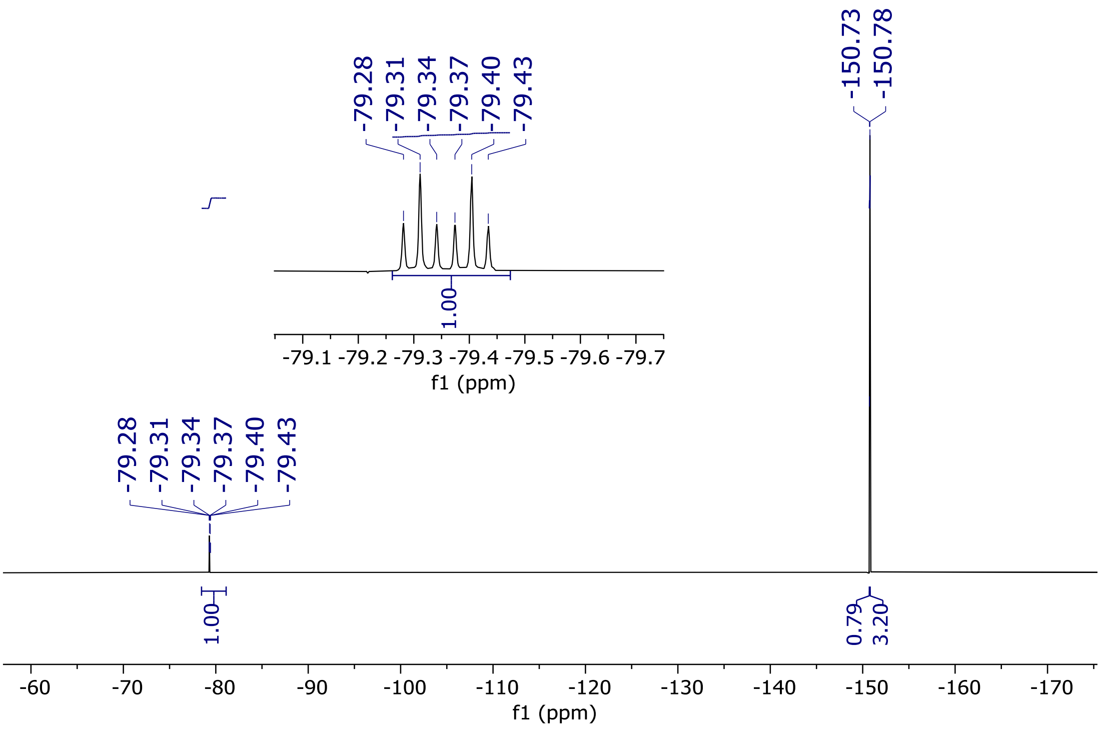

(Z)-(3-chloro-2-fluoroprop-1-en-1-yl)(mesityl)iodonium BF_4 ***(3j)***

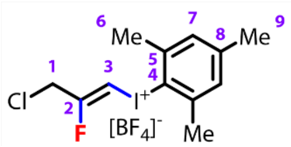

^{1}H NMR (500 MHz, $C(CD_3)_2O$):

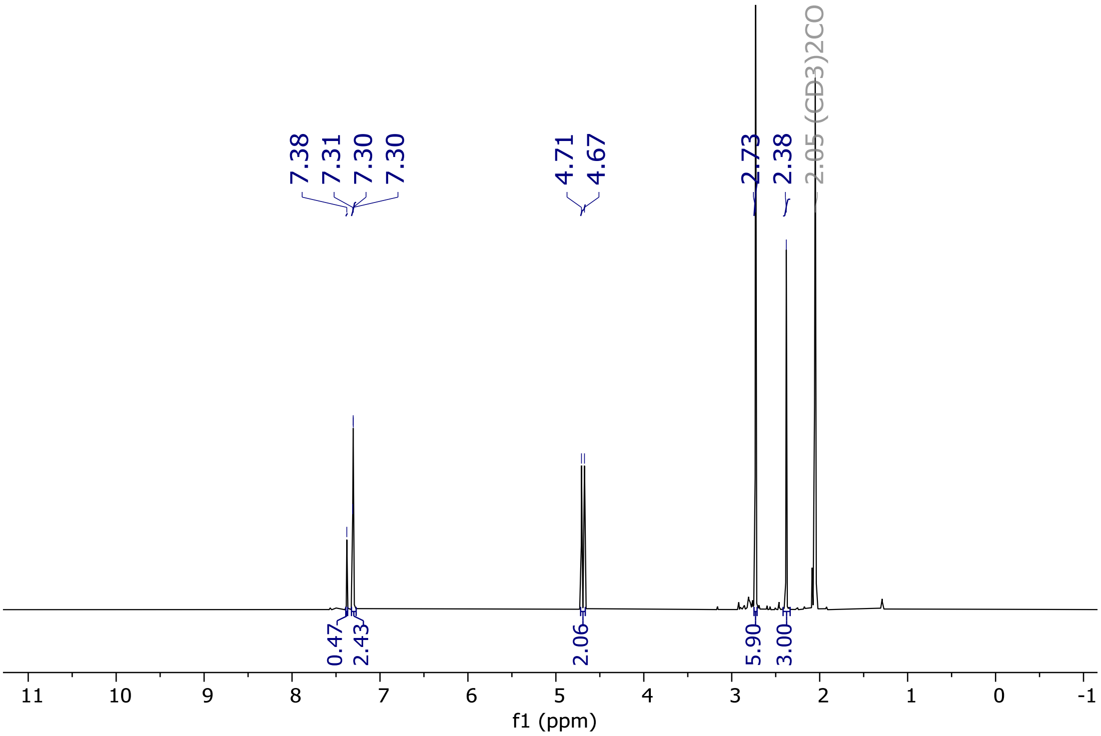

^{13}C NMR (126 MHz, $C(CD_3)_2O$):

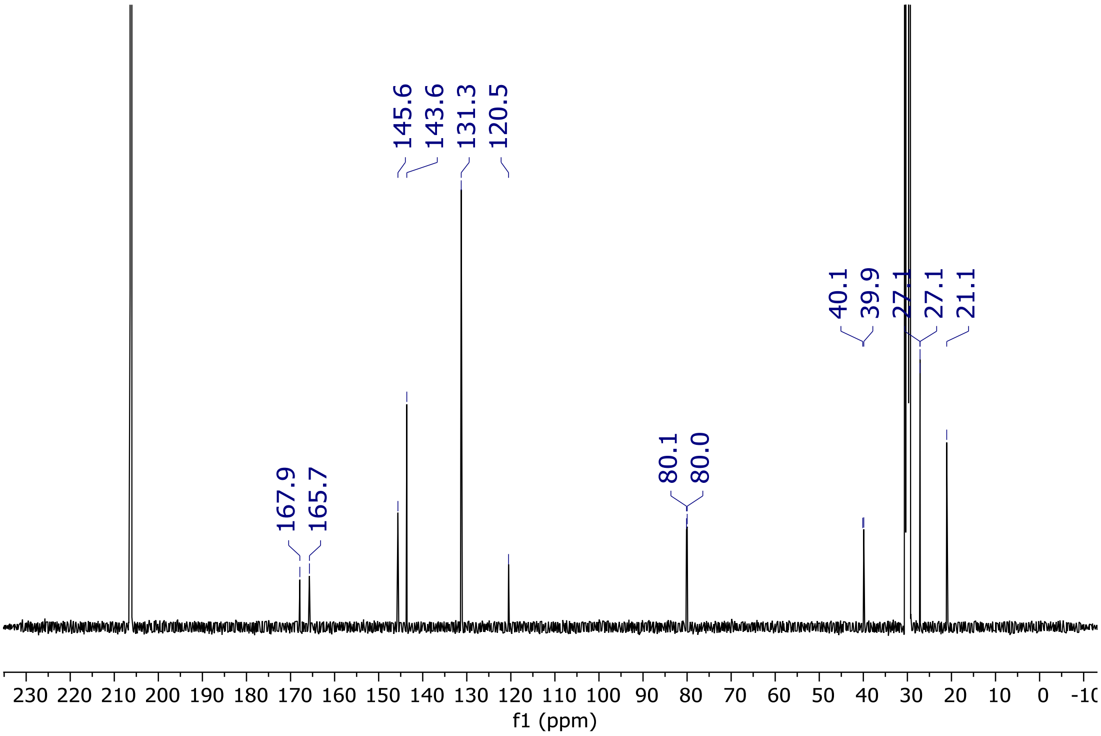

^{19}F NMR (376 MHz, $C(CD_3)_2O$):

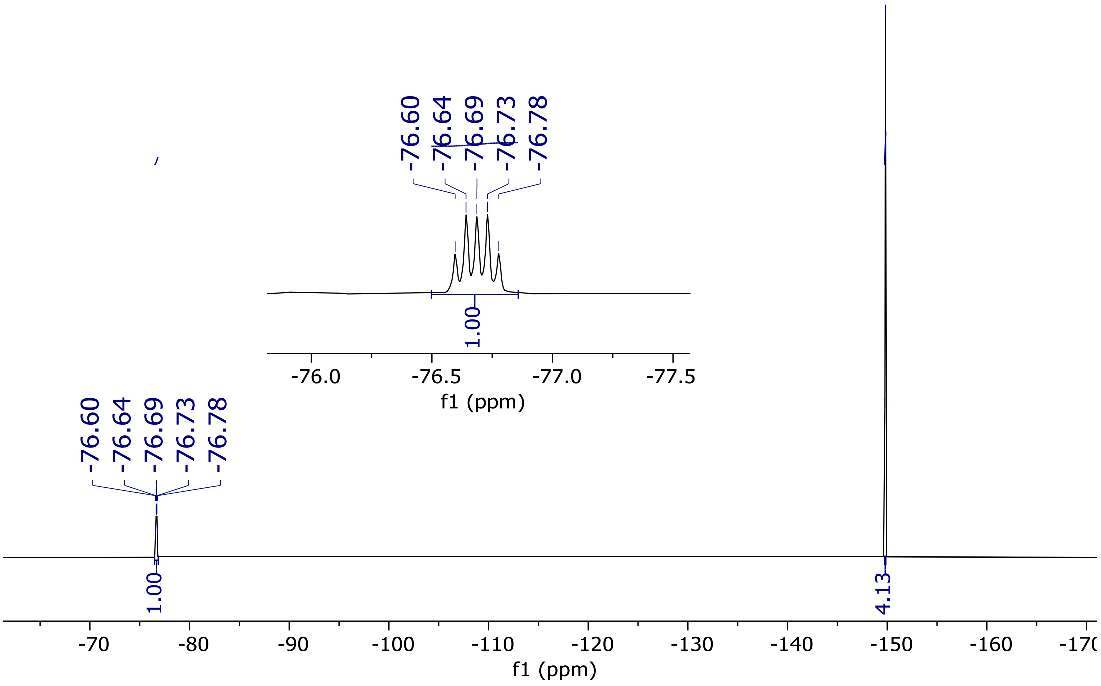

(Z)-(3-bromo-2-fluoroprop-1-en-1-yl)(mesityl)iodonium BF_4 ***(3k)***

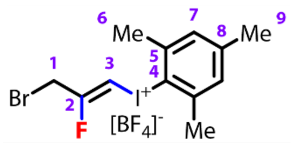

^{1}H NMR (500 MHz, CD_3CN):

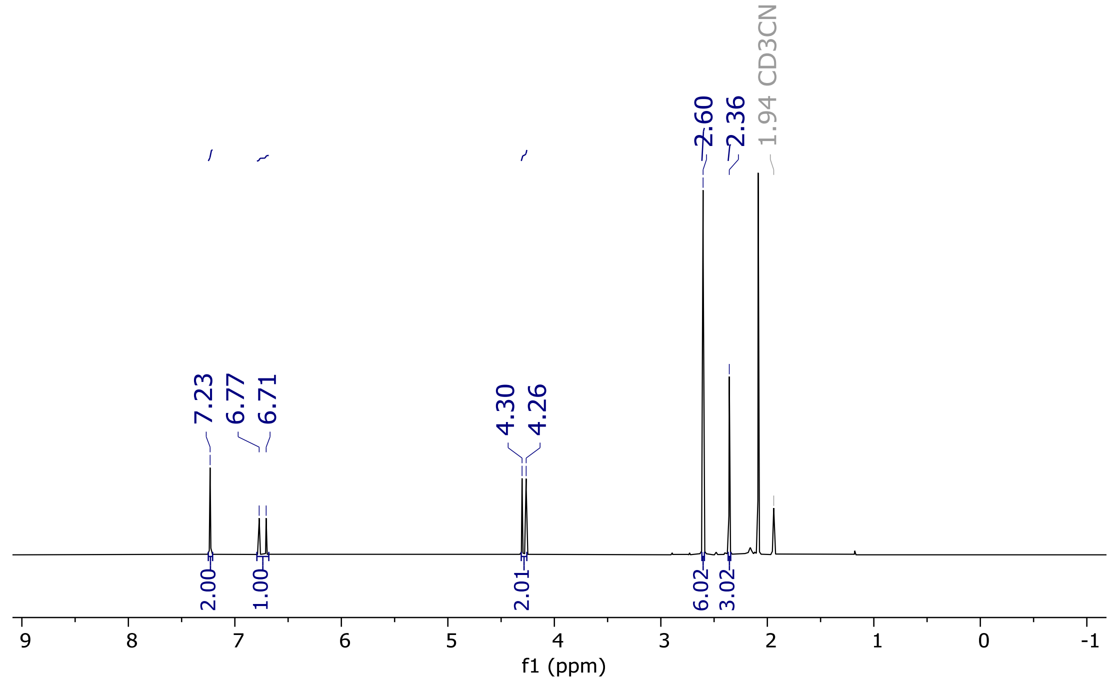

^{13}C NMR (126 MHz, $C(CD_3)_2O$):

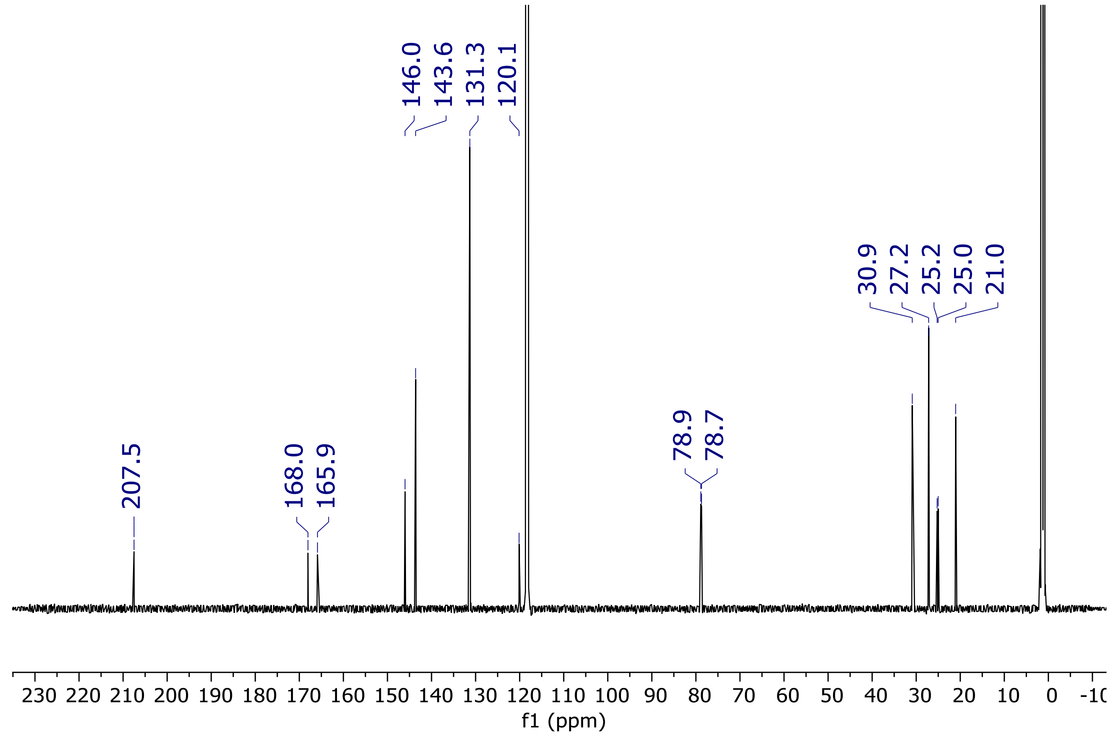

^{19}F NMR (376 MHz, $C(CD_3)_2O$):

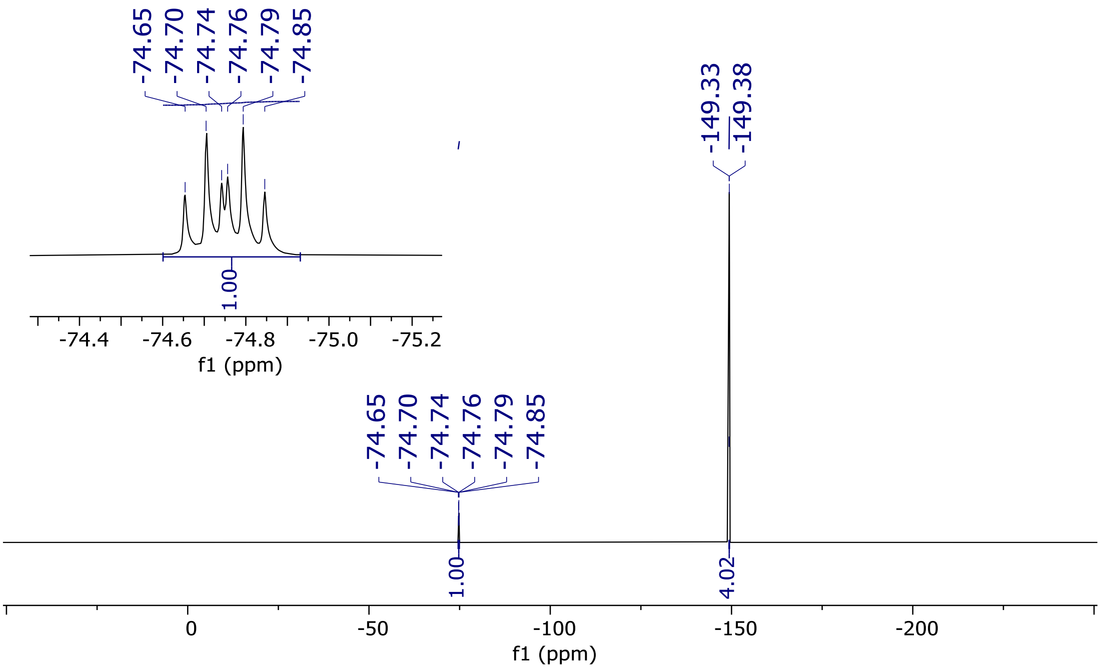

(Z)-(2-fluoro-3-((methylsulfonyl)oxy)prop-1-en-1-yl)(mesityl)iodonium BF_4 ***(3l)***

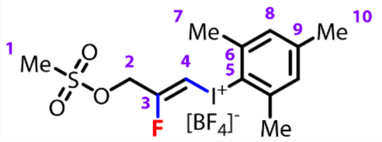

^{1}H NMR (400 MHz, $C(CD_3)_2O$):

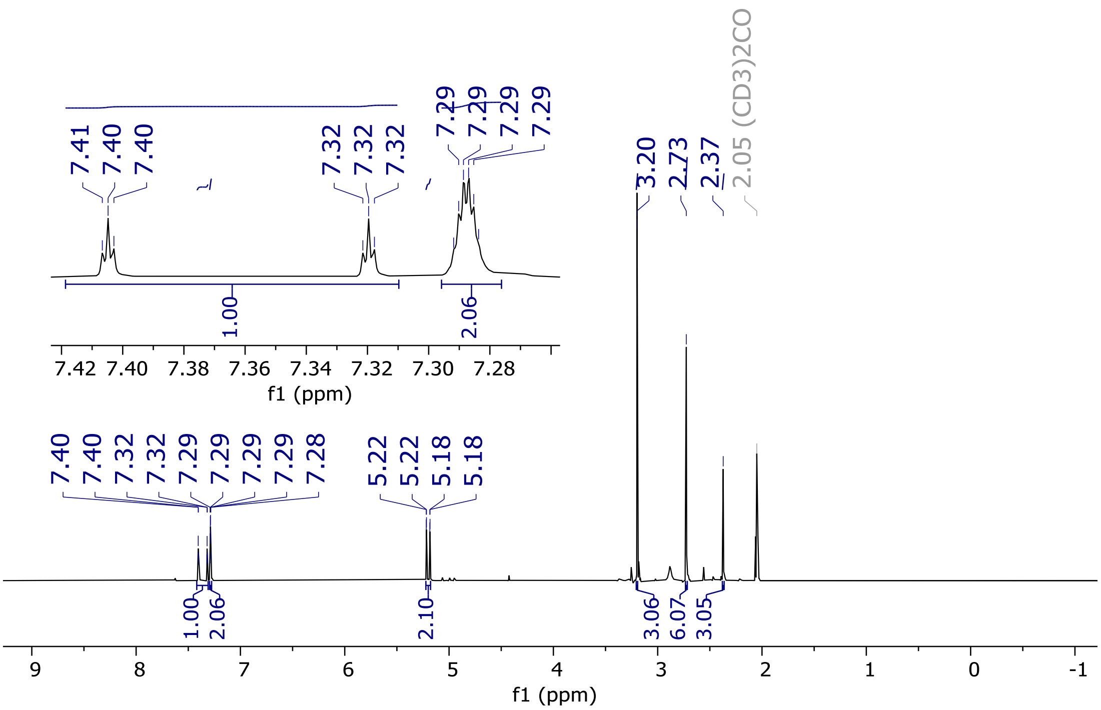

^{13}C NMR (126 MHz, $C(CD_3)_2O$):

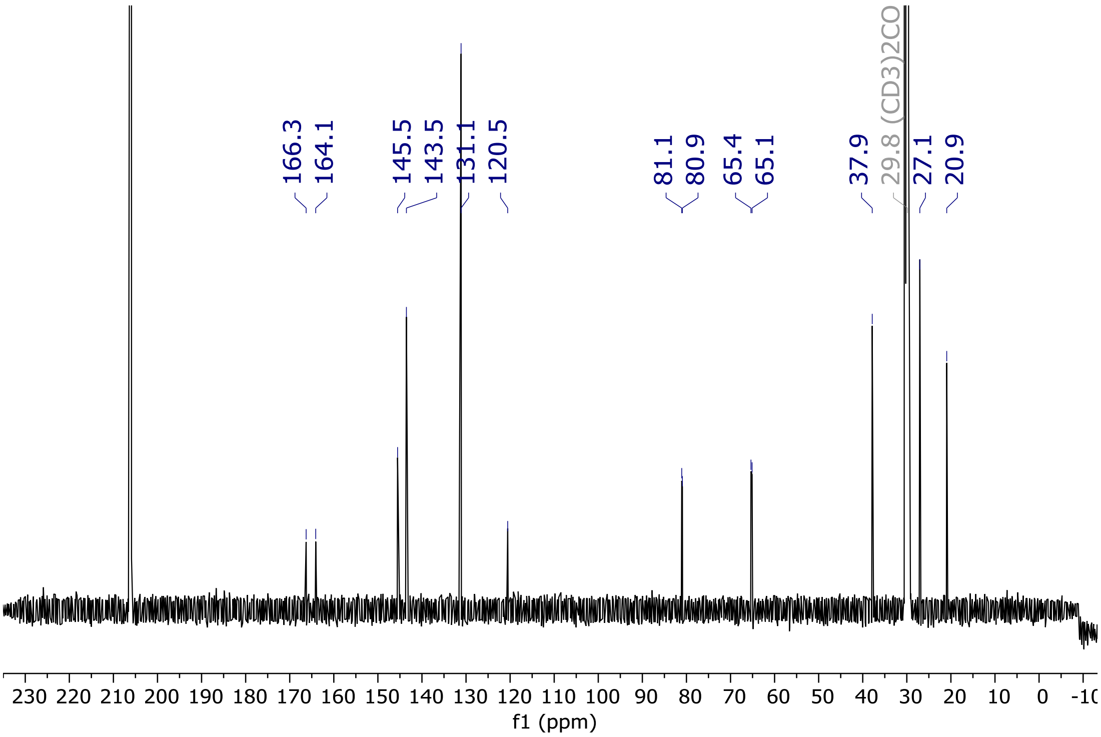

^{19}F NMR (376 MHz, $C(CD_3)_2O$):

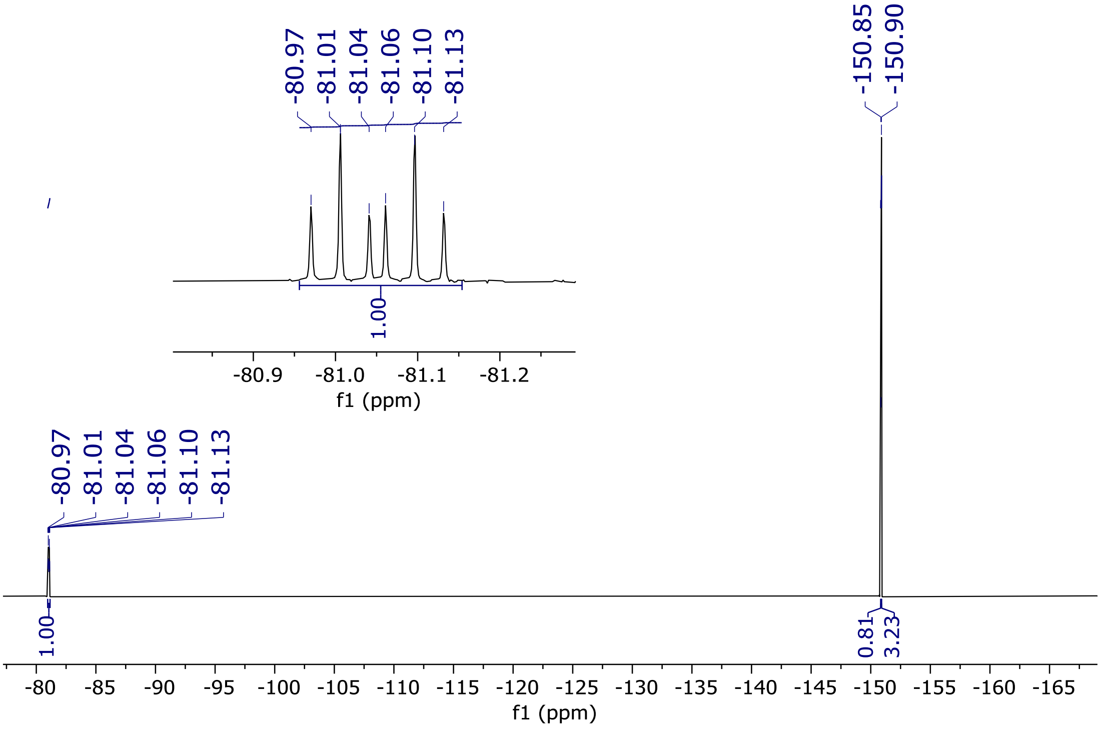

<u>(Z)-(2-fluoro-3-(tosyloxy)prop-1-en-1-yl)(mesityl)iodonium BF_4</u> ***<u>(3m)</u>***

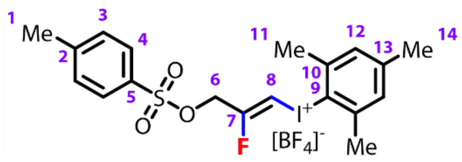

^{1}H NMR (400 MHz, $C(CD_3)_2O$):

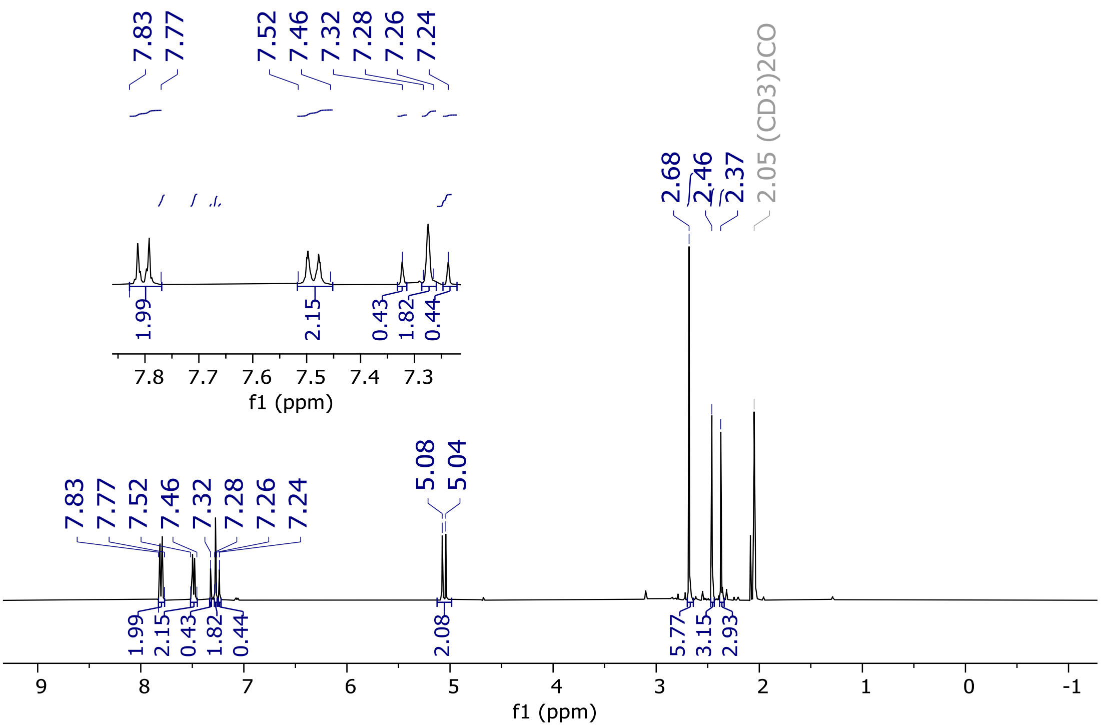

^{13}C NMR (126 MHz, $C(CD_3)_2O$):

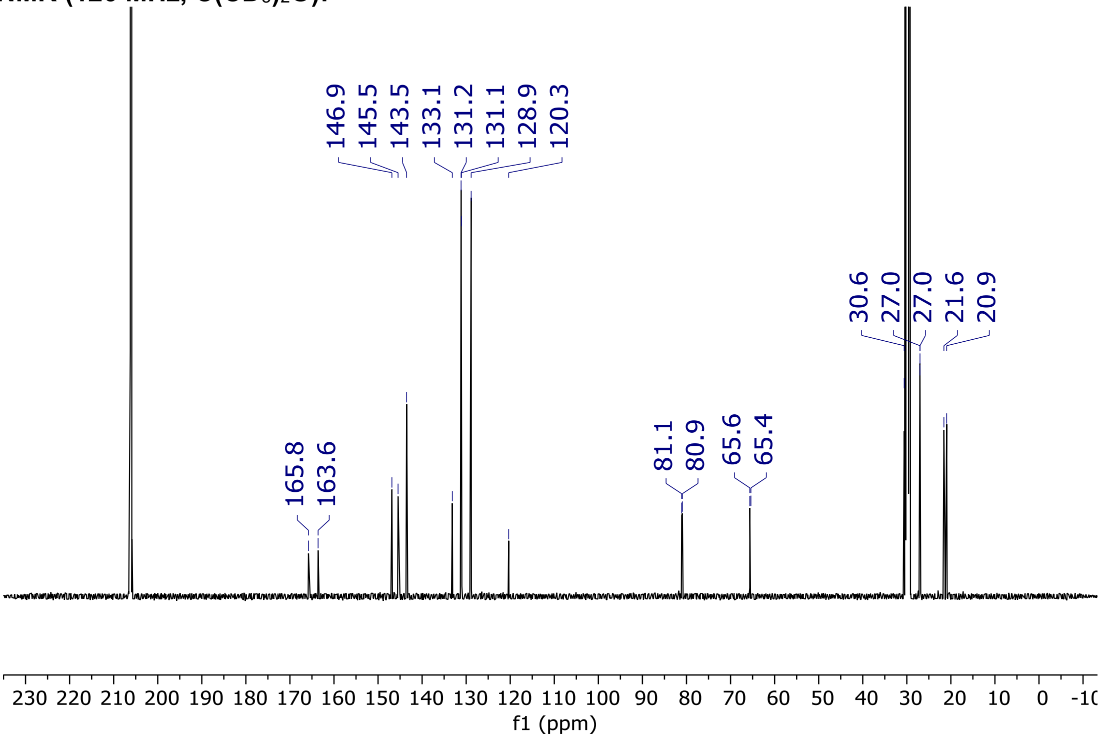

^{19}F NMR (376 MHz, $C(CD_3)_2O$):

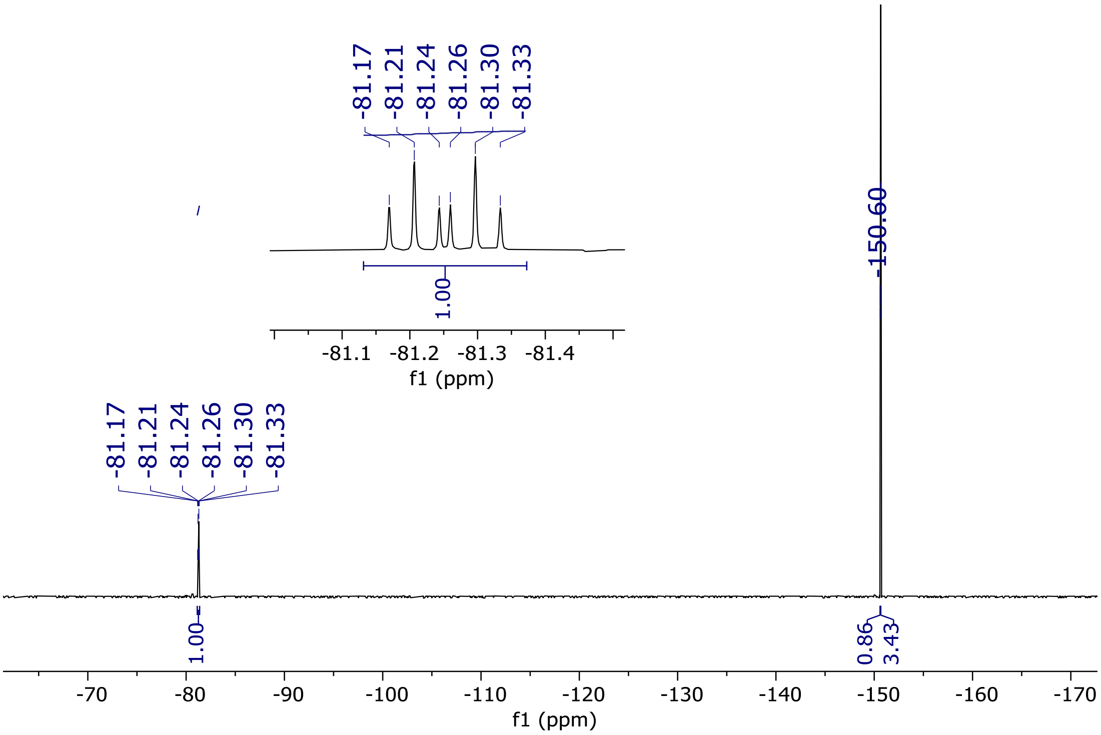

(Z)-(2-fluoro-3-(4-nitrophenoxy)prop-1-en-1-yl)(mesityl)iodonium BF_4 ***(3n)***

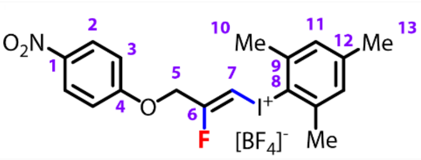

^{1}H NMR (500 MHz, CD_3CN):

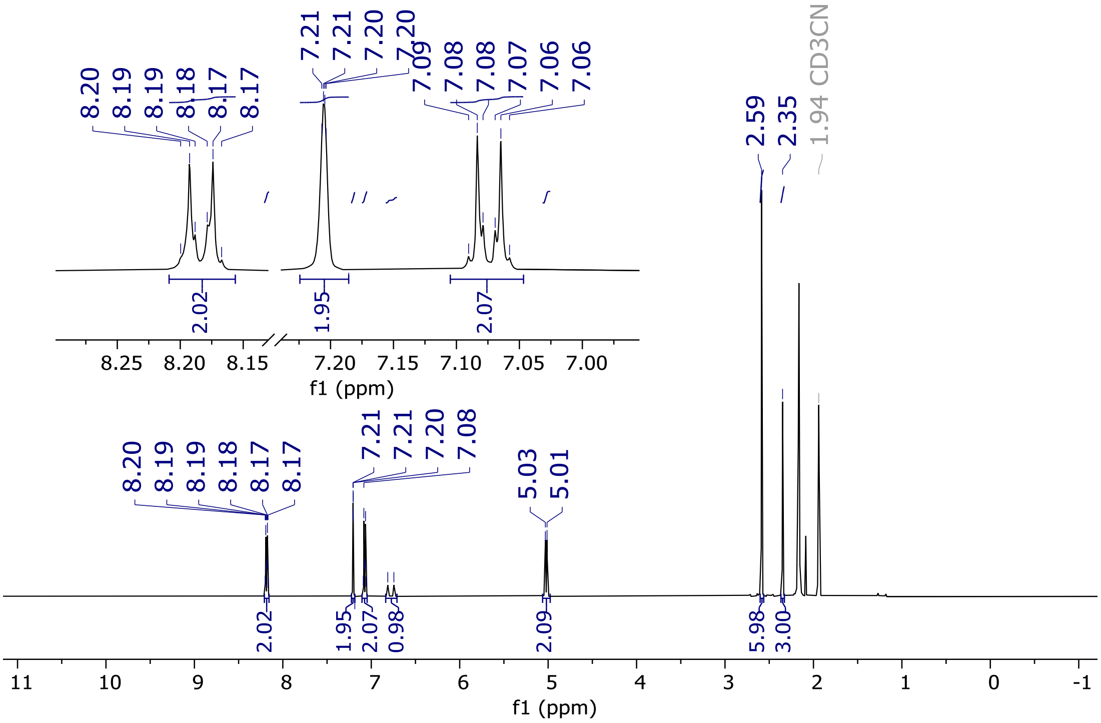

^{13}C NMR (126 MHz, CD_3CN):

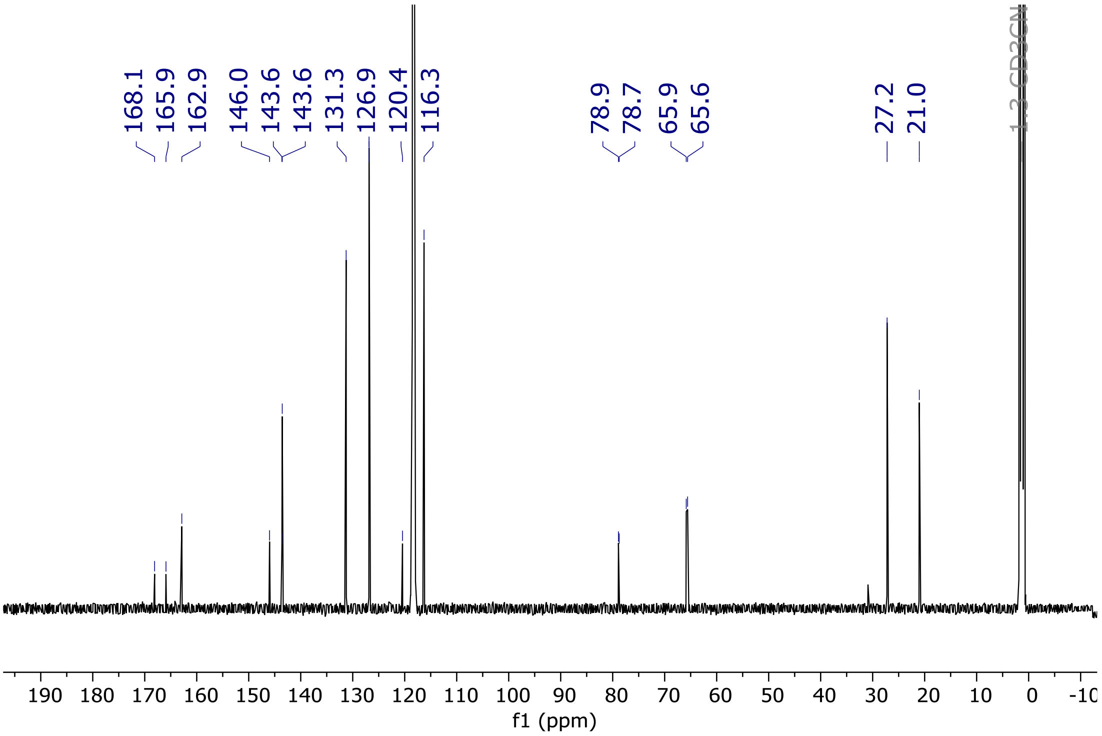

^{19}F NMR (376 MHz, $C(CD_3)_2O$):

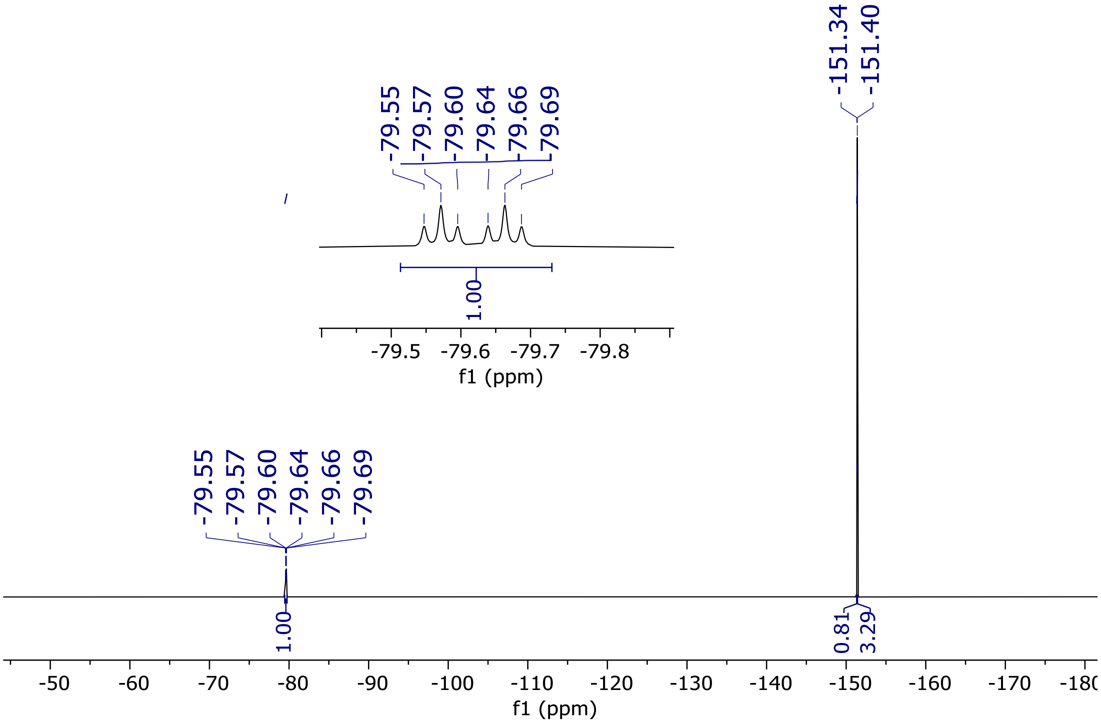

(Z)-(2-fluoro-3-(4-fluorophenoxy)prop-1-en-1-yl)(mesityl)iodonium *BF_4* ***(3o)***

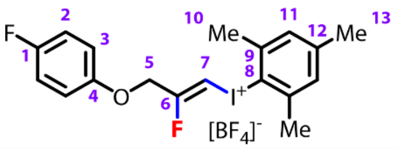

^1H NMR (500 MHz, $C(CD_3)_2O$):

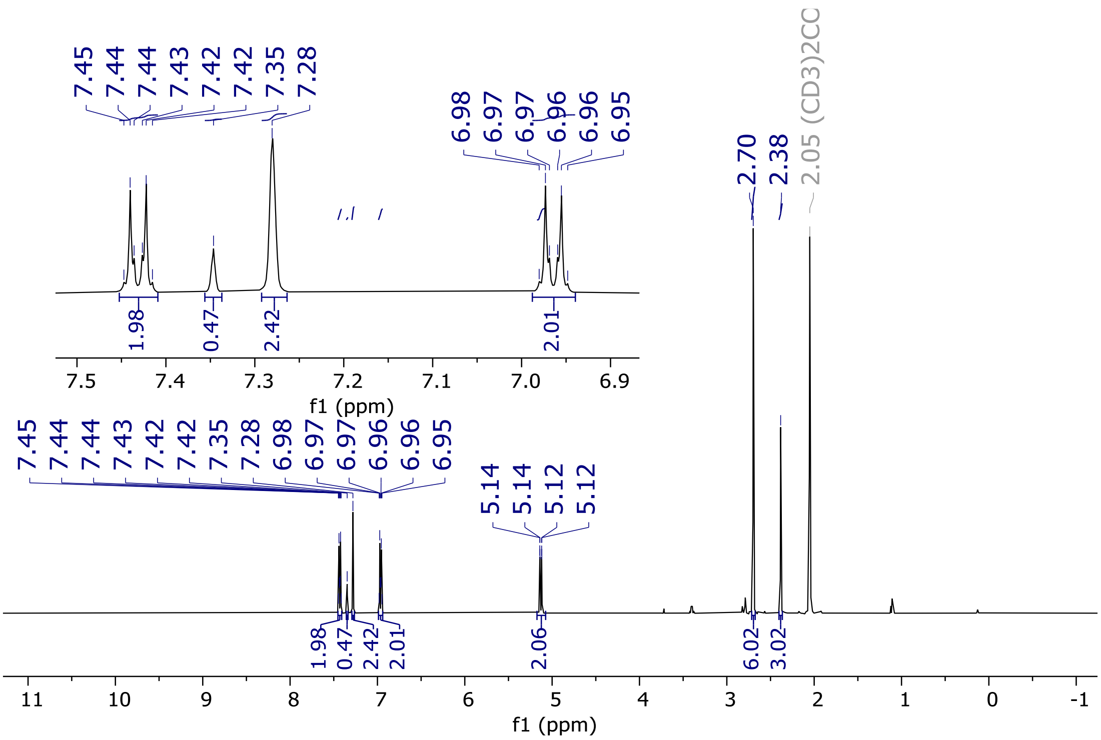

^{13}C NMR (126 MHz, $C(CD_3)_2O$):

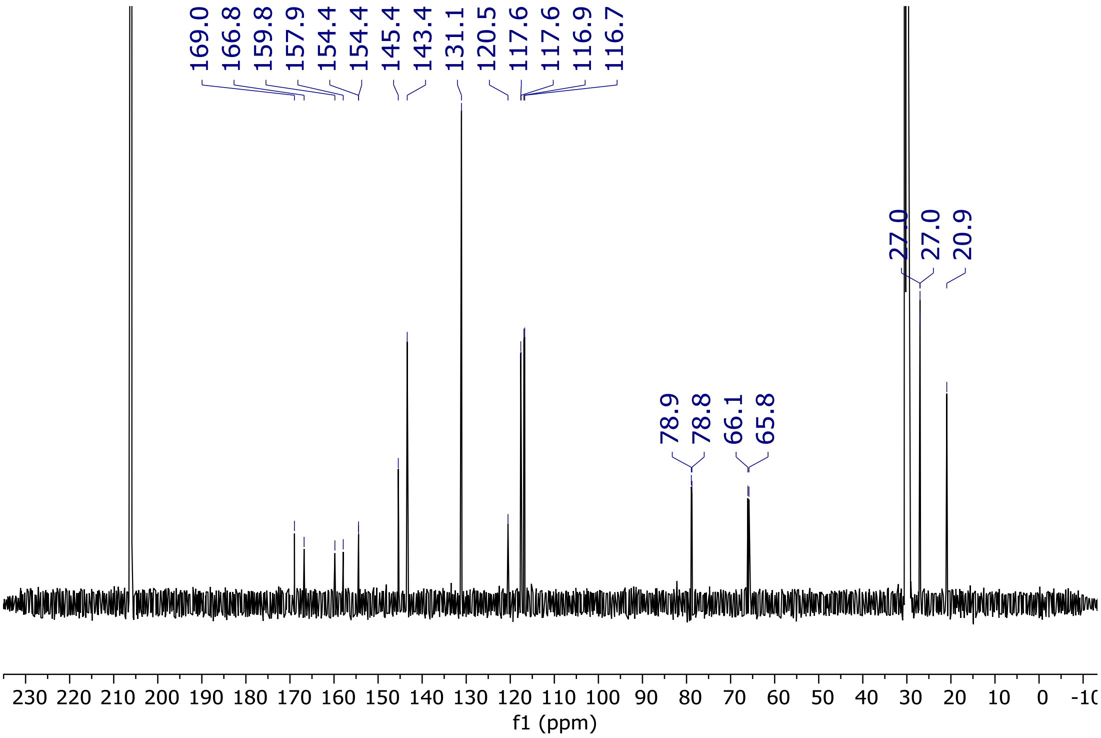

^{19}F NMR (376 MHz, $C(CD_3)_2O$):

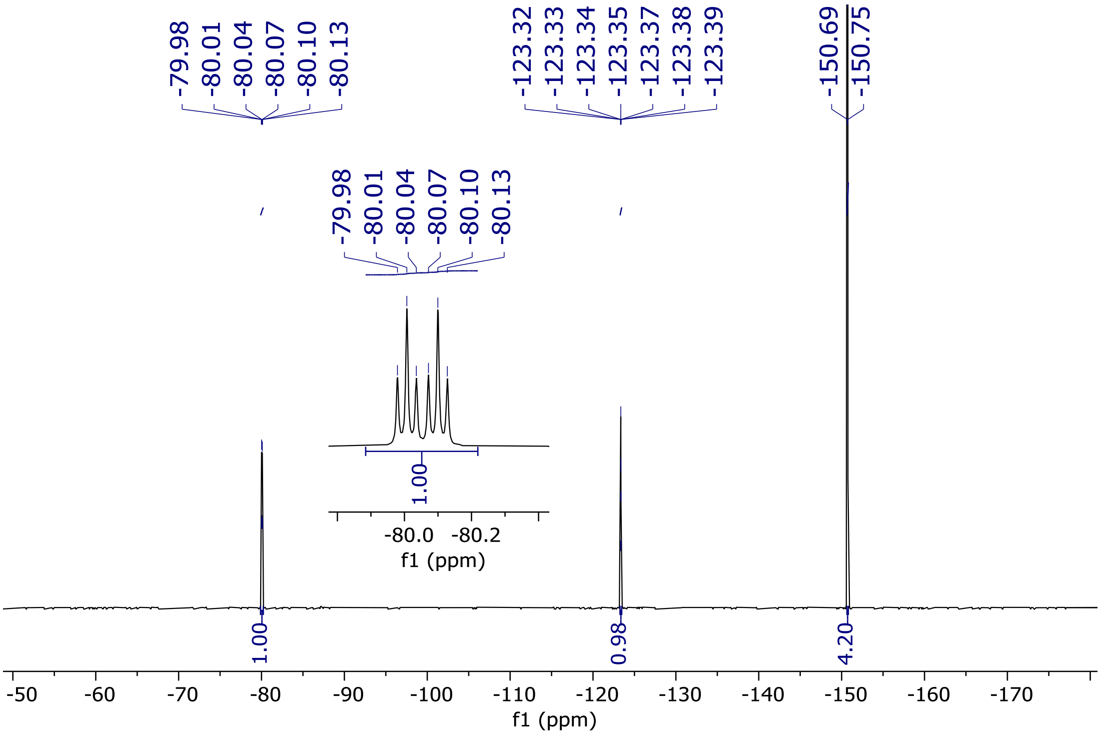

(Z)-(3-(4-chlorophenoxy)-2-fluoroprop-1-en-1-yl)(mesityl)iodonium BF_4 **(3p)**

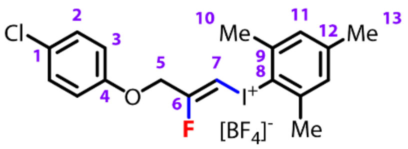

^{1}H NMR (500 MHz, $C(CD_3)_2O$):

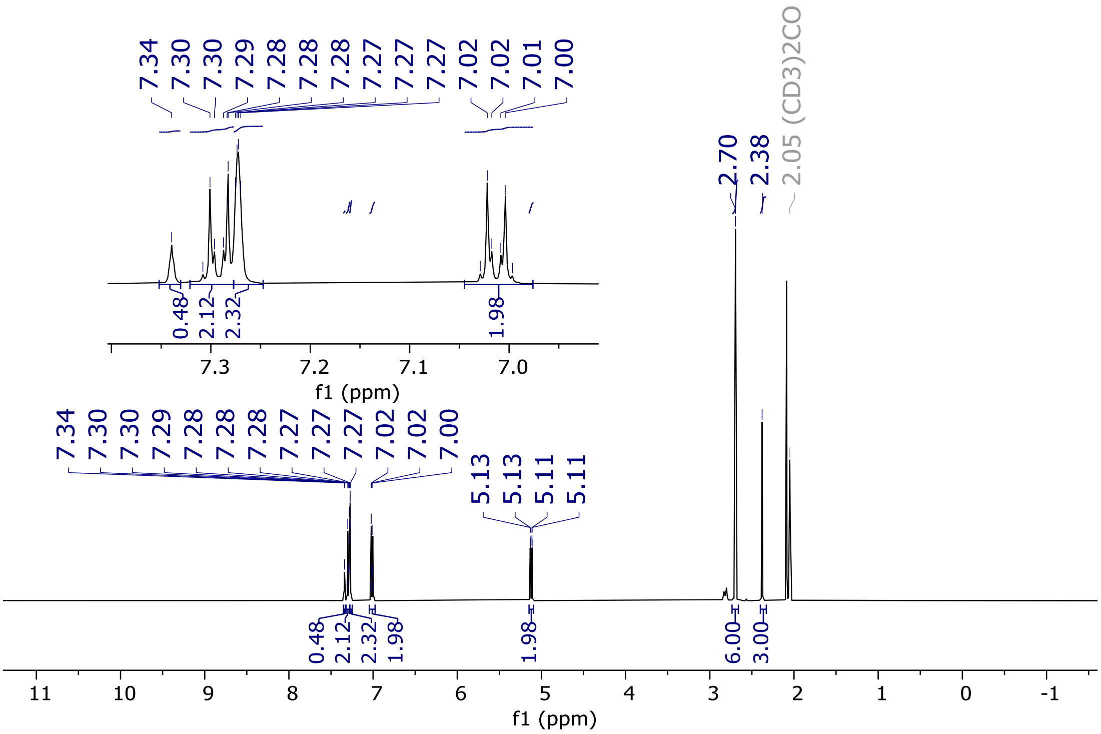

^{13}C NMR (126 MHz, $C(CD_3)_2O$):

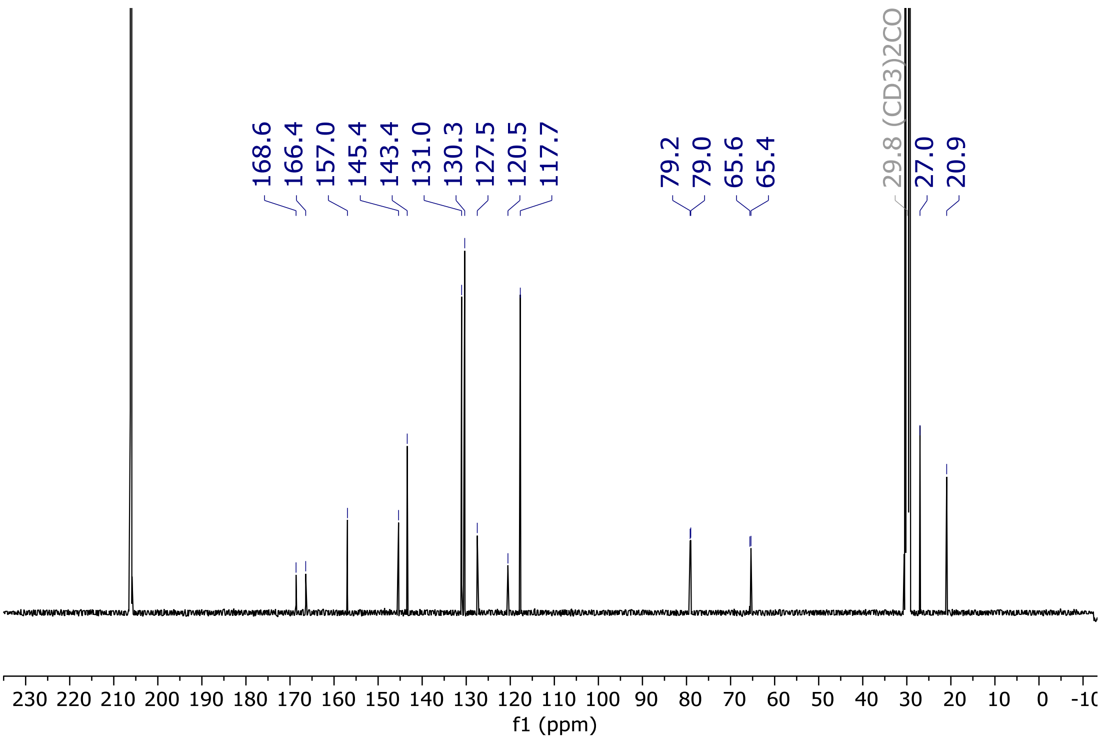

^{19}F NMR (283 MHz, $C(CD_3)_2O$):

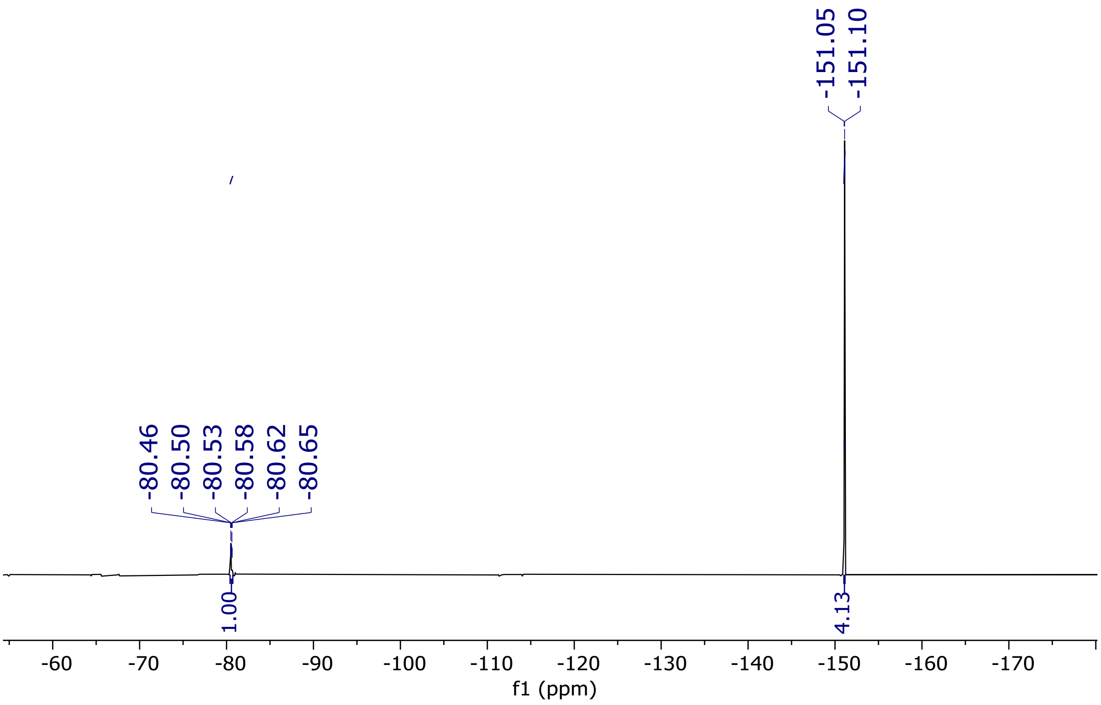

(Z)-(3-(4-bromophenoxy)-2-fluoroprop-1-en-1-yl)(mesityl)iodonium BF_4 **(3q)**

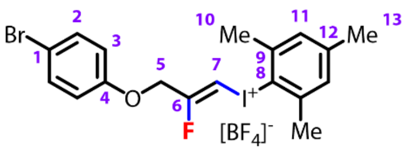

^{1}H NMR (500 MHz, $C(CD_3)_2O$):

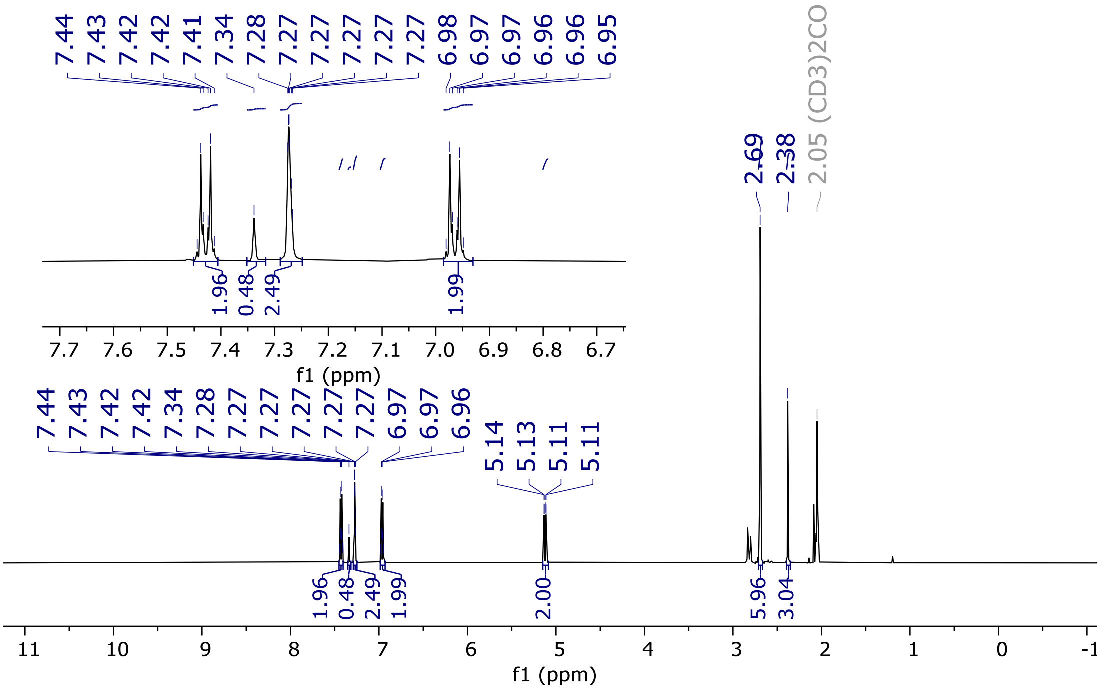

^{13}C NMR (126 MHz, $C(CD_3)_2O$):

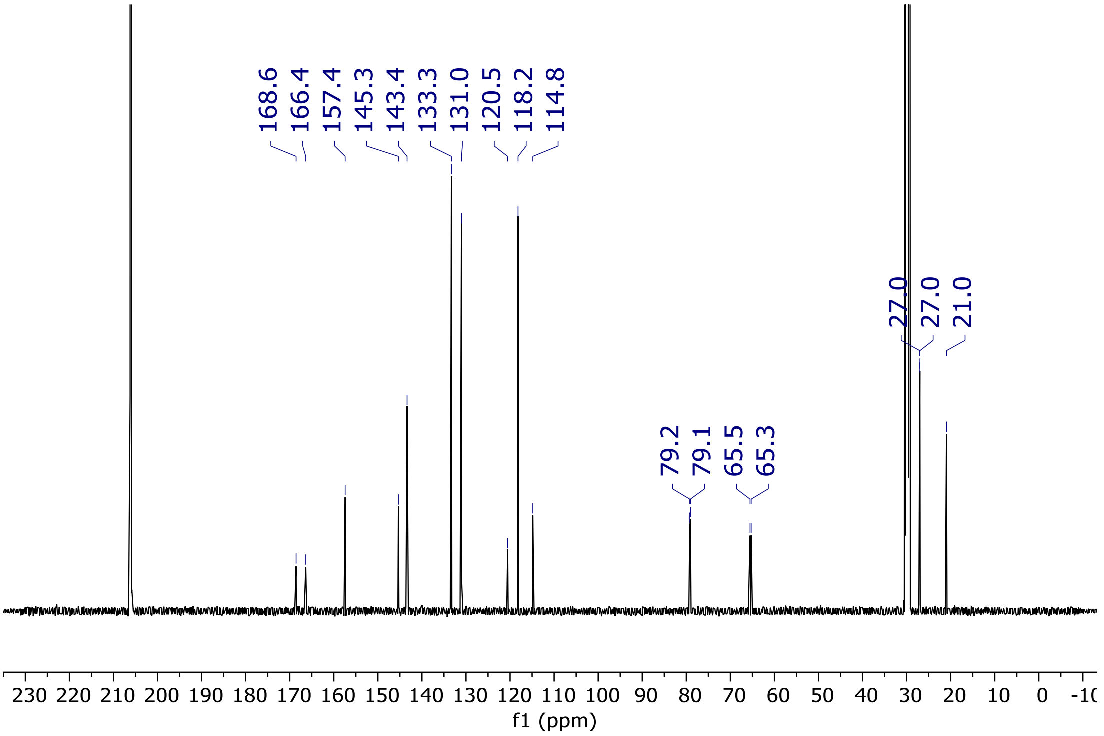

^{19}F NMR (376 MHz, $C(CD_3)_2O$):

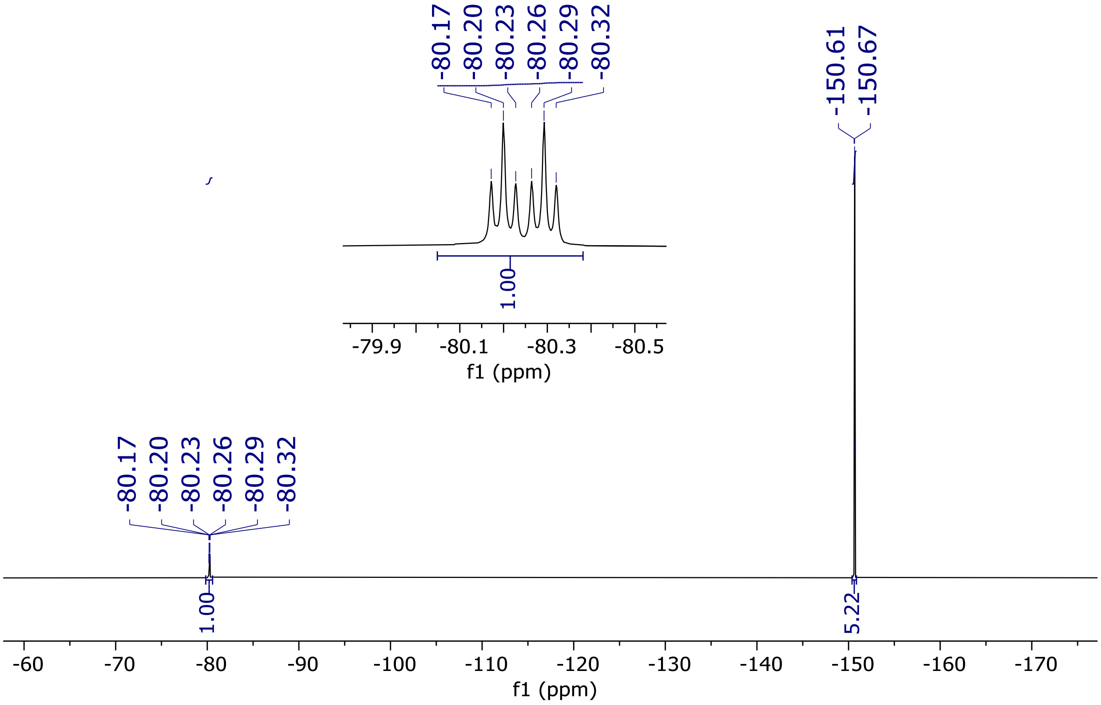

(Z)-(2-fluoro-3-phenoxyprop-1-en-1-yl)(mesityl)iodonium BF_4 **(3r)**

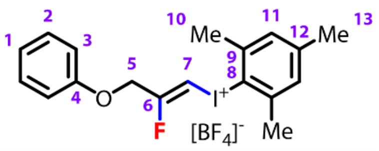

^{1}H NMR (600 MHz, $C(CD_3)_2O$):

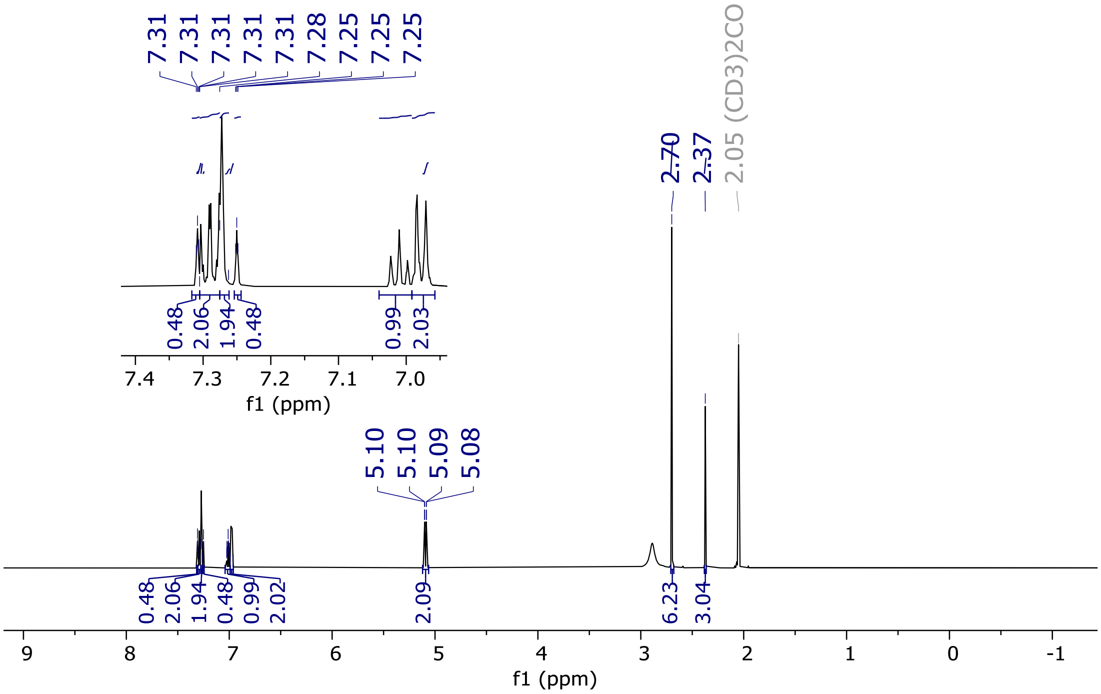

^{13}C NMR (151 MHz, $C(CD_3)_2O$):

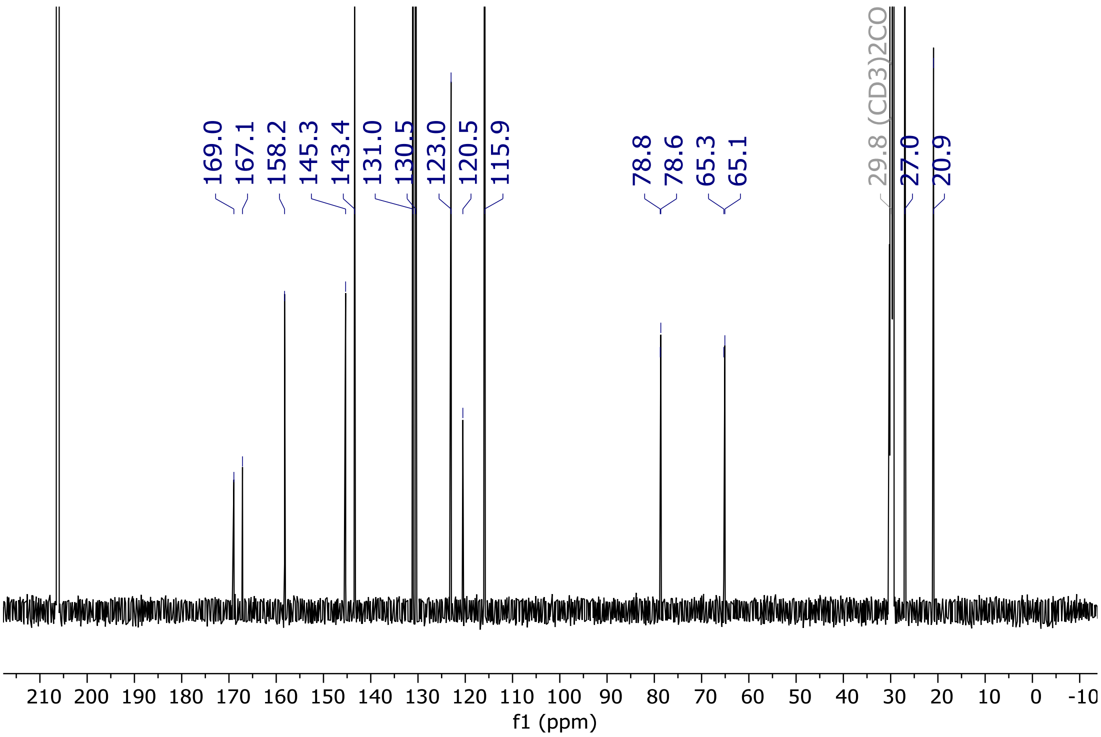

^{19}F NMR (376 MHz, $C(CD_3)_2O$):

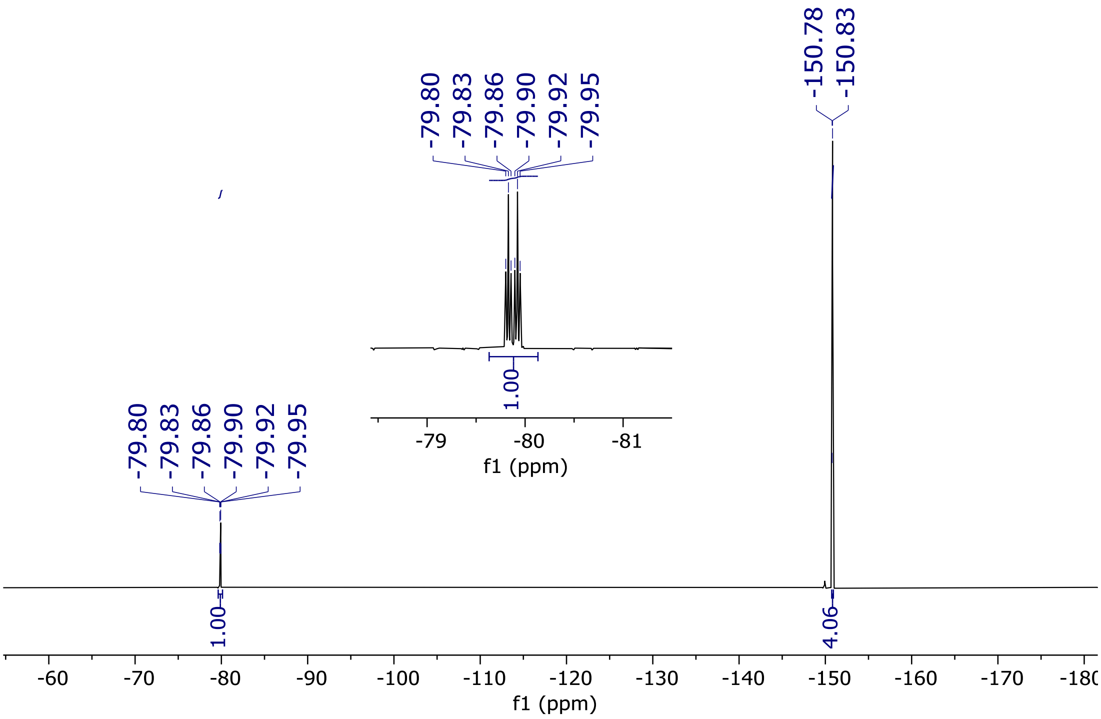

(Z)-1-((2-fluoro-3-iodoallyl)oxy)-2-methoxybenzene ***(3s)***

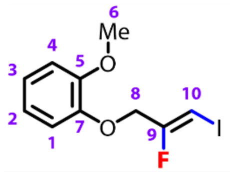

^{1}H NMR (500 MHz, CDCl$_3$):

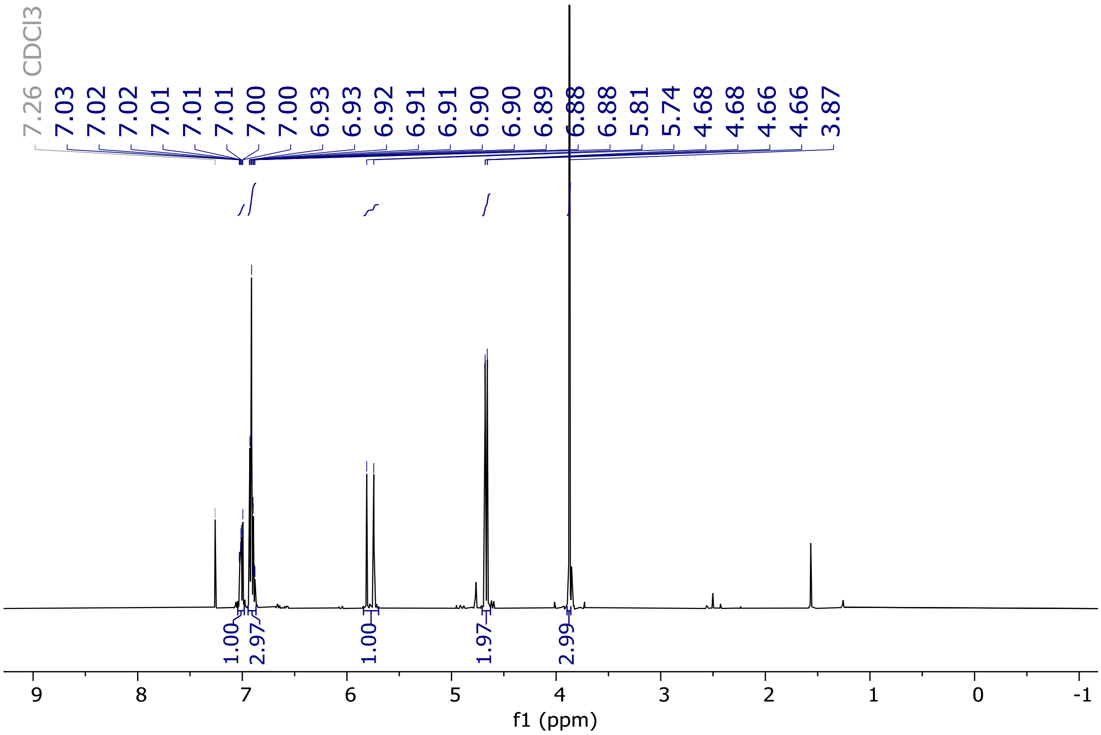

^{13}C NMR (126 MHz, CDCl$_3$):)

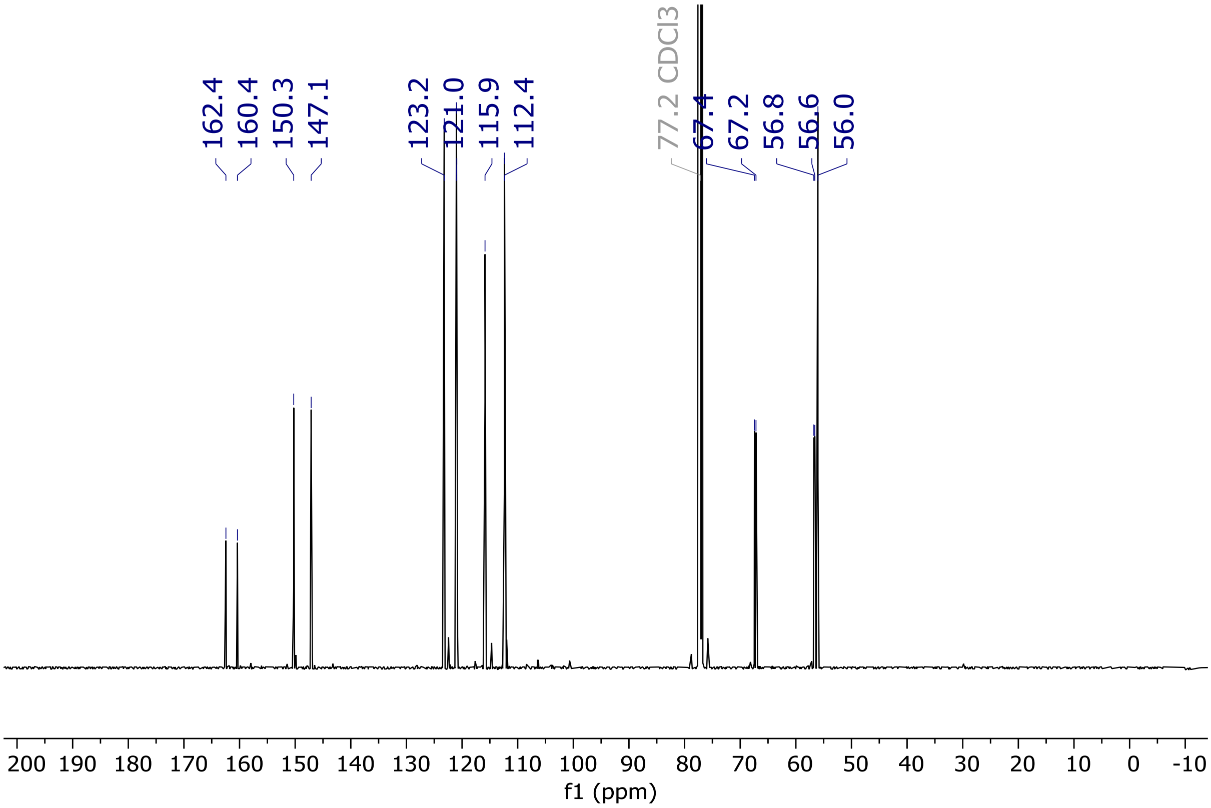

^{19}F NMR (376 MHz, $C(CD_3)_2O$):

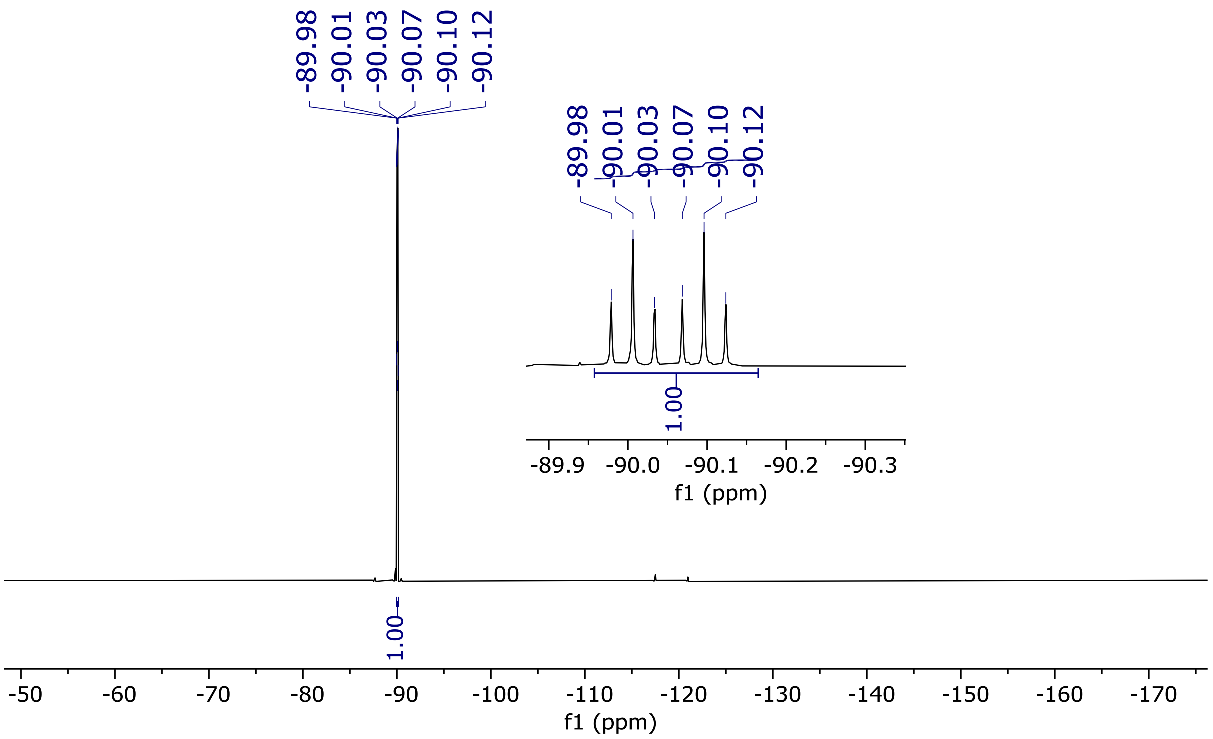

(Z)-(3-(3,5-dibromophenoxy)-2-fluoroprop-1-en-1-yl)(mesityl)iodonium BF_4 ***(3t)***

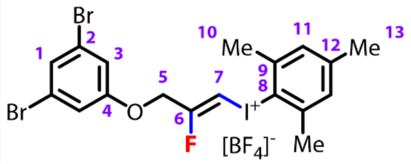

^{1}H NMR (600 MHz, $C(CD_3)_2O$):

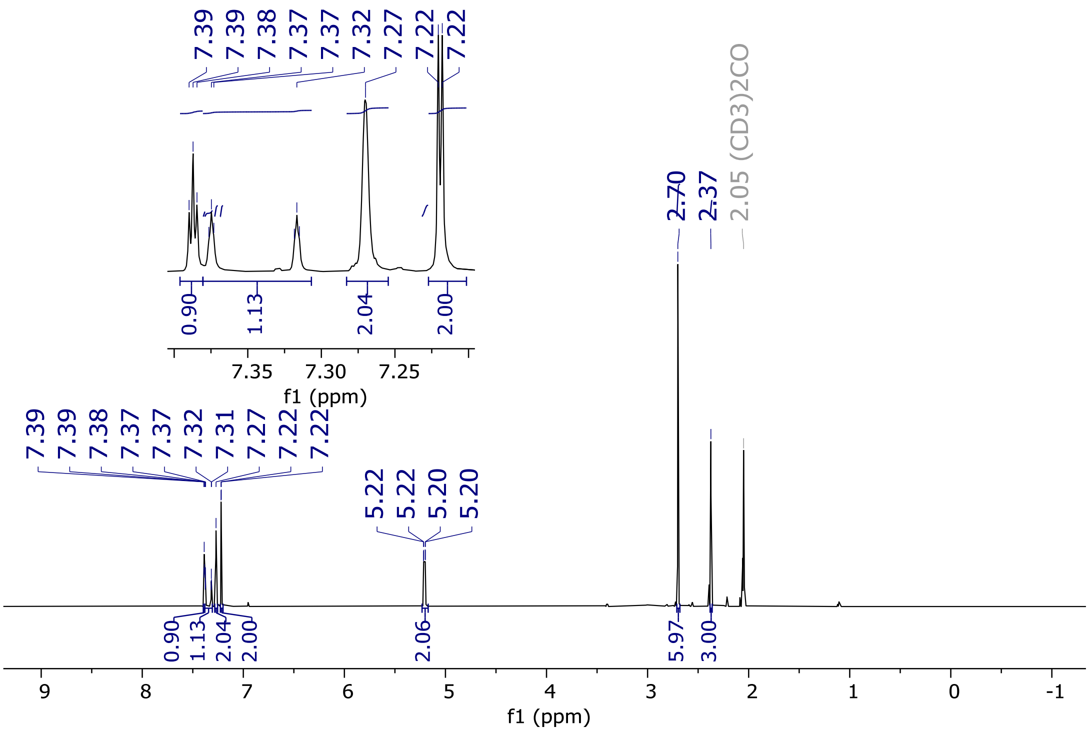

^{13}C NMR (151 MHz, $C(CD_3)_2O$):

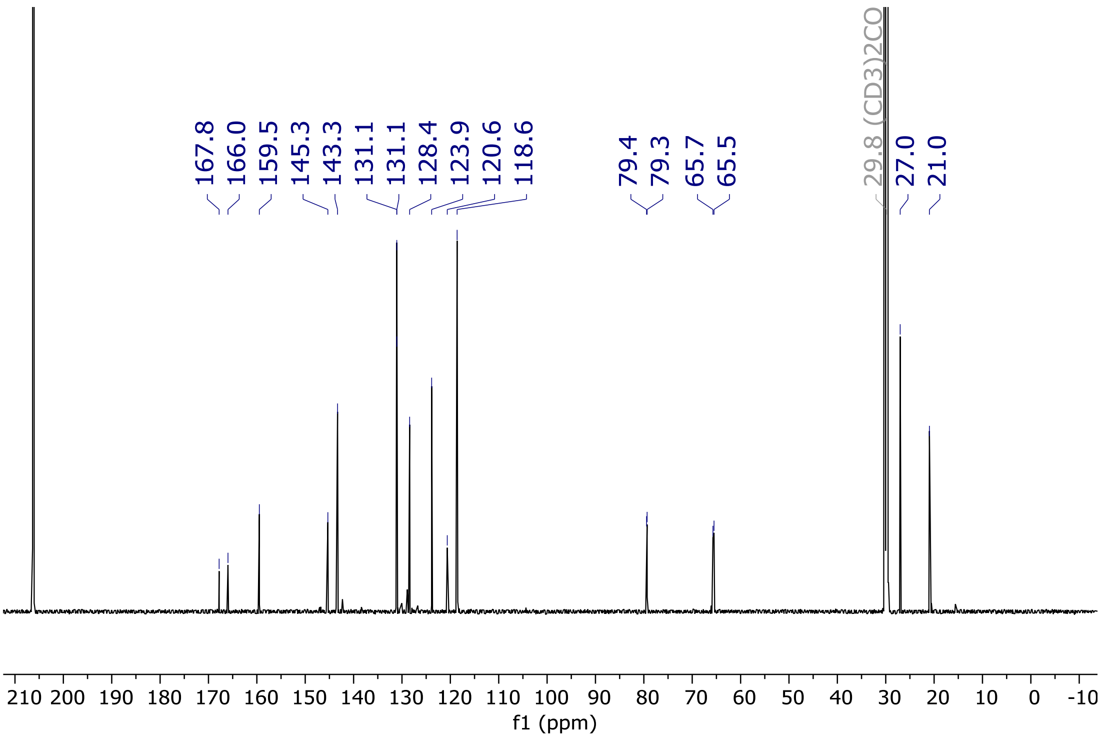

^{19}F NMR (376 MHz, $C(CD_3)_2O$):

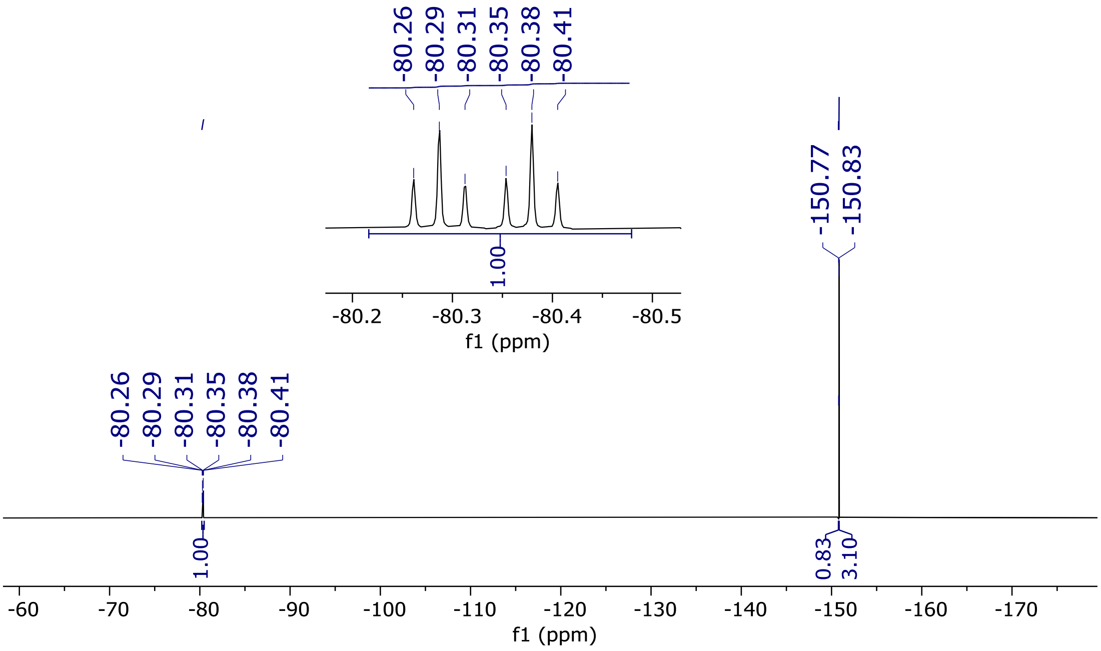

(Z)-(2-fluoro-3-(2,4,6-trichlorophenoxy)prop-1-en-1-yl)(mesityl)iodonium BF_4 **(3u)**

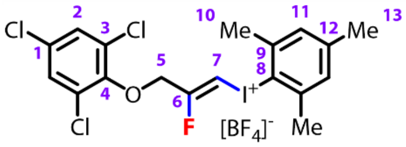

^{1}H NMR (500 MHz, $C(CD_3)_2O$):

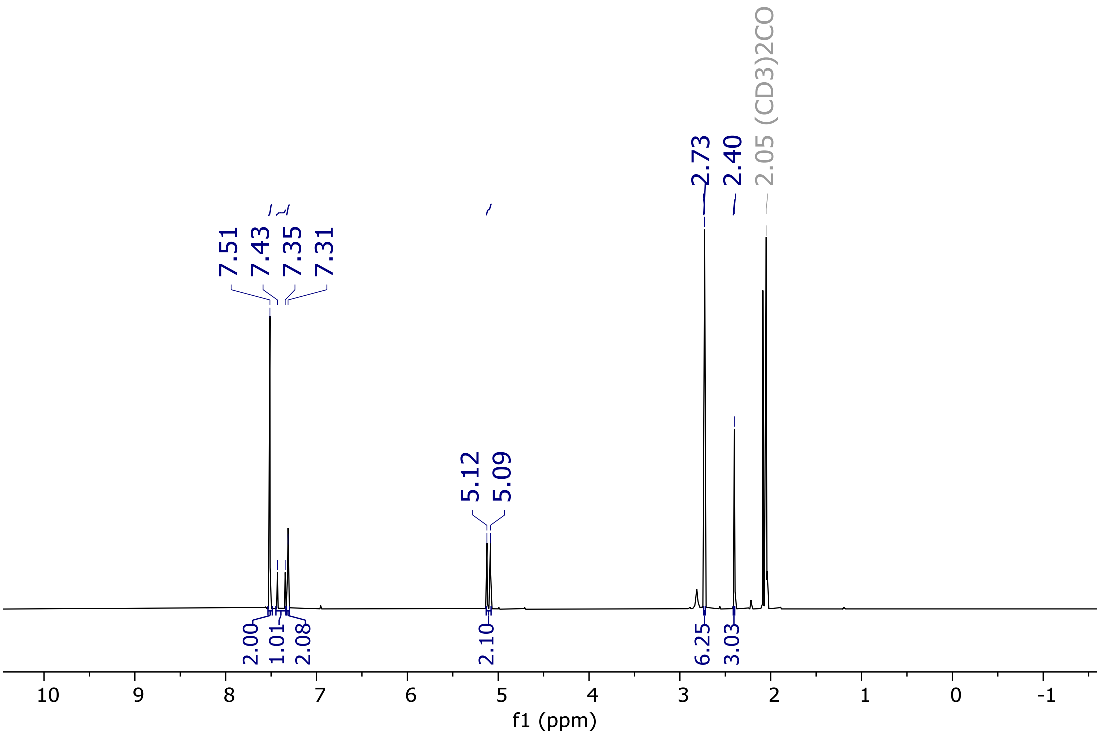

^{13}C NMR (126 MHz, $C(CD_3)_2O$):

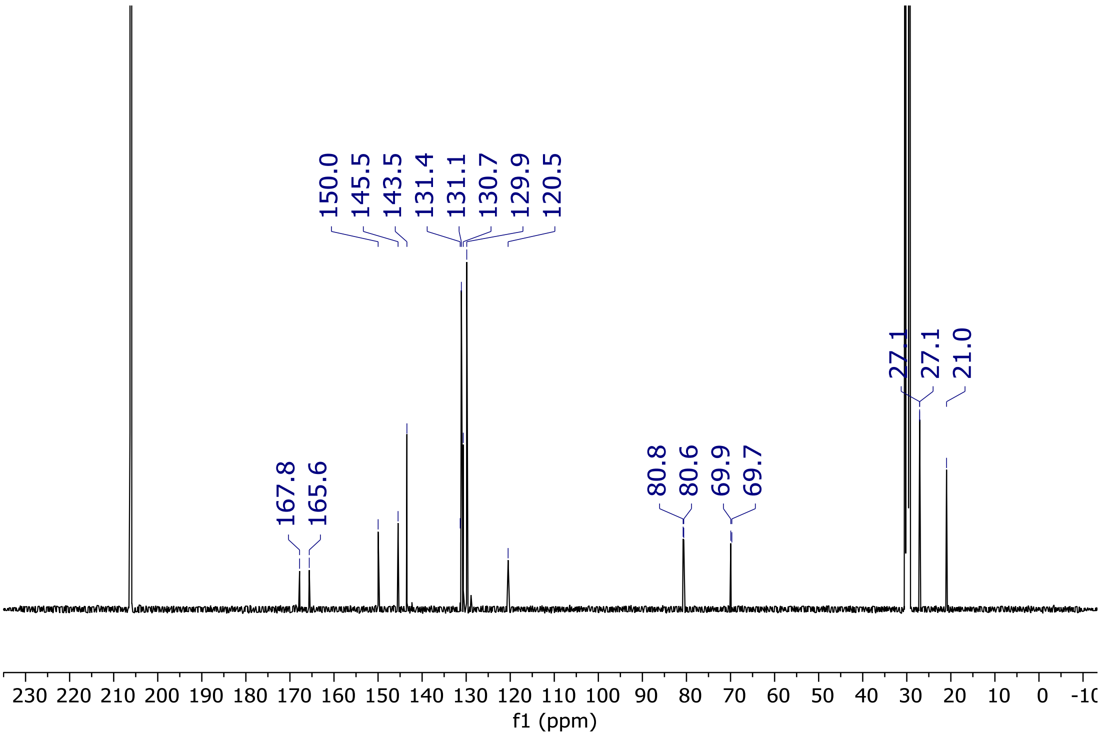

^{19}F NMR (376 MHz, $C(CD_3)_2O$):

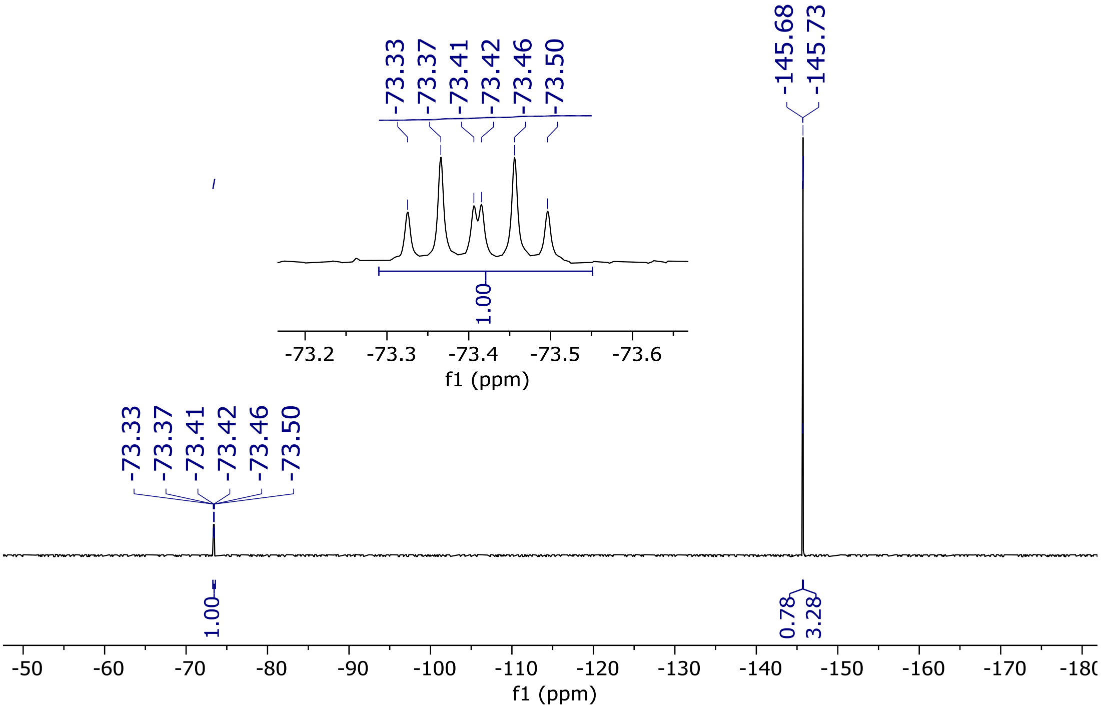

(Z)-(3-(2,6-dimethylphenoxy)-2-fluoroprop-1-en-1-yl)(mesityl)iodonium BF_4 ***(3v)***

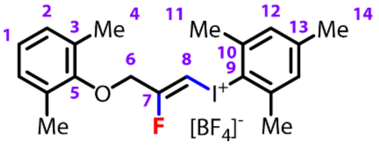

^{1}H NMR (500 MHz, $C(CD_3)_2O$):

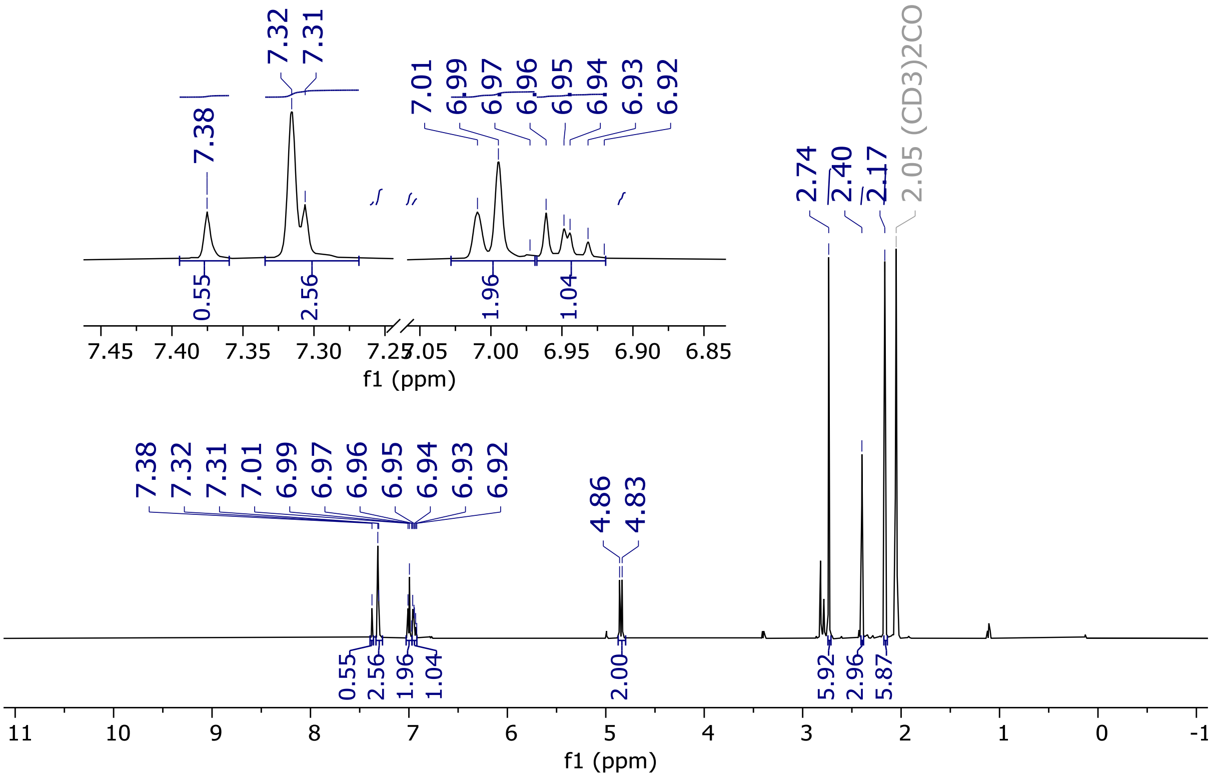

^{13}C NMR (126 MHz, $C(CD_3)_2O$):

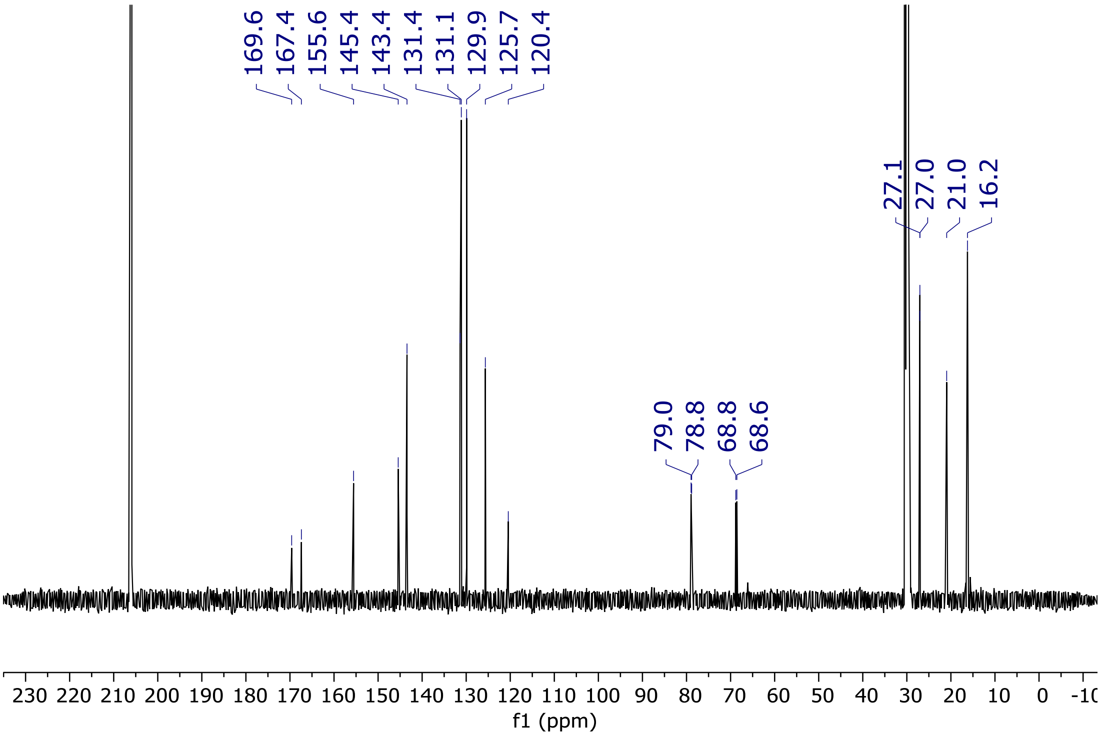

^{19}F NMR (376 MHz, $C(CD_3)_2O$):

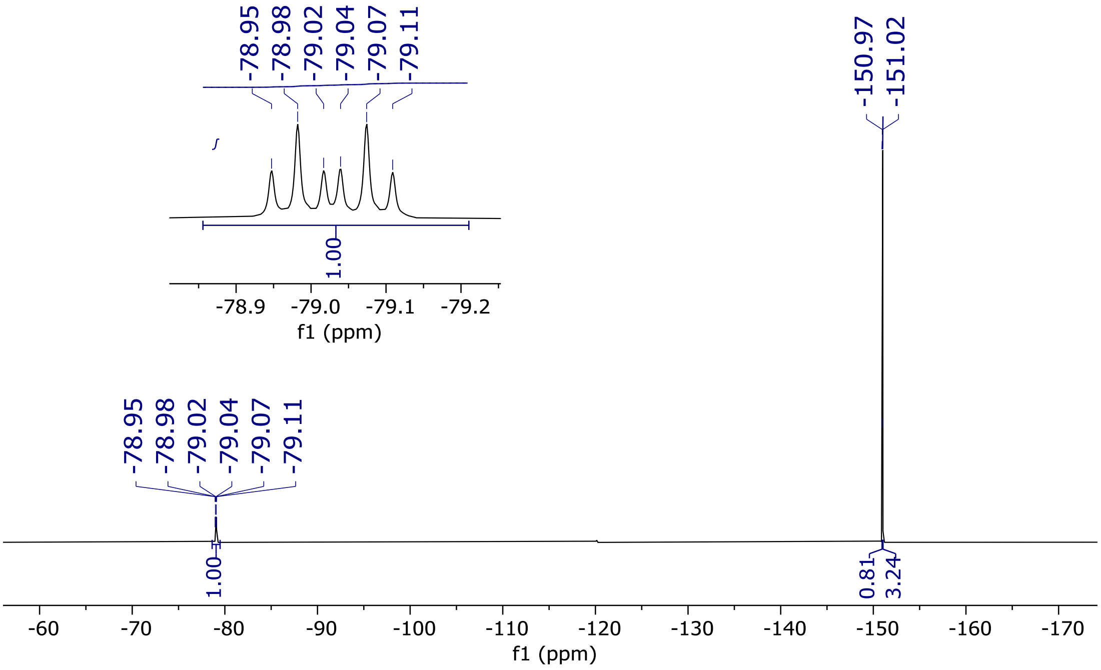

(Z)-(2-fluoro-3-methoxyprop-1-en-1-yl)(mesityl)iodonium BF_4 ***(3w)***

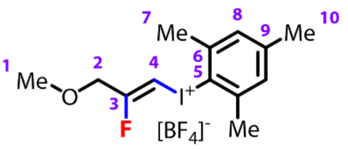

^{1}H NMR (400 MHz, $C(CD_3)_2O$):

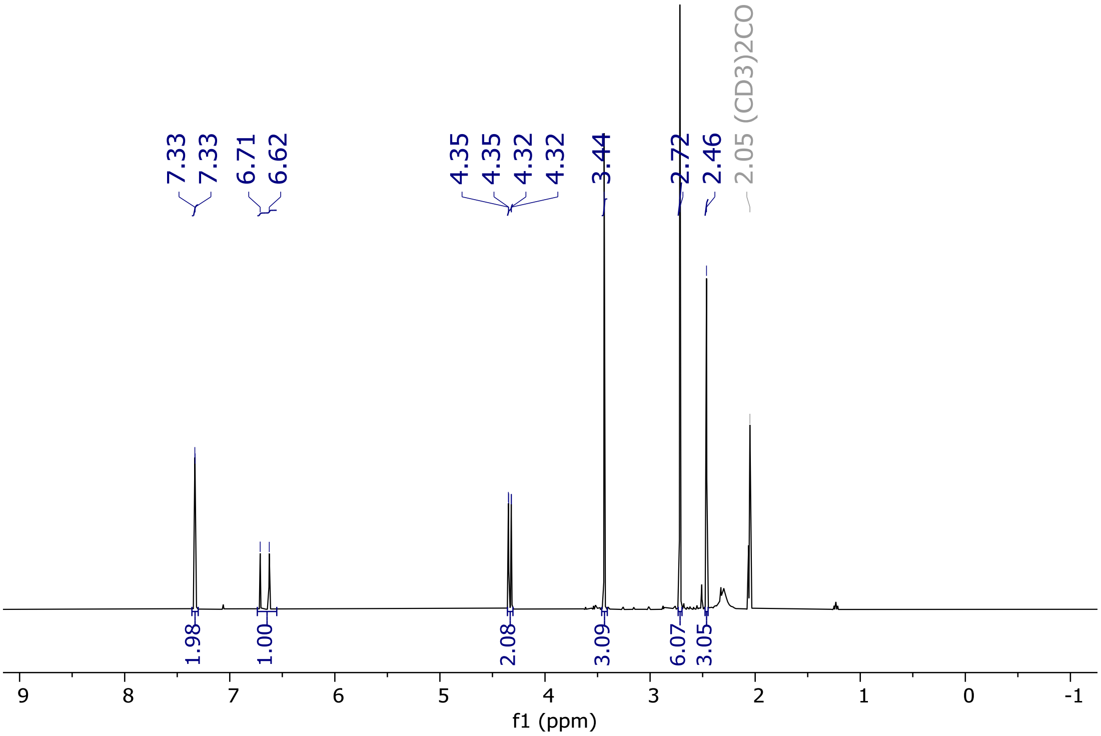

^{13}C NMR (126 MHz, CD_3CN):

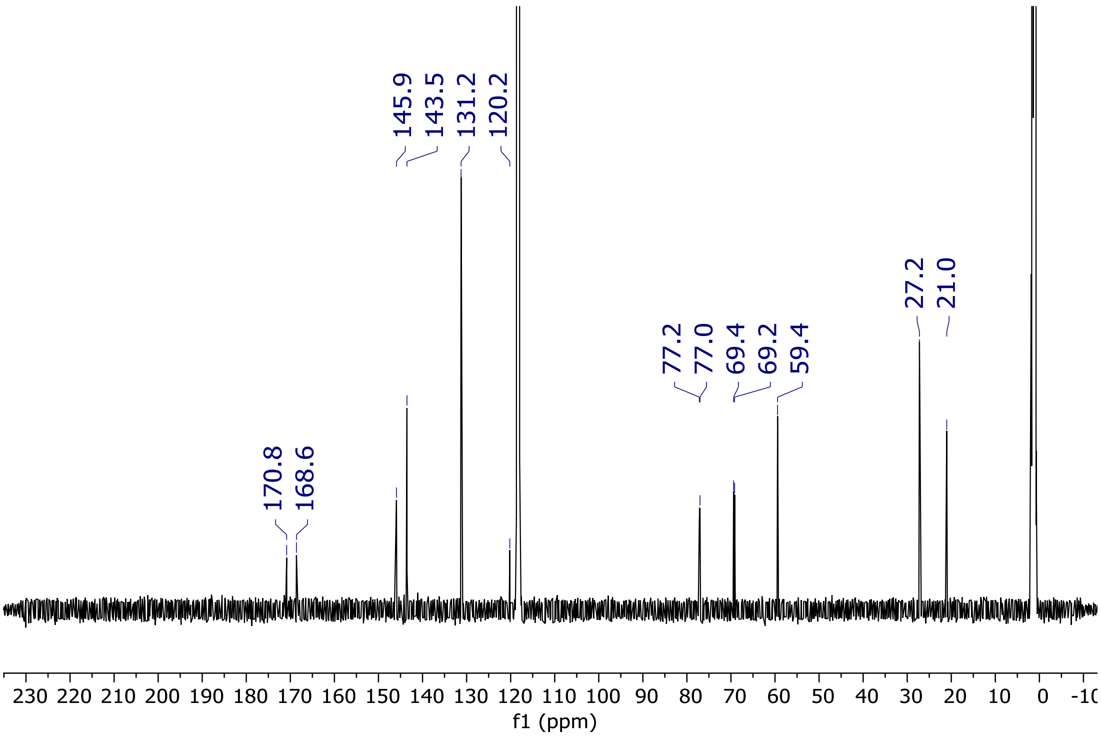

^{19}F NMR (376 MHz, CD_3CN):

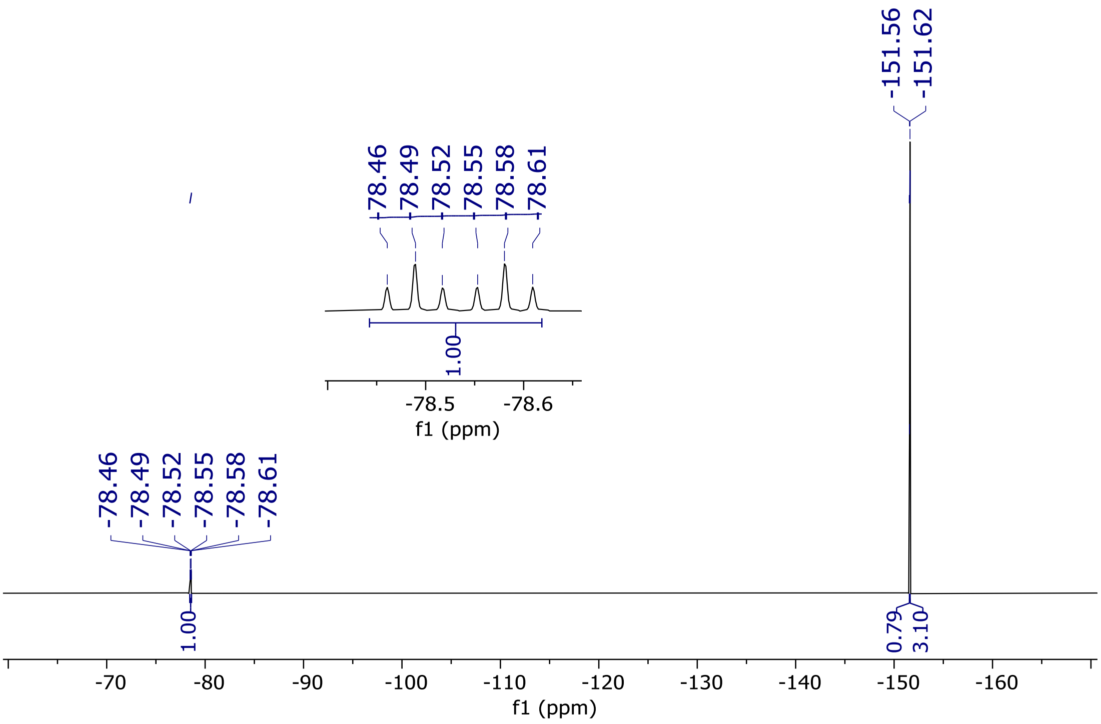

(Z)-(2-fluoro-3-hydroxy-3-methylbut-1-en-1-yl)(mesityl)iodonium BF_4 ***(3x)***

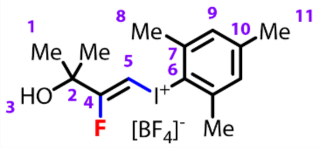

^{1}H NMR (500 MHz, $C(CD_3)_2O$):

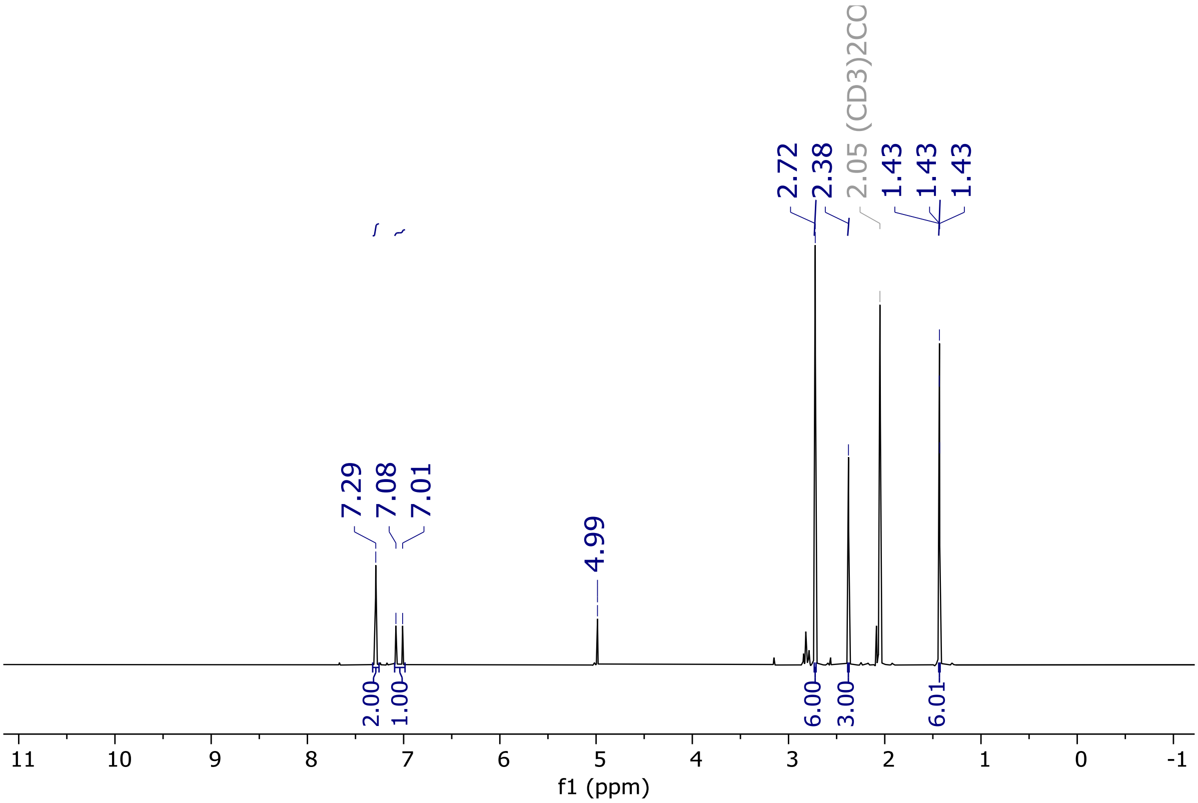

^{13}C NMR (126 MHz, $C(CD_3)_2O$):

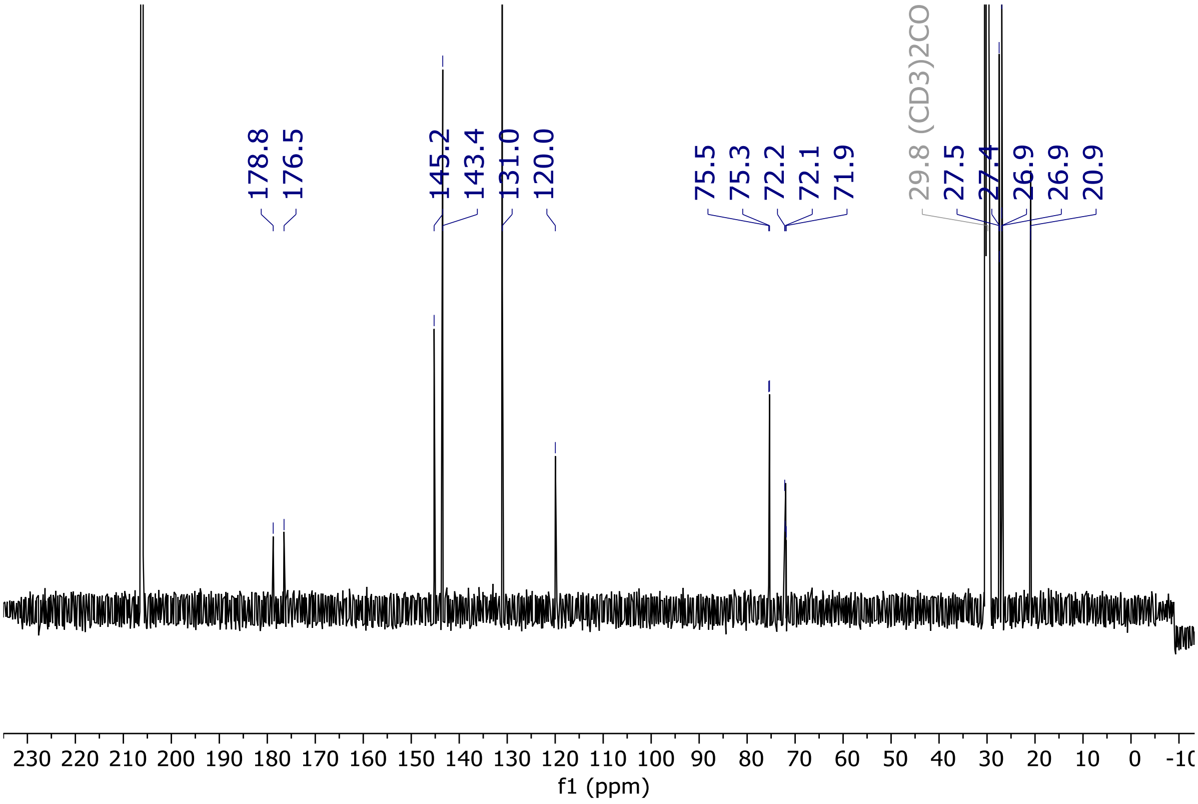

^{19}F NMR (377 MHz, $C(CD_3)_2O$):

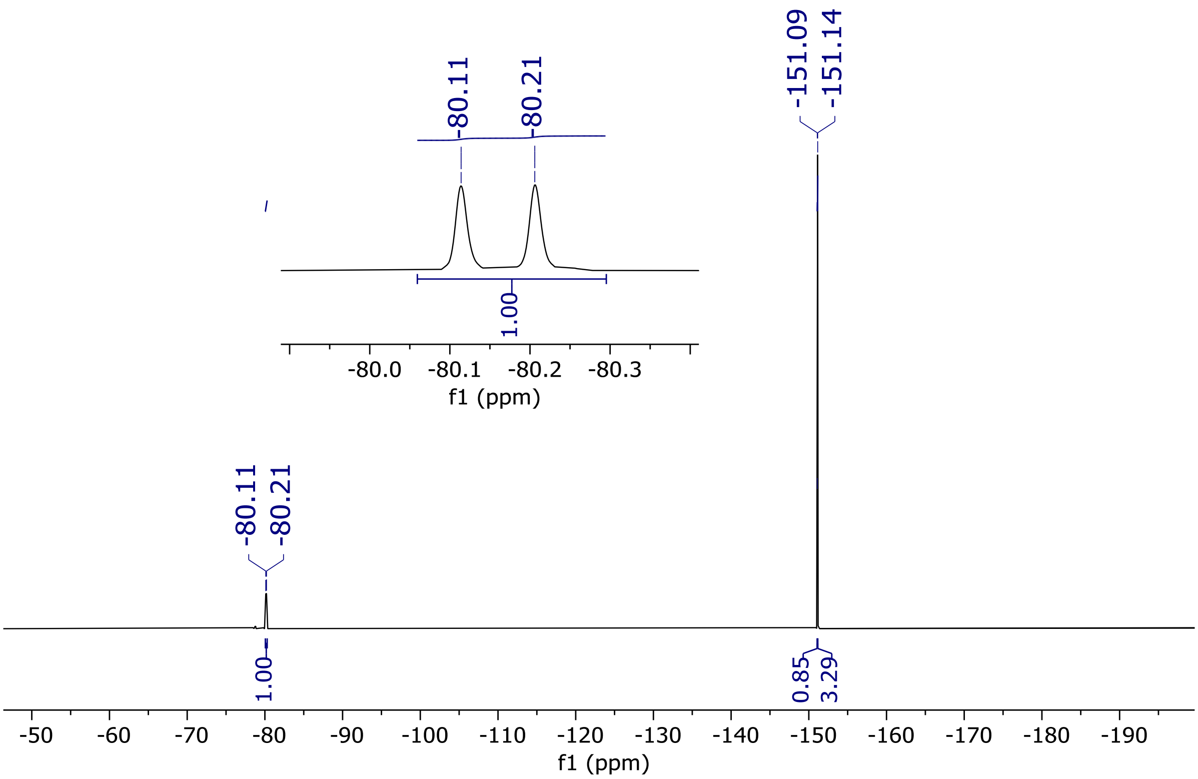

(Z)-(2-fluoro-3-((N-(4-fluorobenzyl)-4-nitrophenyl)sulfonamido)prop-1-en-1-yl)(mesityl)iodonium BF_4 ***(3y)***

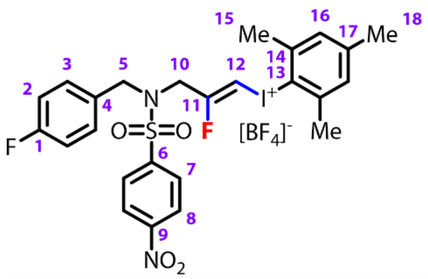

^{1}H NMR (500 MHz, CD_3CN):

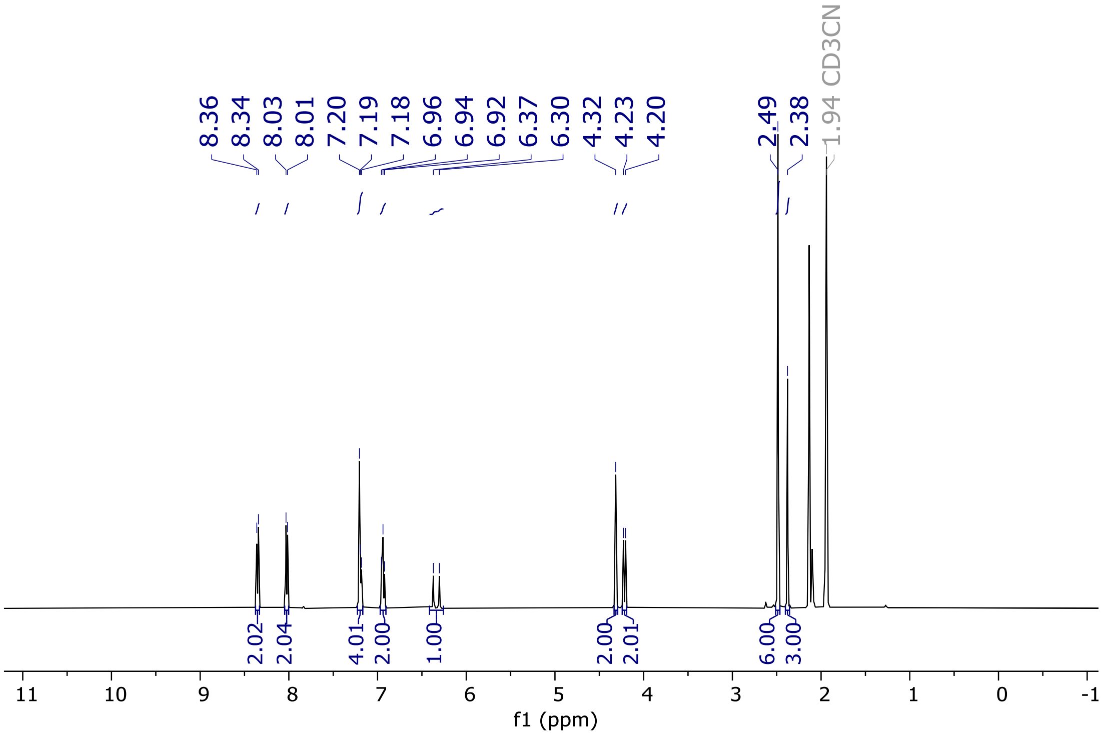

^{13}C NMR (126 MHz, CD_3CN):

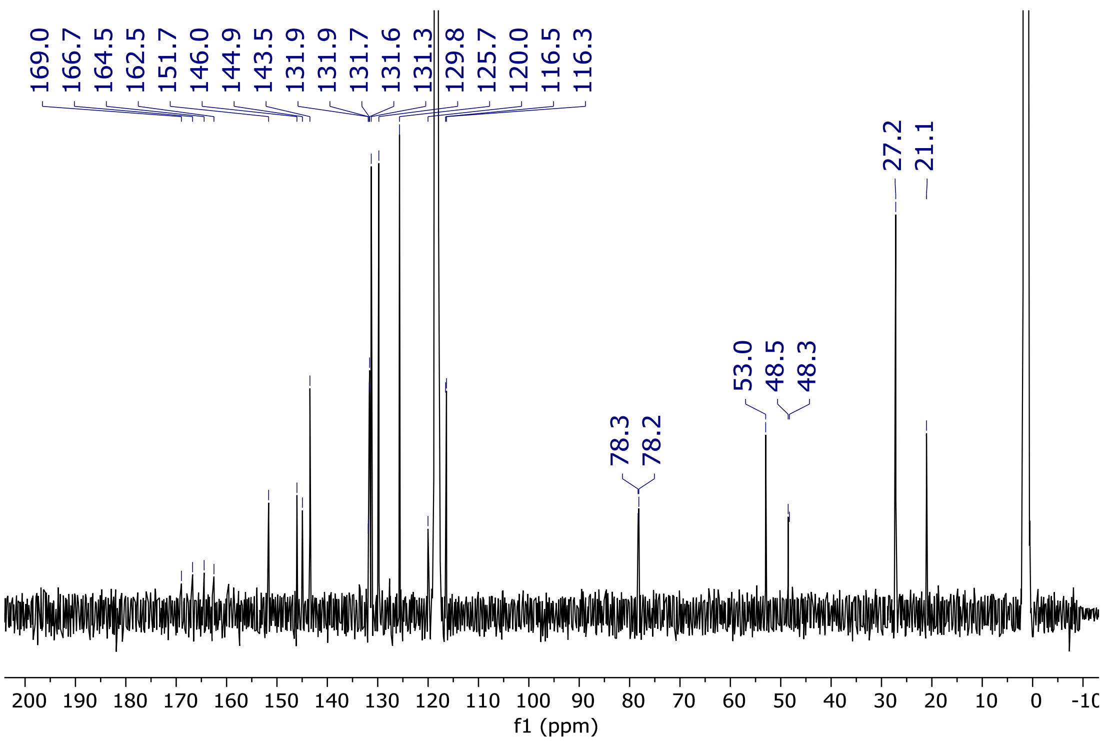

^{19}F NMR (376 MHz, CD_3CN):

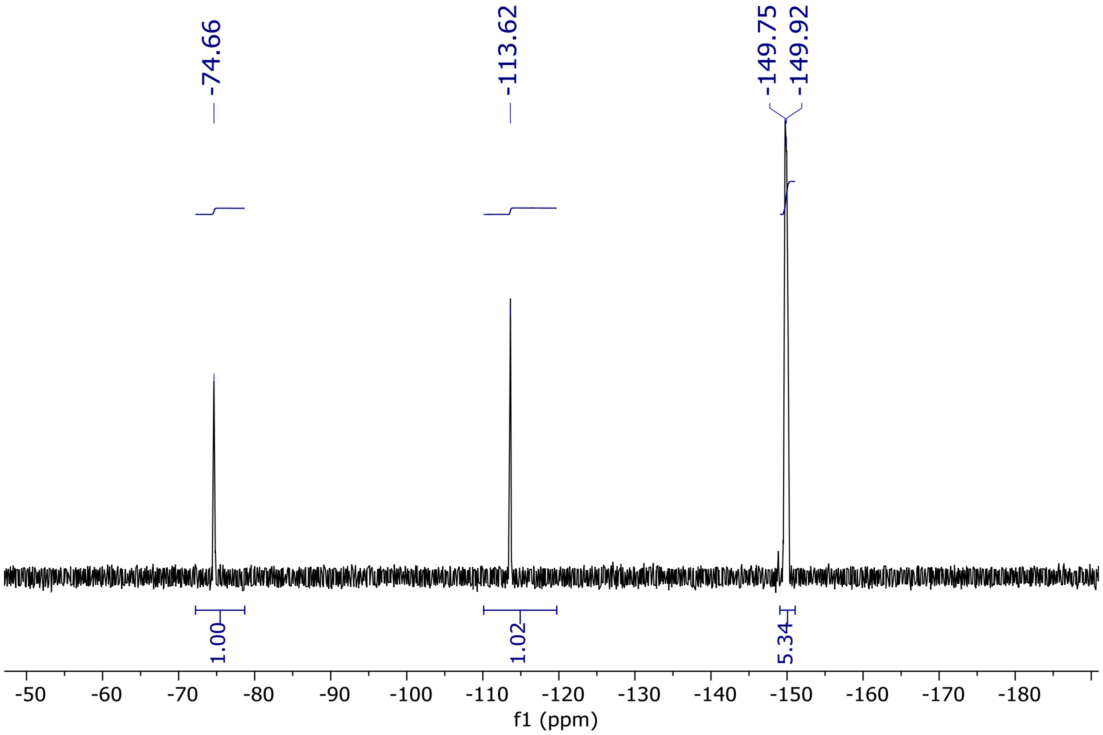

(Z)-(3-(1,3-dioxoisoindolin-2-yl)-2-fluoroprop-1-en-1-yl)(mesityl)iodonium BF_4 ***(3z)***

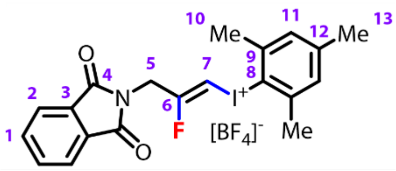

^{1}H NMR (500 MHz, CD_3CN):

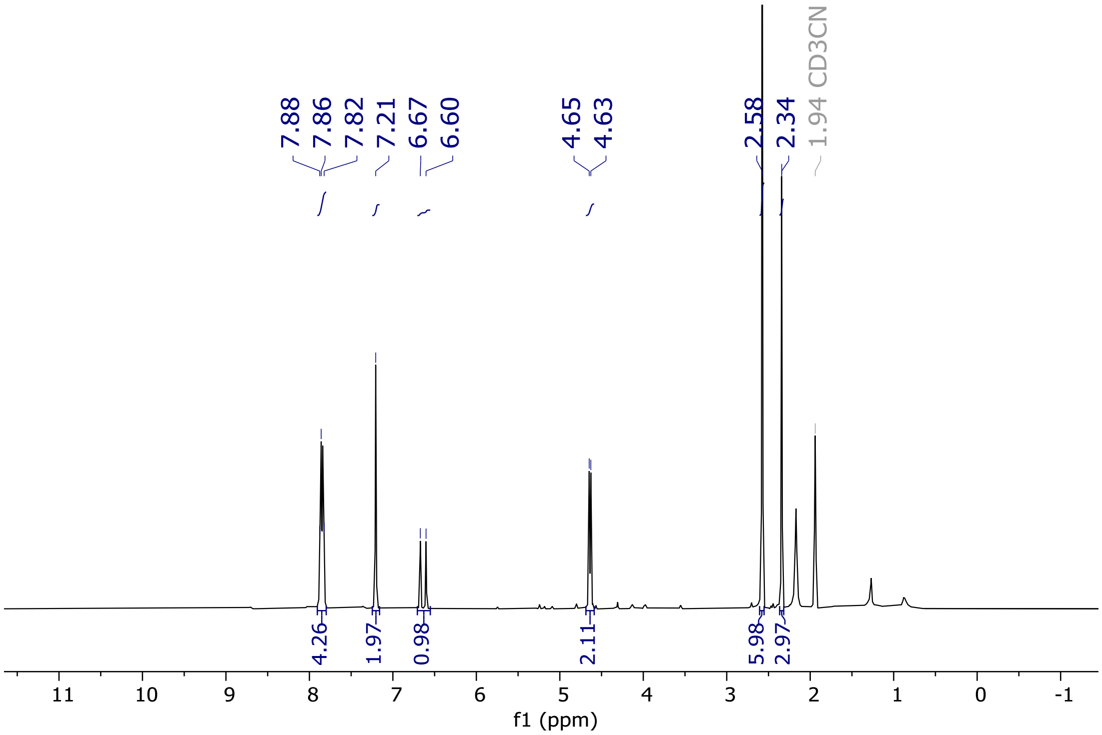

^{13}C NMR (126 MHz, CD_3CN):

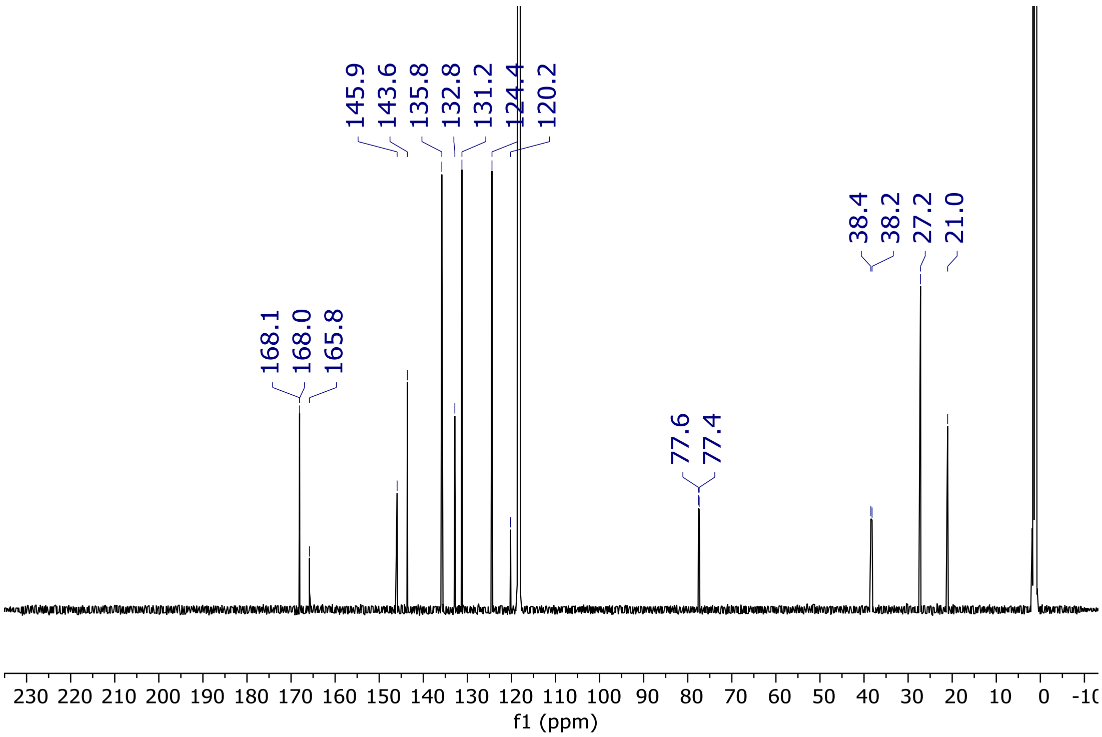

^{19}F NMR (376 MHz, CD_3CN):

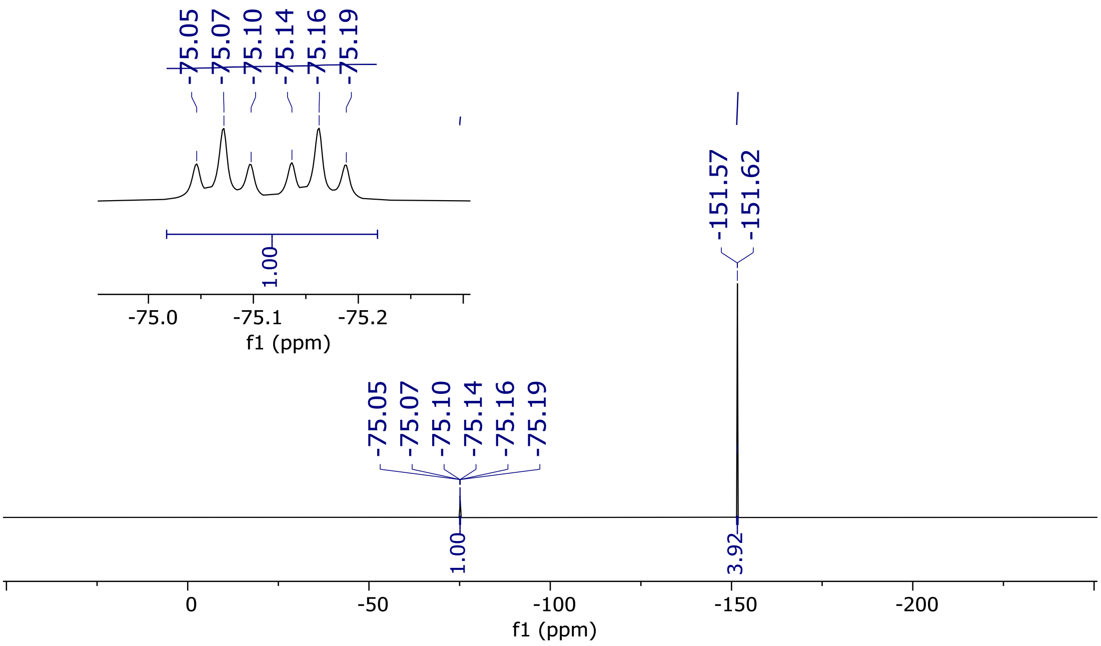

(Z)-(3-((N-cyclopropyl-4-methylphenyl)sulfonamido)-2-fluoroprop-1-en-1-yl)(mesityl)iodonium BF_4 ***(3aa)***

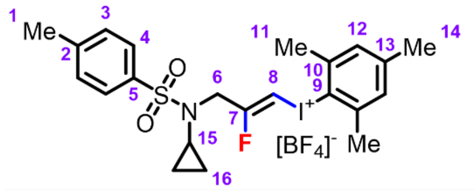

^{1}H NMR (500 MHz, $C(CD_3)_2O$):

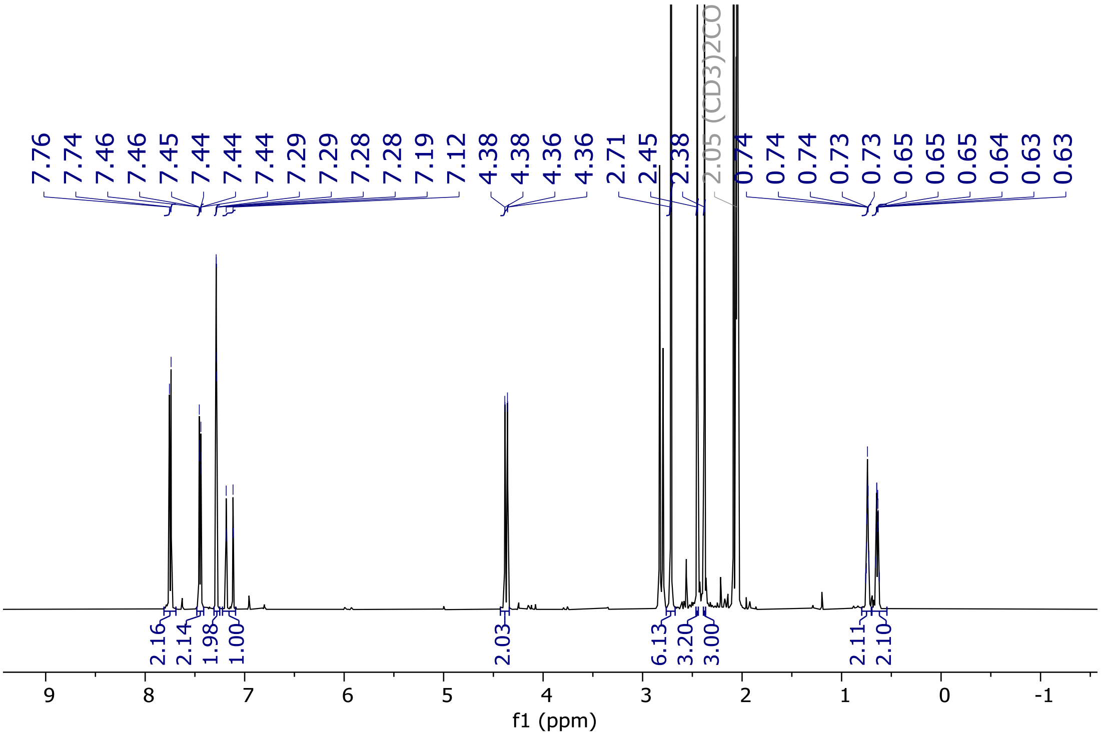

^{13}C NMR (126 MHz, $C(CD_3)_2O$):

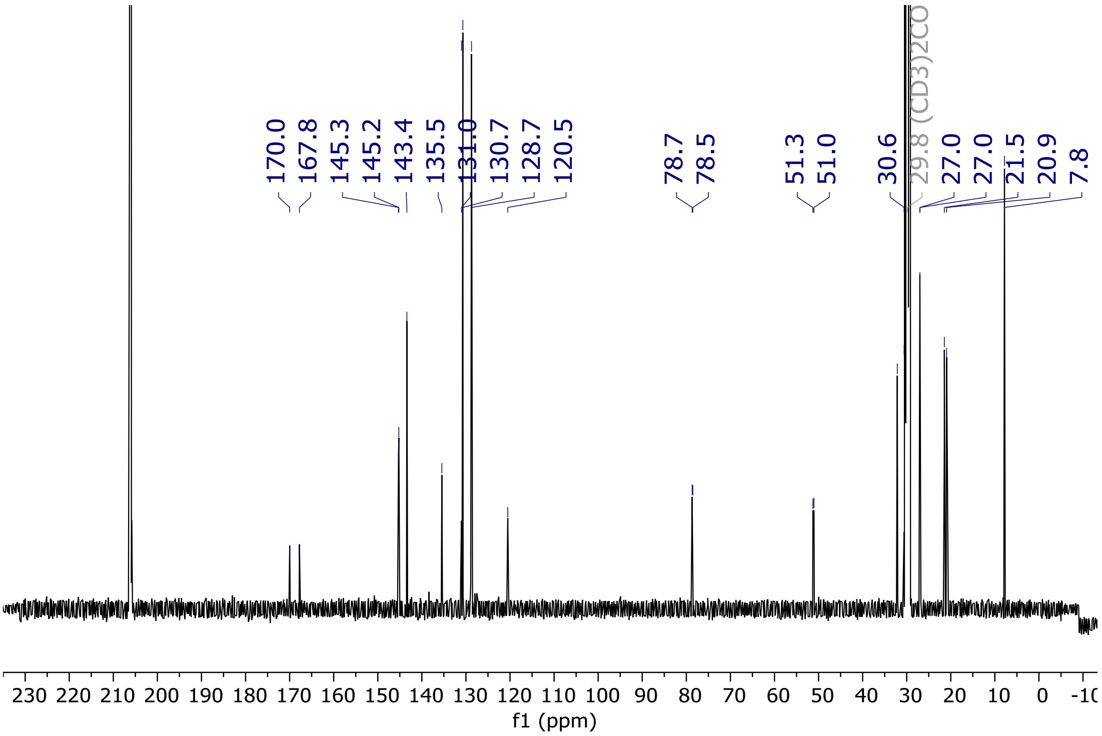

^{19}F NMR (376 MHz, $C(CD_3)_2O$):

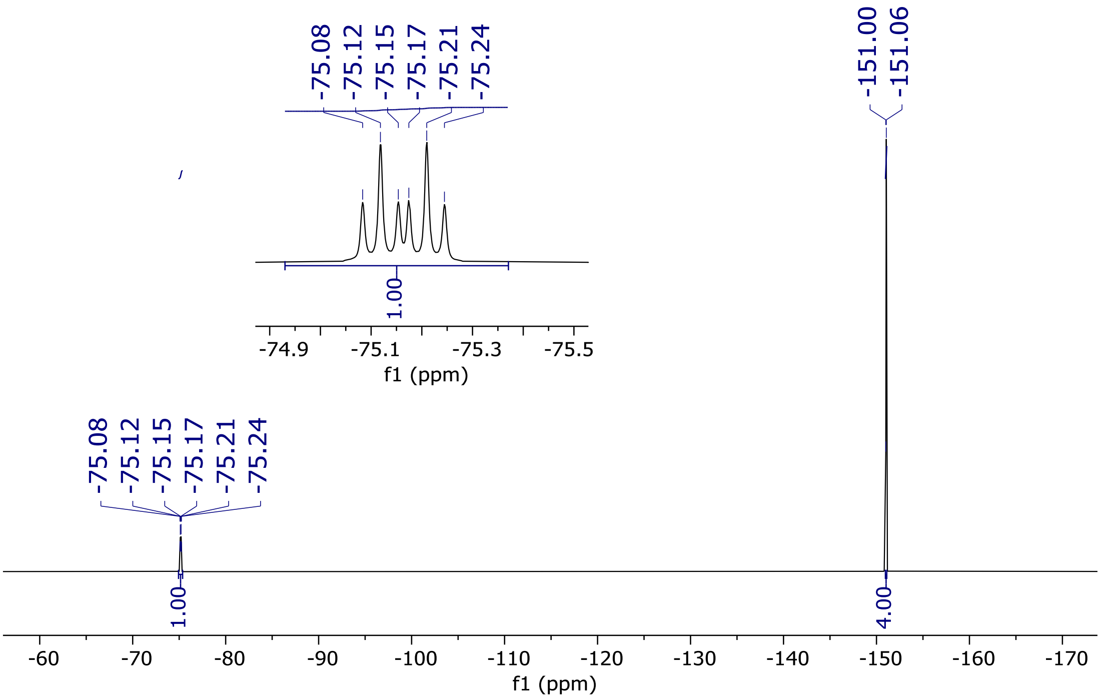

(Z)-(2-fluoro-3-((4-methylphenyl)sulfonamido)prop-1-en-1-yl)(mesityl)iodonium BF_4 ***(3ab)***

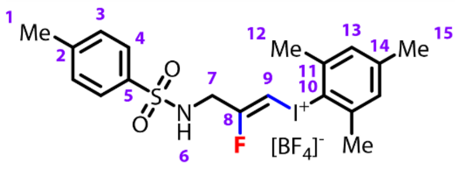

¹H NMR (400 MHz, $C(CD_3)_2O$):

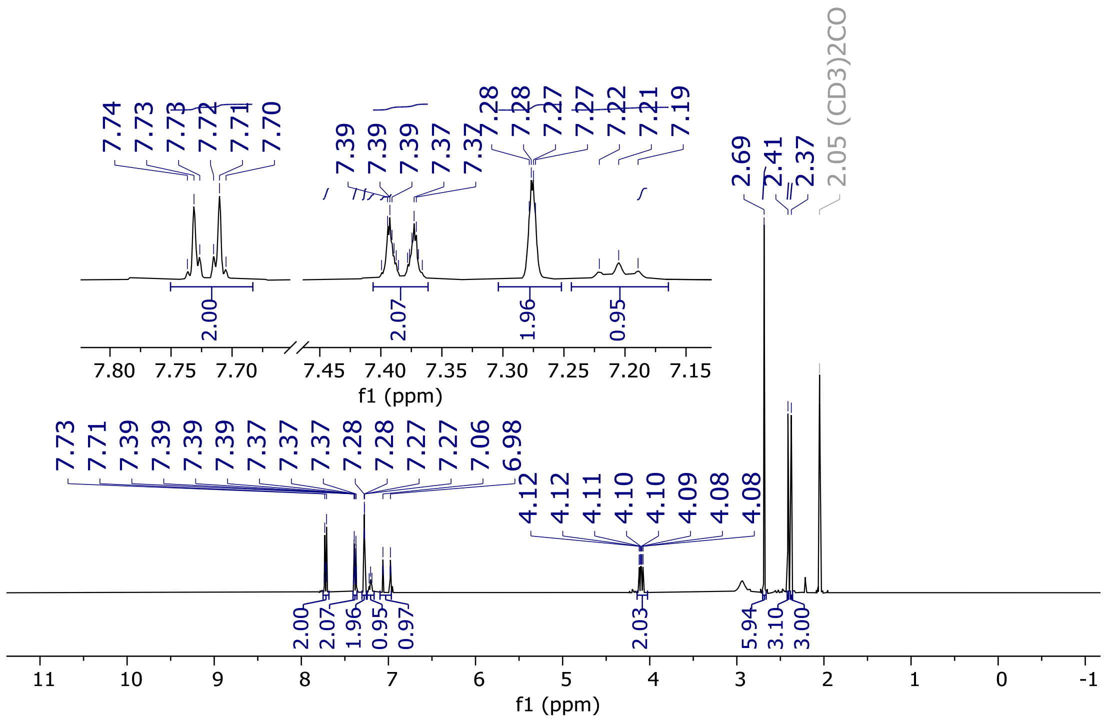

¹³C NMR (126 MHz, $C(CD_3)_2O$):

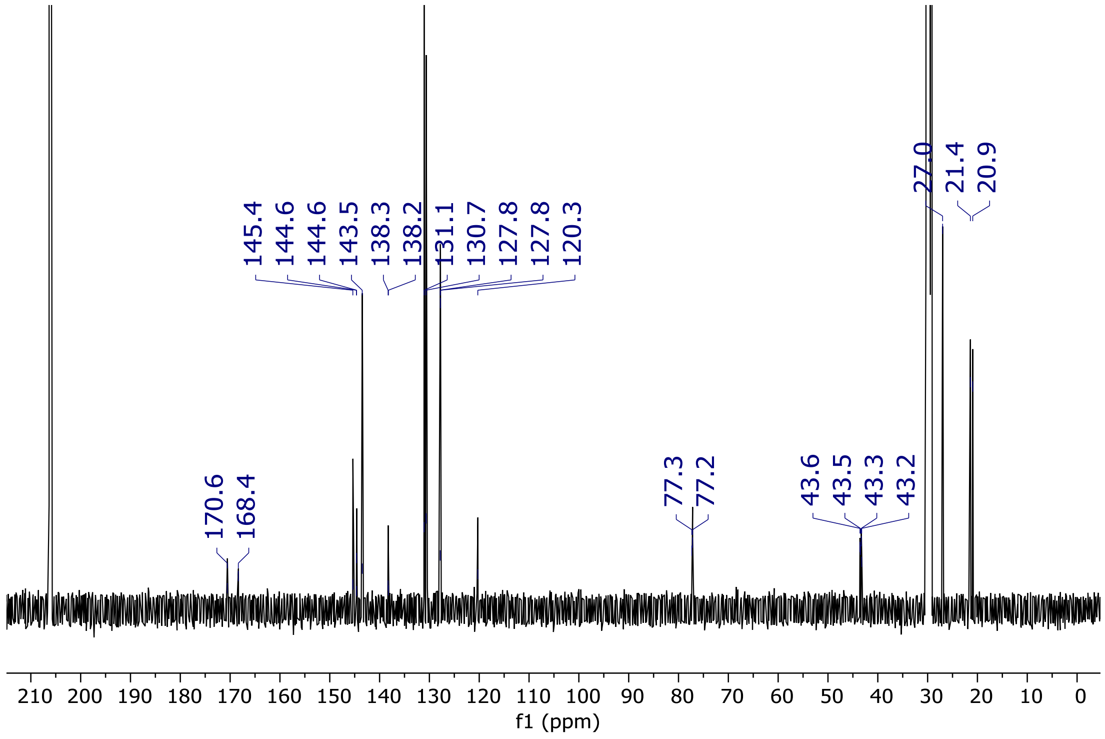

^{19}F NMR (376 MHz, $C(CD_3)_2O$):

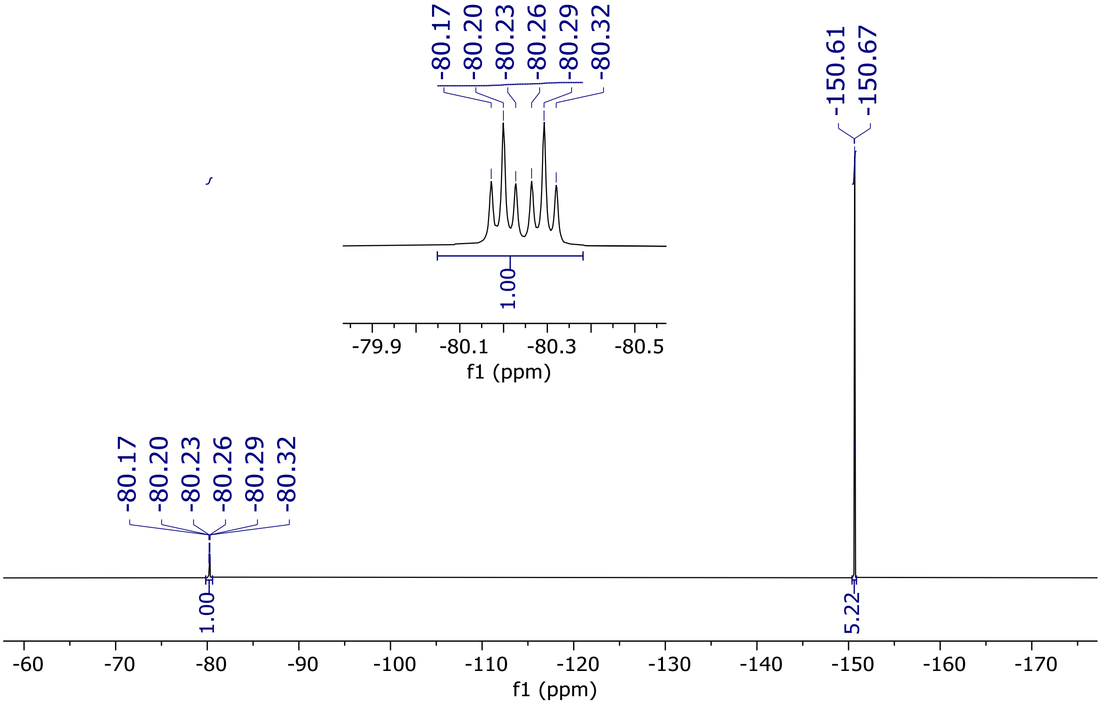

(Z)-(2-fluoro-3-((N-(4-fluorobenzyl)-4-methylphenyl)sulfonamido)prop-1-en-1-yl)(mesityl)iodonium BF_4 ***(3ac)***

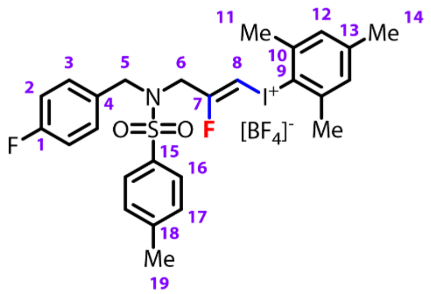

^{1}H NMR (500 MHz, CD_3CN):

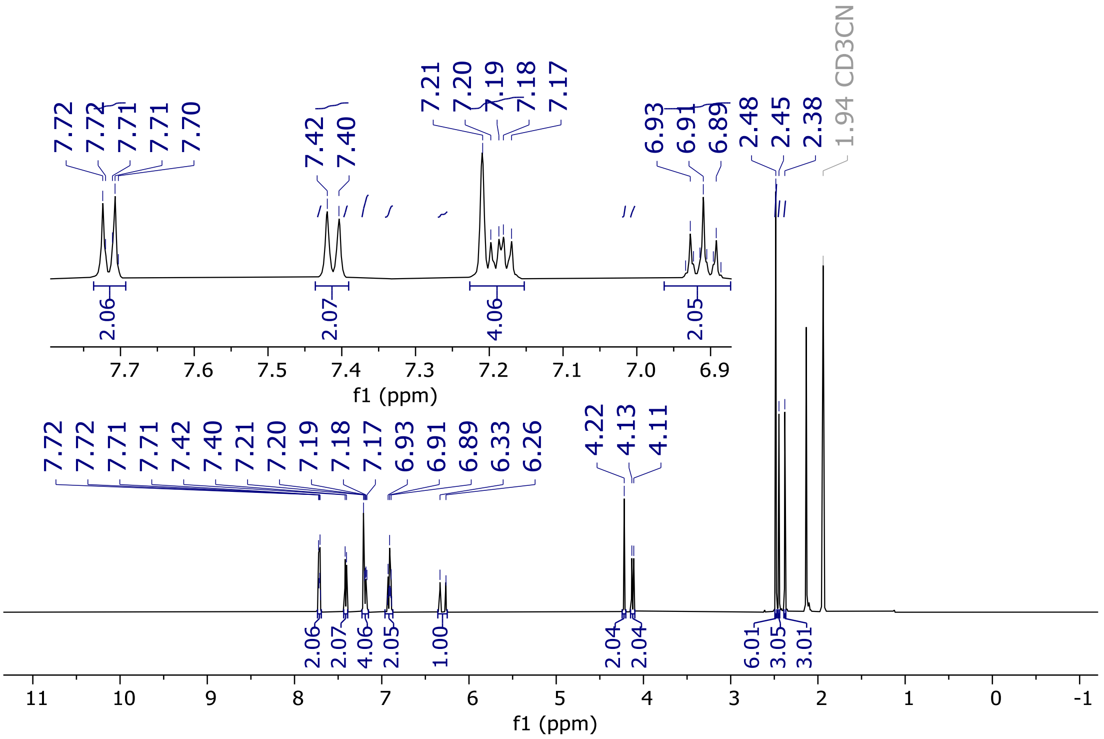

^{13}C NMR (126 MHz, CD_3CN):

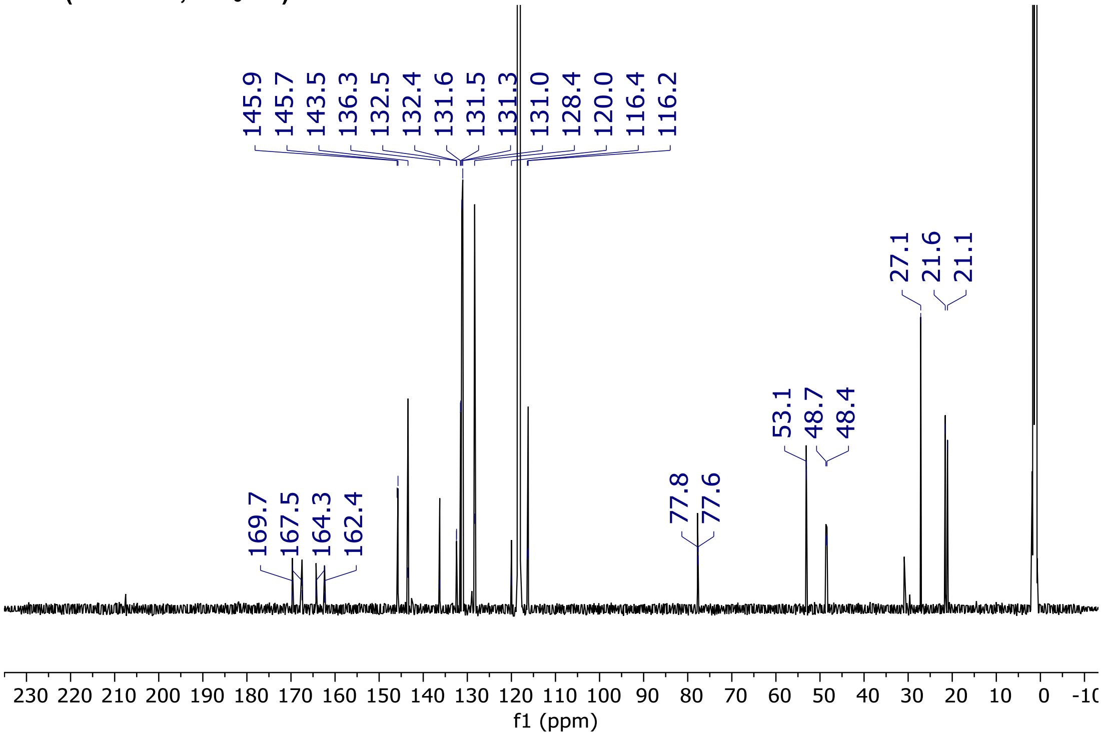

^{19}F NMR (376 MHz, CD_3CN):

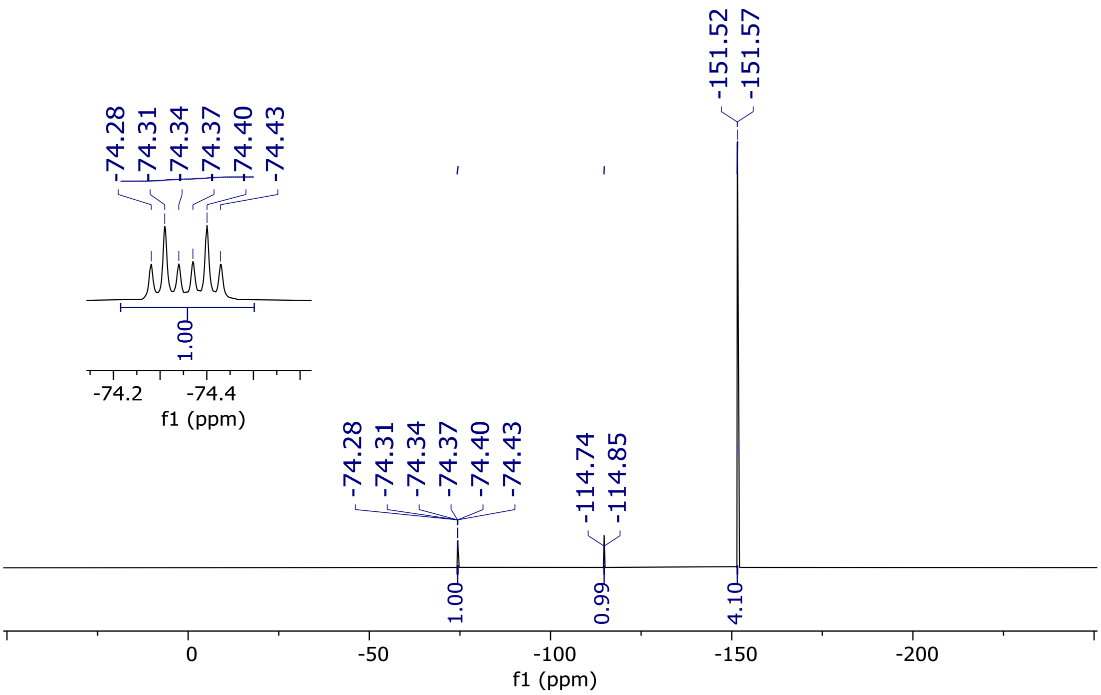

(Z)-(2-fluoro-3-((4-methyl-N-(4-methylbenzyl)phenyl)sulfonamido)prop-1-en-1-yl)(mesityl)iodonium BF_4 ***(3ad)***

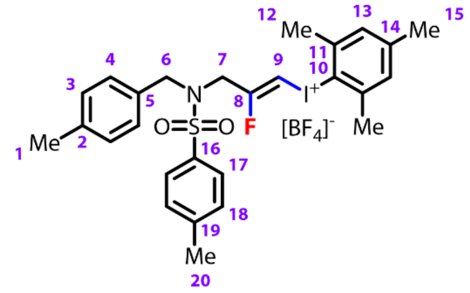

^{1}H NMR (500 MHz, CD_3CN):

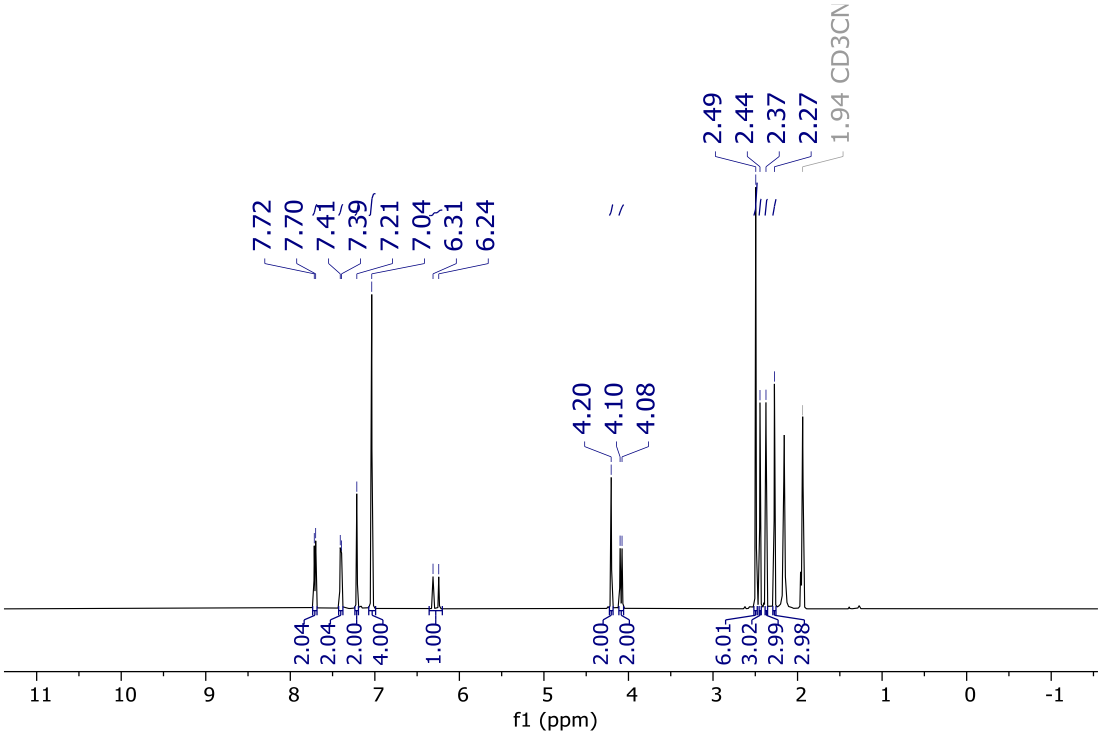

^{13}C NMR (126 MHz, CD_3CN):

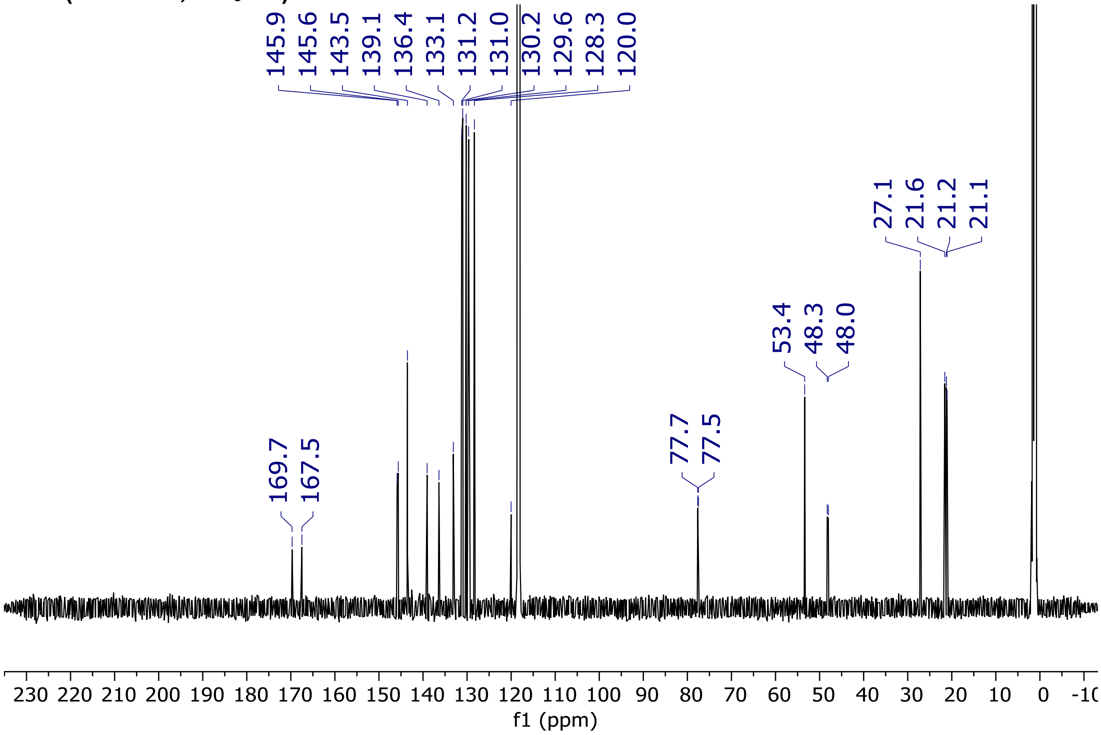

^{19}F NMR (376 MHz, CD_3CN):

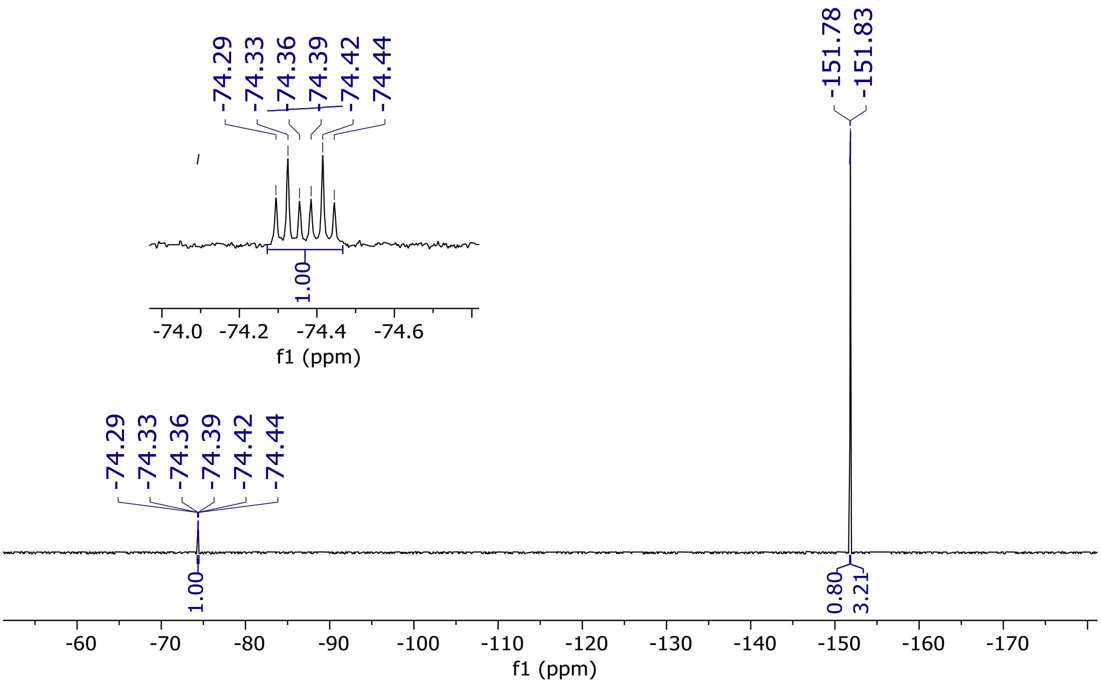

(Z)-(2-fluoro-3-((N-(4-fluorophenyl)-4-methylphenyl)sulfonamido)prop-1-en-1-yl)(mesityl)iodonium BF_4 ***(3ae)***

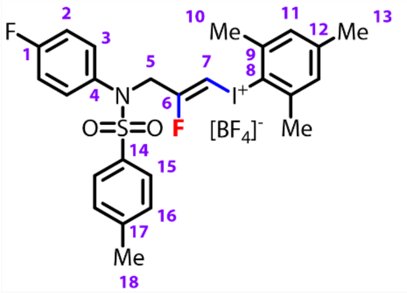

^{1}H NMR (500 MHz, $C(CD_3)_2O$):

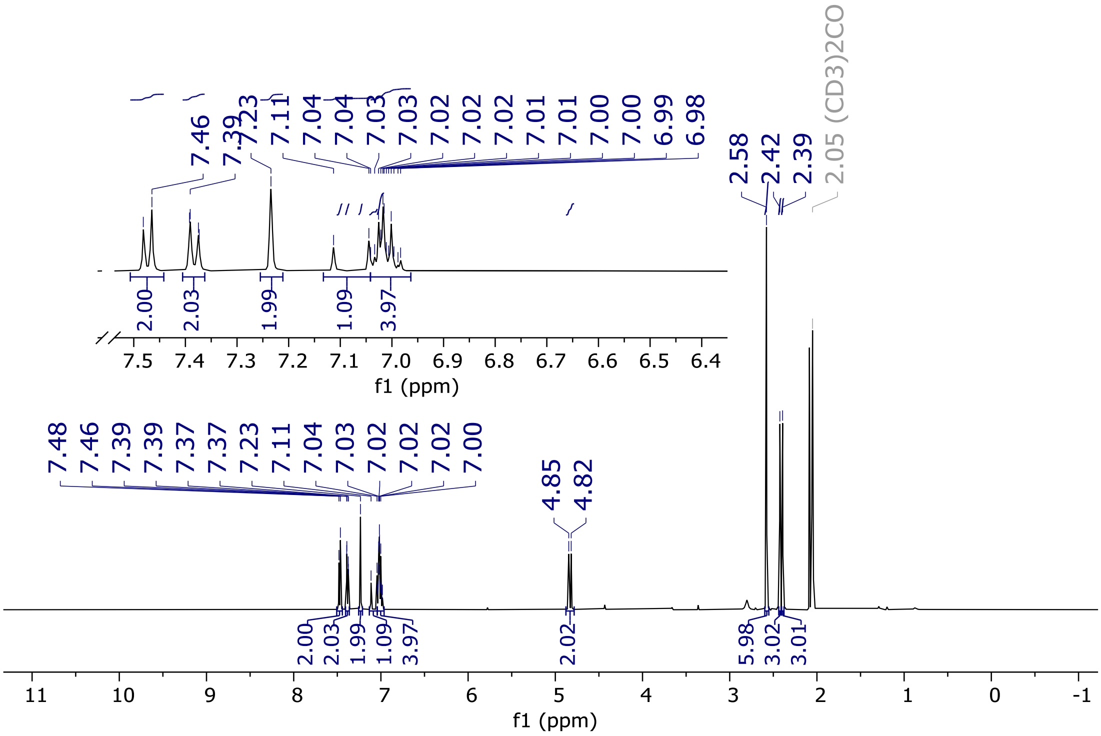

^{13}C NMR (126 MHz, $C(CD_3)_2O$):

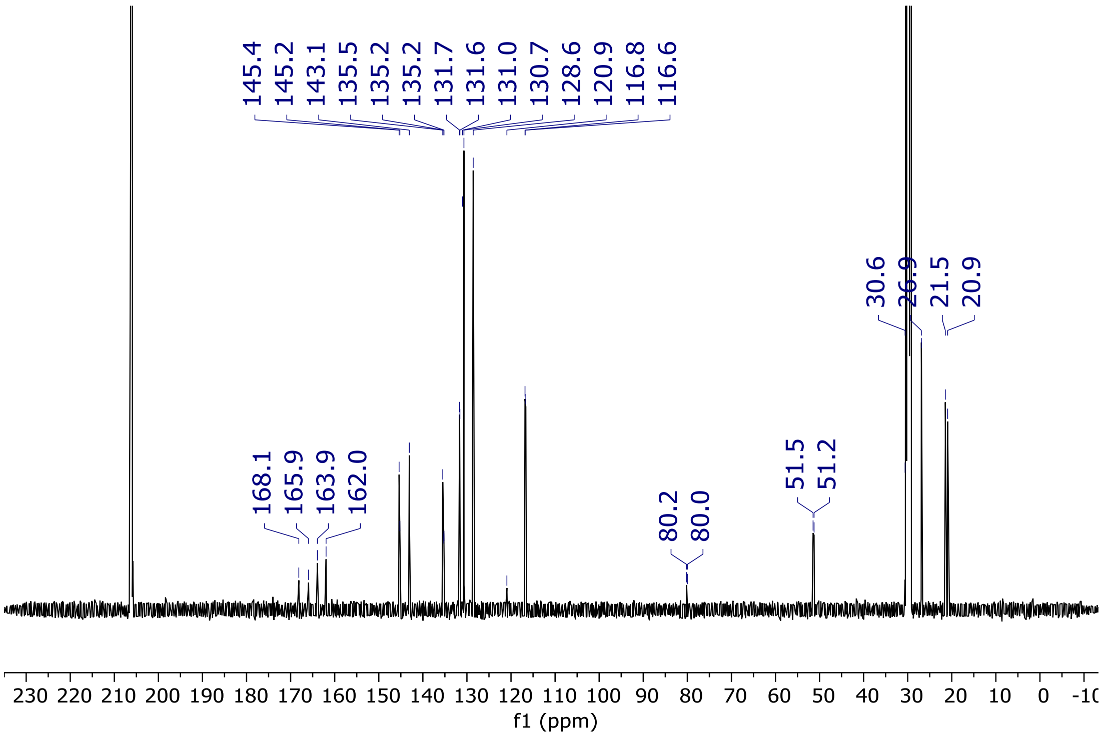

^{19}F NMR (376 MHz, $C(CD_3)_2O$):

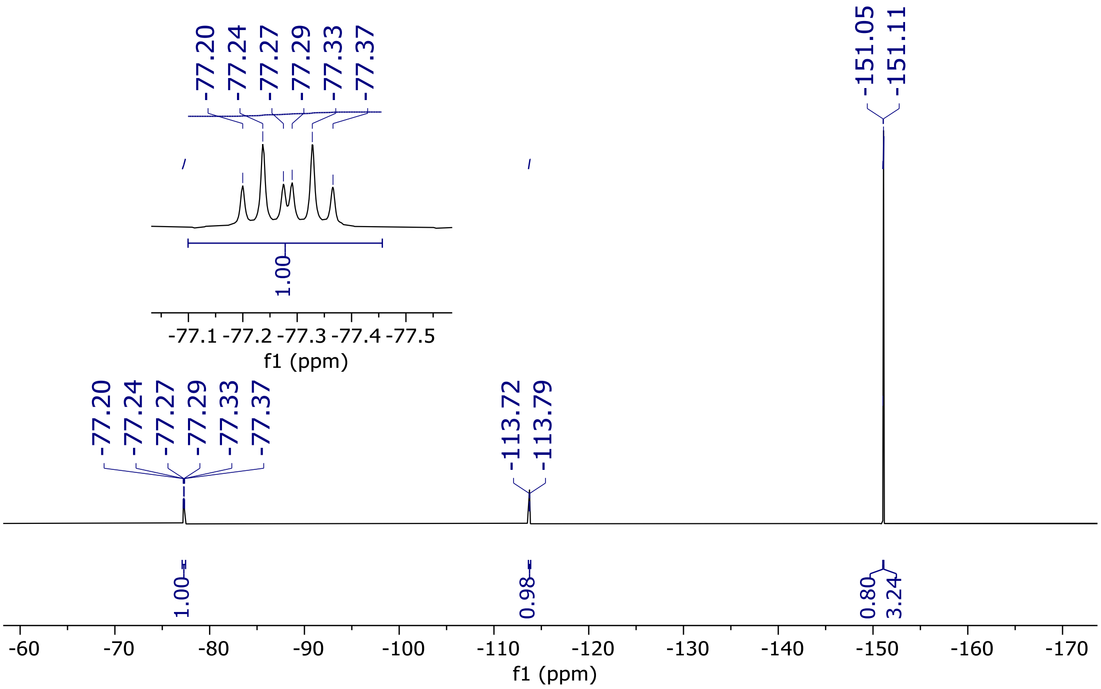

(Z)-(3-((N-(4-bromophenyl)-4-methylphenyl)sulfonamido)-2-fluoroprop-1-en-1-yl)(mesityl)iodonium BF_4 ***(3af)***

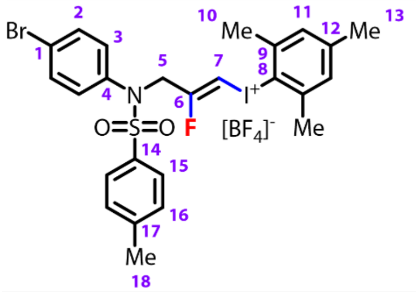

^{1}H NMR (600 MHz, $C(CD_3)_2O$):

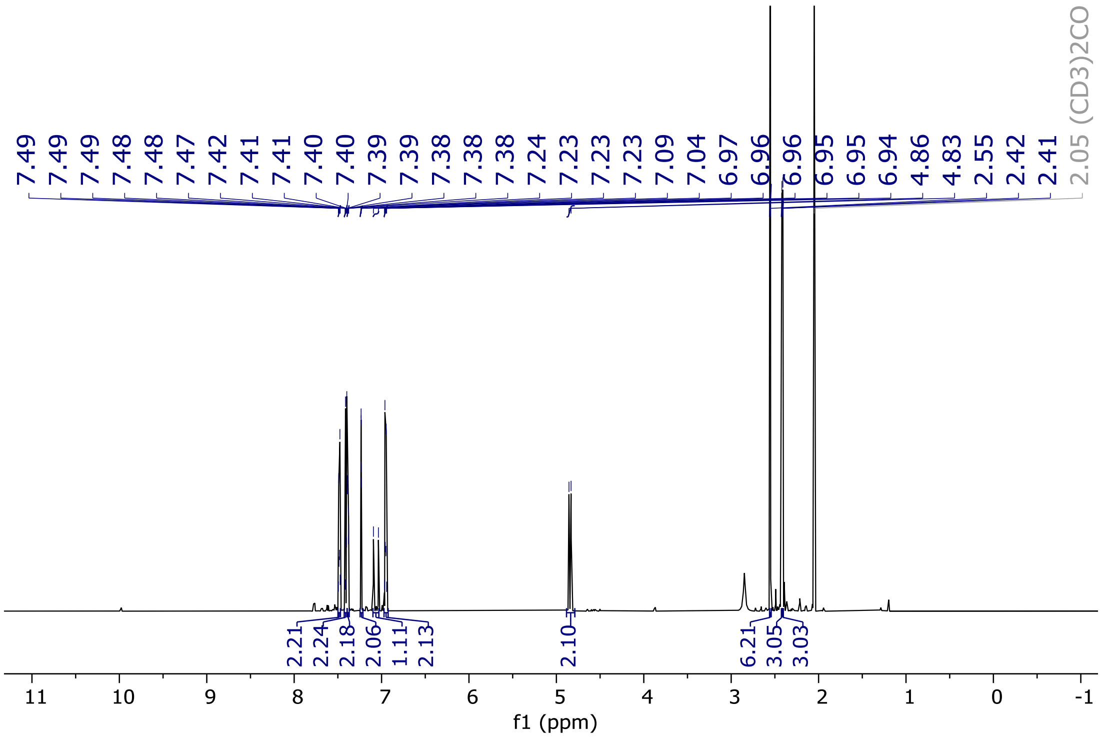

^{13}C NMR (151 MHz, $C(CD_3)_2O$):

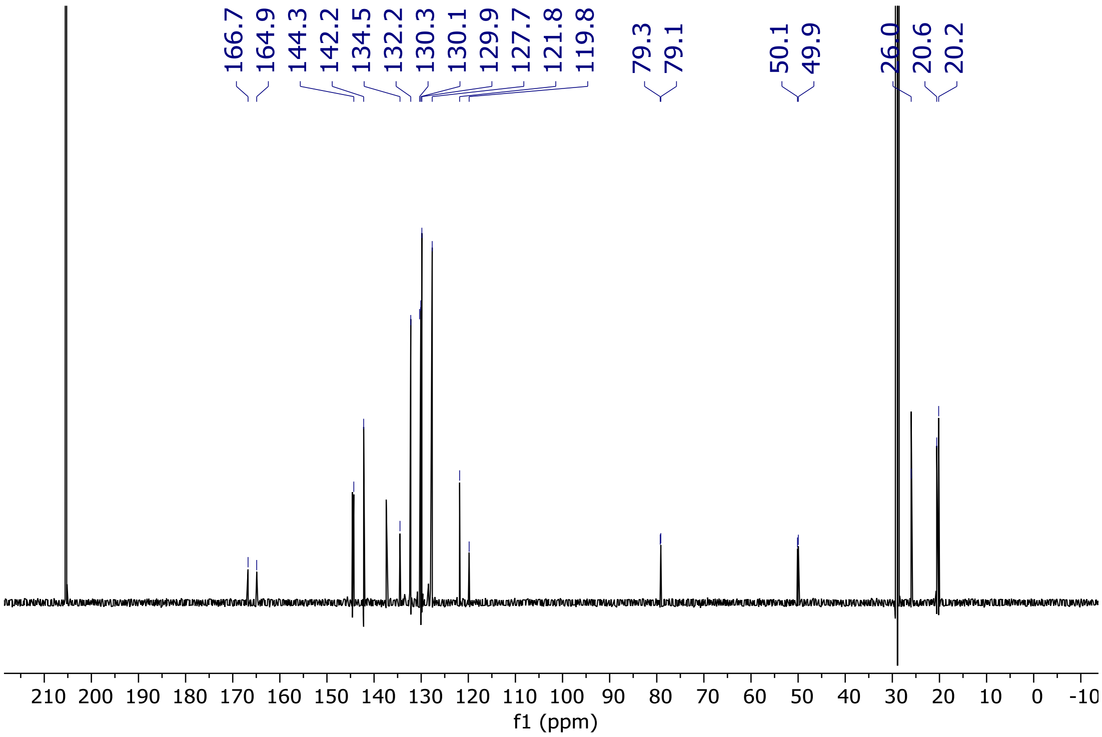

^{19}F NMR (376 MHz, $C(CD_3)_2O$):

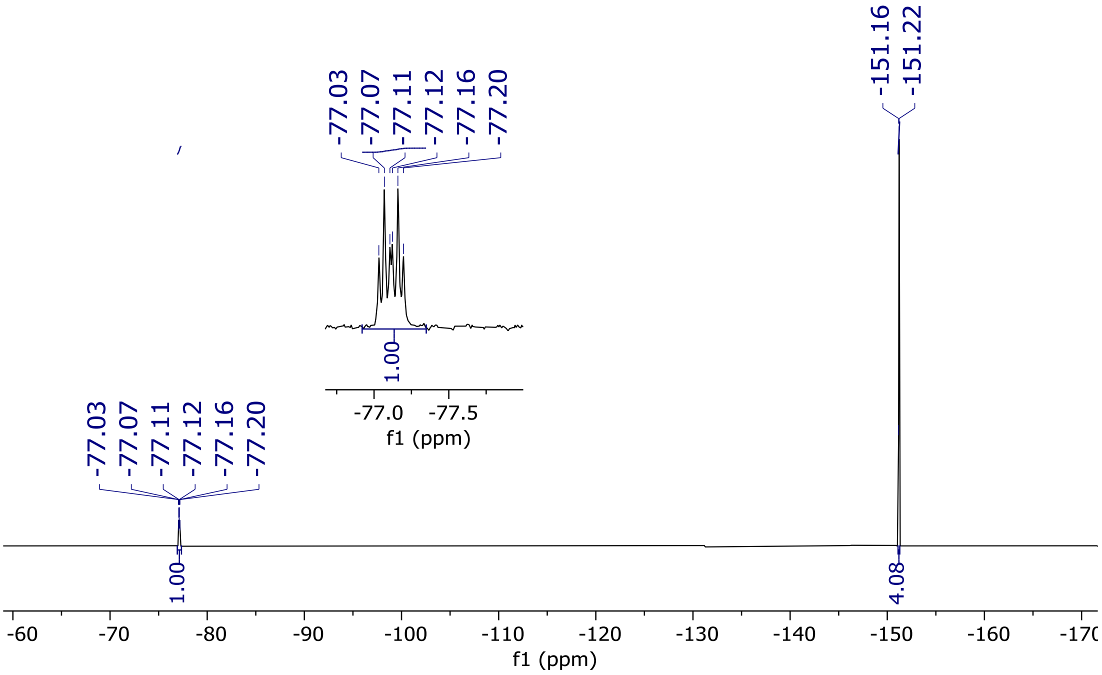

(Z)-(2-fluoro-2-(4-fluorophenyl)vinyl)(mesityl)iodonium BF_4 **(3ag)**

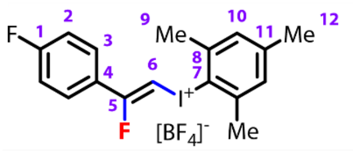

^{1}H NMR (500 MHz, CD_3CN):

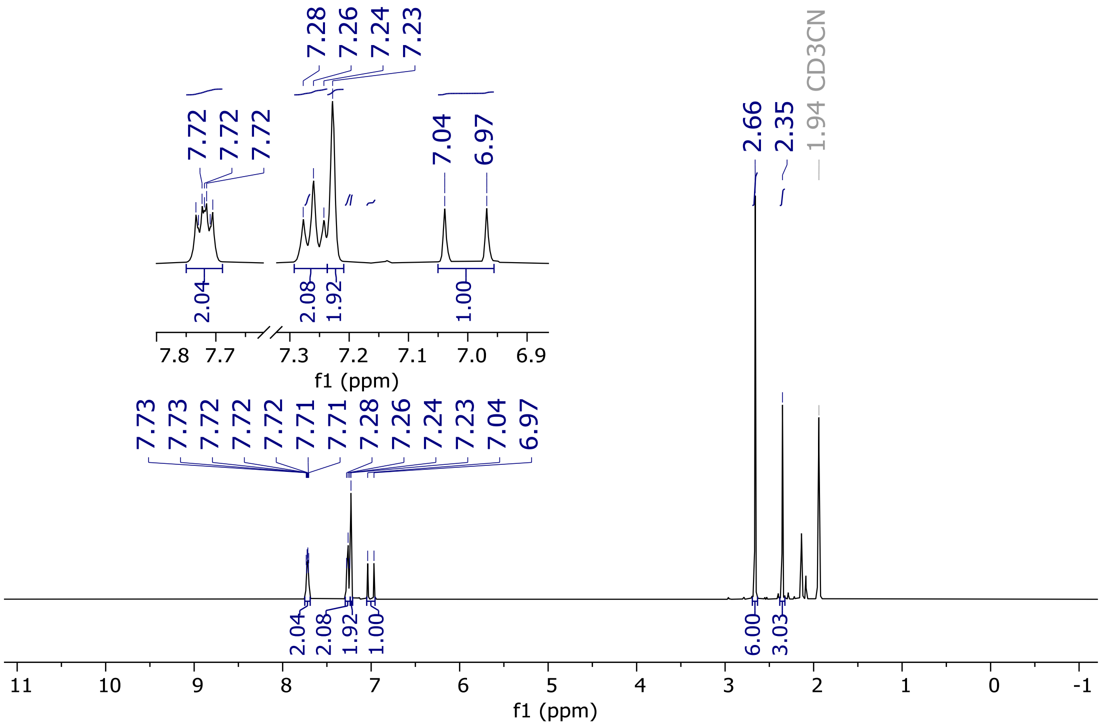

^{13}C NMR (126 MHz, CD_3CN):

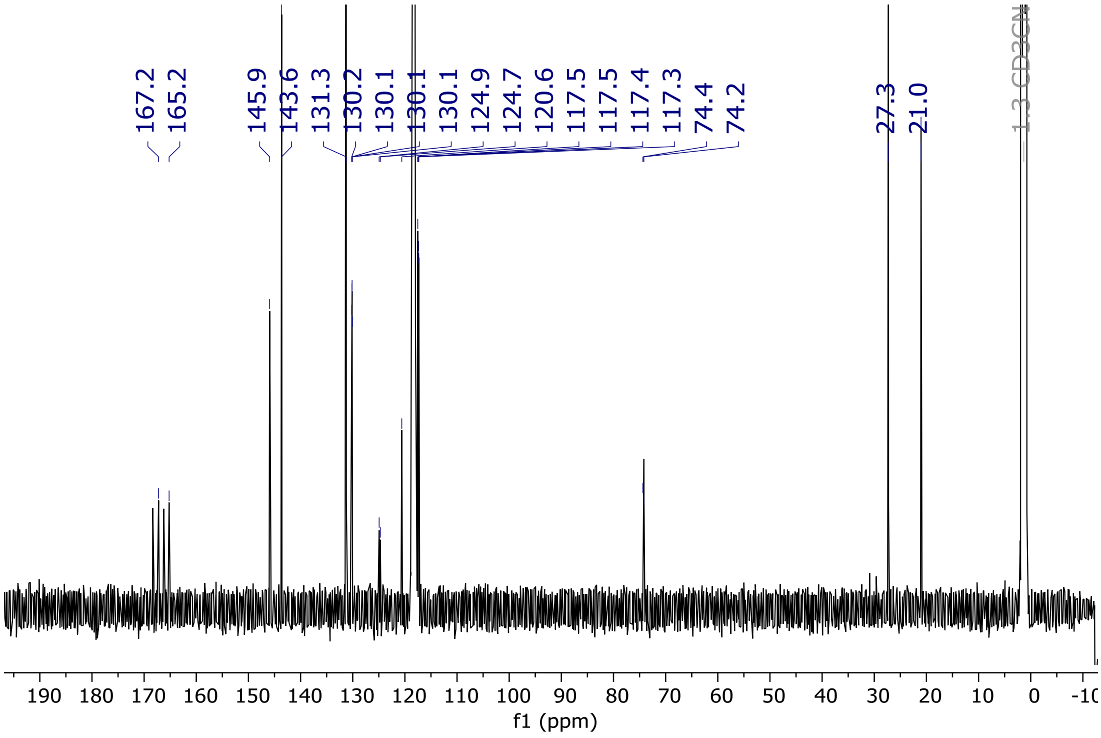

^{19}F NMR (376 MHz, CD_3CN):

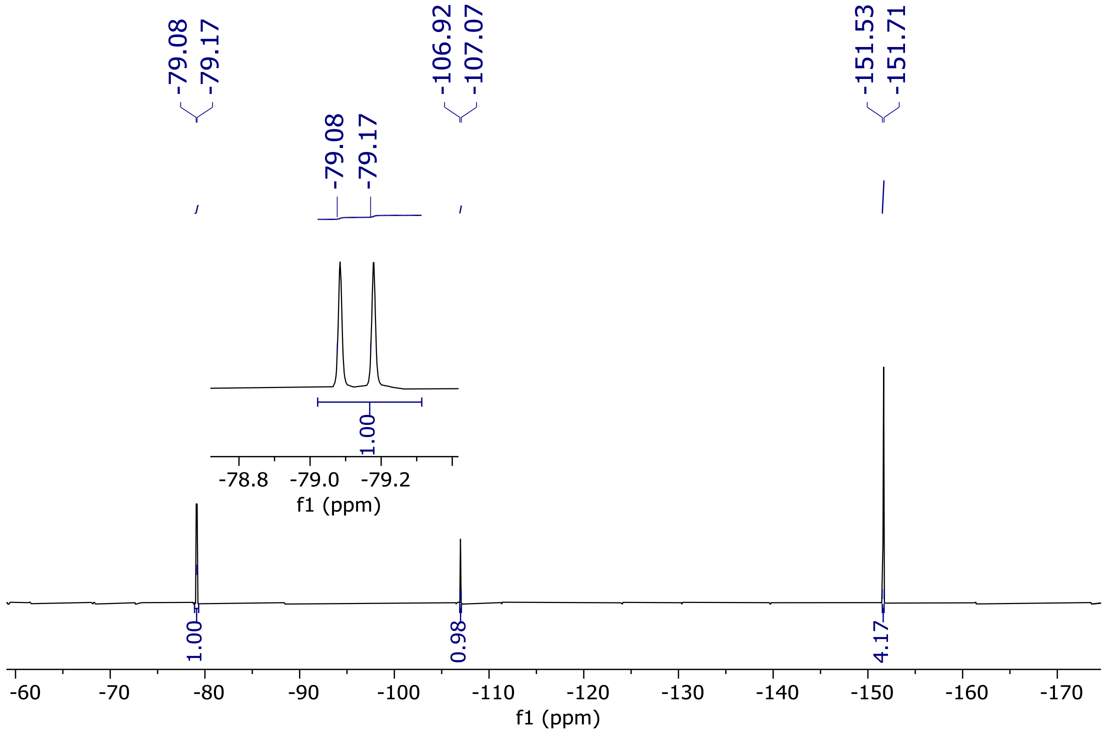

(Z)-(2-(4-bromophenyl)-2-fluorovinyl)(mesityl)iodonium BF_4 **(3ah)**

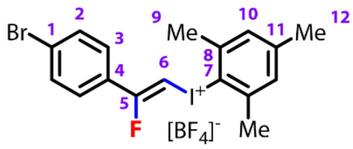

^{1}H NMR (500 MHz, $C(CD_3)_2O$):

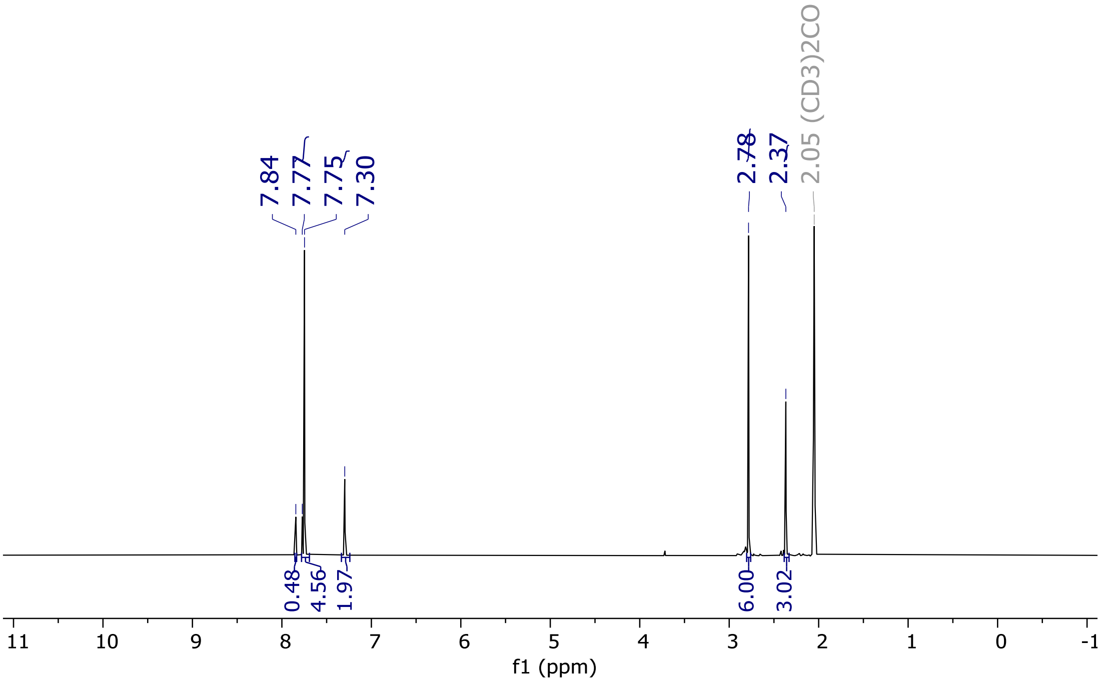

^{13}C NMR (126 MHz, $C(CD_3)_2O$):

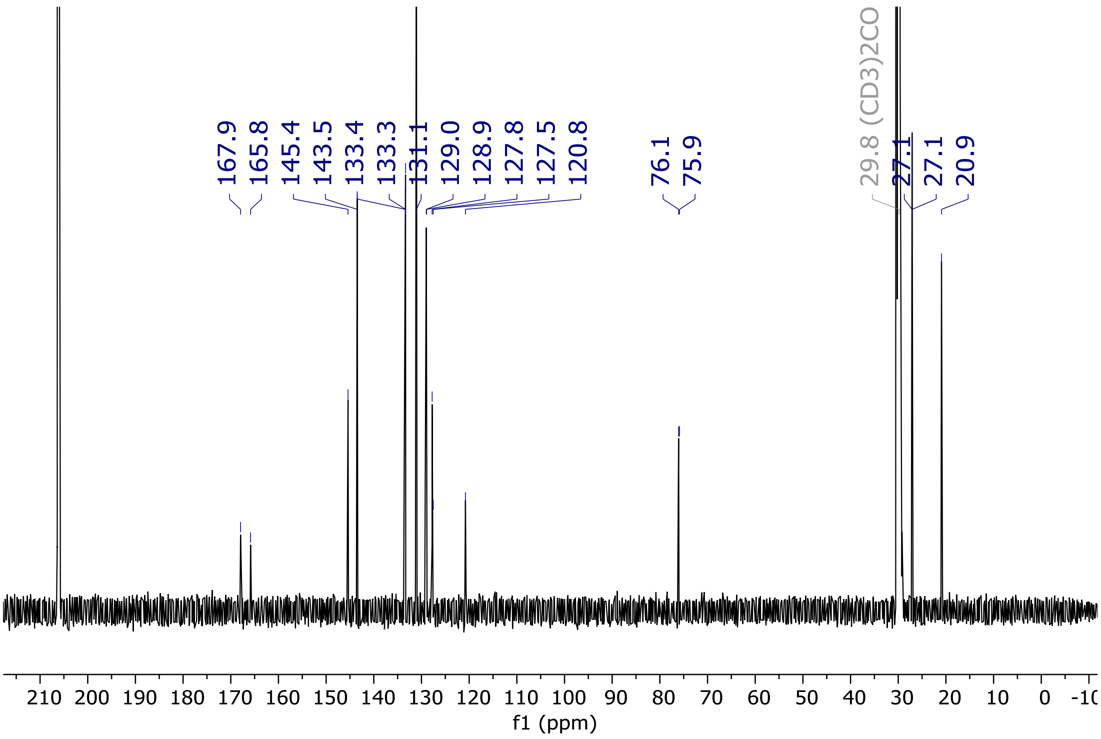

^{19}F NMR (376 MHz, $C(CD_3)_2O$):

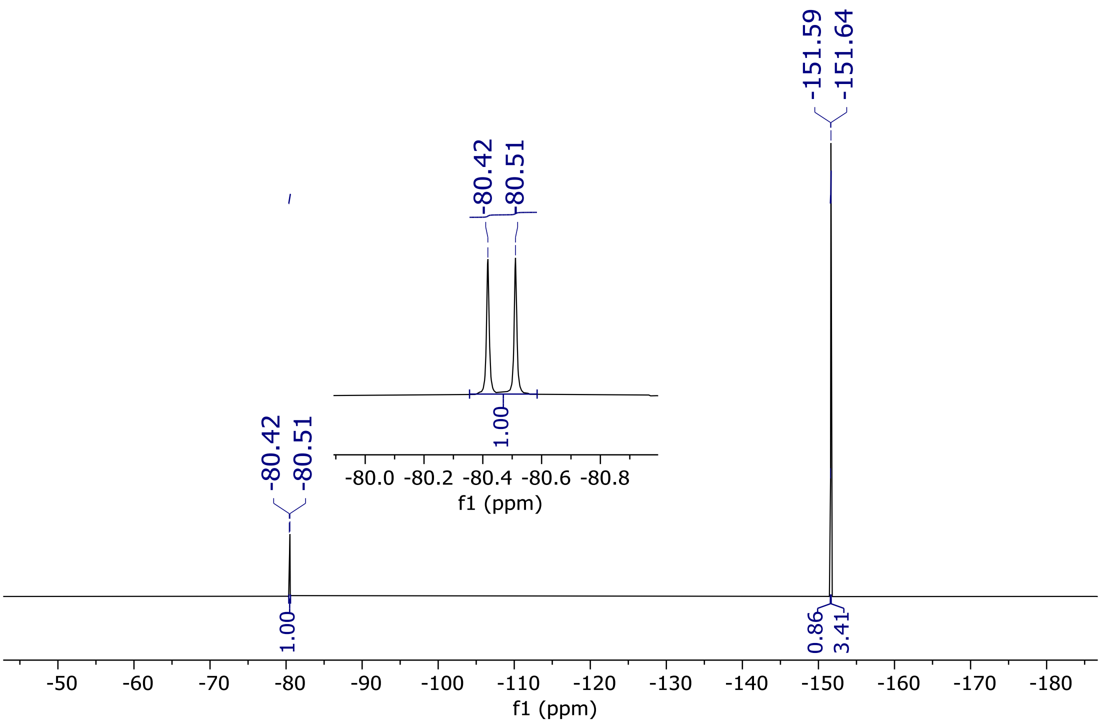

(Z)-(2-fluoro-2-(4-(trifluoromethyl)phenyl)vinyl)(mesityl)iodonium BF_4 ***(3ai)***

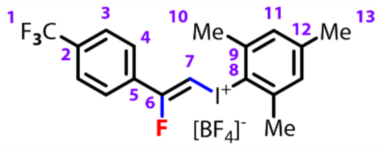

^{1}H NMR (500 MHz, $C(CD_3)_2O$):

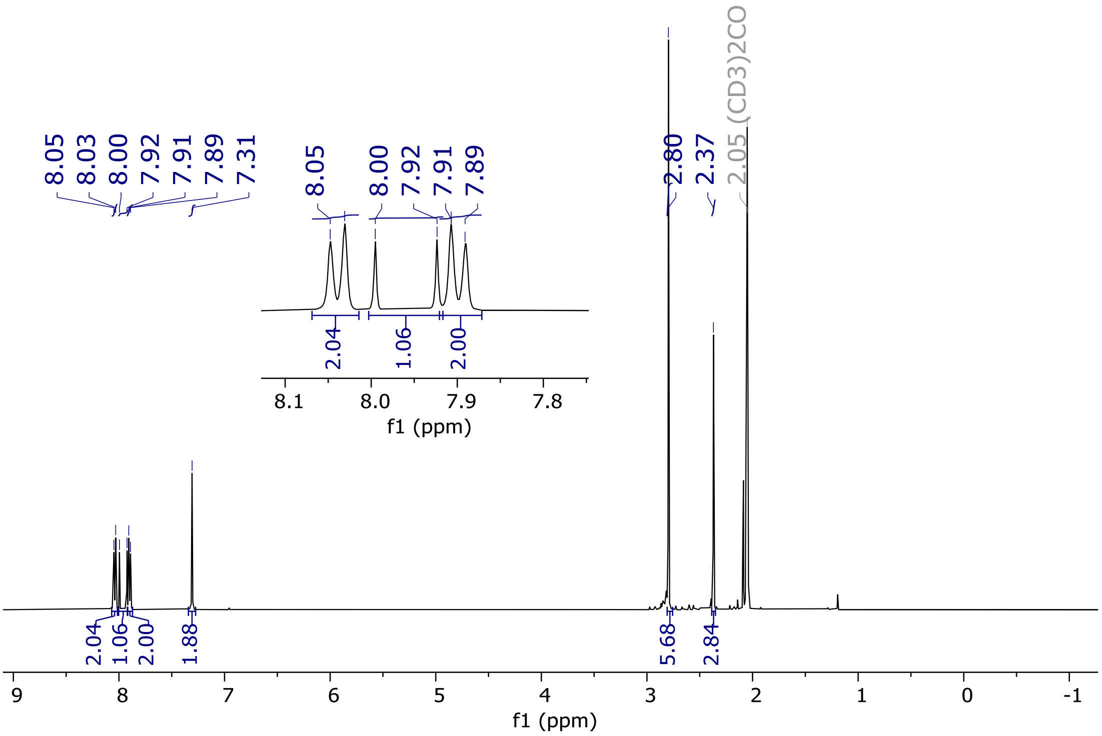

^{13}C NMR (126 MHz, $C(CD_3)_2O$):

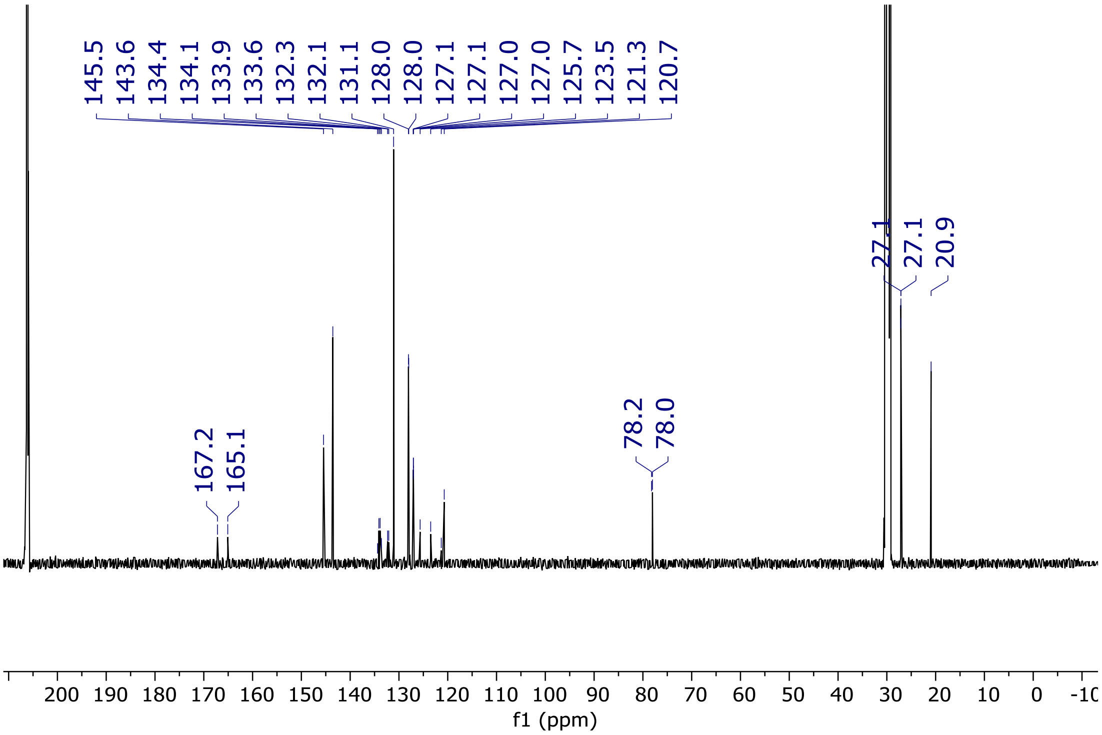

^{19}F NMR (376 MHz, $C(CD_3)_2O$):

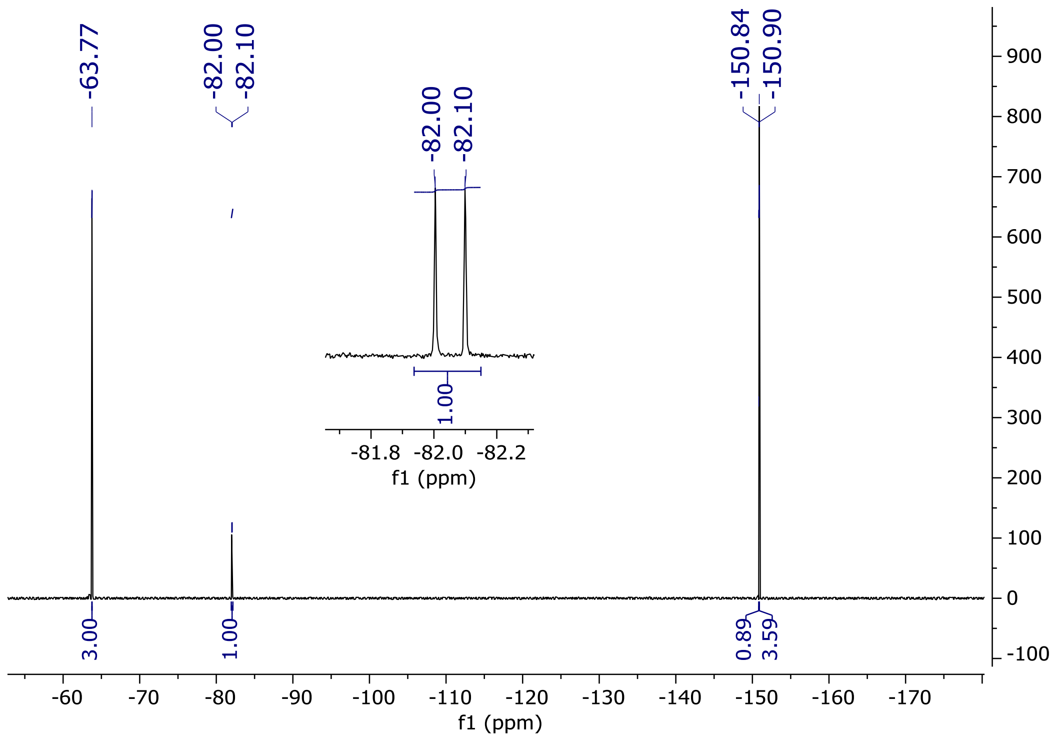

(Z)-(2-fluoro-2-(4-nitrophenyl)vinyl)(mesityl)iodonium BF_4 ***(3aj)***

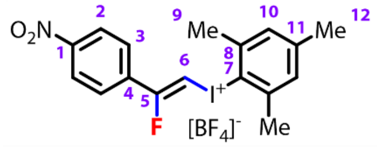

^{1}H NMR (500 MHz, $C(CD_3)_2O$):

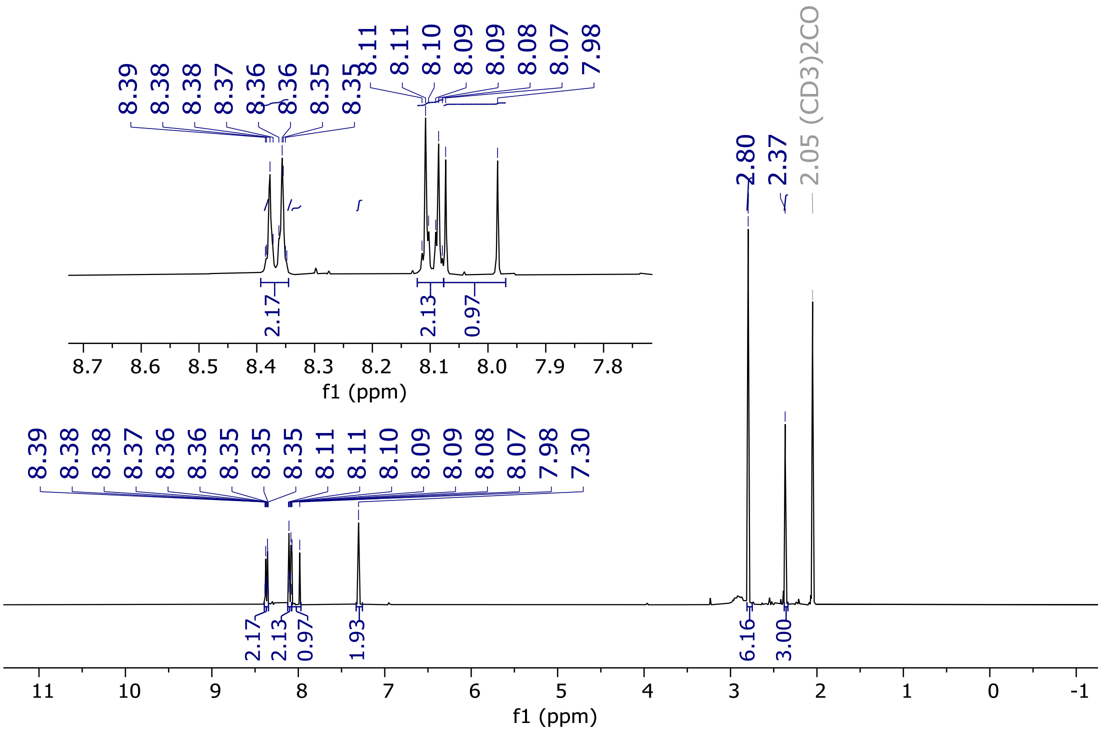

^{13}C NMR (126 MHz, $C(CD_3)_2O$):

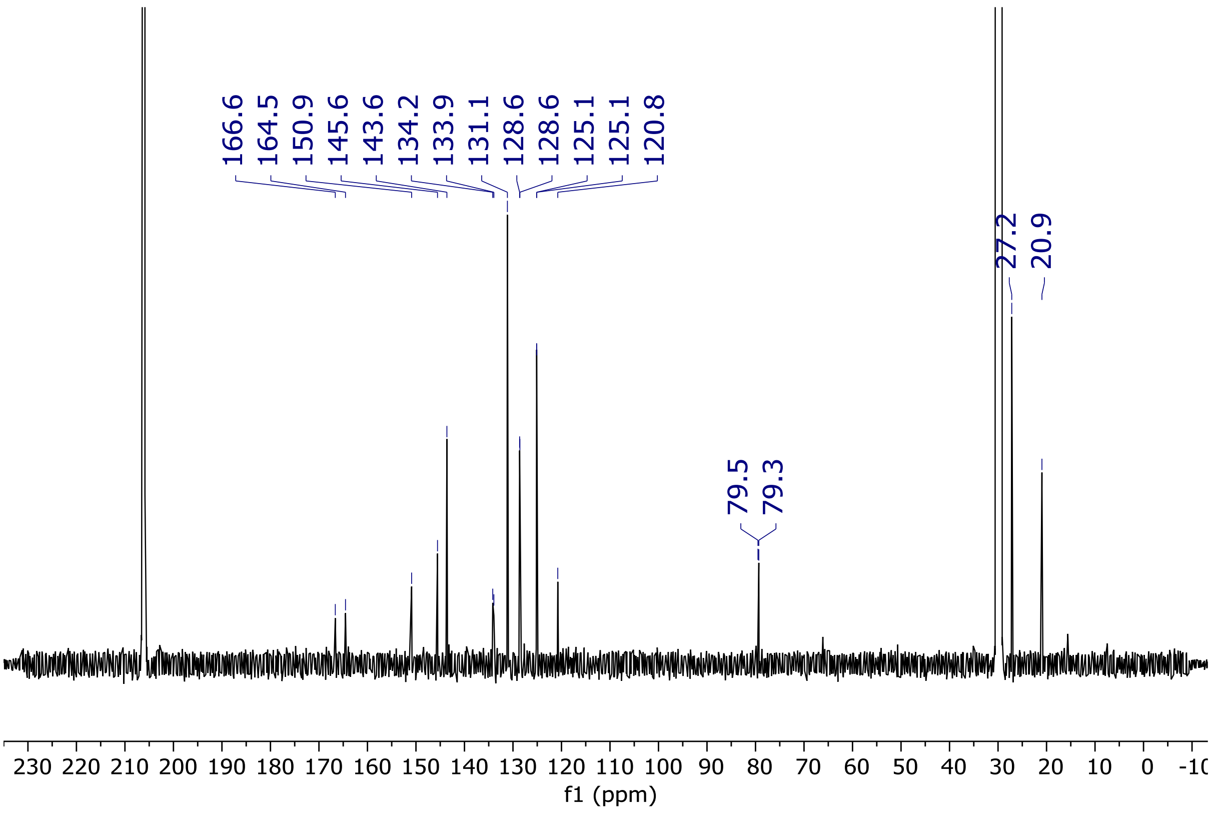

^{19}F NMR (376 MHz, $C(CD_3)_2O$):

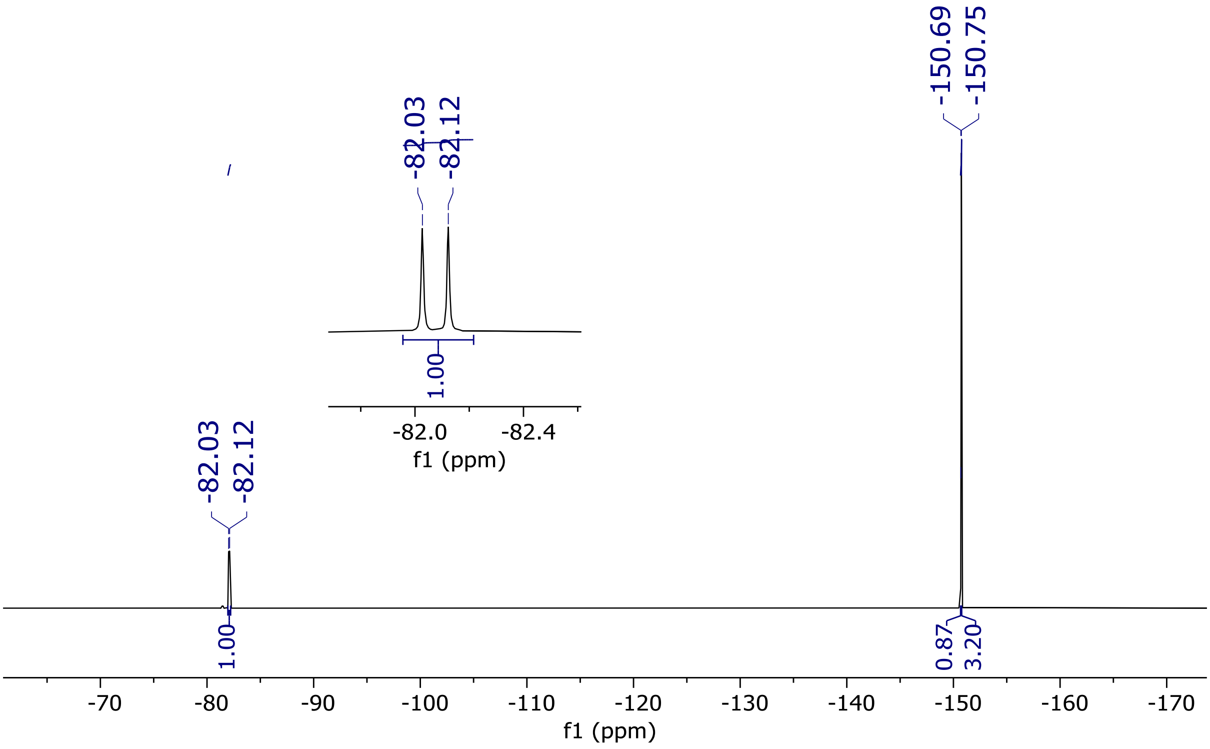

(Z)-(2-fluoro-2-(4-methoxyphenyl)vinyl)(mesityl)iodonium BF_4
(3ak)

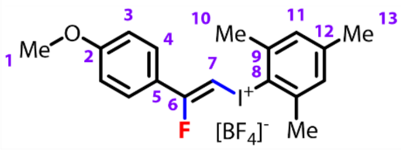

^{1}H NMR (500 MHz, $C(CD_3)_2O$):

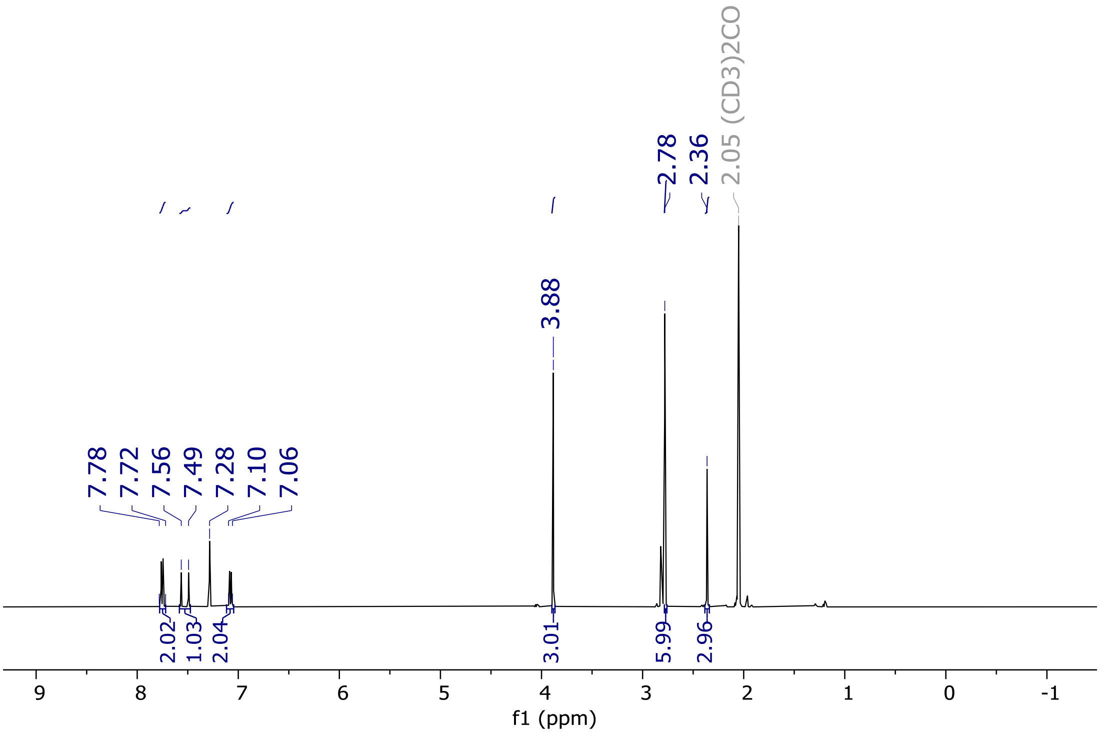

^{13}C NMR (126 MHz, $C(CD_3)_2O$):

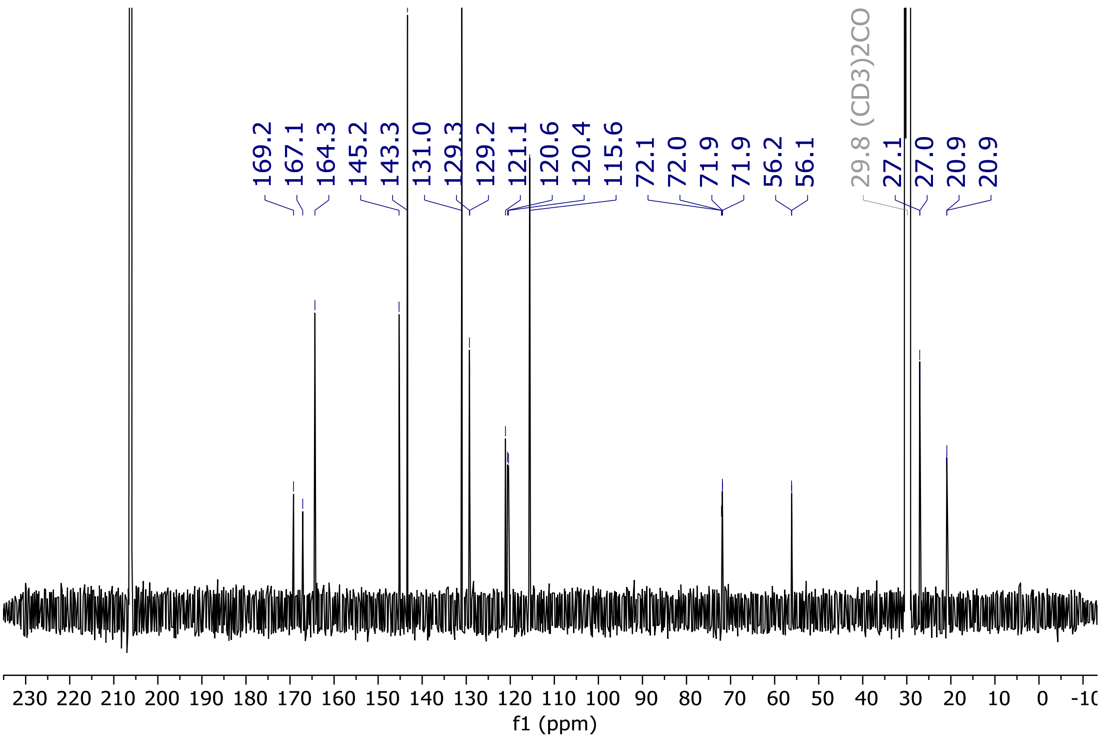

^{19}F NMR (377 MHz, $C(CD_3)_2O$):

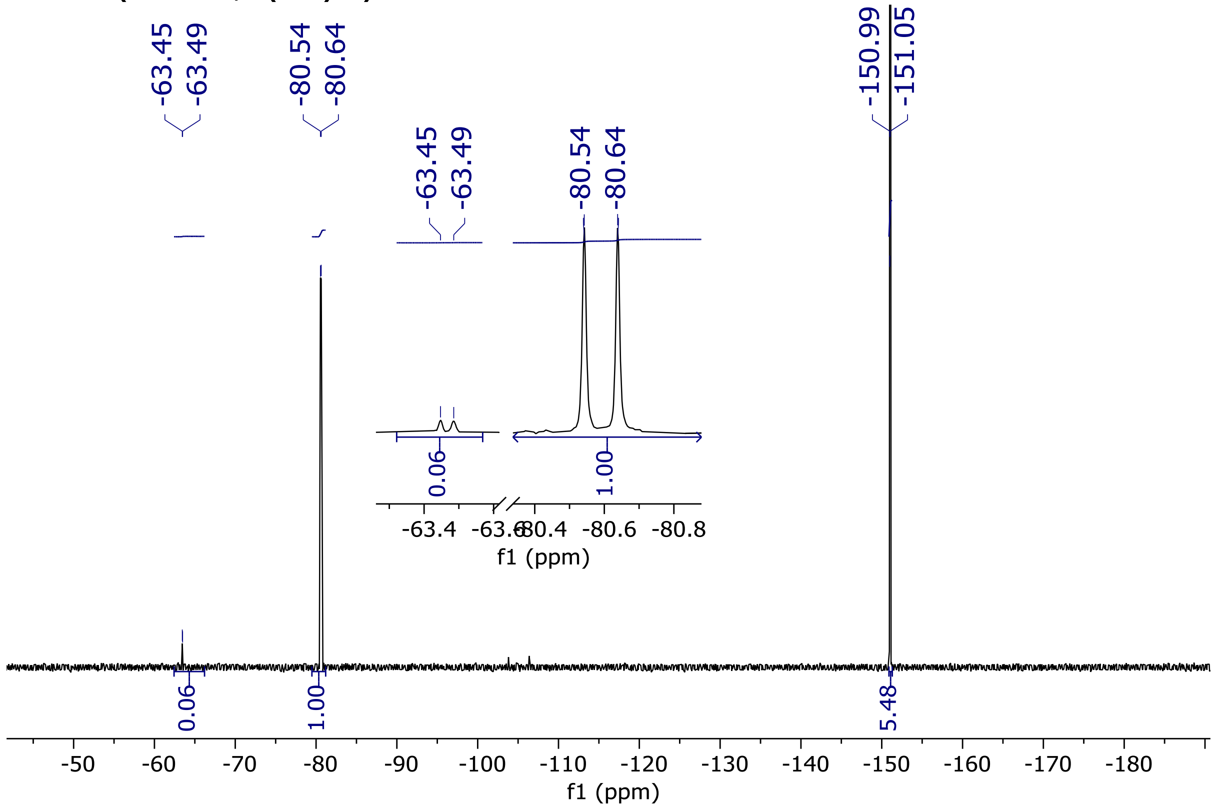

(Z)-(2-(4-acetylphenyl)-2-fluorovinyl)(mesityl)iodonium BF_4 **(3al)**

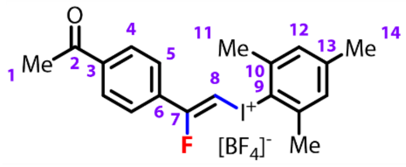

^{1}H NMR (500 MHz, $C(CD_3)_2O$):

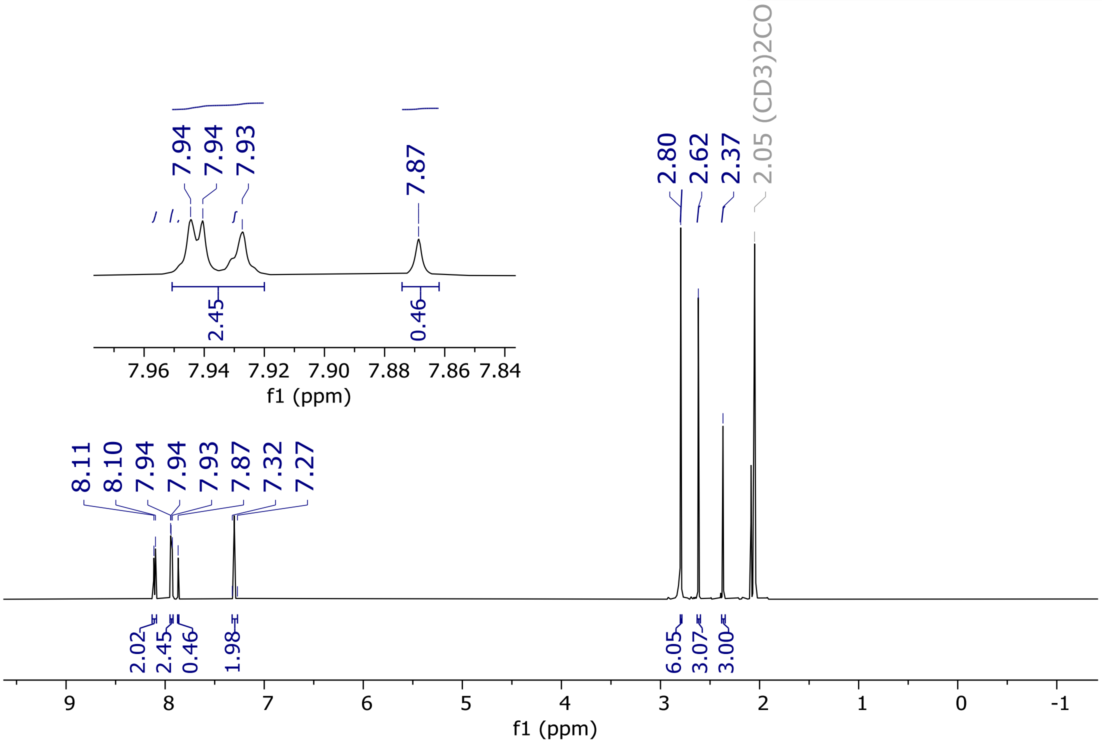

^{13}C NMR (126 MHz, $C(CD_3)_2O$):

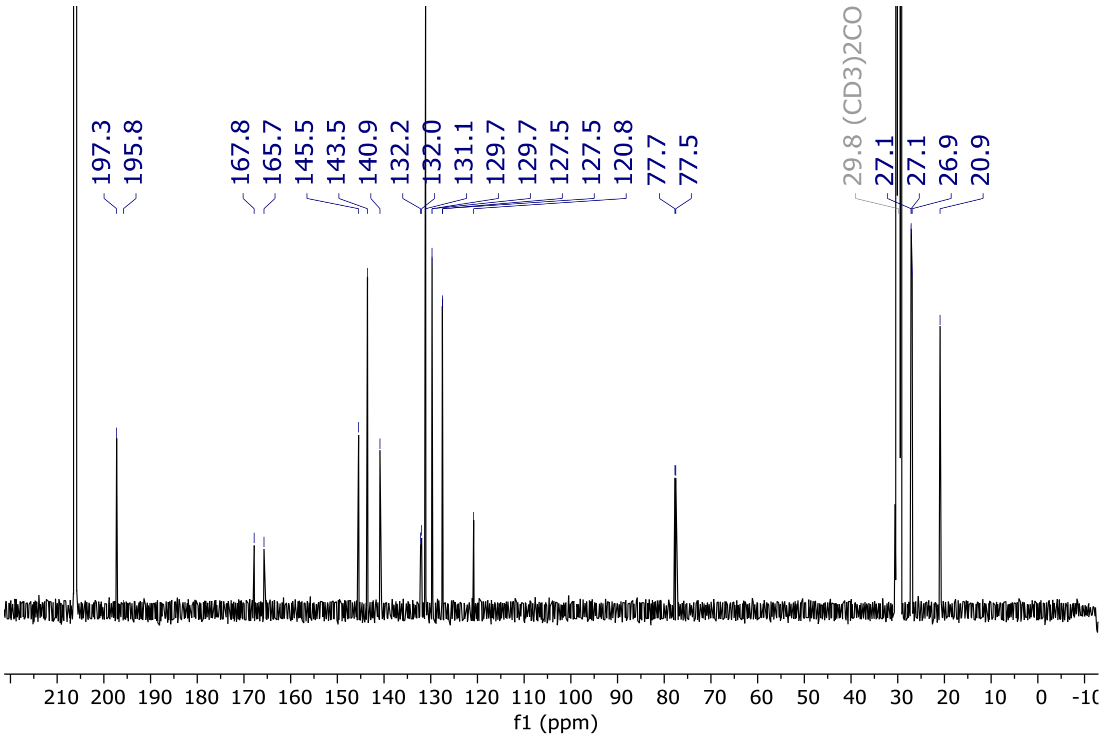

^{19}F NMR (376 MHz, $C(CD_3)_2O$):

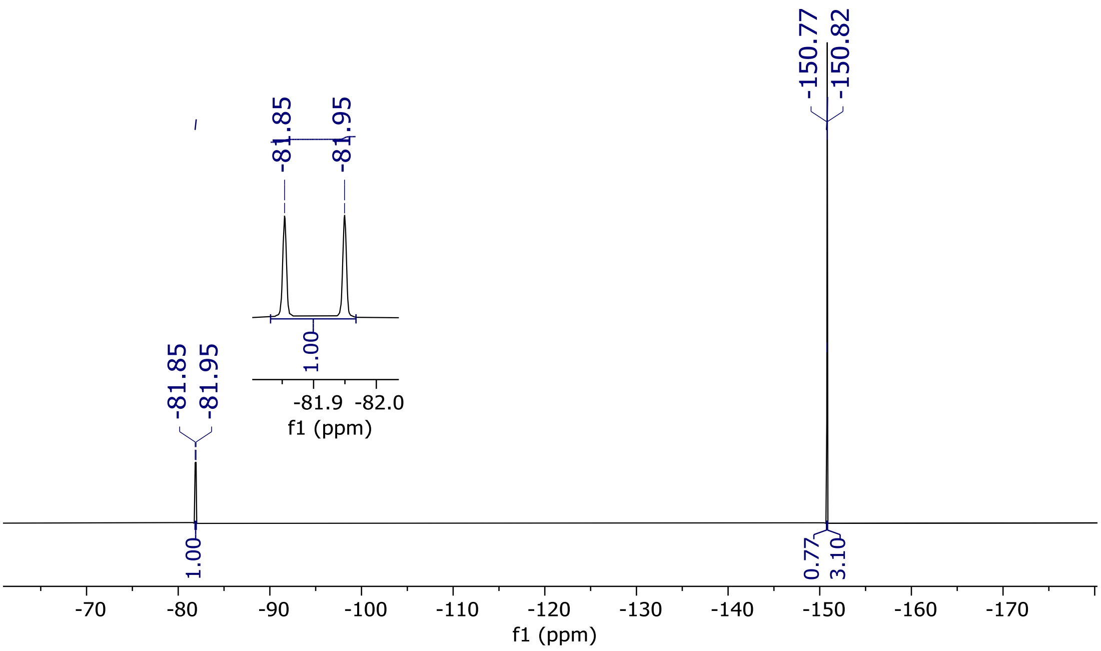

(Z)-(2-fluoro-2-(4-formylphenyl)vinyl)(mesityl)iodonium BF_4 **(3am)**

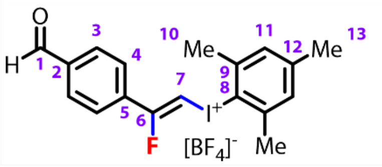

^{1}H NMR (500 MHz, $C(CD_3)_2O$):

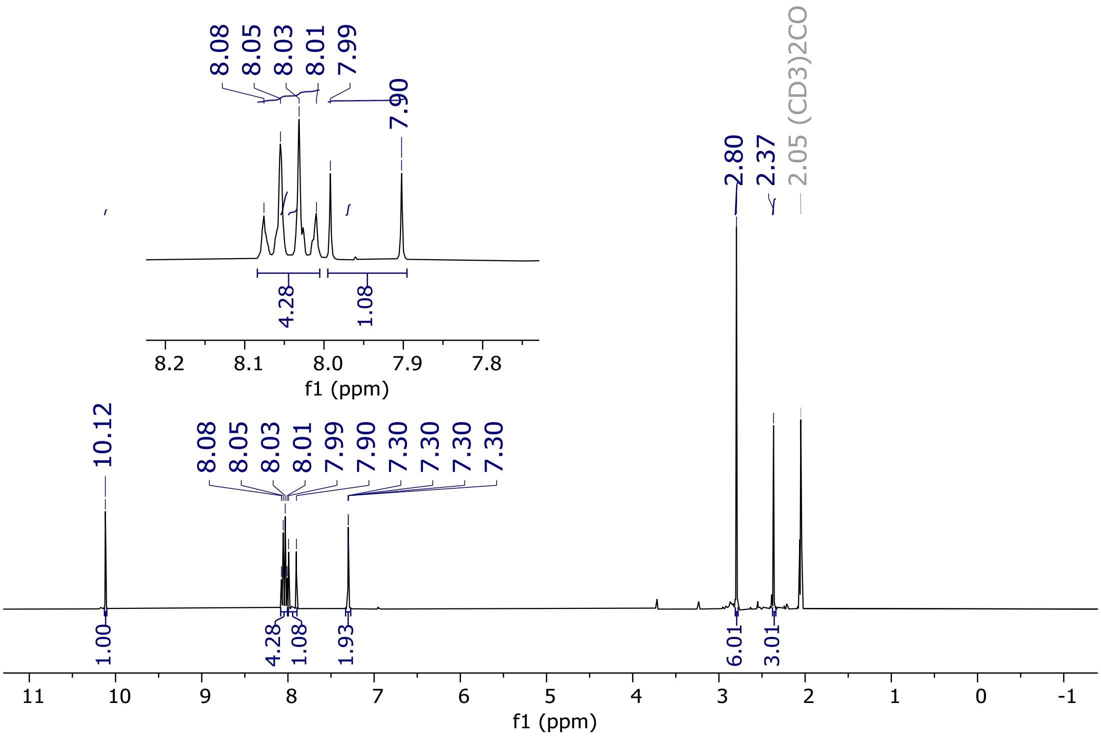

^{13}C NMR (126 MHz, $C(CD_3)_2O$):

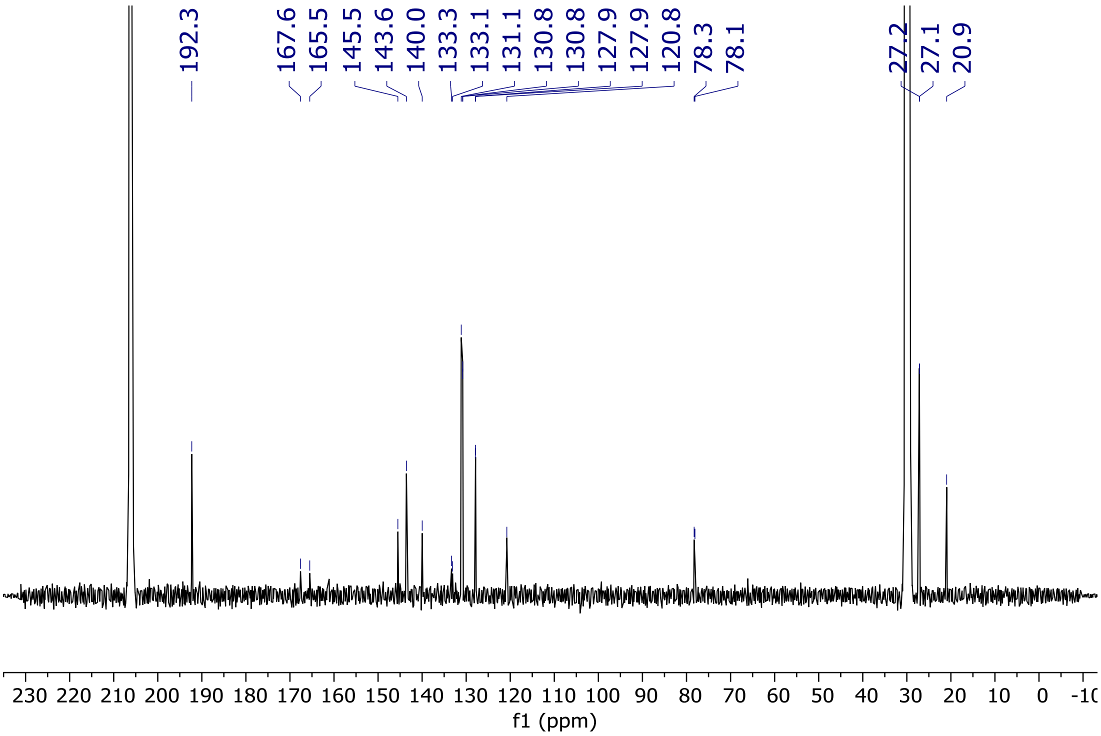

^{19}F NMR (376 MHz, $C(CD_3)_2O$):

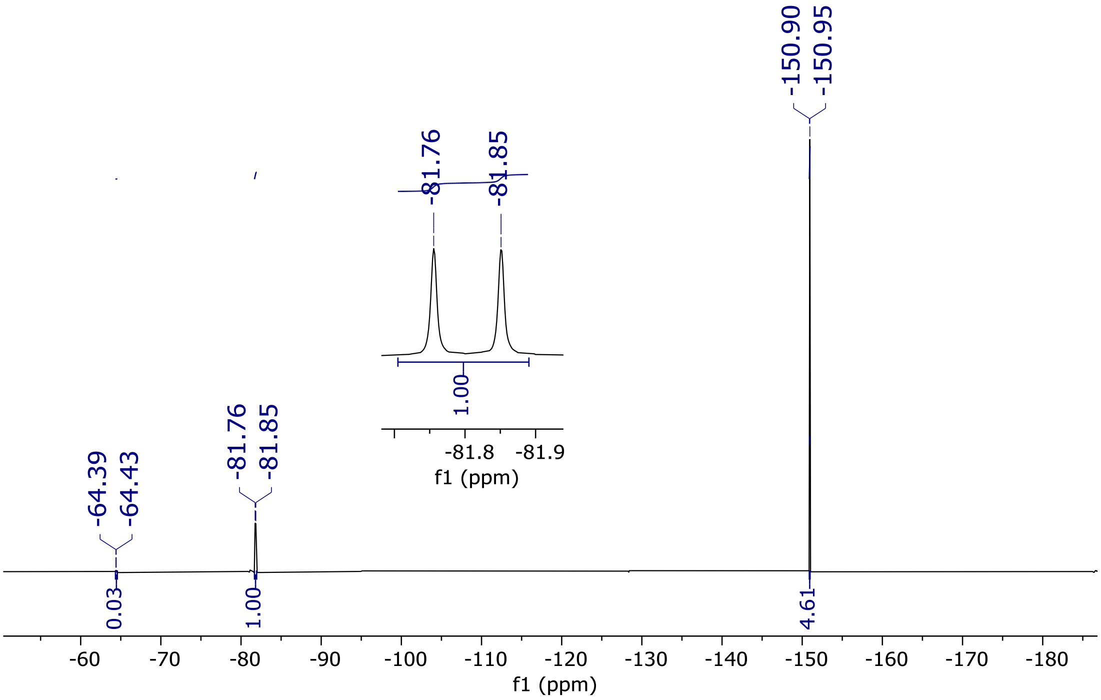

(Z)-(2-fluoro-2-(pyridin-2-yl)vinyl)(mesityl)iodonium BF_4 ***(3an)***

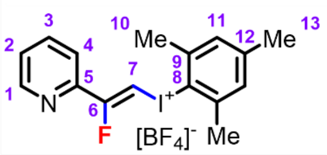

^{1}H NMR (500 MHz, CD_3CN):

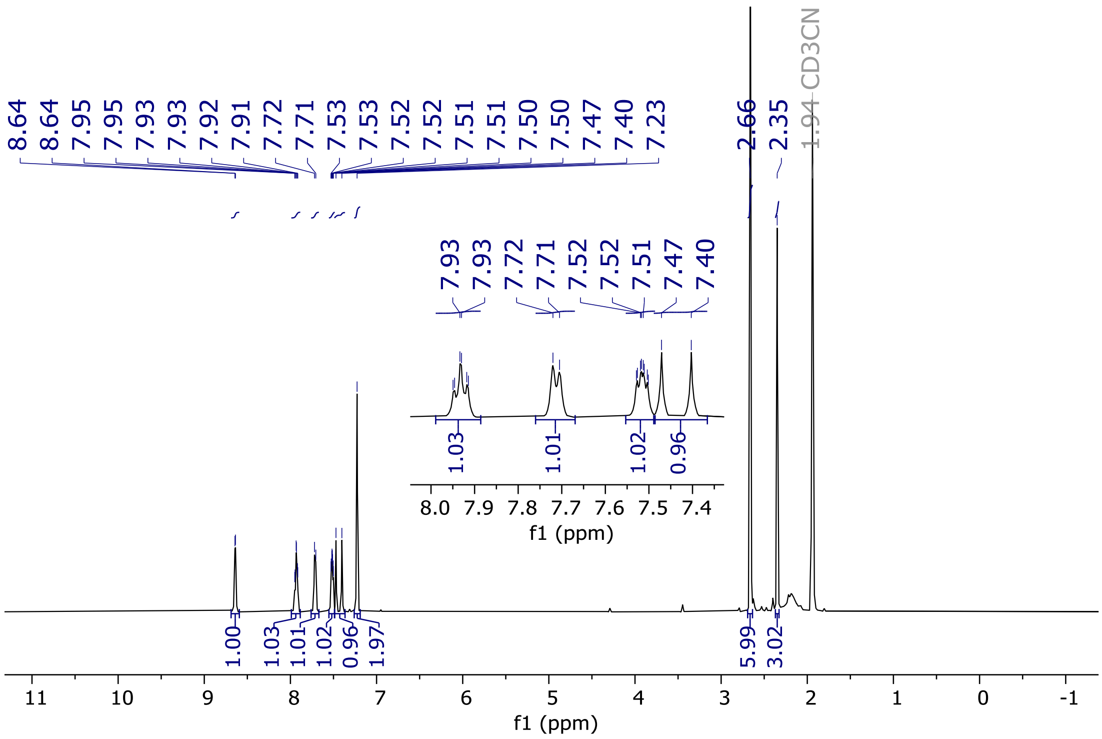

^{13}C NMR (126 MHz, CD_3CN):

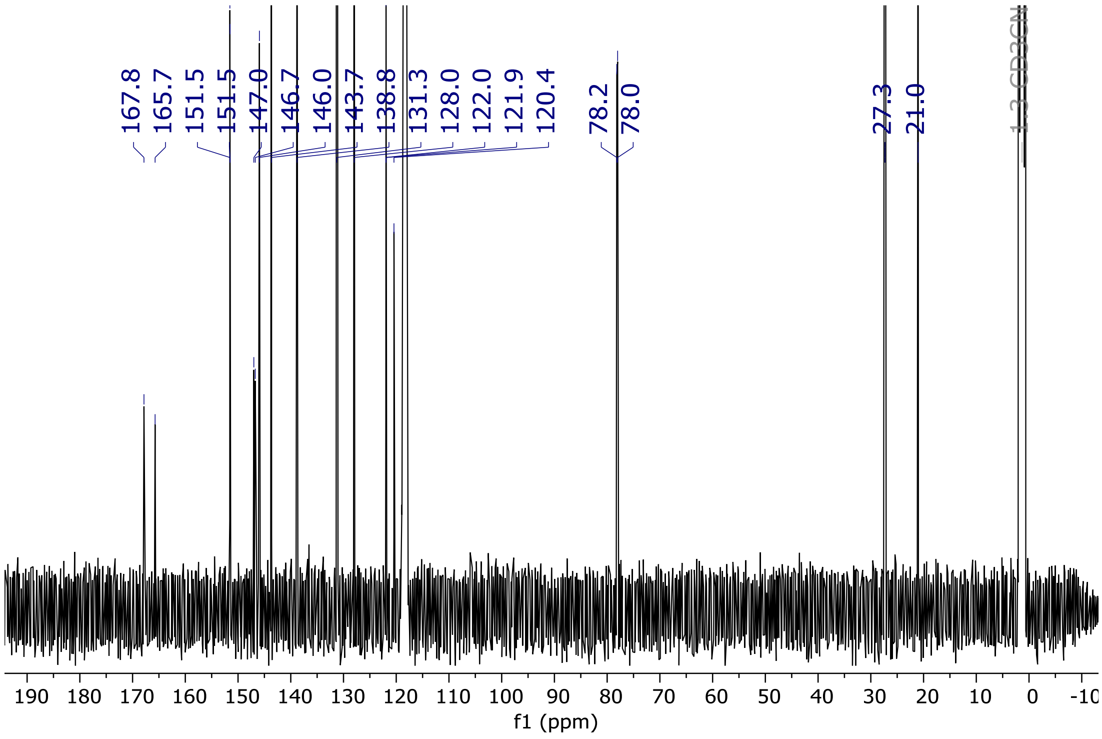

^{19}F NMR (376 MHz, $CDCl_3$):

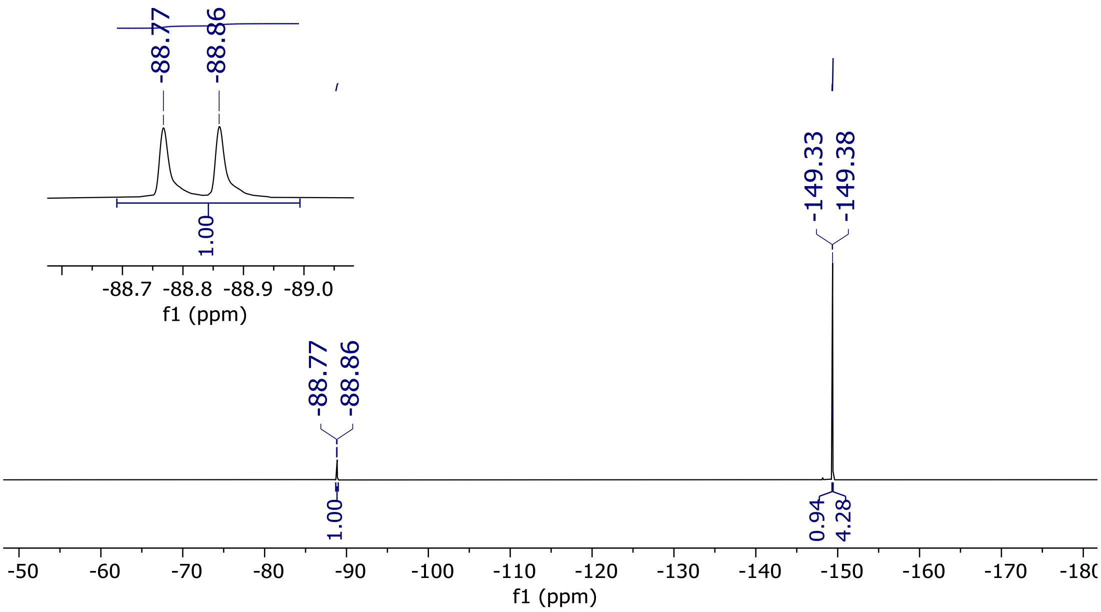

(Z)-3-(1-fluoro-2-iodovinyl)quinoline

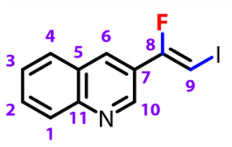

^{1}H NMR (500 MHz, $CDCl_3$):

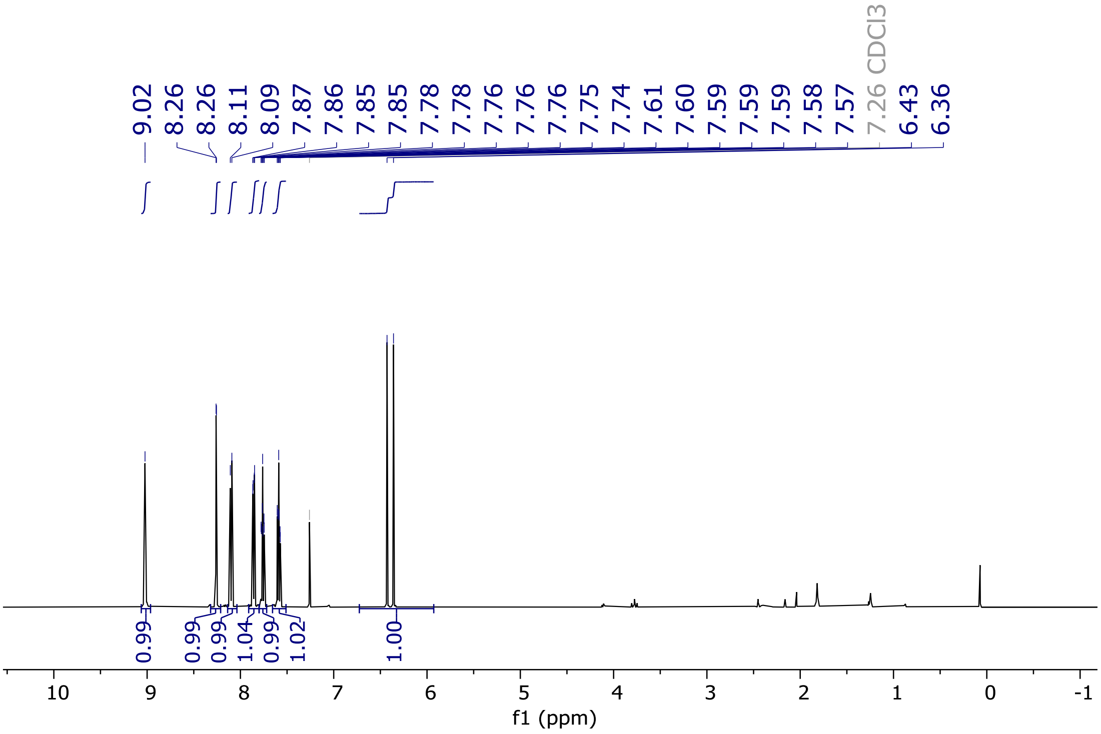

^{13}C NMR (126 MHz, $CDCl_3$):

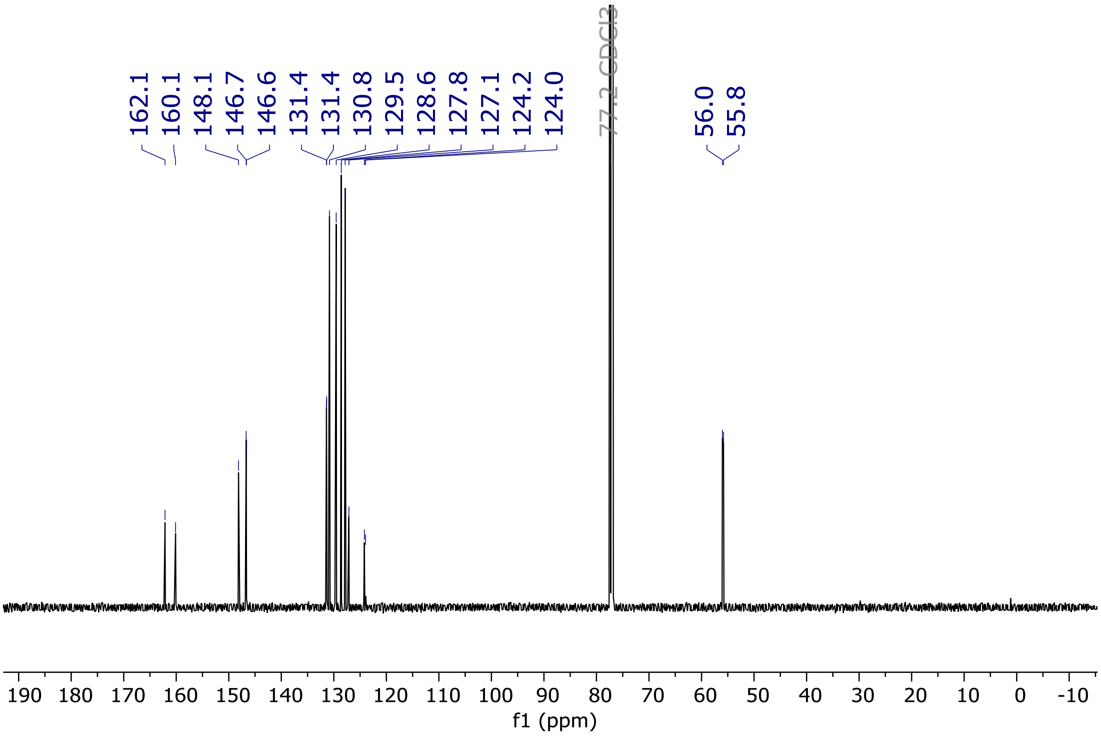

^{19}F (376 MHz, $CDCl_3$):

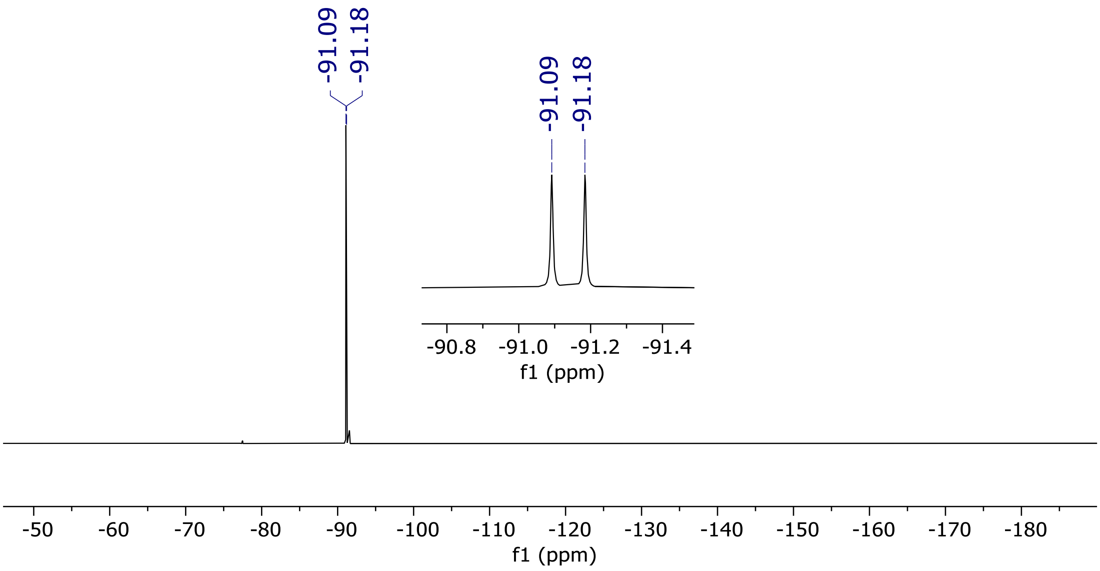

(Z)-(2-fluoro-2-(thiophen-3-yl)vinyl)(mesityl)iodonium BF_4 **(3ap)**

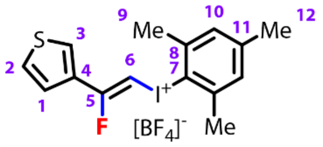

^{1}H NMR (500 MHz, $C(CD_3)_2O$):

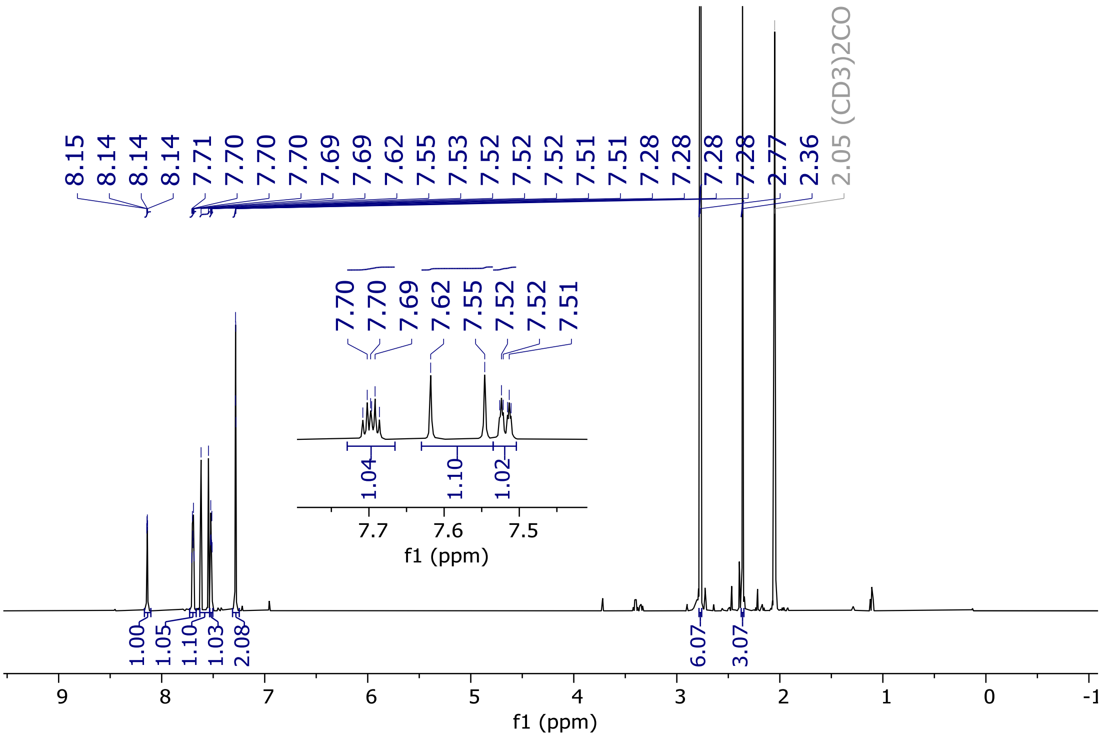

^{13}C NMR (126 MHz, $C(CD_3)_2O$):

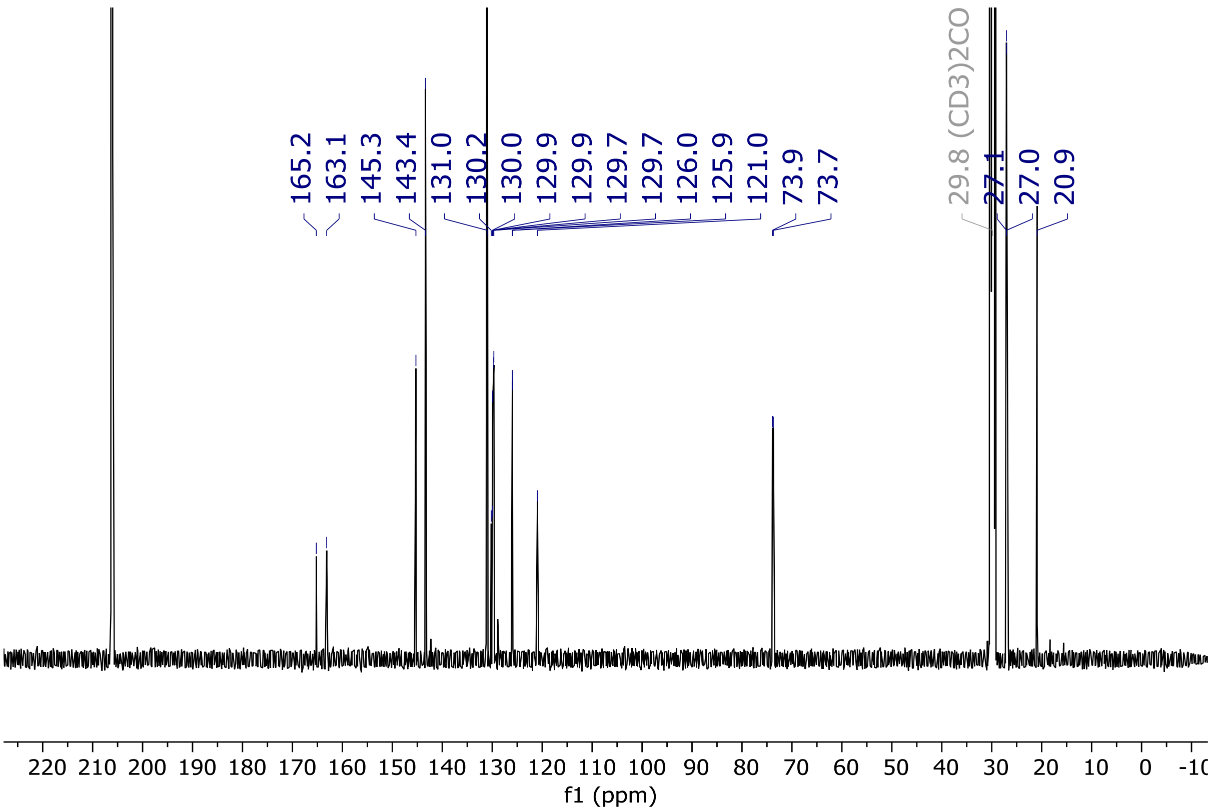

^{19}F NMR (376 MHz, $C(CD_3)_2O$):

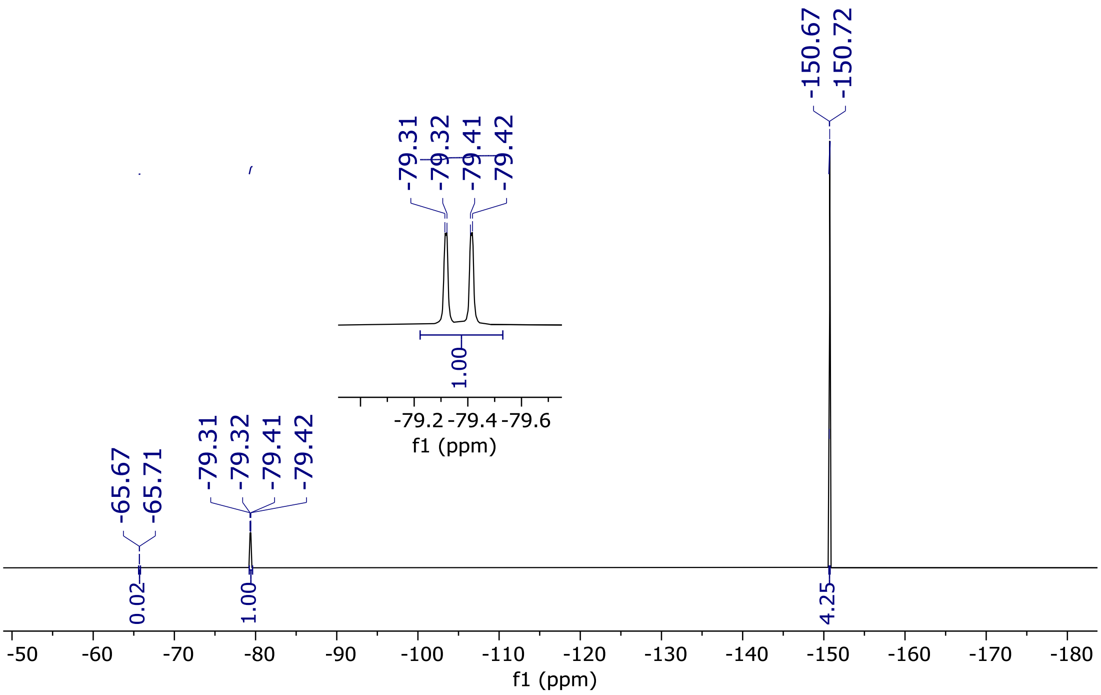

(Z)-(2-fluoro-3-oxobut-1-en-1-yl)(mesityl)iodonium BF_4 ***(3aq)***

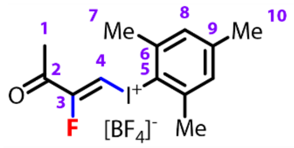

^{1}H NMR (500 MHz, CD_3CN):

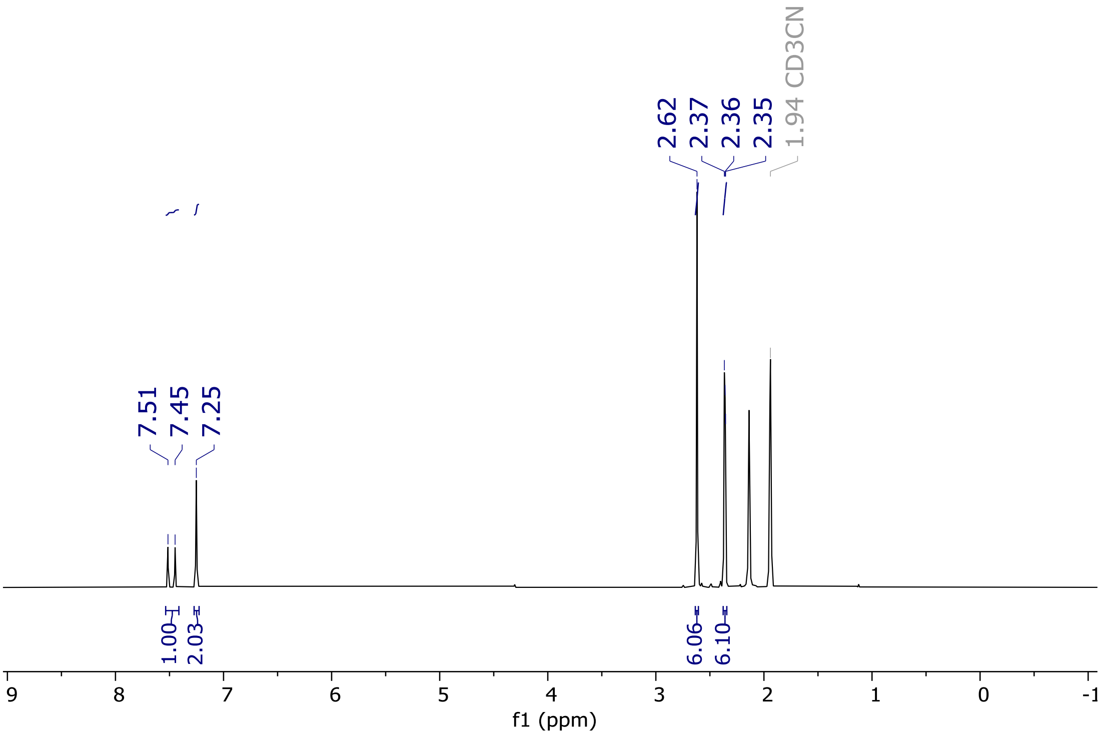

^{13}C NMR (126 MHz, CD_3CN):

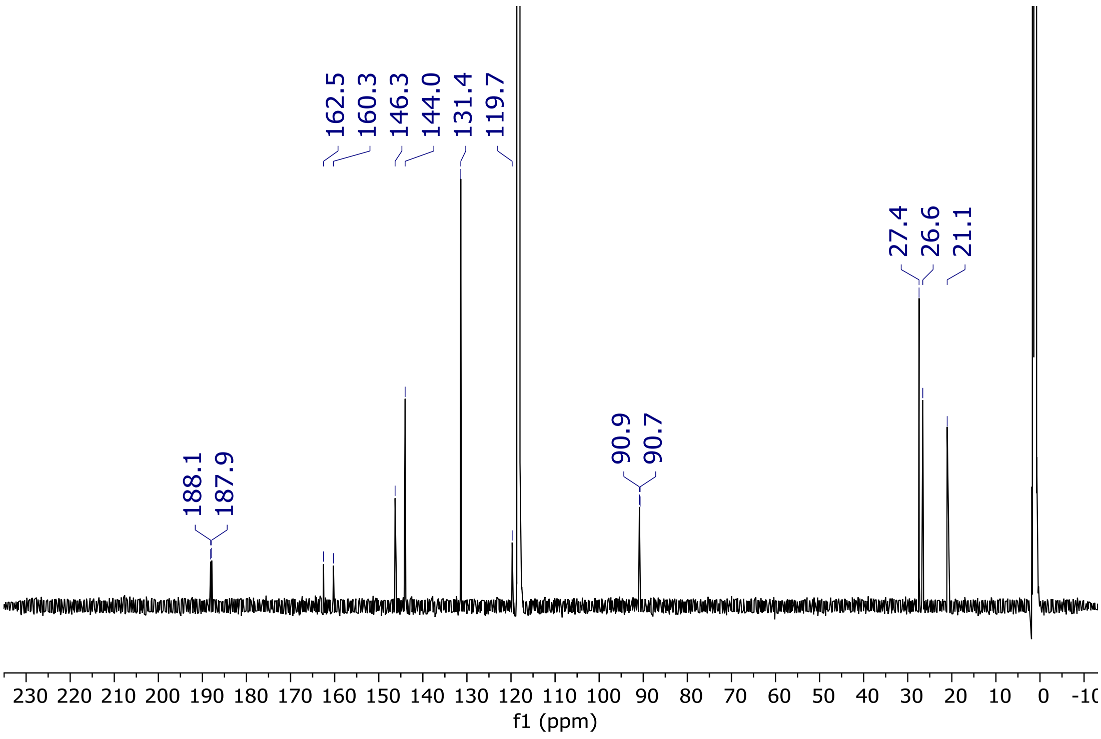

^{19}F NMR (376 MHz, CD_3CN):

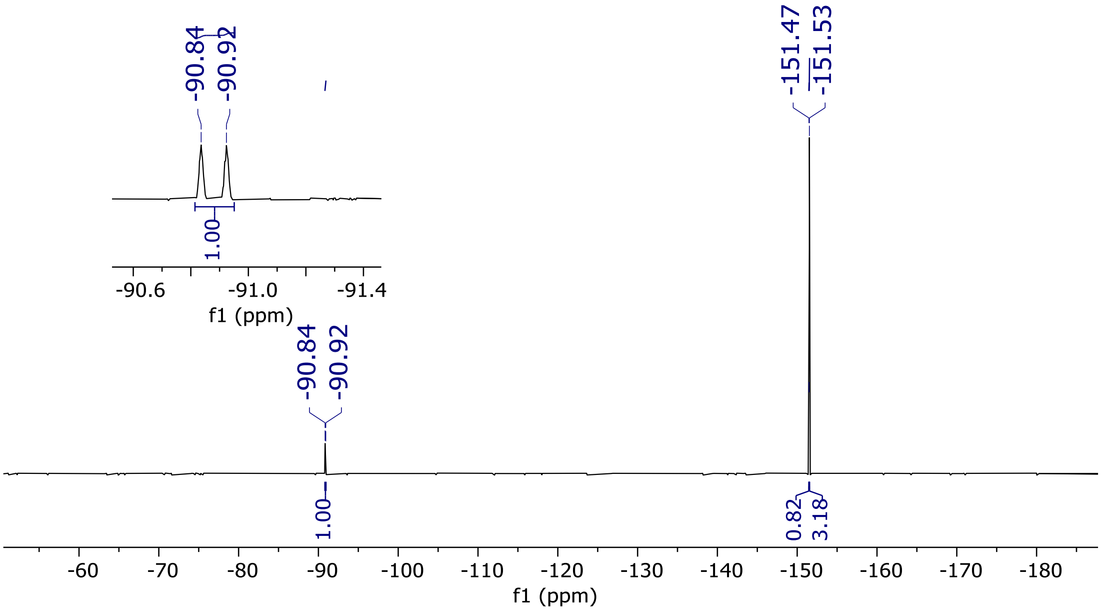

(Z)-(3-ethoxy-2-fluoro-3-oxoprop-1-en-1-yl)(mesityl)iodonium BF_4 **(3ar)**

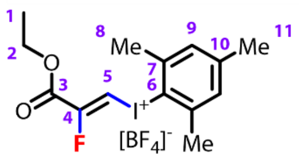

^{1}H NMR (500 MHz, CD_3CN):

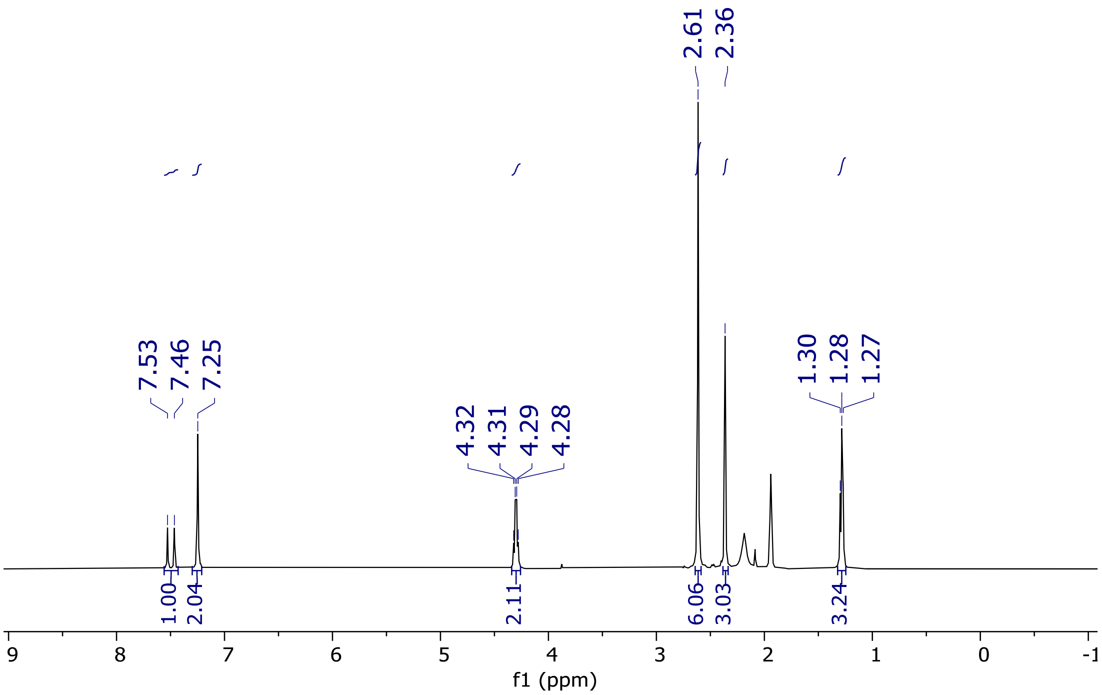

^{13}C NMR (126 MHz, CD_3CN):

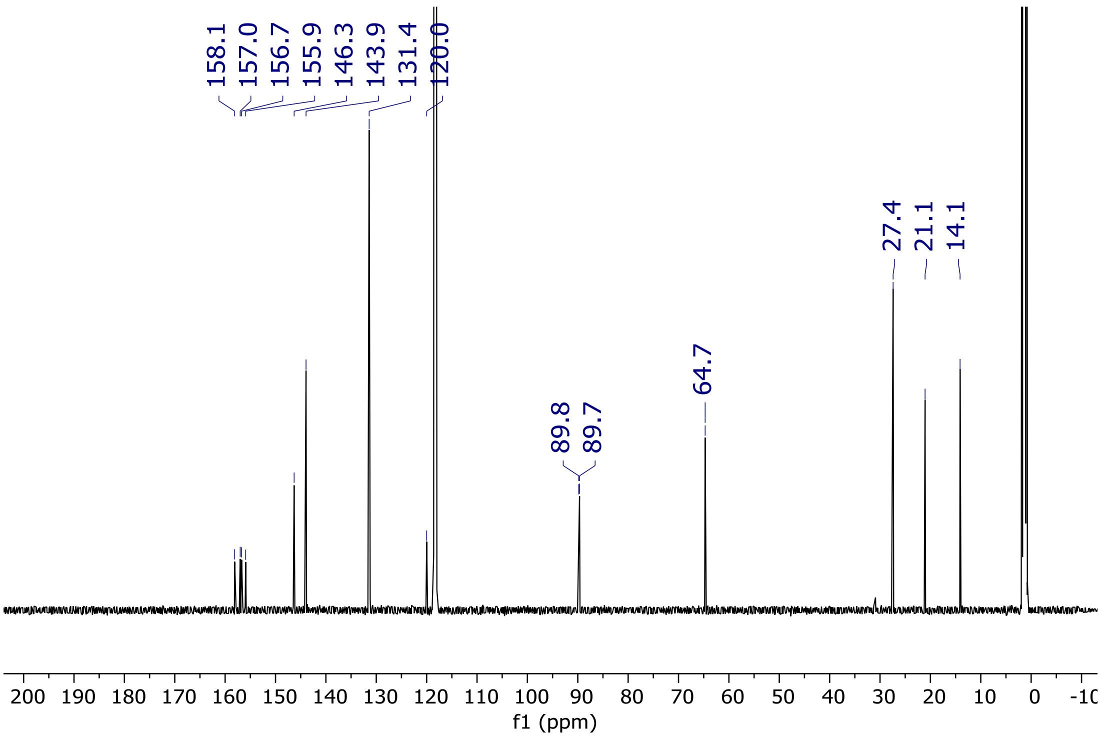

^{19}F NMR (376 MHz, CD_3CN):

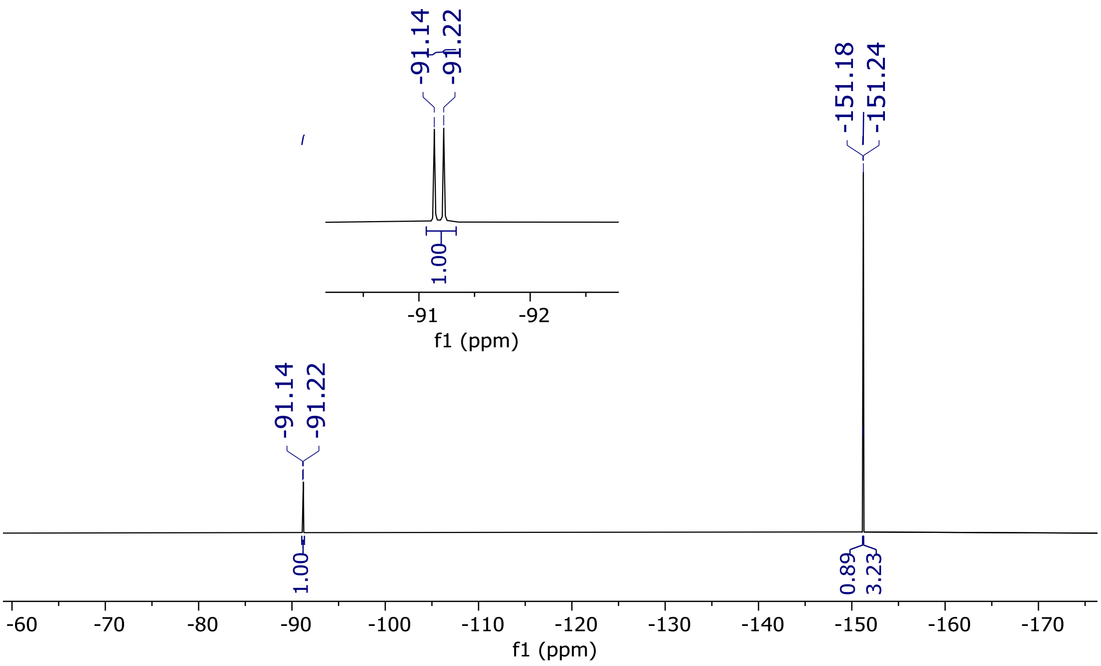

(Z)-(4-bromophenyl)(2-fluoro-5-phenylpent-1-en-1-yl)iodonium BF_4
(4a)

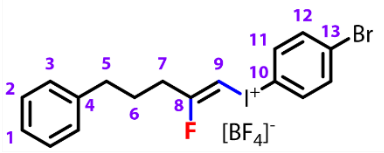

^{1}H NMR (400 MHz, $C(CD_3)_2O$):

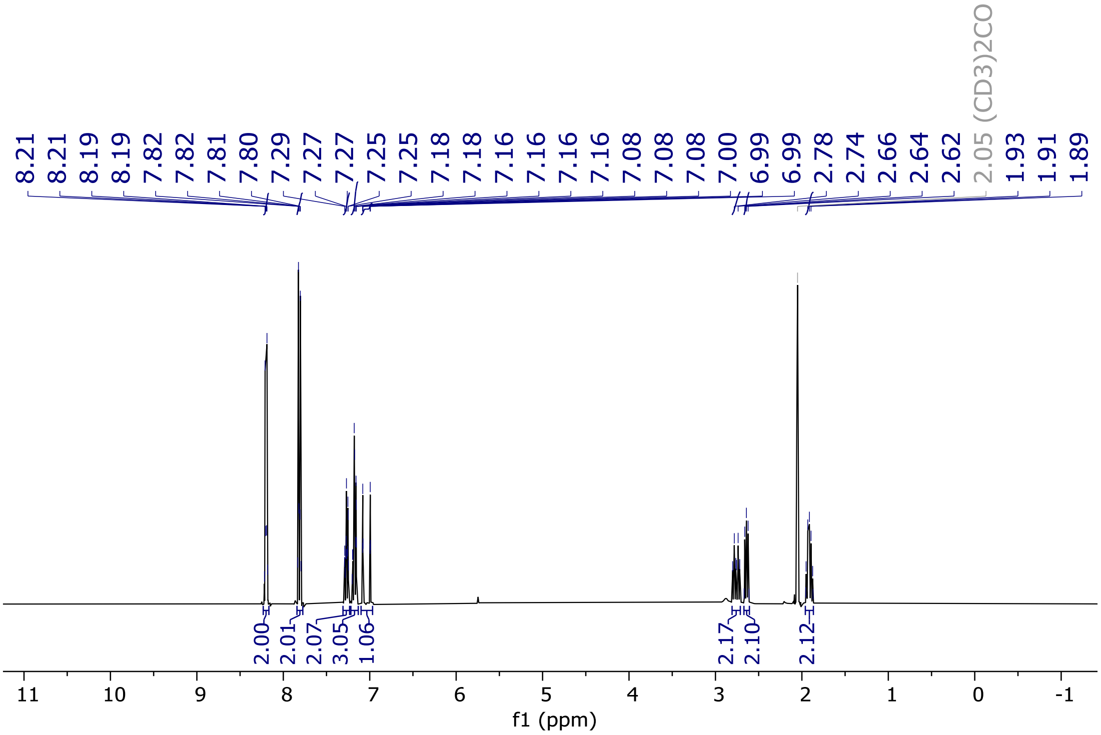

^{13}C NMR (126 MHz, $C(CD_3)_2O$):

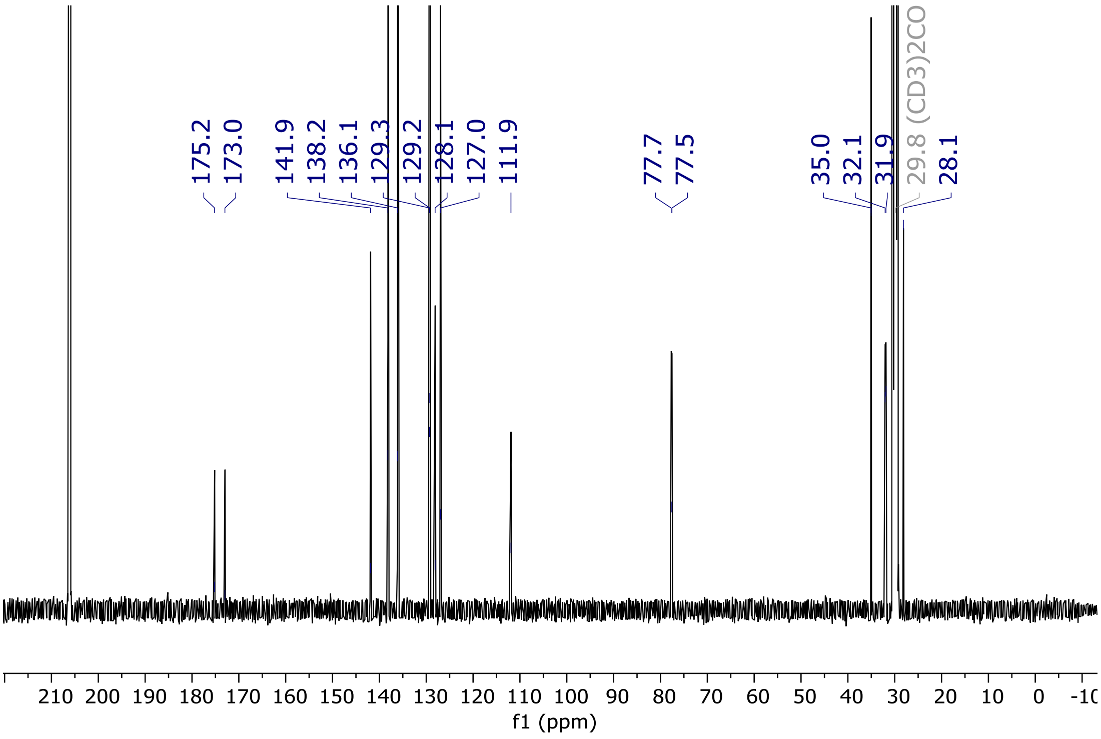

^{19}F NMR (376 MHz, $C(CD_3)_2O$):

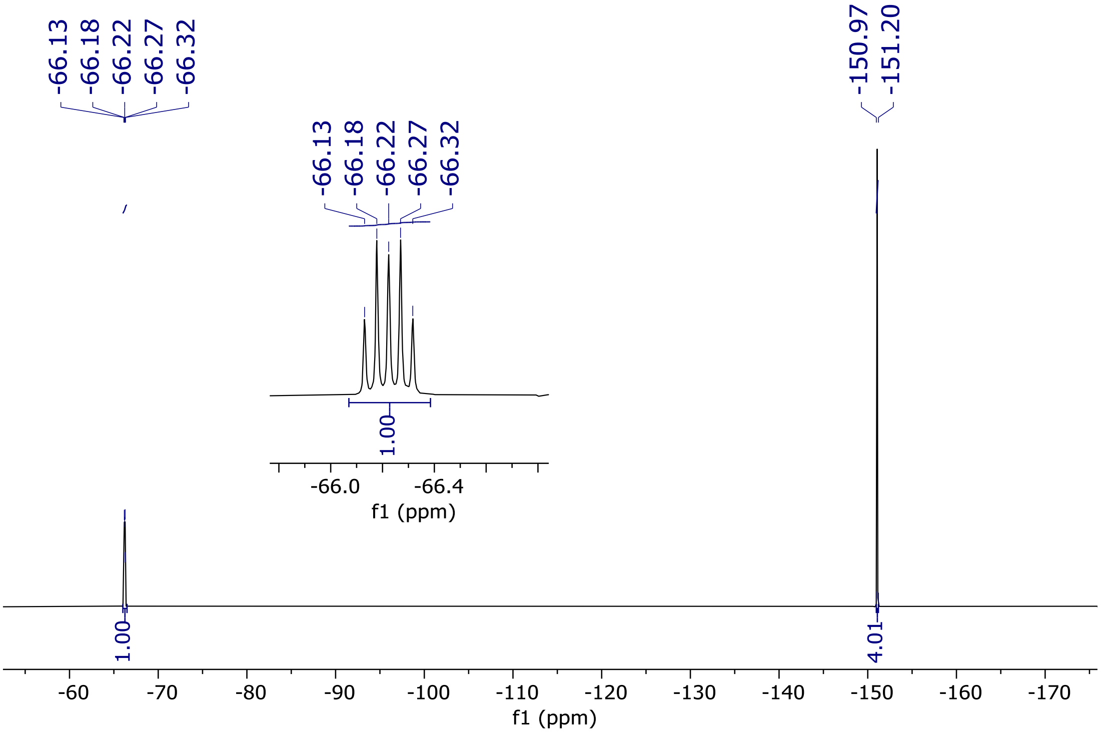

(Z)-[1,1’-biphenyl]-4-yl(2-fluoro-5-phenylpent-1-en-1-yl)iodonium BF_4 ***(4b)***

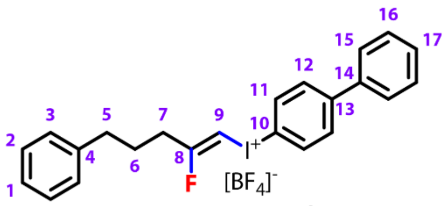

^{1}H NMR (400 MHz, $C(CD_3)_2O$):

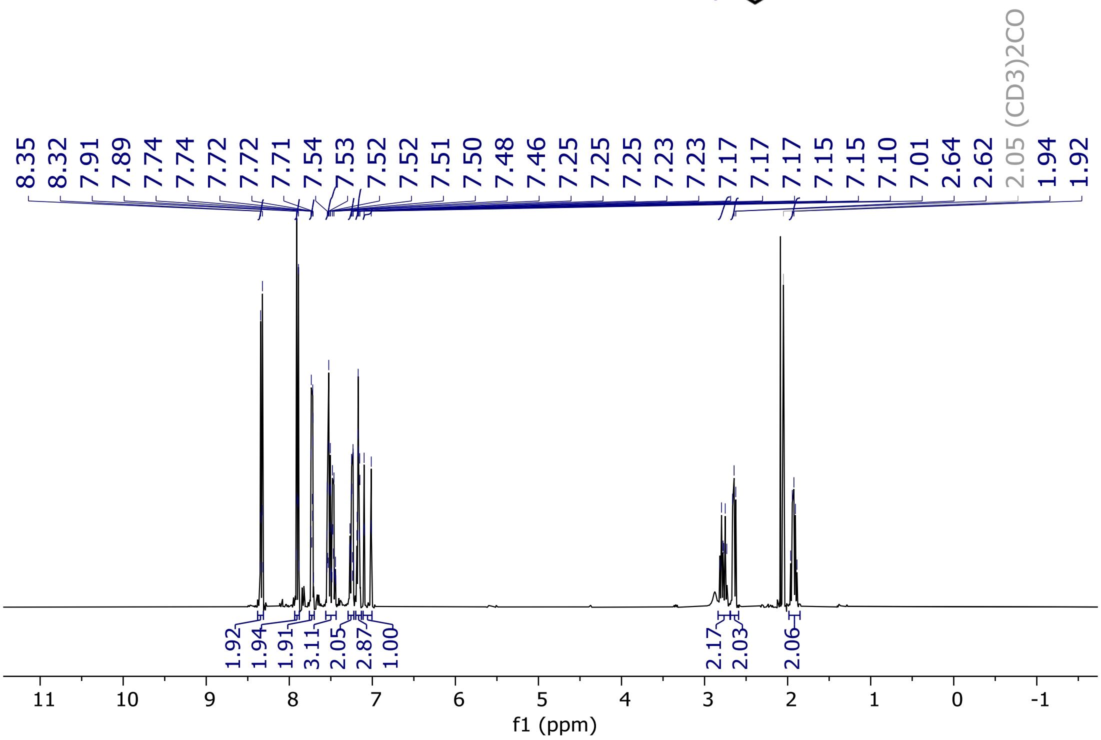

^{13}C NMR (126 MHz, $C(CD_3)_2O$):

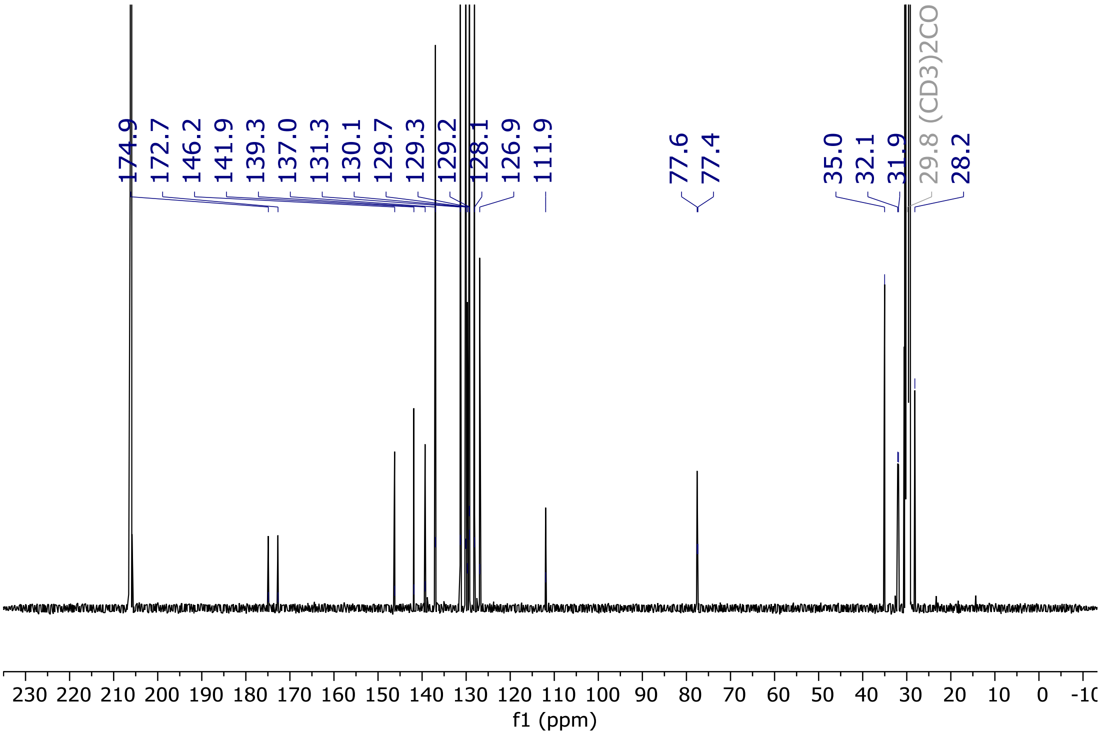

^{19}F NMR (376 MHz, $C(CD_3)_2O$):

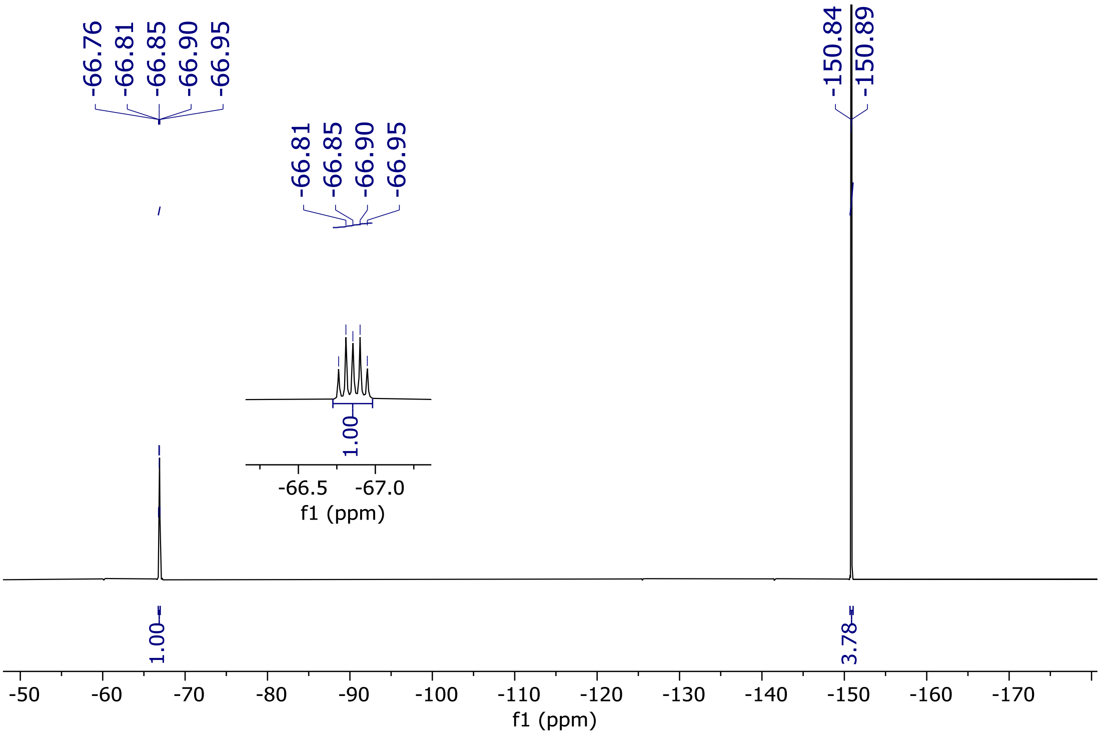

(Z)-(2-fluoro-5-phenylpent-1-en-1-yl)(p-tolyl)iodonium BF_4 ***(4c)***

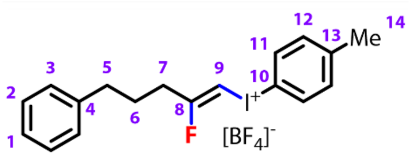

^{1}H NMR (400 MHz, $C(CD_3)_2O$):

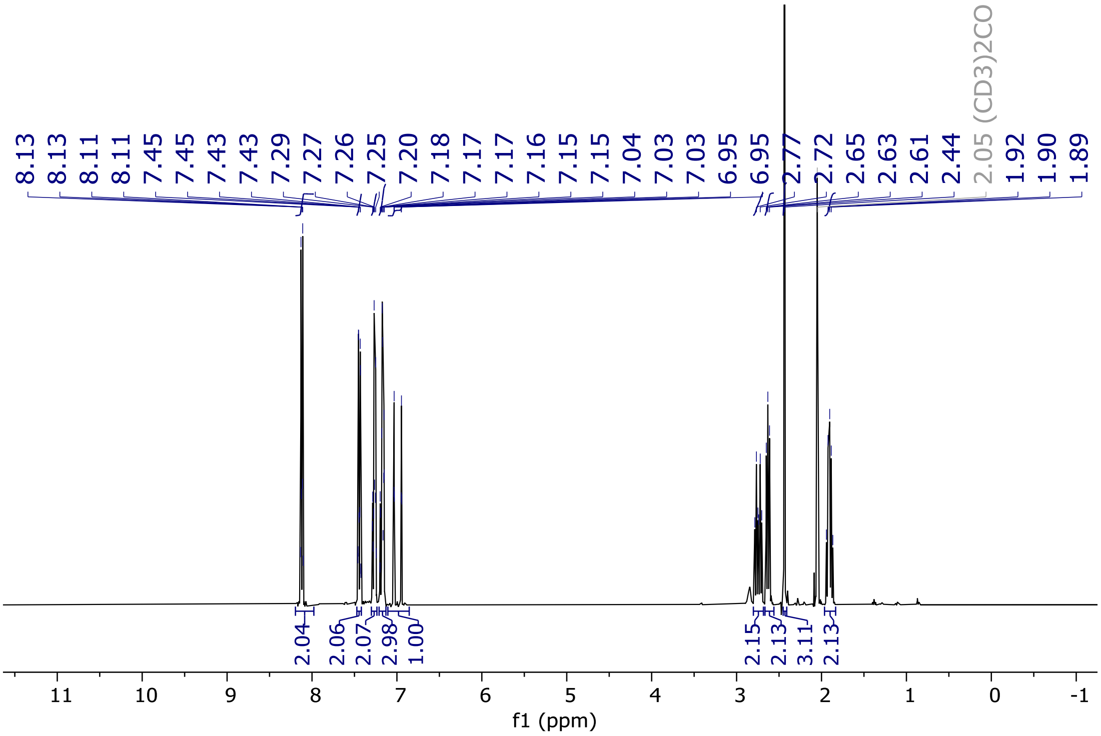

^{13}C NMR (126 MHz, CD_3CN):

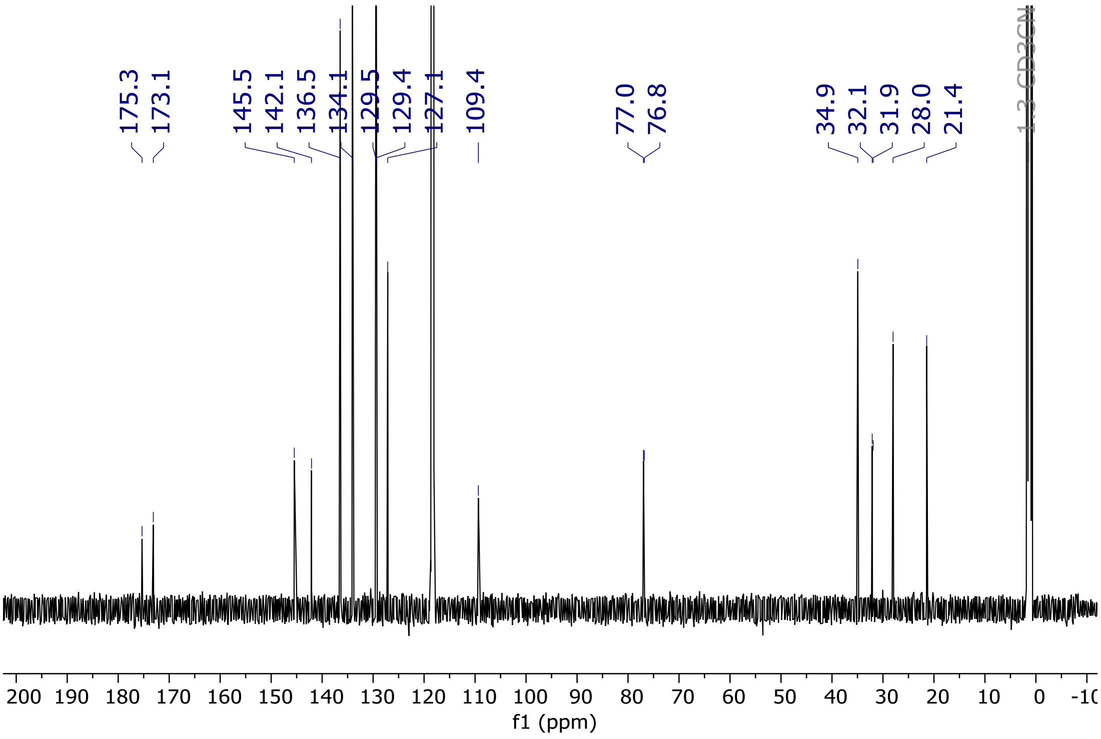

^{19}F NMR (377 MHz, CD_3CN):

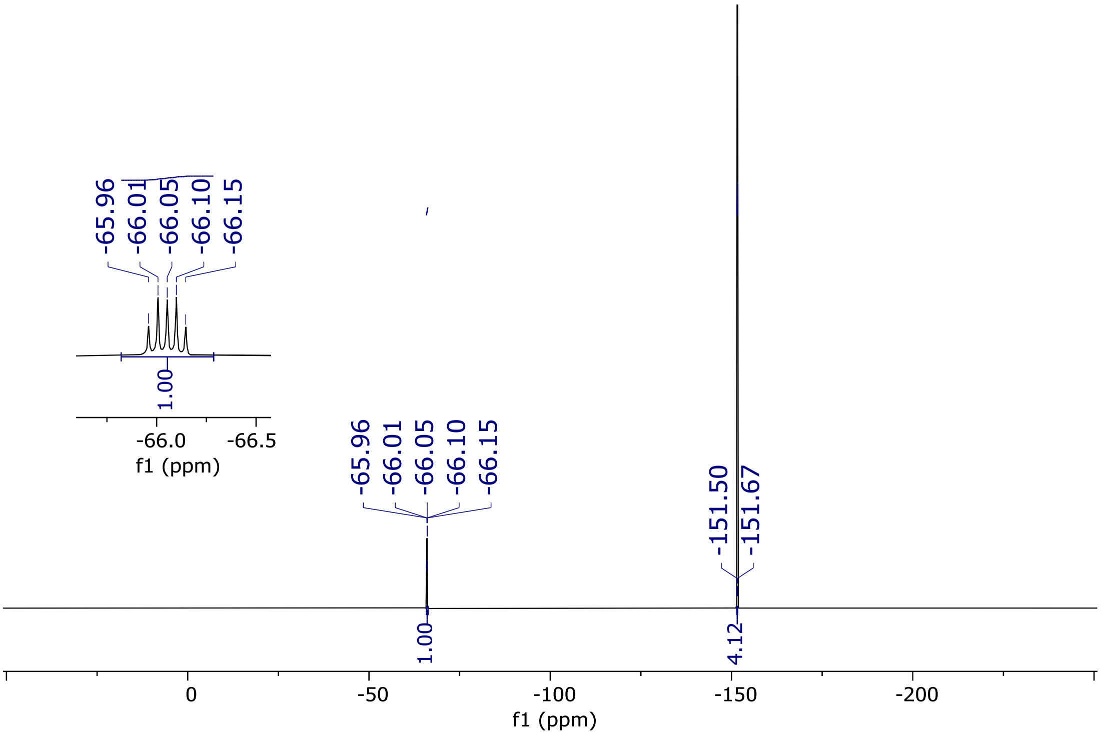

(Z)-(2-fluoro-5-phenylpent-1-en-1-yl)(4-methoxyphenyl)iodonium BF_4 ***(4d)***

^{1}H NMR (500 MHz, $C(CD_3)_2O$):

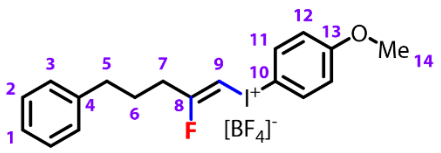

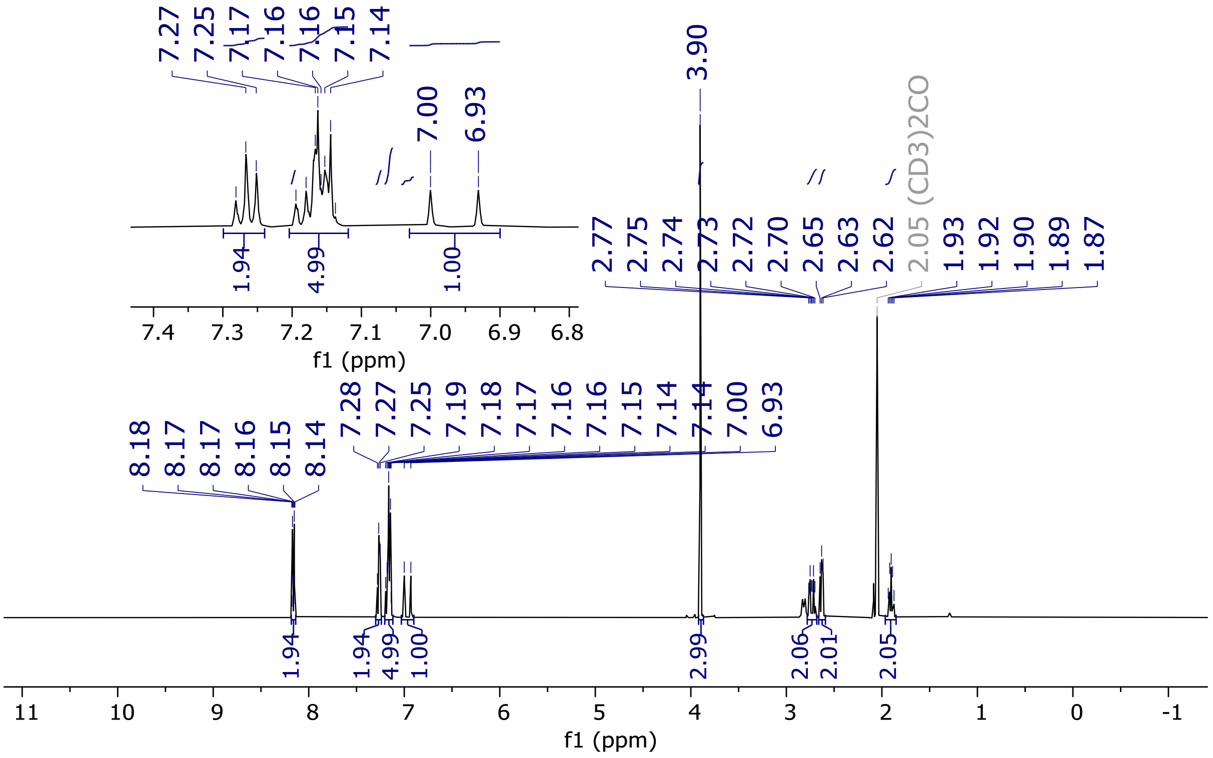

^{13}C NMR (126 MHz, $C(CD_3)_2O$):

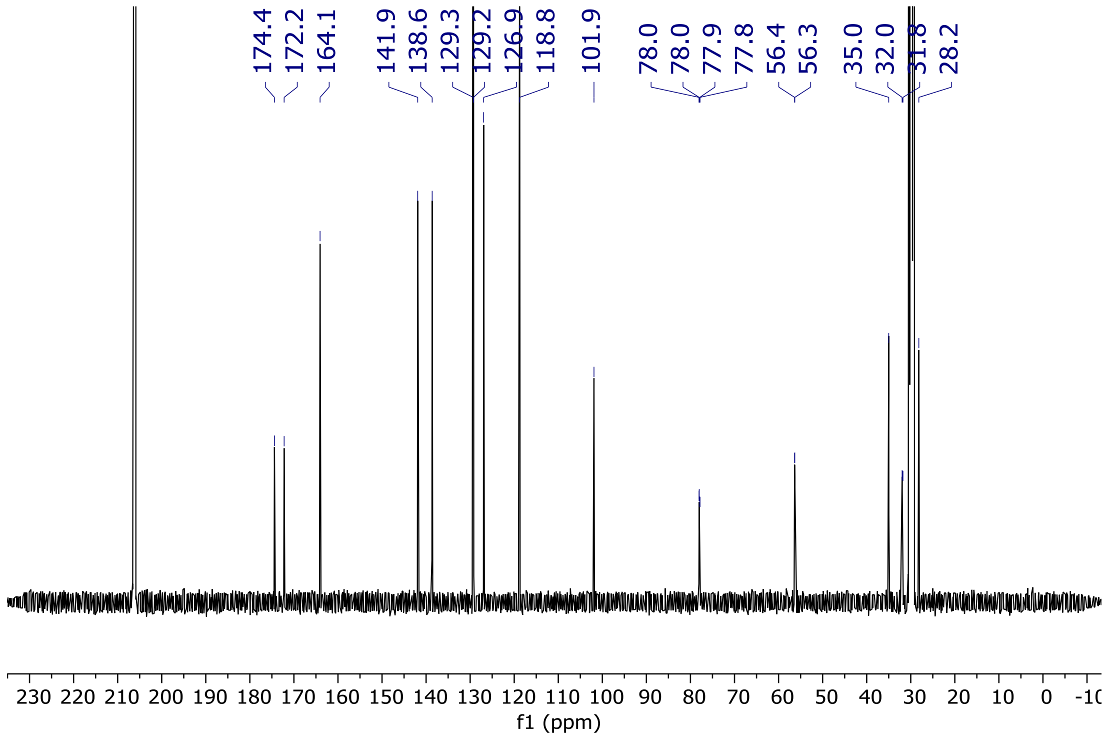

^{19}F NMR (376 MHz, $C(CD_3)_2O$):

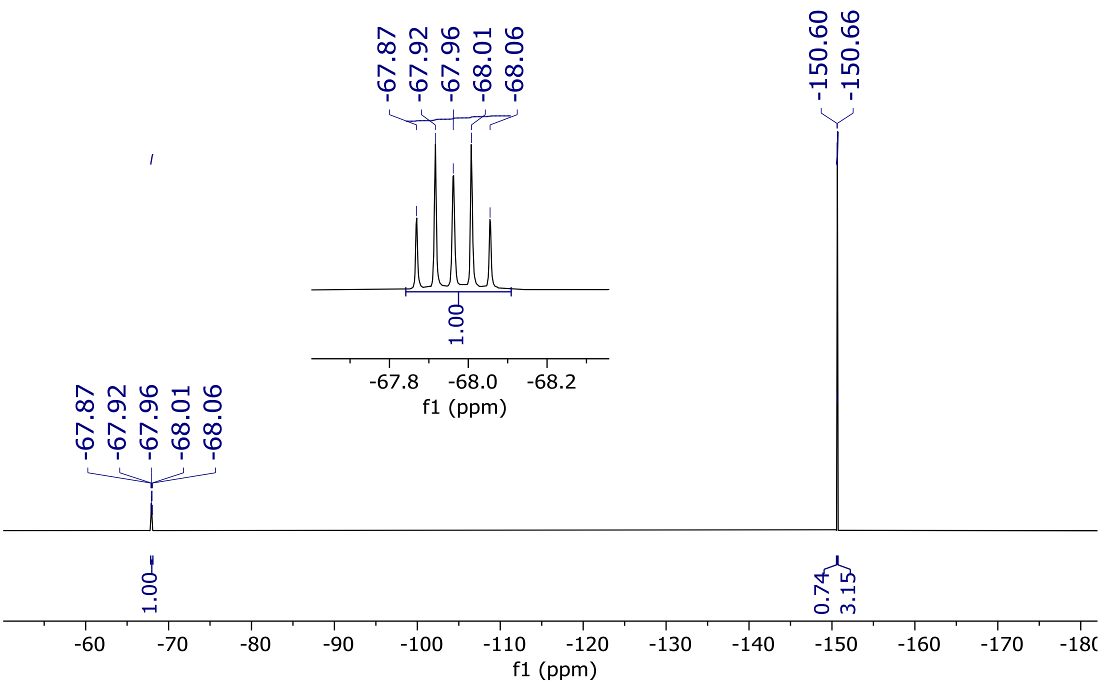

(Z)-(2-fluoro-5-phenylpent-1-en-1-yl)(o-tolyl)iodonium BF_4 ***(4e)***

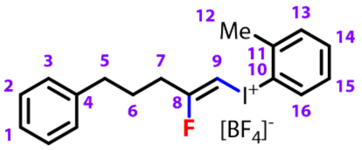

^{1}H NMR (500 MHz, $CDCl_3$):

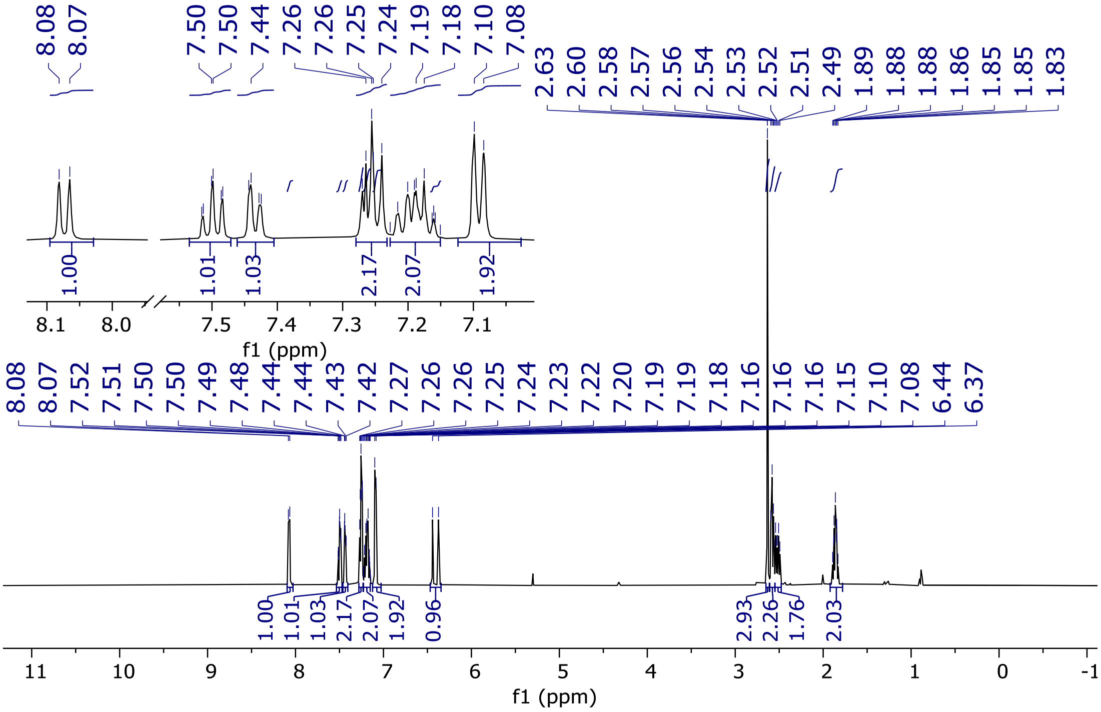

^{13}C NMR (126 MHz, $CDCl_3$):

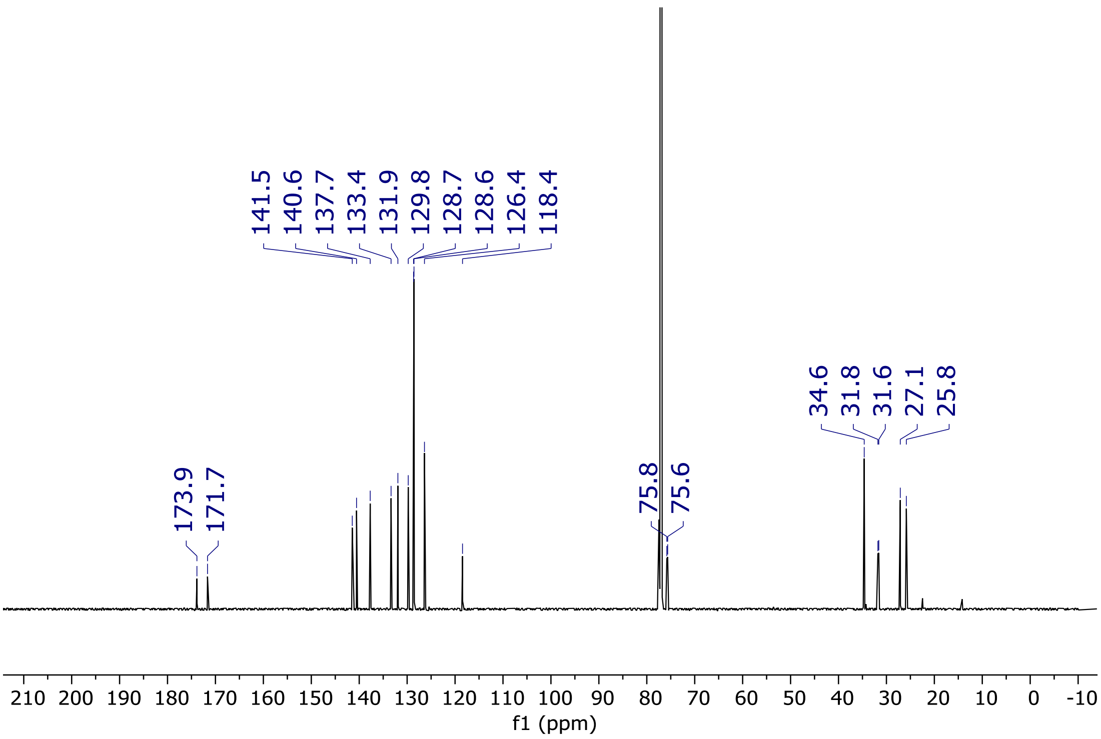

^{19}F NMR (376 MHz, $C(CD_3)_2O$):

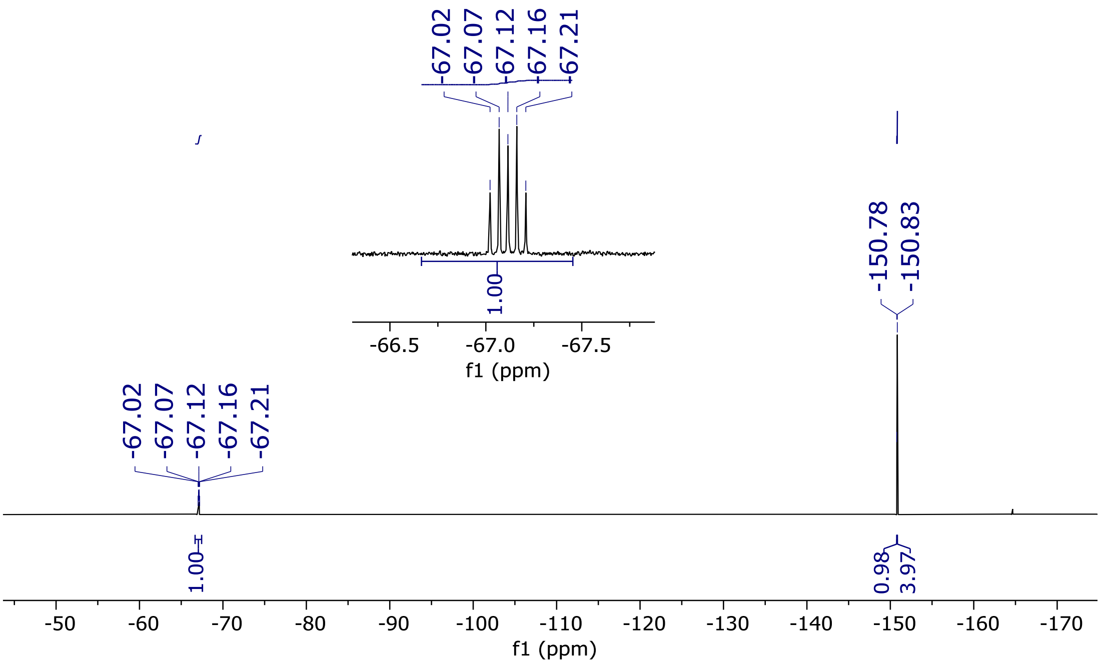

(Z)-(2-fluoro-5-phenylpent-1-en-1-yl)(2,4,6-triisopropylphenyl)iodonium BF_4 ***(4f)***

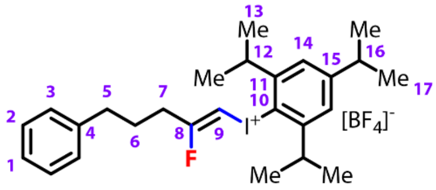

^{1}H NMR (500 MHz, $C(CD_3)_2O$):

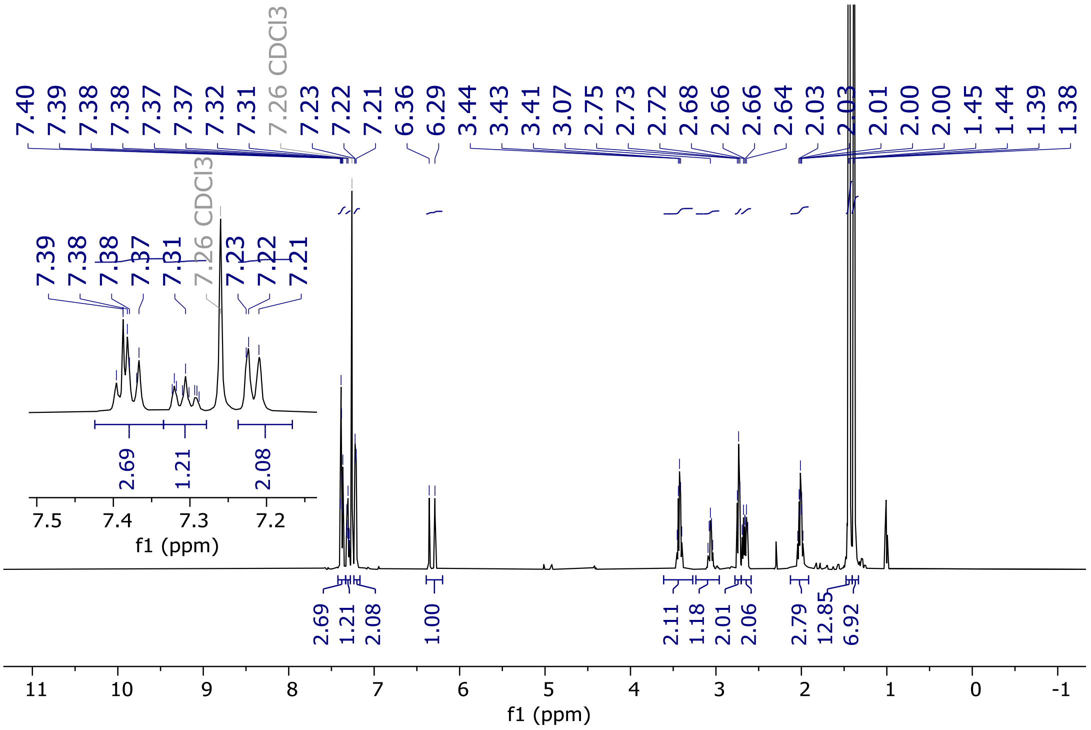

^{13}C NMR (126 MHz, $C(CD_3)_2O$):

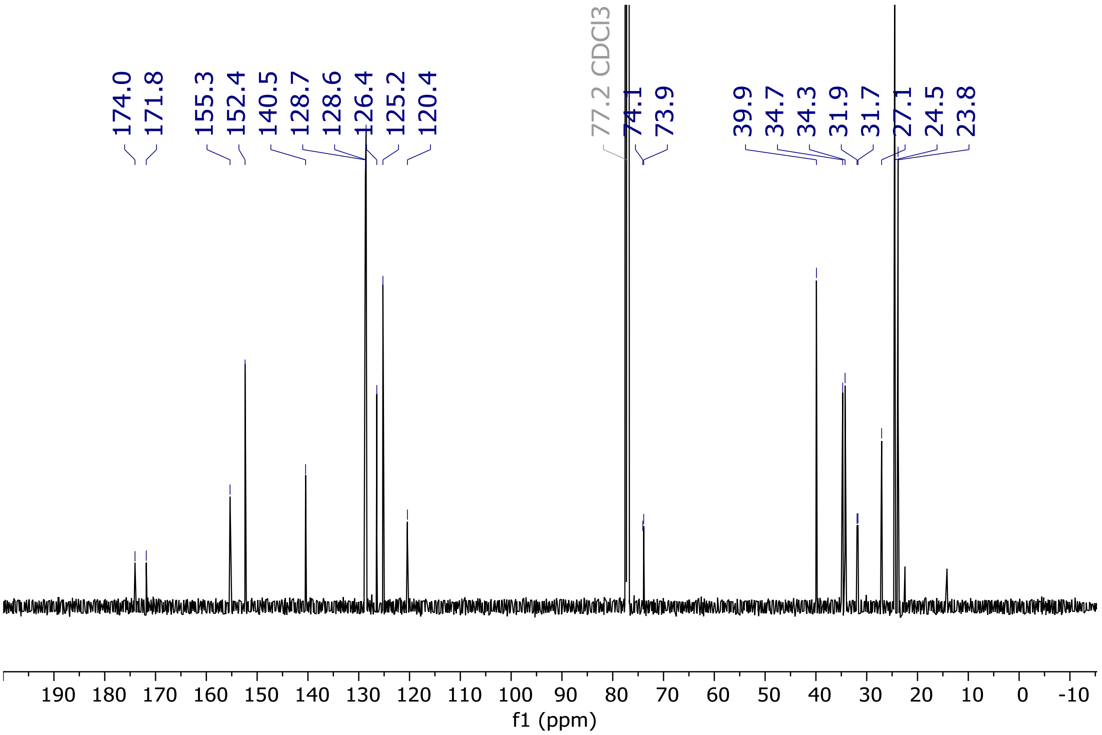

^{19}F NMR (376 MHz, $C(CD_3)_2O$):

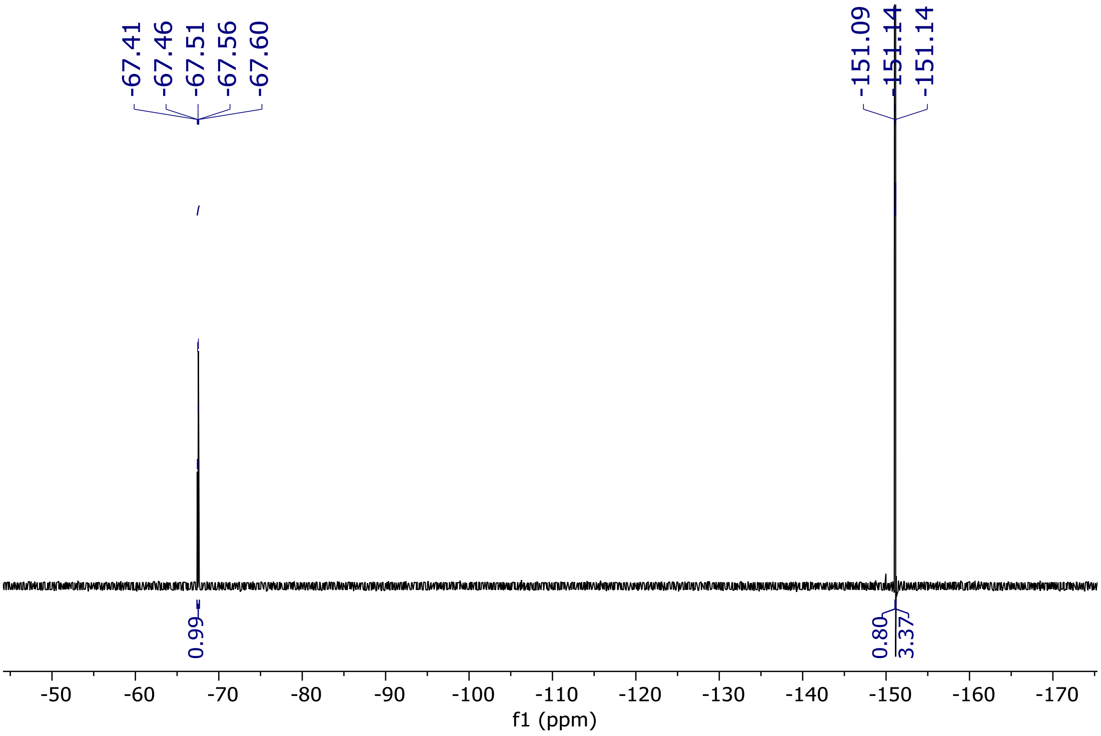

(Z)-(2-fluoro-3-(4-nitrophenoxy)prop-1-en-1-yl)(p-tolyl)iodonium BF_4 **(4g)**

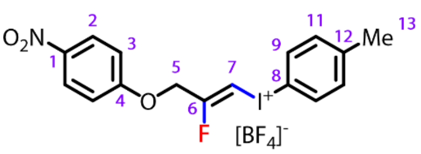

^{1}H NMR (400 MHz, $C(CD_3)_2O$):

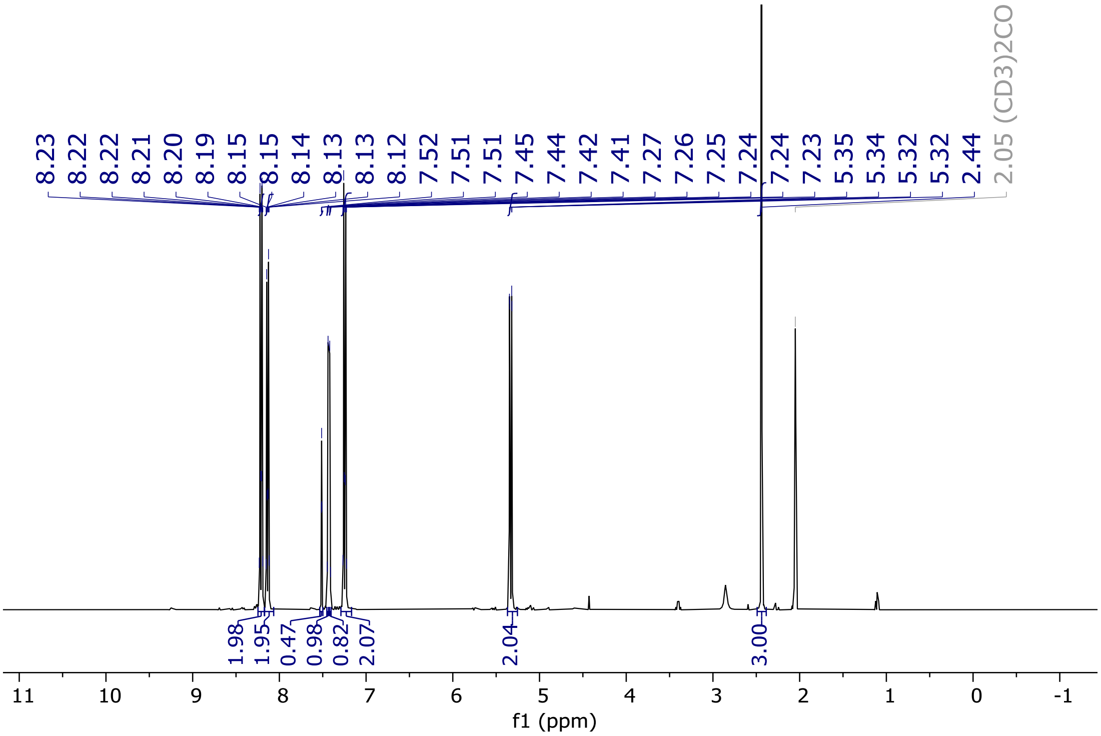

^{13}C NMR (126 MHz, $C(CD_3)_2O$):

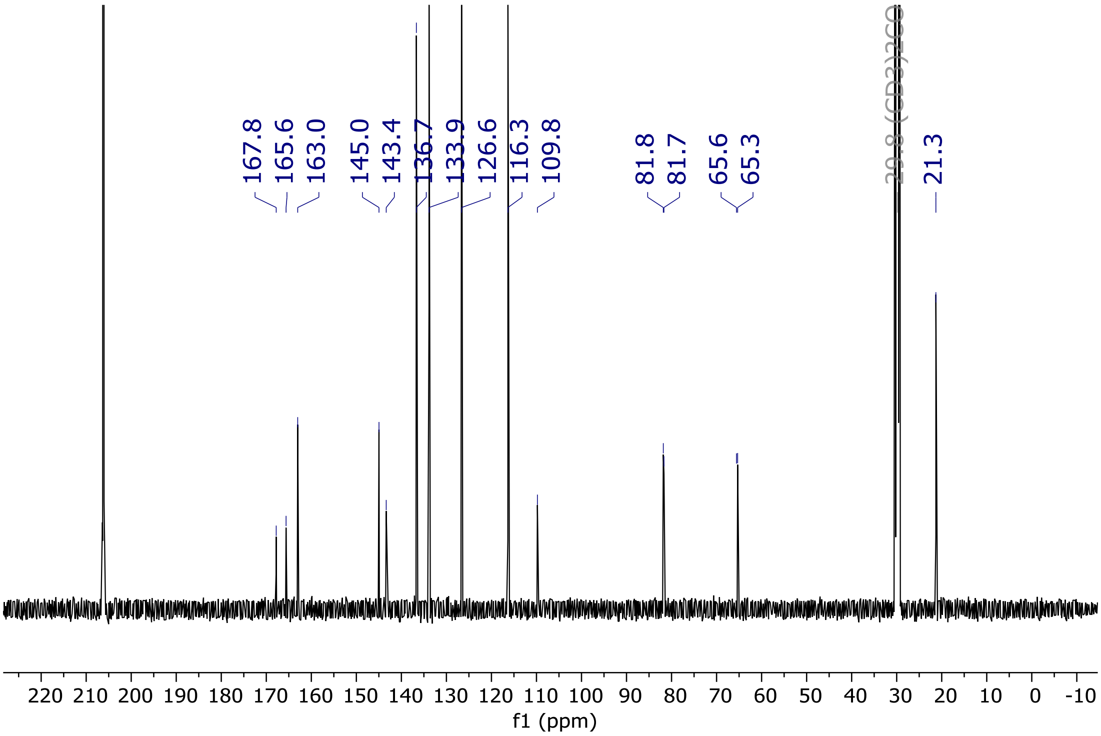

^{19}F NMR (377 MHz, $C(CD_3)_2O$):

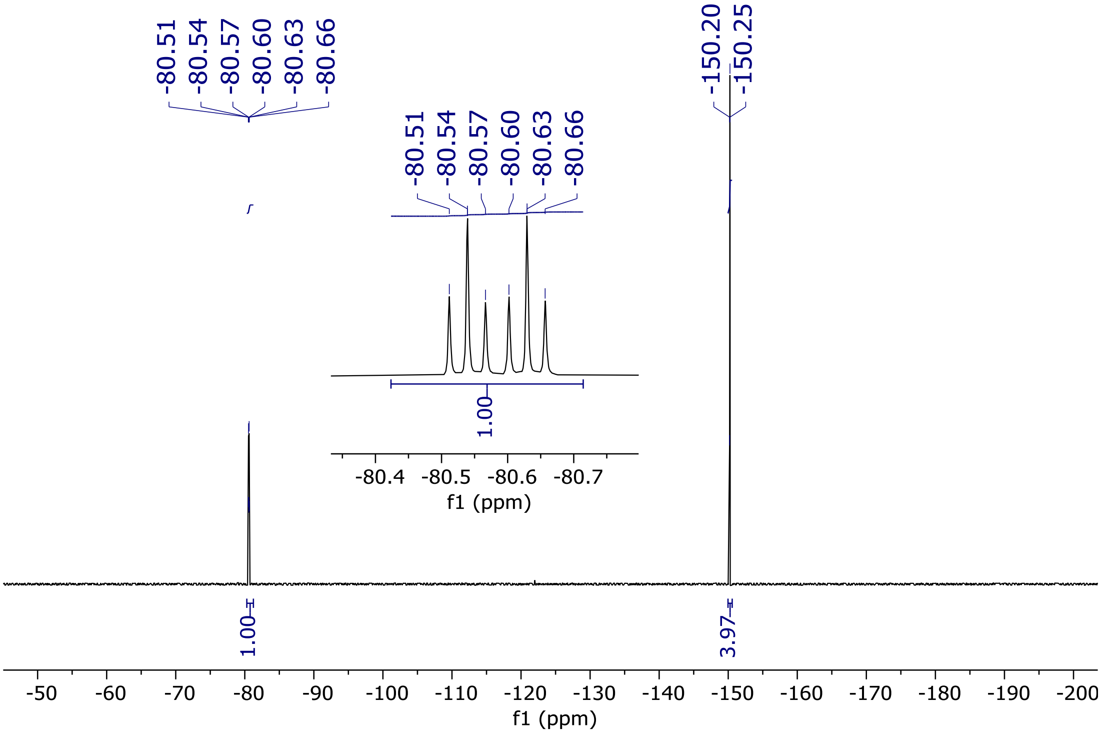

(Z)-(3-((N-cyclopropyl-4-methylphenyl)sulfonamido)-2-fluoroprop-1-en-1-yl)(p-tolyl)iodonium BF_4 ***(4h)***

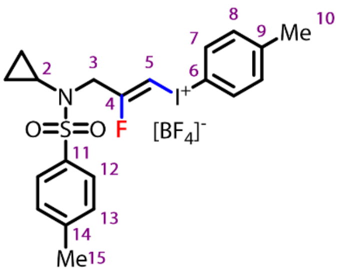

^{1}H NMR (500 MHz, $C(CD_3)_2O$):

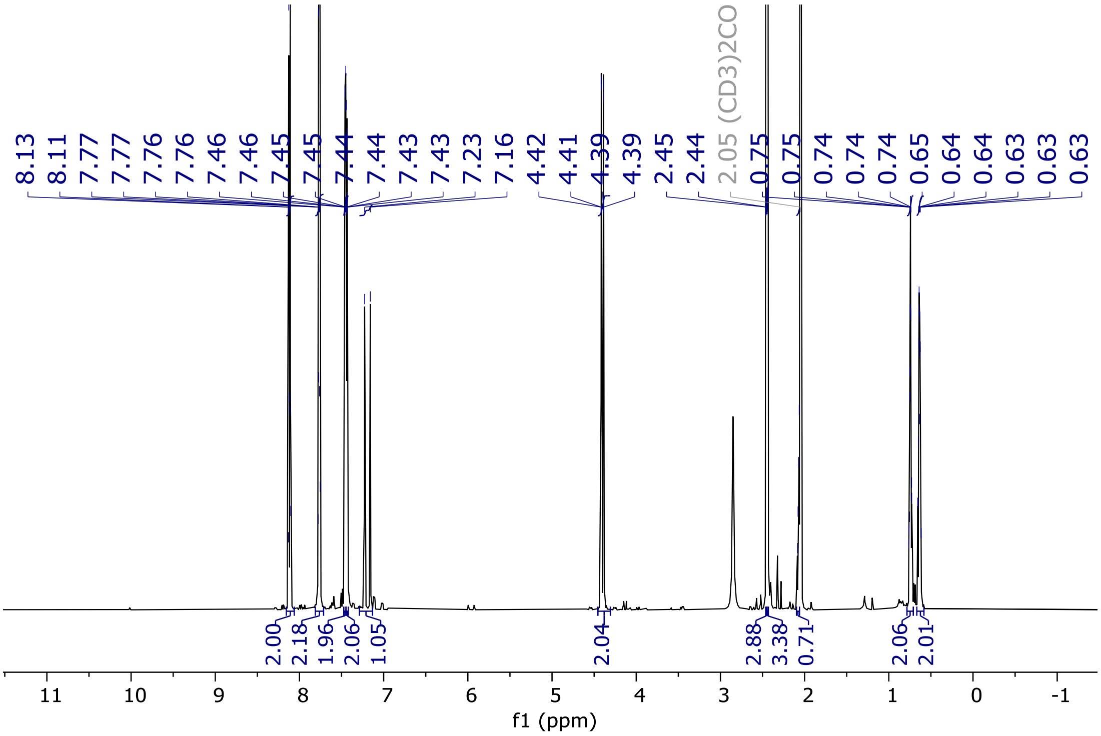

^{13}C NMR (126 MHz, $C(CD_3)_2O$):

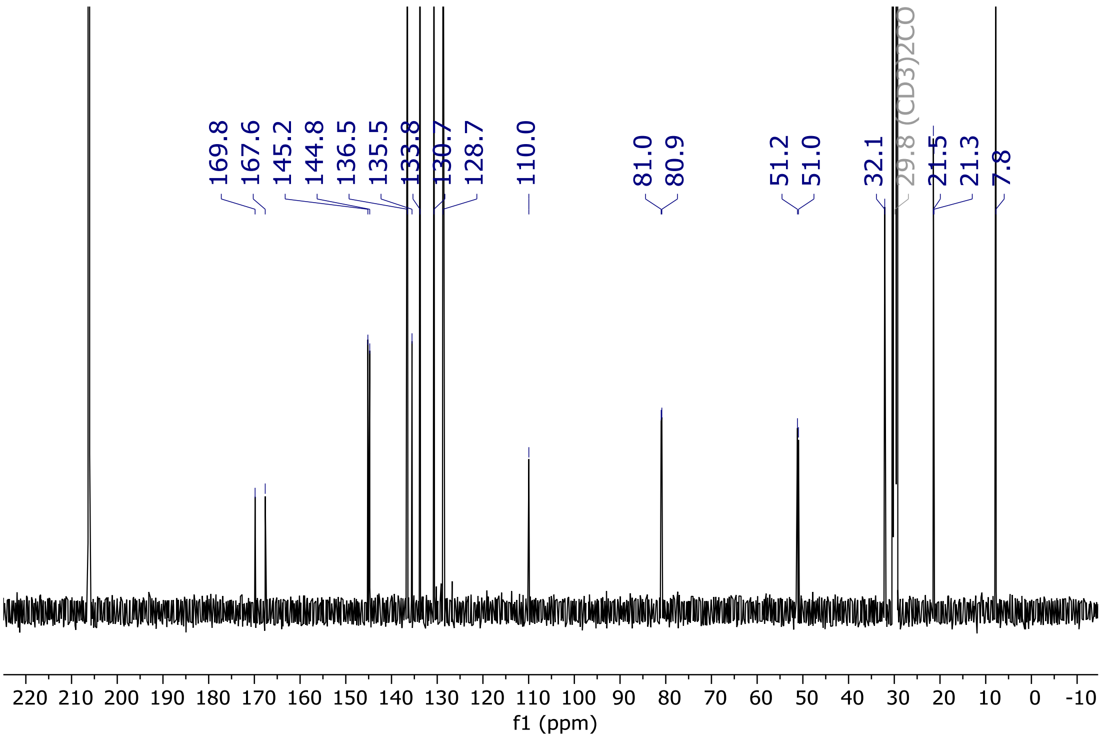

^{19}F NMR (471 MHz, $C(CD_3)_2O$):

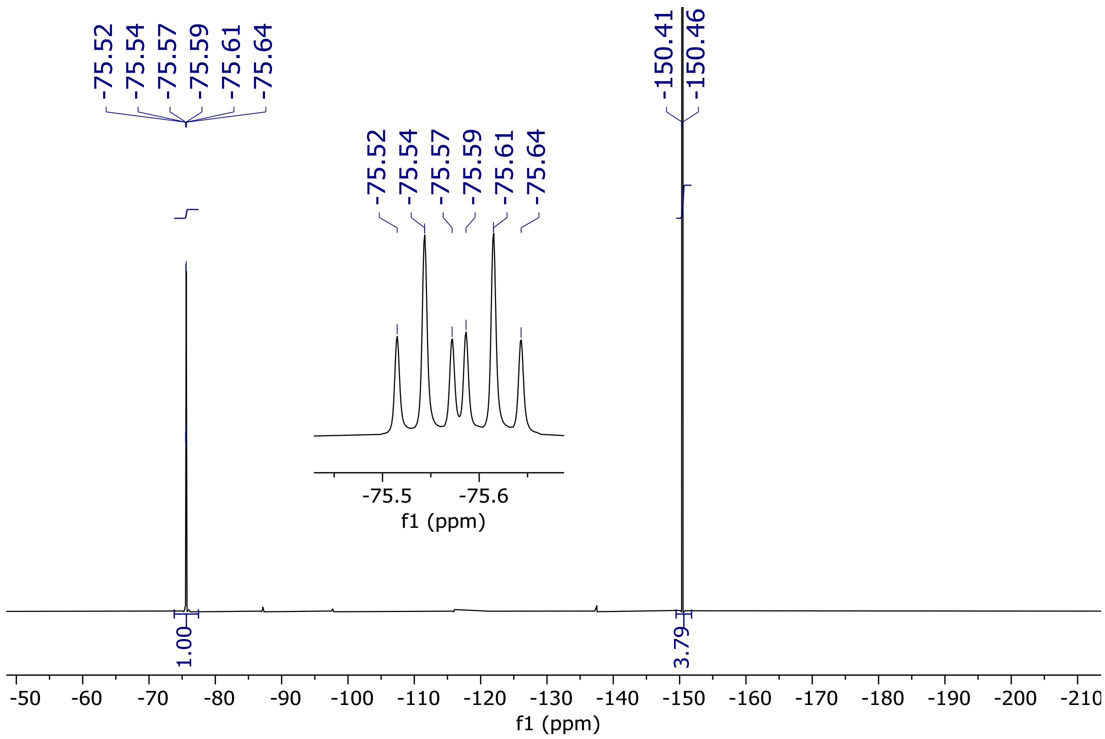

(Z)-(2-fluoro-2-phenylvinyl)(4-fluorophenyl)iodonium BF_4 ***(4i)***

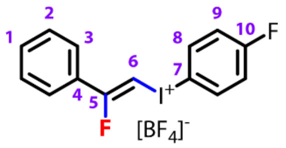

^{1}H NMR (400 MHz, $C(CD_3)_2O$):

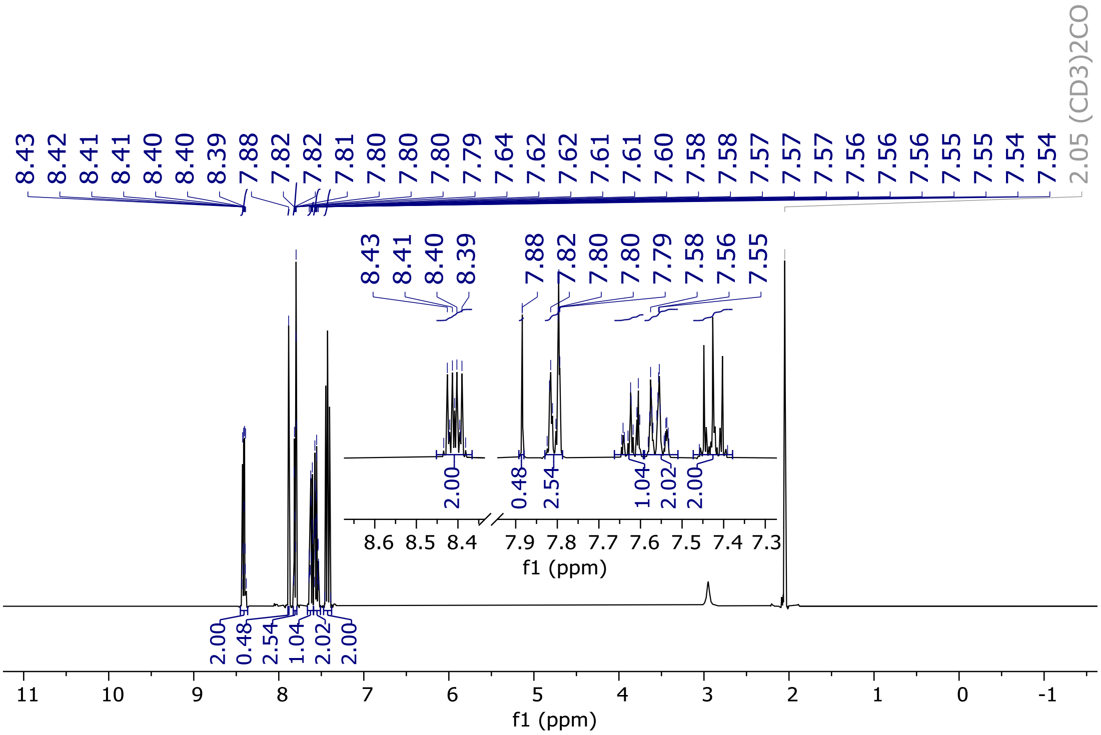

^{13}C NMR (126 MHz, CD_3CN):

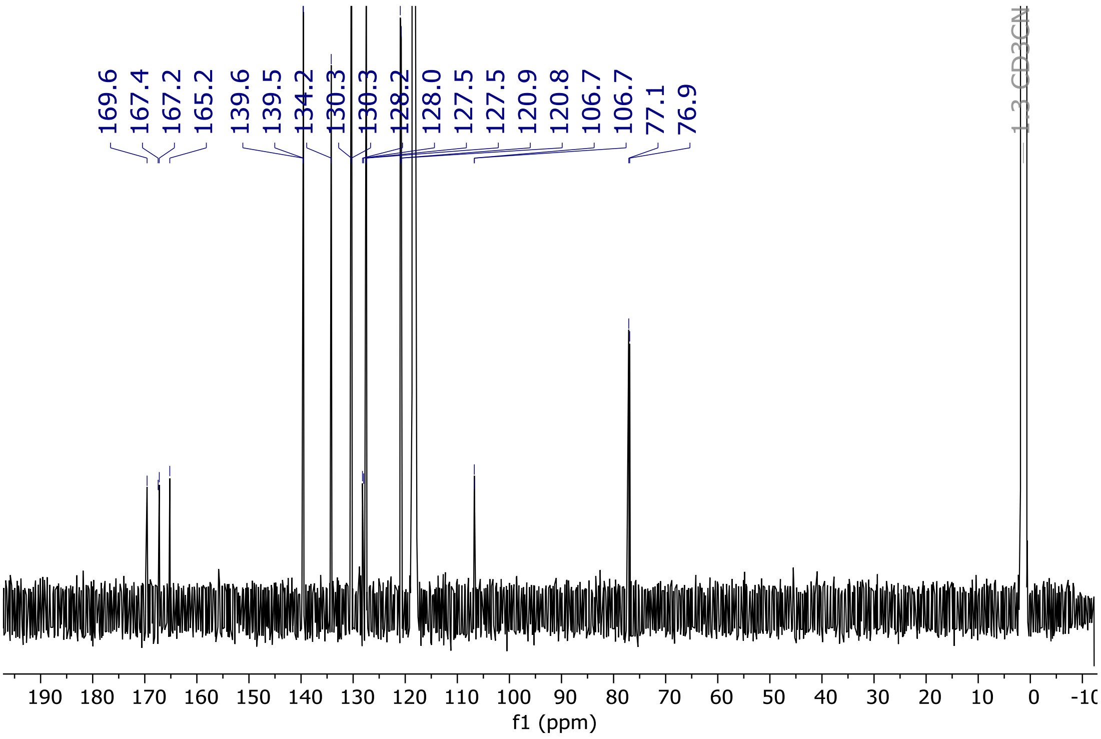

^{19}F NMR (376 MHz, $C(CD_3)_2O$):

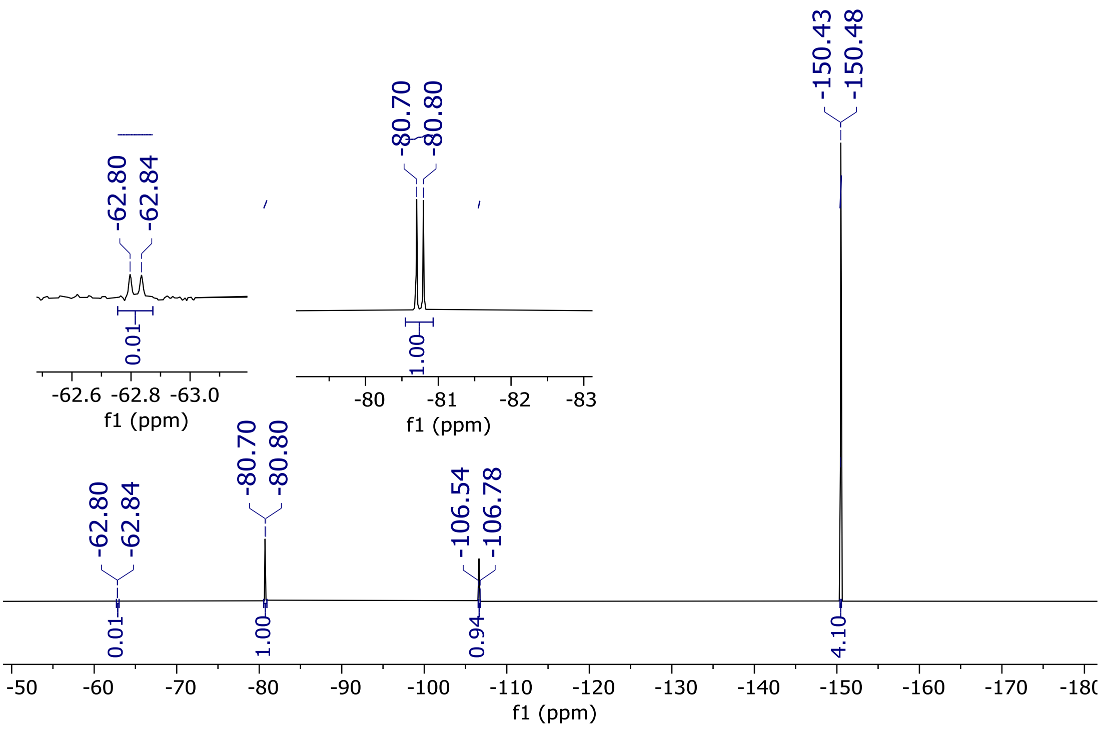

(Z)-(4-bromophenyl)(2-fluoro-2-phenylvinyl)iodonium BF_4 **(4j)**

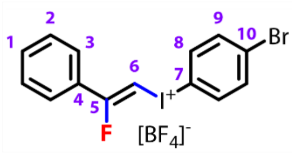

^{1}H NMR (400 MHz, $C(CD_3)_2O$):

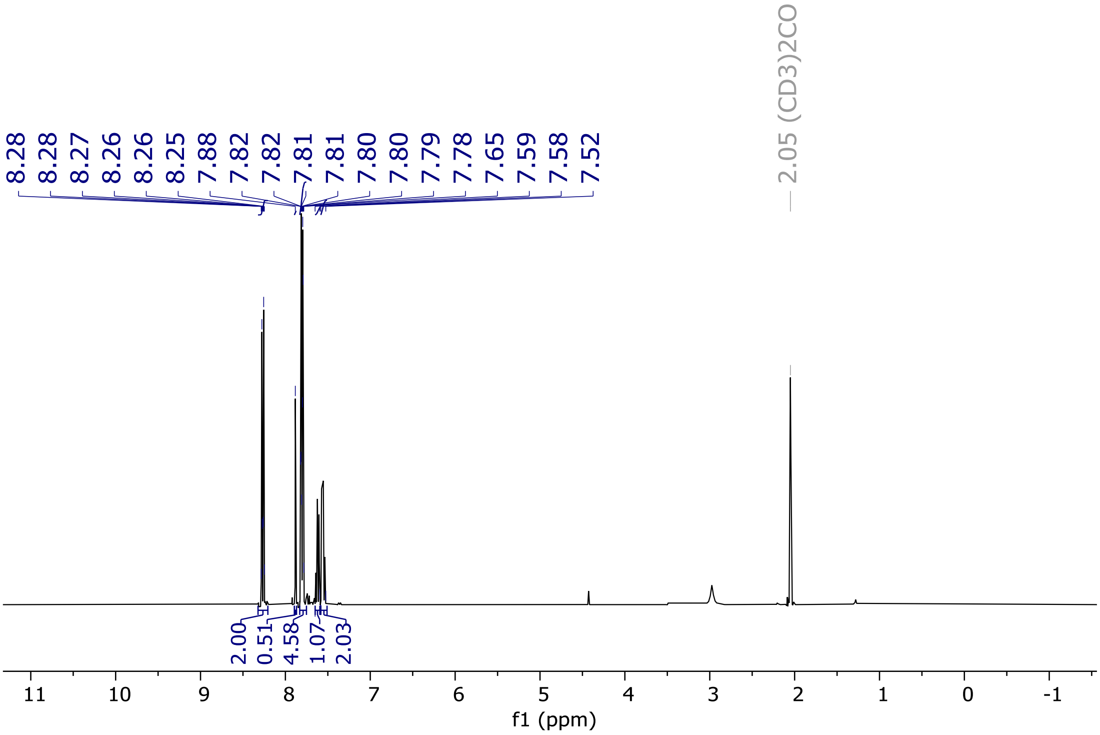

^{13}C NMR (126 MHz, CD_3CN):

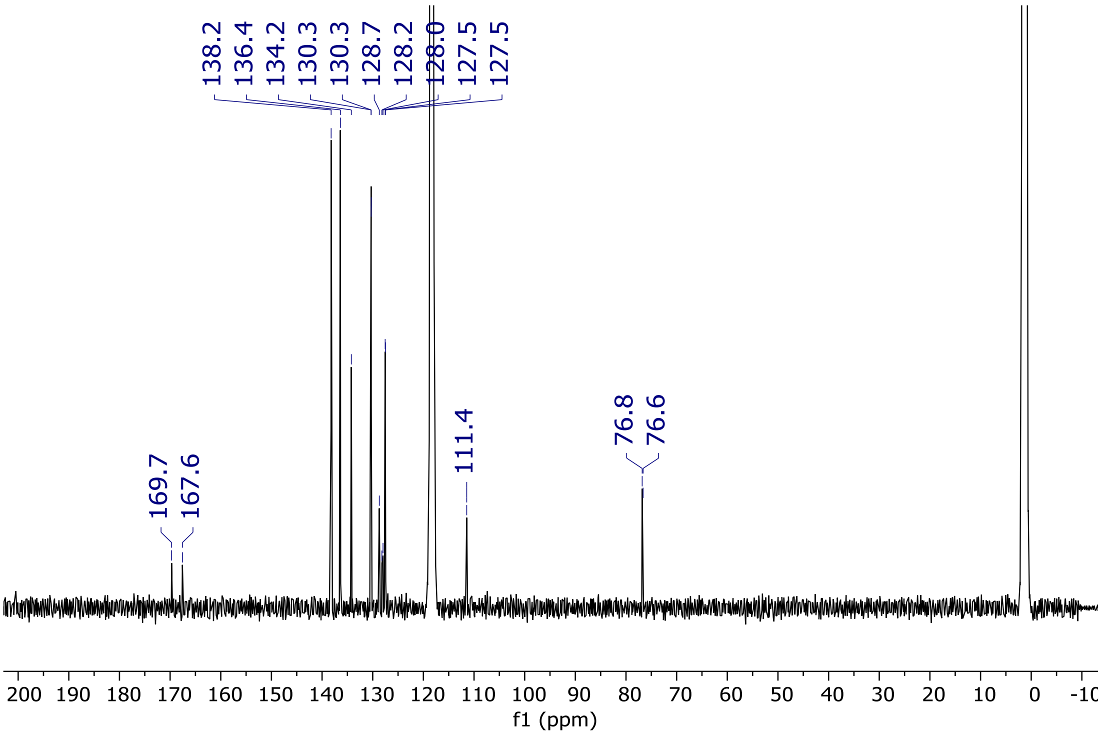

^{19}F NMR (376 MHz, $C(CD_3)_2O$):

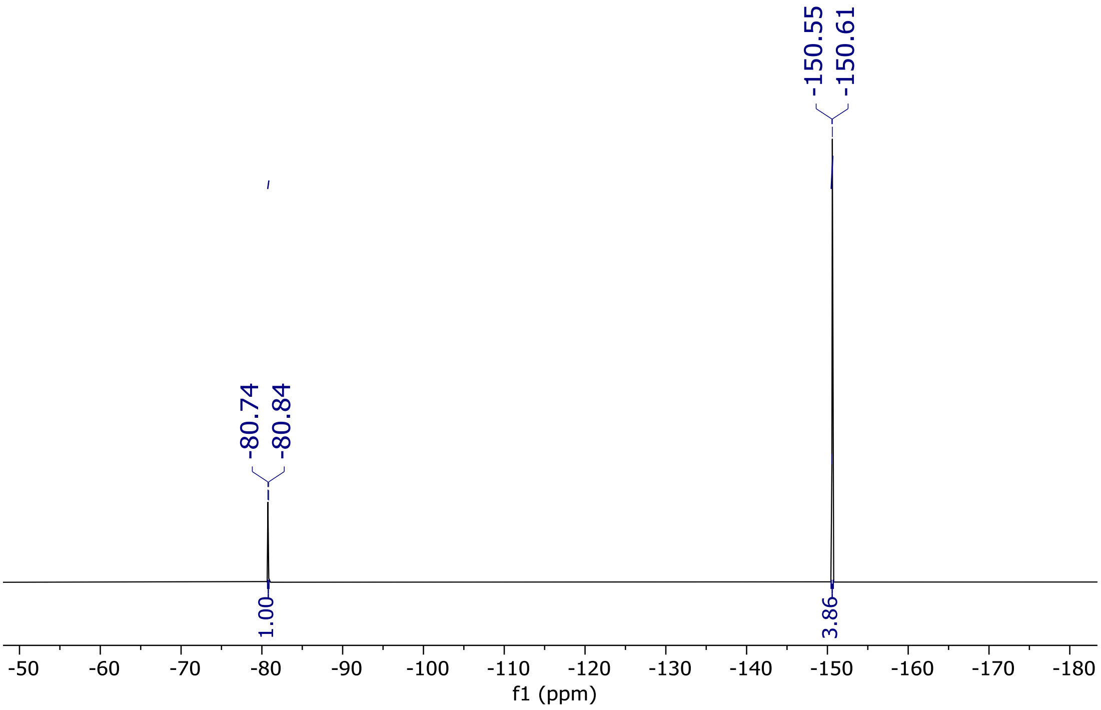

(Z)-(4-cyanophenyl)(2-fluoro-2-phenylvinyl)iodonium BF_4 ***(4k)***

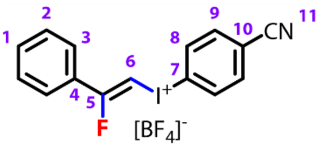

^{1}H NMR (500 MHz, CD_3CN):

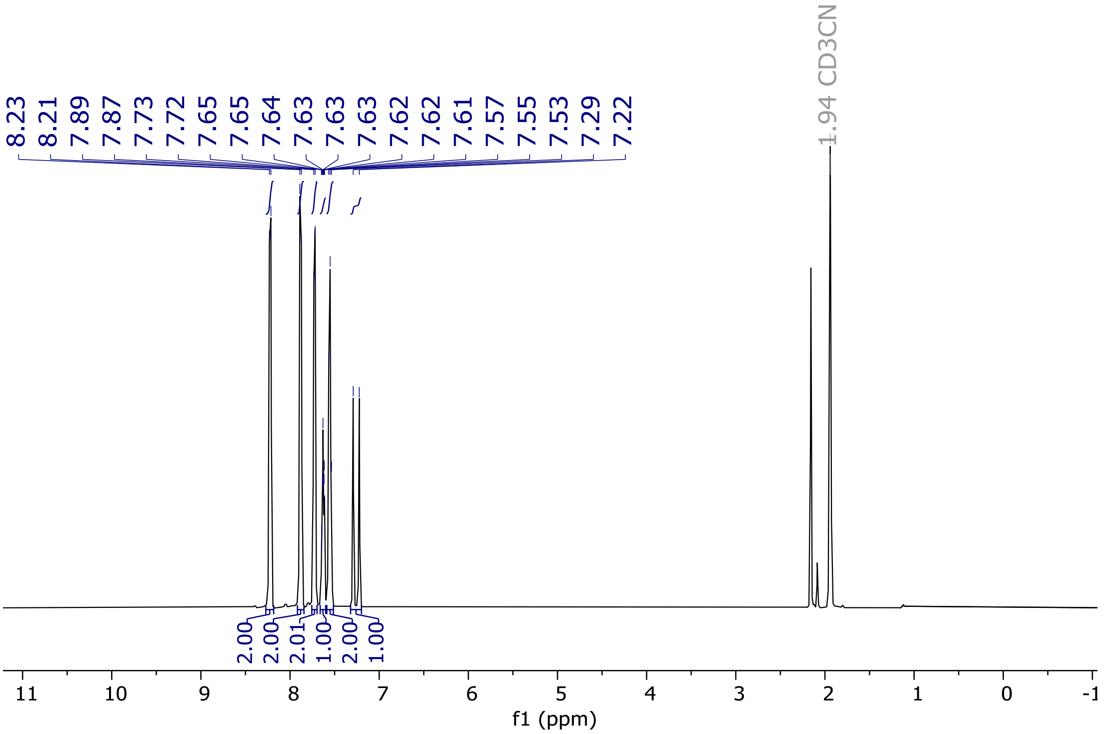

^{13}C NMR (126 MHz, CD_3CN):

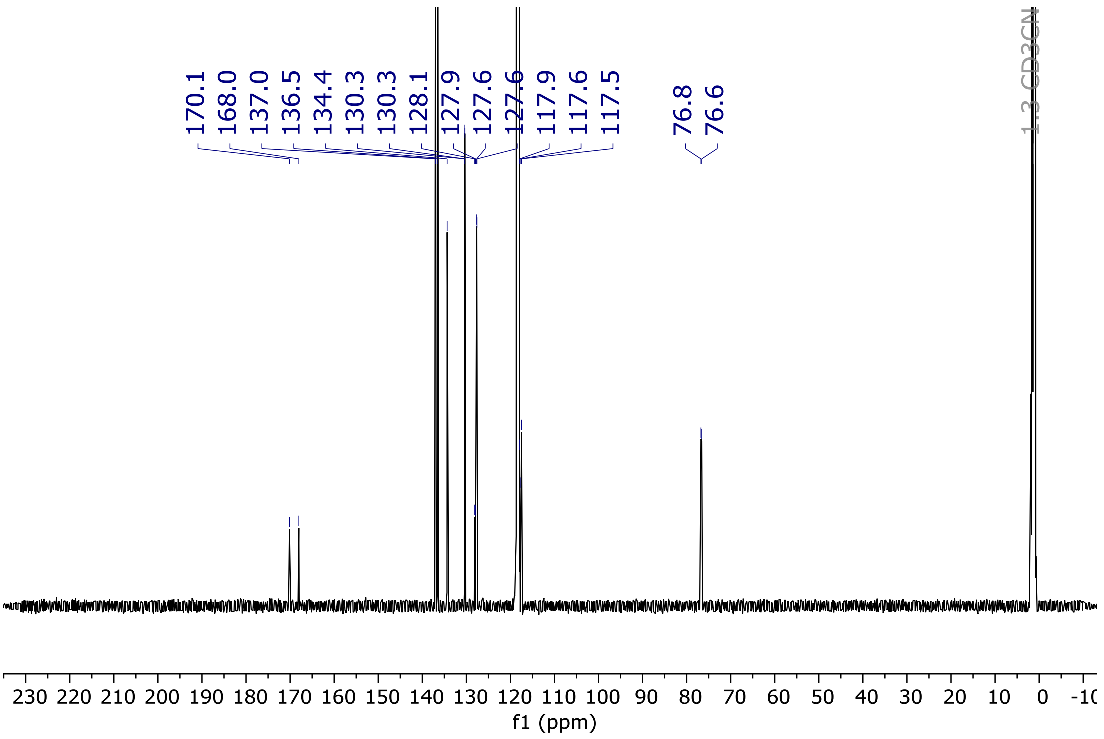

^{19}F NMR (376 MHz, CD_3CN):

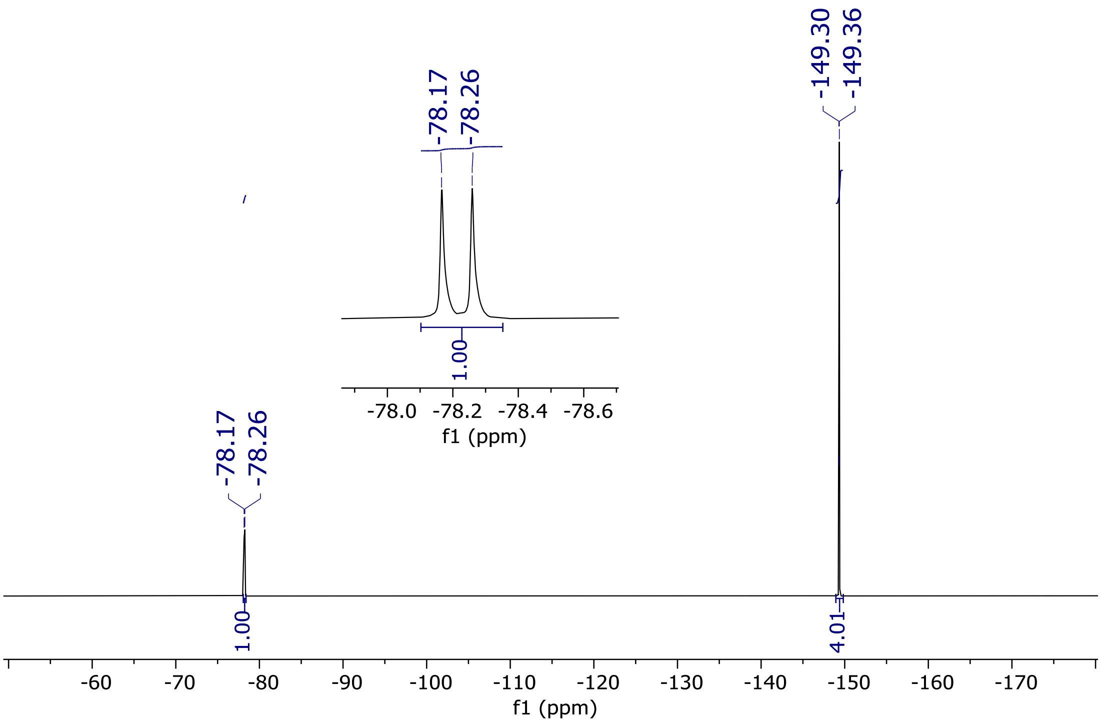

*<u>(Z)-(4-(tert-butyl)phenyl)(2-fluoro-2-phenylvinyl)iodonium BF$_4$ **(4l)**</u>*

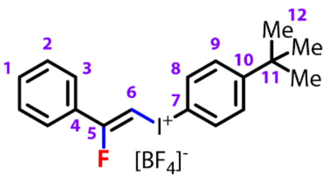

^{1}H NMR (500 MHz, (CD$_3$)$_2$CO):

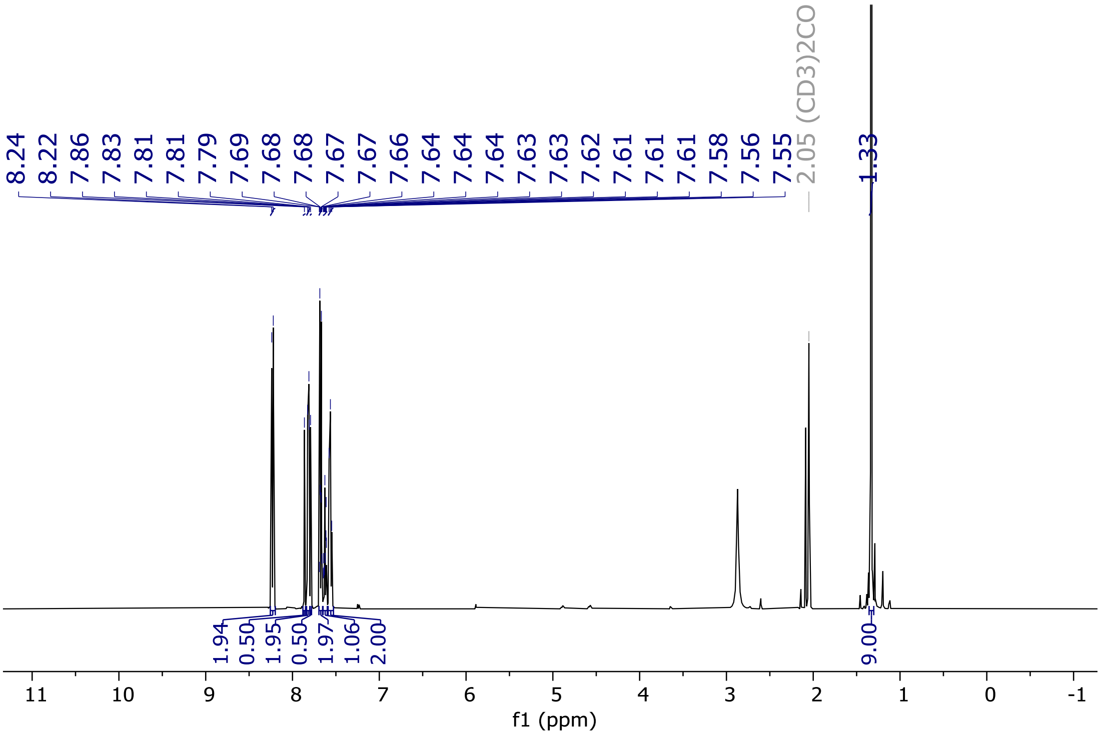

^{13}C NMR (126 MHz, C(CD$_3$)$_2$O):

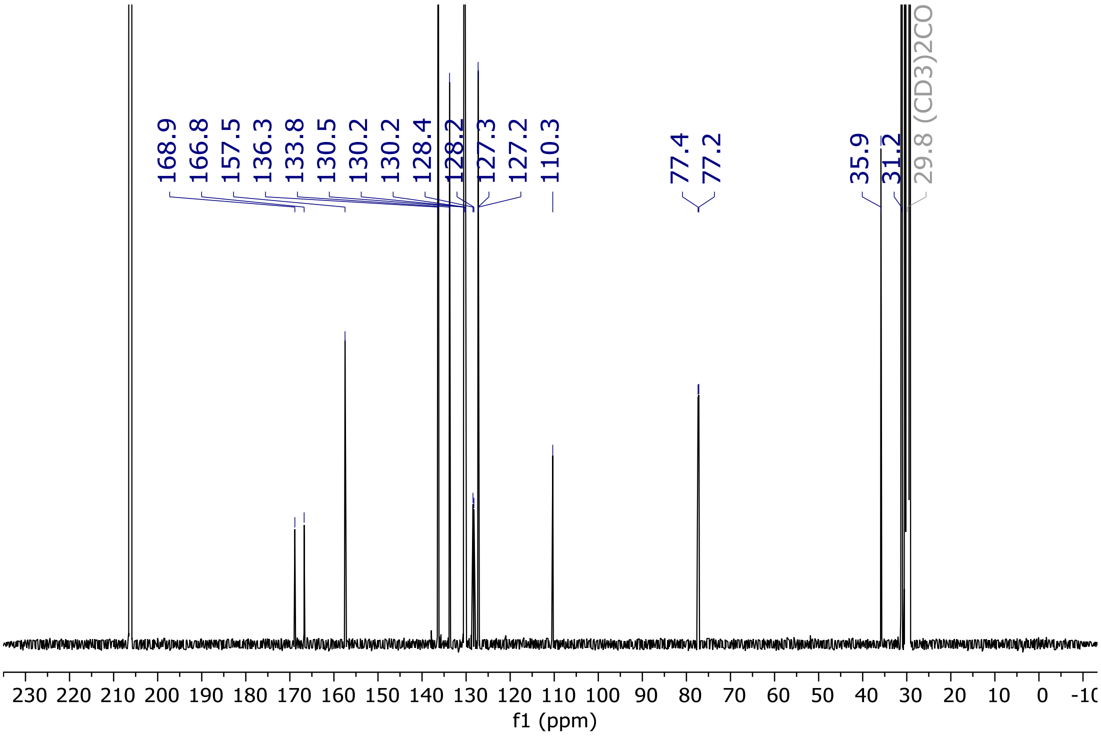

^{19}F NMR (376 MHz, $C(CD_3)_2O$):

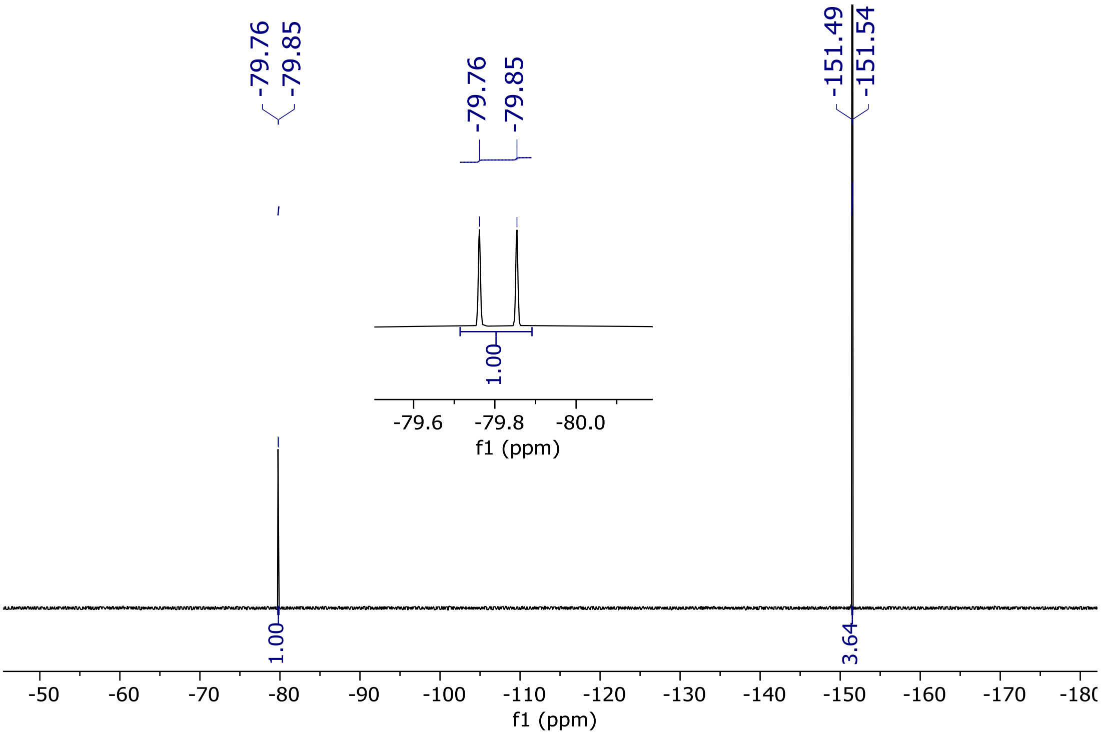

(5-Phenylpent-1-yn-1-yl)(2,4,6-triisopropylphenyl)iodonium BF_4
(III-b)

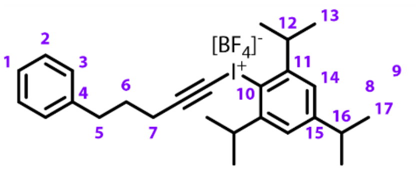

^{1}H NMR (500 MHz, $C(CD_3)_2O$):

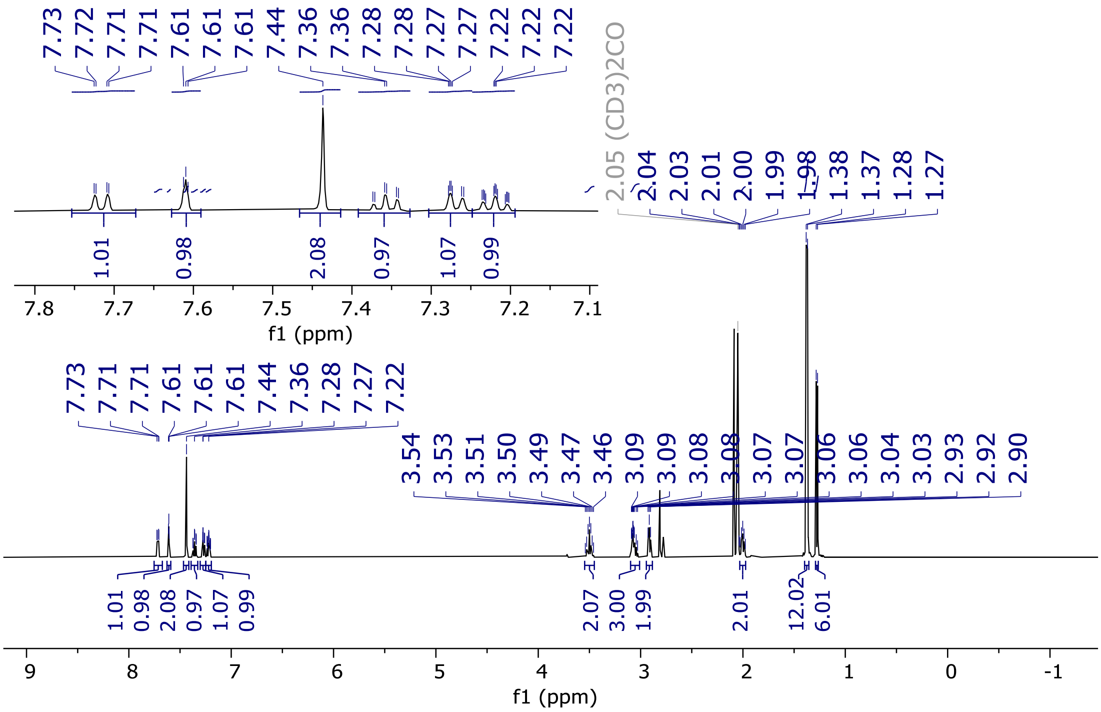

^{13}C NMR (126 MHz, $C(CD_3)_2O$):

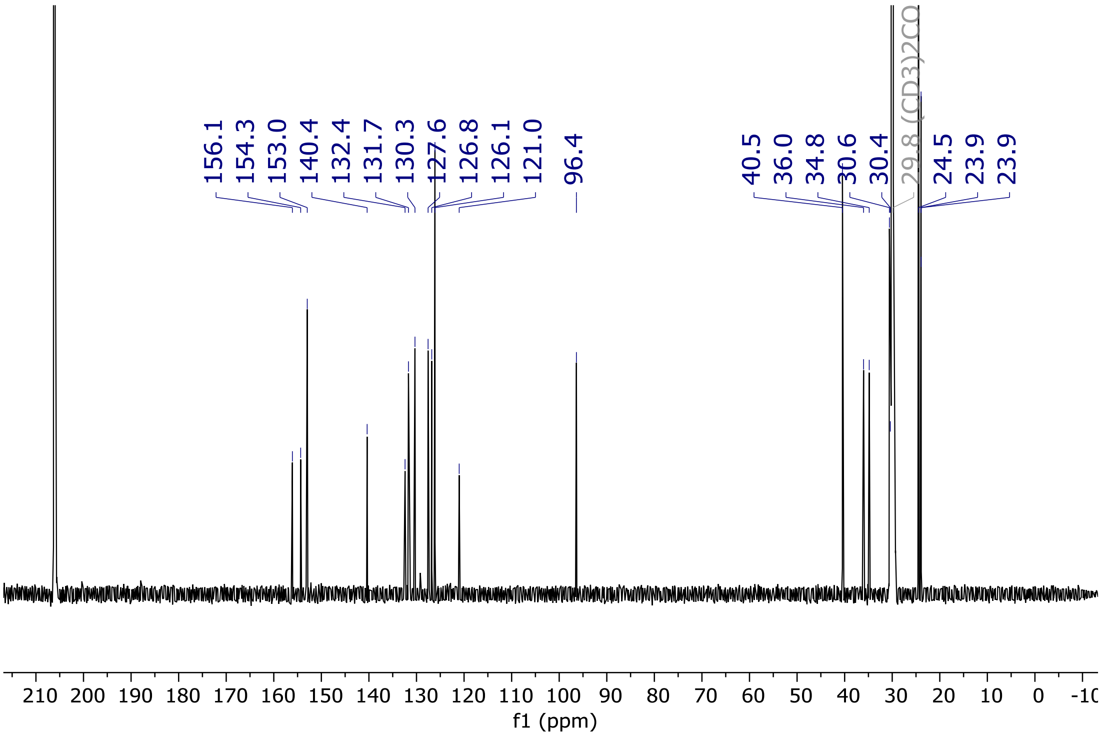

(Z)-(5-bromo-4-fluoropent-4-en-1-yl)benzene ***(5a)***

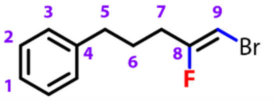

^{1}H NMR (400 MHz, $CDCl_3$):

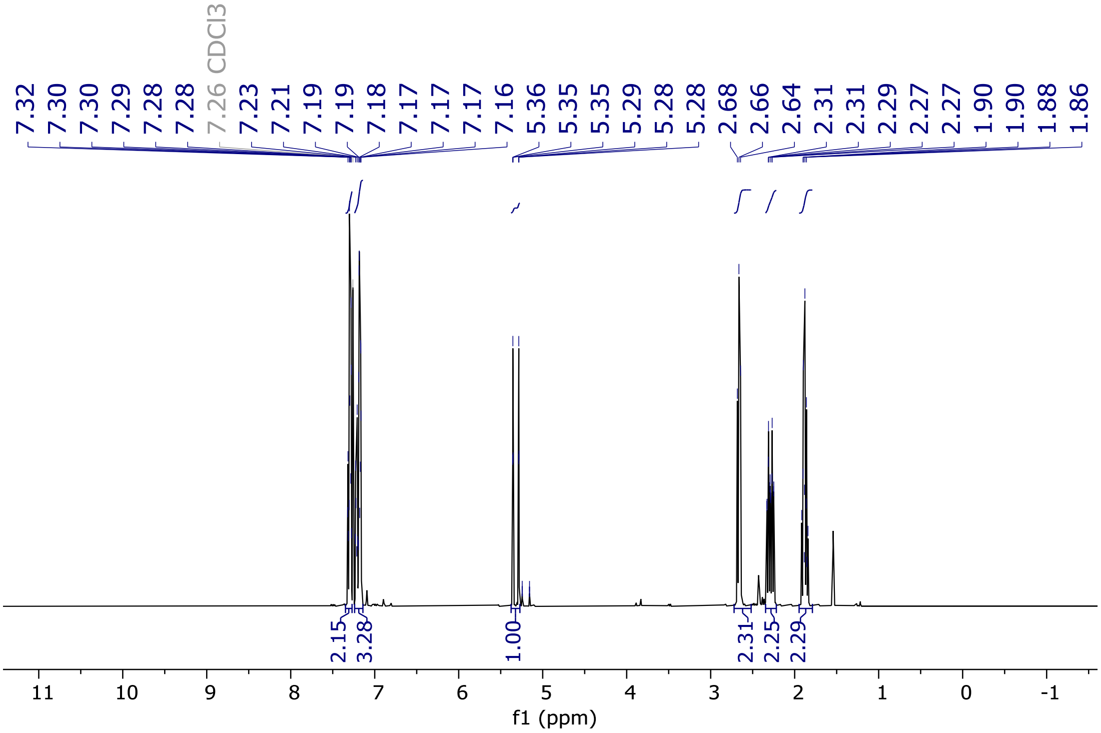

^{13}C NMR (126 MHz, $CDCl_3$):

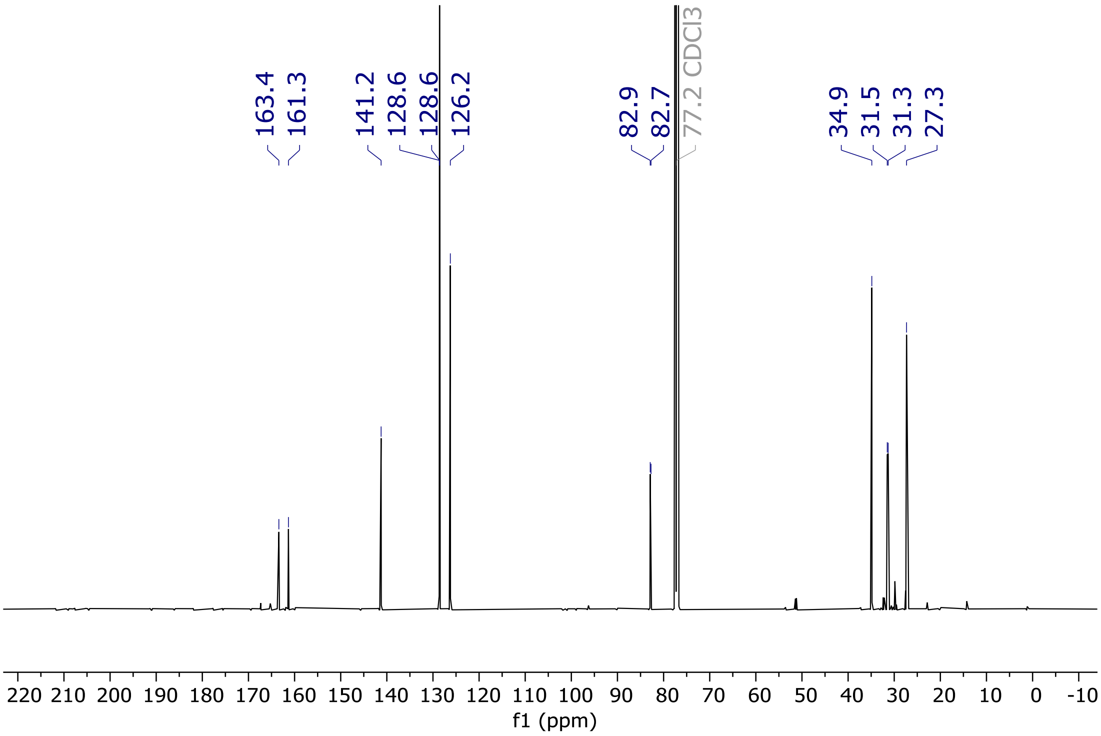

^{19}F NMR (376 MHz, $CDCl_3$):

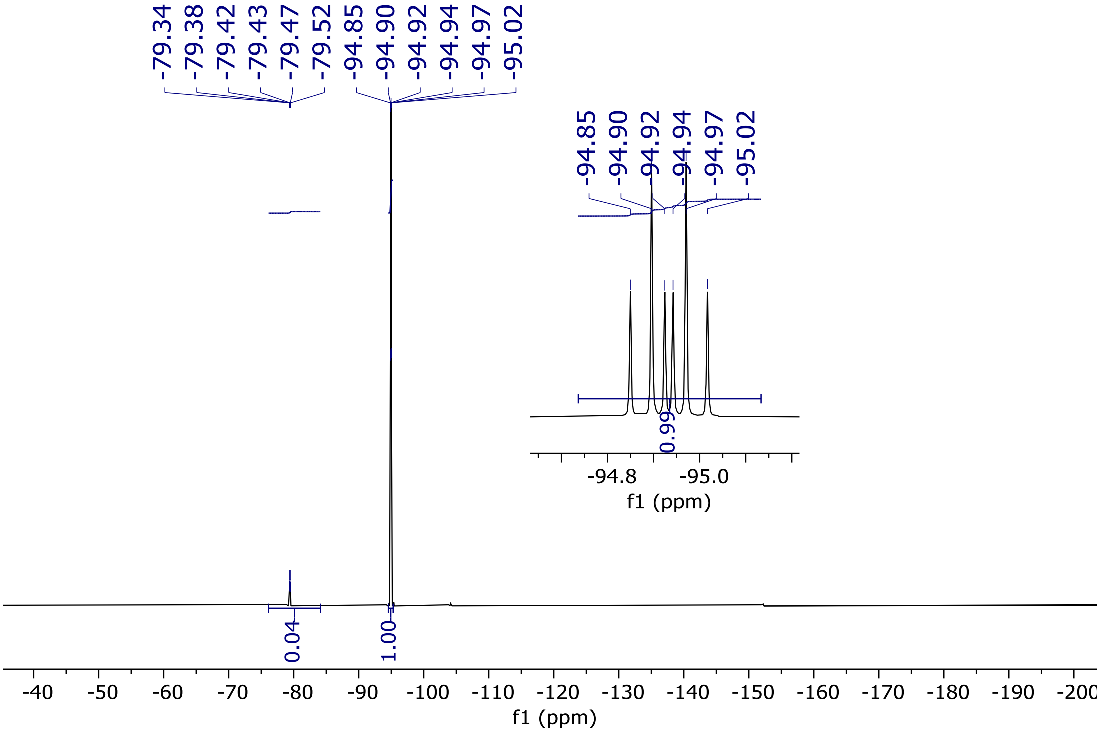

(Z)-1-((3-bromo-2-fluoroallyl)oxy)-4-fluorobenzene ***(5b)***

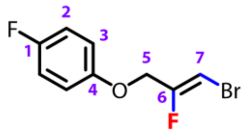

^{1}H NMR (500 MHz, $CDCl_3$):

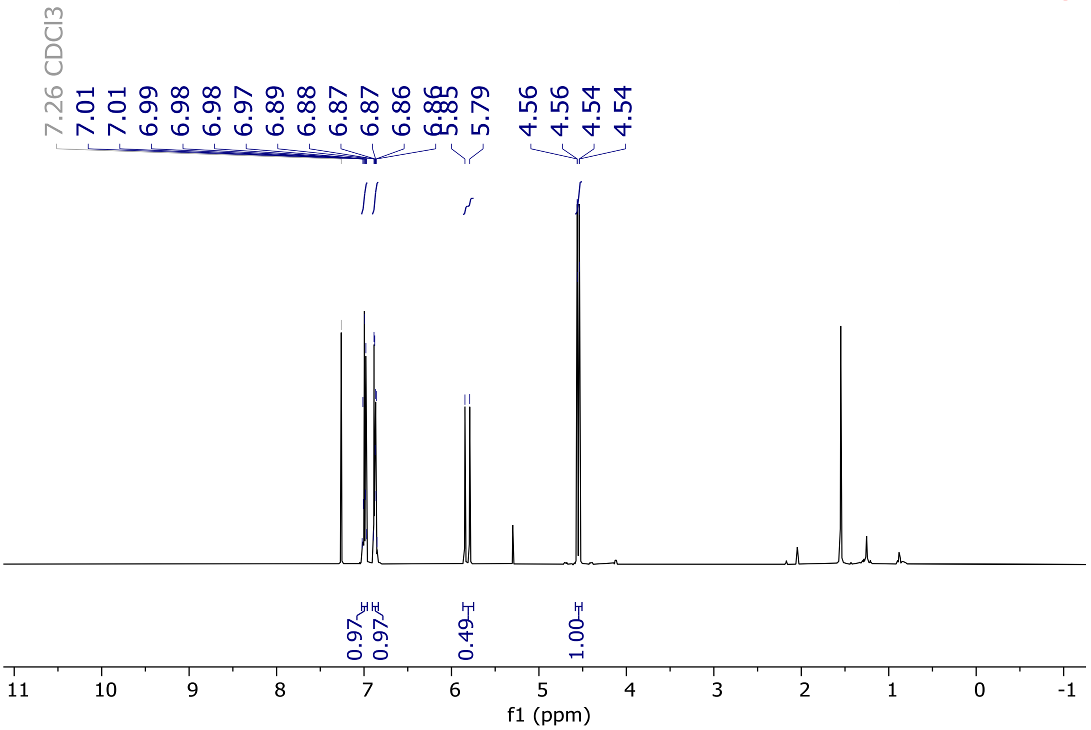

^{13}C NMR (126 MHz, $CDCl_3$):

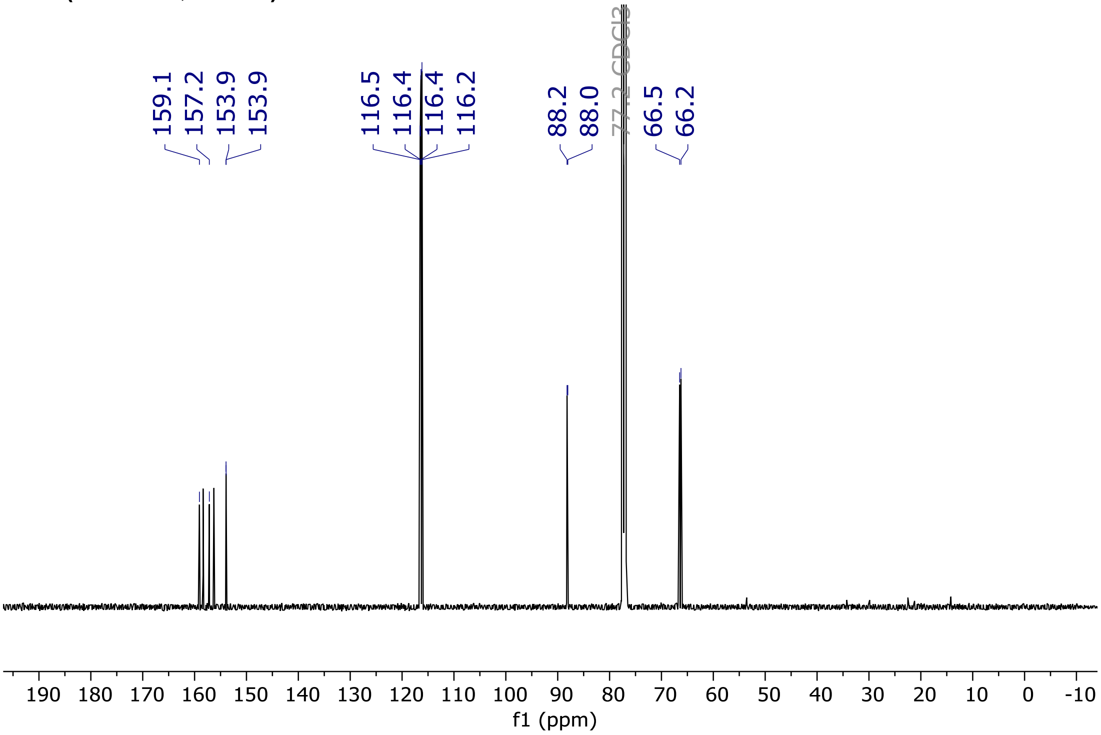

^{19}F NMR (376 MHz, $CDCl_3$):

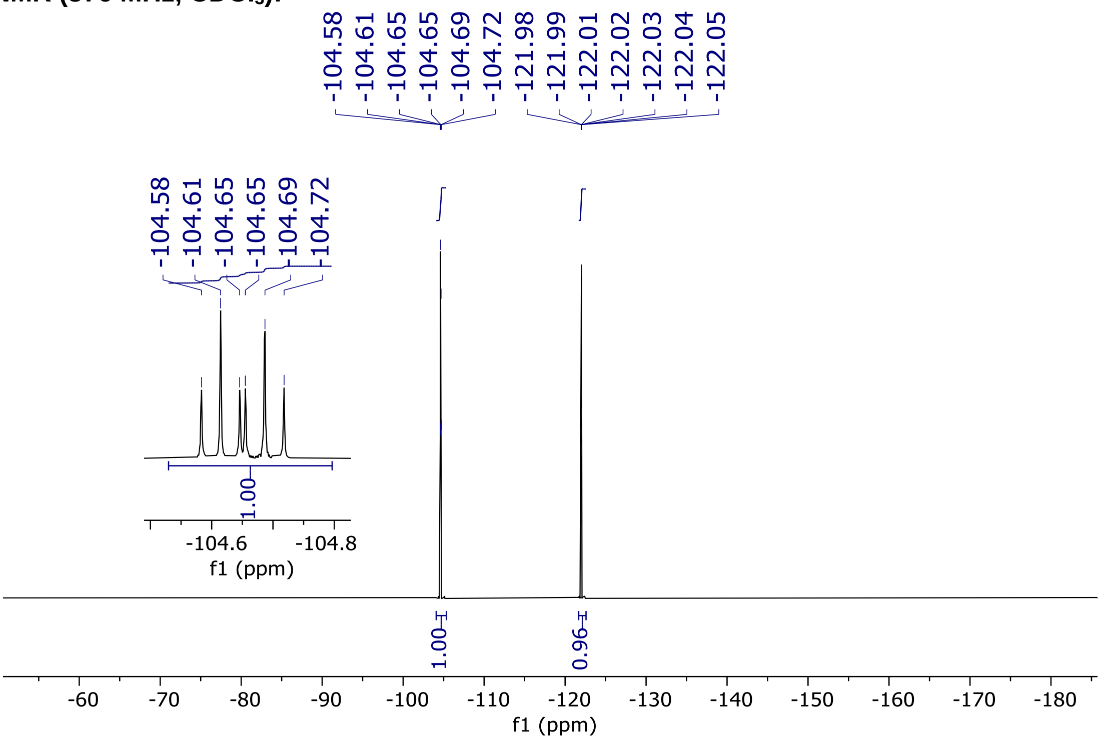

(Z)-2-(3-bromo-2-fluoroallyl)isoindoline-1,3-dione ***(5c)***

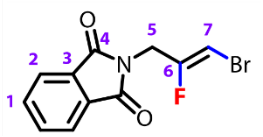

^{1}H NMR (500 MHz, $CDCl_3$):

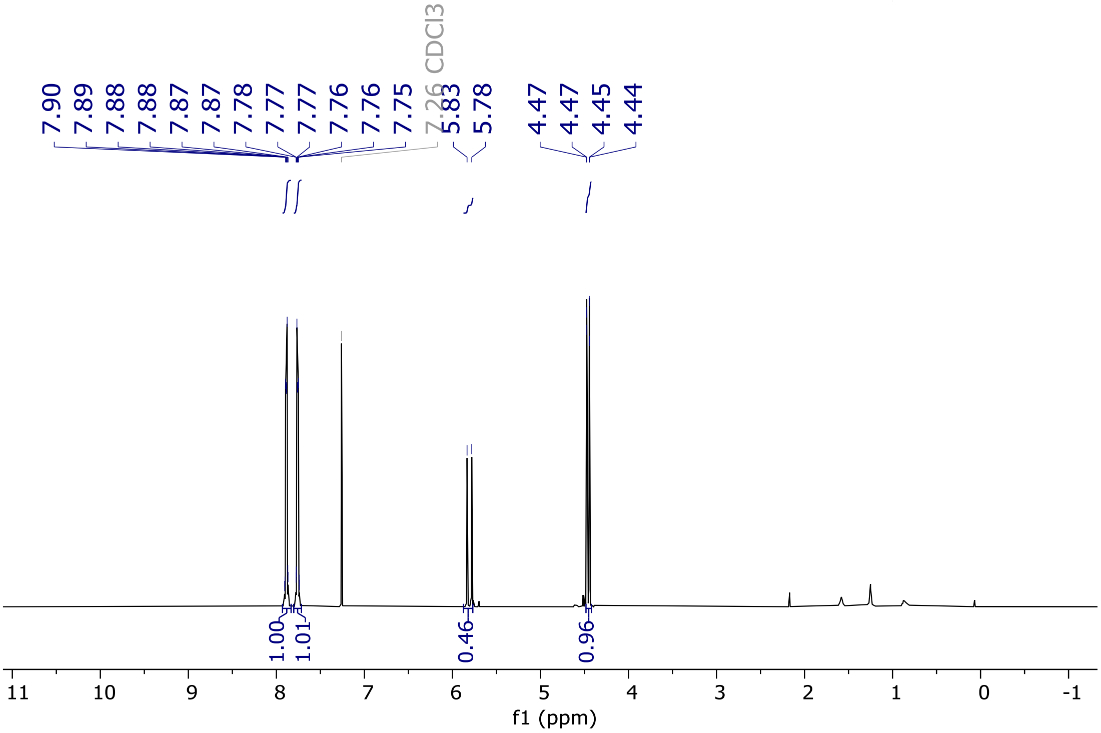

^{13}C NMR (126 MHz, $CDCl_3$):

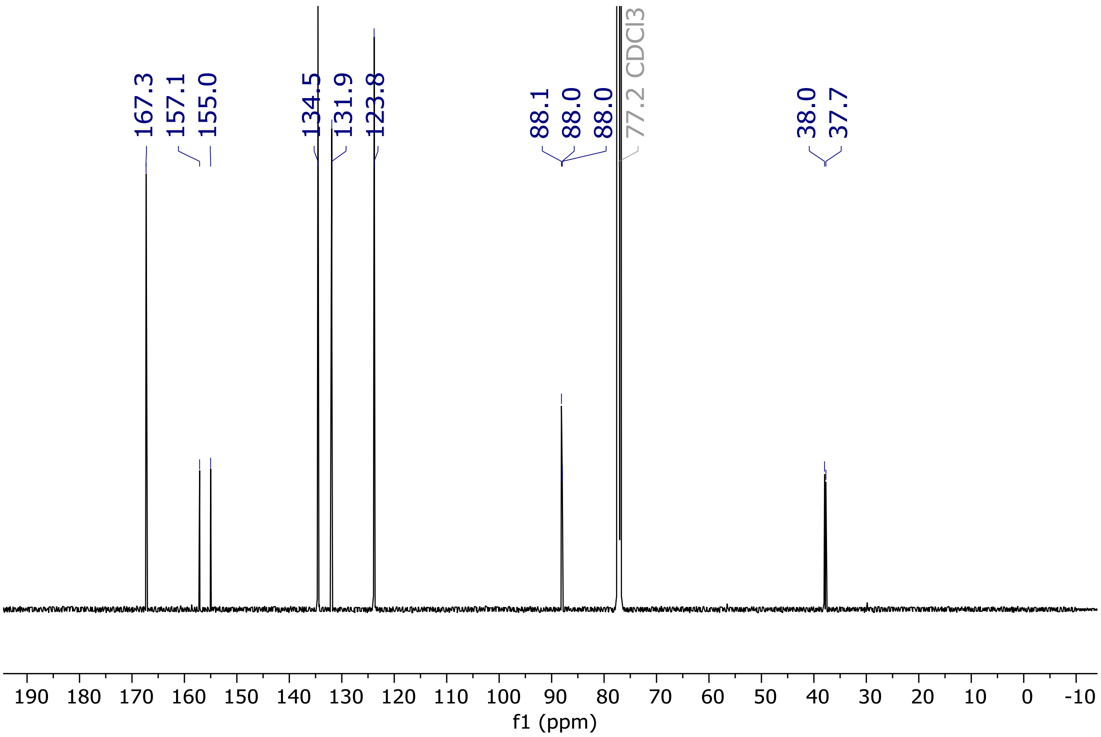

^{19}F NMR (376 MHz, $CDCl_3$):

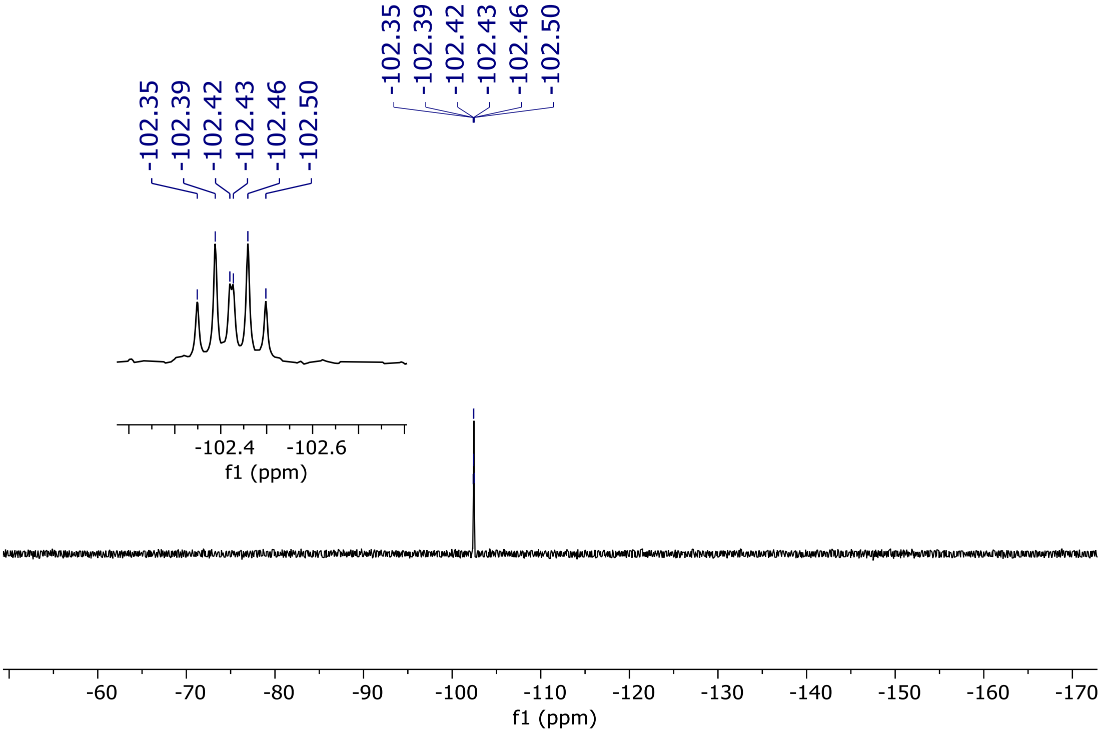

*(Z)-N-(3-bromo-2-fluoroallyl)-N-(4-fluorobenzyl)-4-methylbenzenesulfonamide **(5d)***

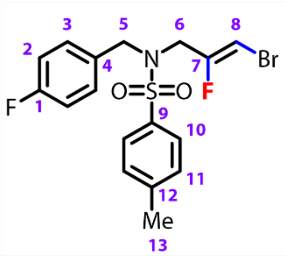

^{1}H NMR (500 MHz, $CDCl_3$):

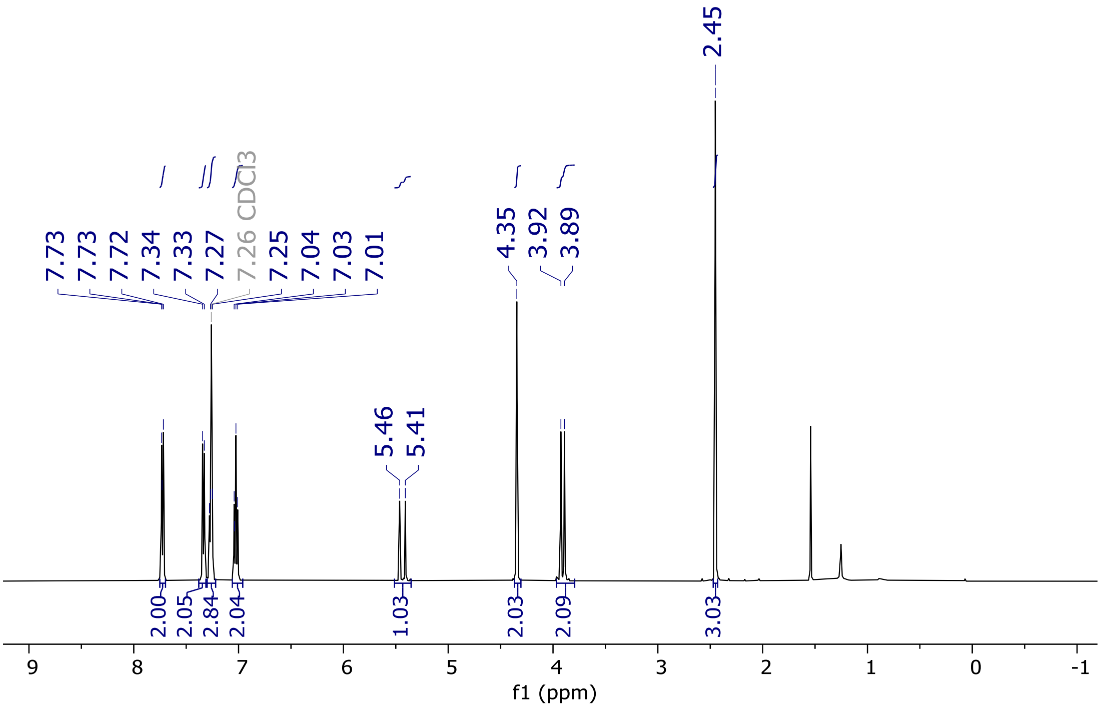

^{13}C NMR (126 MHz, $CDCl_3$):

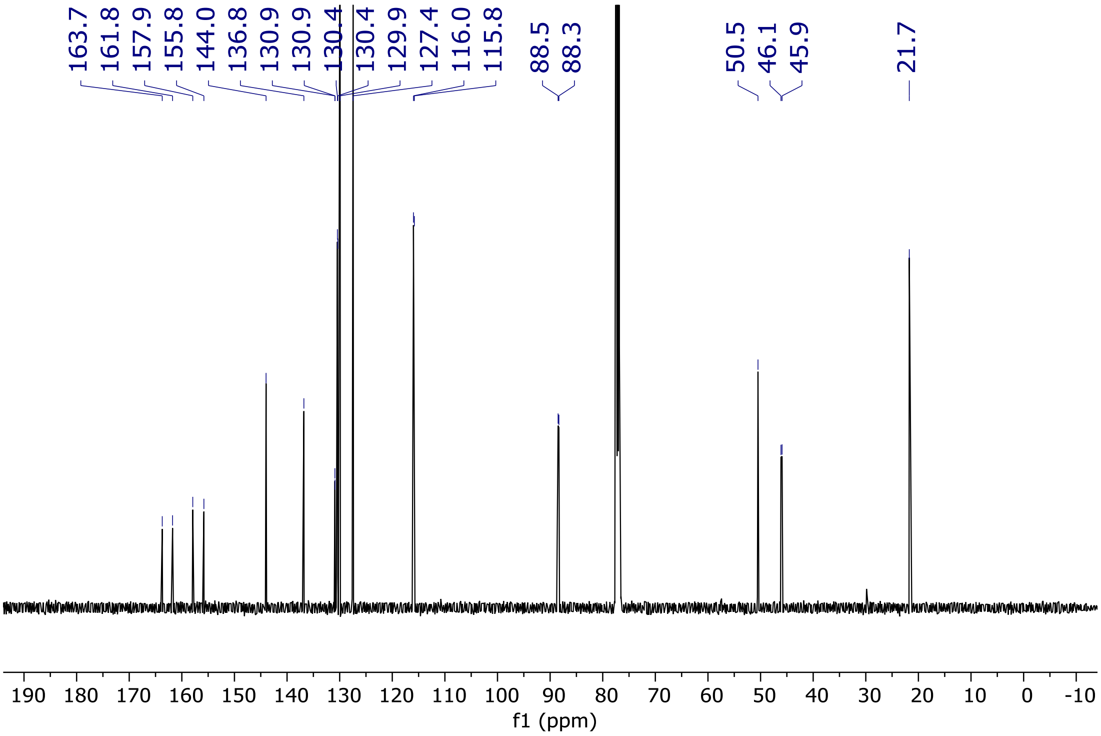

^{19}F NMR (376 MHz, CDCl$_3$):

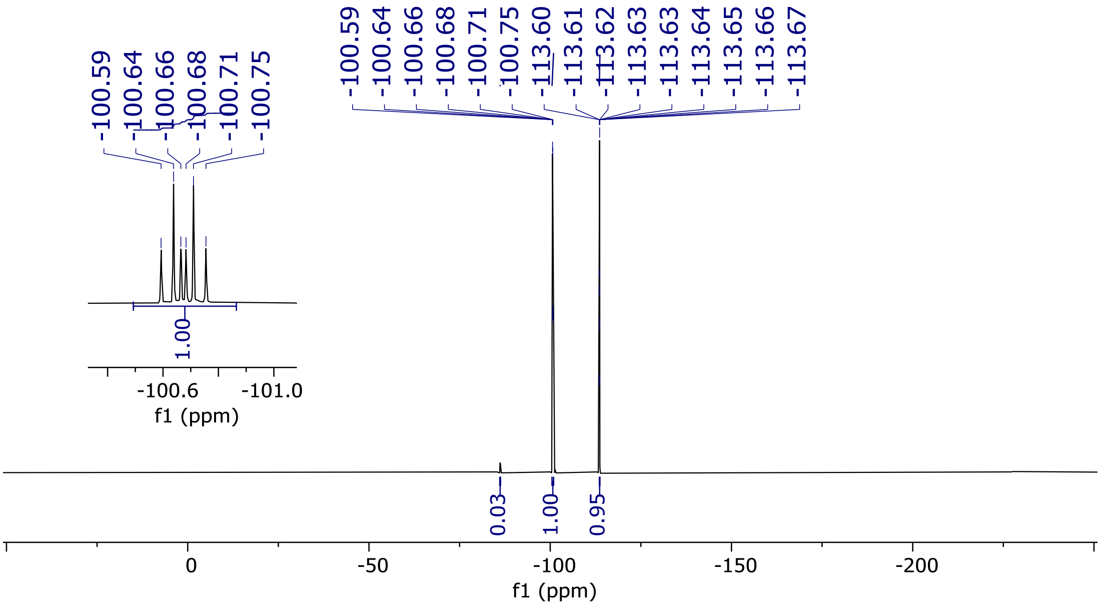

(Z)-(5-chloro-4-fluoropent-4-en-1-yl)benzene ***(6a)***

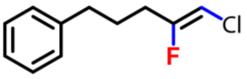

^{1}H NMR (500 MHz, CDCl$_3$):

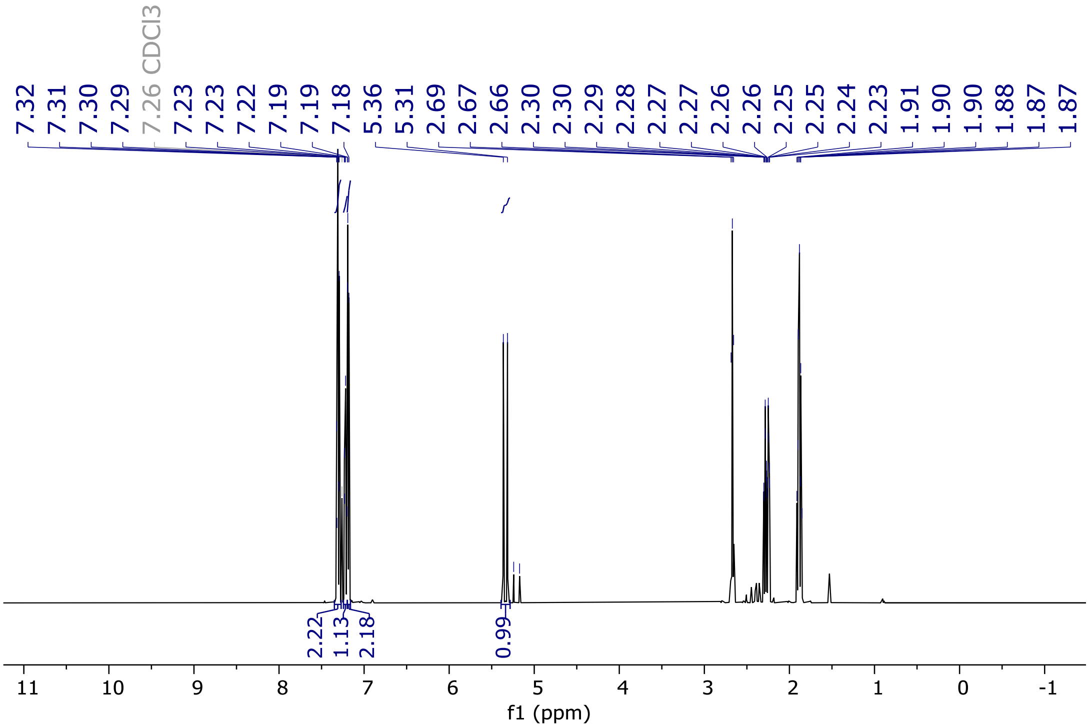

^{13}C NMR (126 MHz, CDCl$_3$):

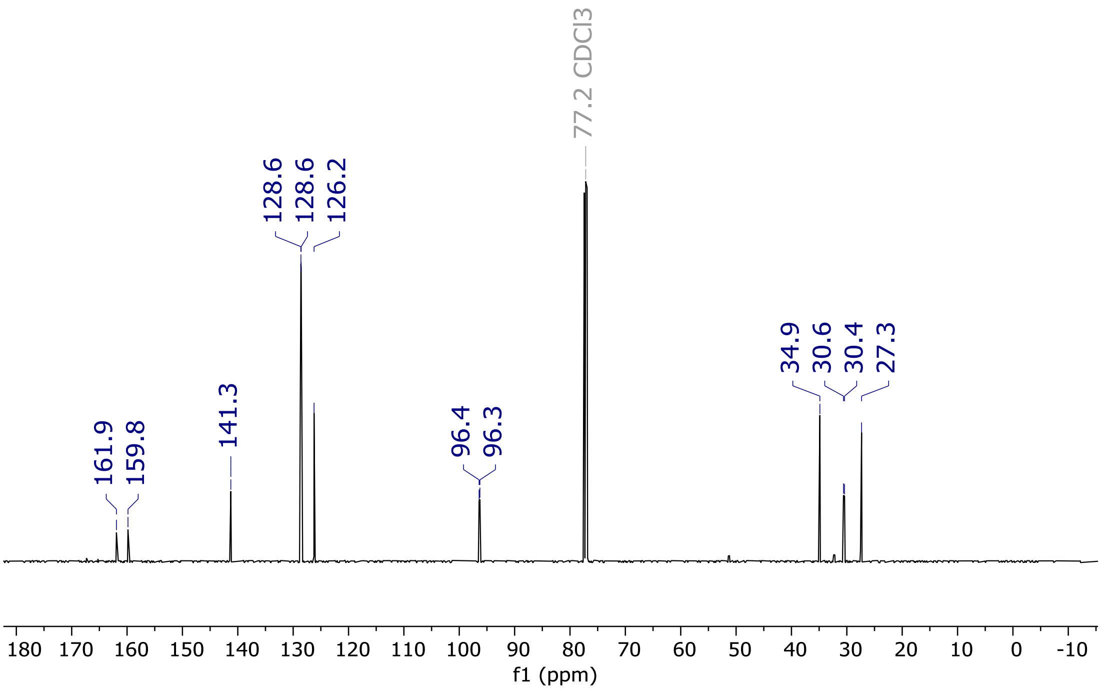

^{19}F (376 MHz, CDCl$_3$):

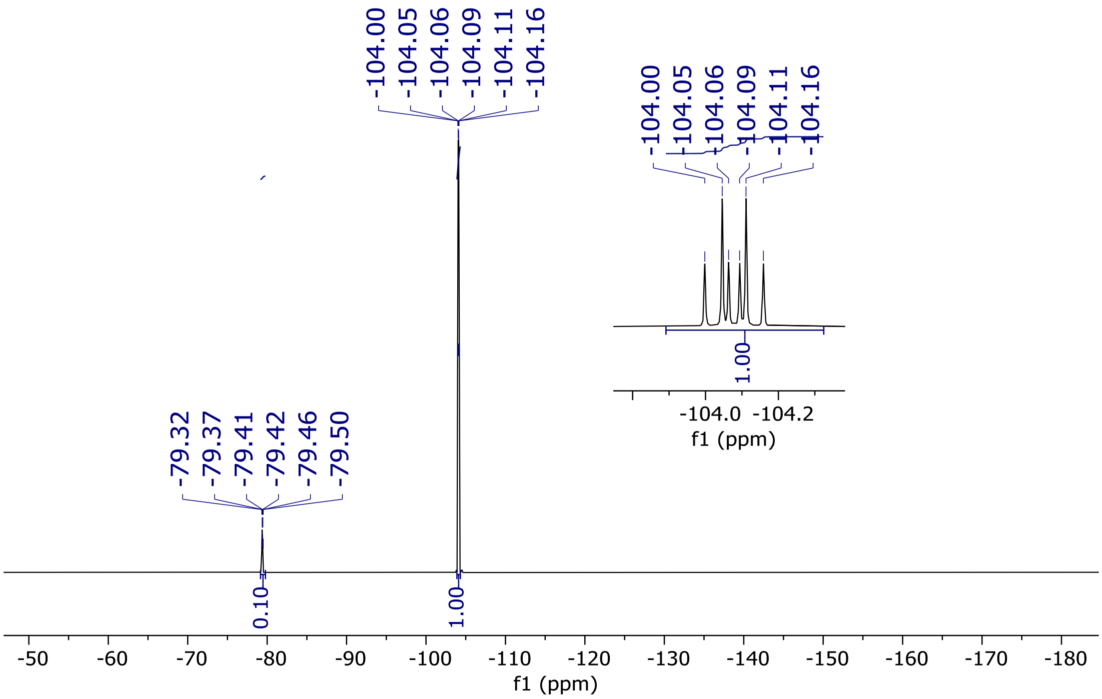

(Z)-(2-fluoro-5-phenylpent-1-en-1-yl)(4-fluorophenyl)sulfane ***(7a)***

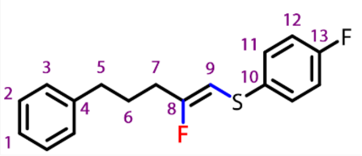

^{1}H NMR (500 MHz, $CDCl_3$):

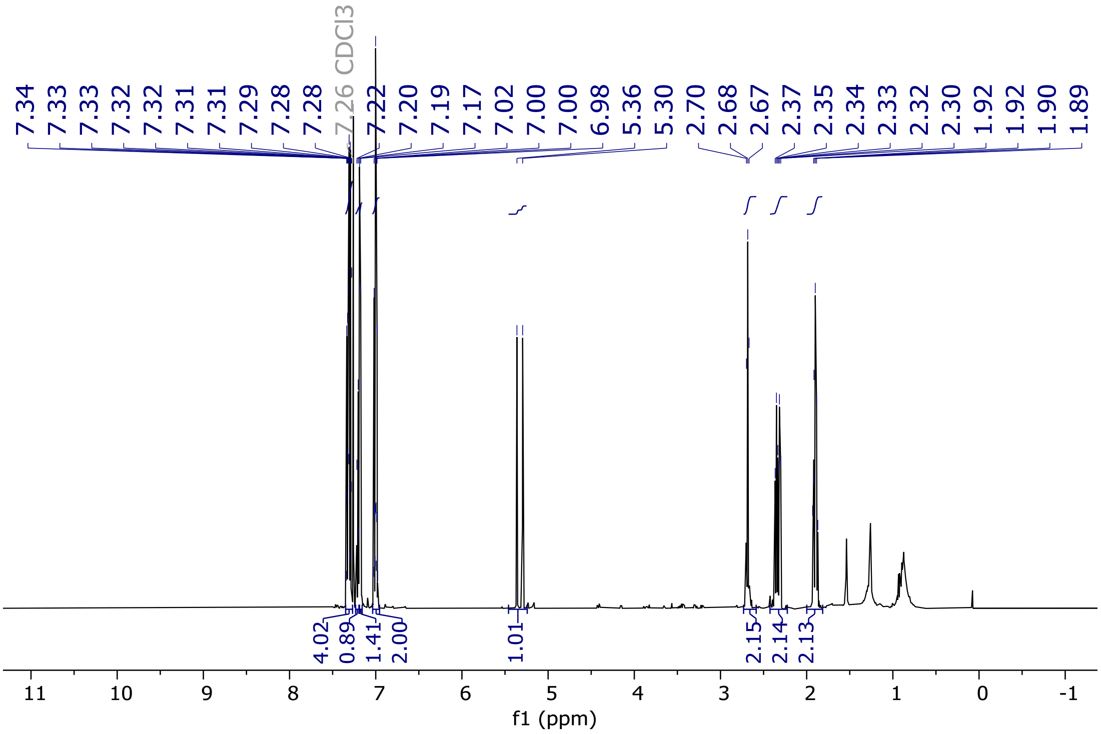

^{13}C NMR (126 MHz, $CDCl_3$):

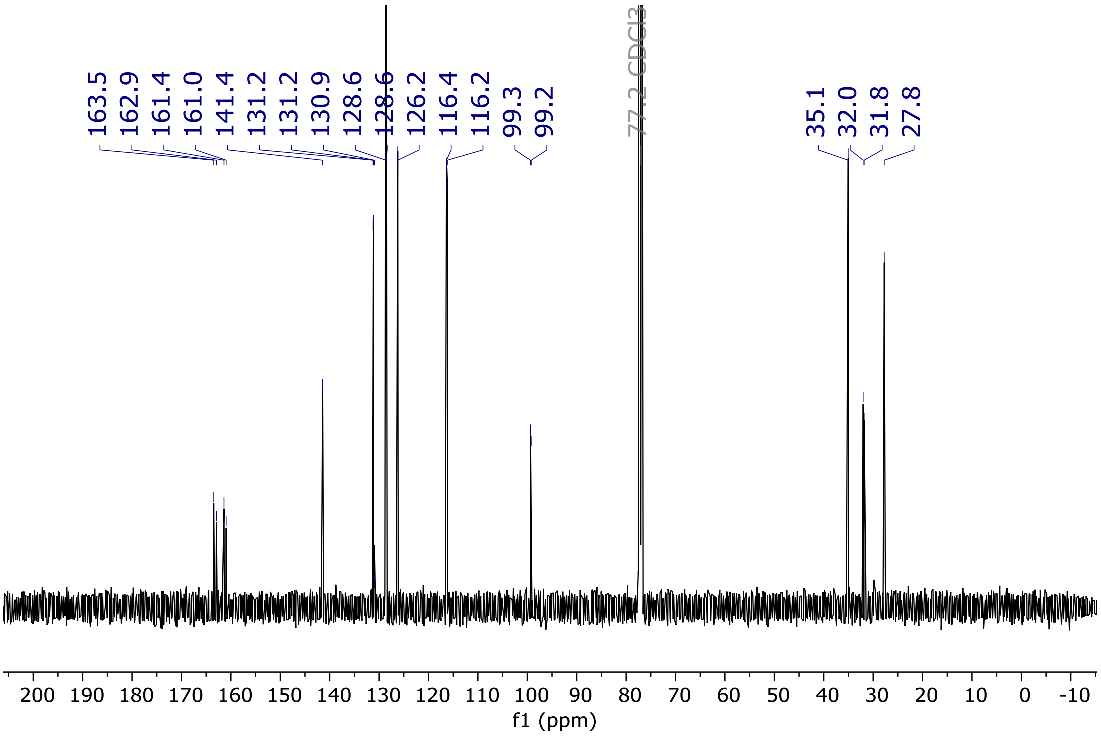

^{19}F NMR (376 MHz, CDCl$_3$):

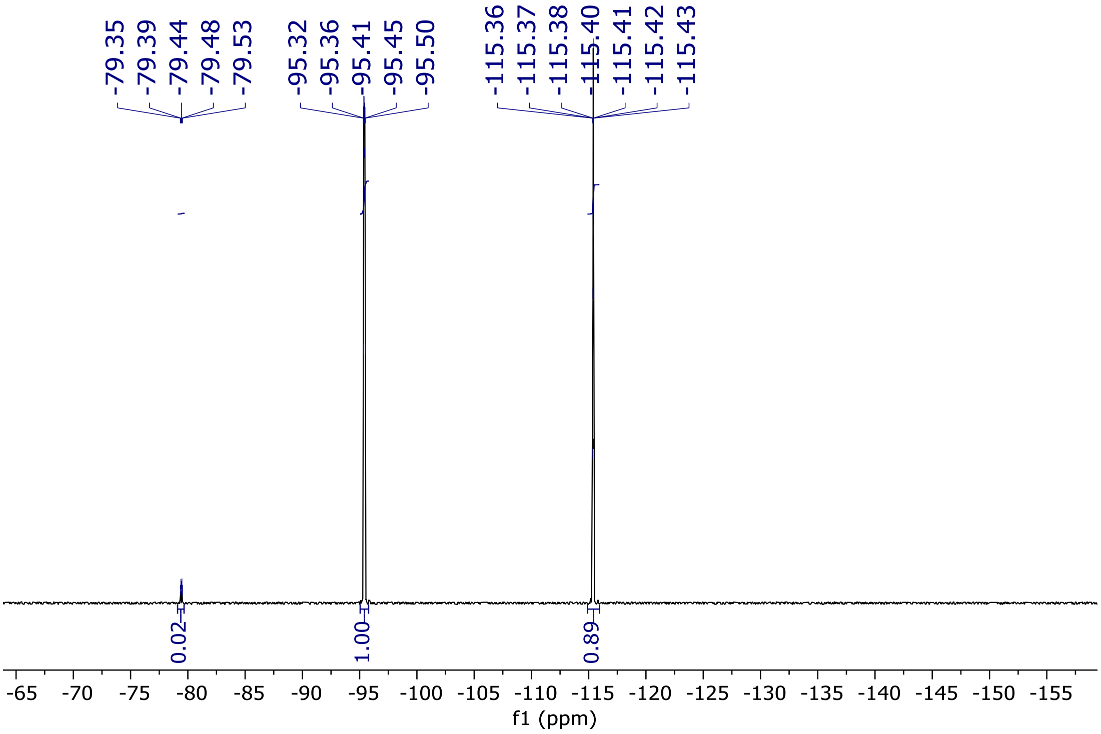

(Z)-(2-fluoro-2-phenylvinyl)(4-nitrophenyl)sulfane ***(7b)***

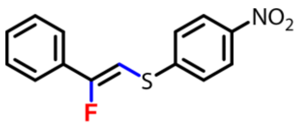

^{1}H NMR (500 MHz, $CDCl_3$):

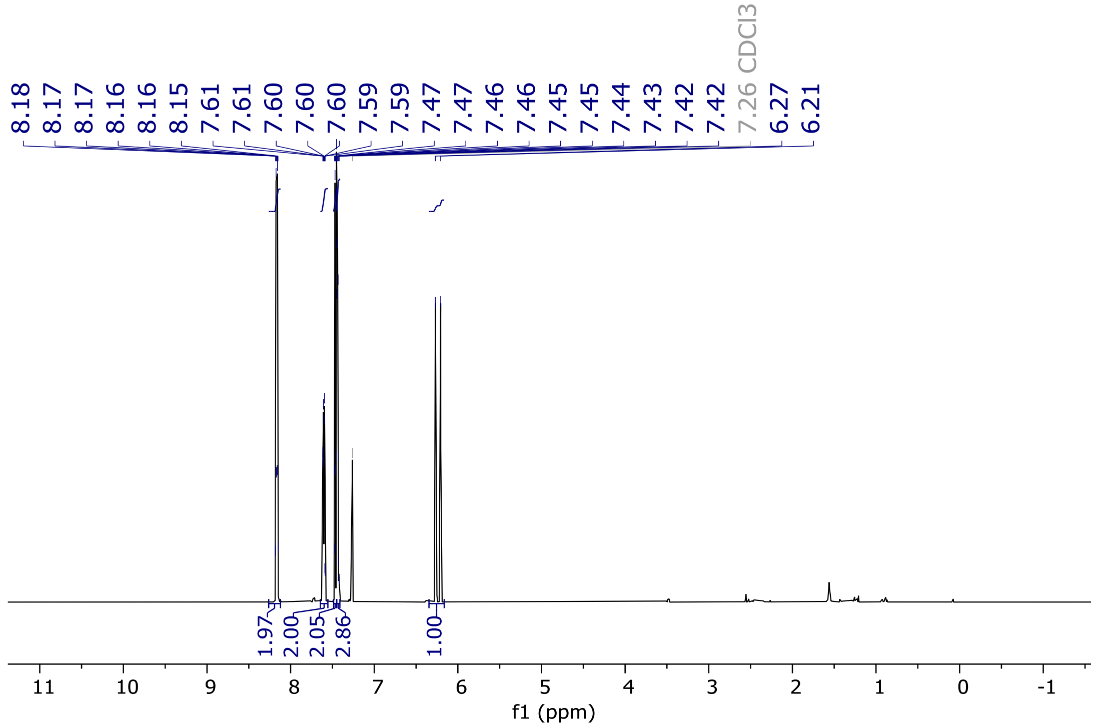

^{13}C NMR (126 MHz, $CDCl_3$):

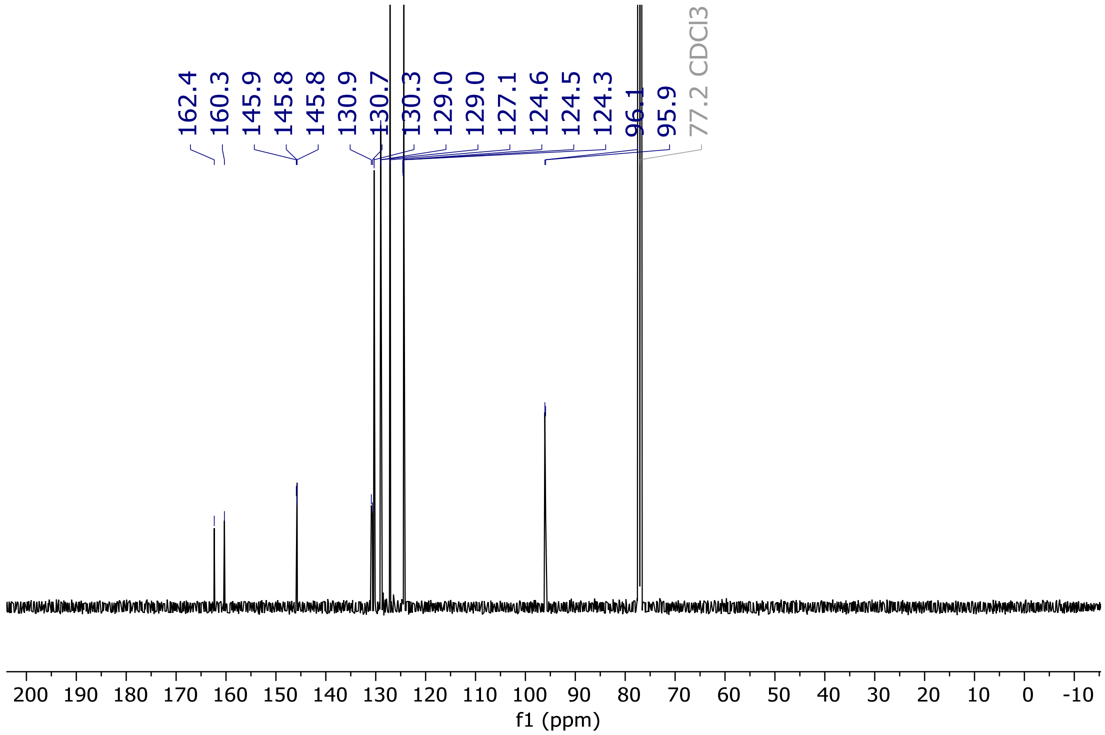

^{19}F NMR (376 MHz, CDCl$_3$):

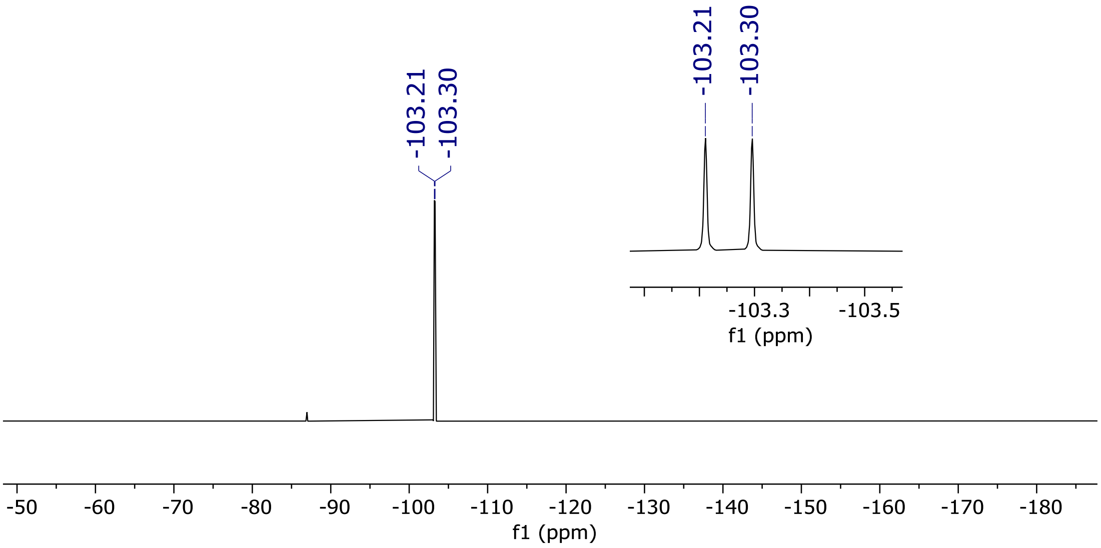

(Z)-(2-fluoro-2-phenylvinyl)(4-methoxyphenyl)sulfane ***(7c)***

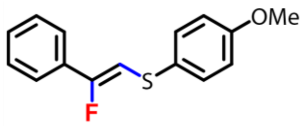

^{1}H NMR (600 MHz, $CDCl_3$):

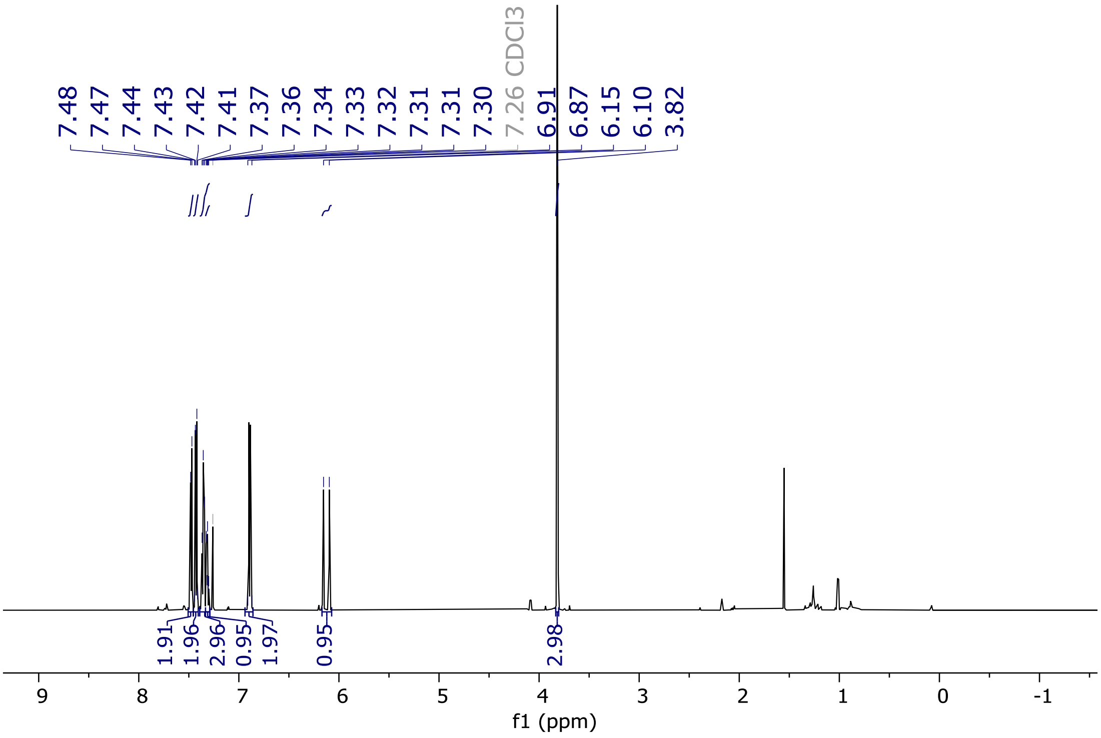

^{13}C NMR (151 MHz, $CDCl_3$):

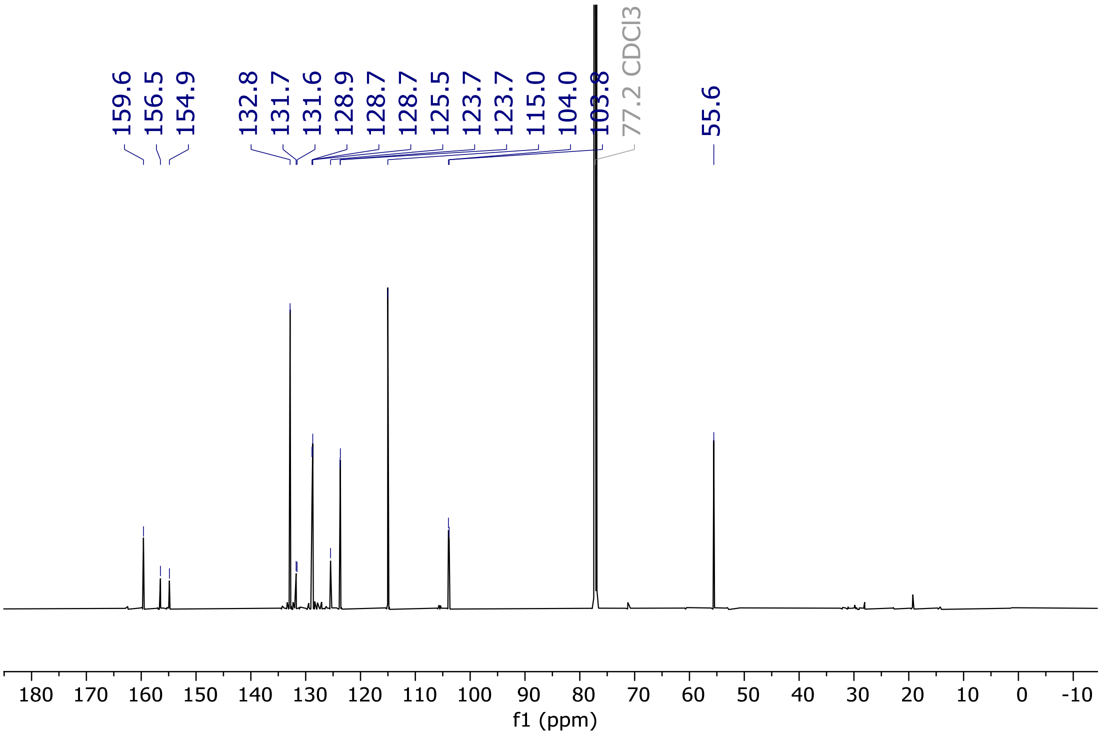

^{19}F NMR (376 MHz, $CDCl_3$):

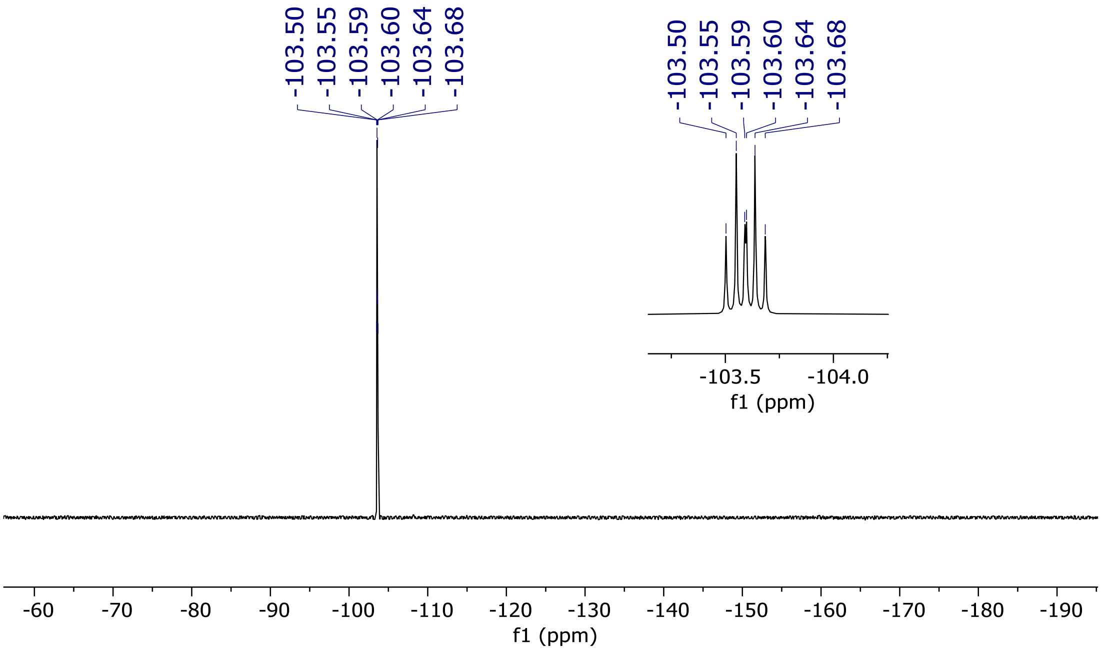

(Z)-(2-fluoro-3,3-dimethylbut-1-en-1-yl)(4-fluorophenyl)sulfane ***(7d)***

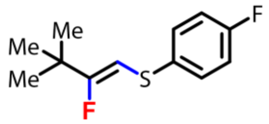

^{1}H NMR (500 MHz, CDCl$_3$):

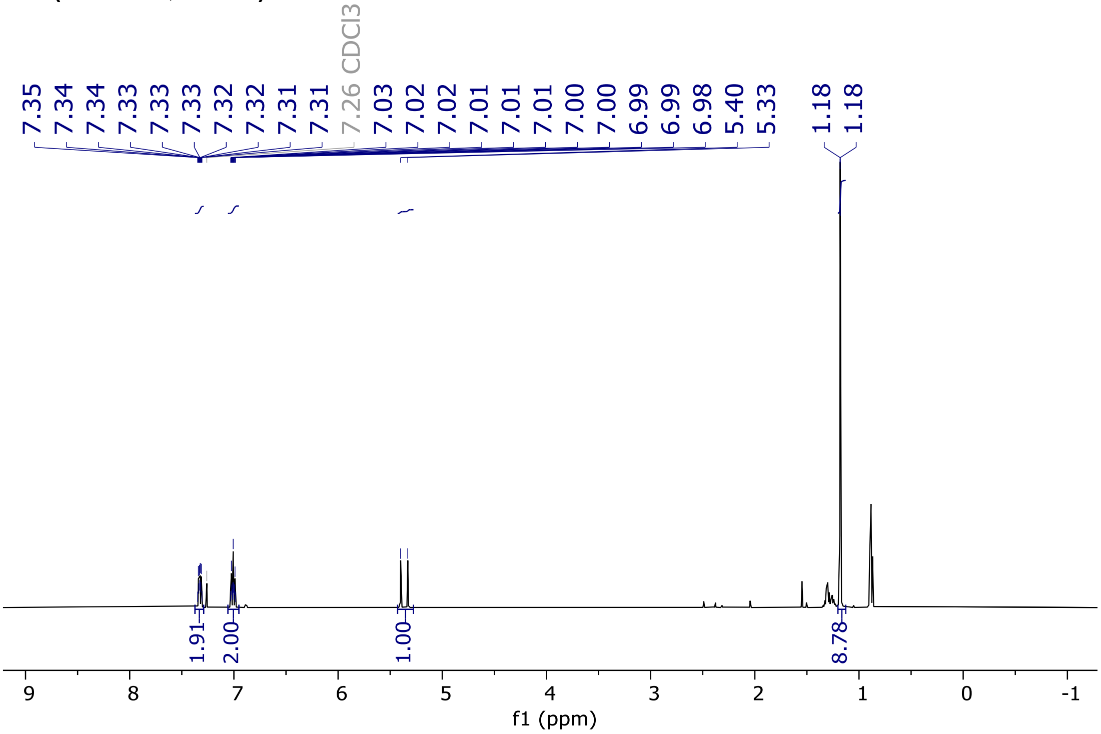

^{13}C NMR (126 MHz, CDCl$_3$):

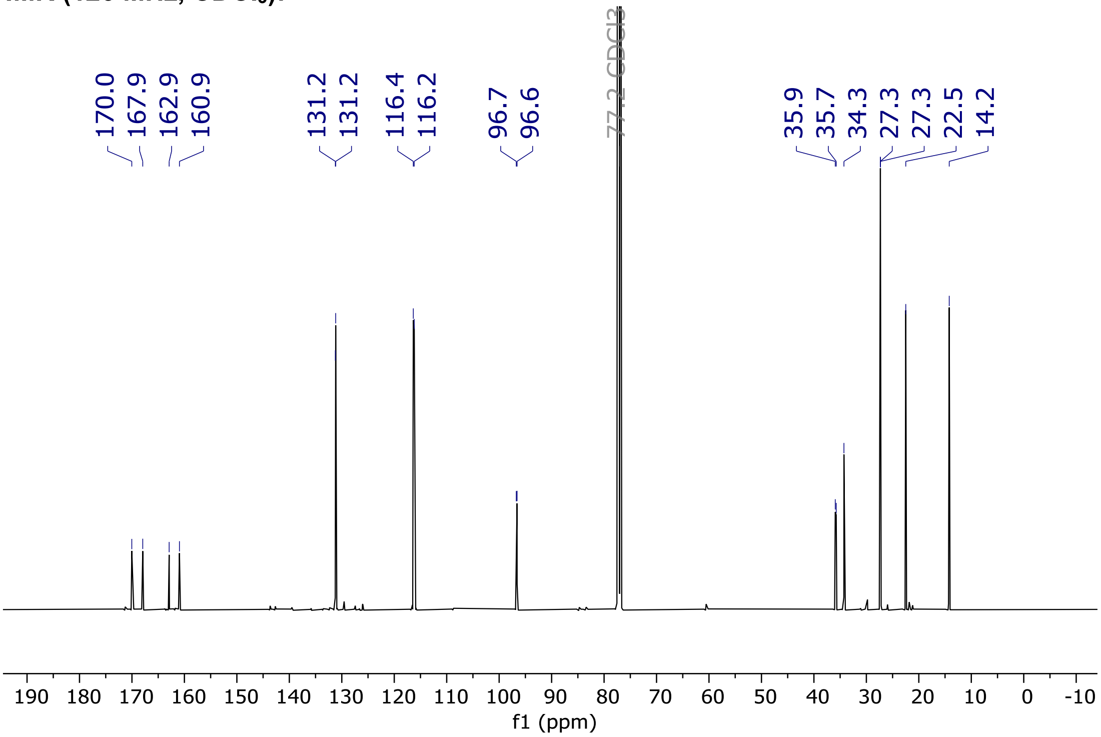

^{19}F NMR (376 MHz, $CDCl_3$):

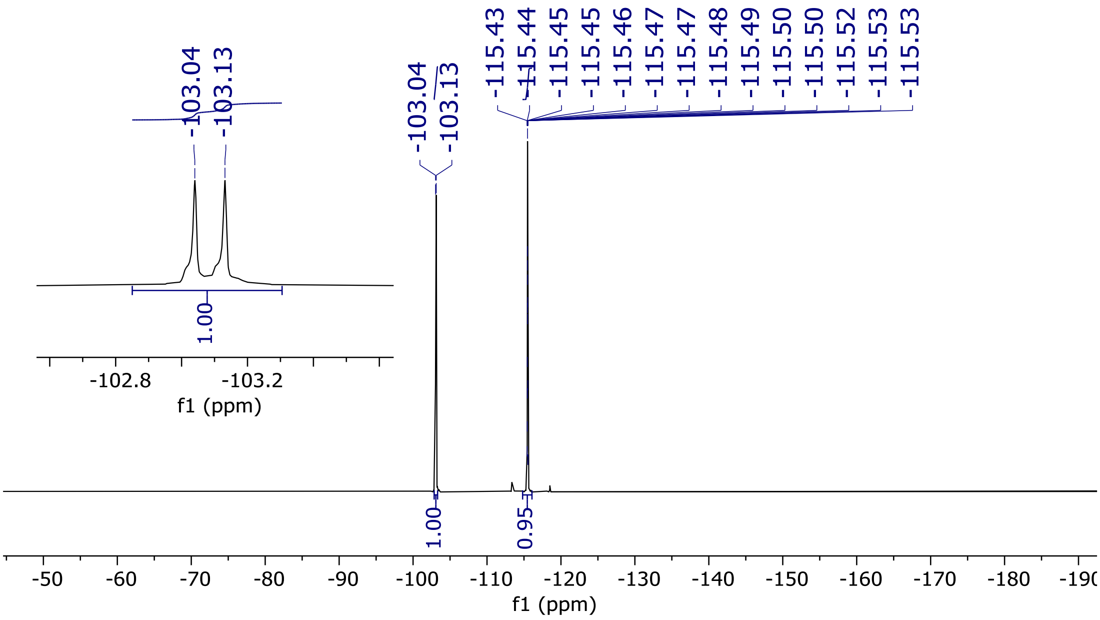

(Z)-2-(2-fluorododec-1-en-1-yl)isothiouronium BF_4

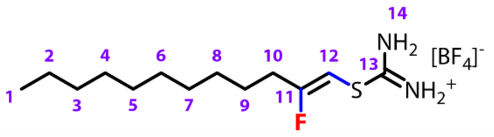

^{1}H NMR (500 MHz, $C(CD_3)_2O$):

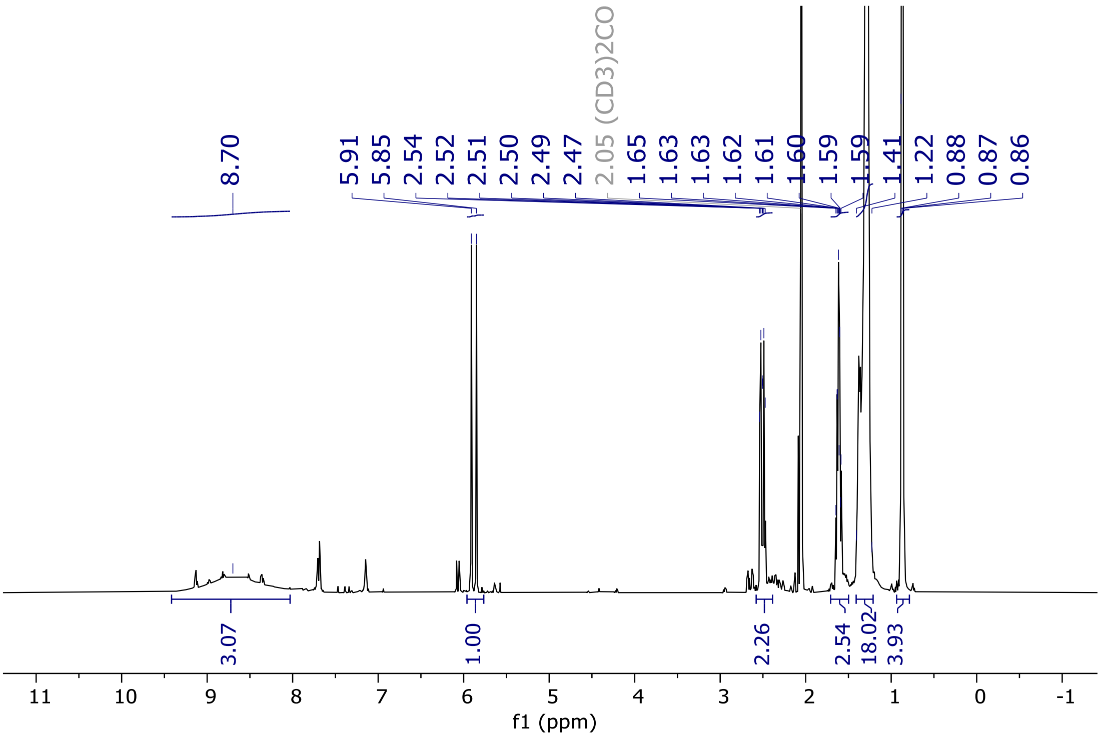

^{13}C NMR (126 MHz, $C(CD_3)_2O$):

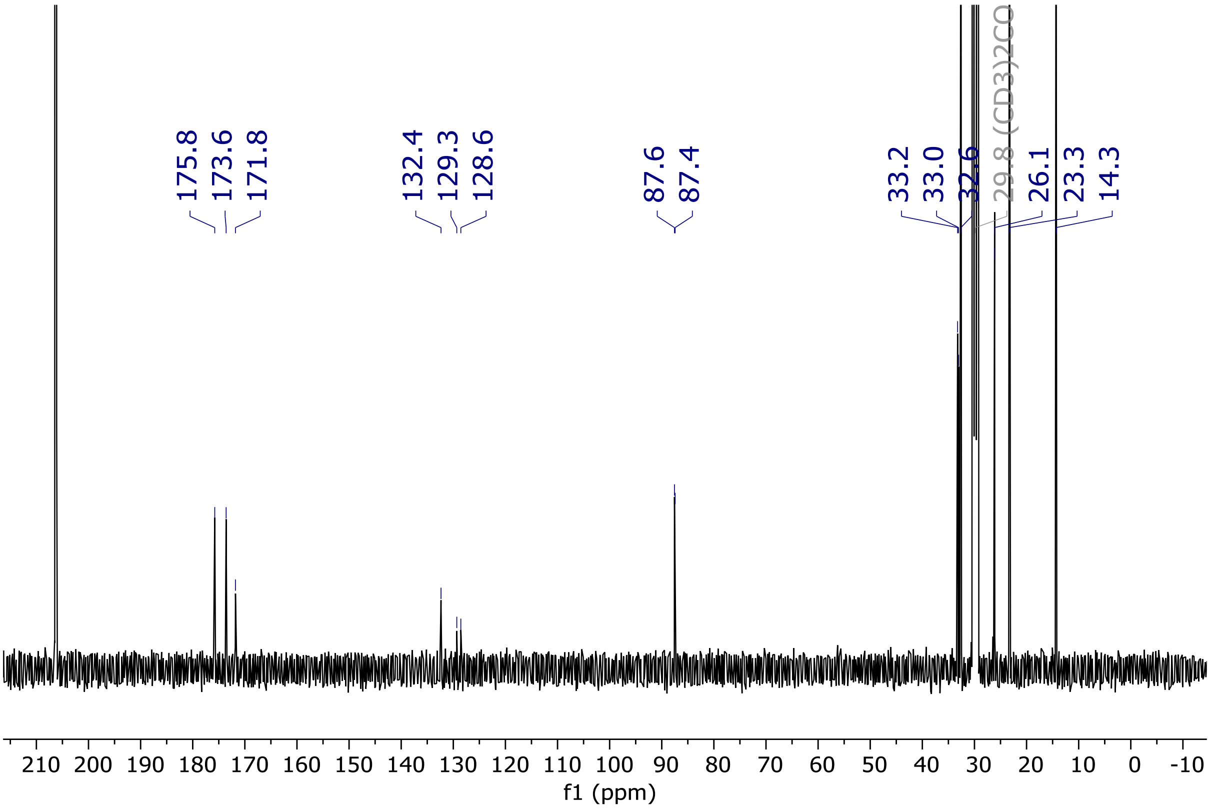

(Z)-1-chloro-4-((2-fluoro-3-iodoallyl)oxy)benzene ***(9c)***

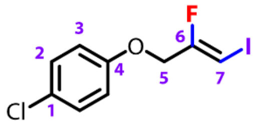

^{1}H NMR (500 MHz, $CDCl_3$):

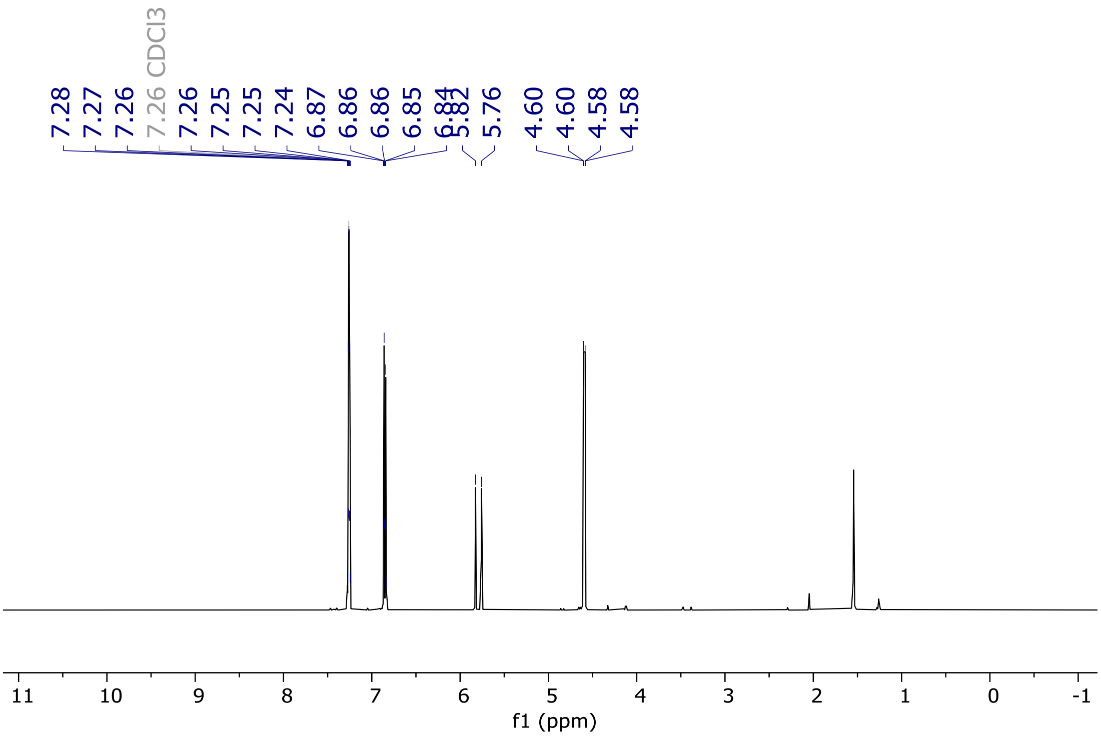

^{13}C NMR (126 MHz, $CDCl_3$):

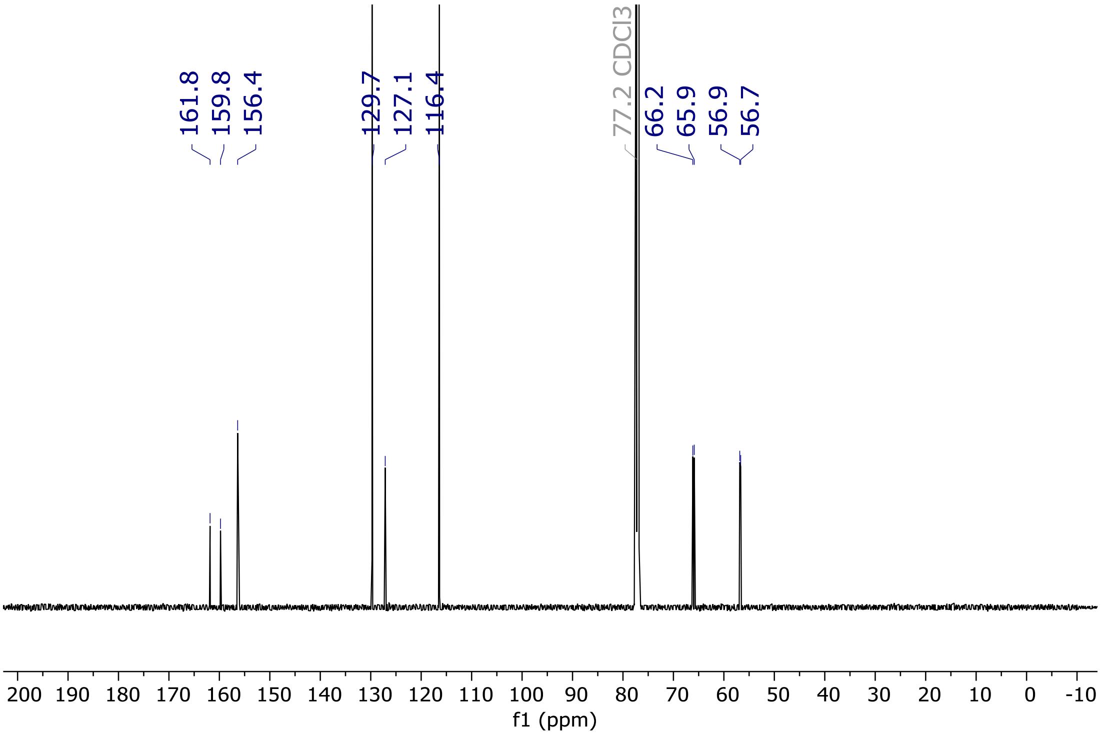

^{19}F NMR (376 MHz, CDCl$_3$):

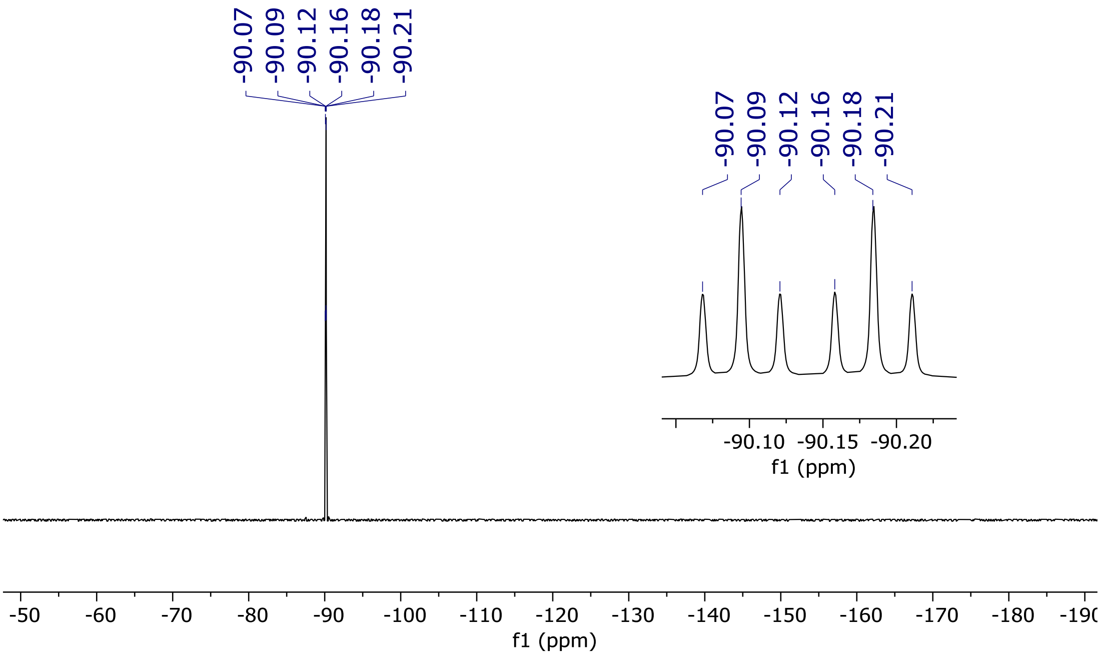

(Z)-1-((2-fluoro-3-iodoallyl)oxy)-4-nitrobenzene ***(9d)***

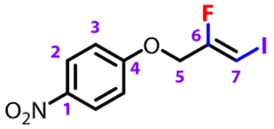

^{1}H NMR (500 MHz, $CDCl_3$):

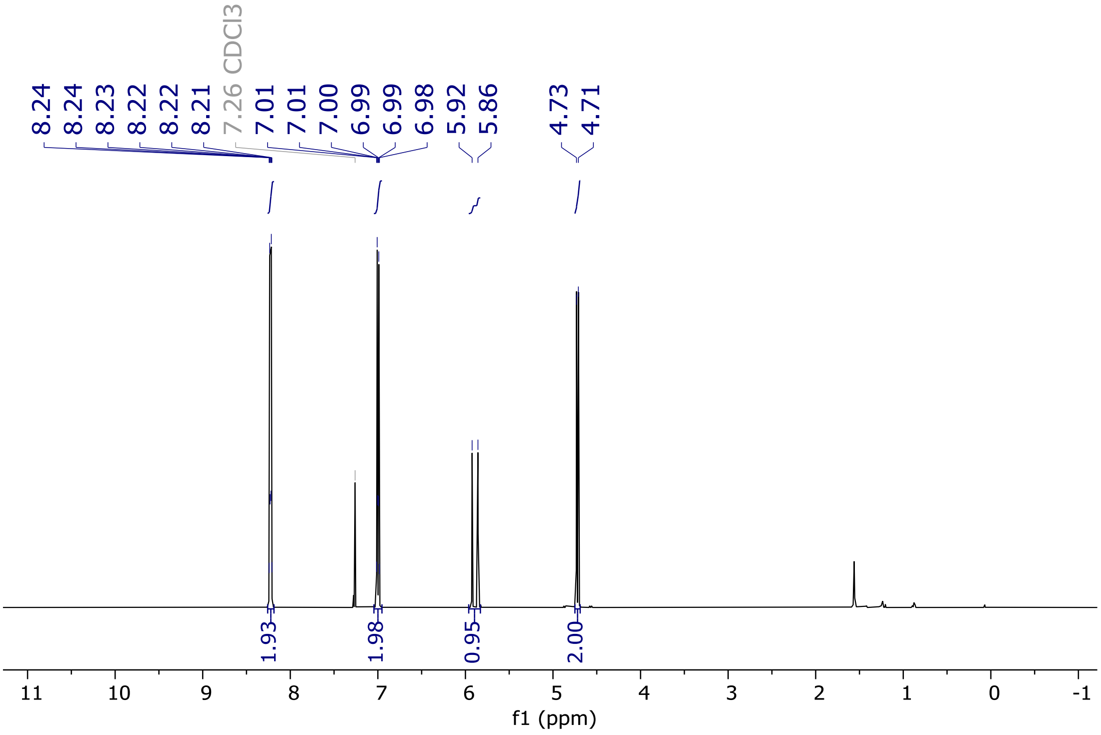

^{13}C NMR (126 MHz, $CDCl_3$):

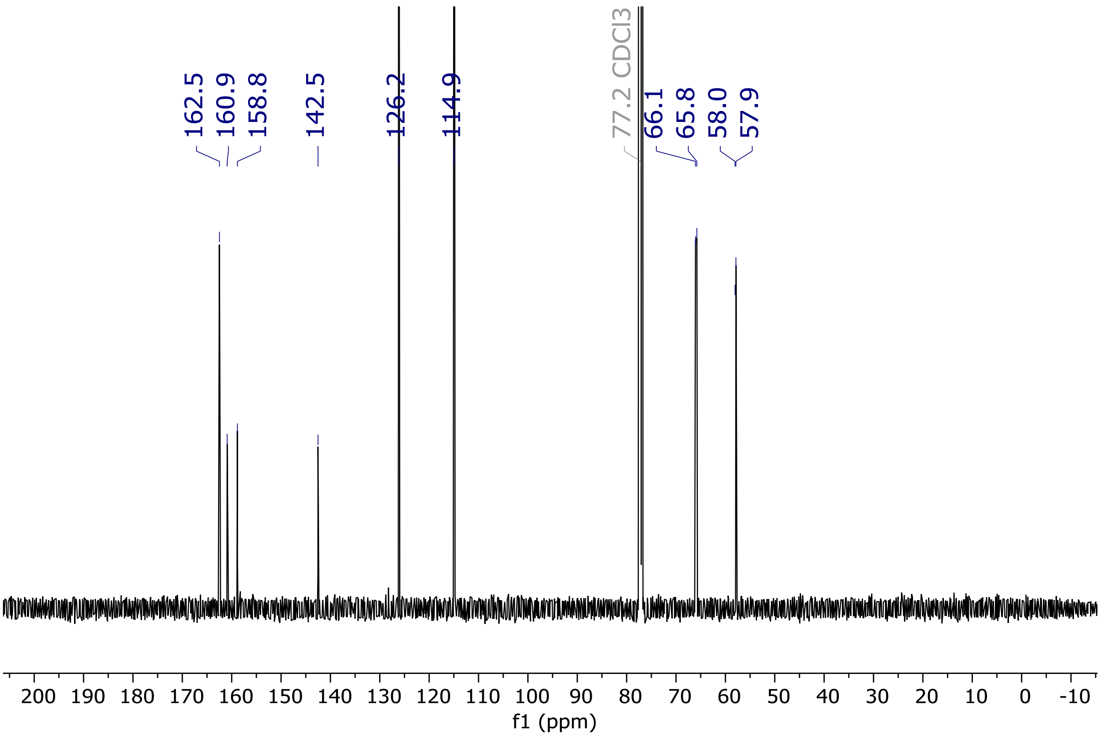

^{19}F NMR (471 MHz, $CDCl_3$):

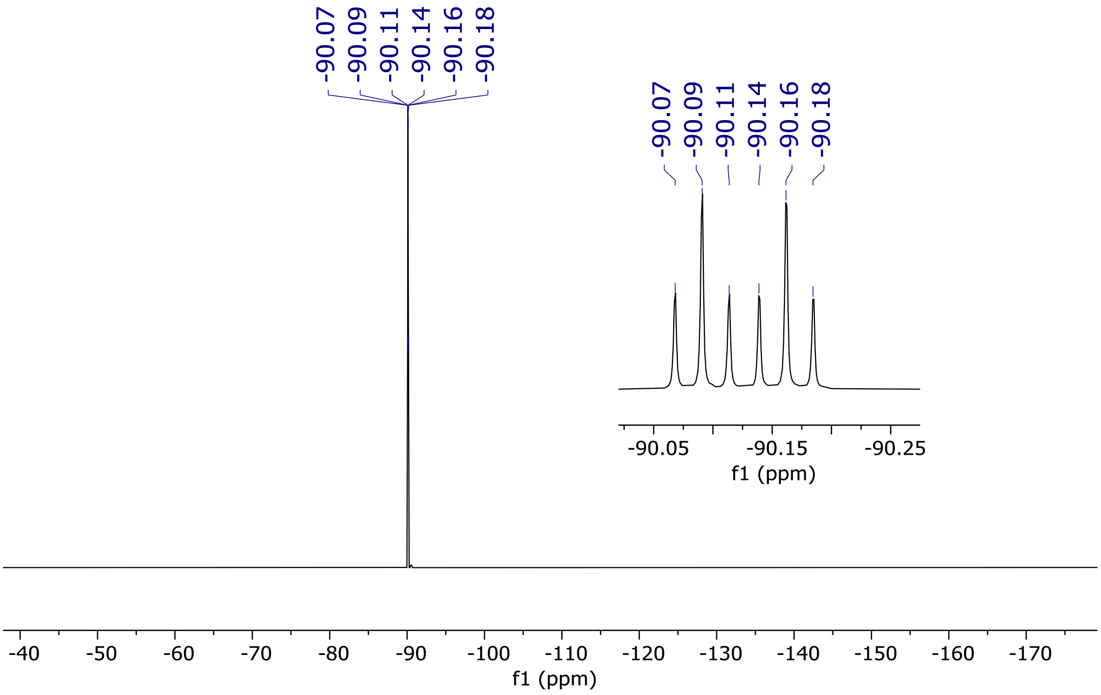

*(Z)-N-(2-fluoro-3-iodoallyl)-N-(4-fluorobenzyl)-4-methylbenzenesulfonamide **(9e)***

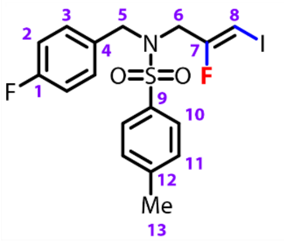

^{1}H NMR (500 MHz, $CDCl_3$):

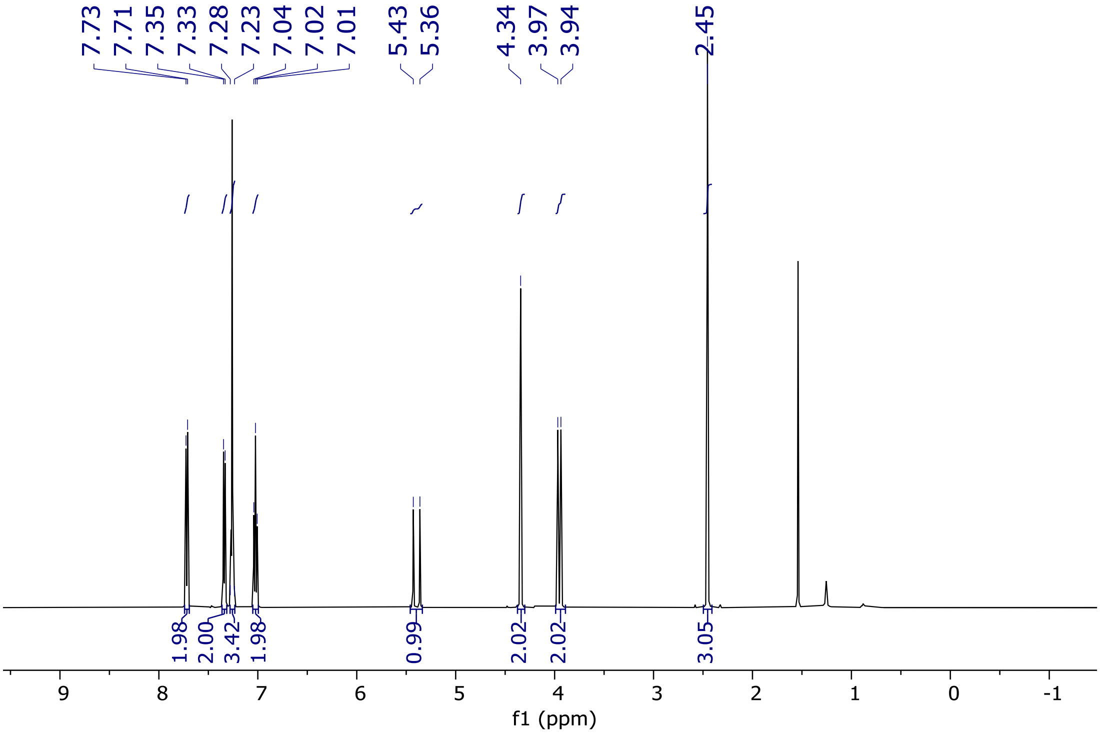

^{13}C NMR (126 MHz, $CDCl_3$):)

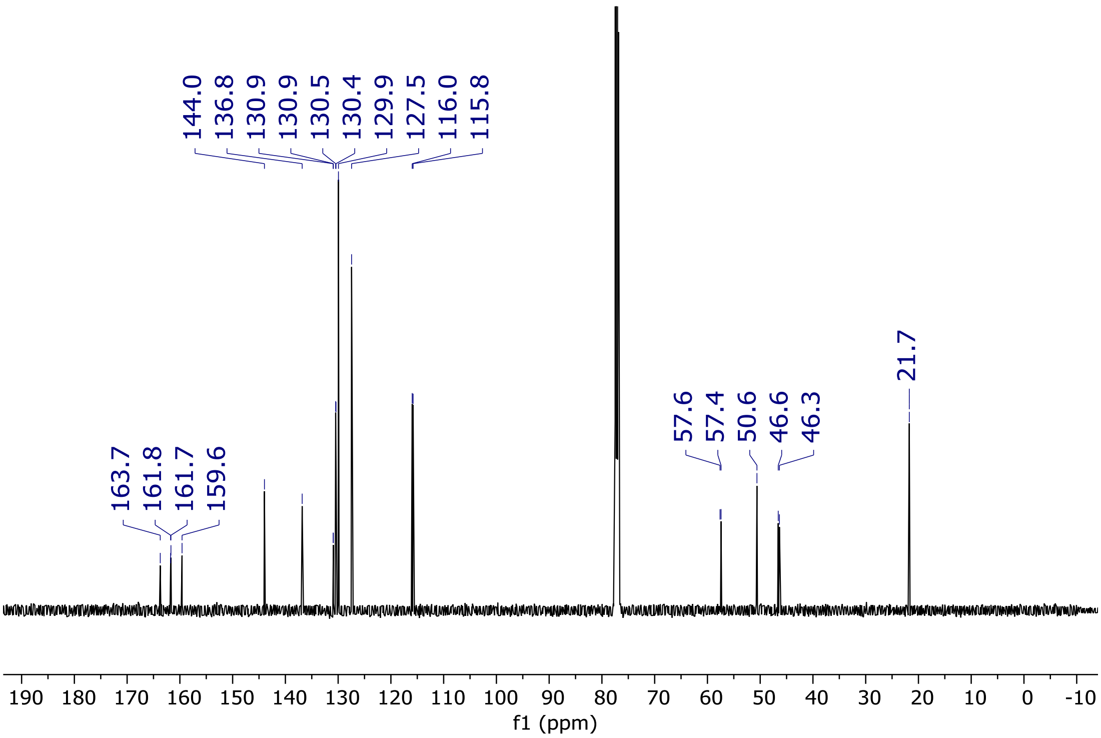

^{19}F NMR (376 MHz, CDCl$_3$):

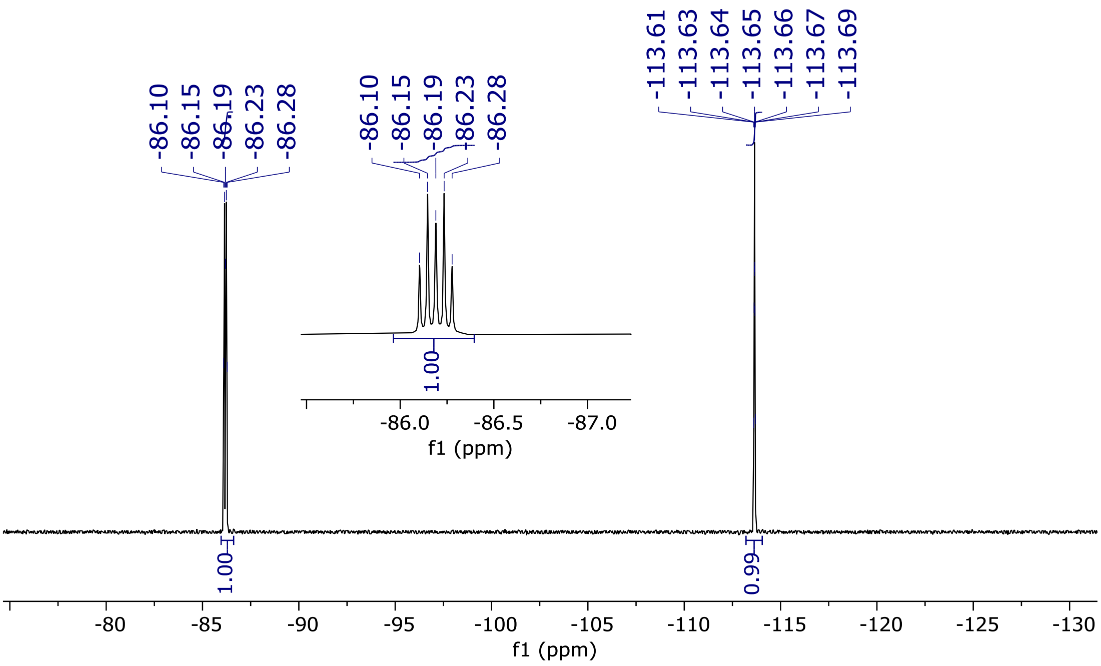

(Z)-N-(2-fluoro-3-iodoallyl)-4-methyl-N-(4-methylbenzyl)benzenesulfonamide ***(9f)***

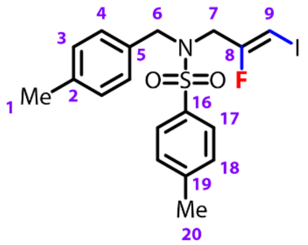

^{1}H NMR (500 MHz, CDCl₃):

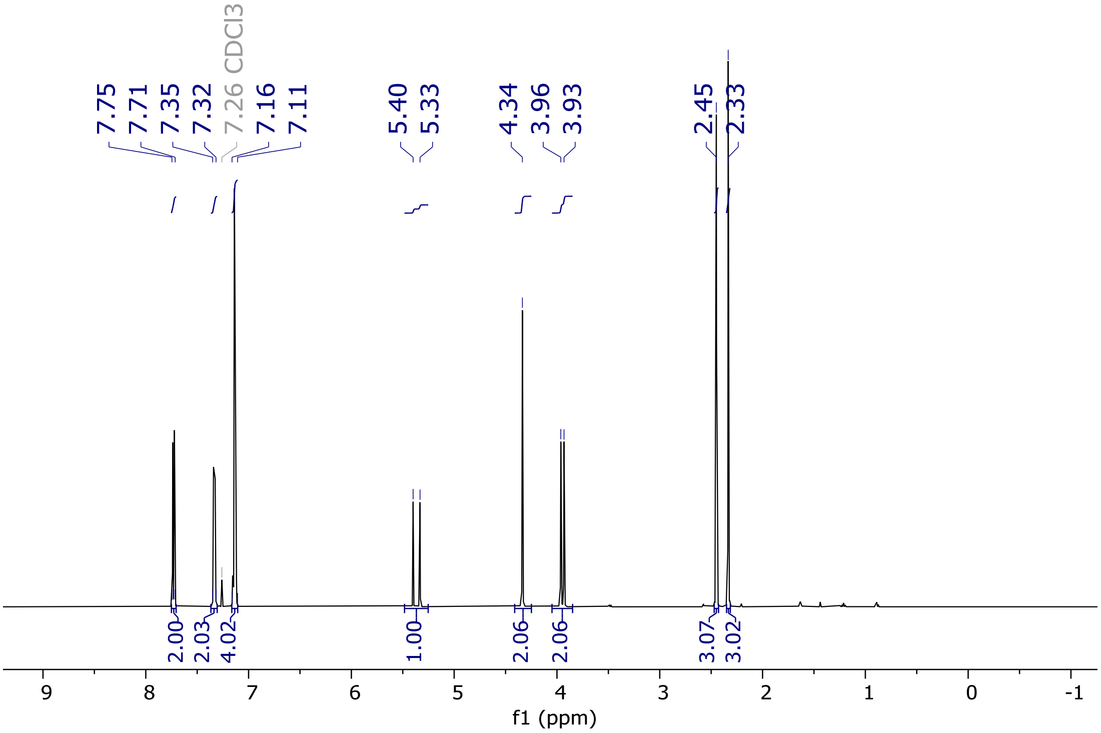

^{13}C NMR (126 MHz, CDCl₃):

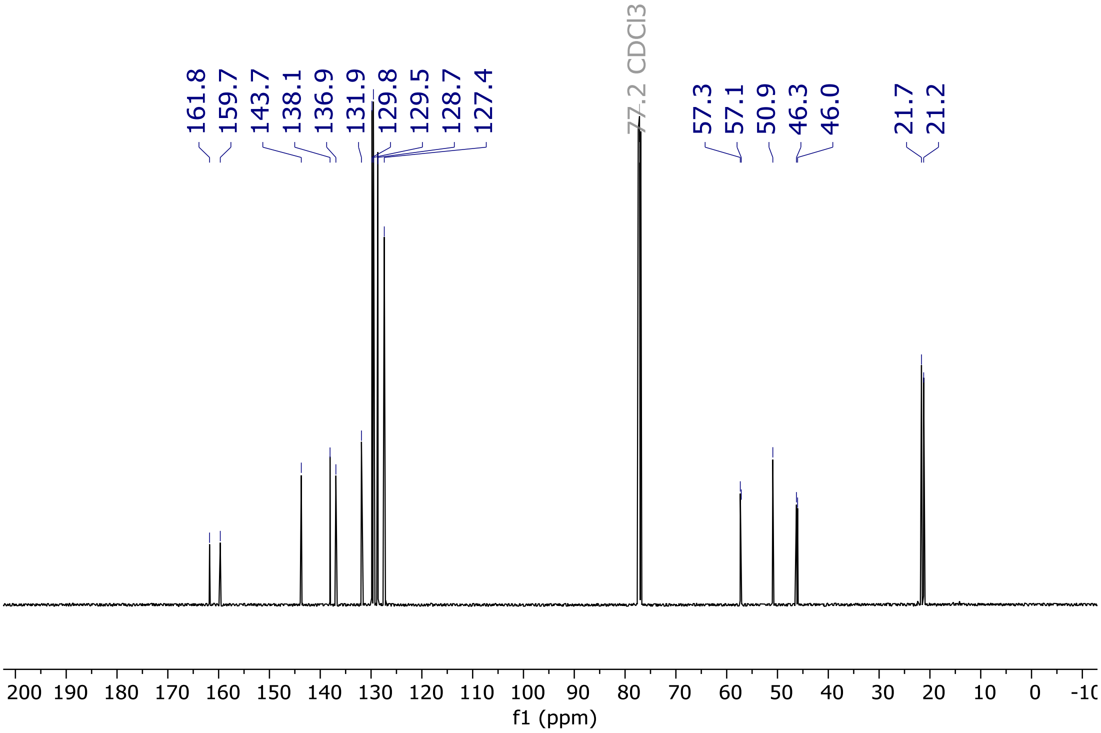

^{19}F NMR (471 MHz, $CDCl_3$):

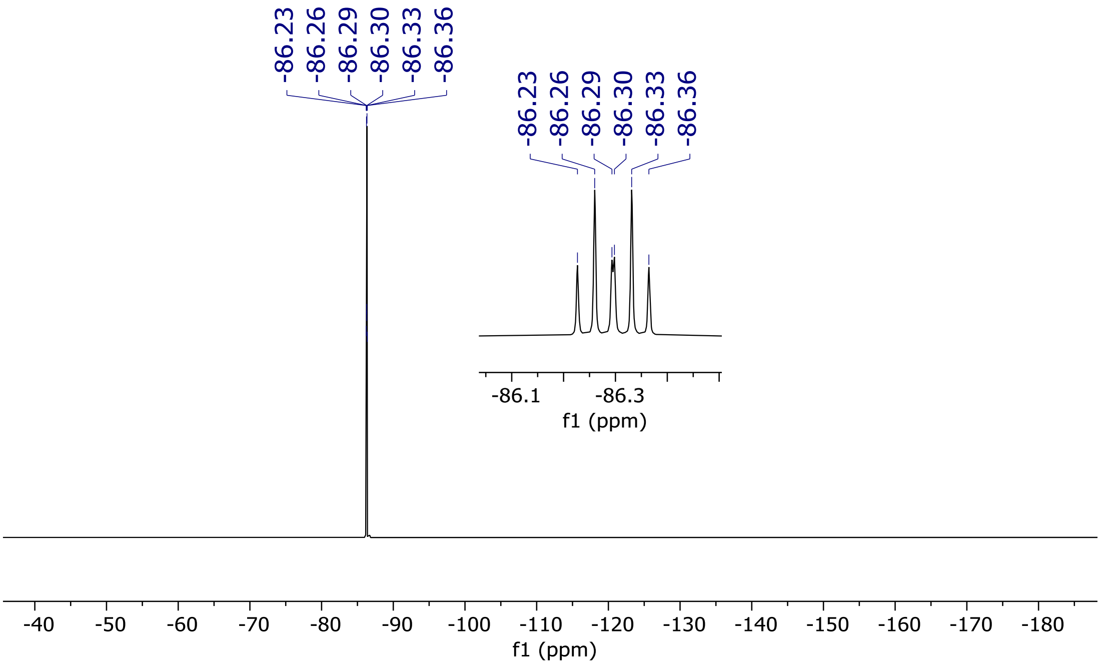

(Z)-N-(2-fluoro-3-iodoallyl)-N-(4-fluorophenyl)-4-methylbenzenesulfonamide ***(9h)***

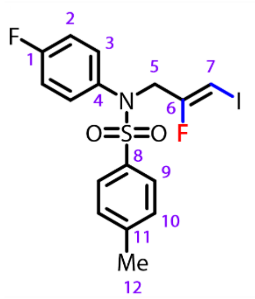

^{1}H NMR (500 MHz, CDCl$_3$):

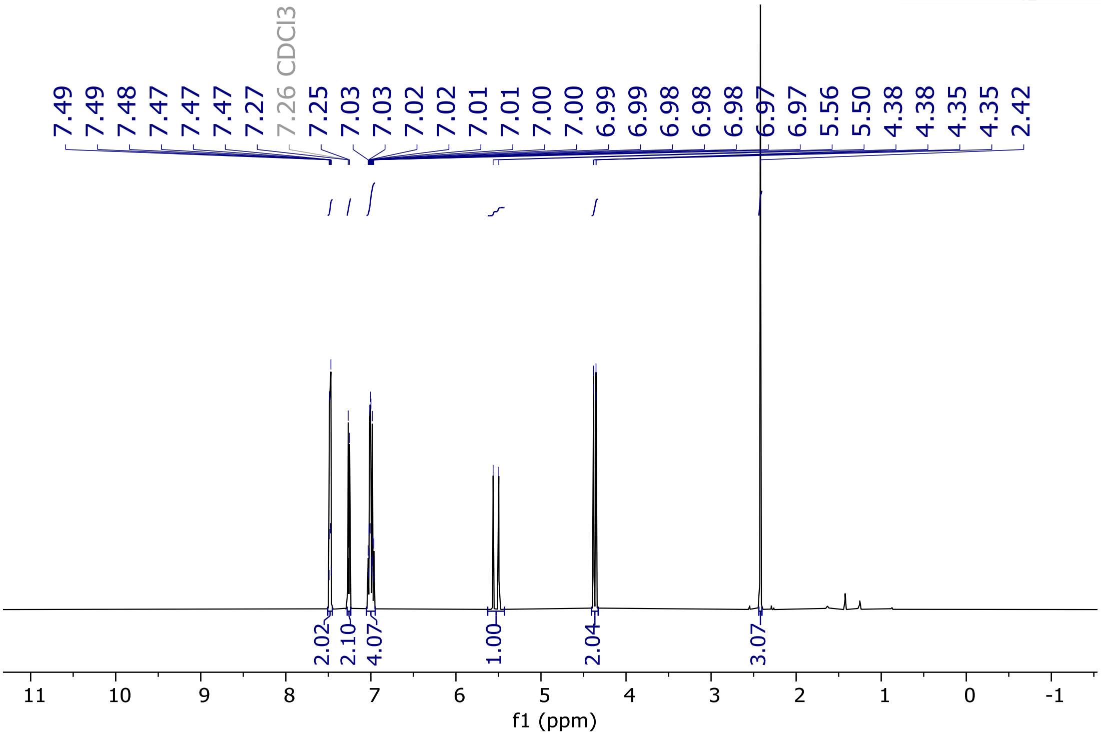

^{13}C NMR (126 MHz, CDCl$_3$):

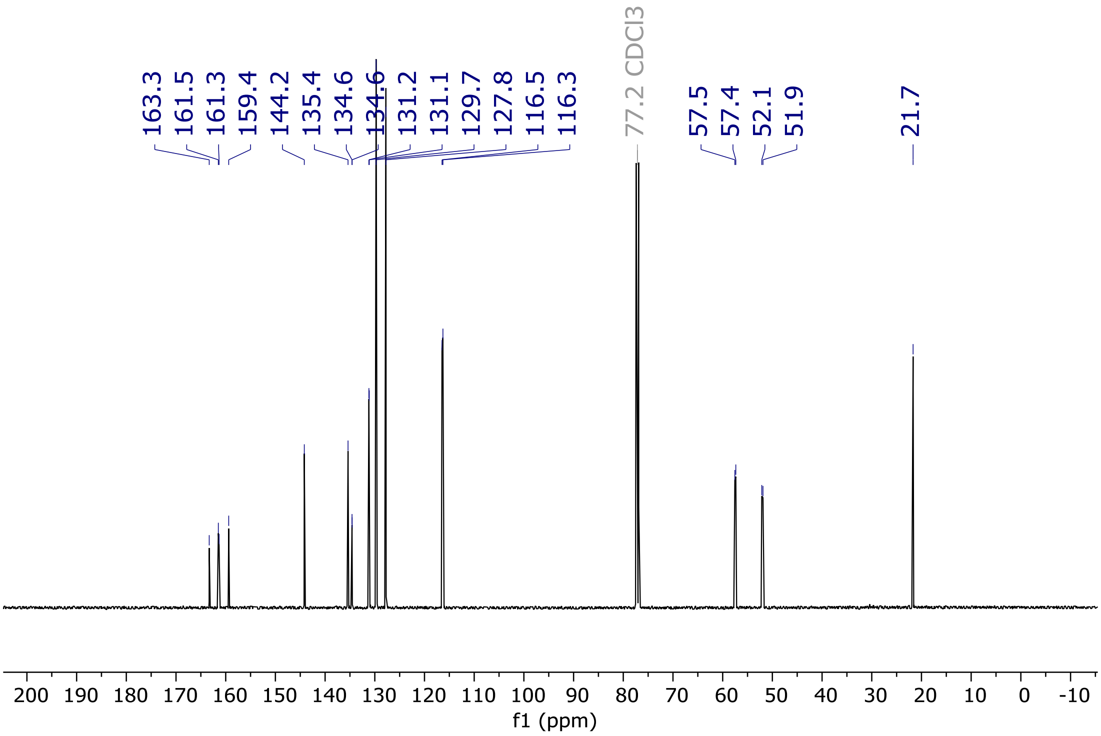

^{19}F NMR (376 MHz, $CDCl_3$):

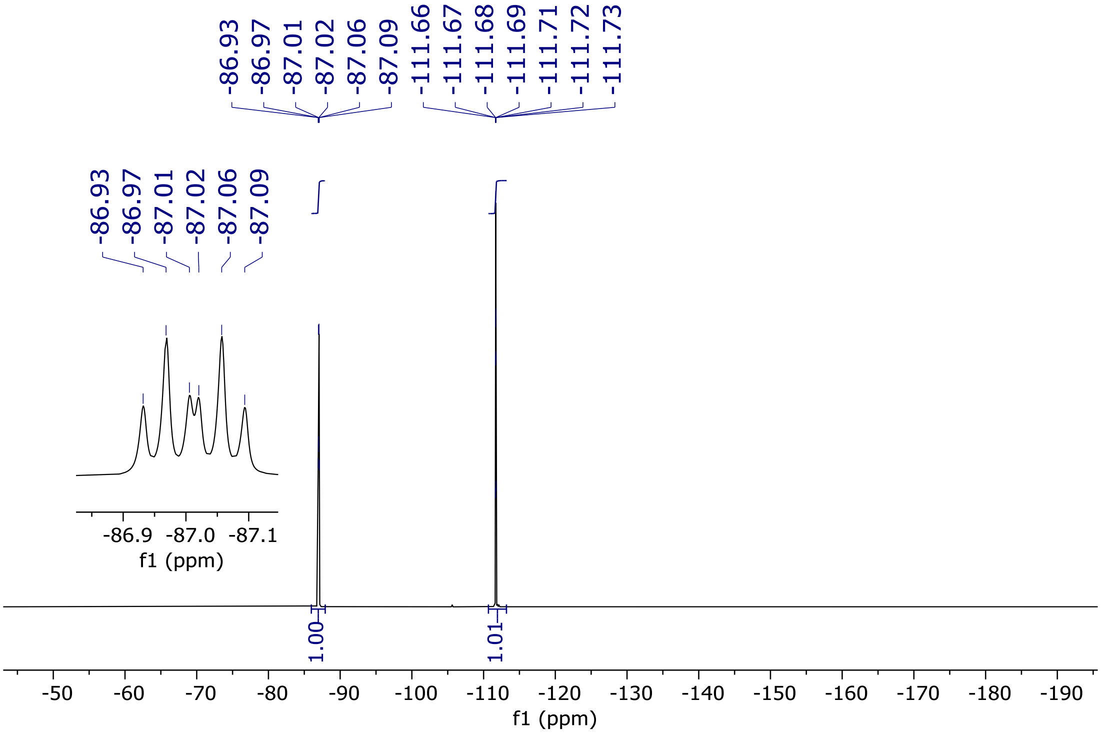

(Z)-(((3-fluoro-4-iodobut-3-en-1-yl)oxy)methyl)benzene ***(9i)***

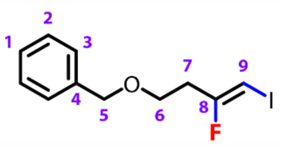

^{1}H NMR (500 MHz, CDCl$_3$):

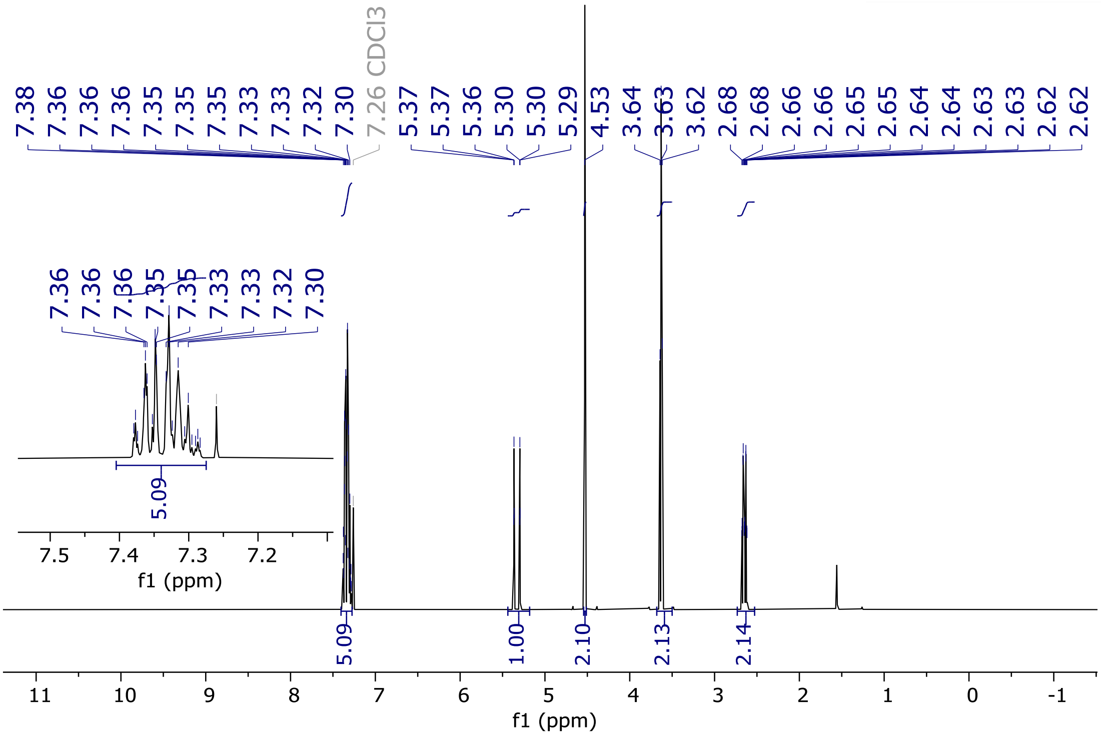

^{13}C NMR (126 MHz, CDCl$_3$):

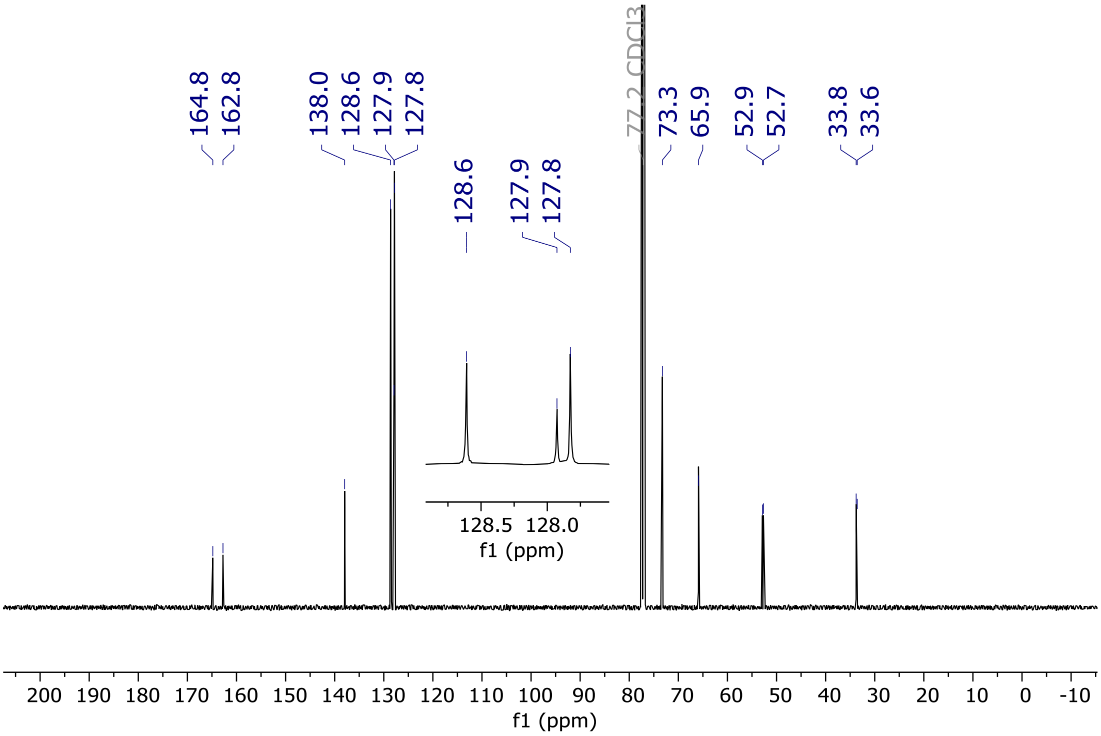

^{19}F NMR (471 MHz, $CDCl_3$):

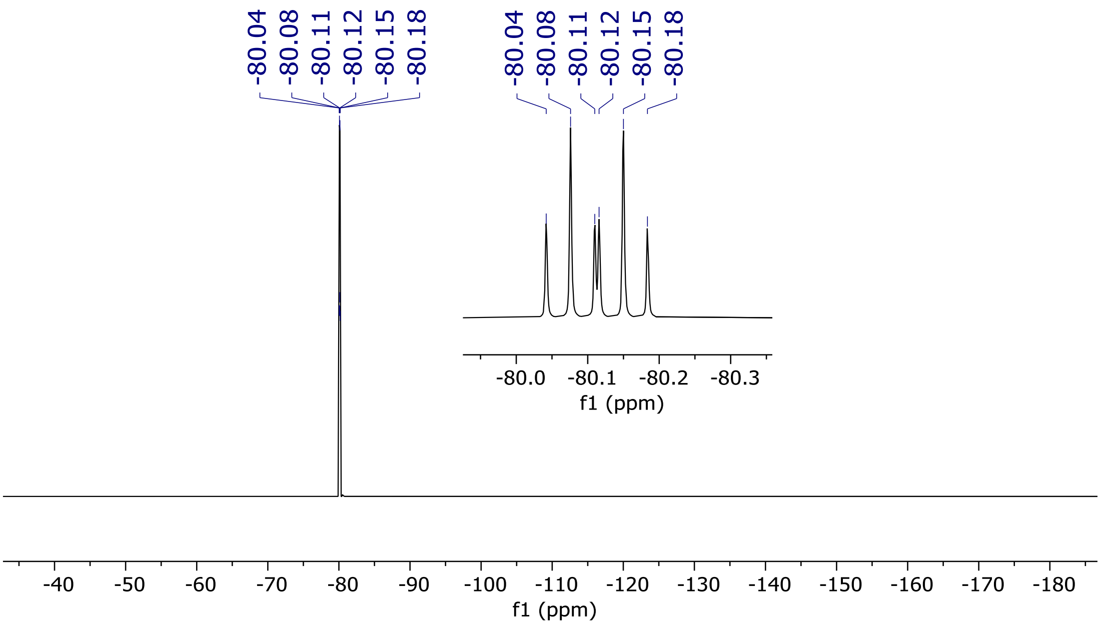

(Z)-3-fluoro-4-iodobut-3-en-1-yl 4-methylbenzenesulfonate ***(9j)***

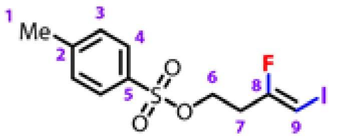

^{1}H NMR (600 MHz, CDCl$_3$):

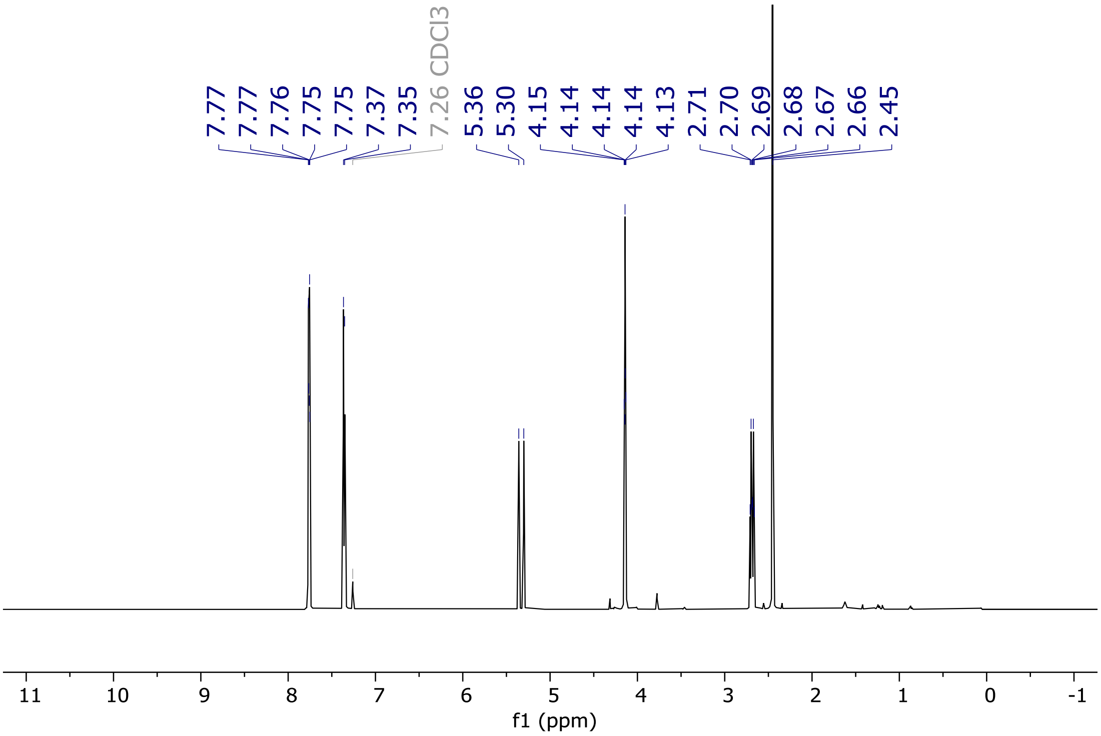

^{13}C NMR (151 MHz, CDCl$_3$):

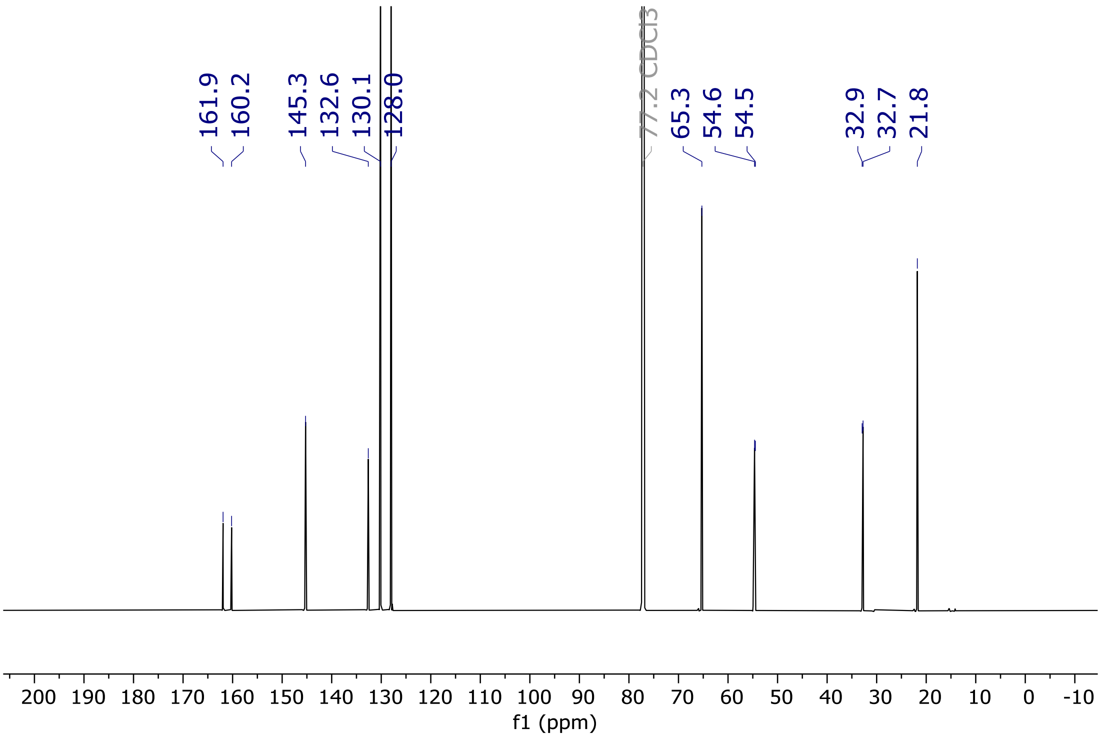

^{19}F NMR (376 MHz, $CDCl_3$):

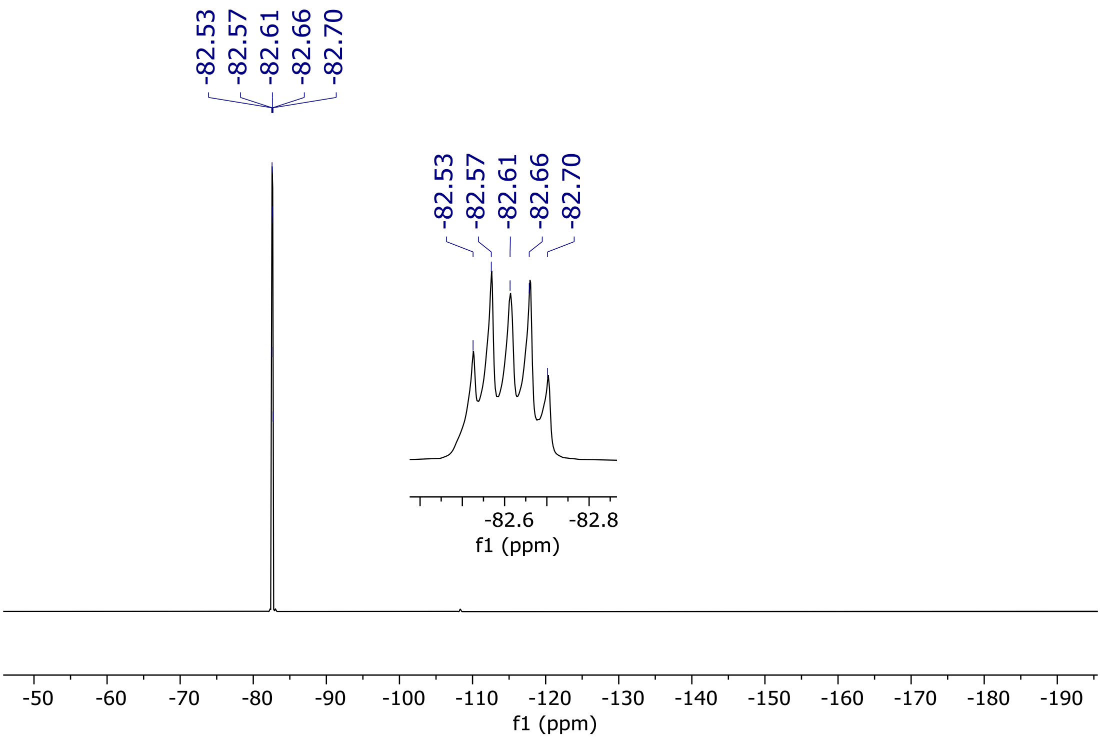

(Z)-N-(2-fluoro-3-iodoallyl)-4-methylbenzenesulfonamide ***(9n)***

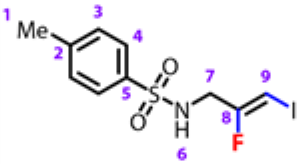

^{1}H NMR (400 MHz, $CDCl_3$):

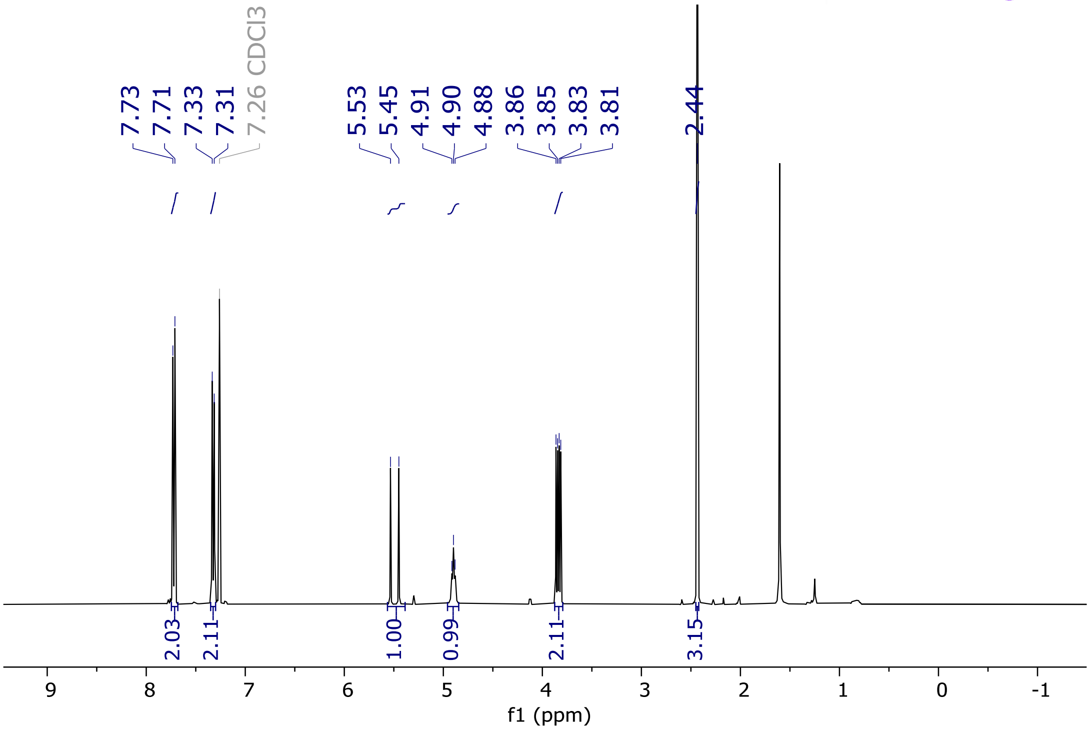

^{13}C NMR (126 MHz, $CDCl_3$):

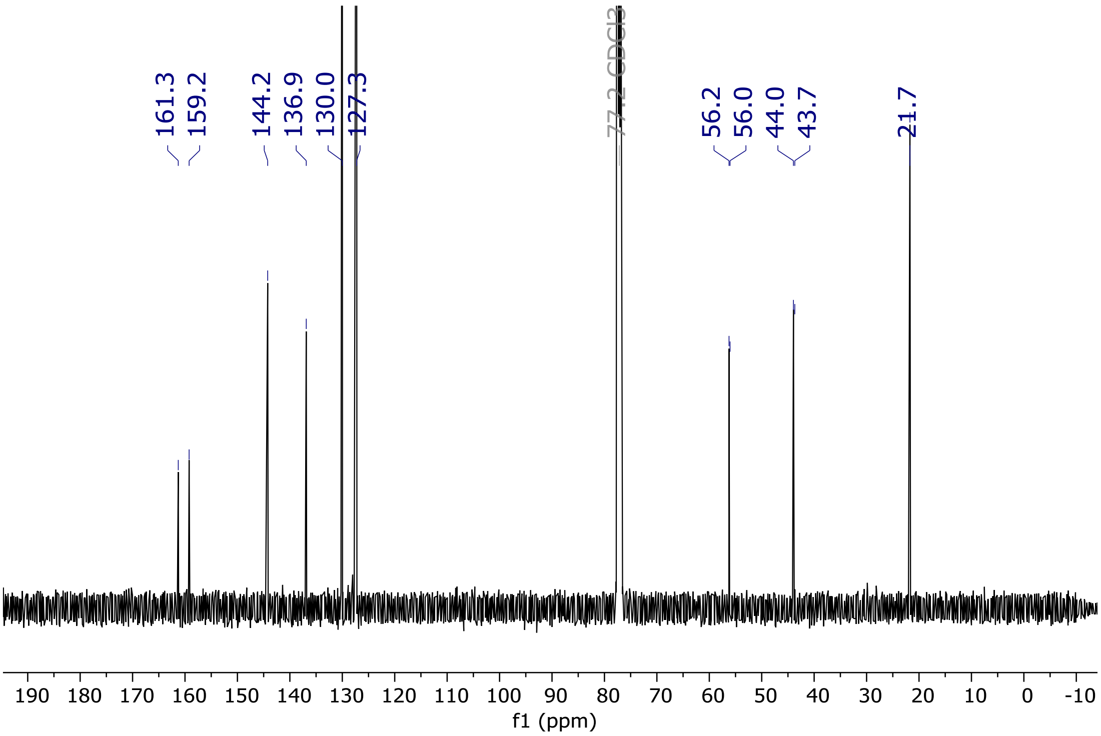

^{19}F NMR (376 MHz, $CDCl_3$):

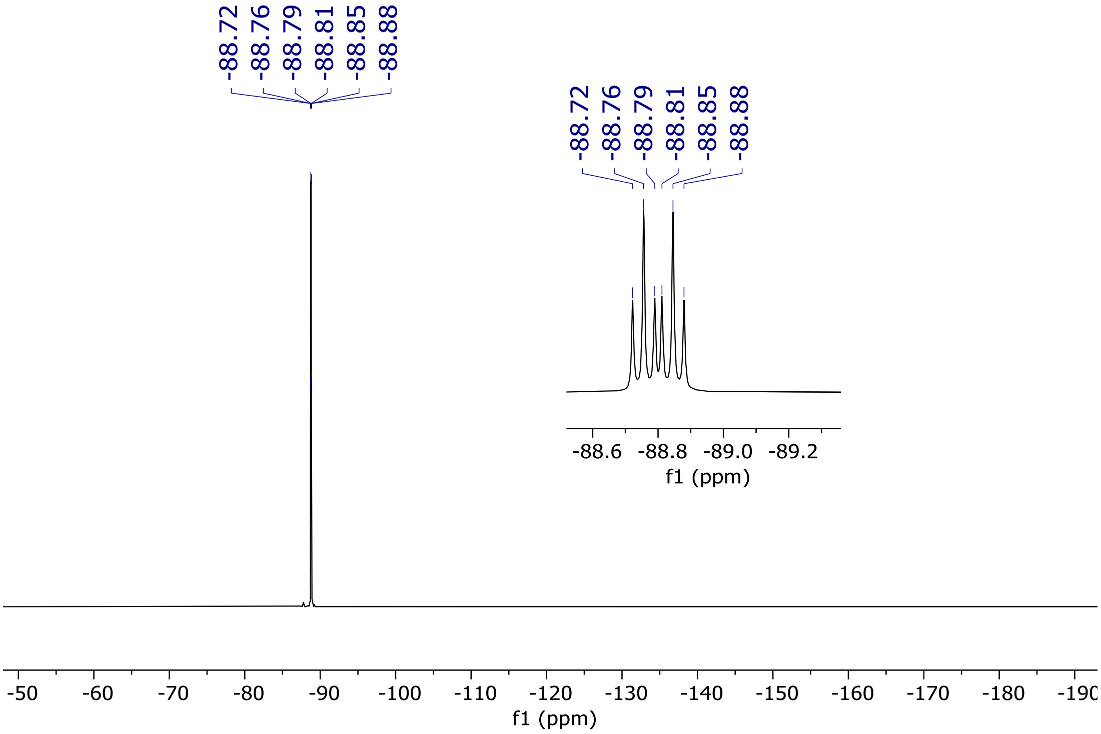

<u>(Z)-4-(1-fluoro-2-iodovinyl)benzaldehyde</u> ***<u>(9l)</u>***

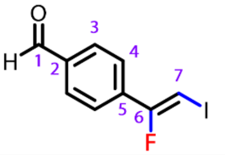

^{1}H NMR (500 MHz, CDCl$_3$):

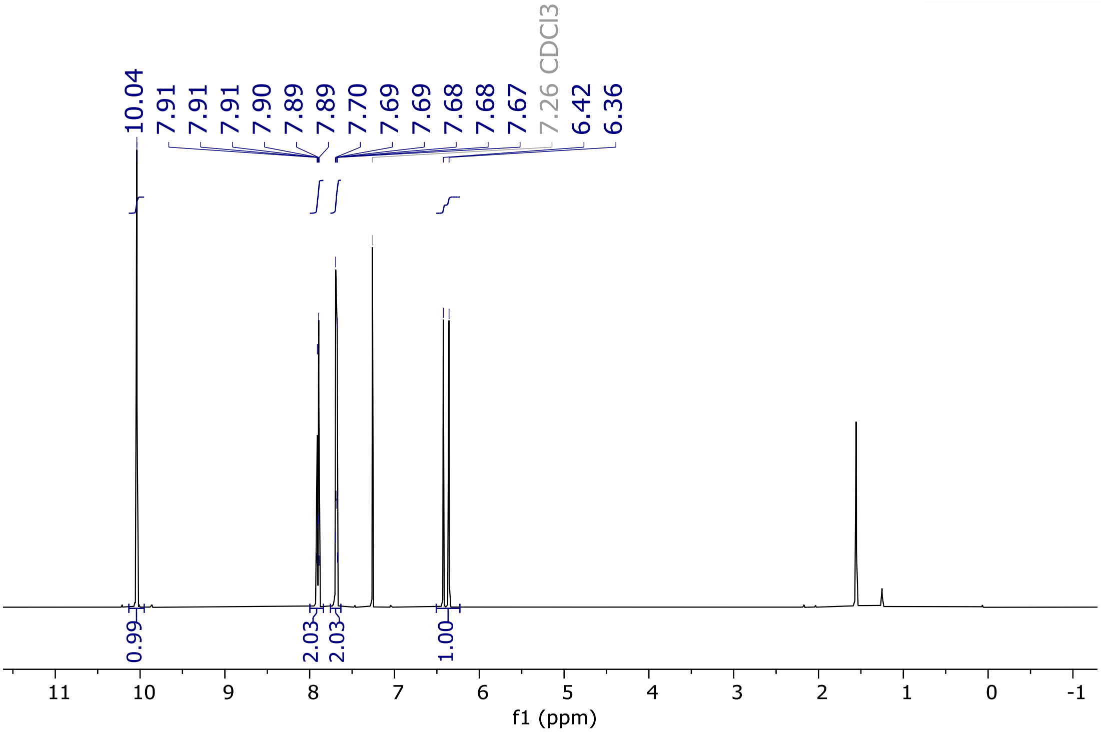

^{13}C NMR (126 MHz, CDCl$_3$):

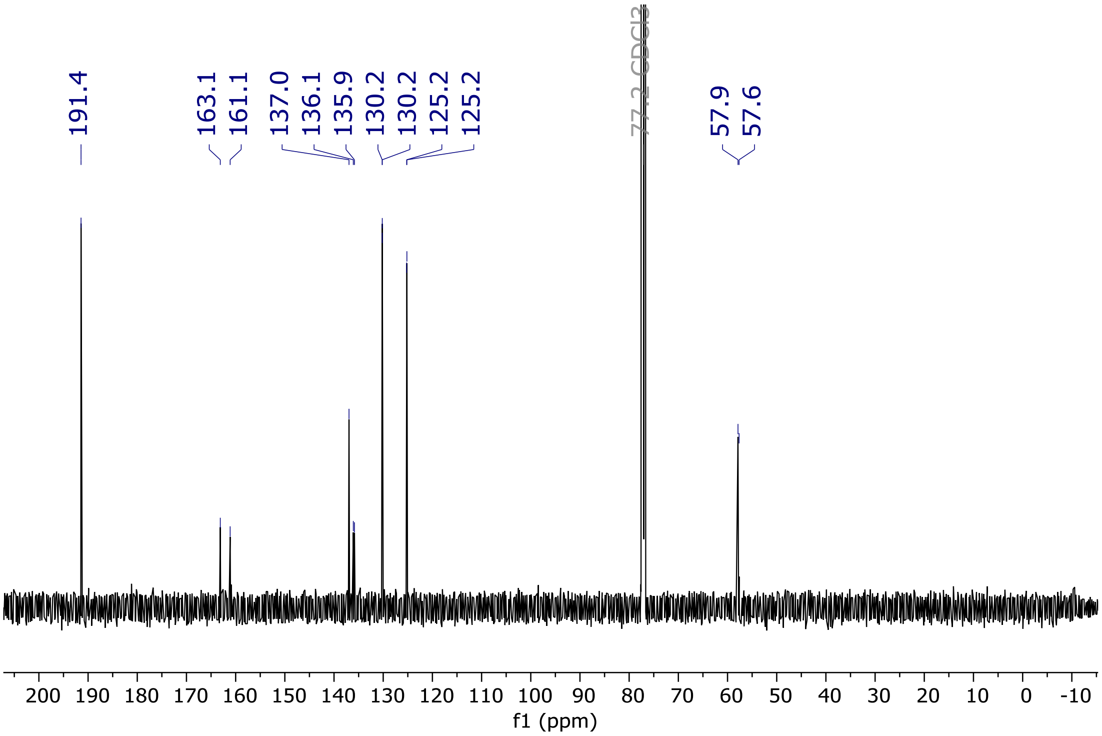

^{19}F NMR (376 MHz, $CDCl_3$):

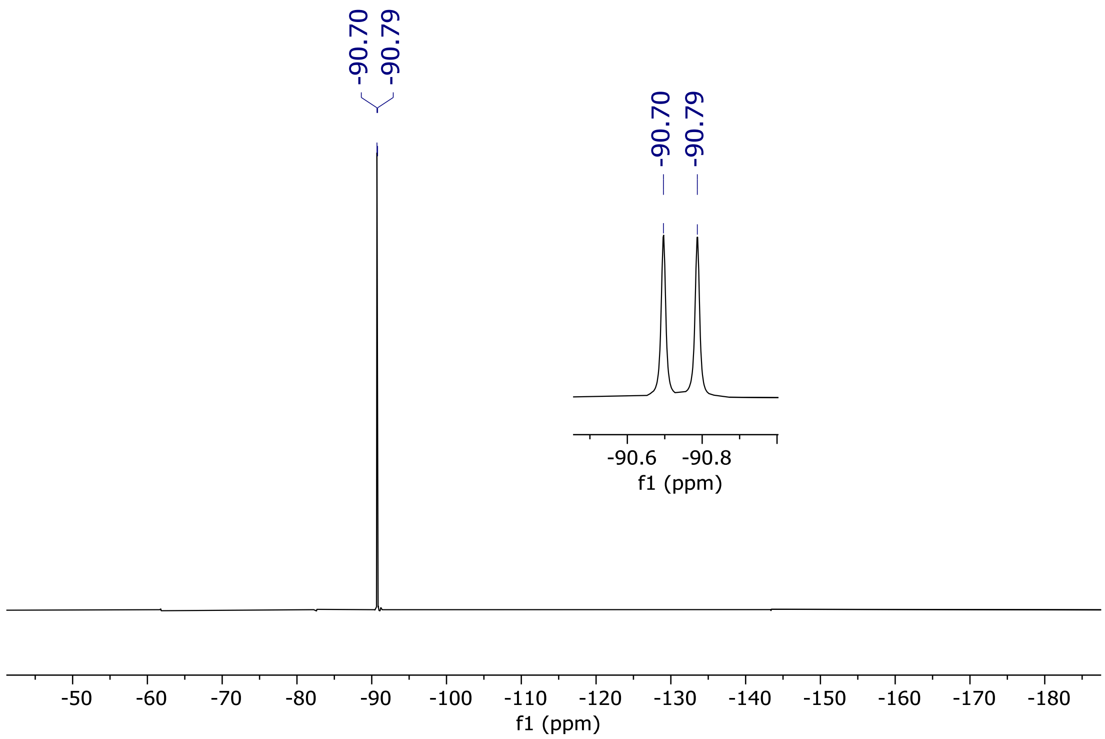

(Z)-2-(2-fluoro-3-iodoallyl)isoindoline-1,3-dione ***(9m)***

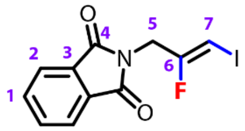

^{1}H NMR (400 MHz, $CDCl_3$):

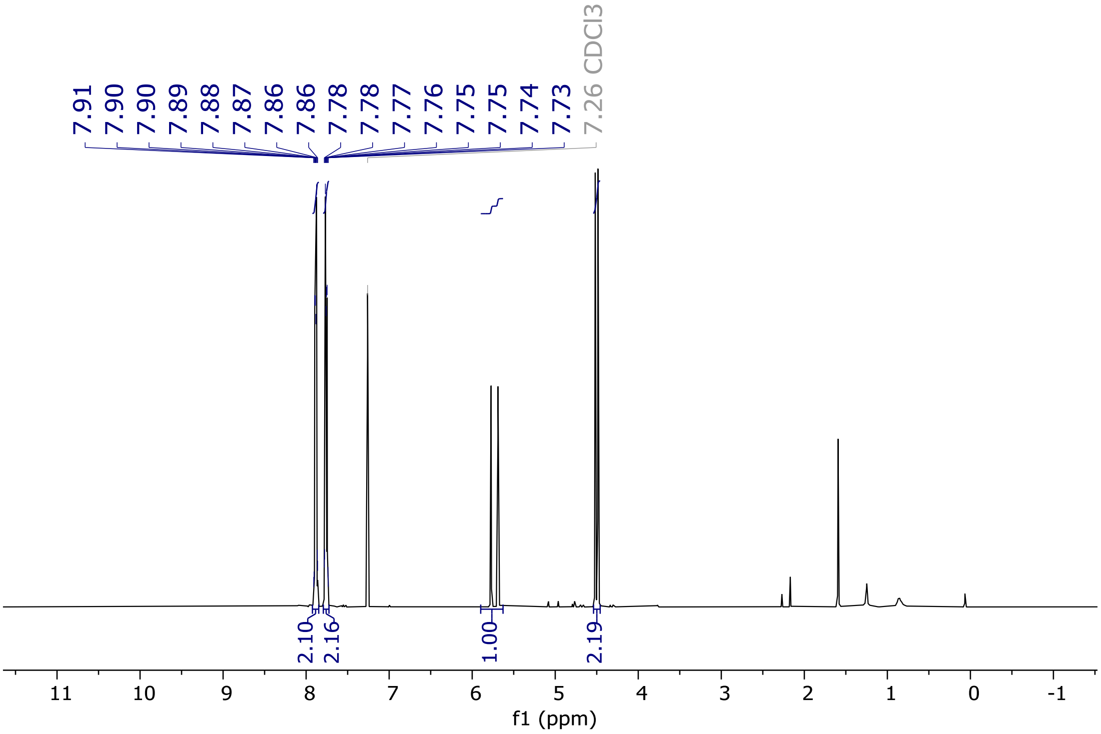

^{13}C NMR (126 MHz, $C(CD_3)_2O$):

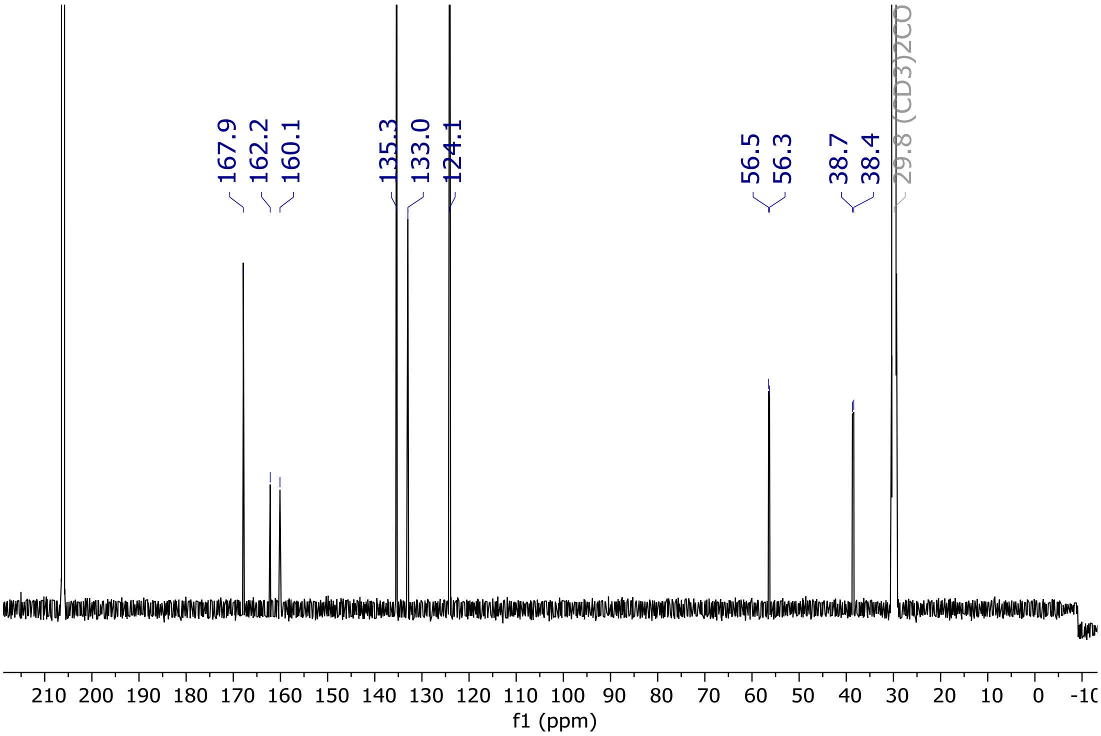

^{19}F NMR (376 MHz, $C(CD_3)_2O$):

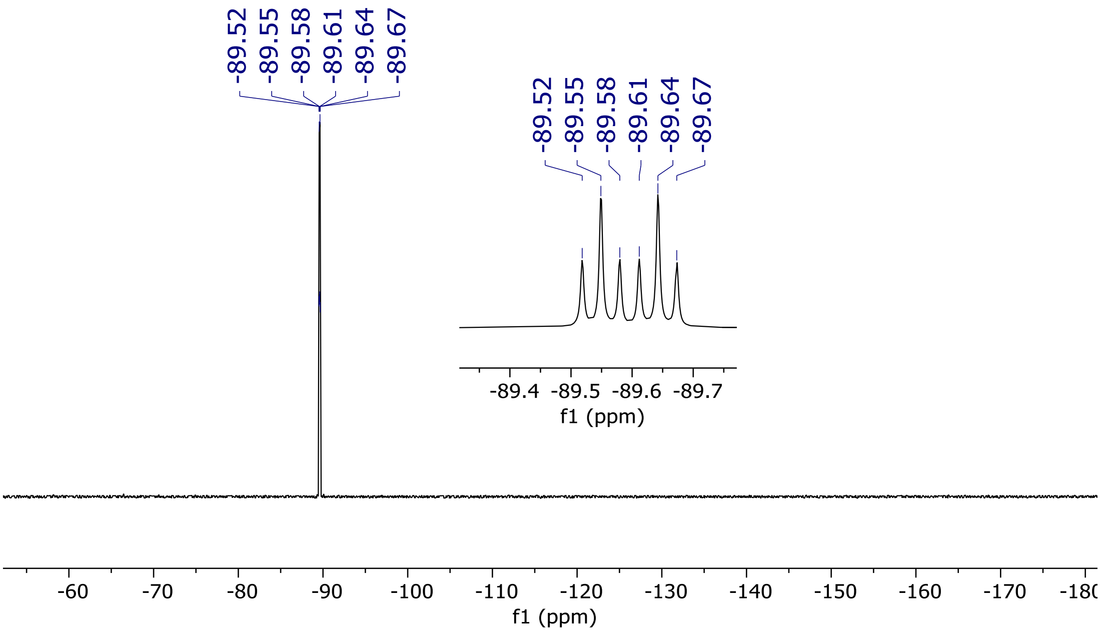

(Z)-1,3,5-trichloro-2-((2-fluoro-3-iodoallyl)oxy)benzene ***(9n)***

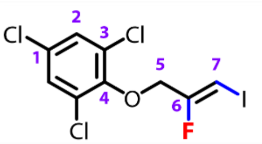

^{1}H NMR (500 MHz, $CDCl_3$):

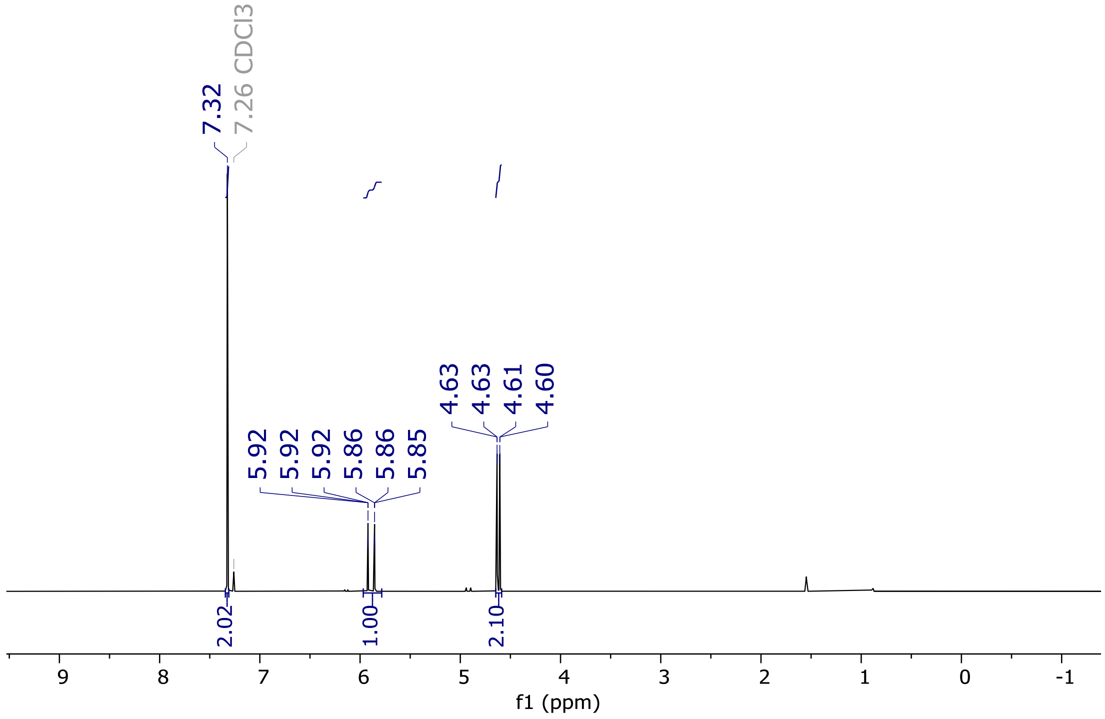

^{13}C NMR (126 MHz, $CDCl_3$):

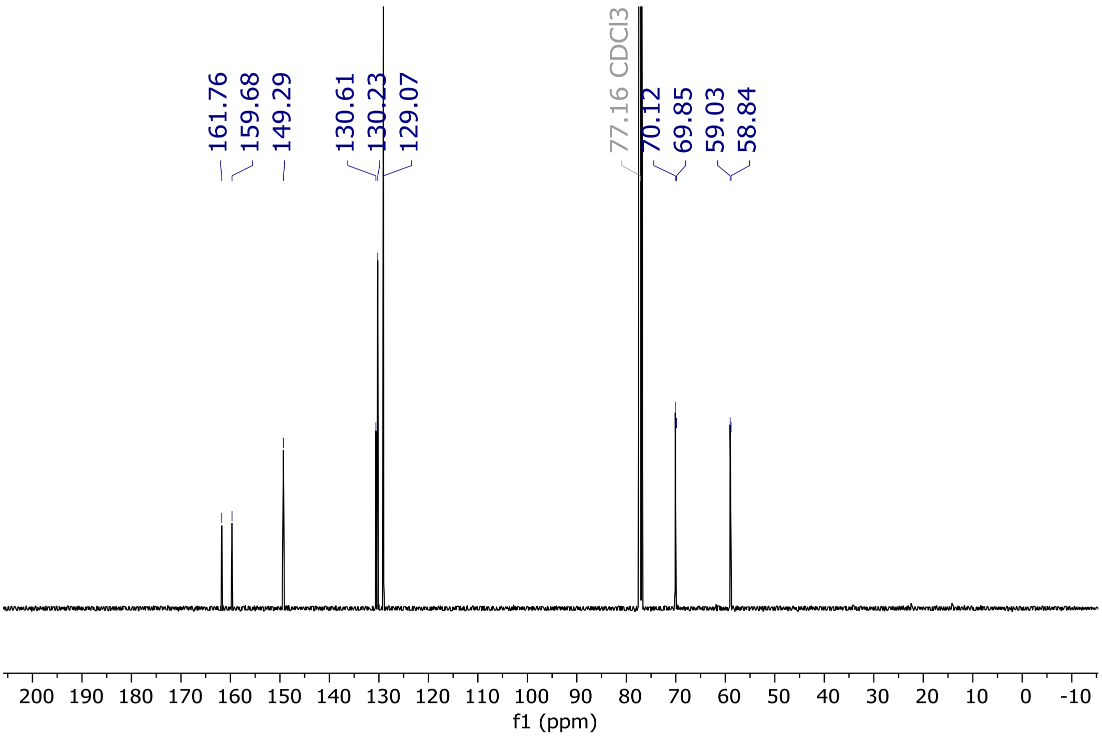

^{19}F NMR (471 MHz, $CDCl_3$):

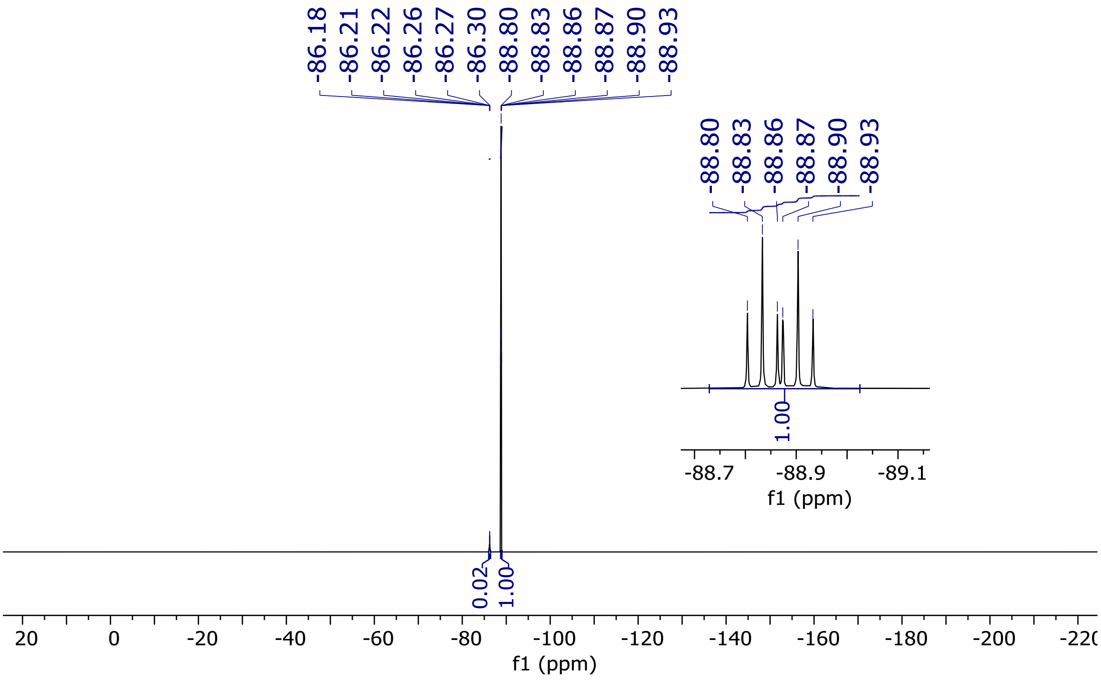

*(Z)-1,3-dibromo-5-((2-fluoro-3-iodoallyl)oxy)benzene **(9o)***

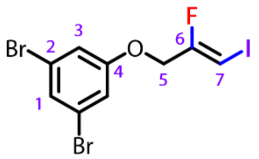

^{1}H NMR (500 MHz, $CDCl_3$):

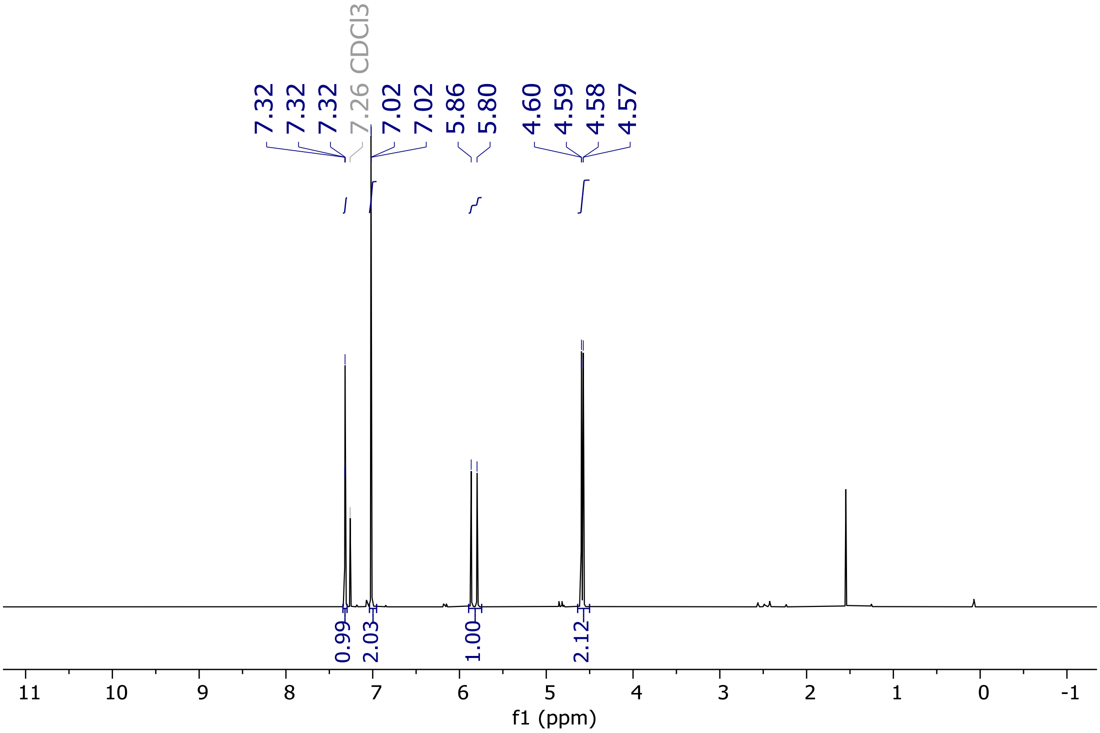

^{13}C NMR (126 MHz, $CDCl_3$):

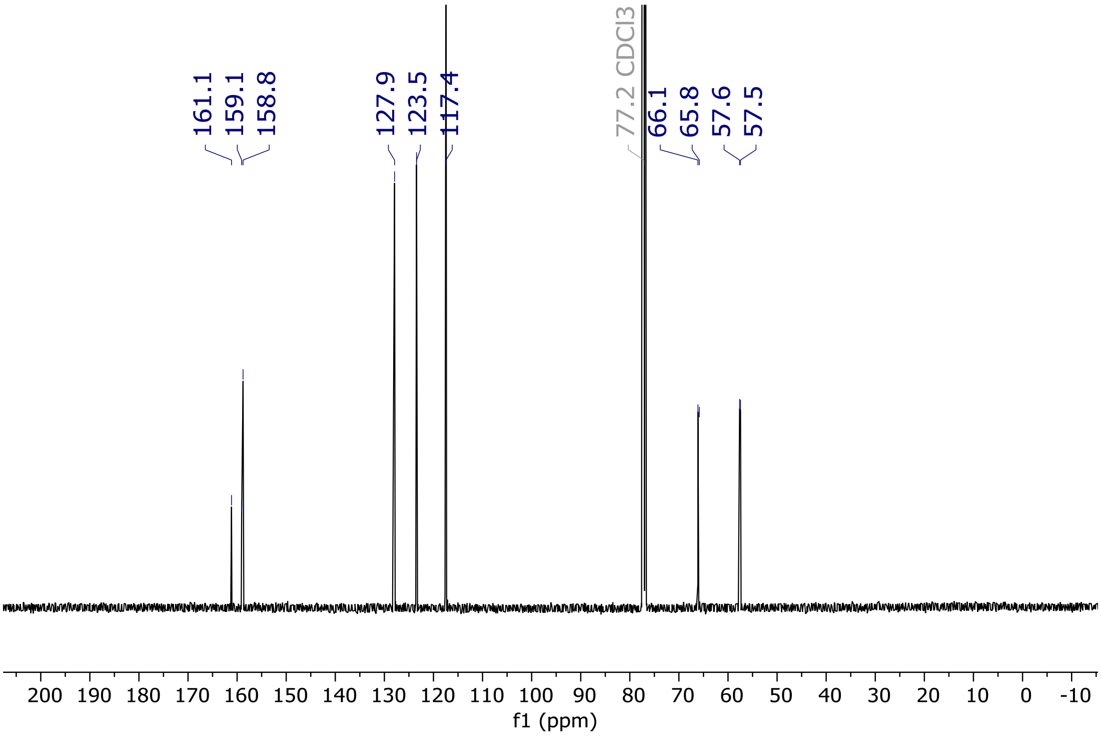

^{19}F NMR (471 MHz, $CDCl_3$):

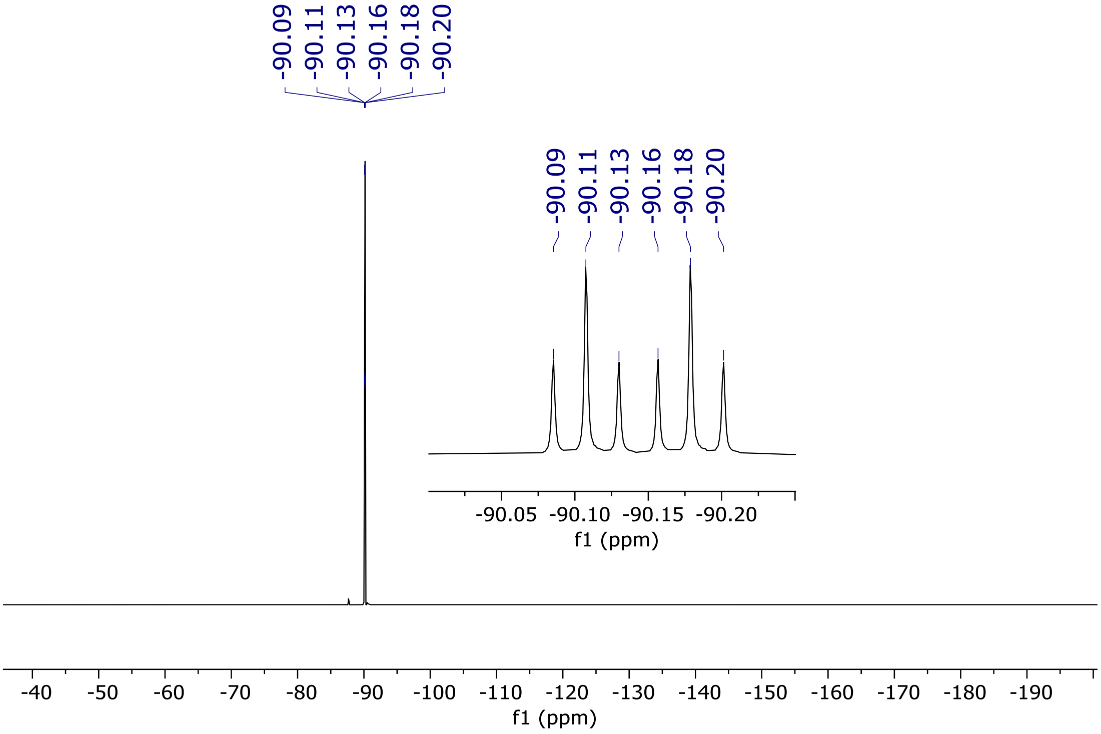

N-(2-fluoroallyl)-N-(4-fluorobenzyl)-4-methylbenzenesulfonamide ***(10a)***

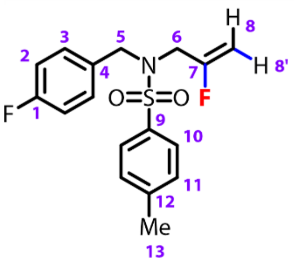

^{1}H NMR (500 MHz, CDCl$_3$):

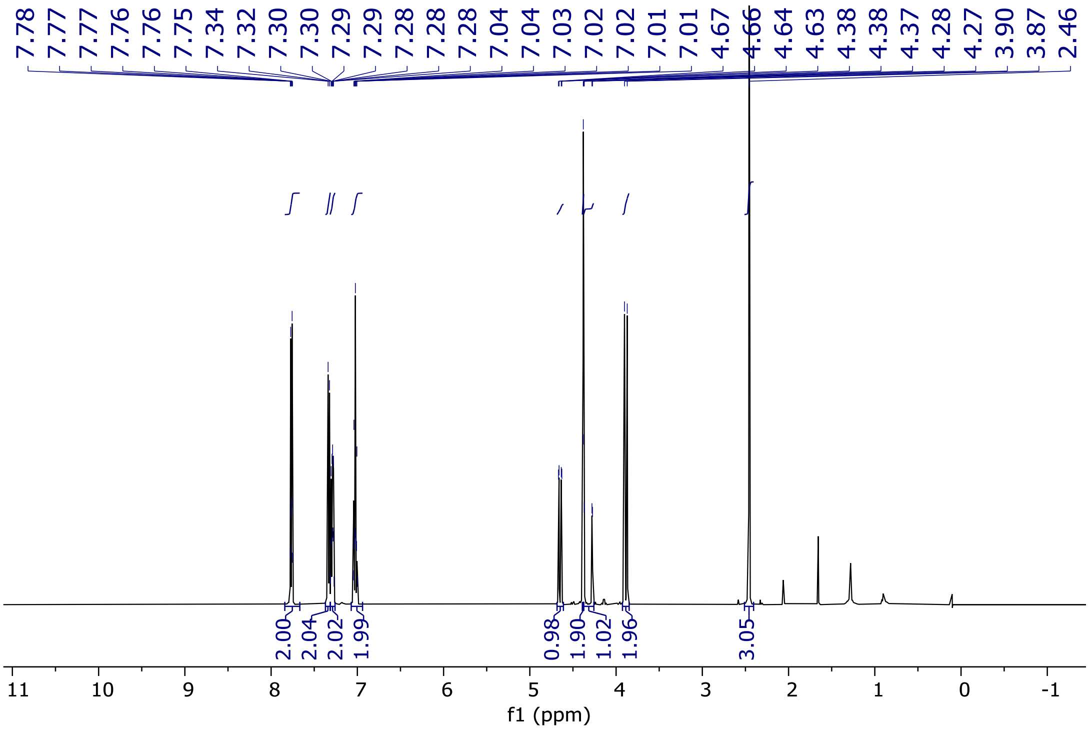

^{13}C NMR (126 MHz, CDCl$_3$):

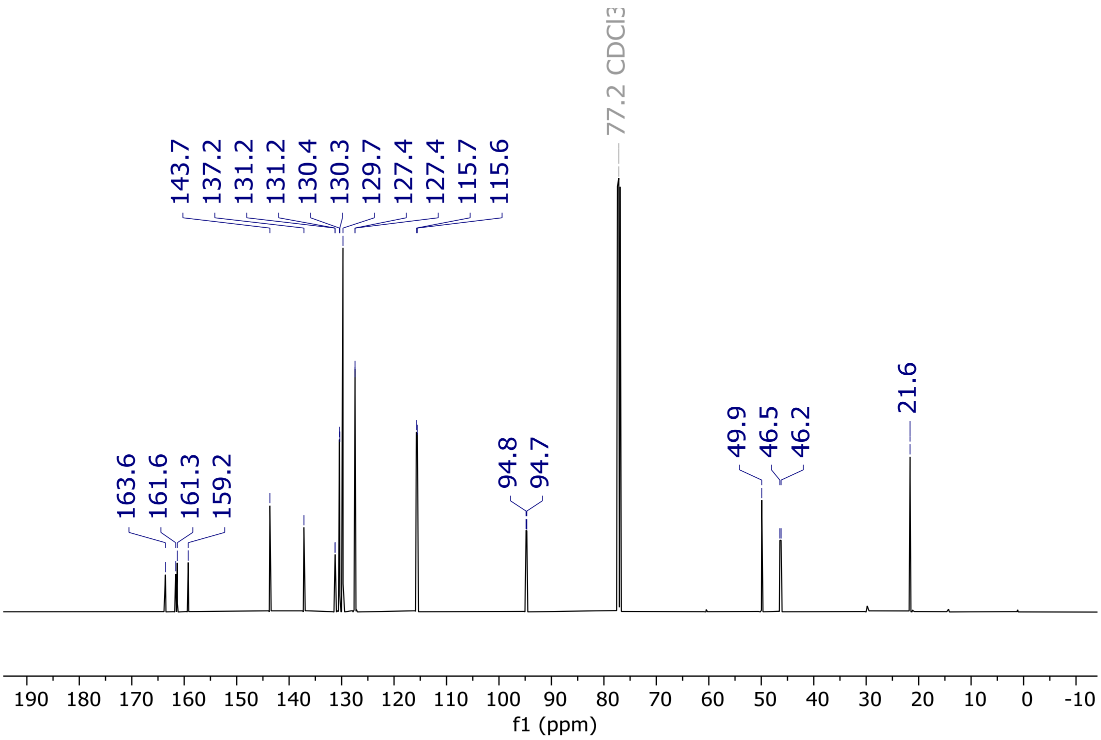

^{19}F NMR (376 MHz, $CDCl_3$):

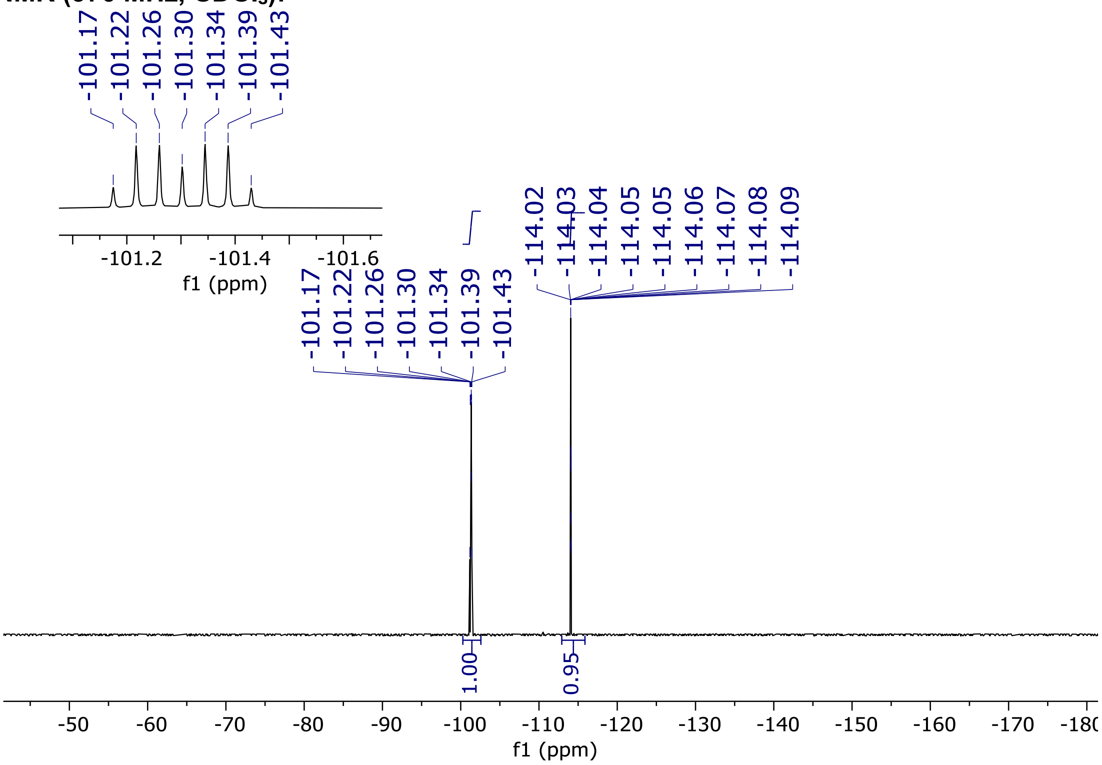

(Z)-3-fluoro-2-methyl-6-phenylhex-3-en-5-yn-2-ol ***(11a)***

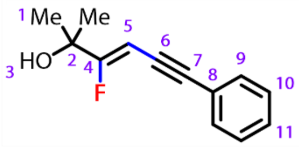

^{1}H NMR (500 MHz, $CDCl_3$):

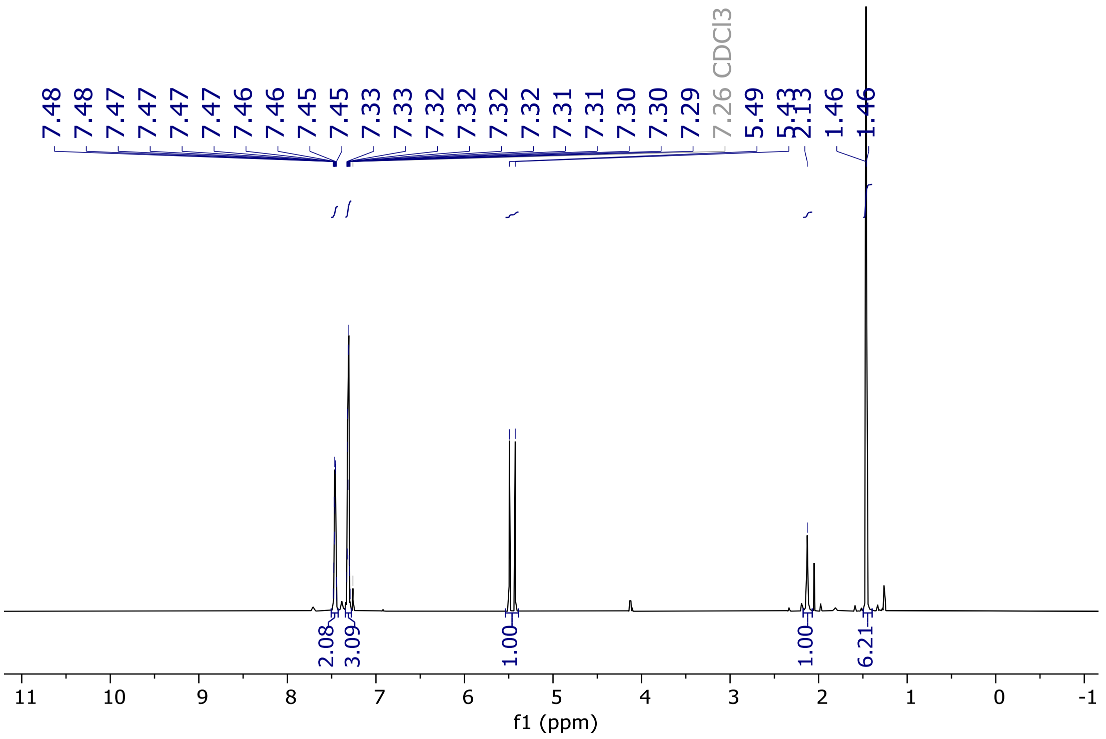

^{13}C NMR (126 MHz, $CDCl_3$):

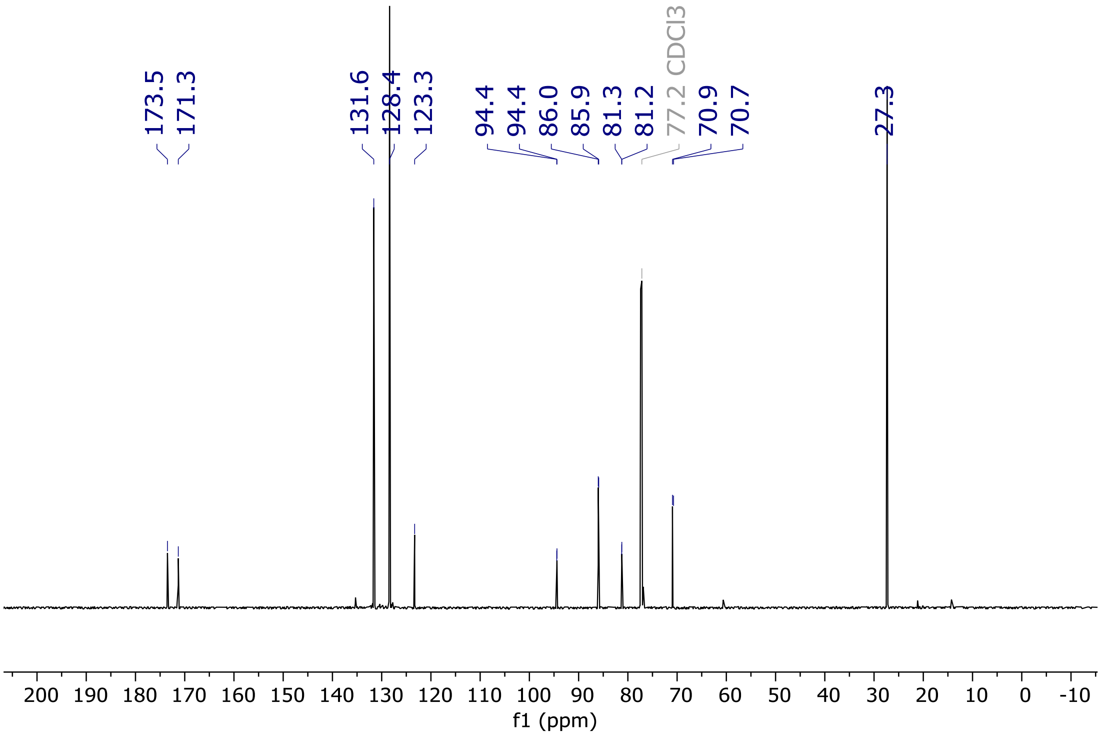

^{19}F NMR (471 MHz, $CDCl_3$):

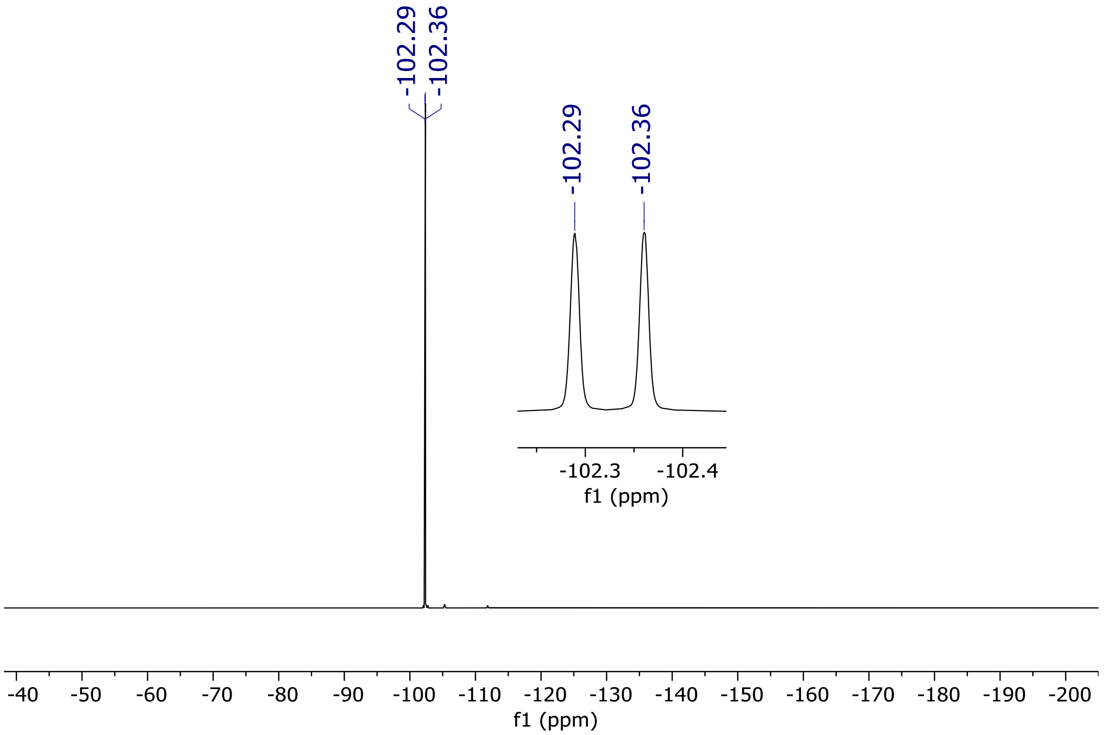

(Z)-6-fluoro-6-(4-fluorophenyl)-2-methylhex-5-en-3-yn-2-ol ***(11b)***

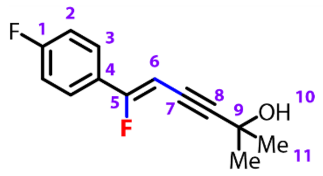

^{1}H NMR (500 MHz, CDCl$_3$):

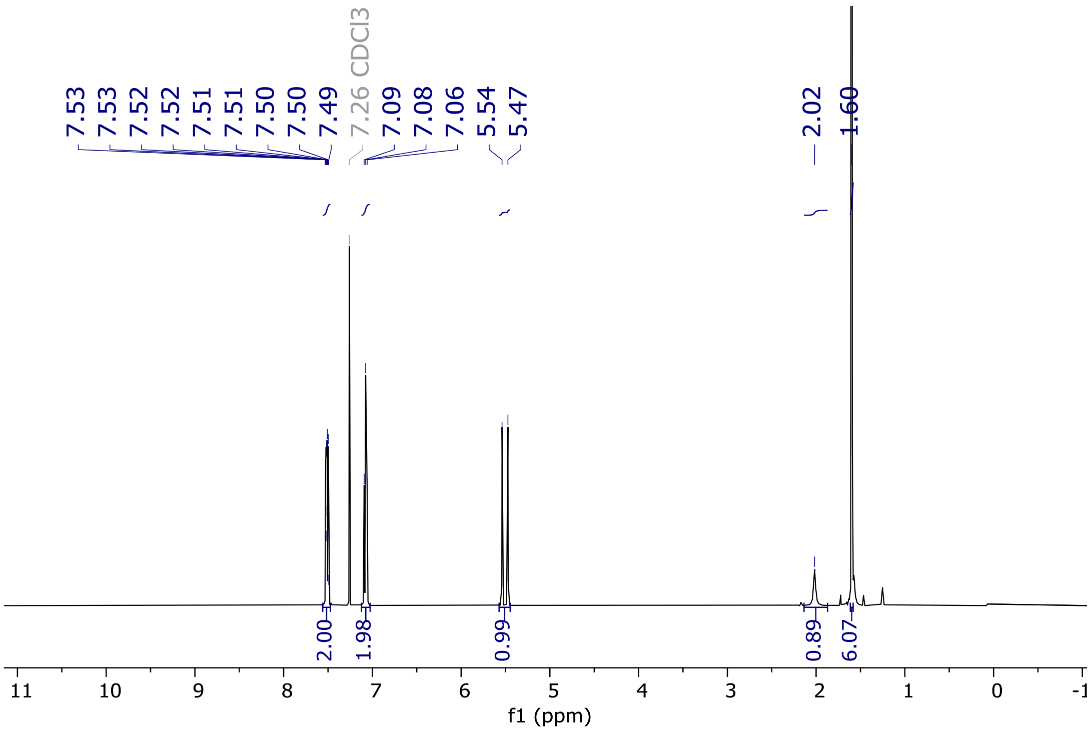

^{13}C NMR (126 MHz, CDCl$_3$):

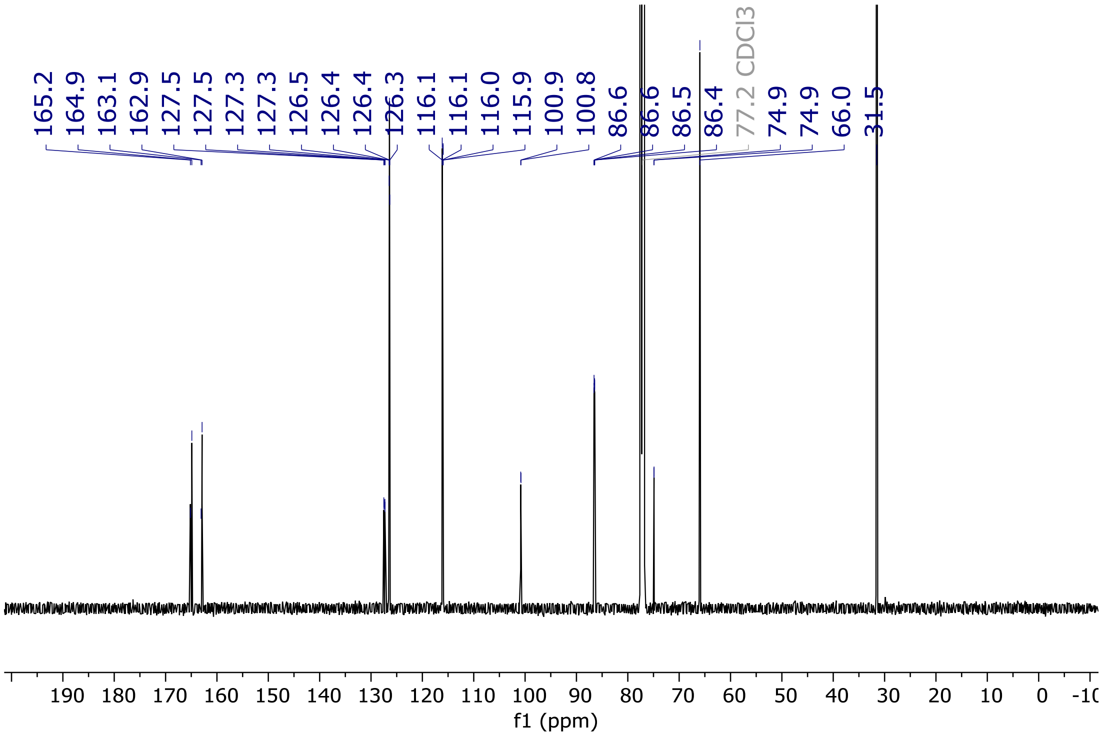

^{19}F NMR (376 MHz, CDCl$_3$):

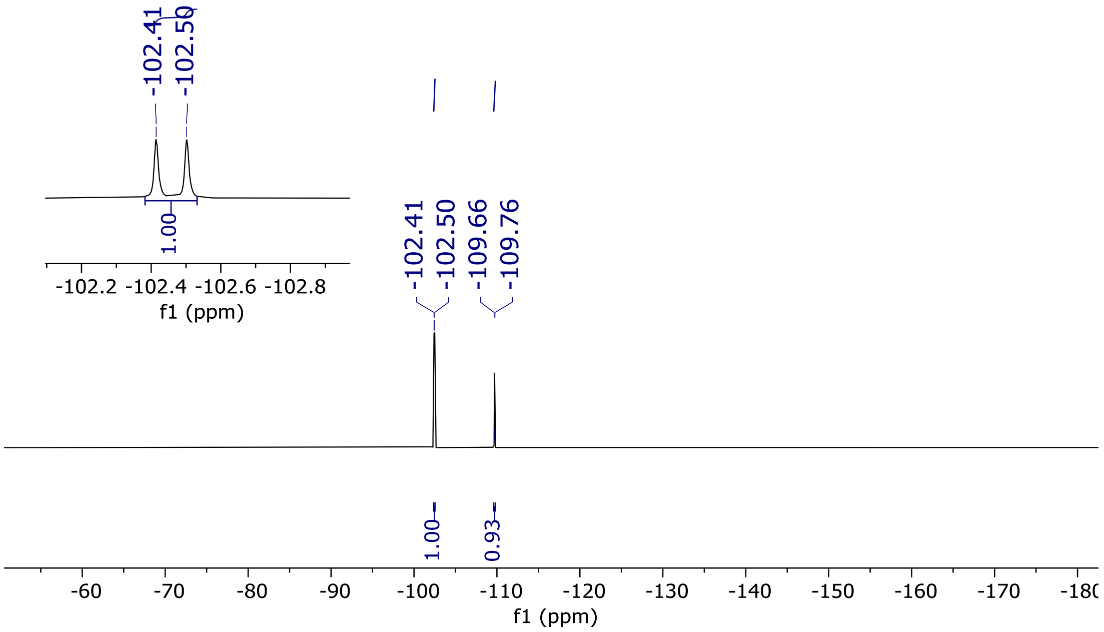

(Z)-(4-fluoro-5-methoxypent-3-en-1-yn-1-yl)benzene ***(11c)***

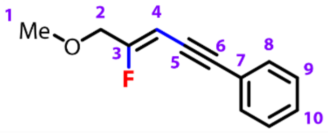

^{1}H NMR (500 MHz, CDCl$_3$):

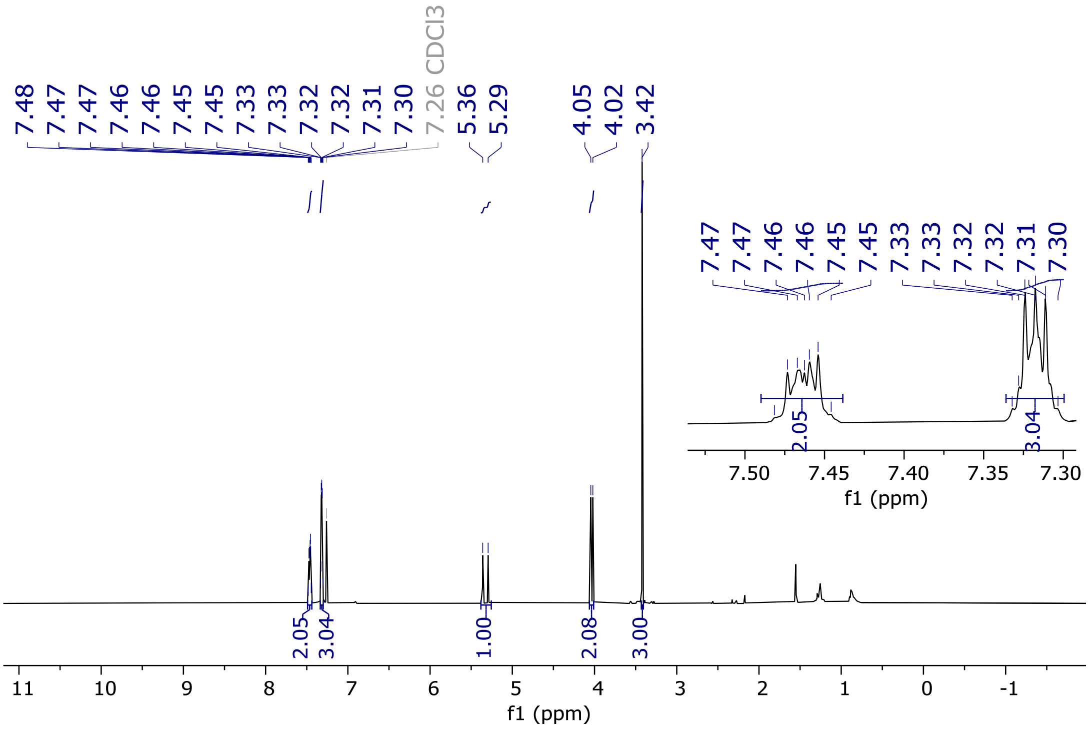

^{13}C NMR (126 MHz, CDCl$_3$):

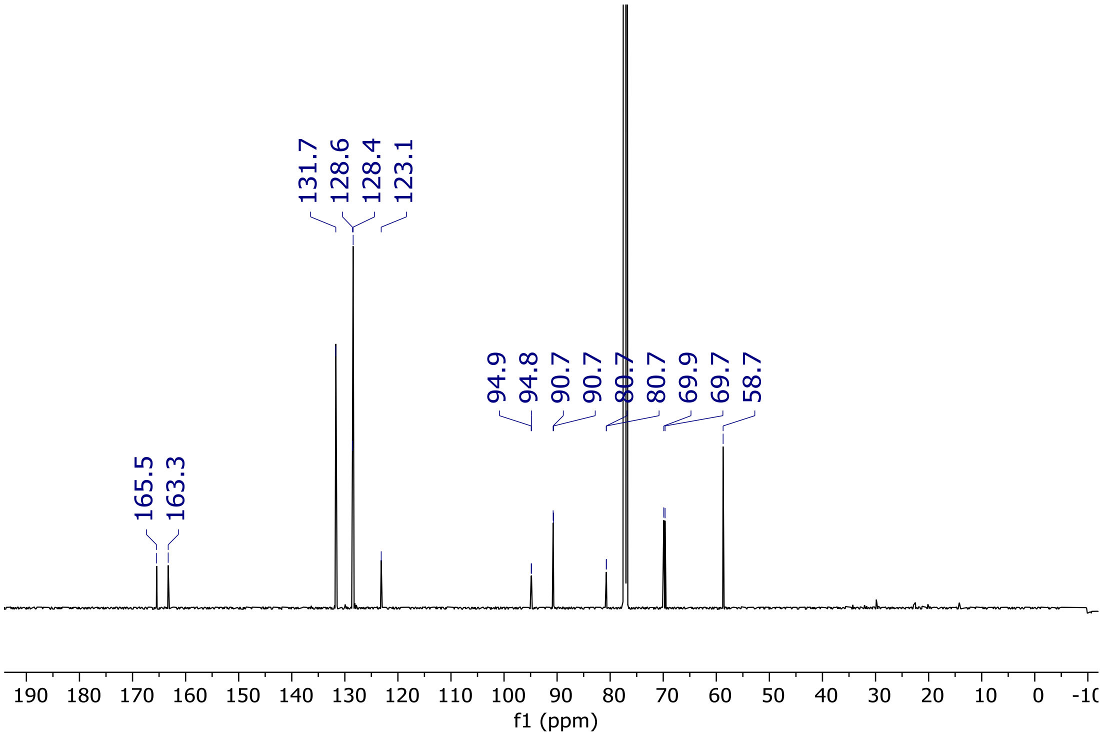

^{19}F NMR (376 MHz, $C(CD_3)_2O$):

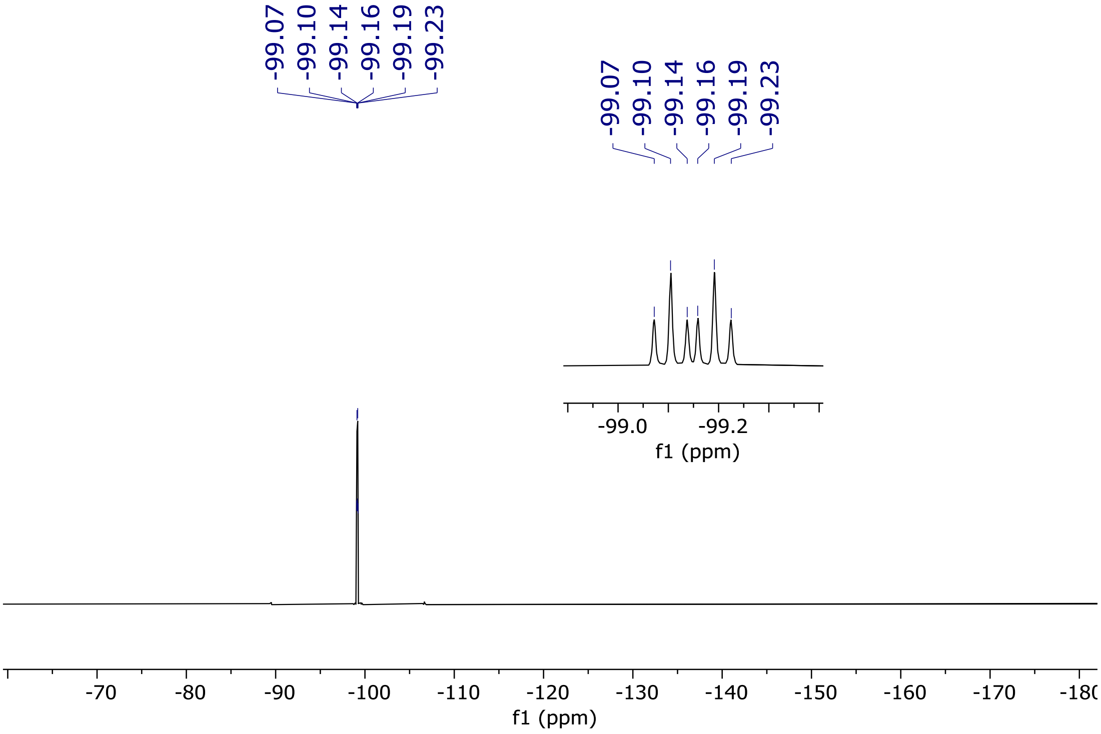

(Z)-N-(2-fluoro-5-phenylpent-2-en-4-yn-1-yl)-N-(4-fluorobenzyl)-4-methylbenzenesulfonamide ***(11d)***

^{1}H NMR (500 MHz, CDCl$_3$):

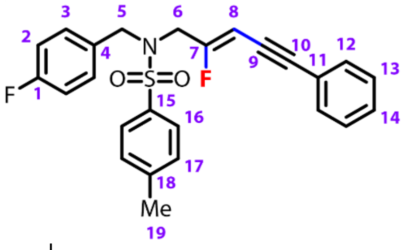

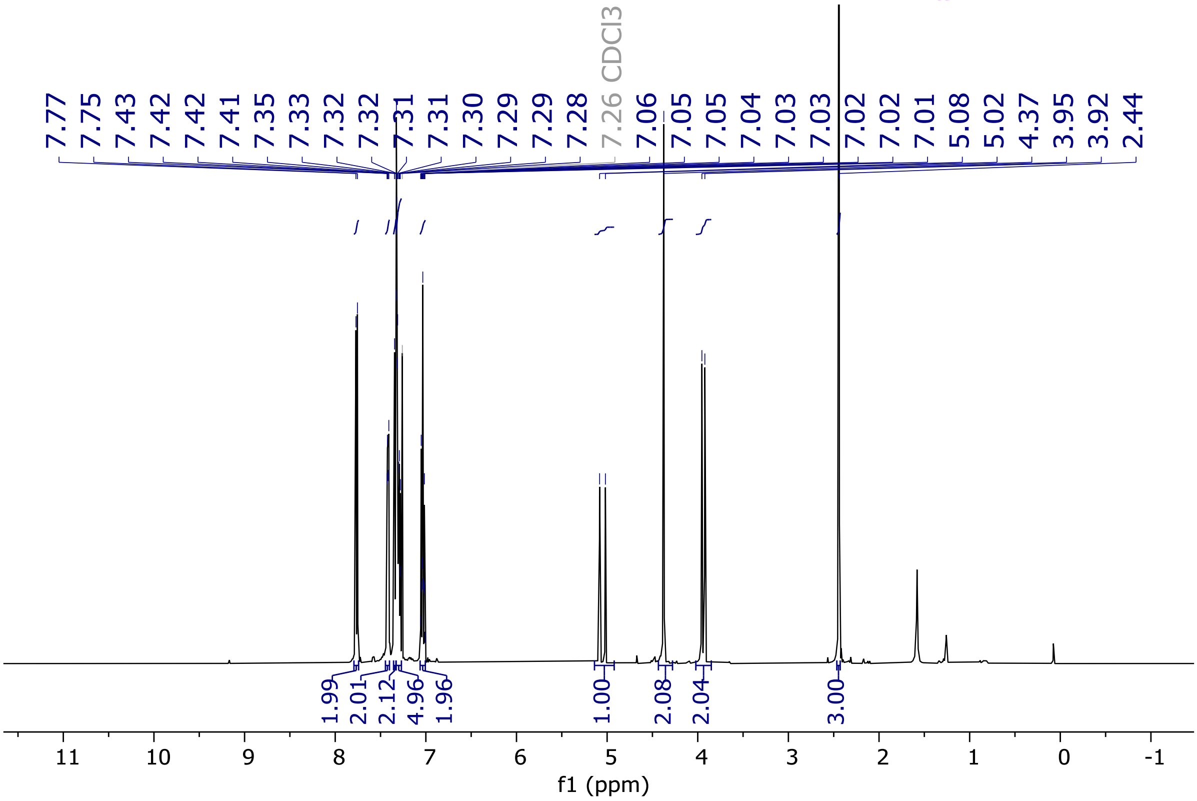

^{13}C NMR (126 MHz, CDCl$_3$):

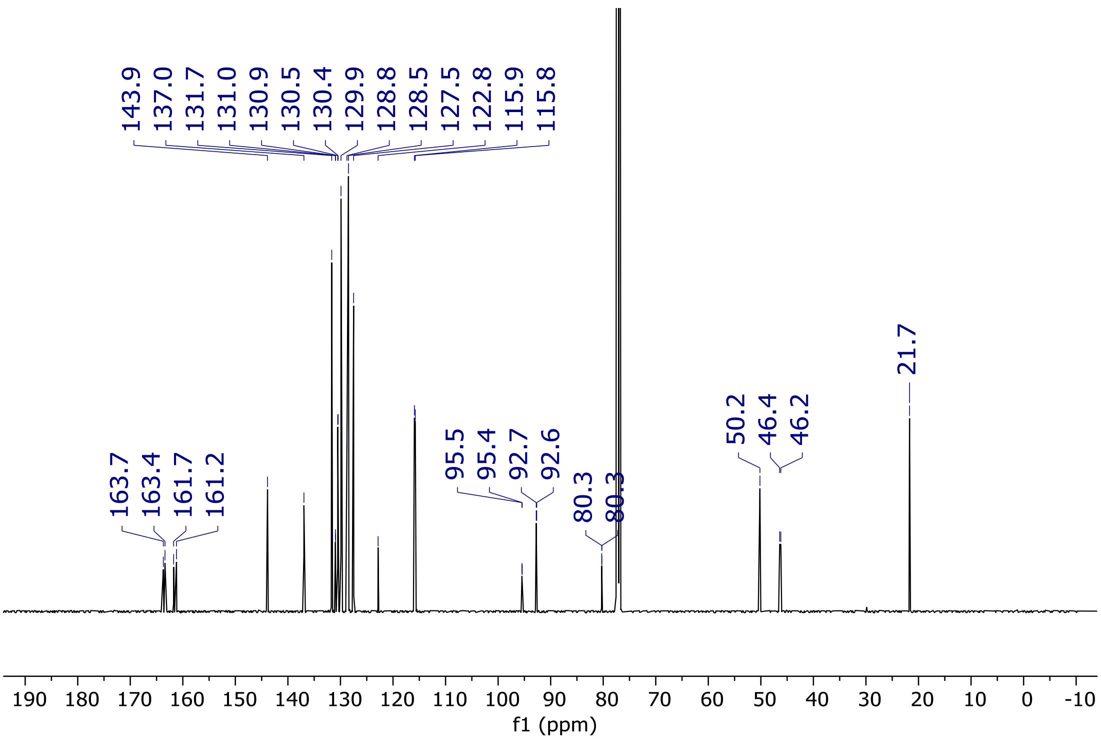

^{19}F NMR (376 MHz, CDCl$_3$):

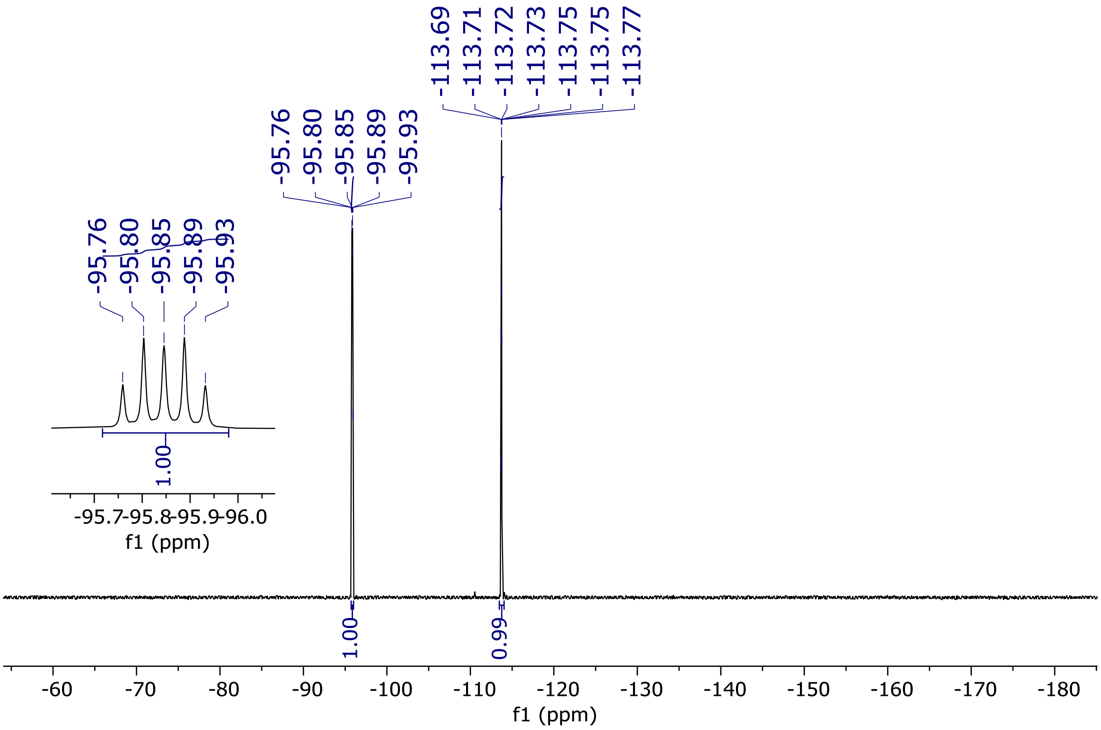

<u>(Z)-6-fluoro-6-(thiophen-3-yl)hex-5-en-3-yn-1-ol</u> ***<u>(11e)</u>***

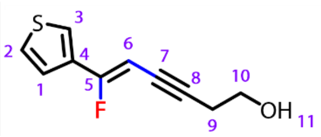

^{1}H NMR (500 MHz, $CDCl_3$):

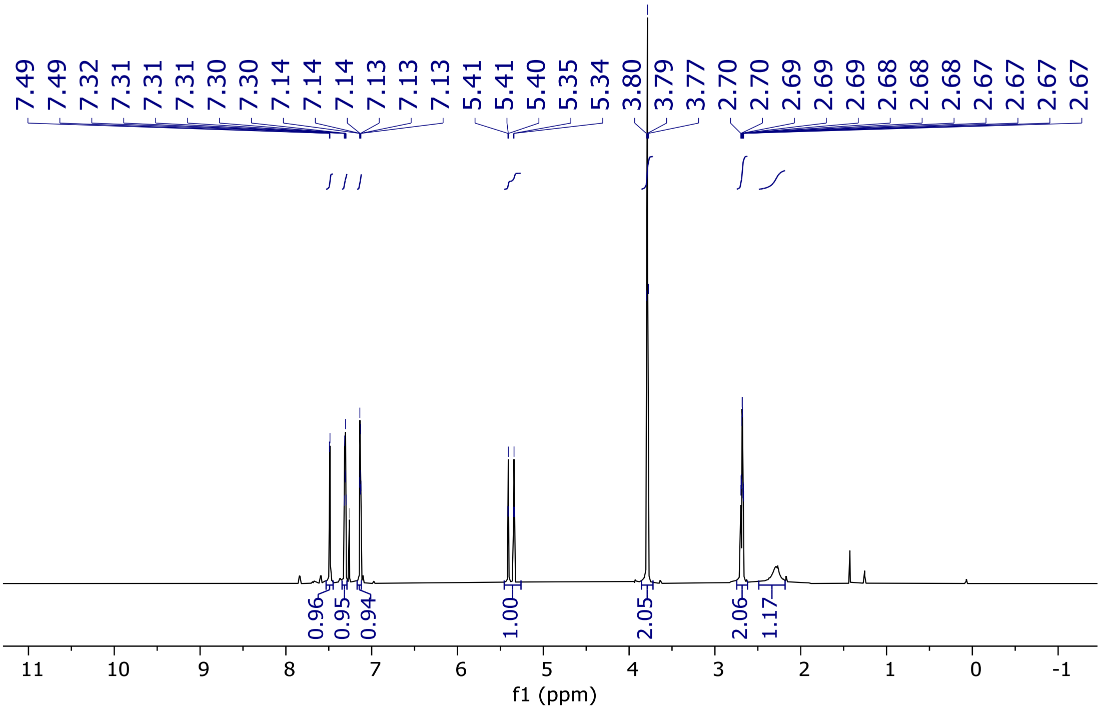

^{13}C NMR (126 MHz, $CDCl_3$):

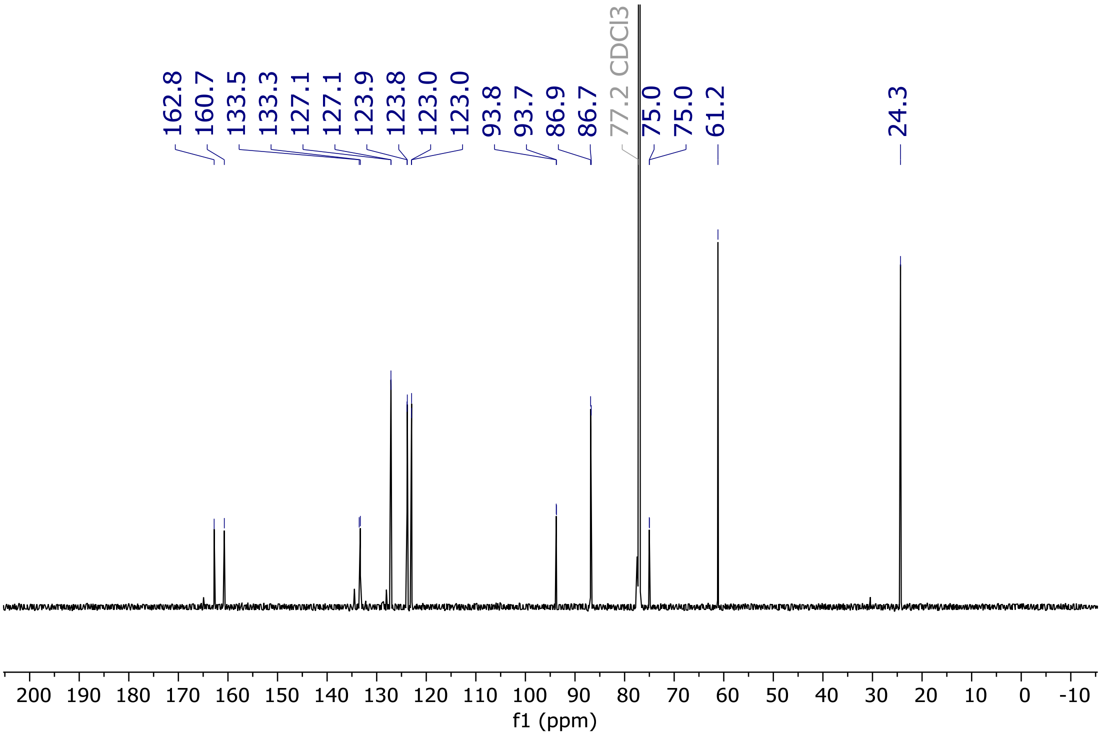

^{19}F NMR (471 MHz, $CDCl_3$):

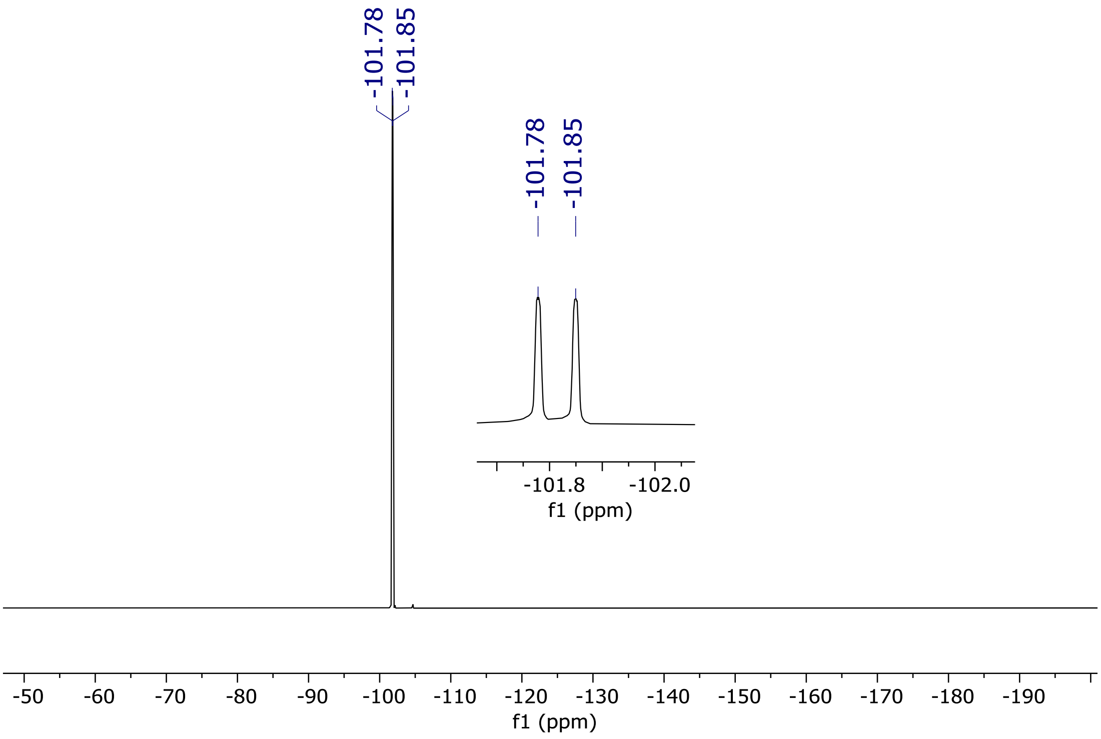

(Z)-1-fluoro-4-(2-fluoro-5-phenylpent-1-en-1-yl)benzene ***(12a)***

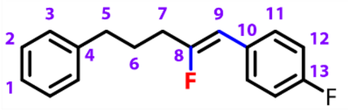

^{1}H NMR (600 MHz, CDCl$_3$):

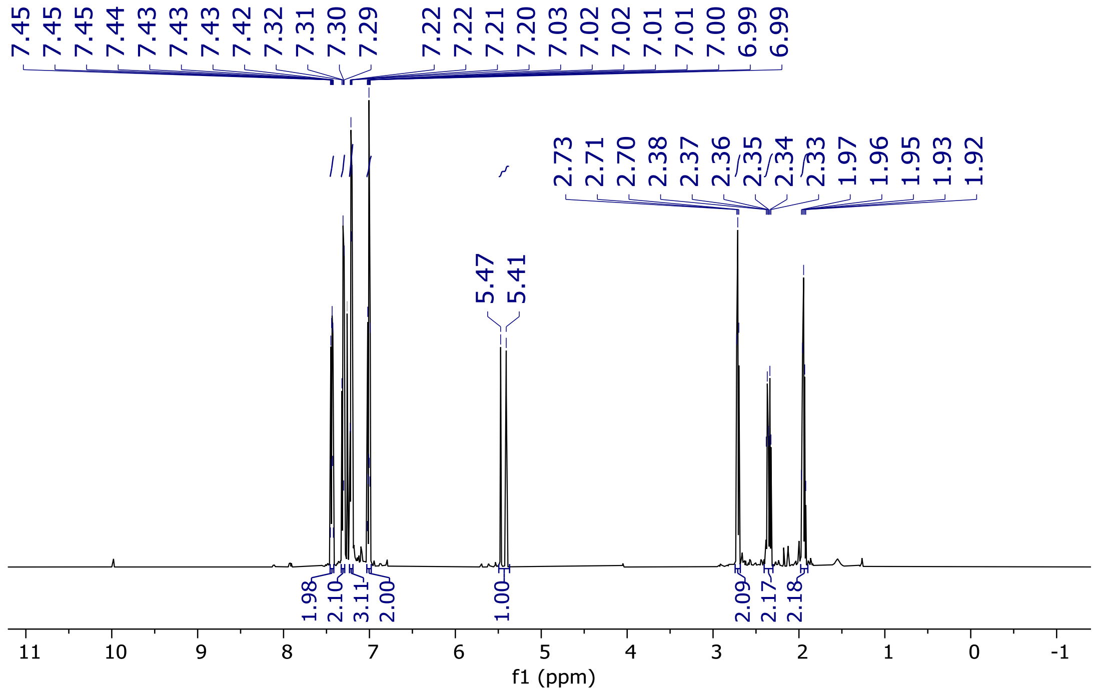

^{13}C NMR (151 MHz, CDCl$_3$):

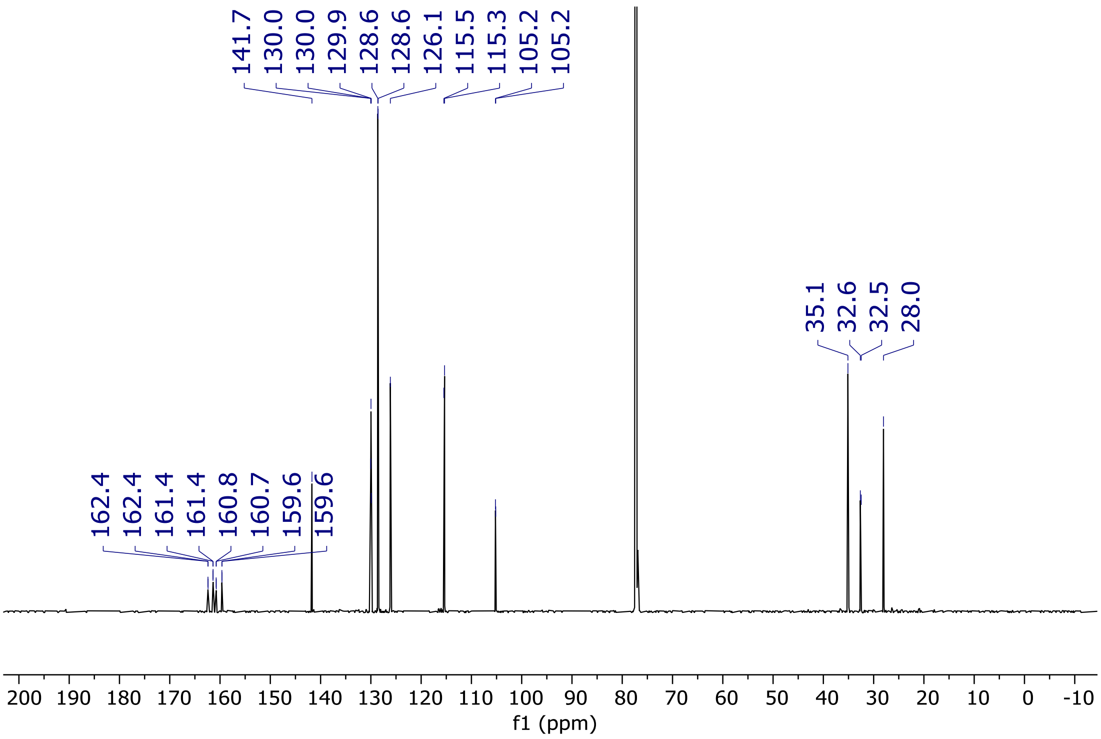

^{19}F NMR (283 MHz, CDCl$_3$):

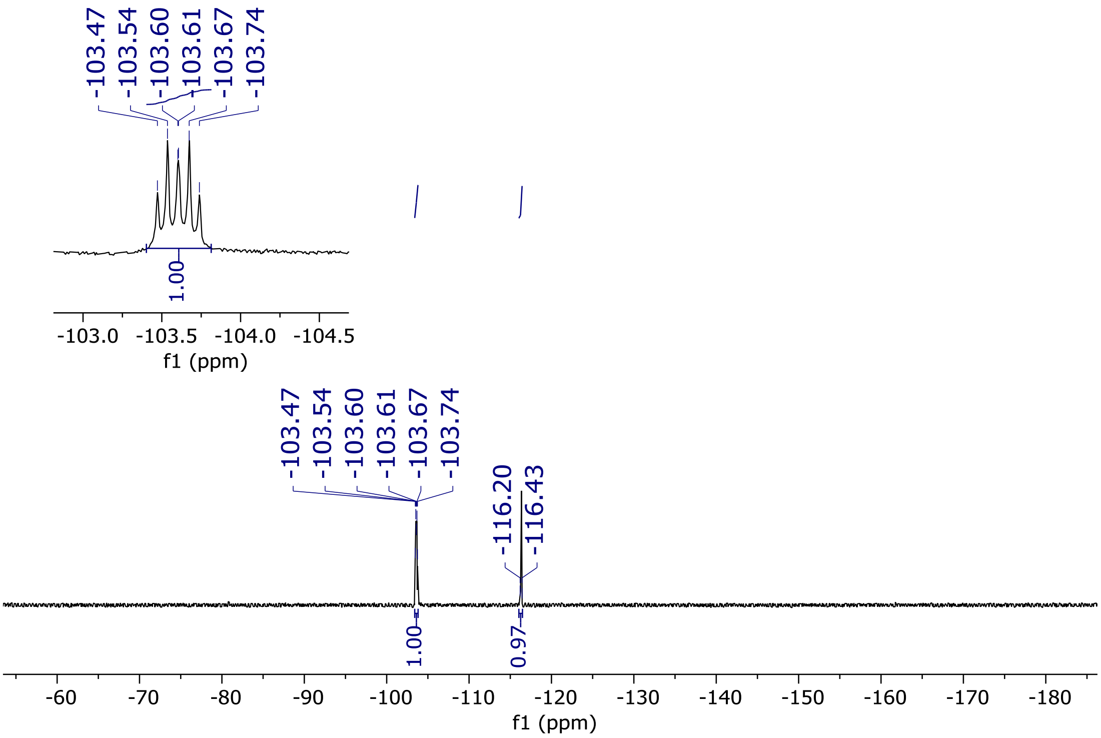

(Z)-2-(2-fluoro-5-phenylpent-1-en-1-yl)benzo[b]thiophene ***(12b)***

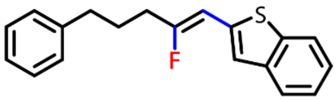

^{1}H NMR (500 MHz, $CDCl_3$):

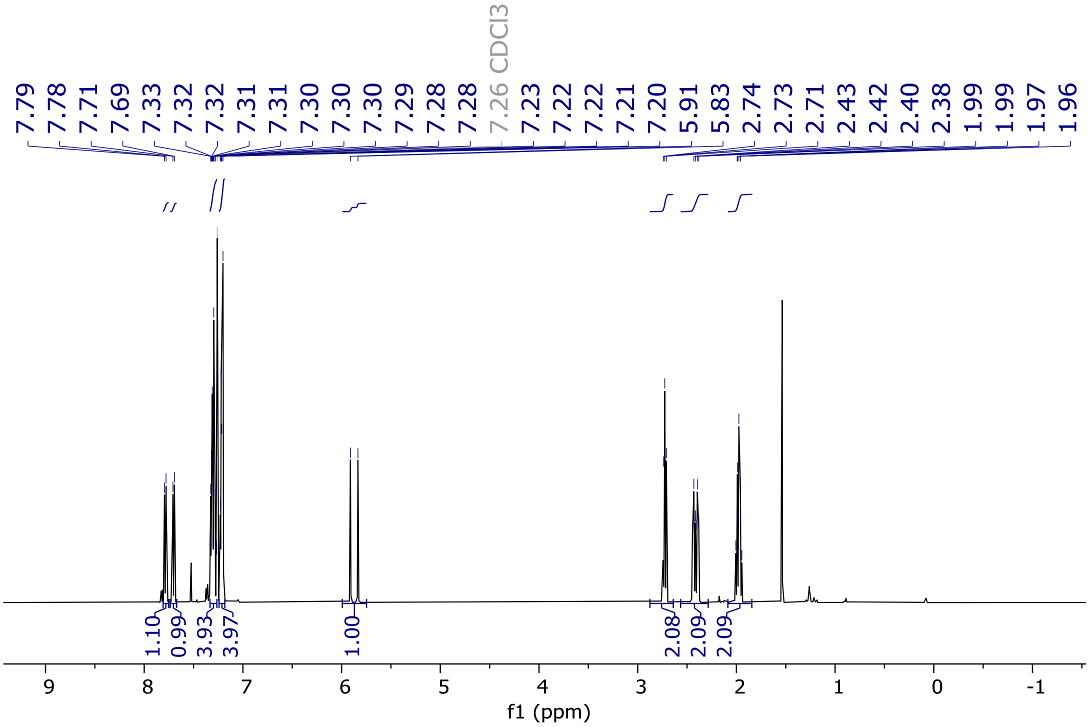

^{13}C NMR (126 MHz, $CDCl_3$):

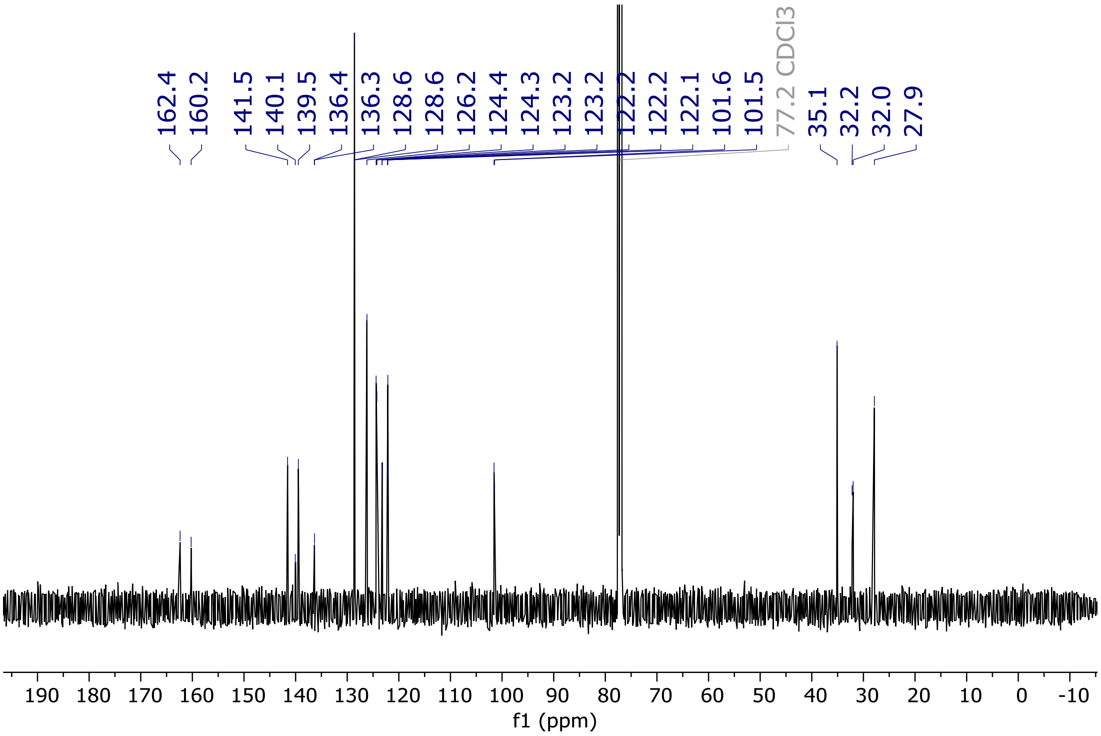

^{19}F NMR (376 MHz, $CDCl_3$)

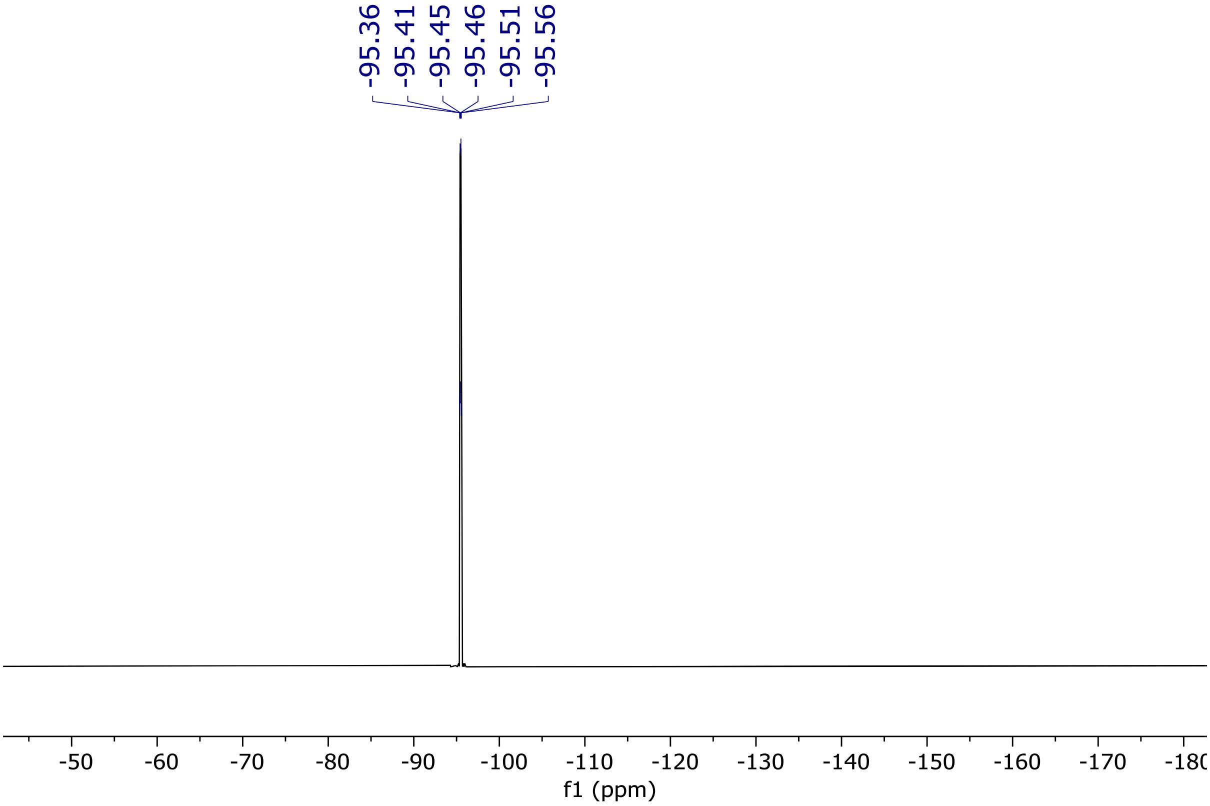

*Methyl (Z)-3-fluoro-6-phenylhex-2-enoate **(13a)***

^{1}H NMR (500 MHz, $CDCl_3$):

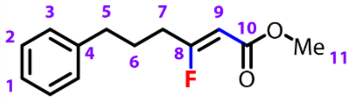

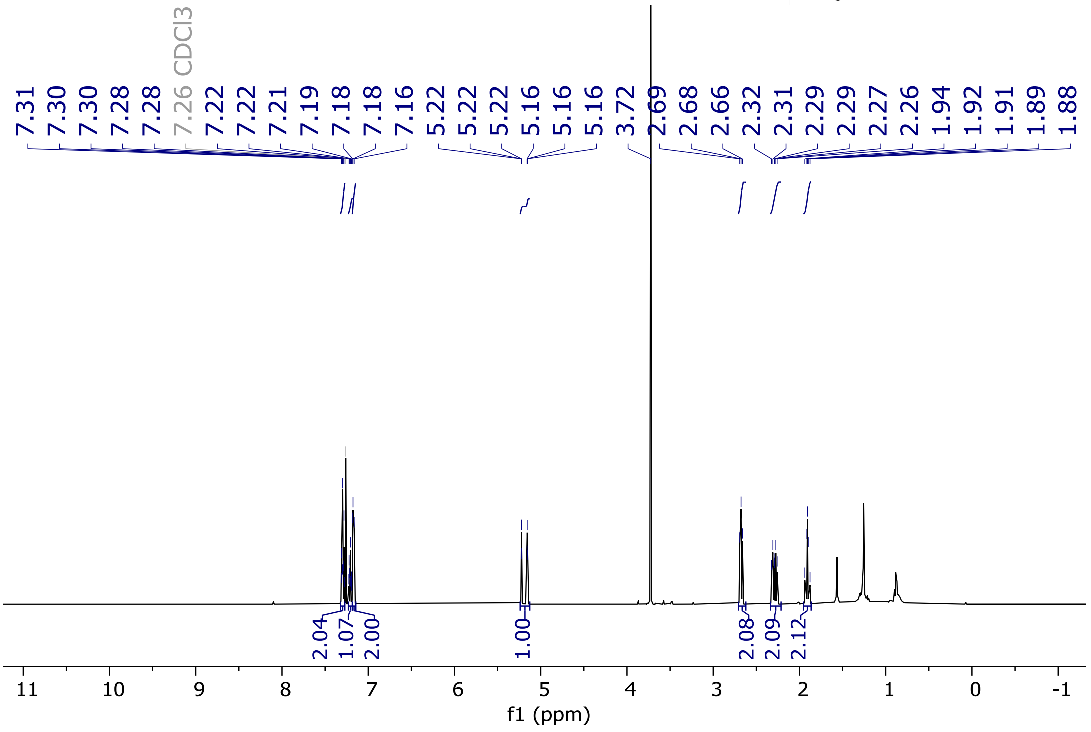

^{13}C NMR (126 MHz, $CDCl_3$):

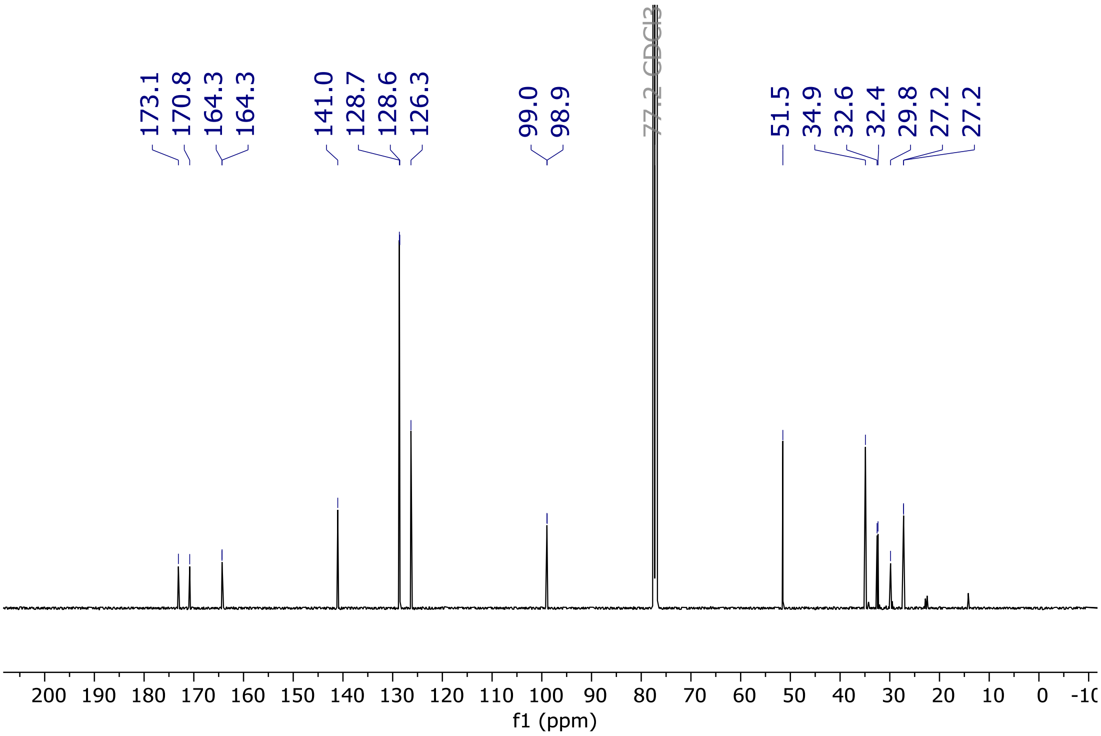

^{19}F NMR (376 MHz, CDCl$_3$):

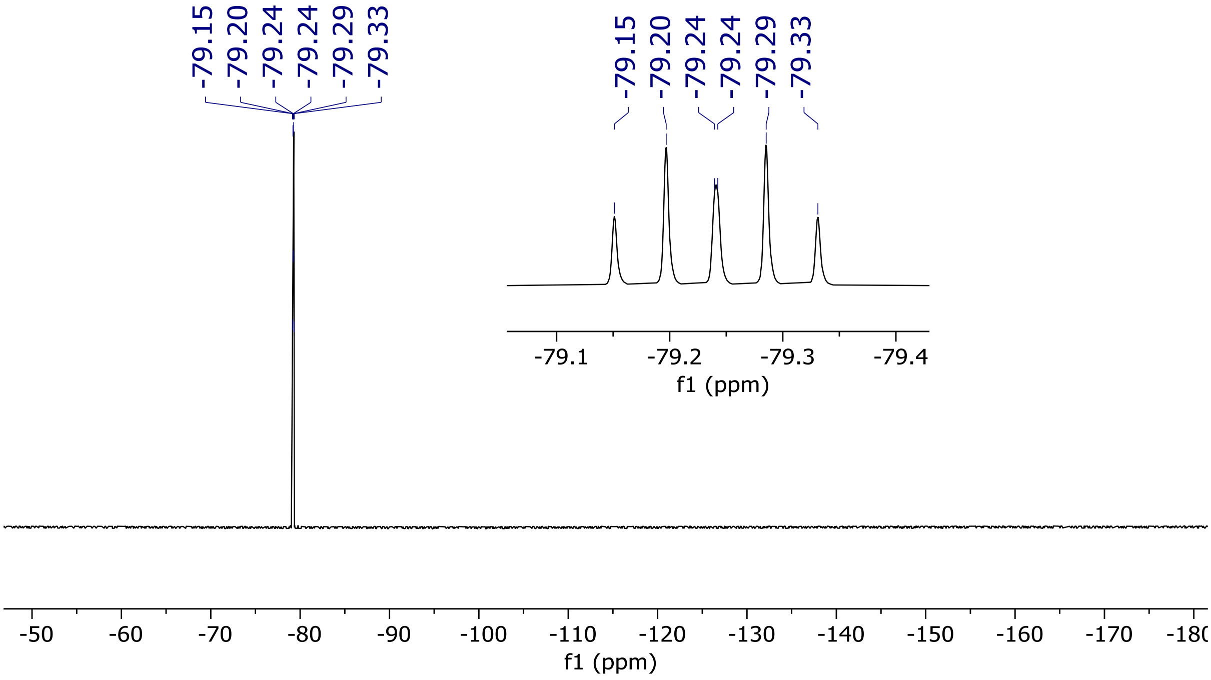

2,4,6-Trichlorophenyl (Z)-3-fluoro-4,4-dimethylpent-2-enoate ***(13b)***

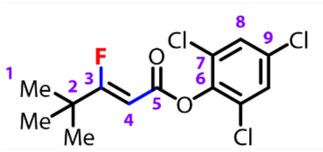

^{1}H NMR (500 MHz, CDCl$_3$):

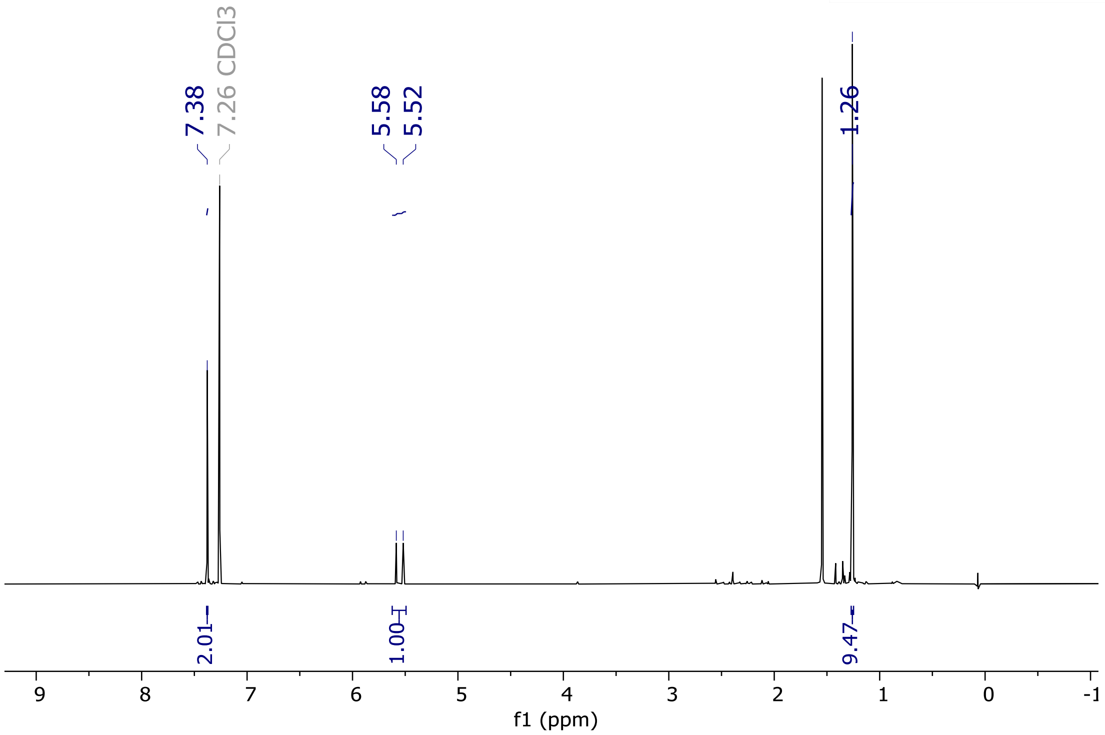

^{13}C NMR (126 MHz, CDCl$_3$):

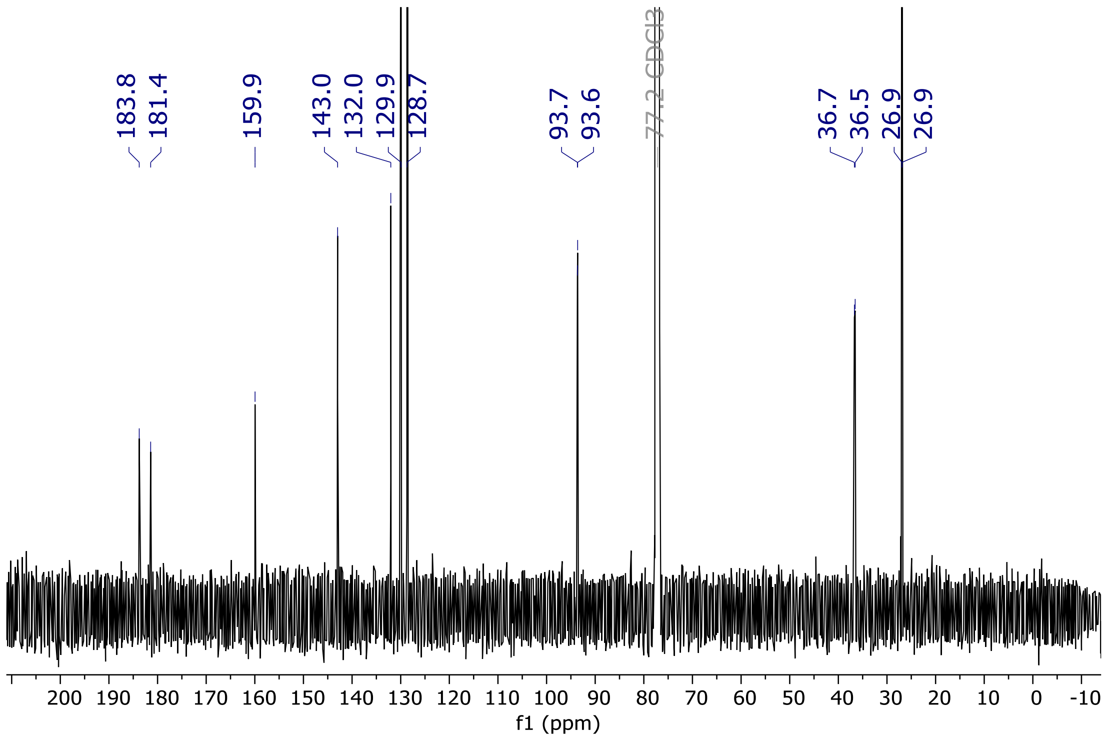

^{19}F NMR (376 MHz, CDCl$_3$):

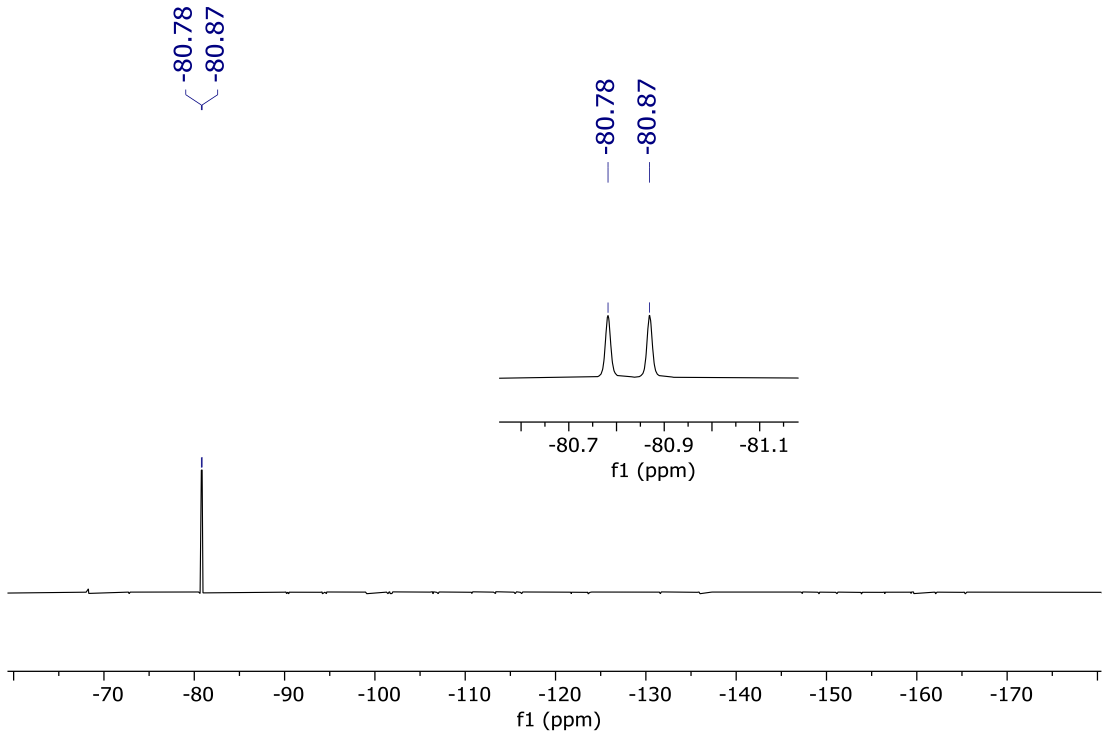

(Z)-3-fluoro-4,4-dimethyl-N-(4-methylbenzyl)pent-2-enamide ***(13c)***

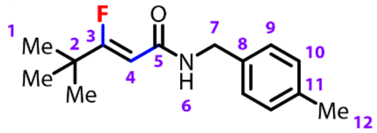

^{1}H NMR (500 MHz, CDCl$_3$):

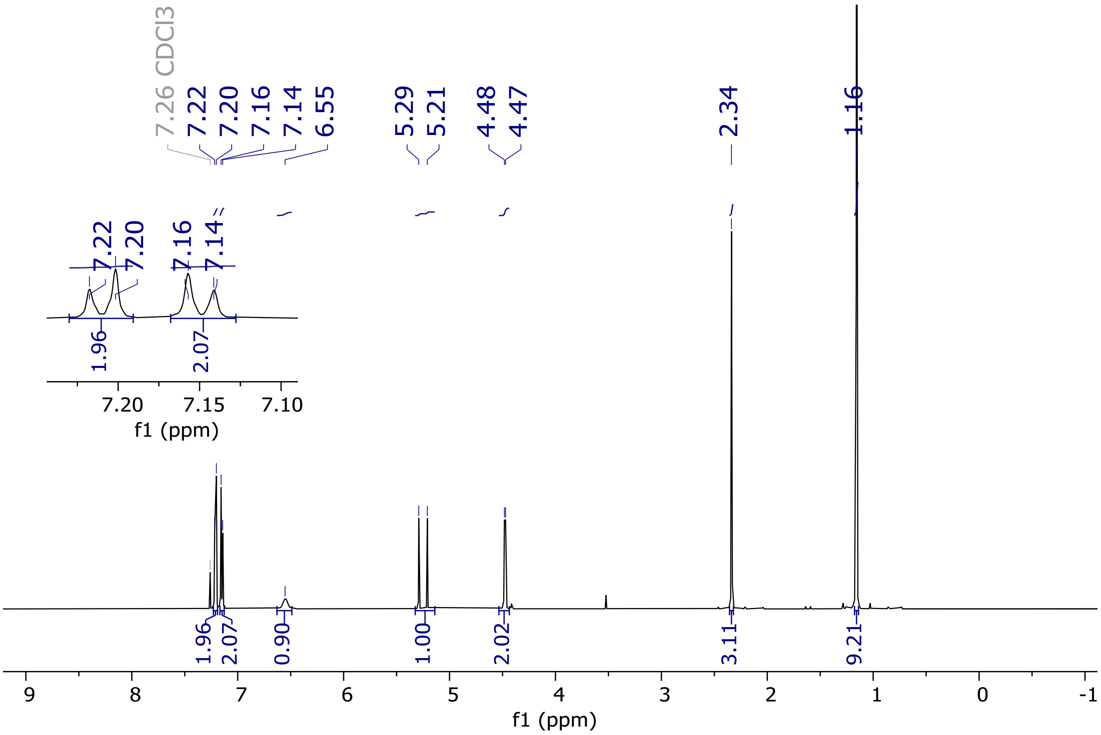

^{13}C NMR (126 MHz, CDCl$_3$):

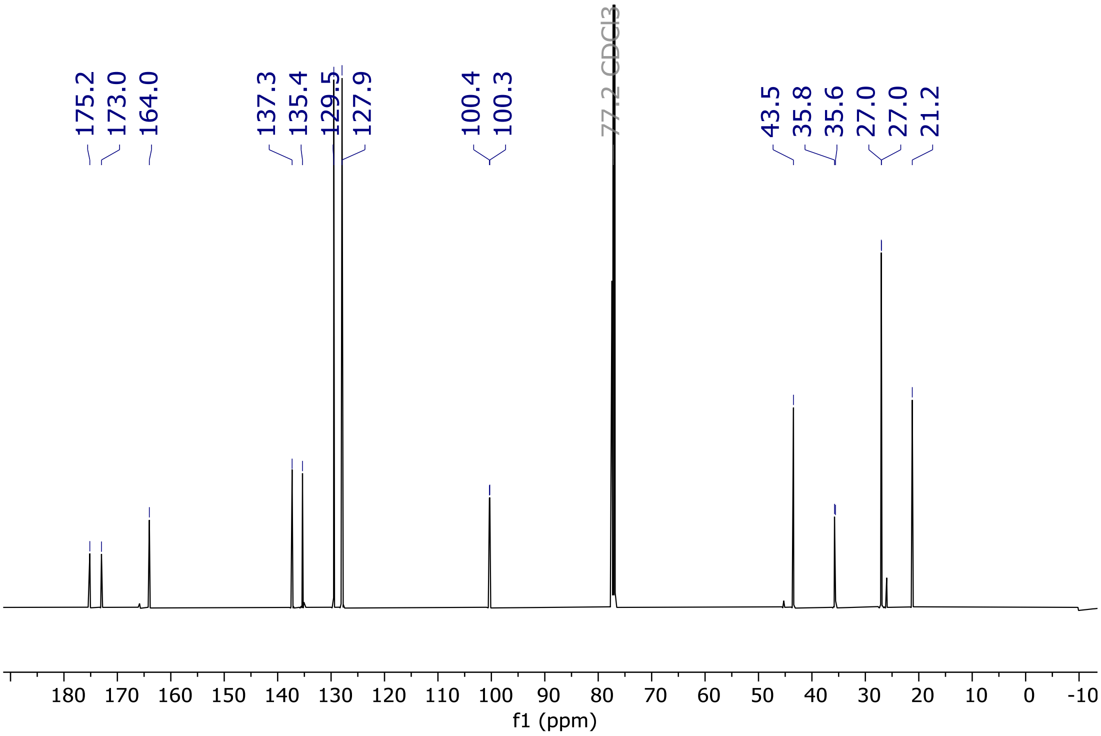

^{19}F NMR (376 MHz, $CDCl_3$):

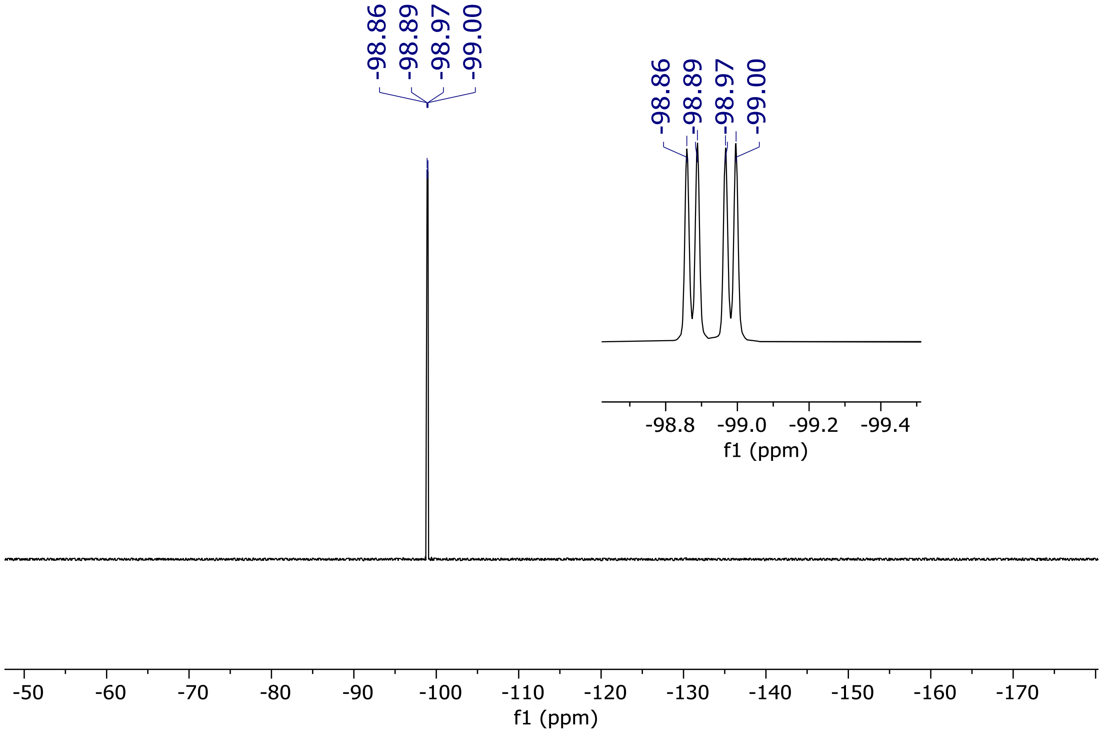

^{19}F NMR (376 MHz, $C(CD_3)_2O$):

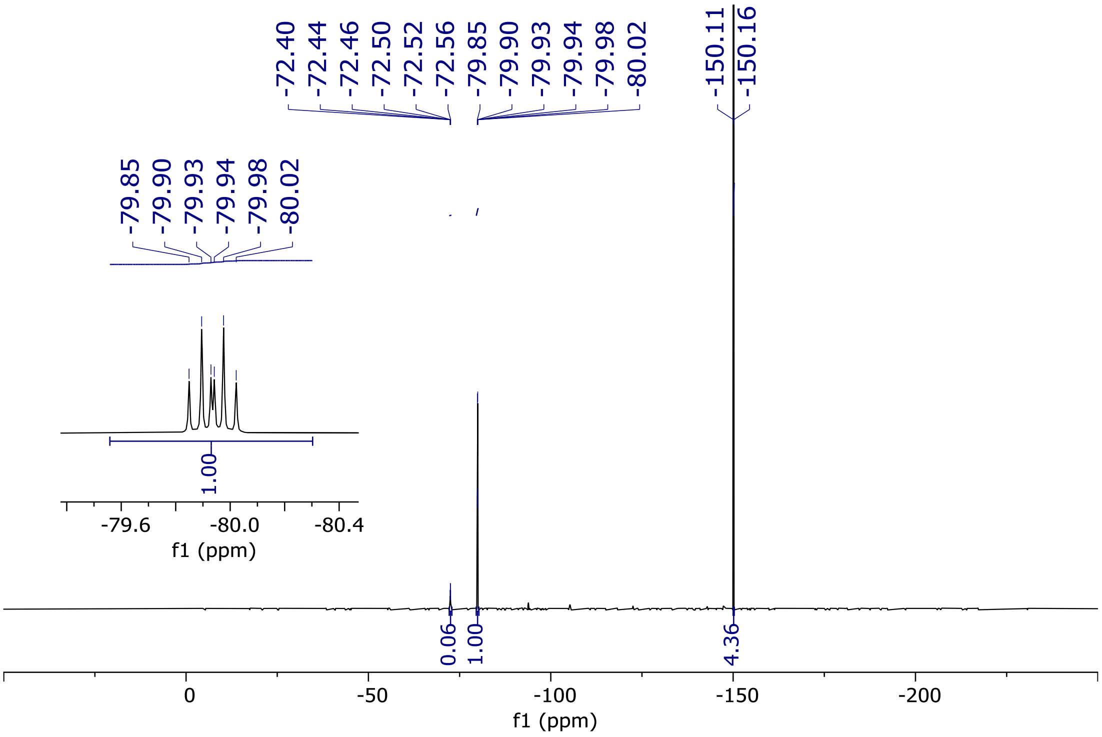

(Z)-(4-fluoro-5-iodopent-4-en-1-yl)benzene

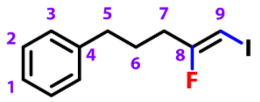

^{1}H NMR (500 MHz, CDCl$_3$):

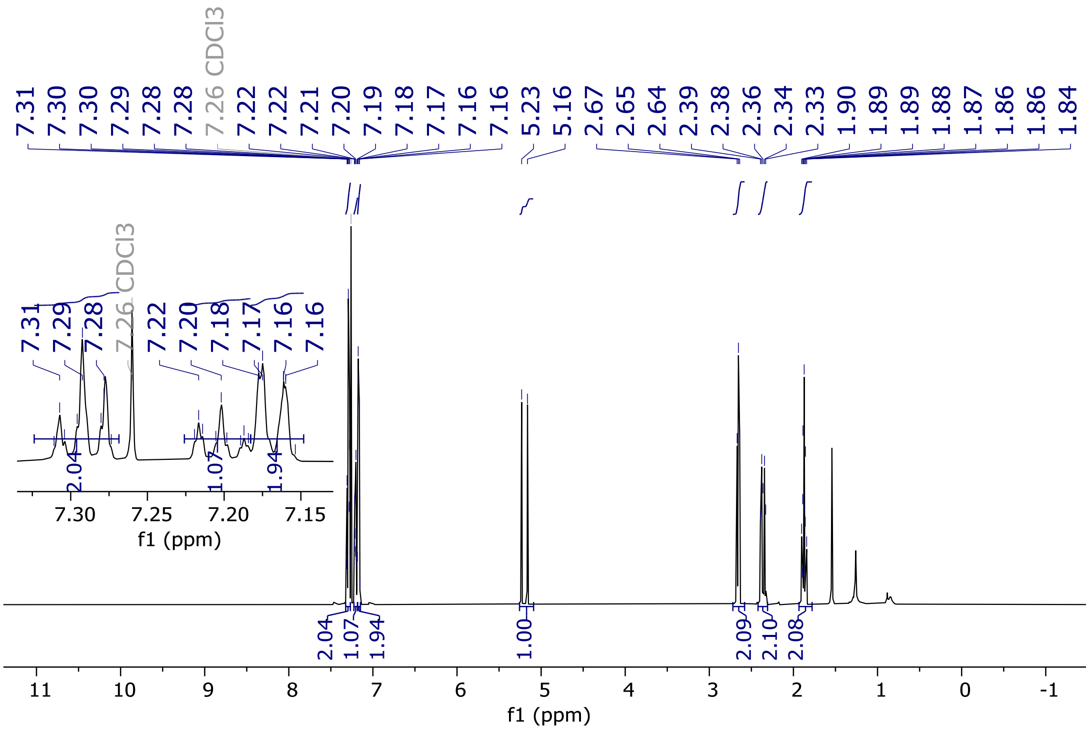

^{13}C NMR (126 MHz, CDCl$_3$):

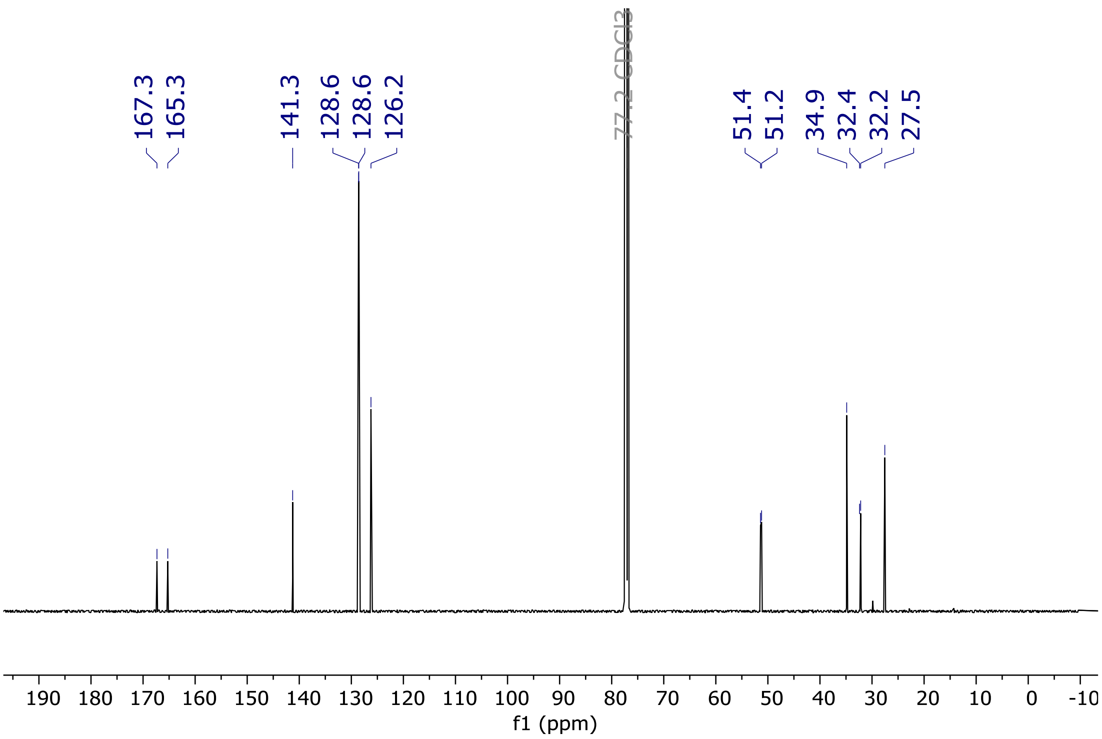

^{19}F NMR (376 MHz, $CDCl_3$):

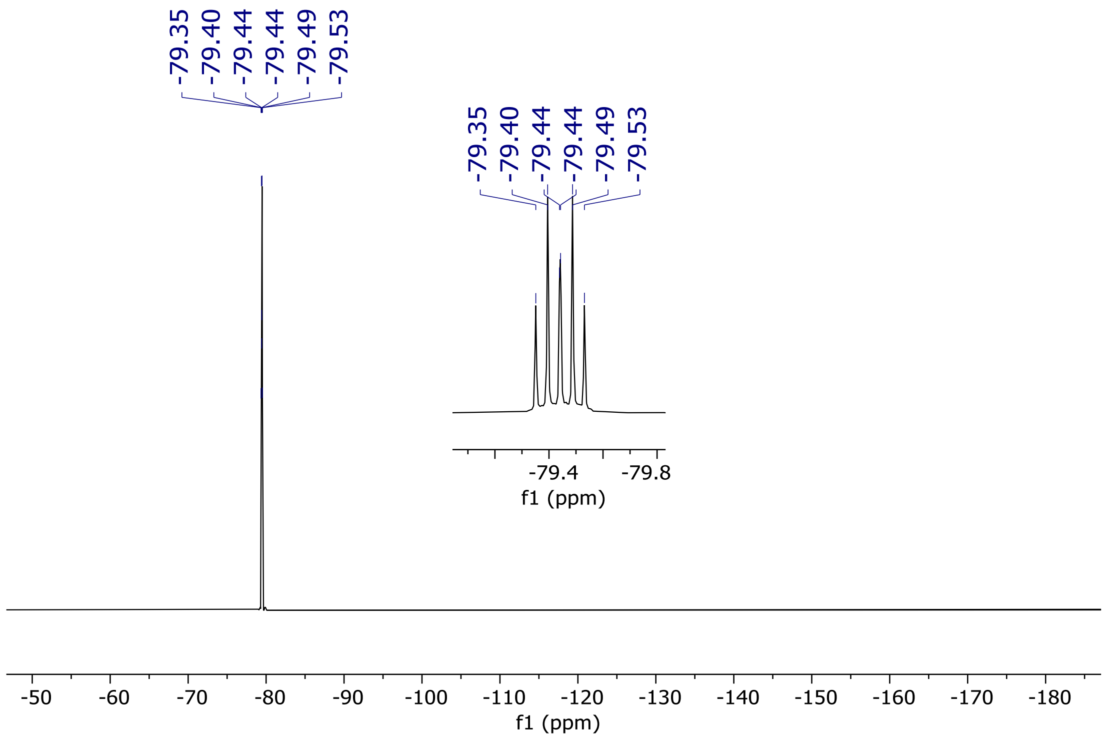

Computational Data

The following computational work was carried out using the computational facilities of the Advanced Computing Research Centre, University of Bristol - http://www.bristol.ac.uk/acrc/. Density functional theory (DFT) calculations were undertaken using Gaussian 16. Geometry optimisations and frequency calculations were performed using the MO62x functional, using 6-31+G(d,p) for H, C, B, and F atoms, and the SDD basis set along with its associated pseudopotential for I and Ag atoms. frequency calculations were conducted after geometry optimisations, both to verify stationary points as minima (no imaginary frequencies) or transition states (exactly one imaginary frequency), and to calculate thermodynamic data. Single-point energy calculations were conducted on the geometry-optimised structures using both (1) Def2TZVP on all atoms, and (2) aug-cc-pVTZ on H, C, B, and F atoms, and aug-cc-pVTZ-pp on I and Ag. Intrinsic reaction coordinate (IRC) calculations were carried out on each transition state (TS) to verify the reaction pathway, and to find/confirm the intermediates either side of the transition state. All calculations included implicit solvation ($MeNO_2$) using the integral equation formalism variant (IEFPCM) of the Polarisable Continuum Model (PCM). CYLview20 was used for the visualisation of the optimised structures in this text. The Basis Set Exchange resource was used for calculations employing aug-cc-pVTZ(-pp).[42]

Natural Bond Order (NBO) Analyses

Alkynyl Iodonium species

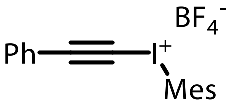

Atom No.	Atom	Charge
1	C	-0.20813
2	C	-0.16667
3	C	-0.16731
4	C	-0.16275
5	C	-0.20948
6	C	-0.19044
7	H	0.22543
8	H	0.24559
9	H	0.22863
10	H	0.22591
11	H	0.22346
12	C	0.08391
13	C	-0.38917
14	C	-0.24993
15	C	-0.0317
16	C	-0.03014
17	C	-0.201
18	C	-0.20244
19	C	0.01677
20	H	0.22624
21	H	0.22616
22	I	1.25174
23	C	-0.60892
24	H	0.23019
25	H	0.2296
26	H	0.23048
27	C	-0.61026
28	H	0.22903
29	H	0.23125
30	H	0.23088
31	C	-0.60517
32	H	0.22983
33	H	0.22327
34	H	0.21971
35	F	-0.59267
36	F	-0.58028
37	F	-0.58633
38	F	-0.57998
39	B	1.36473

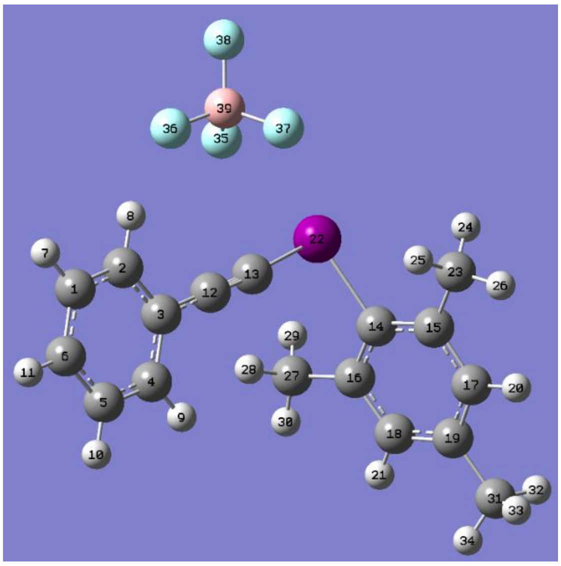

Alkynyl Iodonium species – Ag(I) Coordination

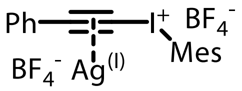

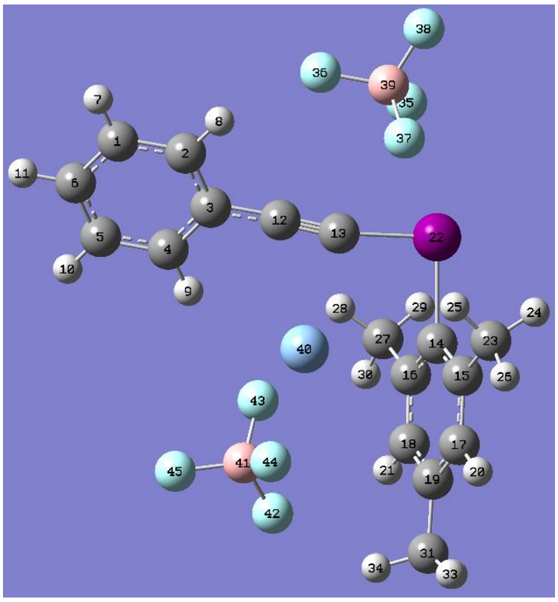

Atom No.	Atom	Charge
1	C	-0.21177
2	C	-0.15474
3	C	-0.19093
4	C	-0.15209
5	C	-0.2119
6	C	-0.17653
7	H	0.23319
8	H	0.25334
9	H	0.24118
10	H	0.23426
11	H	0.23083
12	C	0.1083
13	C	-0.42494
14	C	-0.19835
15	C	-0.03358
16	C	-0.02573
17	C	-0.21026
18	C	-0.1939
19	C	0.04617
20	H	0.23245
21	H	0.23834
22	I	1.1781
23	C	-0.63344
24	H	0.23683
25	H	0.23456
26	H	0.23947
27	C	-0.63212
28	H	0.23481
29	H	0.23458
30	H	0.24031
31	C	-0.63565
32	H	0.2315
33	H	0.22233
34	H	0.24332
35	F	-0.59377
36	F	-0.5802
37	F	-0.58294
38	F	-0.57593
39	B	1.35995
40	Ag	0.88492
41	B	1.36728
42	F	-0.56576
43	F	-0.58199
44	F	-0.59342
45	F	-0.56607

Alkynyl-iodonium formation

Pathways for the putative alkynyl-iodonium formation step (**A**) were calculated under differing hydrogen-bonding environments (**B**)

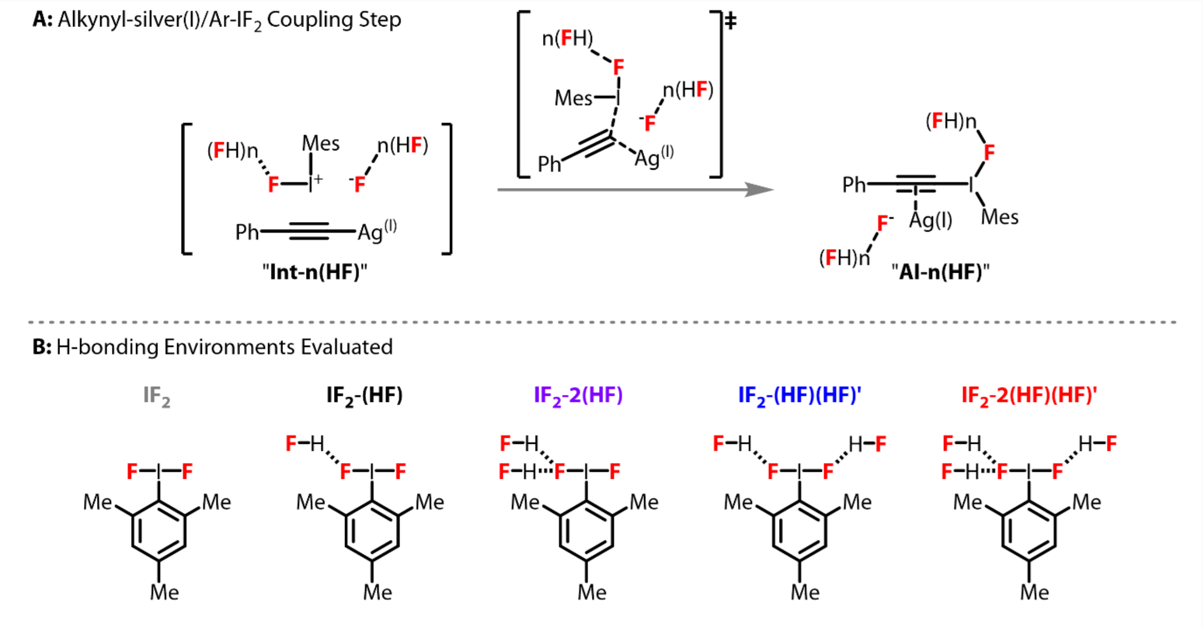

Def2-TZVP

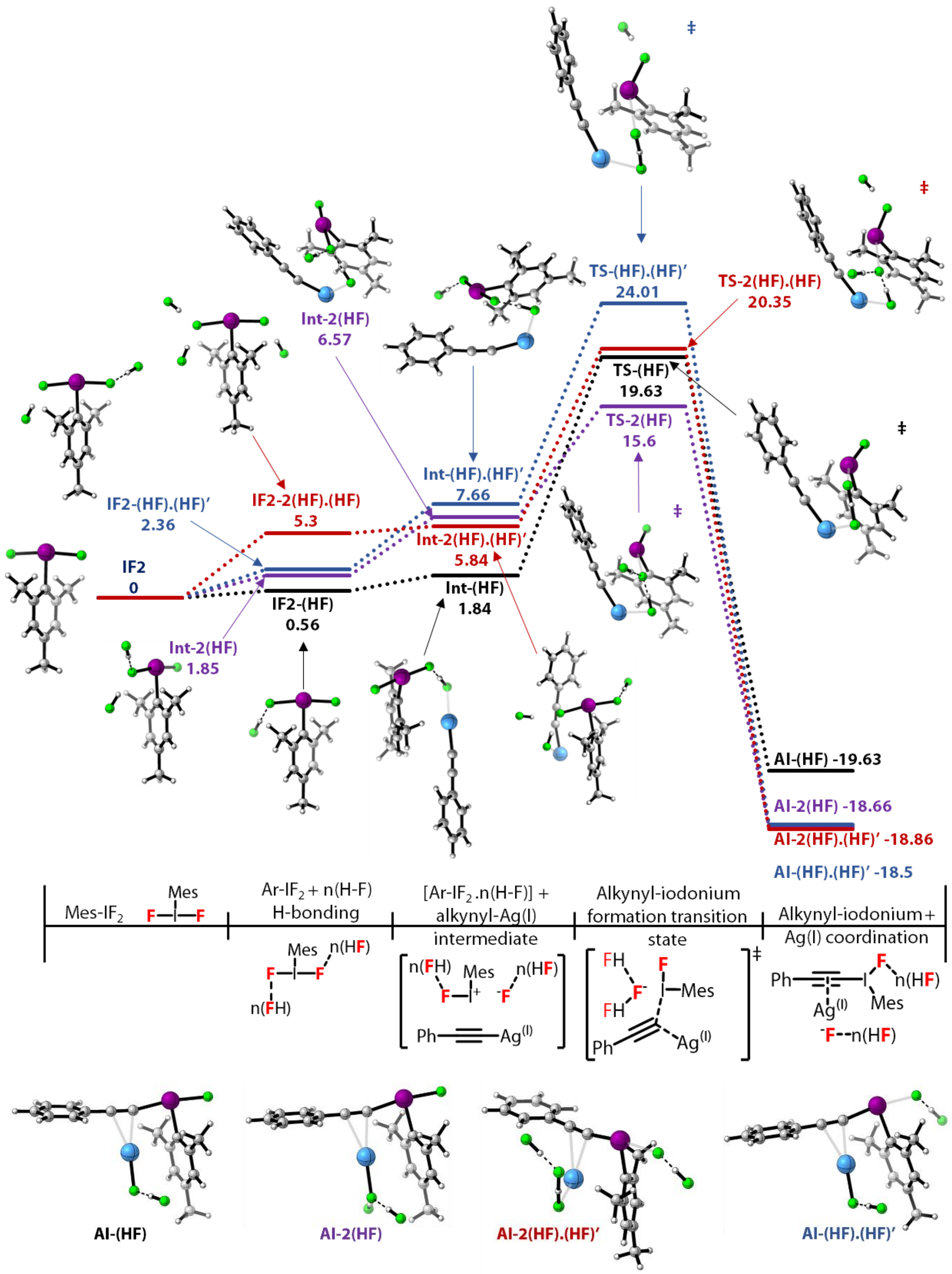

TS-(HF).(HF)′
24.01
TS-2(HF).(HF)
20.35
TS-(HF)
19.63
TS-2(HF)
15.6
Int-2(HF)
6.57
Int-(HF).(HF)′
7.66
Int-2(HF).(HF)′
5.84
Int-(HF)
1.84
IF2-(HF).(HF)′
2.36
IF2-2(HF).(HF)
5.3
IF2
0
IF2-(HF)
0.56
Int-2(HF)
1.85
AI-(HF) -19.63
AI-2(HF) -18.66
AI-2(HF).(HF)′ -18.86
AI-(HF).(HF)′ -18.5
Mes-IF2
Ar-IF2 + n(H-F) H-bonding
[Ar-IF2.n(H-F)] + alkynyl-Ag(I) intermediate
Alkynyl-iodonium formation transition state
Alkynyl-iodonium + Ag(I) coordination
AI-(HF)
AI-2(HF)
AI-2(HF).(HF)′
AI-(HF).(HF)′

Aug-cc-pVTZ, aug-cc-pVTZ-pp on I and Ag

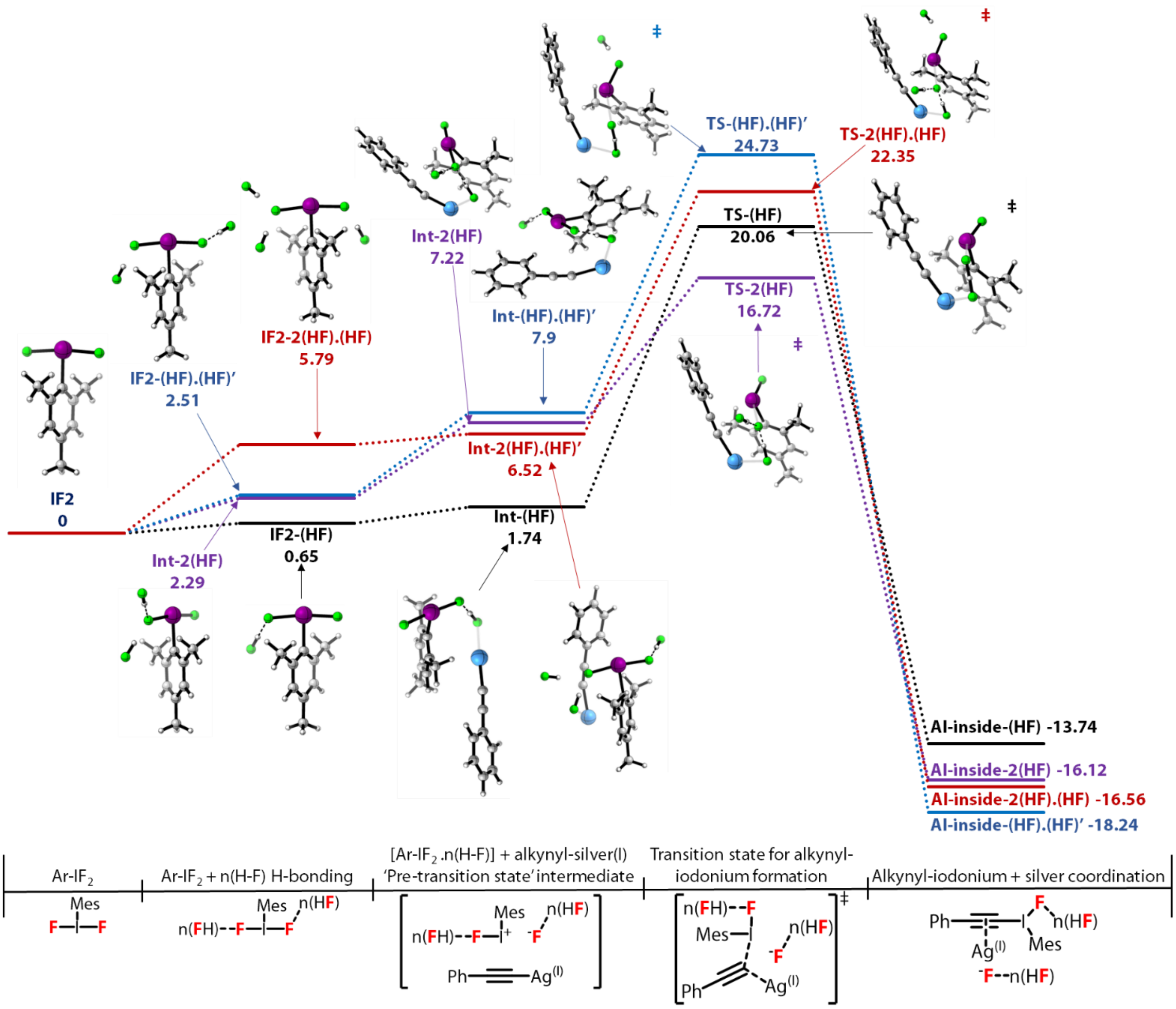

IF_2

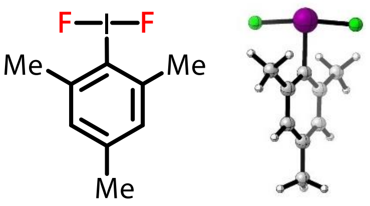

Thermal correction to the Gibbs free energy:
0.135550
Electronic energies,
Def2TZVP: **-846.841771407**
Aug-cc-pVTZ (-pp on **I**):
-844.837161511
Charge = 0 Multiplicity = 1

C	-3.81887	-0.06977	0.0662
C	-2.42163	-0.10757	0.05153
C	-1.76999	1.1277	-0.00218
C	-2.41929	2.36453	-0.04491
C	-3.8168	2.33019	-0.03982
C	-4.52926	1.13098	0.01542
H	-4.3612	-1.01053	0.1145
H	-4.35748	3.27257	-0.07225
I	0.35688	1.12551	-0.0124
F	0.22972	0.34107	1.86122
F	0.21192	1.91105	-1.88436
C	-1.70142	-1.4298	0.09752
H	-1.08959	-1.57409	-0.79865
H	-1.04444	-1.4895	0.96941
H	-2.42167	-2.24714	0.14903
C	-1.69658	3.68527	-0.0941
H	-1.07599	3.82632	0.79654
H	-1.04775	3.7454	-0.97204
H	-2.41541	4.50435	-0.13728
C	-6.03515	1.13094	-0.00549
H	-6.43539	2.04645	0.43551
H	-6.39995	1.07012	-1.03607
H	-6.43669	0.27418	0.54009

IF$_2$.(HF)

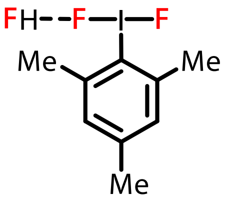

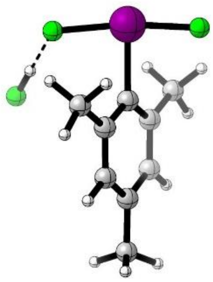

Thermal correction to the Gibbs free energy:
0.143682
Electronic energies,
Def2TZVP: **-947.313127628**
Aug-cc-pVTZ (-pp on I): **-945.309815199**
Charge = 0 Multiplicity = 1

C	1.049494	0.838975	-0.89414
C	2.445497	0.843687	-0.8325
C	3.160105	-0.1096	-0.10441
C	2.454434	-1.09845	0.583792
C	1.058366	-1.15666	0.574734
C	0.407316	-0.17452	-0.177
H	2.984185	1.614713	-1.37701
H	2.999635	-1.84883	1.150242
I	-1.71761	-0.22245	-0.22066
C	0.324861	1.888493	-1.69557
H	-0.28275	1.433301	-2.48382
H	-0.3366	2.483594	-1.05969
H	1.042915	2.559575	-2.1679
C	0.340186	-2.23515	1.342813
H	-0.30062	-1.80345	2.118332
H	-0.28627	-2.84152	0.682883
H	1.062213	-2.89132	1.829928
C	4.663537	-0.05744	-0.04251
H	4.985691	0.558765	0.803258
H	5.086991	-1.05505	0.092195
H	5.080095	0.381401	-0.95169
F	-1.57478	-1.96719	-1.18815
F	-1.58134	1.608305	0.797251
H	-0.43386	1.963816	1.70217
F	0.298995	2.186188	2.275776

int-(HF)

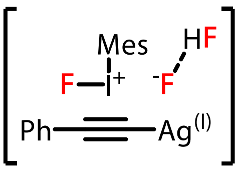

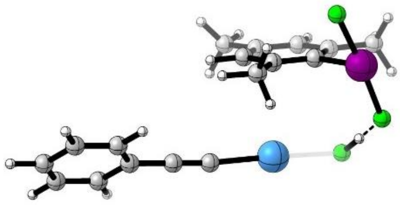

Thermal correction to the Gibbs free energy:
0.228010
Electronic energies,
Def2TZVP: **-1402.04615434**
Aug-cc-pVTZ (-pp on I and Ag): -
1399.98325082
Charge = 0 Multiplicity = 1

C	-5.58063	1.558051	-1.39864
C	-4.38458	0.97837	-0.98324
C	-3.64914	0.152508	-1.85205
C	-4.14801	-0.07511	-3.14663
C	-5.34453	0.50733	-3.55755
C	-6.0661	1.325515	-2.68669
H	-6.13551	2.192852	-0.71462
H	-4.00714	1.158684	0.01882
H	-5.71511	0.3211	-4.5609
H	-3.58793	-0.71176	-3.82453
H	-6.99859	1.777921	-3.00876
C	-2.41395	-0.44472	-1.4193
C	-1.36076	-0.94779	-1.03315
C	3.394975	0.733258	0.857461
C	3.358907	1.114603	-0.48782
C	2.189942	1.568318	-1.1001
C	1.017614	1.641909	-0.34343
C	0.973633	1.258077	0.998281
C	2.183061	0.816295	1.548793
H	4.278403	1.058501	-1.06395
H	0.09693	1.979274	-0.81208
I	2.163326	0.244563	3.595926
C	4.690485	0.279992	1.477829
H	4.971892	0.929089	2.31297
H	4.620028	-0.7436	1.855273
H	5.490199	0.317004	0.737639
C	-0.32509	1.316987	1.758212
H	-0.60857	0.323195	2.122235
H	-0.25454	1.991149	2.616356
H	-1.12183	1.667372	1.101081
C	2.172161	1.955611	-2.5543
H	1.554188	1.254375	-3.12437
H	1.742048	2.952208	-2.68508
H	3.177385	1.950978	-2.97933
F	1.686858	2.114997	4.05121
F	2.685583	-1.70661	2.869575
Ag	0.479701	-1.64728	-0.26968
H	2.503112	-2.15895	1.61958
F	2.366832	-2.47333	0.681049

TS-AI-(HF)

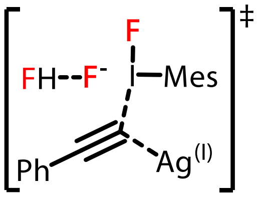

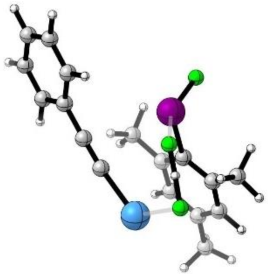

Thermal correction to the Gibbs free energy:
0.226898
Electronic energies,
Def2TZVP: **-1402.01680920**
Aug-cc-pVTZ (-pp on I and Ag): **-1399.95306321**
Charge = 0 Multiplicity = 1

C	-1.04777	-0.7473	0
C	0.25568	-1.20589	-0.17176
C	1.03365	-0.75206	-1.25214
C	0.47284	0.16991	-2.15405
C	-0.83228	0.62111	-1.97899
C	-1.59574	0.16592	-0.90212
H	-1.63748	-1.1054	0.83791
H	0.68217	-1.92262	0.5242
H	1.06797	0.52189	-2.9914
H	-1.25467	1.33022	-2.68388
H	-2.61252	0.52064	-0.76763
C	2.38404	-1.21178	-1.41357
C	3.55767	-1.57487	-1.53188
C	6.70705	0.37777	0.5036
C	7.7586	0.86066	-0.28077
C	7.54554	1.70201	-1.37381
C	6.23767	2.09019	-1.68474
C	5.13911	1.63883	-0.95652
C	5.42059	0.7759	0.11489
H	8.77266	0.57334	-0.01748
H	6.06524	2.76709	-2.51731
I	3.7848	0.05311	1.23546
C	7.00015	-0.50524	1.68758
H	6.60295	-0.07207	2.61015
H	6.56427	-1.50023	1.56179
H	8.07851	-0.61894	1.80415
C	3.75173	2.09746	-1.31285
H	3.11293	1.24116	-1.54844
H	3.29453	2.65212	-0.48743
H	3.78543	2.75348	-2.18331
C	8.69723	2.18606	-2.21152
H	8.71955	1.65085	-3.16627
H	8.5961	3.25105	-2.43474
H	9.65118	2.02108	-1.70771
F	3.59735	1.77339	2.15769
F	4.33079	-2.57744	1.42341
Ag	5.52964	-2.43287	-1.40318
H	5.24298	-3.12108	1.02684
F	6.16935	-3.58906	0.55913

AI-(HF)

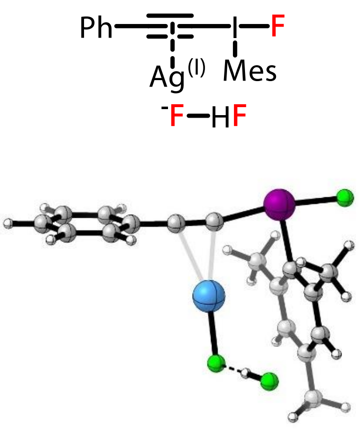

Thermal correction to G: **0.228316**
Electronic energies,
Def2TZVP: **-1402.07177777**
Aug-cc-pVTZ (-pp on I and Ag): -**1400.00813519**
Charge = 0 Multiplicity = 1

C	-5.0482	2.03974	0.460611
C	-3.81649	1.408963	0.599303
C	-2.71238	2.134846	1.071853
C	-2.85028	3.491362	1.401343
C	-4.08793	4.111953	1.261303
C	-5.18543	3.389258	0.791278
H	-5.90122	1.478789	0.093934
H	-3.69825	0.360872	0.342952
H	-1.9903	4.045122	1.763205
H	-4.19523	5.160646	1.517372
H	-6.1481	3.877794	0.681286
C	-1.43702	1.488904	1.229815
C	-0.34729	0.956942	1.410398
C	2.415558	-0.86567	-0.61198
C	2.925337	-0.67069	-1.89743
C	3.272894	0.595175	-2.37539
C	3.121615	1.695757	-1.53233
C	2.622507	1.573485	-0.22943
C	2.272642	0.282741	0.174267
H	3.03766	-1.53617	-2.54475
H	3.398982	2.685	-1.88662
I	1.458962	0.02846	2.141558
C	2.036892	-2.2458	-0.14435
H	2.560282	-2.50241	0.781207
H	0.959427	-2.31328	0.04216
H	2.292868	-2.98239	-0.90671
C	2.479128	2.794444	0.640484
H	1.429719	2.982675	0.89207
H	3.03381	2.682921	1.576699
H	2.863656	3.671494	0.11901
C	3.782114	0.758014	-3.78286
H	2.973395	0.583268	-4.49973
H	4.173807	1.763207	-3.94928
H	4.573207	0.034864	-3.99767
F	3.34415	-0.86751	2.550382
F	-0.41941	0.26564	-3.12849
Ag	-0.47137	0.687214	-0.994
H	-0.04772	-0.92586	-3.40777
F	0.23109	-1.88938	-3.63065

IF₂.(HF).(HF)'

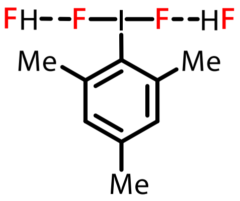

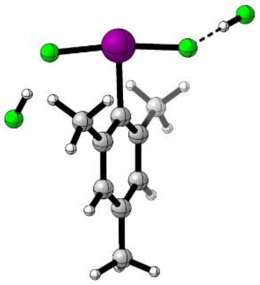

Thermal correction to the Gibbs free energy:
0.150572
Electronic energies,
Def2TZVP: -1047.78128440
Aug-cc-pVTZ (-pp on I):
-1045.77932387
Charge = 0 Multiplicity = 1

C	-3.12876	1.03943	-0.22692
C	-2.18559	1.633304	0.61478
C	-0.86321	1.188497	0.679198
C	-0.53582	0.118916	-0.1598
C	-1.42638	-0.52193	-1.02649
C	-2.73461	-0.03168	-1.03108
H	-2.48311	2.463456	1.249802
H	-3.46145	-0.50598	-1.68497
I	1.448008	-0.6334	-0.07067
C	-1.05188	-1.68473	-1.90774
H	-0.24556	-1.41566	-2.59668
H	-0.71981	-2.54175	-1.31423
H	-1.91271	-1.99532	-2.50037
C	0.113883	1.853359	1.612584
H	0.506861	1.142763	2.346297
H	0.95907	2.281038	1.065987
H	-0.37909	2.659938	2.155967
C	-4.53917	1.562193	-0.2831
H	-4.84732	1.967464	0.683092
H	-4.61336	2.367605	-1.02109
H	-5.23938	0.776745	-0.57476
F	0.608124	-2.29788	0.790161
F	2.024499	1.128498	-0.95642
F	-1.59856	-2.25473	2.005515
H	-0.76744	-2.27844	1.551033
F	3.579159	2.910845	-0.14493
H	3.005031	2.227513	-0.46616

int-(HF).(HF)'

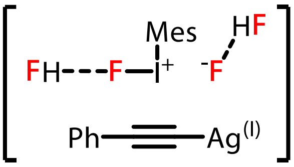

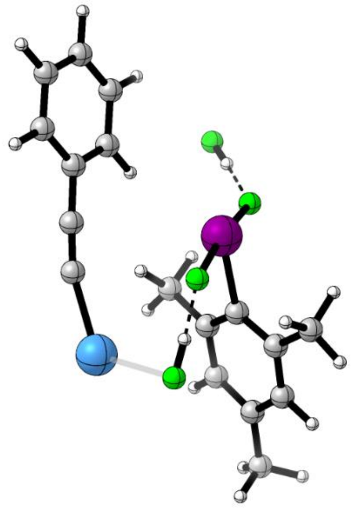

Thermal correction to the Gibbs free energy:
0.236572
Electronic energies,
Def2TZVP: **-1502.50959645**
Aug-cc-pVTZ (-pp on I and Ag):
-1500.44760714
Charge = 0 Multiplicity = 1

C	-5.66424	-1.08733	0.105845
C	-4.41742	-1.70617	0.057466
C	-3.30598	-1.02959	-0.47396
C	-3.48202	0.279537	-0.95765
C	-4.72926	0.896348	-0.90262
C	-5.82573	0.214856	-0.37167
H	-6.51318	-1.62335	0.519023
H	-4.29198	-2.71715	0.432543
H	-2.63588	0.808985	-1.38712
H	-4.83753	1.909359	-1.27701
H	-6.79867	0.694084	-0.33018
C	-2.00406	-1.6379	-0.51478
C	-0.85708	-2.0809	-0.54441
C	2.668204	1.518373	1.180491
C	3.925073	1.488747	0.574112
C	4.086505	1.250363	-0.7933
C	2.950992	1.06057	-1.58093
C	1.659054	1.077788	-1.04351
C	1.574499	1.286438	0.33686
H	4.801444	1.660819	1.193574
H	3.061091	0.901436	-2.65026
I	-0.35329	1.213531	1.229902
C	2.552454	1.801929	2.6558
H	1.919086	2.675506	2.838248
H	2.122535	0.956551	3.199522
H	3.538985	2.007066	3.072215
C	0.466651	0.928587	-1.94932
H	-0.22857	0.171002	-1.57348
H	-0.06596	1.880439	-2.03881
H	0.790678	0.624497	-2.94567
C	5.46422	1.196697	-1.39737
H	5.94796	0.245525	-1.15326
H	5.423268	1.286143	-2.48446
H	6.095049	1.997059	-1.00284
F	-0.8224	2.863186	0.105363
F	0.373045	-0.39243	2.311492
Ag	1.24871	-2.39934	-0.33728
H	1.621801	-1.17848	2.036639
F	2.394692	-1.68966	1.784116
H	-1.95428	3.204422	-0.91535
F	-2.61195	3.389685	-1.57201

TS-AI-(HF).(HF)’

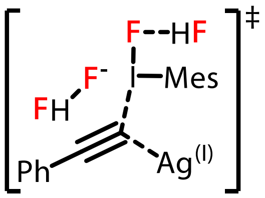

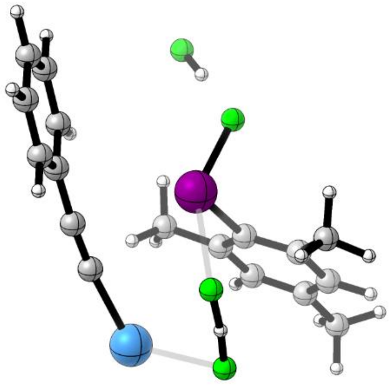

Thermal correction to the Gibbs free energy:
0.236799
Electronic energies,
Def2TZVP: -1502.48386551
Aug-cc-pVTZ (-pp on I and Ag):
-1500.42112658
Charge = 0 Multiplicity = 1

C	-5.51468	-0.87822	0.382584
C	-4.30204	-1.52859	0.185069
C	-3.33663	-0.97714	-0.67753
C	-3.61261	0.241153	-1.32872
C	-4.82708	0.890498	-1.11734
C	-5.77961	0.331923	-0.26556
H	-6.25565	-1.31179	1.046423
H	-4.08726	-2.46403	0.691616
H	-2.8718	0.662332	-2.00298
H	-5.02984	1.829475	-1.62228
H	-6.72684	0.836619	-0.10501
C	-2.0604	-1.59842	-0.86341
C	-0.90791	-2.01397	-1.03604
C	2.357058	1.136354	1.262893
C	3.687854	1.376428	0.927987
C	4.121288	1.436002	-0.40075
C	3.185613	1.282406	-1.42569
C	1.832198	1.053692	-1.16687
C	1.470323	0.966967	0.186636
H	4.40691	1.519629	1.730126
H	3.510005	1.353158	-2.46012
I	-0.55036	0.587973	0.618468
C	1.93469	1.098101	2.706467
H	1.216191	1.894588	2.925009
H	1.468499	0.138641	2.944108
H	2.801463	1.241413	3.352465
C	0.858649	0.949523	-2.31212
H	0.301206	0.007578	-2.28878
H	0.133735	1.769557	-2.28348
H	1.394877	1.007805	-3.25984
C	5.576107	1.654636	-0.71299
H	6.133381	0.722375	-0.57415
H	5.715782	1.981324	-1.74498
H	6.01165	2.40063	-0.04393
F	-0.88317	2.567049	0.828154
F	0.650754	-1.51243	1.633735

Ag	1.157519	-2.64682	-1.06614
H	1.694405	-1.866	1.230093
F	2.616285	-2.234	0.749474
H	-1.66562	3.457945	-0.29438
F	-2.11773	3.928329	-0.97258

AI-(HF).(HF)'

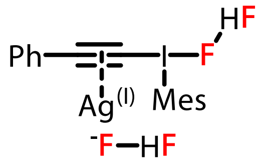

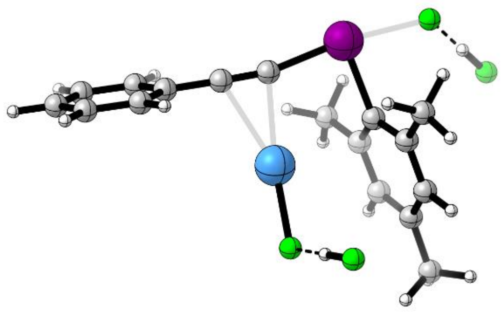

Thermal correction to the Gibbs free energy:
0.234230
Electronic energies,
Def2TZVP: **-1502.54878281**
Aug-cc-pVTZ (-pp on I and Ag):
-1500.48676276
Charge = 0 Multiplicity = 1

C	0.645239	0.025802	-0.25899
C	-0.7136	0.244588	-0.45639
C	-1.45737	0.91072	0.529626
C	-0.83847	1.352527	1.708828
C	0.522244	1.130645	1.89312
C	1.262807	0.468411	0.912569
H	1.222992	-0.48957	-1.01862
H	-1.2057	-0.09416	-1.36236
H	-1.42528	1.864672	2.464069
H	1.004506	1.47336	2.802214
H	2.323724	0.296389	1.061755
C	-2.85702	1.159902	0.332537
C	-4.04635	1.405412	0.185674
C	-7.53161	-0.35498	-0.23945
C	-8.10296	-1.51282	0.294837
C	-7.99987	-1.83756	1.64934
C	-7.3225	-0.9644	2.501307
C	-6.73279	0.218071	2.040845
C	-6.85167	0.459624	0.670203
H	-8.63609	-2.18049	-0.37669
H	-7.24963	-1.19678	3.560339
I	-5.94054	2.247012	-0.10382
C	-7.66536	-0.04459	-1.70602
H	-8.15667	0.921497	-1.85558
H	-6.68486	-0.00904	-2.19222
H	-8.25739	-0.81669	-2.19822
C	-6.02715	1.1364	3.003063
H	-4.96086	1.221722	2.7671
H	-6.45955	2.141178	2.980747
H	-6.11629	0.750483	4.018718
C	-8.58896	-3.12072	2.171071
H	-7.90801	-3.95436	1.969697
H	-8.75166	-3.07184	3.249493
H	-9.53952	-3.34436	1.681499
F	-8.13124	2.871952	-0.31378
F	-5.90504	-3.87627	-1.61834
Ag	-4.23344	-1.04678	0.201251
H	-5.33805	-3.57377	-0.81628
F	-4.62491	-3.18944	0.171452
H	-9.13351	2.328752	0.413754
F	-9.87264	1.933682	0.950853

IF_2.2(HF)

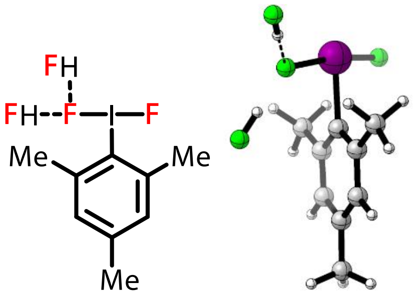

Thermal correction to the Gibbs free energy:
0.149730
Electronic energies,
Def2TZVP: **-1047.78124302**
Aug-cc-pVTZ (-pp on I and Ag):
-1045.77883436
Charge = 0 Multiplicity = 1

C	3.320325	0.392468	-0.19711
C	2.421865	1.032758	-1.05373
C	1.061853	0.715767	-1.07633
C	0.651938	-0.27767	-0.18139
C	1.493699	-0.95922	0.702362
C	2.84282	-0.59916	0.662028
H	2.784493	1.801209	-1.73119
H	3.534063	-1.10878	1.327774
I	-1.41151	-0.77882	-0.13977
C	1.020744	-2.02407	1.656677
H	0.298061	-1.6171	2.371134
H	0.544963	-2.85319	1.125348
H	1.864743	-2.42138	2.221224
C	0.1325	1.437719	-2.01613
H	-0.367	0.739603	-2.69466
H	-0.63728	1.98664	-1.46605
H	0.693742	2.151662	-2.61957
C	4.773432	0.784707	-0.18314
H	5.104146	1.105292	-1.17341
H	4.930572	1.620257	0.506801
H	5.40278	-0.04403	0.147479
F	-0.97123	-2.56232	-0.87104
F	-1.60924	1.228247	0.657655
F	-3.52996	2.769955	0.230289
H	-2.80164	2.185814	0.402105
F	0.290615	2.197387	1.993304
H	-0.42726	1.840784	1.48672

int-2(HF)

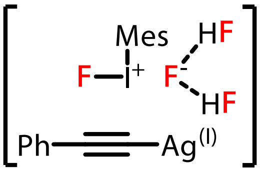

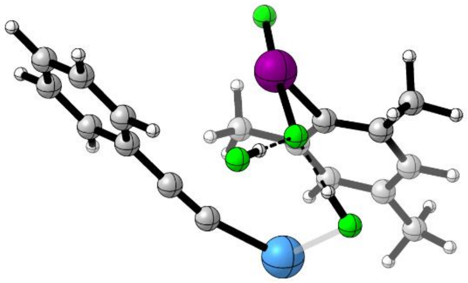

Thermal correction to the Gibbs free energy:
0.235026
Electronic energies,
Def2TZVP: **-1502.50976975**
Aug-cc-pVTZ (-pp on I and Ag):
-1500.44714926
Charge = 0 Multiplicity = 1

C	-0.17265	-0.4414	-0.01893
C	1.108449	-0.88951	0.295564
C	1.891505	-1.54438	-0.67203
C	1.354946	-1.7359	-1.9582
C	0.07367	-1.28489	-2.26602
C	-0.69569	-0.63635	-1.29846
H	-0.76527	0.062147	0.738859
H	1.516128	-0.73804	1.290664
H	1.953709	-2.2407	-2.71063
H	-0.32505	-1.44059	-3.26379
H	-1.69411	-0.28571	-1.53976
C	3.227449	-1.98289	-0.36956
C	4.394396	-2.31412	-0.16167
C	7.324287	1.357911	-0.18296
C	8.579675	0.813061	-0.47109
C	8.766449	-0.12741	-1.48504
C	7.665237	-0.51107	-2.25975
C	6.38233	-0.00048	-2.03425
C	6.261341	0.899024	-0.96711
H	9.430032	1.134172	0.12409
H	7.803691	-1.21677	-3.07509
I	4.303869	1.542343	-0.45763
C	7.185762	2.383056	0.91199
H	6.756259	3.311089	0.522401
H	6.543431	2.02872	1.722122
H	8.16599	2.6121	1.330936
C	5.242446	-0.3919	-2.93408
H	4.406242	-0.79291	-2.35297
H	4.891699	0.473081	-3.50382
H	5.568259	-1.1624	-3.63441
C	10.13296	-0.68659	-1.77678
H	10.0707	-1.73205	-2.0873
H	10.60227	-0.1259	-2.59166
H	10.78312	-0.61653	-0.90267
F	4.145541	2.428391	-2.21933
F	4.714322	0.56252	1.443443
Ag	6.549996	-2.39603	-0.04274
H	5.958109	-0.27512	1.786384
F	6.753057	-0.74913	2.02403
H	3.633266	0.017834	2.405768
F	2.98844	-0.29515	3.030655

TS-AI-2(HF)

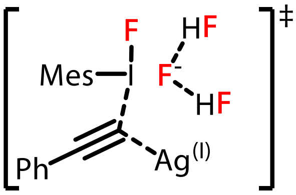

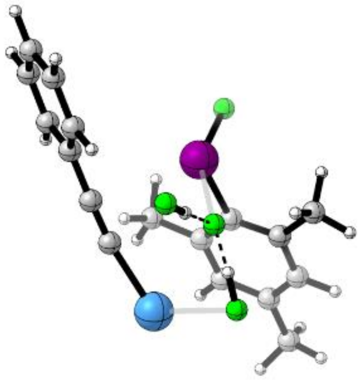

Thermal correction to the Gibbs free energy:
0.236528
Electronic energies,
Def2TZVP: **-1502.49693904**
Aug-cc-pVTZ (-pp on I and Ag):
-1500.43356767
Charge = 0 Multiplicity = 1

C	-3.74426	0.053903	-0.000058
C	-5.01499	0.619641	-0.06015
C	-5.84299	0.379643	-1.17179
C	-5.37503	-0.44622	-2.20955
C	-4.10303	-1.00928	-2.14052
C	-3.28361	-0.75831	-1.03877
H	-3.1111	0.245359	0.860696
H	-5.38992	1.240817	0.748026
H	-6.0166	-0.63606	-3.06511
H	-3.75063	-1.64354	-2.94781
H	-2.29199	-1.19659	-0.98776
C	-7.16376	0.939363	-1.23204
C	-8.32132	1.362024	-1.29363
C	-11.5414	-1.47283	0.594665
C	-12.8728	-1.29827	0.20925
C	-13.2406	-1.09432	-1.12102
C	-12.2473	-1.09546	-2.10749
C	-10.8995	-1.2749	-1.80097
C	-10.5921	-1.42615	-0.4361
H	-13.6399	-1.32076	0.978082
H	-12.5297	-0.96673	-3.14906
I	-8.55892	-1.5811	0.082863
C	-11.2077	-1.72303	2.040929
H	-10.7377	-2.7036	2.166185
H	-10.5224	-0.96566	2.428304
H	-12.1195	-1.70808	2.638754
C	-9.87623	-1.33284	-2.90362
H	-9.10882	-0.56292	-2.77522
H	-9.38396	-2.31033	-2.92539
H	-10.362	-1.17929	-3.8679
C	-14.6842	-0.91707	-1.50403
H	-14.7968	-0.11496	-2.23758
H	-15.0679	-1.8356	-1.95975
H	-15.3002	-0.68742	-0.63296
F	-8.53963	-3.52497	-0.1754
F	-9.11889	0.558715	1.534736
Ag	-10.378	1.979664	-1.06554
H	-10.263	1.18742	1.428108
F	-11.1437	1.666143	1.268267
H	-7.99252	1.28972	1.938137
F	-7.20294	1.774242	2.259025

AI-2(HF)

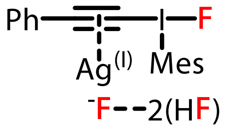

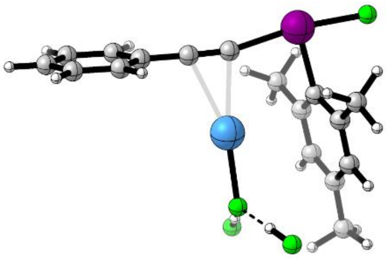

Thermal correction to the Gibbs free energy:
0.236995
Electronic energies,
Def2TZVP: **-1502.55179956**
Aug-cc-pVTZ (-pp on I and Ag):
-1500.48837113
Charge = 0 Multiplicity = 1

C	-5.54686	-1.064	-0.77046
C	-4.16458	-1.17687	-0.8780
C	-3.38234	-1.2625	0.284012
C	-3.99105	-1.2309	1.547705
C	-5.37447	-1.11871	1.643373
C	-6.15217	-1.0352	0.487264
H	-6.15232	-0.99862	-1.6682
H	-3.68365	-1.19944	-1.85021
H	-3.37681	-1.29569	2.439816
H	-7.23085	-0.94725	0.5663
H	-5.84575	-1.09599	2.620191
C	-1.95447	-1.39879	0.18375
C	-0.74014	-1.55399	0.119017
C	2.450794	0.31033	-1.03424
C	2.809812	1.661424	-0.9644
C	2.659941	2.409884	0.202444
C	2.164582	1.779489	1.347454
C	1.793568	0.431908	1.352838
C	1.932268	-0.24846	0.135775
H	3.197886	2.139509	-1.85922
H	2.052367	2.351507	2.264638
I	1.28815	-2.29023	0.047814
C	2.615909	-0.45582	-2.31972
H	3.232171	-1.3463	-2.16572
H	1.645581	-0.77775	-2.71226
H	3.092536	0.173077	-3.0724
C	1.275306	-0.20637	2.61557
H	0.243971	-0.55661	2.497979
H	1.88927	-1.06522	2.902924
H	1.294501	0.5159	3.432265
C	3.000834	3.874761	0.23276
H	2.082639	4.467181	0.297792
H	3.6111	4.11621	1.107342
H	3.540695	4.173948	-0.66742
F	3.373638	-2.70883	-0.0286
F	-0.44152	3.029938	-0.60374
Ag	-0.78423	0.847901	-0.30533
H	-0.27312	3.65567	0.619116
F	-0.17411	4.094762	1.495057
H	0.332327	3.456676	-1.68126
F	0.84798	3.769124	-2.45647

IF_2.2(HF).(HF)’

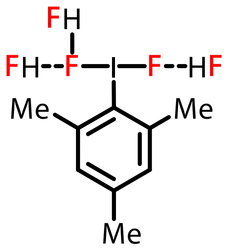

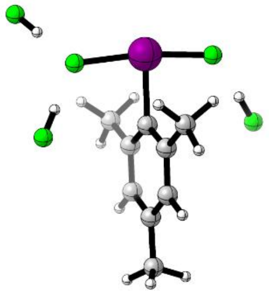

Thermal correction to the Gibbs free energy:
0.158079
Electronic energies,
Def2TZVP: **-1148.24824813**
Aug-cc-pVTZ (-pp on I):
-1146.24718608
Charge = 0 Multiplicity = 1

C	2.904486	0.806951	0.141037
C	4.107702	1.008363	8.21E-01
C	4.77186	2.236913	0.80703
C	4.162927	3.249644	0.059553
C	2.964768	3.11808	-0.65058
C	2.352058	1.865743	-0.5836
H	4.548735	0.189294	1.38182
H	1.418934	1.717569	-1.12028
I	5.124933	5.138727	-0.0049
F	6.584005	4.137001	-1.15578
F	3.590843	5.844869	1.110911
C	2.200101	-0.52114	0.207934
H	2.906894	-1.33411	0.385626
H	1.475894	-0.52107	1.029135
H	1.654472	-0.72368	-0.71615
C	6.068863	2.40472	1.553592
H	6.889943	2.651299	0.873846
H	5.995674	3.199393	2.302167
H	6.323879	1.478711	2.069428
C	2.334935	4.228837	-1.44912
H	2.099428	5.091092	-0.81814
H	3.000242	4.564437	-2.25064
H	1.407565	3.880495	-1.90428
H	8.165878	4.39417	-1.12342
H	6.17208	2.813687	-2.00147
H	2.664387	4.882527	2.03655
F	5.916835	2.043993	-2.48203
F	9.09771	4.537763	-1.09079
F	2.134516	4.297195	2.550862

int-2(HF).(HF)'

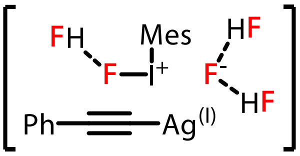

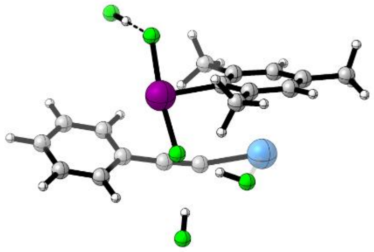

Thermal correction to the Gibbs free energy:
0.244173
Electronic energies,
Def2TZVP: **-1602.98419711**
Aug-cc-pVTZ (-pp on I):
-1600.92296712
Charge = 0 Multiplicity = 1

C	-0.94496	2.057432	0.204045
C	0.327641	1.52264	1.93E-02
C	0.970319	1.639634	-1.22518
C	0.311879	2.300023	-2.27575
C	-0.95901	2.835853	-2.0818
C	-1.5907	2.717861	-0.84277
H	-1.43187	1.962127	1.169489
H	0.837218	1.017341	0.833989
H	0.80635	2.393111	-3.23803
H	-1.45603	3.347929	-2.8997
H	-2.58048	3.137356	-0.6941
C	2.310125	1.146775	-1.40556
C	3.483331	0.799032	-1.55197
C	5.998398	4.007886	0.686603
C	7.299334	3.736683	0.254711
C	7.601951	3.496528	-1.0869
C	6.568491	3.557171	-2.03021
C	5.242861	3.837763	-1.6691
C	5.007877	4.022533	-0.30231
H	8.093469	3.704882	0.99555
H	6.79577	3.411046	-3.08376
I	2.992773	4.21863	0.337792
C	5.732112	4.254115	2.148224
H	5.227182	5.21306	2.298564
H	5.104124	3.469457	2.579012
H	6.673359	4.276258	2.69798
C	4.190767	3.964376	-2.73658
H	3.275311	3.4292	-2.4693
H	3.94503	5.017921	-2.89743
H	4.559425	3.54835	-3.67551
C	9.016521	3.221563	-1.52031
H	9.581981	2.728469	-0.72709
H	9.039455	2.593533	-2.41347
H	9.524409	4.161558	-1.7593
F	2.929969	5.970812	-0.74464
F	3.301106	2.470626	1.361997
Ag	5.662542	0.838444	-1.5502
H	4.552205	1.511942	1.19294
F	5.372552	1.032261	1.184925
H	2.878699	-0.2898	-0.08381
F	2.732017	-0.91122	0.628415
H	1.973654	6.212911	-1.93515
F	1.396459	6.327045	-2.68106

TS-AI-2(HF).(HF)'

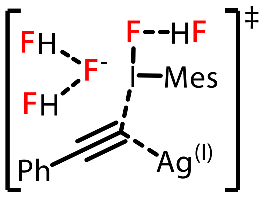

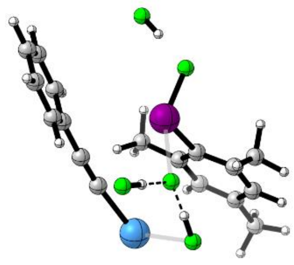

Thermal correction to the Gibbs free energy:
0.245616
Electronic energies,
Def2TZVP: **-1602.96117610**
Aug-cc-pVTZ (-pp on I):
-1600.89928037
Charge = 0 Multiplicity = 1

C	-5.29309	-1.10634	0.350323
C	-3.9919	-1.57058	1.84E-01
C	-3.13256	-0.94539	-0.73742
C	-3.60037	0.159079	-1.47342
C	-4.90266	0.620625	-1.29602
C	-5.7522	-0.01254	-0.38822
H	-5.95144	-1.59486	1.061743
H	-3.61657	-2.40509	0.767925
H	-2.93578	0.643531	-2.18341
H	-5.25385	1.47331	-1.86841
H	-6.76736	0.346698	-0.25292
C	-1.77702	-1.38583	-0.89829
C	-0.58597	-1.6801	-1.04621
C	2.372753	1.118667	1.27226
C	3.713788	1.146971	0.882551
C	4.097002	1.186589	-0.45853
C	3.106224	1.241517	-1.44681
C	1.748356	1.227189	-1.13527
C	1.43048	1.132347	0.233326
H	4.47647	1.134162	1.655848
H	3.398148	1.310585	-2.49136
I	-0.60003	0.960507	0.737393
C	2.017686	1.103406	2.734719
H	1.453006	2.000981	3.00609
H	1.411251	0.230543	2.987558
H	2.927015	1.083186	3.335932
C	0.722435	1.348447	-2.2304
H	0.035654	0.496205	-2.22978
H	0.135116	2.264915	-2.11487
H	1.21971	1.388604	-3.20012
C	5.54925	1.17289	-0.84785
H	5.813938	0.200549	-1.27563
H	5.755863	1.931703	-1.60677
H	6.193373	1.351929	0.014538
F	-0.83154	2.951324	0.750792
F	0.166401	-1.24897	1.810912
Ag	1.538196	-2.1135	-1.02461
H	1.370434	-1.75605	1.588799
F	2.278985	-2.12381	1.351359
H	-0.88448	-2.18927	2.031947
F	-1.60134	-2.81578	2.23844
H	-1.8381	3.795457	-0.26031
F	-2.41194	4.231923	-0.86199

AI-2(HF).(HF)’

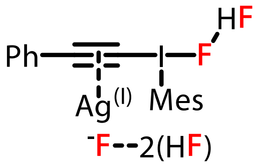

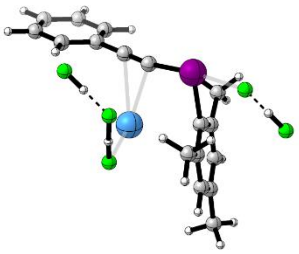

Thermal correction to the Gibbs free energy:
0.247176
Electronic energies,
Def2TZVP: **-1603.02496660**
Aug-cc-pVTZ (-pp on I and Ag):
-1600.96259953
Charge = 0 Multiplicity = 1

C	-4.79604	-2.61781	-0.43485
C	-3.85786	-1.77335	0.151371
C	-3.38332	-0.66789	-0.57144
C	-3.84607	-0.41355	-1.8718
C	-4.78269	-1.26692	-2.44524
C	-5.25711	-2.36753	-1.72855
H	-5.16796	-3.47369	0.118506
H	-3.48574	-1.94767	1.156715
H	-3.46888	0.445188	-2.41792
H	-5.14273	-1.07424	-3.45022
H	-5.98766	-3.03116	-2.17981
C	-2.41554	0.21159	0.021253
C	-1.58909	0.965019	0.508572
C	2.34355	1.234605	1.174195
C	3.463368	0.753857	0.485808
C	3.55702	0.787281	-0.90504
C	2.505428	1.345772	-1.63694
C	1.35803	1.856212	-1.02023
C	1.316147	1.742108	0.376385
H	4.283858	0.343111	1.067974
H	2.579272	1.410052	-2.71927
I	-0.44327	2.466728	1.381826
C	2.311034	1.214128	2.67745
H	2.274462	2.236115	3.066813
H	1.434466	0.67295	3.045339
H	3.206227	0.723278	3.061272
C	0.288153	2.518733	-1.85017
H	-0.67252	1.998765	-1.77998
H	0.13436	3.553631	-1.529
H	0.588269	2.529488	-2.89826
C	4.763533	0.227017	-1.60979
H	4.575934	-0.80737	-1.91569
H	4.995395	0.802066	-2.50902
H	5.637893	0.22924	-0.95602
F	1.068119	4.04885	2.137444
F	-0.20106	-0.79527	2.955309
Ag	0.062257	-0.85172	-0.33209
H	0.56154	-1.37776	2.203785
F	1.185768	-1.82784	1.469581
H	-1.48086	-1.24063	3.006406
F	-2.41532	-1.57602	3.051119
H	2.166249	4.362714	1.441015
F	2.986118	4.597793	0.916858

Stereo- and Regio-selectivity Study

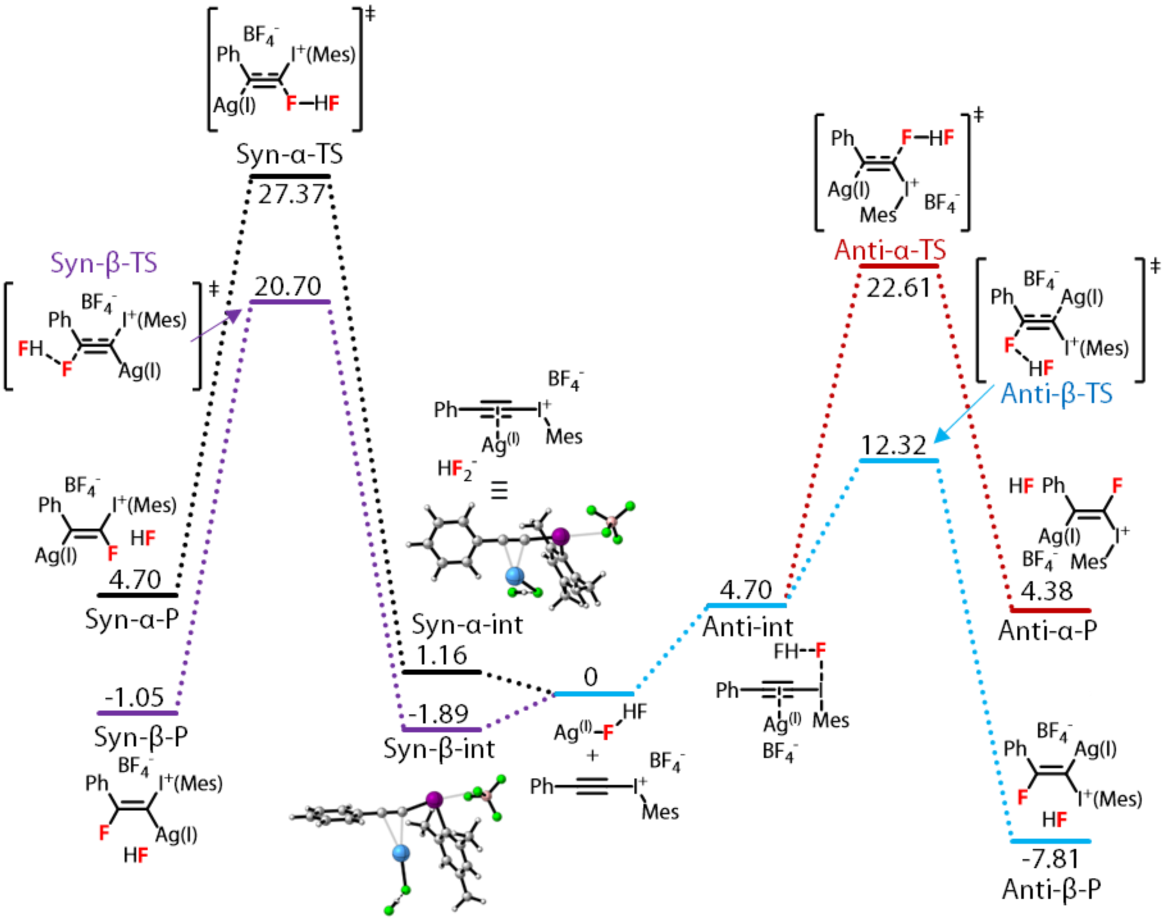

Anti-, α Pathway

Anti-int

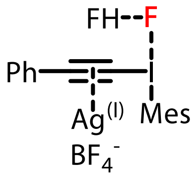

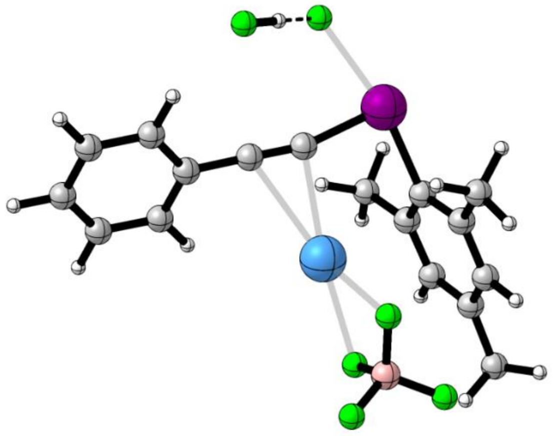

Thermal correction to the Gibbs free energy:
0.237887
Electronic energies,
Def2TZVP: **-1439.95123962**
Aug-cc-pVTZ (-pp on I): **-1724.66150604**
Charge = 0 Multiplicity = 1

H	-13.9598	-2.57983	-1.37047
H	-13.1308	-1.10973	-1.88246
F	-12.4595	-0.16509	0.414098
F	-10.3146	-0.45572	1.214264
F	-10.6279	0.243382	-0.92841
F	-11.1291	1.658791	0.814605
B	-11.1645	0.334007	0.390226
Ag	-8.55264	-0.95998	-0.45817
F	-5.00889	-4.78653	0.414523
H	-4.35479	-3.90393	1.020334
F	-3.82062	-3.16472	1.523489

C	-3.07415	0.747279	-0.16824
C	-3.91076	-0.34701	0.029425
C	-5.03375	-0.50417	-0.7979
C	-5.31444	0.422213	-1.8146
C	-4.47151	1.512722	-1.99404
C	-3.3537	1.675409	-1.17247
H	-2.20177	0.87607	0.463523
H	-3.70953	-1.09206	0.793487
H	-6.18518	0.282274	-2.44804
H	-4.68472	2.234947	-2.7746
H	-2.69814	2.527909	-1.3176
C	-5.88362	-1.63976	-0.60202
C	-6.6199	-2.59338	-0.41366
C	-9.34452	-3.72788	-0.85151
C	-10.3091	-3.53377	0.148767
C	-9.56779	-3.47738	-2.21429
C	-11.553	-3.05542	-0.26886
C	-10.8317	-2.9932	-2.56092
C	-11.8286	-2.77329	-1.60778
H	-12.3168	-2.87248	0.481217
H	-11.0351	-2.77917	-3.60683
I	-7.40665	-4.45345	-0.26404
C	-10.056	-3.77977	1.614264
H	-9.80682	-4.82592	1.81402
H	-9.23436	-3.15721	1.984006
H	-10.947	-3.52812	2.190025
C	-8.52213	-3.68524	-3.27974
H	-7.64193	-3.05615	-3.10468
H	-8.18935	-4.72652	-3.31773
H	-8.92812	-3.4238	-4.25729
C	-13.1583	-2.19701	-2.00632
H	-13.3925	-2.42199	-3.04886

Anti-α-TS

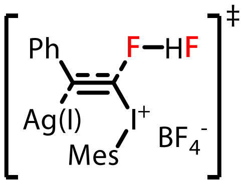

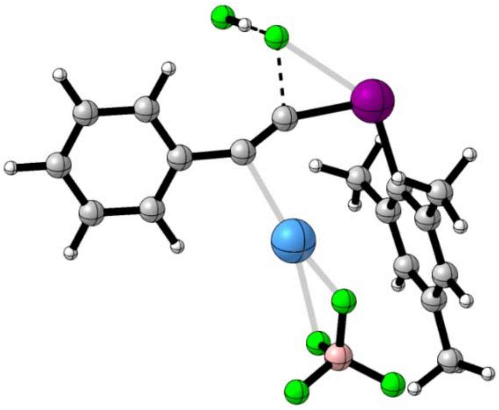

Thermal correction to the Gibbs free energy:
0.236803
Electronic energies,
Def2TZVP: **-1726.70019145**
Aug-cc-pVTZ (-pp on I): **-1724.63185732**
Charge = 0 Multiplicity = 1

C	5.027575	2.493838	0.300144
C	4.124815	1.447598	0.459121
C	2.844852	1.530297	-0.11384
C	2.488883	2.669191	-0.8484
C	3.40078	3.70934	-1.01197
C	4.668012	3.625324	-0.43551
H	6.013945	2.425646	0.747259
H	4.393671	0.55916	1.021444
H	1.50488	2.7327	-1.30523
H	3.119514	4.585313	-1.58695
H	5.375482	4.438749	-0.56001
C	1.928138	0.415106	0.063815
C	1.801733	-0.78133	0.379829
C	-1.14248	-1.74458	-0.21982
C	-2.15486	-1.41994	0.694995
C	-1.24917	-1.61284	-1.61293
C	-3.33865	-0.92315	0.145237
C	-2.46021	-1.10475	-2.08775
C	-3.50543	-0.75182	-1.23009
H	-4.1405	-0.63666	0.818642
H	-2.58009	-0.97562	-3.15998
I	0.690617	-2.52081	0.556128
C	-2.02023	-1.56315	2.18834
H	-1.88293	-2.60846	2.480338
H	-1.16934	-0.98962	2.568623
H	-2.91992	-1.18833	2.676439
C	-0.14141	-1.95724	-2.57286
H	0.756646	-1.36259	-2.37166
H	0.131035	-3.01447	-2.50778
H	-0.45795	-1.75073	-3.59543
C	-4.76957	-0.14911	-1.77415
H	-4.97595	-0.50555	-2.78555
H	-5.62337	-0.37712	-1.13274
H	-4.66177	0.939892	-1.80579
F	-4.33874	1.94774	0.645116
F	-2.25764	1.667286	1.601175
F	-2.41634	2.154145	-0.61421
F	-2.96191	3.75693	0.944119
B	-3.02538	2.397776	0.653406
Ag	-0.36571	1.060051	0.085814
F	3.251142	-1.86165	0.875422
H	4.101263	-1.88264	-0.22042
F	4.682275	-1.89437	-1.01511

Anti-α-P

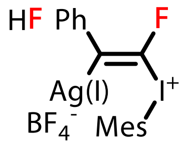

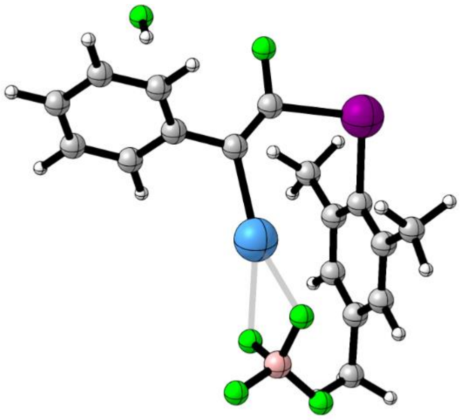

Thermal correction to the Gibbs free energy:
0.238186
Electronic energies,
Def2TZVP: **-1726.73050529**
Aug-cc-pVTZ (-pp on I): **-1724.66127174**
Charge = 0 Multiplicity = 1

F	-11.5705	-0.37893	1.43842
F	-11.6533	0.151203	-0.77122
F	-12.2403	1.727733	0.79851
B	-12.2988	0.373134	0.484014
Ag	-9.68366	-1.05952	-0.13703
F	-6.05294	-3.70364	0.041091
H	-4.39487	-1.82592	-1.16738
F	-3.96438	-2.27311	-1.86622

C	-4.4489	0.213575	0.242815
C	-5.4383	-0.74957	0.459921
C	-6.5386	-0.85494	-0.41034
C	-6.61772	0.028547	-1.49836
C	-5.61764	0.971453	-1.72412
C	-4.53091	1.072505	-0.85325
H	-3.61245	0.284271	0.930946
H	-5.37057	-1.41089	1.318837
H	-7.47206	-0.03225	-2.16685
H	-5.69268	1.636675	-2.57849
H	-3.75907	1.81534	-1.02421
C	-7.61178	-1.85873	-0.18462
C	-7.25075	-3.09111	0.010925
C	-10.3983	-3.93161	-0.48426
C	-11.4026	-3.52903	0.408185
C	-10.4674	-3.79597	-1.8761
C	-12.5402	-2.957	-0.16696
C	-11.6343	-3.21126	-2.37934
C	-12.6713	-2.78632	-1.54722
H	-13.3333	-2.61081	0.489175
H	-11.7222	-3.08125	-3.45463
I	-8.63641	-4.80647	0.340135
C	-11.3041	-3.66402	1.905355
H	-11.2267	-4.71219	2.209029
H	-10.4286	-3.13398	2.294208
H	-12.1912	-3.23583	2.372618
C	-9.36554	-4.21144	-2.81273
H	-8.44911	-3.6454	-2.61132
H	-9.1343	-5.27579	-2.71236
H	-9.66118	-4.0216	-3.84498
C	-13.8908	-2.11379	-2.11374
H	-14	-2.32152	-3.18003
H	-14.7952	-2.43963	-1.5941
H	-13.8041	-1.0319	-1.97345
F	-13.6157	-0.06807	0.420051

Anti-, β Pathway

Anti-β-TS

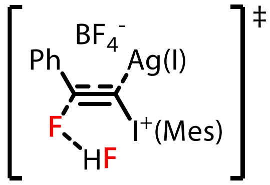

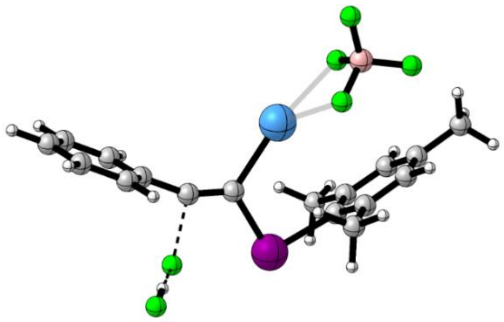

C	0.27307	-0.04601	0.183685
C	-0.85204	-0.85628	0.113949
C	-1.40413	-1.16332	-1.14285
C	-0.82402	-0.66523	-2.32337
C	0.301622	0.14329	-2.23996
C	0.846906	0.450463	-0.9903
H	0.70466	0.200649	1.147364
H	-1.31324	-1.25325	1.012054
H	-1.26197	-0.91999	-3.2825
H	0.755636	0.53491	-3.14343
H	1.726469	1.083391	-0.93054
C	-2.56093	-1.98558	-1.22064
C	-3.80379	-2.25812	-1.24187
C	-6.49008	-3.70089	-1.34479
C	-7.13543	-3.77069	-0.10695
C	-7.07687	-3.20503	-2.51844
C	-8.45483	-3.30588	-0.06987
C	-8.39006	-2.74744	-2.40654
C	-9.09113	-2.78886	-1.19804
H	-8.98899	-3.34293	0.875672
H	-8.87249	-2.3332	-3.28753
I	-4.42227	-4.23933	-1.42402
C	-6.48622	-4.28521	1.152017
H	-6.1248	-5.31016	1.031466
H	-5.63462	-3.66098	1.44183
H	-7.20443	-4.27435	1.972548
C	-6.36435	-3.12731	-3.84409
H	-5.48951	-2.46937	-3.79545
H	-6.02429	-4.11347	-4.1735
H	-7.03693	-2.72547	-4.60233
C	-10.501	-2.26889	-1.12898
H	-10.9175	-2.38148	-0.12628
H	-10.5173	-1.21046	-1.40403
H	-11.1444	-2.8043	-1.83344
Ag	-5.43119	-0.71696	-1.12263
B	-7.79591	0.828509	-2.27456

Thermal correction to the Gibbs free energy:
0.233494
Electronic energies,
Def2TZVP: **-1726.71319905**
Aug-cc-pVTZ (-pp on I): **-1724.64541299**
Charge = 0 Multiplicity = 1

F	-9.0879	0.388496	-2.53364
F	-6.86441	0.154919	-3.09732
F	-7.44005	0.505992	-0.92315
F	-7.68932	2.204279	-2.45563
F	-1.67545	-3.80978	-1.39329
H	-1.0898	-3.88331	-2.57452
F	-0.66386	-3.92712	-3.48717

Anti-β-P

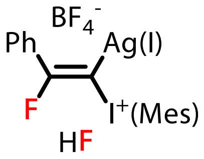

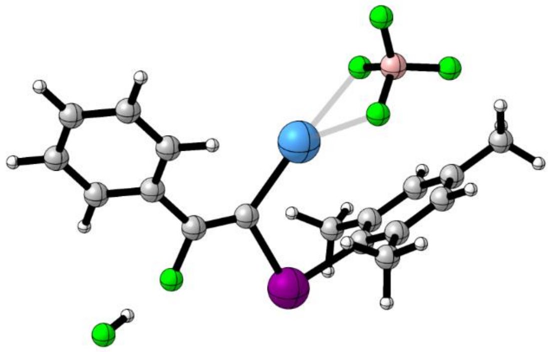

Thermal correction to the Gibbs free energy:
0.238991
Electronic energies,
Def2TZVP: **-1726.75065916**
Aug-cc-pVTZ (-pp on I): **-1724.68180752**
Charge = 0 Multiplicity = 1

C	-1.19082	0.105859	-0.62263
C	-2.04854	-0.99002	-0.67919
C	-3.25211	-0.9018	-1.39147
C	-3.57768	0.287041	-2.05716
C	-2.7203	1.381413	-1.989
C	-1.52616	1.294304	-1.27175
H	-0.26159	0.031175	-0.06736
H	-1.79036	-1.90905	-0.16249
H	-4.49046	0.342133	-2.64283
H	-2.97816	2.297897	-2.50947
H	-0.85609	2.146575	-1.226
C	-4.16317	-2.05946	-1.4284
C	-5.49404	-2.02447	-1.47503
C	-8.32526	-3.27932	-1.53059
C	-8.99059	-3.22886	-0.3033
C	-8.83809	-2.77471	-2.7336
C	-10.2588	-2.63601	-0.31208
C	-10.1019	-2.18523	-2.66618
C	-10.826	-2.11194	-1.47326
H	-10.8079	-2.57935	0.624054
H	-10.5248	-1.76156	-3.57321
I	-6.33292	-4.05459	-1.55426
C	-8.41274	-3.74667	0.988057
H	-8.17409	-4.81205	0.92421
H	-7.49323	-3.21297	1.24943
H	-9.12771	-3.60657	1.799642
C	-8.09906	-2.82482	-4.04563
H	-7.15055	-2.27894	-3.99314
H	-7.87539	-3.85561	-4.33633
H	-8.70444	-2.36909	-4.82979
C	-12.1874	-1.47086	-1.45672
H	-12.6072	-1.45753	-0.44908
H	-12.1191	-0.44423	-1.82657
H	-12.8765	-2.01535	-2.10941
Ag	-7.01121	-0.43199	-1.47644
B	-9.19912	1.368324	-2.60659
F	-10.5506	1.091027	-2.77273
F	-8.41385	0.553771	-3.44984
F	-8.80852	1.050345	-1.26213
F	-8.93104	2.712669	-2.84844
F	-3.47354	-3.26445	-1.37349
H	-2.00894	-3.54409	-2.37734
F	-1.24343	-3.69275	-2.88864

Syn-, β Pathway

Syn-β-int

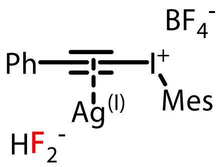

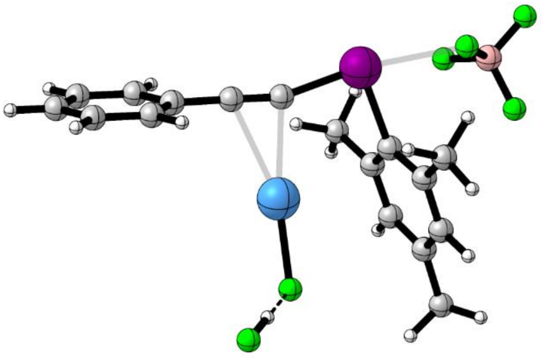

Thermal correction to the Gibbs free energy:
0.234560
Electronic energies,
Def2TZVP: **-1726.73550556**
Aug-cc-pVTZ (-pp on I): **-1724.66856088**
Charge = 0 Multiplicity = 1

C	-1.75502	-0.31231	1.235213
C	-2.24627	-1.6097	1.408027
C	-2.70579	-2.38061	0.337593
C	-2.68295	-1.83551	-0.94721
C	-2.20416	-0.5466	-1.20167
C	-1.74723	0.149553	-0.08301
C	-1.26449	0.483064	2.414506
H	-2.26847	-2.02042	2.413906
C	-3.18823	-3.78822	0.56313
H	-3.04789	-2.42253	-1.78565
C	-2.20673	0.00706	-2.60065
I	-0.94456	2.126197	-0.41765
H	-1.41743	-0.08322	3.333433
H	-1.80837	1.428072	2.494122
H	-0.19412	0.703549	2.332952
H	-2.62689	-0.72603	-3.28958
H	-1.19149	0.243263	-2.93582
H	-2.8095	0.918281	-2.6558
H	-3.63581	-3.89762	1.553189
H	-2.34694	-4.48617	0.497469
H	-3.92303	-4.07902	-0.19026
C	0.97693	1.501666	-0.1397
C	2.191141	1.4912	0.007329
C	3.609962	1.464242	0.185471
C	4.152947	1.669134	1.463824
H	3.493153	1.834036	2.308919
C	5.533444	1.659249	1.626802
H	5.960358	1.818551	2.610892
C	6.366327	1.442993	0.527096
H	7.443112	1.434415	0.660635
C	5.824205	1.23681	-0.74339
H	6.47592	1.068412	-1.59368
C	4.446063	1.246087	-0.92156
H	4.009438	1.085927	-1.90181
Ag	1.17905	-0.92154	-0.2814
B	-4.42348	2.209091	0.079907
F	-5.41553	3.18329	0.106627
F	-4.95635	0.953477	-0.19513
F	-3.7106	2.190072	1.288976
F	-3.47792	2.542115	-0.95553
F	1.029263	-3.08143	-0.51443
H	1.873134	-3.64203	-1.29202
F	2.555306	-4.08649	-1.91819

Syn-β-TS

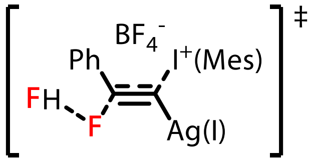

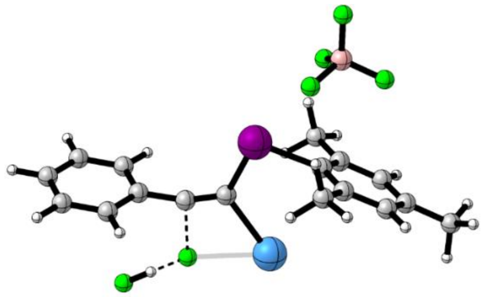

Thermal correction to the Gibbs free energy:
0.234996
Electronic energies,
Def2TZVP: **-1726.70142183**
Aug-cc-pVTZ (-pp on I): **-1724.63289730**
Charge = 0 Multiplicity = 1

C	0.335465	0.833998	-0.1226
C	-0.5473	-0.25062	-0.08842
C	-1.20599	-0.70902	-1.23105
C	-0.97592	-0.06164	-2.44661
C	-0.1052	1.026755	-2.55607
C	0.519832	1.426893	-1.37408
C	1.02128	1.295924	1.13577
H	-0.72446	-0.74135	0.865109
C	-2.1288	-1.89706	-1.15981
H	-1.48803	-0.40552	-3.34157
C	0.115036	1.693841	-3.88728
I	1.929229	3.038958	-1.49343
H	0.690275	0.69439	1.9831
H	0.784392	2.344132	1.337487
H	2.110008	1.199078	1.05906
H	-0.49303	1.211247	-4.65316
H	1.164005	1.625791	-4.19357
H	-0.15889	2.751585	-3.84108
H	-2.57159	-1.99294	-0.16619
H	-1.57695	-2.81909	-1.37013
H	-2.93194	-1.81568	-1.89549
C	3.543603	1.754636	-1.42948
C	4.77023	2.061057	-1.38314
C	5.982609	2.821335	-1.31283
C	6.455915	3.253021	-0.06414
H	5.91239	2.986036	0.836304
C	7.616005	4.016941	0.002046
H	7.985699	4.355611	0.96387
C	8.303724	4.343302	-1.16845
H	9.210254	4.93728	-1.1123
C	7.833523	3.912281	-2.41155
H	8.372351	4.170359	-3.3168
C	6.67439	3.149656	-2.48926
H	6.298914	2.80191	-3.4461
Ag	3.210963	-0.53167	-1.52596
B	-1.41718	4.198948	-0.83645
F	-2.06015	5.431659	-0.70145
F	-2.35001	3.177546	-1.02172
F	-0.62745	3.936443	0.298397
F	-0.54489	4.250079	-1.96377

F	5.603661	0.288158	-1.39382
H	6.93378	0.201609	-1.254
F	7.92626	0.125646	-1.14852

Syn-β-P

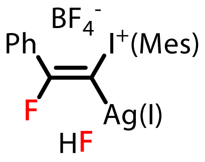

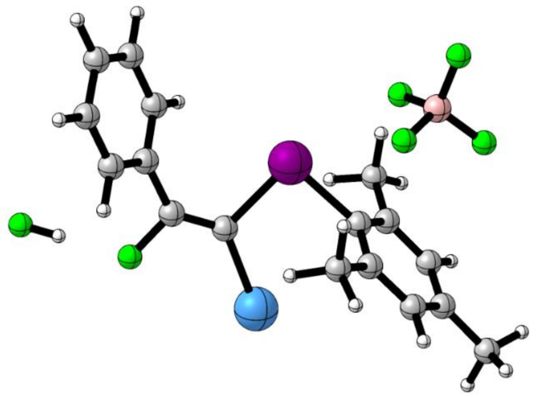

Thermal correction to the Gibbs free energy:
0.236115
Electronic energies,
Def2TZVP: **-1726.73839135**
Aug-cc-pVTZ (-pp on **I**): **-1724.66926084**
Charge = 0 Multiplicity = 1

C	2.486908	0.500774	1.158295
C	3.64582	1.242002	0.908137
C	3.997966	1.659328	-0.37698
C	3.165967	1.318245	-1.44447
C	1.994071	0.574936	-1.26607
C	1.696579	0.197032	0.046478
C	2.148756	0.078122	2.563251
H	4.288922	1.495208	1.747301
C	5.23789	2.485333	-0.59927
H	3.431943	1.629362	-2.45147
C	1.132388	0.223383	-2.44927
I	-0.12913	-0.85957	0.375908
H	2.919338	0.422662	3.254184
H	2.079511	-1.01099	2.632417
H	1.190678	0.500203	2.883269
H	1.581885	0.604769	-3.36712
H	0.130552	0.656498	-2.3548
H	1.028908	-0.8621	-2.5341
H	6.041282	2.176949	0.073792
H	5.030404	3.542704	-0.40461
H	5.591151	2.397909	-1.62883
C	-1.2891	0.951121	0.120757
C	-2.58359	0.725071	-0.08645
C	-3.43615	-0.47885	-0.1394
C	-3.42519	-1.4095	0.906277
H	-2.83004	-1.22094	1.796234
C	-4.21103	-2.55845	0.82841
H	-4.19881	-3.27438	1.643397
C	-5.02145	-2.77476	-0.28488
H	-5.63433	-3.66821	-0.34419
C	-5.05209	-1.83751	-1.31977
H	-5.68635	-2.00229	-2.18452
C	-4.26555	-0.69167	-1.24926
H	-4.28289	0.03553	-2.05553
Ag	-0.15084	2.840728	0.128168
B	2.789425	-3.09842	-0.18773
F	2.960165	-4.48833	-0.2517
F	4.036762	-2.46349	-0.14969
F	2.057205	-2.77223	0.980826
F	2.059913	-2.65686	-1.30774
F	-3.33095	1.872933	-0.35096
H	-5.00598	1.915889	0.151788
F	-5.89655	1.947922	0.433572

Syn-, α Pathway

Syn-α-int

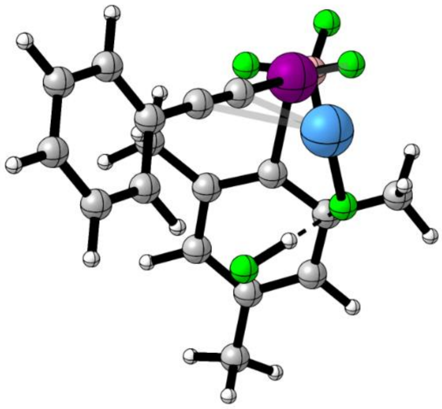

Thermal correction to the Gibbs free energy:
0.235793
Electronic energies,
Def2TZVP: **-1726.73322605**
Aug-cc-pVTZ (-pp on I): **-1724.66375191**
Charge = 0 Multiplicity = 1

C	-4.24734	1.272033	-0.1613
C	-4.74173	2.547266	0.132998
C	-5.29402	2.861621	1.375334
C	-5.37296	1.86446	2.349175
C	-4.89813	0.567098	2.132302
C	-4.33765	0.345819	0.87552
C	-3.67739	0.975269	-1.52103
H	-4.69224	3.30821	-0.64136
C	-5.78201	4.255951	1.667661
H	-5.82297	2.090448	3.312454
C	-5.03077	-0.48052	3.202764
I	-3.57462	-1.64243	0.492781
H	-3.82599	1.830713	-2.18083
H	-4.17298	0.107281	-1.96583
H	-2.6029	0.772944	-1.46817
H	-5.48707	-0.04399	4.092369
H	-4.04296	-0.86572	3.470052
H	-5.66482	-1.3037	2.861419
H	-6.07411	4.772216	0.750778
H	-4.98853	4.841617	2.143303
H	-6.63591	4.239602	2.348599
C	-1.63282	-1.10937	0.232698
C	-0.53913	-0.67483	-0.07204
C	0.745812	-0.12773	-0.40053
C	1.200295	0.998676	0.304088
H	0.565821	1.430119	1.074489
C	2.441419	1.54241	-0.01219
H	2.798783	2.414112	0.525744
C	3.223966	0.970261	-1.01686
H	4.191703	1.398162	-1.25768
C	2.766785	-0.14891	-1.71547
H	3.376118	-0.58961	-2.49726
C	1.526715	-0.70262	-1.41365
H	1.160135	-1.57078	-1.95102
Ag	-0.2528	-1.55296	2.5446
B	-7.05323	-1.4777	-0.19456
F	-6.17473	-2.14518	0.726991
F	-7.67579	-0.42139	0.466703
F	-7.99247	-2.3979	-0.65687
F	-6.26594	-1.00131	-1.25369
F	-1.97644	-0.19586	3.100881
H	-1.68539	0.907124	2.556722
F	-1.39281	1.79859	2.113198

Syn-α-TS

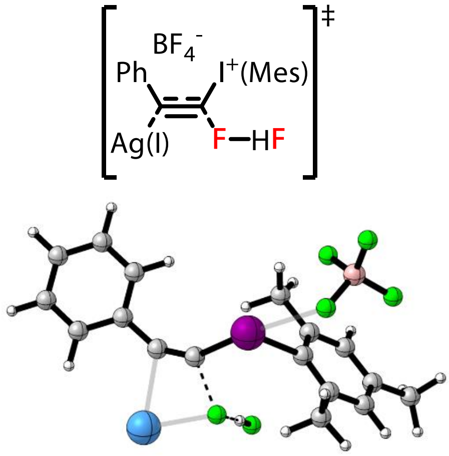

Thermal correction to the Gibbs free energy:
0.234885
Electronic energies,
Def2TZVP: **-1726.69071091**
Aug-cc-pVTZ (-pp on I): **-1724.62111927**
Charge = 0 Multiplicity = 1

C	-0.66644	-0.15086	0.156935
C	-1.5148	0.890145	-0.23174
C	-2.45261	1.448106	0.638869
C	-2.53598	0.956995	1.943107
C	-1.72406	-0.08616	2.398498
C	-0.82136	-0.59899	1.468065
C	0.350797	-0.70329	-0.80381
H	-1.42833	1.274239	-1.24494
C	-3.37154	2.547109	0.173947
H	-3.25464	1.390194	2.634001
C	-1.85138	-0.58778	3.811194
I	0.427251	-2.22543	2.112281
H	0.251201	-0.21815	-1.77553
H	0.213647	-1.78028	-0.93751
H	1.369421	-0.52551	-0.44063
H	-2.60531	-0.00988	4.347051
H	-0.90021	-0.49266	4.344146
H	-2.15035	-1.64006	3.824275
H	-2.89894	3.155234	-0.60048
H	-3.65925	3.197075	1.003214
H	-4.28732	2.121313	-0.24906
C	2.228691	-1.19882	2.233469
C	3.447081	-1.2003	1.989279
C	4.591982	-1.73108	1.272746
C	4.388244	-2.41005	0.058636
H	3.378789	-2.5247	-0.32465
C	5.469363	-2.92603	-0.64849
H	5.300036	-3.44076	-1.58869
C	6.765063	-2.79143	-0.14565
H	7.606625	-3.19991	-0.69567
C	6.975558	-2.13047	1.064347
H	7.980175	-2.02432	1.460297
C	5.898146	-1.59367	1.764928
H	6.067838	-1.07332	2.703728
Ag	3.925277	0.750146	3.435298
B	-2.68587	-3.39894	0.7285
F	-2.09718	-3.35027	2.02838
F	-3.66889	-2.4126	0.635624
F	-3.24203	-4.66557	0.530719
F	-1.66759	-3.16471	-0.21206
F	1.547949	0.115555	3.320658
H	1.240999	1.279651	2.530974
F	1.132614	2.087249	2.001007

Syn-α-P

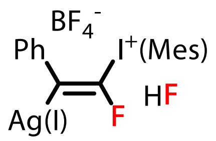

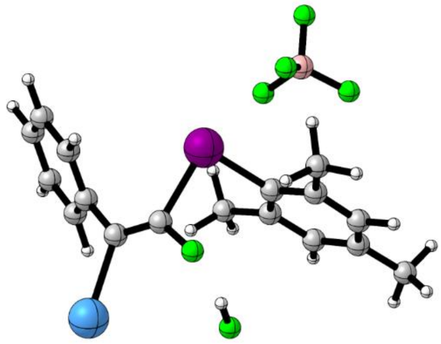

Thermal correction to the Gibbs free energy:
0.235952
Electronic energies,
Def2TZVP: **-1726.72700588**
Aug-cc-pVTZ (-pp on I): **-1724.65726409**
Charge = 0 Multiplicity = 1

C	0.233463	0.399118	0.196946
C	-0.78263	1.249802	-0.25084
C	-1.84238	1.637187	0.569602
C	-1.88362	1.161448	1.881668
C	-0.90595	0.302966	2.392837
C	0.12441	-0.04292	1.516647
C	1.365391	0.026581	-0.7213
H	-0.73191	1.620413	-1.2715
C	-2.93497	2.531586	0.044873
H	-2.70146	1.458832	2.533198
C	-1.00444	-0.1976	3.808991
I	1.668195	-1.34226	2.253555
H	1.192878	0.440111	-1.71594
H	1.454891	-1.06024	-0.806
H	2.319226	0.419919	-0.35139
H	-1.89295	0.212827	4.290513
H	-0.13049	0.098545	4.397588
H	-1.0722	-1.2893	3.826624
H	-2.57117	3.163679	-0.76806
H	-3.33292	3.172007	0.835354
H	-3.76346	1.930641	-0.34414
C	3.09189	0.343014	2.410368
C	4.267488	0.417361	1.857325
C	4.850919	-0.70145	1.106867
C	5.197493	-0.543	-0.2446
H	5.056292	0.425352	-0.71769
C	5.707002	-1.61285	-0.97404
H	5.954917	-1.4783	-2.02227
C	5.910148	-2.85173	-0.36034
H	6.31807	-3.68131	-0.92856
C	5.598667	-3.01155	0.988717
H	5.765632	-3.9661	1.47784
C	5.071555	-1.94596	1.718811
H	4.852559	-2.06906	2.77671
Ag	5.31014	2.388599	1.969634
B	-1.21841	-3.14069	0.780725
F	-0.7642	-3.00649	2.117279
F	-2.33316	-2.31792	0.584286
F	-1.562	-4.47917	0.543886
F	-0.17653	-2.7576	-0.08419
F	2.437664	1.285149	3.140409
H	1.937915	2.623259	2.003483
F	1.812544	3.21555	1.295228

Silver-free Fluorination of the Alkynyl-iodonium Species – Concerted Hydrofluorination

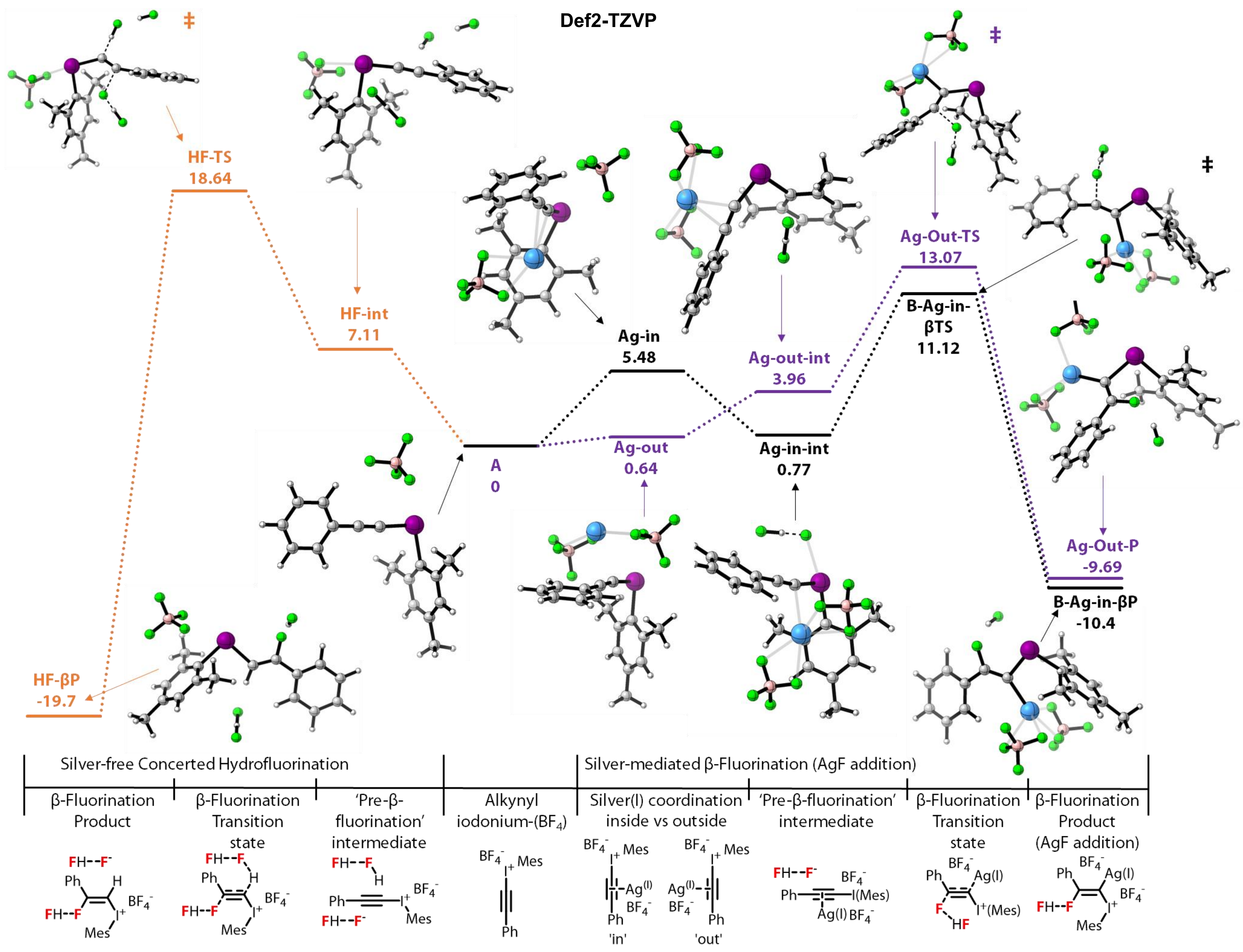

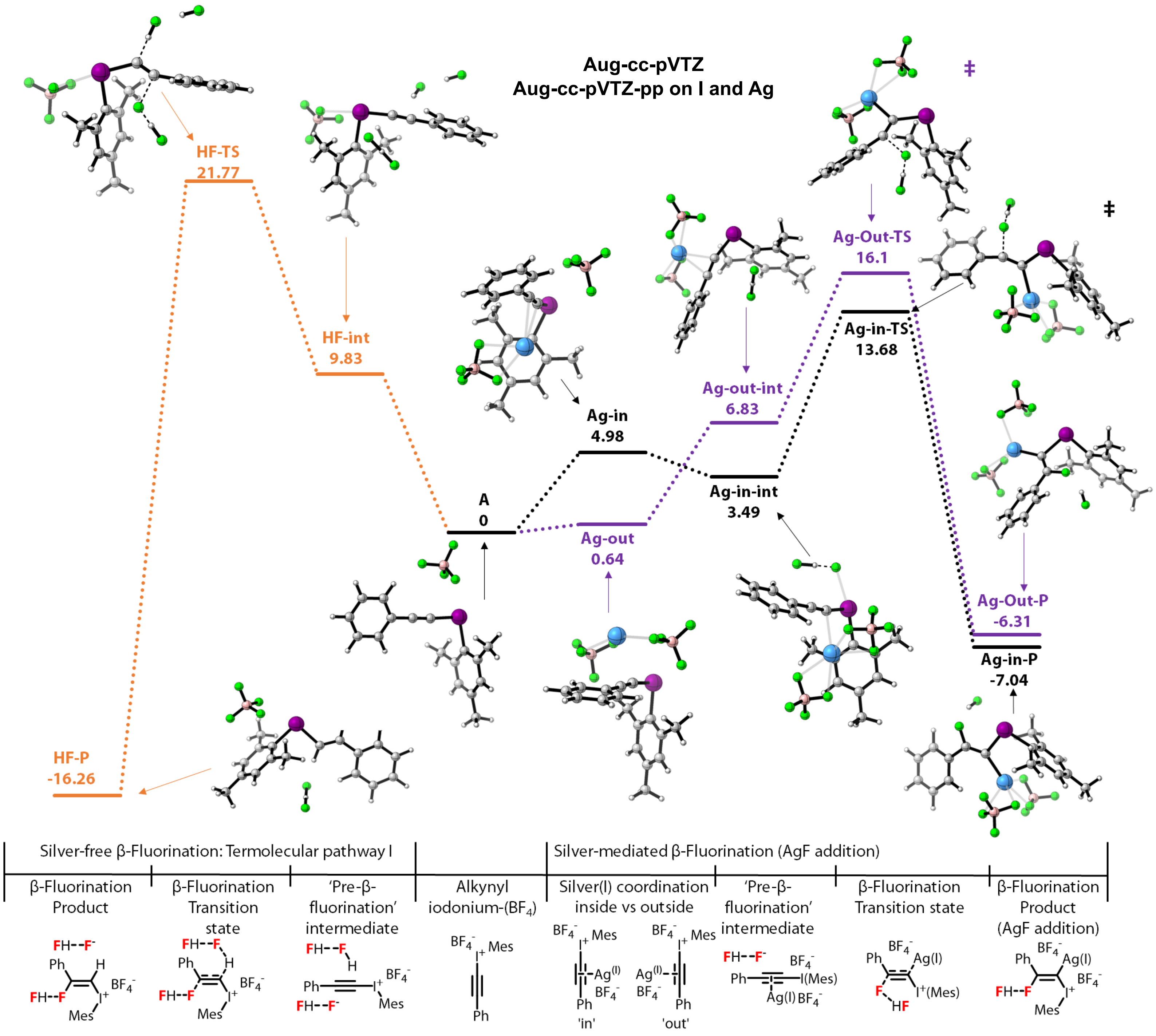

Aug-cc-pVTZ
Aug-cc-pVTZ-pp on I and Ag
HF-TS
21.77
HF-int
9.83
HF-P
-16.26
A
0
Ag-in
4.98
Ag-out
0.64
Ag-out-int
6.83
Ag-in-int
3.49
Ag-Out-TS
16.1
Ag-in-TS
13.68
Ag-Out-P
-6.31
Ag-in-P
-7.04
‡
‡
Silver-free β-Fluorination: Termolecular pathway I
Silver-mediated β-Fluorination (AgF addition)
β-Fluorination Product
β-Fluorination Transition state
'Pre-β-fluorination' intermediate
Alkynyl iodonium-(BF4)
Silver(I) coordination inside vs outside
'Pre-β-fluorination' intermediate
β-Fluorination Transition state
β-Fluorination Product (AgF addition)
'in'
'out'

HF-int

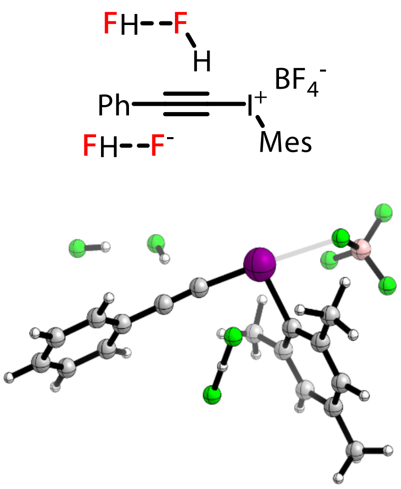

Thermal correction to the Gibbs free energy:
0.251107
Def2TZVP: **-1780.84836142**
Aug-cc-pVTZ (-pp on I):
-1778.84277018
Charge = -1 Multiplicity = 1

C	-1.31618	0.645898	-0.01079
C	-1.87521	1.752647	0.635326
C	-1.68152	2.000522	1.995566
C	-0.91522	1.103859	2.74078
C	-0.32286	-0.02569	2.165543
C	-0.54747	-0.18605	0.80102
H	-1.57005	0.408043	-1.47325
H	-2.48634	2.433575	0.048332
H	-2.2757	3.225106	2.640351
H	-0.76755	1.276474	3.803597
H	0.491542	-0.96754	3.008917
C	0.312588	-1.93035	-0.14462
C	-2.15867	1.229084	-1.8854
I	-2.12728	-0.52121	-1.62289
C	-0.61606	0.355136	-2.0054
C	0.424694	-0.68079	4.059033
C	1.548053	-0.94314	2.721398
C	0.12711	-1.99444	2.908785
C	-3.25201	3.461182	2.210678
C	-1.6247	4.090767	2.48034
H	-2.39108	3.087591	3.717479
H	2.270862	-1.47741	0.124501
C	3.416591	-1.10172	0.262715
H	4.772502	-0.66529	0.413692
H	5.570439	-0.47949	-0.72519
H	5.156814	-0.6776	-1.70831
C	6.882264	-0.04131	-0.5783
H	7.502349	0.103139	-1.45649
H	7.397183	0.214111	0.694009
H	8.421116	0.556402	0.803234
C	6.601079	0.032545	1.826309
H	7.003072	0.233608	2.813474
H	5.288161	-0.40648	1.692527
H	4.659589	-0.55058	2.565695

B	3.405944	-3.71219	1.801339
F	5.915774	-4.07023	2.488504
F	5.022697	-3.93321	2.2388
F	-3.02754	-2.54969	0.917872
F	-3.74653	-1.36844	1.092226
Ag	-2.09066	-2.71322	1.949291
B	-3.88598	-3.64741	0.858633
F	-2.30056	-2.47034	-0.31798
F	1.469755	0.800809	-1.50996
F	2.1907	1.840275	0.380215
F	1.835962	1.319998	-0.53468
F	3.200055	-2.96629	1.260093

HF-TS

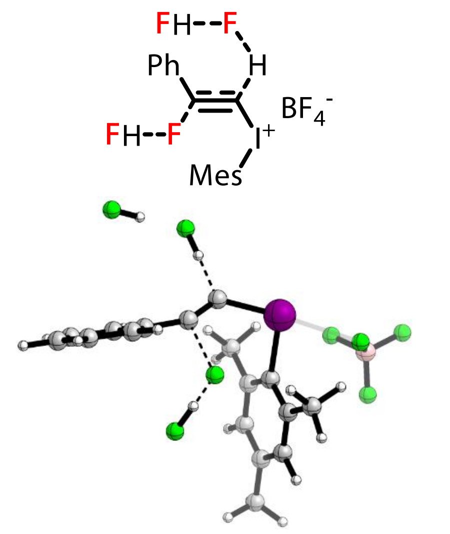

Thermal correction to the Gibbs free energy:
0.254612
Def2TZVP: **-1780.83350621**
Aug-cc-pVTZ (-pp on I):
-1778.82731921
Charge = -1 Multiplicity = 1

C	-0.9211	-0.31944	-0.016264
C	-1.60183	0.601572	0.784608
C	-1.45687	0.630967	2.173177
C	-0.60779	-0.29482	2.780563
C	0.096712	-1.25298	2.042961
C	-0.09908	-1.21616	0.663908
H	-1.0909	-0.30565	-1.509618
H	-2.25999	1.318151	0.299493
H	-2.17938	1.66616	2.994912
H	-0.48777	-0.28537	3.860834
H	0.984065	-2.24832	2.740295
C	0.907822	-2.72001	-0.50466
C	-1.74581	0.515598	-1.804576
I	-1.53434	-1.24201	-1.860878
C	-0.11735	-0.16755	-1.989671
C	0.970844	-2.07295	3.816727
C	2.021661	-2.16999	2.39822
C	0.63501	-3.26814	2.550951
C	-3.16391	1.885043	2.57496
C	-1.61043	2.601586	3.01142
H	-2.30537	1.333297	4.027362
H	2.859775	-2.1158	-0.242919
C	3.378101	-0.97005	-0.277601
H	4.436966	-0.03281	-0.064637
H	4.951239	0.723871	-1.129609
H	4.512488	0.621193	-2.115832
C	6.004064	1.59833	-0.89745
H	6.407845	2.187159	-1.713899
H	6.542918	1.719784	0.387619
H	7.366607	2.404436	0.562594
C	6.029291	0.970704	1.446737
H	6.450023	1.071102	2.441283
H	4.969968	0.095885	1.227314
H	4.554714	-0.49342	2.038751
B	4.673254	-3.85228	0.48062
F	7.043303	-2.97675	0.453527
F	6.15236	-3.29817	0.46414
F	-2.42261	-3.75064	0.459411
F	-3.2596	-2.70792	0.86112
Ag	-1.43481	-3.98532	1.430129
B	-3.1644	-4.91467	0.236088
F	-1.76731	-3.38326	-0.754857
F	1.973413	0.363363	-0.966099
F	1.854947	2.031186	0.621204
F	1.892224	1.282756	-0.089574
F	3.972527	-3.19464	0.19164

HF-P

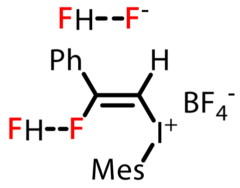

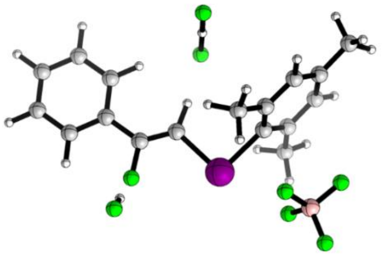

Thermal correction to the Gibbs free energy: **0.258944**
Def2TZVP: **-1780.89882196**
Aug-cc-pVTZ (-pp on **I** and Ag): **-1778.89202282**
Charge = -1 Multiplicity = 1

C	0.360989	0.606346	0.06063
C	0.018338	1.750988	0.794234
C	0.094245	1.789589	2.190341
C	0.536851	0.652618	2.869654
C	0.910335	-0.51806	2.200577
C	0.807942	-0.48351	0.808495
H	0.235181	0.606584	-1.438813
H	-0.32739	2.627049	0.251954
H	-0.32063	3.023572	2.946089
H	0.604672	0.670676	3.954052
H	1.391828	-1.71286	2.977077
C	1.47271	-2.22211	-0.265913
C	-0.13215	1.57418	-1.78246
I	-0.46787	-0.16721	-1.759805
C	1.199269	0.416037	-1.919176
C	1.329572	-1.50959	4.047076
C	2.432527	-1.96657	2.743647
C	0.767401	-2.58305	2.753223
C	-0.16361	3.923796	2.348176
C	0.23682	3.119433	3.880163
H	-1.38522	2.969525	3.195735
H	3.504641	-1.92066	0.174738
C	4.180357	-0.94288	-0.430144
H	5.574467	-0.55493	-0.173693
H	6.195567	0.383602	-1.005533
H	5.646836	0.825528	-1.829637
C	7.520417	0.746419	-0.770556
H	7.999759	1.473928	-1.417208
H	8.225161	0.178314	0.289904
H	9.256521	0.463591	0.471024
C	7.6015	-0.75537	1.121642
H	8.139635	-1.19853	1.95306
H	6.280217	-1.12264	0.896563
H	5.805708	-1.84118	1.55815
B	4.37946	-3.35805	2.607612
F	6.469437	-3.18865	3.487156
F	5.467663	-3.28243	3.080322
F	-2.0898	-2.20079	0.498586
F	-2.49759	-0.91634	0.868052
Ag	-1.26836	-2.75456	1.496413
B	-3.20168	-3.02107	0.28183
F	-1.32845	-2.12342	-0.702338
F	3.573048	-0.20673	-1.377176
F	3.288146	1.60099	0.980636
F	2.362669	1.71384	0.959272
F	3.893871	-2.55751	0.967687

B-Ag-in

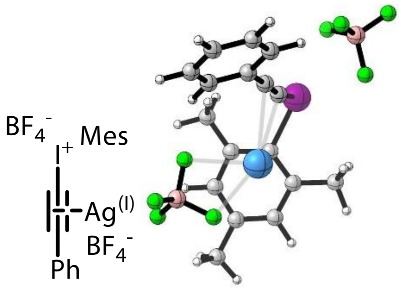

Thermal correction to the Gibbs free energy: **0.236405**
Def2TZVP: **-1950.92436129**
Aug-cc-pVTZ (-pp on I and Ag): **-1948.85018099**
Charge = 0 Multiplicity = 1

C	-3.4049	4.27663	-0.35002
C	-3.10902	2.91722	-0.33788
C	-1.88473	2.49079	0.19833
C	-0.96436	3.41541	0.71887
C	-1.27367	4.7702	0.69373
C	-2.49156	5.19999	0.16178
H	-4.35021	4.61633	-0.75939
H	-3.80785	2.18126	-0.72035
H	-0.01936	3.06521	1.12454
H	-0.56624	5.49066	1.08983
H	-2.72908	6.25874	0.14661
C	-1.57007	1.09538	0.23599
C	-1.25325	-0.08151	0.25375
C	1.1053	-1.95735	0.38404
C	1.57791	-2.50823	-0.81297
C	1.90152	-1.3402	1.35927
C	2.9561	-2.39515	-1.03079
C	3.2641	-1.26472	1.07086
C	3.80492	-1.77731	-0.11132
H	3.36553	-2.79867	-1.9526
H	3.91552	-0.76608	1.78237
I	-1.01758	-2.03099	0.74154
C	0.7012	-3.17386	-1.84253
H	0.2165	-4.06746	-1.43889
H	-0.08078	-2.49682	-2.20114
H	1.30222	-3.47632	-2.70025
C	1.36101	-0.74011	2.62892
H	0.66725	0.08009	2.4133
H	0.83691	-1.48488	3.23465
H	2.17962	-0.32966	3.2201
C	5.27596	-1.63173	-0.3847
H	5.8648	-2.06637	0.428
H	5.55827	-2.11941	-1.31926
H	5.5327	-0.56994	-0.44655
F	-3.77248	-1.52797	0.92487
F	-4.68317	0.11106	-0.40699
F	-3.4346	-1.57939	-1.33776
F	-5.522	-2.02445	-0.48264
B	-4.36931	-1.25108	-0.3398
Ag	0.88068	0.64571	-1.01887
B	3.36989	2.04941	0.0907
F	4.52677	1.5381	0.66585
F	2.23951	1.74287	0.89008
F	3.14623	1.42564	-1.1762
F	3.46337	3.42675	-0.08636

Ag-in-int

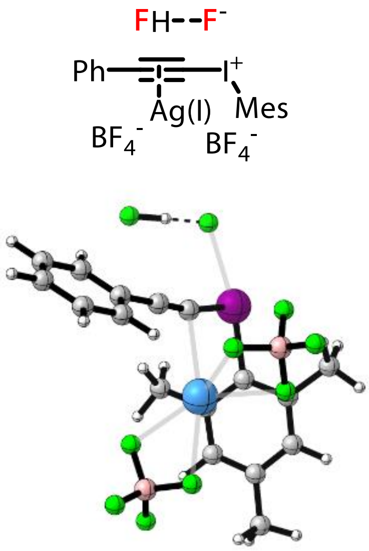

Thermal correction to the Gibbs free energy:
0.245605
Def2TZVP: **-2151.43468163**
Aug-cc-pVTZ (-pp on I and Ag):
-2149.36309363
Charge = -1 Multiplicity = 1

F	0.993802	0.868156	2.657599
F	-1.05576	-0.11337	2.791864
F	0.310987	-0.07283	4.648206
B	0.280077	-0.19433	3.267006
Ag	-0.44537	0.765993	0.511821
B	-2.10552	2.841809	-1.024272
F	-3.242	2.6509	-1.805254
F	-1.02574	2.072461	-1.539087
F	-2.33993	2.375332	0.297073
F	-1.74661	4.18728	-0.990209
F	2.961931	-3.46567	-0.297141
H	3.6014	-2.821	-1.15374
F	4.114177	-2.2734	-1.883002

C	3.984539	3.107106	0.337891
C	3.080676	2.062634	0.504265
C	3.134458	0.962811	-0.365157
C	4.0985	0.902067	-1.384353
C	4.999105	1.952103	-1.532793
C	4.940451	3.05447	-0.678145
H	3.944749	3.96183	1.00473
H	2.34405	2.083349	1.300555
H	4.137502	0.023685	-2.021078
H	5.748373	1.910009	-2.316297
H	5.643387	3.872097	-0.801141
C	2.215342	-0.12977	-0.236772
C	1.429588	-1.05541	-0.1789
C	-1.44762	-1.91589	-0.261791
C	-2.28592	-2.11991	0.838699
C	-1.78946	-1.13244	-1.378986
C	-3.50895	-1.43869	0.824303
C	-3.01489	-0.46723	-1.31811
C	-3.87683	-0.59749	-0.224426
H	-4.17775	-1.56233	1.671818
H	-3.29703	0.179413	-2.144043
I	0.493365	-2.84343	-0.243723
C	-1.93695	-3.01795	1.995887
H	-1.84426	-4.06059	1.674493
H	-0.9959	-2.71061	2.459398
H	-2.72069	-2.97024	2.753068
C	-0.92415	-0.99616	-2.6065
H	0.04012	-0.52702	-2.386641
H	-0.72637	-1.97479	-3.05498
H	-1.43066	-0.37531	-3.345811
C	-5.16569	0.177165	-0.193022
H	-5.76635	-0.0839	0.680205
H	-4.94798	1.249181	-0.163818
H	-5.75626	-0.01355	-1.093623
F	0.830624	-1.41381	2.851954

Ag-in-TS

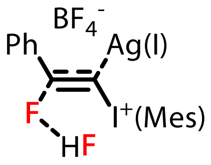

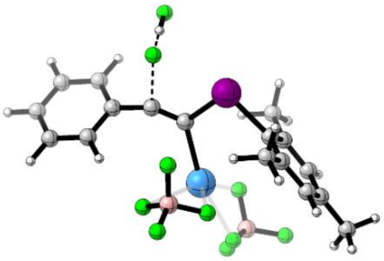

C	-0.05911	4.929606	-2.282205
C	-0.36305	4.480091	-0.99682
C	-0.25222	3.127726	-0.686908
C	0.157731	2.225738	-1.681981
C	0.46504	2.676649	-2.974988
C	0.354971	4.029296	-3.269081
H	-0.14326	5.985665	-2.518137
H	-0.68377	5.181815	-0.234189
H	-0.49278	2.764923	0.309061
H	0.798537	1.964093	-3.72083
H	0.592064	4.386253	-4.265554
C	0.237367	0.833215	-1.350195
C	-0.21742	-0.24193	-0.870199
I	0.830668	-1.95368	-0.397538
C	2.47954	-1.08206	0.661874
C	3.758738	-1.23828	0.122783
C	2.192768	-0.42653	1.861945
C	4.810415	-0.7126	0.877091
C	3.291532	0.0903	2.556699
C	4.597823	-0.04602	2.085732

Thermal correction to the Gibbs free energy:
0.243505
Def2TZVP: **-2151.41614881**
Aug-cc-pVTZ (-pp on I and Ag):
-2149.34481751
Charge = -1 Multiplicity = 1

H	5.823103	-0.82038	0.49674
H	3.111565	0.608512	3.495041
C	0.801619	-0.24357	2.409587
H	0.199902	0.409913	1.768302
H	0.271715	-1.19722	2.500329
H	0.843469	0.215689	3.398065
C	4.032028	-1.91348	-1.194167
H	3.765841	-2.97491	-1.171069
H	3.457447	-1.42166	-1.985089
H	5.093275	-1.84297	-1.43701
C	5.755856	0.541491	2.84861
H	5.986219	1.544021	2.473225
H	5.52495	0.628366	3.912449
H	6.653821	-0.06981	2.733281
B	-2.73571	-2.88205	-0.96671
F	-1.54237	-3.00101	-1.709997
F	-2.39911	-2.58806	0.374422
F	-3.48311	-1.79528	-1.474306
F	-3.47323	-4.05575	-1.040839
Ag	-2.37316	0.041302	-0.073186
B	-2.65714	1.893251	2.31644
F	-1.41922	2.536822	2.400957
F	-3.53591	2.373982	3.280685
F	-3.20102	2.101603	1.017852
F	-2.47592	0.498134	2.46668
F	3.461593	2.153106	-0.958888
H	2.890184	1.419257	-1.390653
F	2.155462	0.51988	-1.94586

Ag-in-P

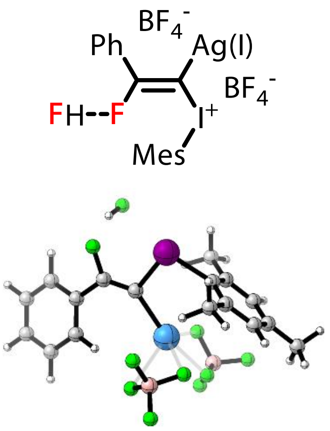

Thermal correction to the Gibbs free energy:
0.243264
Def2TZVP: **-2151.4500722**
Aug-cc-pVTZ (-pp on I and Ag):
-2149.37751519
Charge = -1 Multiplicity = 1

C	4.046287	2.626529	0.671515
C	3.232591	1.501131	0.574343
C	3.548772	0.485435	-0.339031
C	4.702036	0.597585	-1.126679
C	5.515275	1.723514	-1.017219
C	5.187234	2.743341	-0.124041
H	3.795462	3.40719	1.382551
H	2.366984	1.398197	1.222453
H	4.964333	-0.19023	-1.824896
H	6.405465	1.802996	-1.632766
H	5.822449	3.619279	-0.040588
C	2.673429	-0.6926	-0.475275
C	1.359066	-0.72184	-0.252108
C	-1.44772	-1.97808	-0.391425
C	-2.11844	-2.09576	0.82709
C	-1.93891	-1.29123	-1.510721
C	-3.374	-1.47987	0.898948
C	-3.1904	-0.69217	-1.36359
C	-3.9184	-0.77561	-0.173313
H	-3.9224	-1.53922	1.835368
H	-3.59452	-0.1234	-2.19647
I	0.542263	-2.75315	-0.520682
C	-1.54838	-2.79057	2.034538
H	-1.27968	-3.82815	1.816716
H	-0.65541	-2.26548	2.390058
H	-2.28054	-2.78895	2.843399
C	-1.1792	-1.14468	-2.802669
H	-0.25077	-0.5831	-2.648701
H	-0.91832	-2.11828	-3.228173
H	-1.78359	-0.5988	-3.527886
C	-5.25765	-0.09759	-0.064389
H	-5.65973	-0.17335	0.947865
H	-5.16139	0.958514	-0.331383
H	-5.97684	-0.55021	-0.754211
F	0.778841	-0.5248	3.468416
F	0.612233	1.62962	2.683734
F	-1.22227	0.265463	2.667531
F	-0.39488	1.044985	4.668375
B	-0.05454	0.598701	3.389863
Ag	-0.23427	0.753812	0.288359
B	-2.28509	2.686297	-1.031832
F	-3.5873	2.461772	-1.475914
F	-1.36033	1.986742	-1.832863

F	-2.14402	2.192321	0.300798
F	-1.9917	4.05142	-1.043358
F	3.369487	-1.81327	-0.917291
H	3.099582	-2.04236	-2.694966
F	2.700847	-2.01976	-3.537259

Ag-out

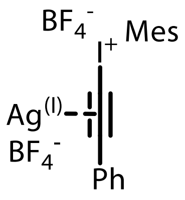

'out'

Thermal correction to the Gibbs free energy: **0.235912**
Def2TZVP: **-1950.93154395**
Aug-cc-pVTZ (-pp on I and Ag): **-1948.85674705**
Charge = 0 Multiplicity = 1

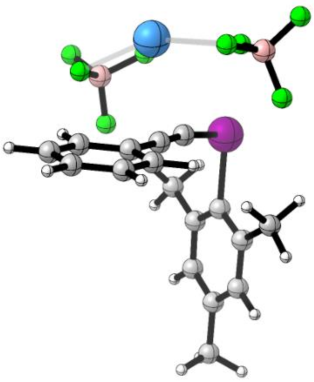

C	-0.43041	0.606025	0.016681
C	-0.14236	-0.20394	1.116969
C	0.589832	-1.37473	0.94957
C	1.03337	-1.73095	-0.333649
C	0.741726	-0.92059	-1.441606
C	0.010177	0.248052	-1.259136
H	-1.00164	1.518534	0.153267
H	-0.48926	0.076938	2.105627
H	0.814952	-2.02398	1.789617
H	1.088719	-1.21375	-2.427304
H	-0.21743	0.879333	-2.111338
C	1.792661	-2.93253	-0.515505
C	2.409843	-3.9648	-0.67198
I	3.677818	-5.4922	-1.008134
C	5.484716	-4.35347	-0.746233
C	5.781314	-3.39924	-1.72504
C	6.269375	-4.63752	0.372415
C	6.959455	-2.67617	-1.527626
C	7.438395	-3.87928	0.498885
C	7.793397	-2.89925	-0.428666
H	7.230398	-1.9209	-2.260714
H	8.084034	-4.06813	1.352224
C	5.933432	-5.69041	1.395514
H	4.941171	-5.52376	1.823758
H	5.952216	-6.69153	0.953457
H	6.666183	-5.67005	2.202918
C	4.91587	-3.12707	-2.926945
H	4.770368	-4.03022	-3.527064
H	3.927037	-2.75883	-2.634218
H	5.383081	-2.37083	-3.558127
C	9.042694	-2.07975	-0.245169
H	9.736804	-2.56569	0.443002
H	9.548947	-1.919	-1.199958
H	8.792225	-1.09649	0.165782
B	0.577361	-5.4965	-3.062086
F	1.672792	-4.80091	-3.570931
F	1.005934	-6.28472	-1.949137
F	-0.38479	-4.58045	-2.562695
F	0.004581	-6.31439	-4.02387
Ag	-0.26718	-4.90448	-0.119984
B	1.580802	-5.43339	2.297868
F	2.840244	-4.83151	2.251395
F	1.414882	-6.15652	3.470852
F	0.572799	-4.43778	2.192354
F	1.429613	-6.28953	1.172092

Ag-out-int

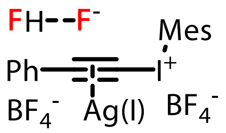

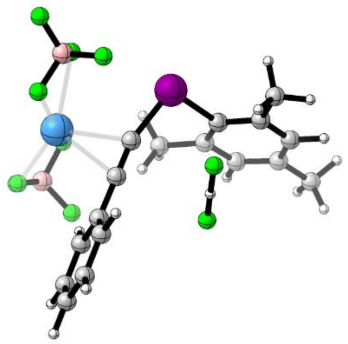

C	0.237354	0.631067	-0.001199
C	0.306526	0.512101	-1.39066
C	0.97339	1.472792	-2.143746
C	1.5744	2.560442	-1.490918
C	1.508376	2.681214	-0.094277
C	0.837094	1.713652	0.645227
H	-0.2856	-0.12191	0.579541
H	-0.16082	-0.33061	-1.88888
H	1.025856	1.389627	-3.226313
H	1.984221	3.525378	0.393567
H	0.782838	1.803058	1.724863
C	2.292865	3.551332	-2.233423
C	2.899712	4.424896	-2.825162
I	4.339287	5.596712	-3.609999
C	5.775026	4.081531	-4.135373
C	7.053184	4.227115	-3.588192
C	5.378954	3.070498	-5.008705
C	7.982862	3.254586	-3.958356
C	6.364777	2.129432	-5.33207
C	7.657747	2.200918	-4.817898
H	8.992355	3.329248	-3.56189
H	6.098709	1.319576	-6.006116
C	3.992654	2.918601	-5.57159
H	3.305009	2.525359	-4.814968
H	3.58188	3.865684	-5.935325
H	3.999092	2.215962	-6.405788
C	7.449839	5.351857	-2.6699
H	7.385587	6.323492	-3.171476
H	6.799433	5.353306	-1.790268
H	8.482919	5.220231	-2.344969
C	8.692546	1.165093	-5.170103
H	8.926703	0.548574	-4.296757
H	8.339458	0.507292	-5.966364
H	9.622336	1.638889	-5.496091
B	1.116645	7.369431	-3.374953

Thermal correction to the Gibbs free energy:
0.240282
Def2TZVP: **-2151.42429878**
Aug-cc-pVTZ (-pp on I and Ag):
-2149.35247318
Charge = -1 Multiplicity = 1

F	2.292869	7.414245	-2.602618
F	1.455817	6.866191	-4.660639
F	0.212739	6.453384	-2.794891
F	0.546878	8.626822	-3.480287
Ag	0.72538	4.457808	-4.322969
B	0.156093	2.024137	-6.023435
F	0.96299	0.997885	-5.530197
F	-0.64091	1.580423	-7.069669
F	-0.65723	2.52279	-4.965535
F	0.968809	3.10676	-6.445949
F	4.913913	1.910146	-1.731014
H	5.0531	2.948277	-1.381792
F	5.208624	4.041817	-0.993052

Ag-out-TS

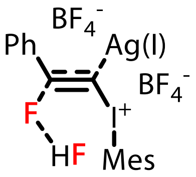

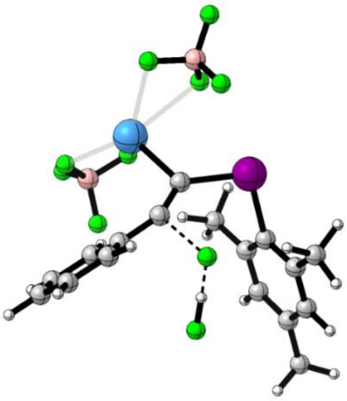

Thermal correction to the Gibbs free energy:
0.242031
Def2TZVP: **-2151.41157883**
Aug-cc-pVTZ (-pp on I and Ag):
-2149.33950959
Charge = -1 Multiplicity = 1

F	-1.54237	-3.00101	-1.709997
F	-2.39911	-2.58806	0.374422
F	-3.48311	-1.79528	-1.474306
F	-3.47323	-4.05575	-1.040839
Ag	-2.37316	0.041302	-0.073186
B	-2.65714	1.893251	2.31644
F	-1.41922	2.536822	2.400957
F	-3.53591	2.373982	3.280685
F	-3.20102	2.101603	1.017852
F	-2.47592	0.498134	2.46668
F	3.461593	2.153106	-0.958888
H	2.890184	1.419257	-1.390653
F	2.155462	0.51988	-1.94586

C	-0.05911	4.929606	-2.282205
C	-0.36305	4.480091	-0.99682
C	-0.25222	3.127726	-0.686908
C	0.157731	2.225738	-1.681981
C	0.46504	2.676649	-2.974988
C	0.354971	4.029296	-3.269081
H	-0.14326	5.985665	-2.518137
H	-0.68377	5.181815	-0.234189
H	-0.49278	2.764923	0.309061
H	0.798537	1.964093	-3.72083
H	0.592064	4.386253	-4.265554
C	0.237367	0.833215	-1.350195
C	-0.21742	-0.24193	-0.870199
I	0.830668	-1.95368	-0.397538
C	2.47954	-1.08206	0.661874
C	3.758738	-1.23828	0.122783
C	2.192768	-0.42653	1.861945
C	4.810415	-0.7126	0.877091
C	3.291532	0.0903	2.556699
C	4.597823	-0.04602	2.085732
H	5.823103	-0.82038	0.49674
H	3.111565	0.608512	3.495041
C	0.801619	-0.24357	2.409587
H	0.199902	0.409913	1.768302
H	0.271715	-1.19722	2.500329
H	0.843469	0.215689	3.398065
C	4.032028	-1.91348	-1.194167
H	3.765841	-2.97491	-1.171069
H	3.457447	-1.42166	-1.985089
H	5.093275	-1.84297	-1.43701
C	5.755856	0.541491	2.84861
H	5.986219	1.544021	2.473225
H	5.52495	0.628366	3.912449
H	6.653821	-0.06981	2.733281
B	-2.73571	-2.88205	-0.96671

Ag-out-P

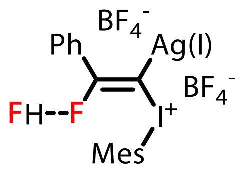

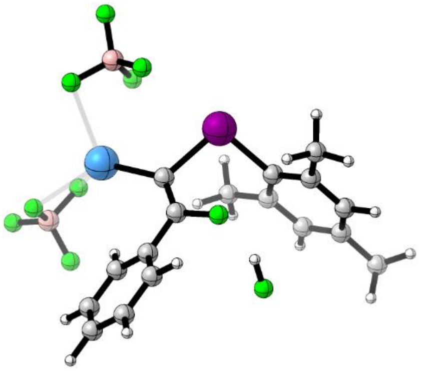

Thermal correction to the Gibbs free energy:
0.241302
Def2TZVP: **-2151.44696985**
Aug-cc-pVTZ (-pp on I and Ag):
-2149.37435763
Charge = -1 Multiplicity = 1

Ag	-2.2305	0.106674	0.155793
B	-3.56997	2.227037	1.833835
F	-2.56914	3.194425	1.949531
F	-4.72929	2.623772	2.490692
F	-3.84094	1.999284	0.457914
F	-3.10133	0.998273	2.365264
F	3.609101	2.181318	-0.453112
H	3.152779	1.459676	-0.829363
F	1.875067	0.540514	-1.748217
C	-0.51548	4.824215	-2.390061
C	-1.01528	4.260738	-1.215396
C	-0.6431	2.972394	-0.841044
C	0.222506	2.227626	-1.653085
C	0.736468	2.802051	-2.823706
C	0.365967	4.09417	-3.188361
H	-0.8031	5.830646	-2.676664
H	-1.68642	4.826964	-0.577505
H	-1.00927	2.559624	0.09414
H	1.417921	2.234817	-3.449133
H	0.764855	4.529636	-4.098801
C	0.600278	0.84596	-1.294933
C	-0.17264	-0.02854	-0.64694
I	0.889784	-1.90221	-0.241729
C	2.705887	-1.10671	0.578265
C	3.901615	-1.31454	-0.116784
C	2.615663	-0.41576	1.791353
C	5.061682	-0.79351	0.464178
C	3.812207	0.081463	2.317648
C	5.036226	-0.09389	1.671733
H	6.008538	-0.93794	-0.050187
H	3.777598	0.623504	3.259355
C	1.319862	-0.19124	2.528234
H	0.609271	0.378531	1.919049
H	0.842694	-1.14046	2.7919
H	1.502531	0.365739	3.44806
C	3.98224	-2.05157	-1.428117
H	3.65451	-3.09038	-1.325143
H	3.35062	-1.57674	-2.185304
H	5.009939	-2.05686	-1.793722
C	6.299371	0.49134	2.245961
H	6.466985	1.495781	1.842904
H	6.237184	0.5754	3.333167
H	7.169039	-0.11823	1.990419
B	-2.75581	-2.88806	-0.372644
F	-1.67915	-2.89533	-1.285505
F	-2.25763	-2.57879	0.908781
F	-3.6676	-1.87662	-0.749835
F	-3.38877	-4.12799	-0.365907

Fluoride Addition to the Alkynyl-iodonium - Iodonium Ylide Formation

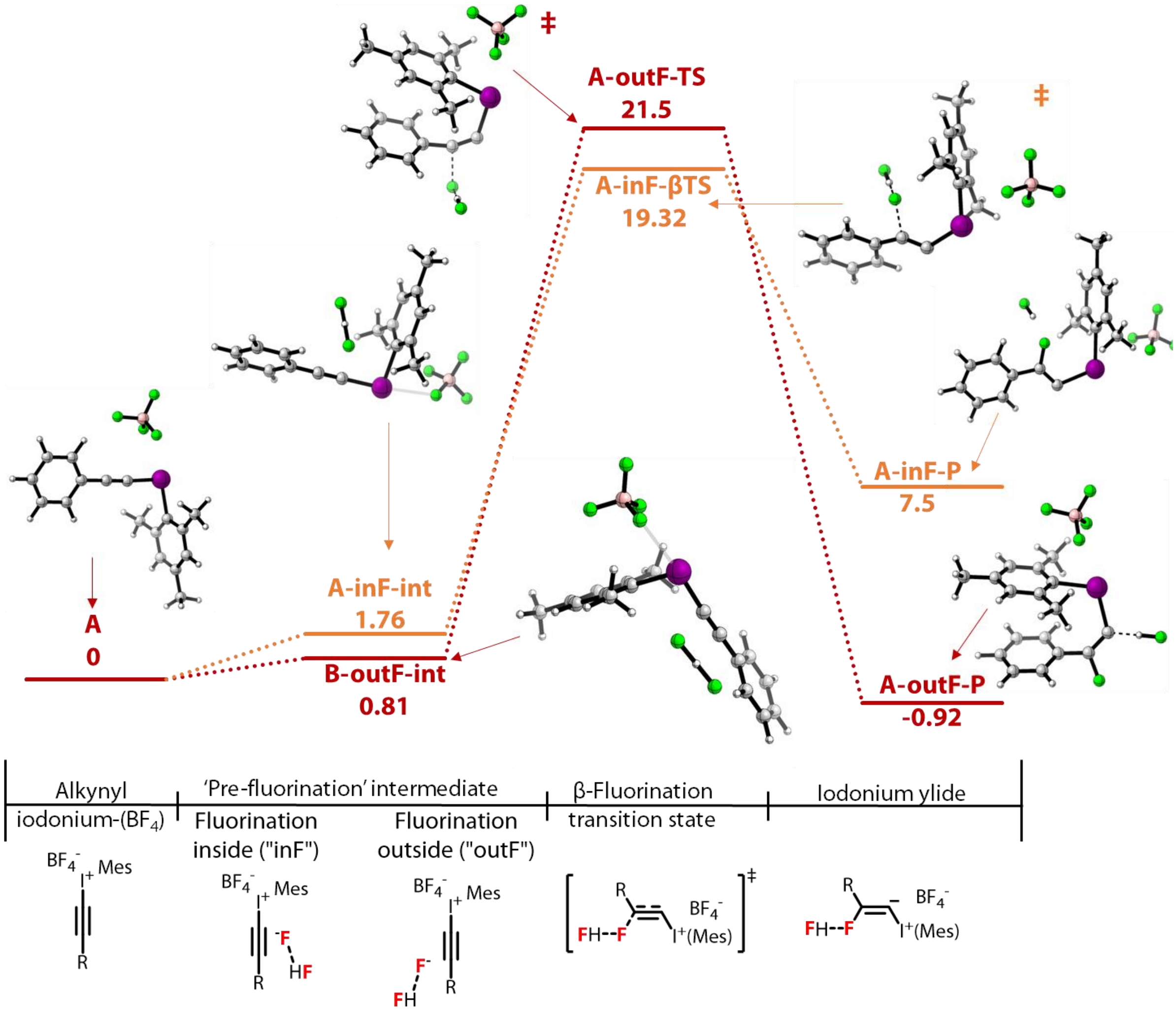

A-inF-int

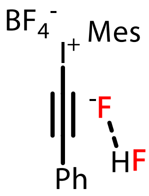

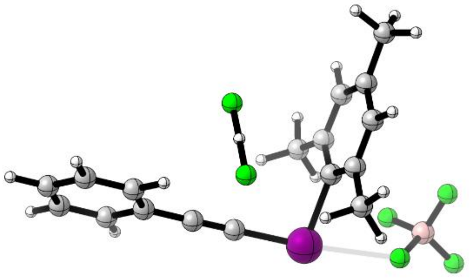

Thermal correction to the Gibbs free energy:
0.238021
Def2TZVP: **-1579.92014304**
Aug-cc-pVTZ (-pp on I): **-1577.91230286**
Charge = -1 Multiplicity = 1

C	-1.41324	1.346334	-1.0301
C	-1.80105	2.654384	-0.72071
C	-1.85551	3.123738	0.592289
C	-1.51827	2.256672	1.633769
C	-1.12725	0.934496	1.403302
C	-1.09956	0.544987	0.065099
C	-1.33377	0.893511	-2.46184
H	-2.06025	3.320332	-1.53955
C	-2.23844	4.551329	0.880402
H	-1.5575	2.610191	2.660803
C	-0.76414	0.033005	2.551385
I	-0.52151	-1.49404	-0.35318
H	-1.66551	1.695537	-3.12268
H	-1.96618	0.019354	-2.63963
H	-0.29602	0.651867	-2.71455
H	-0.88335	0.564706	3.495994
H	0.277671	-0.29755	2.480502
H	-1.40907	-0.85026	2.565997
H	-2.88387	4.952277	0.096078
H	-1.34257	5.178997	0.929953
H	-2.75631	4.635116	1.838447
C	1.449522	-1.23663	0.013359
C	2.63623	-1.03793	0.142363
C	4.036745	-0.75818	0.252909
C	4.580075	0.26839	-0.53603
H	3.920799	0.827716	-1.19532
C	5.939907	0.550388	-0.44548
H	6.365618	1.344177	-1.05053
C	6.754411	-0.1832	0.419339
H	7.814374	0.040776	0.484605
C	6.210589	-1.20347	1.202169
H	6.844905	-1.7718	1.874258
C	4.852062	-1.49438	1.124476

H	4.419041	-2.28413	1.729527
B	-4.03651	-1.25738	0.063966
F	-4.53039	0.04239	-0.02401
F	-3.31406	-1.42807	1.256933
F	-5.07159	-2.1884	-0.01574
F	-3.1262	-1.48614	-1.01979
F	1.900212	1.571193	-2.05044
F	2.052463	2.766045	-0.12476
H	1.966142	2.165849	-1.07856

A-inF-TS

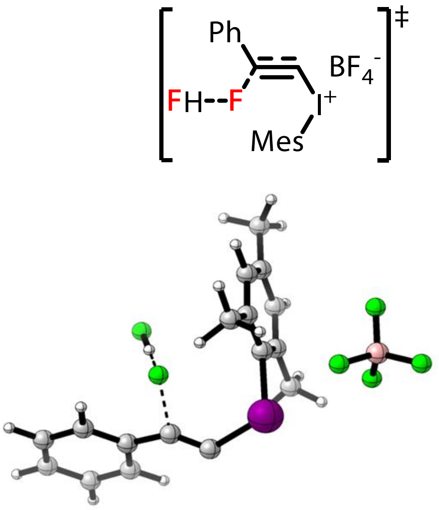

Thermal correction to the Gibbs free energy:
0.239316
Def2TZVP: **-1579.89259492**
Aug-cc-pVTZ (-pp on I): **-1577.88379077**
Charge = -1 Multiplicity = 1

C	-0.62309	-0.14311	-0.04688
C	-1.10843	1.110989	0.331334
C	-1.12186	1.531427	1.664127
C	-0.63163	0.669459	2.644566
C	-0.13161	-0.60123	2.333908
C	-0.1618	-0.95674	0.987483
C	-0.60526	-0.54951	-1.49435
H	-1.48396	1.775851	-0.44284
C	-1.62672	2.904294	2.024737
H	-0.63196	0.98461	3.68493
C	0.395337	-1.49602	3.423814
I	0.53209	-2.93353	0.484332
H	-0.98429	0.261349	-2.11828
H	-1.22847	-1.4331	-1.6602
H	0.418208	-0.77943	-1.8064
H	0.334576	-0.98985	4.38825
H	1.443089	-1.76267	3.246709
H	-0.18977	-2.41901	3.477837
H	-2.50905	3.165574	1.435235
H	-0.85873	3.657912	1.821867
H	-1.88445	2.964761	3.084176
C	2.567274	-2.80238	0.73471
C	3.327719	-1.81976	0.49382
C	4.679082	-1.30531	0.613819
C	5.271904	-0.53334	-0.3951
H	4.688988	-0.2708	-1.26977
C	6.587746	-0.10391	-0.25578
H	7.042847	0.493962	-1.03877
C	7.321238	-0.43799	0.884874
H	8.346888	-0.09883	0.989525
C	6.732984	-1.2033	1.891979
H	7.29699	-1.45974	2.782801
C	5.414809	-1.63332	1.762309

H	4.944947	-2.2233	2.542505
B	-3.07278	-2.86227	1.164268
F	-3.67831	-1.60289	1.219501
F	-2.23246	-3.04411	2.276771
F	-4.04892	-3.8664	1.134323
F	-2.27962	-2.95223	-0.01179
F	2.491131	-0.35498	-0.33199
F	2.739413	1.613551	0.890955
H	2.623987	0.760517	0.363974

A-inF-P

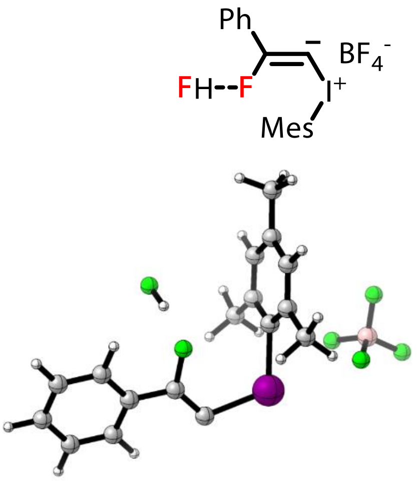

Thermal correction to the Gibbs free energy:
0.240144
Def2TZVP: **-1579.91314254**
Aug-cc-pVTZ (-pp on I): **-1577.90377799**
Charge = -1 Multiplicity = 1

C	-1.42005	1.156896	-1.3642
C	-1.80325	2.503065	-1.30858
C	-1.74075	3.242514	-0.12803
C	-1.28016	2.613226	1.031204
C	-0.8836	1.273605	1.039776
C	-0.97664	0.585375	-0.17249
C	-1.5043	0.403162	-2.66499
H	-2.15996	2.978363	-2.21912
C	-2.13728	4.69624	-0.09863
H	-1.2195	3.17856	1.958479
C	-0.39488	0.636429	2.313418
I	-0.38713	-1.48427	-0.18783
H	-1.86937	1.05775	-3.4581
H	-2.18665	-0.44704	-2.57453
H	-0.52368	0.018825	-2.96308
H	-0.36501	1.373346	3.117537
H	0.61373	0.222789	2.198835
H	-1.0611	-0.18082	2.60625
H	-2.64943	4.984897	-1.01884
H	-1.25444	5.333857	0.012382
H	-2.79966	4.903876	0.746141
C	1.75857	-1.74724	0.06244
C	2.47001	-0.62113	-0.0456
C	3.938295	-0.53496	0.085049
C	4.627981	0.606418	-0.34384
H	4.082667	1.439374	-0.77384
C	6.016768	0.675615	-0.22781
H	6.536569	1.567449	-0.56353
C	6.732877	-0.3908	0.31095
H	7.812927	-0.33497	0.400986
C	6.048806	-1.53164	0.7401
H	6.596689	-2.3653	1.168153
C	4.664628	-1.60331	0.634085
H	4.131285	-2.48237	0.980273
B	-4.0764	-0.90446	0.569512
F	-4.59188	0.392889	0.431854
F	-3.13647	-0.93544	1.615302
F	-5.12221	-1.79994	0.852296
F	-3.44383	-1.29404	-0.63156
F	1.923689	0.638339	-0.3362
F	2.178123	2.543185	1.509184
H	1.998406	1.90367	0.850062

A-outF-Int

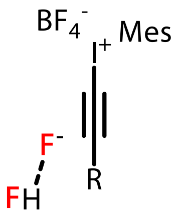

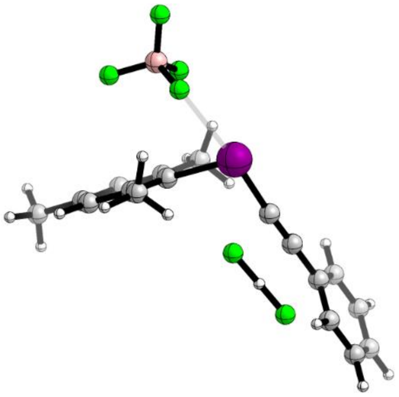

Thermal correction to the Gibbs free energy:
0.237993
Def2TZVP: **-1579.92161915**
Aug-cc-pVTZ (-pp on I): **-1577.91443262**
Charge = -1 Multiplicity = 1

C	-0.0363	0.358912	0.158197
C	-0.65658	1.452341	0.766399
C	-1.20724	1.376752	2.048974
C	-1.14128	0.167406	2.738575
C	-0.53505	-0.97262	2.194391
C	0.003923	-0.81065	0.920653
C	0.527729	0.480157	-1.23127
H	-0.71148	2.386908	0.213727
C	-1.84822	2.589803	2.670469
H	-1.57715	0.091059	3.731177
C	-0.51618	-2.26505	2.963913
I	0.95323	-2.53278	0.03106
H	0.319892	1.472676	-1.63267
H	0.074836	-0.26348	-1.89351
H	1.612781	0.333067	-1.23736
H	-0.92362	-2.10641	3.963547
H	0.503479	-2.65425	3.053299
H	-1.13283	-3.01413	2.45707
H	-2.52822	3.075494	1.965736
H	-1.08541	3.323188	2.950216
H	-2.40908	2.324356	3.568587
C	2.825022	-1.77313	0.028823
C	3.958555	-1.35311	-0.04019
C	5.298073	-0.84722	-0.11023
C	6.21742	-1.17504	0.899023
H	5.895454	-1.81109	1.721167
C	7.51625	-0.67872	0.822832
H	8.232207	-0.92788	1.599369
C	7.899902	0.135416	-0.24441
H	8.914122	0.518562	-0.29586
C	6.983101	0.459083	-1.24665
H	7.282103	1.091689	-2.07584
C	5.682375	-0.03039	-1.18491
H	4.961142	0.213753	-1.95827
B	-2.56604	-2.51352	-0.58578
F	-3.26301	-1.72113	0.325646
F	-1.61547	-3.31435	0.128276
F	-3.43768	-3.35981	-1.27199
F	-1.84993	-1.71123	-1.4879
F	2.555581	-3.10477	2.791693
F	4.808525	-3.04522	3.054315
H	3.685674	-3.07192	2.917213

A-outF-TS

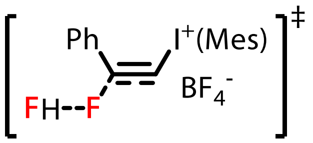

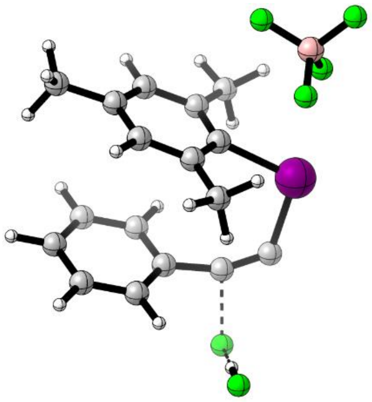

Thermal correction to the Gibbs free energy:
0.240879
Def2TZVP: **-1579.89165603**
Aug-cc-pVTZ (-pp on I): **-1577.88402998**
Charge = -1 Multiplicity = 1

C	0.658452	-0.17868	0.036638
C	0.911069	1.186944	-0.11349
C	0.688935	2.100649	0.919061
C	0.177005	1.635011	2.129849
C	-0.09016	0.279217	2.350388
C	0.165796	-0.57824	1.281596
C	0.908958	-1.12568	-1.10663
H	1.304082	1.539279	-1.06427
C	1.036021	3.553937	0.735785
H	-0.00935	2.337111	2.938676
C	-0.61121	-0.18026	3.686371
I	-0.04489	-2.69316	1.617741
H	1.257865	-0.57282	-1.9802
H	-0.01142	-1.65478	-1.37037
H	1.673934	-1.86889	-0.85547
H	-0.76427	0.676631	4.343891
H	0.09586	-0.8591	4.175169
H	-1.56316	-0.70642	3.572916
H	0.809912	3.889625	-0.27917
H	2.108673	3.705302	0.902778
H	0.493829	4.184806	1.443282
C	1.930535	-3.23642	1.715798
C	2.897404	-2.44754	1.551157
C	3.548757	-1.17705	1.35358
C	4.264098	-0.9009	0.177419
H	4.435305	-1.69996	-0.53782
C	4.737002	0.38504	-0.05476
H	5.285254	0.601534	-0.96592
C	4.502713	1.400004	0.878586
H	4.868309	2.404444	0.688355
C	3.802446	1.125305	2.05216
H	3.616604	1.912948	2.775748
C	3.327784	-0.16162	2.294531
H	2.771534	-0.38312	3.200605
B	-3.11776	-1.11748	0.471763
F	-3.06448	0.273932	0.588976
F	-2.68861	-1.70913	1.693799
F	-4.426	-1.53251	0.200347
F	-2.25256	-1.55215	-0.54769
F	4.523697	-3.52626	1.545915
F	4.649203	-4.1583	-0.67084
H	4.598439	-3.89679	0.32161

A-outF-P

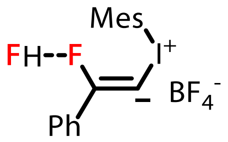

Thermal correction to the Gibbs free energy:
0.24174
Def2TZVP: **-1579.92808952**
Aug-cc-pVTZ (-pp on I): **-1577.91965811**
Charge = -1 Multiplicity = 1

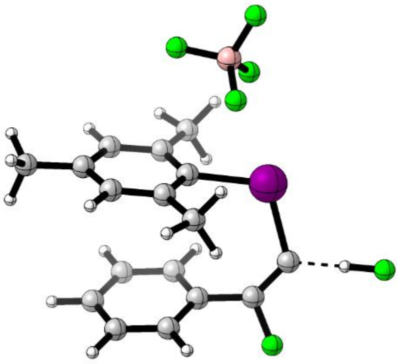

C	0.119661	0.993738	-1.18561
C	0.157308	2.381889	-1.34054
C	-0.20495	3.252666	-0.31105
C	-0.64028	2.714079	0.899541
C	-0.69009	1.334424	1.123907
C	-0.29552	0.52178	0.061219
C	0.49085	0.091947	-2.33126
H	0.486291	2.787896	-2.29428
C	-0.08943	4.742905	-0.49448
H	-0.93818	3.380364	1.705611
C	-1.13631	0.806129	2.461089
I	-0.28732	-1.60424	0.375079
H	0.789091	0.685852	-3.19696
H	-0.36448	-0.53152	-2.61015
H	1.324843	-0.56928	-2.07371
H	-1.40254	1.632937	3.121324
H	-0.34215	0.228024	2.94598
H	-2.00743	0.154379	2.350303
H	-0.3128	5.033933	-1.52368
H	0.932441	5.070464	-0.27213
H	-0.76497	5.279118	0.175641
C	1.708586	-2.39777	0.240245
C	2.763789	-1.59125	0.150699
C	2.998874	-0.13319	0.191943
C	3.705098	0.481929	-0.85071
H	4.068749	-0.11904	-1.67927
C	3.923861	1.856236	-0.83138
H	4.459914	2.328774	-1.64829
C	3.452361	2.624902	0.235308
H	3.620753	3.697356	0.248209
C	2.76739	2.01422	1.284437
H	2.402289	2.606486	2.117669
C	2.542817	0.638802	1.263405
H	2.015431	0.158774	2.084225

B	-3.60682	-0.22808	-0.77931
F	-3.71791	1.166504	-0.69932
F	-3.17063	-0.73947	0.466435
F	-4.85609	-0.78689	-1.0934
F	-2.67062	-0.57875	-1.768
F	3.956596	-2.26416	-0.04055
F	1.580425	-4.94603	0.152045
H	1.629487	-3.90048	0.189082

Regio- and Stereoselectivity Study with Alternative H-Bonding environments – (HF).(HF)'

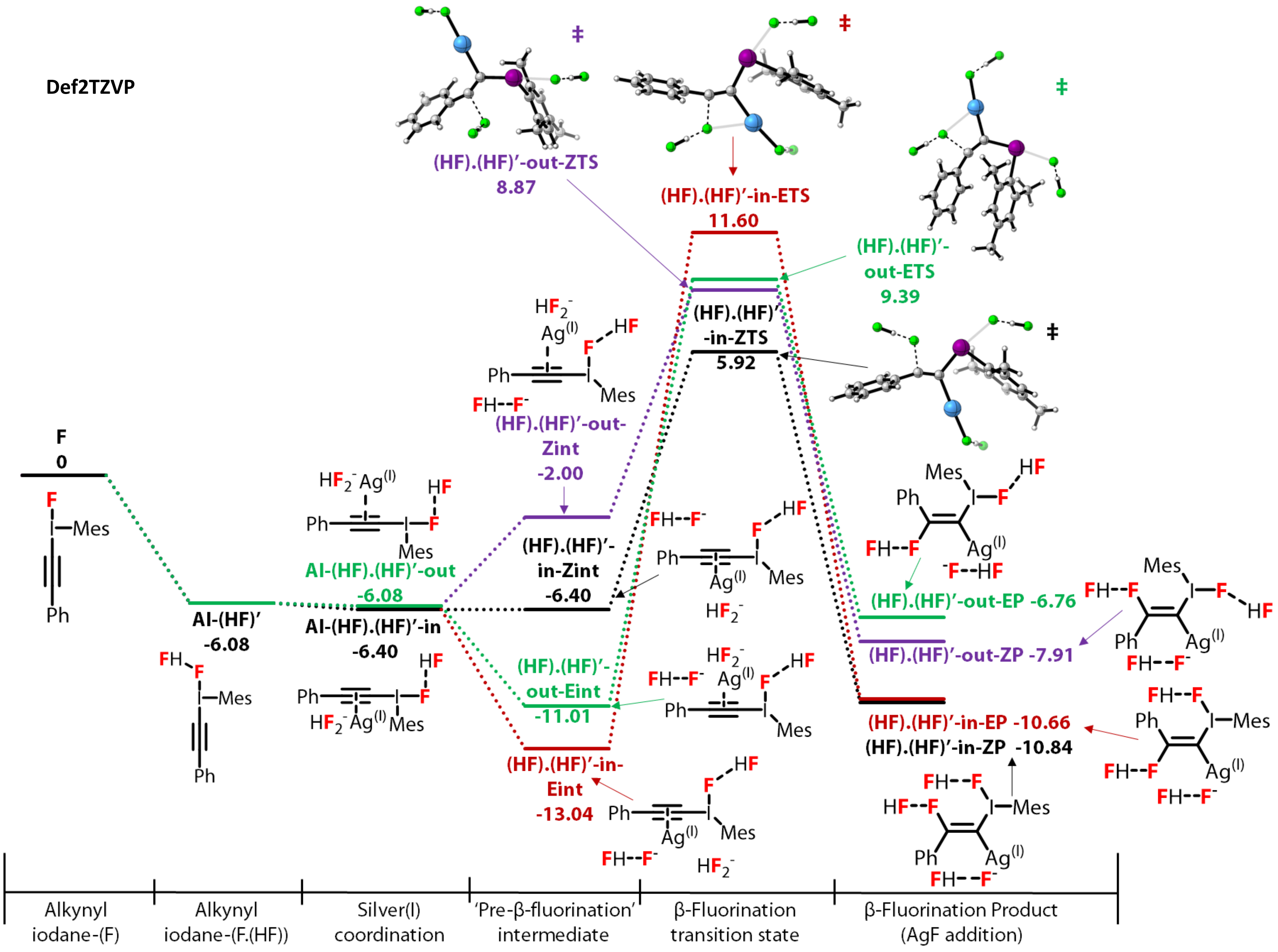

AI-(HF)’

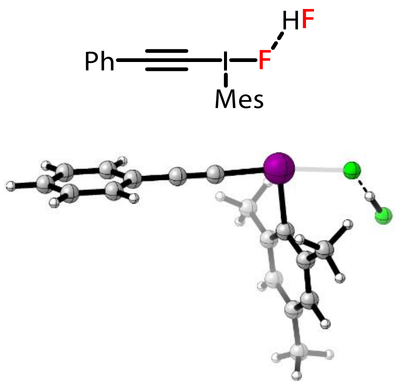

Thermal correction to the Gibbs free energy:
0.233823
Electronic energies (Def2TZVP): **-1155.23741038**
Charge = 0 Multiplicity = 1

C	-5.80586	0.607115	-1.34957
C	-4.49938	0.129409	-1.31773
C	-3.82374	0.027603	-0.09217
C	-4.46677	0.407108	1.096065
C	-5.7731	0.884355	1.053694
C	-6.44349	0.984479	-0.16655
H	-6.32679	0.685253	-2.29803
H	-3.9961	-0.1657	-2.23251
H	-3.93795	0.32516	2.040103
H	-6.26854	1.177657	1.973257
H	-7.46243	1.356696	-0.19559
C	-2.47741	-0.46523	-0.05138
C	-1.33676	-0.87365	-0.0113
I	0.572511	-1.63706	0.08197
C	1.470157	0.316688	-0.03928
C	2.150369	0.629731	-1.21625
C	1.335382	1.15734	1.064333
C	2.729107	1.901026	-1.2625
C	1.937718	2.415164	0.946602
C	2.635218	2.800523	-0.1984
H	3.268807	2.189001	-2.16102
H	1.855793	3.10513	1.782288
C	0.605728	0.784537	2.327721
H	-0.45472	0.591676	2.135108
H	1.035111	-0.11376	2.781694
H	0.675334	1.596527	3.052201
C	2.293421	-0.31409	-2.38009
H	2.791554	-1.23666	-2.0674
H	1.317032	-0.57792	-2.79857
H	2.885053	0.151661	-3.16904
C	3.299332	4.149948	-0.27865
H	4.358153	4.067674	-0.01273
H	3.243262	4.555435	-1.29151
H	2.835409	4.859908	0.409068
F	2.866366	-2.24523	0.141826
H	3.829448	-1.43835	0.508983
F	4.581259	-0.82332	0.797087

AI-(HF)(HF)'-out

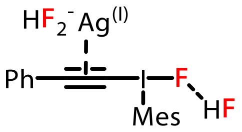

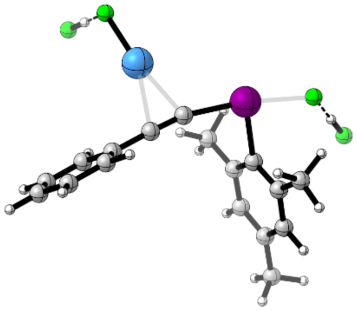

Thermal correction to the Gibbs free energy:
0.232827
Electronic energies (Def2TZVP): **-1502.54466480**
Charge = 0 Multiplicity = 1

C	-0.57794	0.961465	-0.39792
C	-1.49216	0.470279	0.542864
C	-2.61013	-0.27546	0.172938
C	-2.82319	-0.5417	-1.18247
C	-1.95444	-0.08632	-2.17608
C	-0.85797	0.652385	-1.72698
C	0.611613	1.764615	0.057119
H	-1.31525	0.68791	1.592662
C	-3.58092	-0.78588	1.204893
H	-3.69331	-1.11948	-1.48394
C	-2.2308	-0.38401	-3.62547
I	0.488053	1.403982	-3.23143
H	0.5543	1.939035	1.131872
H	0.654033	2.736742	-0.44289
H	1.549624	1.236491	-0.14691
H	-3.14591	-0.96957	-3.71862
H	-1.41395	-0.95828	-4.07372
H	-2.3501	0.542617	-4.19467
H	-3.26425	-0.51656	2.214007
H	-3.66663	-1.87478	1.14839
H	-4.57762	-0.36897	1.03374
C	1.975741	0.064249	-2.62411
C	2.622524	-0.90694	-2.25281
C	3.327717	-2.08359	-1.82926
C	3.904796	-2.13629	-0.55096
H	3.83093	-1.27604	0.107254
C	4.566652	-3.28924	-0.1443
H	5.013938	-3.33295	0.842815
C	4.655491	-4.38541	-1.00473
H	5.174173	-5.28281	-0.6837
C	4.08199	-4.3321	-2.27615
H	4.15272	-5.18517	-2.94228
C	3.416986	-3.18424	-2.69476
H	2.967609	-3.13102	-3.68061
Ag	4.072385	1.183364	-2.18624
F	5.778766	2.475277	-1.83234
F	6.444452	2.721879	0.360111
H	6.150855	2.614842	-0.61805
H	-2.33173	2.98042	-2.83979
F	-1.34868	2.697471	-3.71412
F	-3.06295	3.19827	-2.19699

(HF).(HF)'-out-Zint

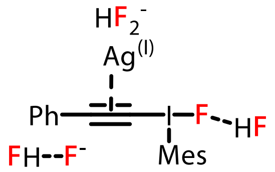

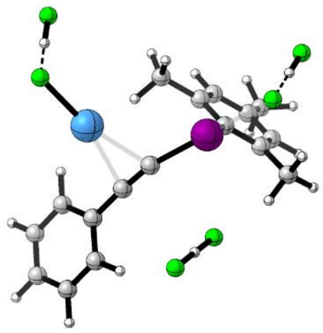

Thermal correction to the Gibbs free energy: **0.239131**
Electronic energies (Def2TZVP): **-1703.03790529**
Charge = -1 Multiplicity = 1

C	-1.87949	-0.14147	-1.75089
C	-2.71886	0.463953	-2.69367
C	-3.84161	1.202883	-2.32273
C	-4.1401	1.333336	-0.96499
C	-3.34958	0.757849	0.034135
C	-2.23592	0.044732	-0.41519
C	-0.67592	-0.91604	-2.21826
H	-2.48003	0.342892	-3.74693
C	-4.70949	1.862055	-3.36227
H	-5.02046	1.894807	-0.6625
C	-3.72394	0.909785	1.483176
I	-1.01842	-0.8989	1.092213
H	-0.73296	-1.08134	-3.29471
H	-0.59829	-1.89089	-1.7281
H	0.245455	-0.3594	-2.015
H	-4.60696	1.544345	1.57294
H	-2.89958	1.365742	2.041926
H	-3.94915	-0.06698	1.921656
H	-4.5787	1.394266	-4.34017
H	-4.44914	2.921108	-3.4583
H	-5.76486	1.805153	-3.08533
C	0.630177	0.288972	0.644991
C	1.427211	1.196767	0.450687
C	2.306471	2.318997	0.280593
C	2.897681	2.582507	-0.96456
H	2.704105	1.921431	-1.80404
C	3.725717	3.69038	-1.10732
H	4.185098	3.899322	-2.0675
C	3.964809	4.529966	-0.01692
H	4.612595	5.392969	-0.13283
C	3.375547	4.264083	1.220068
H	3.564325	4.919581	2.063831
C	2.543506	3.159294	1.379607
H	2.070823	2.928226	2.331525
Ag	2.482525	-1.01905	-0.18819
F	3.798377	-2.53196	-1.02265

F	2.936752	-3.40709	-2.97294
H	3.329219	-3.02308	-2.10426
H	-3.88887	-2.3154	0.46127
F	-2.98445	-2.12281	1.41038
F	-4.57861	-2.47062	-0.25479
F	-0.97714	2.169204	2.404499
H	-0.07756	2.05758	3.08744
F	0.816139	1.954361	3.779454

(HF).(HF)'-out-ZTS

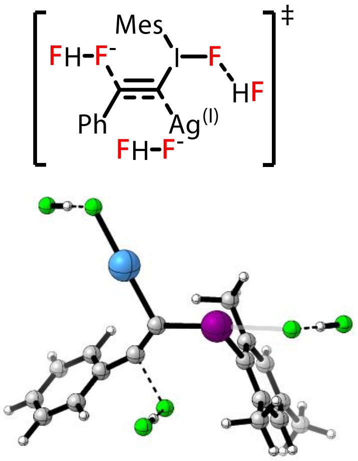

Thermal correction to the Gibbs free energy:
0.239131
Electronic energies (Def2TZVP): **-1703.03790529**
Charge = -1 Multiplicity = 1

F	4.086082	2.220372	1.735119
F	5.448712	0.763716	2.88823
H	4.843279	1.416613	2.374538
H	-3.99851	2.651446	-0.15019
F	-3.10994	2.752088	-1.05001
F	-4.71977	2.583553	0.587391
F	-0.54493	-1.57114	-1.73688
H	-0.25685	-1.23412	-2.93374
F	0.002546	-0.94974	-3.88789

C	-2.27606	-0.15563	1.434944
C	-3.21253	-1.06497	1.945948
C	-4.17806	-1.66451	1.140078
C	-4.2076	-1.34768	-0.2213
C	-3.30146	-0.45656	-0.79916
C	-2.36632	0.10731	0.070589
C	-1.26309	0.474239	2.354995
H	-3.17916	-1.29695	3.007161
C	-5.17297	-2.64043	1.712369
H	-4.95727	-1.80821	-0.86041
C	-3.36584	-0.14606	-2.2685
I	-0.97114	1.520995	-0.7575
H	-1.4314	0.142635	3.380304
H	-1.33321	1.566409	2.334443
H	-0.24111	0.198024	2.072915
H	-4.17128	-0.71298	-2.73787
H	-2.42208	-0.41721	-2.75142
H	-3.54675	0.921136	-2.42475
H	-5.08294	-2.70644	2.798346
H	-5.01589	-3.63954	1.294625
H	-6.19562	-2.34004	1.467675
C	0.747275	0.389769	-0.41397
C	0.983651	-0.8224	-0.67716
C	1.760589	-2.03143	-0.61277
C	2.18313	-2.4959	0.64167
H	1.89387	-1.95439	1.537348
C	2.975634	-3.63746	0.721452
H	3.307027	-3.99704	1.689651
C	3.338478	-4.31602	-0.44223
H	3.954489	-5.20717	-0.37755
C	2.911887	-3.85553	-1.6913
H	3.1958	-4.38805	-2.59274
C	2.123304	-2.71539	-1.783
H	1.775379	-2.34811	-2.74284
Ag	2.441542	1.309872	0.662045

(HF).(HF)’-out-ZTS

Thermal correction to the Gibbs free energy: **0.242911**
Electronic energies (Def2TZVP): **-1703.05107313**
Charge = -1 Multiplicity = 1

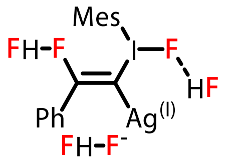

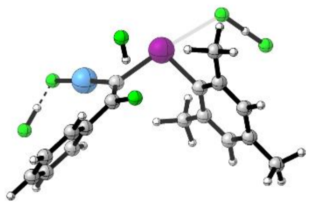

C	-0.45073	0.50314	0
C	0.37681	1.28826	0.81308
C	1.38904	2.08903	0.28652
C	1.58172	2.10038	-1.09728
C	0.7902	1.34388	-1.96522
C	-0.20904	0.56964	-1.37171
C	-1.51258	-0.36376	0.62596
H	0.22144	1.25934	1.8885
C	2.2532	2.93759	1.1828
H	2.37364	2.71338	-1.52113
C	1.04806	1.3723	-3.44703
I	-1.46863	-0.6121	-2.65265
H	-1.49146	-0.25804	1.71144
H	-1.35035	-1.41888	0.38217
H	-2.51319	-0.09047	0.27412
H	1.88542	2.03458	-3.67192
H	0.17371	1.73321	-3.99746
H	1.2813	0.36501	-3.8046
H	2.14855	2.64102	2.22842
H	1.97151	3.99221	1.10038
H	3.30641	2.85587	0.90185
C	-3.37096	0.30783	-2.20197
C	-3.37052	1.63812	-2.28168
C	-4.48183	2.55632	-1.96535
C	-5.37749	2.26343	-0.9285
H	-5.22193	1.37899	-0.31822
C	-6.44399	3.11775	-0.66035
H	-7.13019	2.88131	0.1464
C	-6.6184	4.27973	-1.41246
H	-7.44777	4.94658	-1.19998
C	-5.71489	4.58859	-2.42995
H	-5.84005	5.495	-3.01334
C	-4.64921	3.7353	-2.70472
H	-3.9531	3.98252	-3.49942
Ag	-4.97928	-1.02739	-1.69614
F	-6.63573	-2.29694	-1.1468
F	-8.13197	-1.11902	0.15194
H	-7.47082	-1.65259	-0.42674
H	1.63928	-1.82658	-1.93907
F	0.98637	-1.75993	-2.95424
F	2.21781	-1.90363	-1.03981
F	-2.25606	2.35206	-2.72845
H	-2.07575	2.2851	-4.46638
F	-1.98972	2.13834	-5.38479

(HF).(HF)'-in-Zint

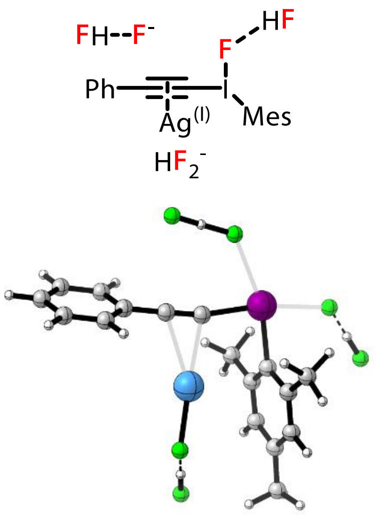

C	2.001246	-0.11241	1.248095
C	2.99544	0.866833	1.182823
C	3.763677	1.070184	0.033601
C	3.53578	0.254468	-1.07505
C	2.552628	-0.74093	-1.07652
C	1.796763	-0.87915	0.092406
C	1.207645	-0.29324	2.516647
H	3.17233	1.485986	2.058816
C	4.791517	2.168487	-0.00738
H	4.135534	0.390976	-1.97113
C	2.354202	-1.60465	-2.29452
I	0.26428	-2.37579	0.125135
H	1.578967	0.380651	3.289479
H	1.285358	-1.31827	2.891374
H	0.145555	-0.07333	2.361012
H	3.066828	-1.32776	-3.07242
H	1.344725	-1.48929	-2.70261
H	2.500468	-2.66083	-2.04808
H	5.347991	2.219172	0.932145
H	4.287746	3.129965	-0.15309
H	5.498898	2.01874	-0.82569
C	-1.17002	-0.87149	0.042487
C	-2.18038	-0.18327	0.055139
C	-3.38372	0.59882	0.106791
C	-4.06652	0.729369	1.324925
H	-3.67199	0.250581	2.214942
C	-5.24595	1.465464	1.375569
H	-5.77742	1.56454	2.316045
C	-5.74235	2.07413	0.221758
H	-6.66181	2.648674	0.266471
C	-5.0603	1.946632	-0.98987
H	-5.44676	2.420813	-1.88566
C	-3.88256	1.209854	-1.05319
H	-3.34336	1.104256	-1.98942
Ag	-0.16348	1.219949	-0.76136
F	0.77484	3.005854	-1.58547
F	2.150801	4.276391	-0.2431
H	1.539025	3.7047	-0.84098
H	3.382635	-3.37212	0.479549
F	2.163629	-3.76884	0.192042
F	4.325365	-3.08399	0.703035
F	-2.02965	-3.65531	0.144018
H	-3.16273	-3.19951	0.013683
F	-4.1443	-2.8215	-0.09783

Thermal correction to the Gibbs free energy:
0.243118
Electronic energies (Def2TZVP): **-1703.04887190**
Charge = -1 Multiplicity = 1

(HF).(HF)’-in-ZTS

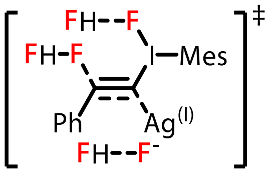

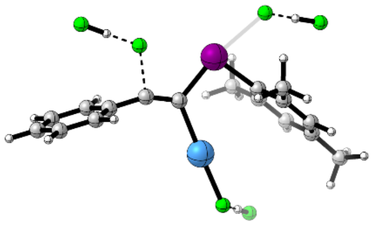

C	2.318489	0.349644	1.239434
C	3.404358	1.215956	1.082556
C	4.13366	1.282923	-0.10626
C	3.775733	0.440579	-1.15996
C	2.701144	-0.44916	-1.06731
C	1.996773	-0.44938	0.137936
C	1.566129	0.315547	2.544929
H	3.68212	1.855309	1.916755
C	5.269706	2.260796	-0.25277
H	4.346486	0.468718	-2.08467
C	2.360262	-1.35057	-2.22397
I	0.278419	-1.70764	0.291083
H	2.024593	1.001145	3.258591
H	1.574403	-0.68848	2.979885
H	0.519755	0.613748	2.415303
H	3.081061	-1.21679	-3.03167
H	1.362959	-1.12884	-2.61805
H	2.374756	-2.39901	-1.91116
H	5.785331	2.410684	0.698559
H	4.890425	3.23406	-0.58146
H	5.994233	1.916511	-0.9939
C	-1.00734	-0.07329	0.063162
C	-2.26695	-0.22523	0.120532
C	-3.63723	0.192249	0.07877
C	-4.2755	0.610504	1.256604
H	-3.72182	0.622381	2.18977
C	-5.60792	1.004654	1.210977
H	-6.10669	1.332108	2.116729
C	-6.30191	0.976212	-0.00086
H	-7.34237	1.283411	-0.03211
C	-5.66822	0.557493	-1.17328
H	-6.21355	0.539303	-2.11066
C	-4.33608	0.162523	-1.13811
H	-3.82848	-0.16916	-2.03804
Ag	-0.18085	1.907571	-0.40749
F	0.684528	3.826915	-0.90627
F	2.093072	4.829824	0.617402
H	1.466761	4.380325	-0.06179
H	3.358205	-3.09339	0.591606
F	2.142127	-3.38686	0.426127
F	4.362232	-2.87141	0.731909
F	-2.54166	-2.14802	0.444282
H	-3.78391	-2.59381	0.558133
F	-4.7241	-2.94158	0.645348

Thermal correction to the Gibbs free energy:
0.241842
Electronic energies (Def2TZVP): **-1703.02803408**
Charge = -1 Multiplicity = 1

(HF).(HF)'-in-ZP

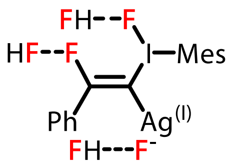

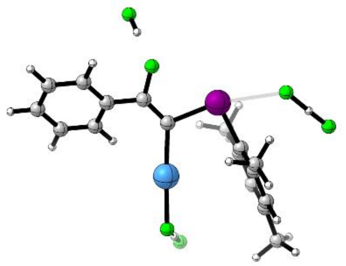

Thermal correction to the Gibbs free energy:
0.244108
Electronic energies (Def2TZVP): **-1703.05690453**
Charge = -1 Multiplicity = 1

C	2.081144	-0.2364	1.195979
C	3.158207	0.646928	1.317047
C	3.997465	0.944778	0.241612
C	3.761597	0.321844	-0.98449
C	2.703306	-0.57335	-1.17288
C	1.888844	-0.81088	-0.06466
C	1.198394	-0.51448	2.384862
H	3.337434	1.115888	2.2814
C	5.118984	1.93777	0.399423
H	4.416968	0.532253	-1.82595
C	2.490452	-1.22697	-2.51157
I	0.168497	-2.04431	-0.33373
H	1.576443	0.011095	3.262708
H	1.164484	-1.58423	2.611875
H	0.171232	-0.17901	2.202552
H	3.28751	-0.94358	-3.20043
H	1.535163	-0.92121	-2.95149
H	2.483328	-2.3158	-2.40456
H	5.561595	1.876735	1.396468
H	4.742629	2.957429	0.265804
H	5.902528	1.769918	-0.34254
C	-1.12534	-0.30625	-0.30752
C	-2.41145	-0.64266	-0.25372
C	-3.59308	0.236623	-0.19929
C	-3.51949	1.501557	0.399636
H	-2.5914	1.823082	0.862356
C	-4.63707	2.330495	0.423902
H	-4.56995	3.306667	0.893167
C	-5.84275	1.902841	-0.13505
H	-6.71421	2.548952	-0.10928
C	-5.92536	0.63822	-0.71698
H	-6.8602	0.296832	-1.1496
C	-4.80785	-0.19317	-0.75041
H	-4.87537	-1.17181	-1.21454
Ag	-0.10773	1.600262	-0.48332
F	0.996531	3.447853	-0.68895
F	1.865419	4.297805	1.268898
H	1.481436	3.917524	0.39414
H	3.40276	-3.55264	-0.23263
F	2.25183	-3.8147	-0.47985
F	4.43296	-3.3424	-0.01254
F	-2.79243	-1.99147	-0.26748
H	-3.93795	-2.59245	0.930414
F	-4.55094	-2.90269	1.562944

(HF).(HF)'-out-Eint

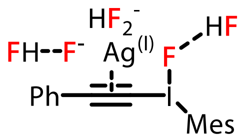

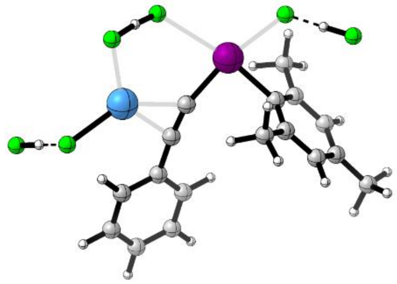

Thermal correction to the Gibbs free energy:
0.241241
Electronic energies (Def2TZVP): **-1703.05431278**
Charge = -1 Multiplicity = 1

C	2.692839	-1.61597	1.360699
C	4.082812	-1.46923	1.30961
C	4.748569	-1.1232	0.133386
C	3.998832	-0.92731	-1.02848
C	2.607511	-1.05925	-1.04529
C	2.002286	-1.3967	0.168059
C	2.021611	-2.00787	2.650861
H	4.65434	-1.63653	2.218906
C	6.243931	-0.94293	0.116817
H	4.505899	-0.66933	-1.9549
C	1.846411	-0.84895	-2.32855
I	-0.13	-1.60893	0.209812
H	2.767007	-2.14632	3.434966
H	1.466422	-2.94323	2.530751
H	1.319454	-1.23662	2.983786
H	2.537721	-0.62527	-3.14183
H	1.142373	-0.01411	-2.24495
H	1.27721	-1.74249	-2.60279
H	6.714575	-1.47139	0.948396
H	6.500036	0.117939	0.204946
H	6.673283	-1.30958	-0.81859
C	-0.26765	0.463826	0.081589
C	0.016565	1.654101	0.109821
C	0.500261	3.003705	0.224327
C	1.701572	3.207276	0.923384
H	2.221455	2.357566	1.355052
C	2.212788	4.493936	1.053957
H	3.141214	4.650515	1.59286
C	1.532775	5.577837	0.495382
H	1.933351	6.580795	0.600828
C	0.339687	5.373495	-0.1984
H	-0.18874	6.215454	-0.6332
C	-0.18231	4.090559	-0.34031
H	-1.10905	3.930227	-0.88666
Ag	-2.19236	1.389272	-1.11318
F	-3.82797	-0.22715	-1.38317
F	-2.86825	-1.38341	0.312178
H	-3.37955	-0.85838	-0.51296
H	1.693155	-4.34408	0.128699
F	0.49472	-3.86712	0.400743
F	2.603817	-4.72647	-0.07934
F	-2.95181	3.333857	-1.96951
H	-3.52421	4.127929	-1.17964
F	-3.98029	4.786855	-0.51967

(HF).(HF)'-out-EP

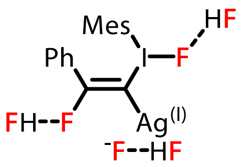

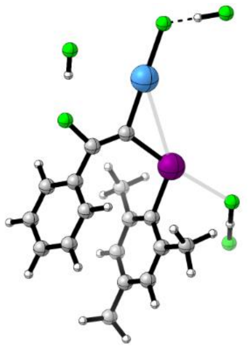

Thermal correction to the Gibbs free energy:
0.242995
Electronic energies (Def2TZVP): **-1703.05091311**
Charge = -1 Multiplicity = 1

Ag	-3.53124	-0.03035	-0.067
F	-5.64408	-0.46169	-0.16649
F	-6.29514	-2.61991	-0.6405
H	-6.00488	-1.6569	-0.42902
H	2.211485	-3.82831	0.031555
F	1.107717	-3.66554	0.495164
F	3.186309	-3.99465	-0.38352
F	-1.85667	2.667694	-0.28825
H	-1.54155	4.238673	0.370772
F	-1.39997	5.087065	0.739915

C	2.64629	-0.88899	1.237434
C	3.974608	-0.47238	1.109721
C	4.468109	0.0799	-0.07255
C	3.614561	0.182054	-1.17205
C	2.279775	-0.2288	-1.11511
C	1.840707	-0.7388	0.10812
C	2.154439	-1.45518	2.542424
H	4.633044	-0.57196	1.969327
C	5.881828	0.591859	-0.15247
H	3.989759	0.597561	-2.10433
C	1.408075	-0.13113	-2.33957
I	-0.22688	-1.30233	0.27427
H	2.972713	-1.50681	3.262312
H	1.748589	-2.45965	2.390931
H	1.364054	-0.83086	2.973792
H	1.961067	0.332126	-3.15829
H	0.51158	0.470324	-2.1576
H	1.082401	-1.12496	-2.66409
H	6.529287	0.084541	0.566114
H	5.905198	1.663828	0.074353
H	6.296352	0.457072	-1.15431
C	-1.42973	0.443237	0.045671
C	-0.90664	1.659448	-0.03908
C	0.43272	2.271881	0.054864
C	1.224792	2.088987	1.191591
H	0.84962	1.487873	2.016028
C	2.484315	2.679974	1.26942
H	3.097276	2.526598	2.151962
C	2.953983	3.46234	0.215438
H	3.936766	3.919592	0.275718
C	2.160252	3.660898	-0.91634
H	2.523383	4.271495	-1.73671
C	0.901503	3.073156	-0.9958
H	0.281096	3.223721	-1.87502

(HF).(HF)’-in-Eint

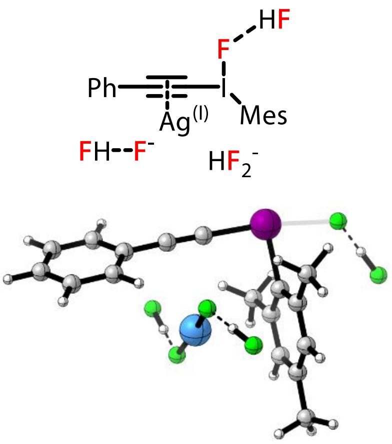

Thermal correction to the Gibbs free energy:
0.241576
Electronic energies (Def2TZVP): **-1703.05786367**
Charge = -1 Multiplicity = 1

C	-2.2077	0.55126	-0.00514
C	-2.55228	1.881117	-0.27642
C	-3.02267	2.744064	0.715729
C	-3.16016	2.262151	2.019131
C	-2.83031	0.94867	2.365611
C	-2.3581	0.149243	1.323989
C	-1.70451	-0.34263	-1.10636
H	-2.44097	2.241839	-1.29538
C	-3.34517	4.179208	0.393281
H	-3.51613	2.923309	2.803909
C	-2.98345	0.47383	3.783722
I	-1.81851	-1.8762	1.813877
H	-1.70391	0.200165	-2.05219
H	-2.33807	-1.22831	-1.21591
H	-0.67617	-0.67037	-0.91794
H	-3.41288	1.267056	4.395692
H	-2.00622	0.216679	4.205094
H	-3.63374	-0.40476	3.833861
H	-3.64162	4.292437	-0.65165
H	-2.46598	4.809908	0.561804
H	-4.14964	4.55401	1.029815
C	0.191476	-1.41615	1.751598
C	1.366634	-1.11514	1.780808
C	2.738012	-0.69696	1.846266
C	3.72205	-1.33604	1.078784
H	3.444666	-2.15653	0.425277
C	5.043556	-0.90789	1.161306
H	5.806412	-1.40173	0.568648
C	5.386222	0.155082	1.999131
H	6.417961	0.486549	2.057354
C	4.40582	0.793022	2.761038
H	4.673335	1.619545	3.411095
C	3.081265	0.3706	2.692822
H	2.302284	0.854132	3.278771
Ag	0.346752	1.650106	1.255802
F	0.133779	1.65976	3.450574
F	-1.25971	3.16219	4.504685

H	-0.64896	2.484997	4.024762
H	-4.99902	-1.28414	1.242225
F	-4.17386	-2.07492	1.884721
F	-5.64515	-0.68506	0.744089
F	0.789573	1.810804	-0.89479
H	1.220724	0.76082	-1.44809
F	1.576533	-0.10031	-1.90142

(HF).(HF)’-in-EP

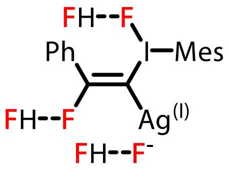

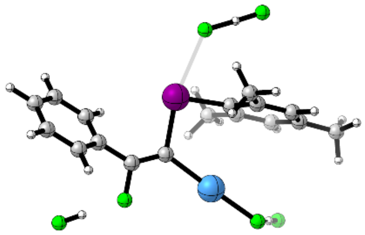

C	0.879238	0.71996	0.04346
C	1.963743	1.601437	0.034026
C	2.829544	1.701035	-1.05771
C	2.612836	0.876804	-2.16201
C	1.546245	-0.0276	-2.21736
C	0.7056	-0.05959	-1.10497
C	-0.03231	0.657592	1.241574
H	2.128257	2.229964	0.905877
C	3.96039	2.695941	-1.04251
H	3.288477	0.931778	-3.01176
C	1.35361	-0.90312	-3.42606
I	-1.02556	-1.3057	-1.19567
H	0.324401	1.334919	2.018554
H	-0.07125	-0.35383	1.65706
H	-1.05507	0.950496	0.978293
H	2.169061	-0.75208	-4.13477
H	0.411811	-0.67115	-3.9346
H	1.331023	-1.95661	-3.13166
H	4.457009	2.710829	-0.06904
H	3.579861	3.704696	-1.23378

Thermal correction to the Gibbs free energy: **0.239824**

Electronic energies (Def2TZVP): **-1703.05234143**

Charge = -1 Multiplicity = 1

H	4.702086	2.463742	-1.8094
C	-2.28214	0.412009	-1.58878
C	-3.56655	0.103387	-1.72874
C	-4.35564	-1.14164	-1.62227
C	-4.33786	-1.89952	-0.44525
H	-3.76582	-1.55361	0.41173
C	-5.07513	-3.08012	-0.3631
H	-5.05836	-3.66214	0.55238
C	-5.84313	-3.50033	-1.44812
H	-6.41854	-4.41802	-1.38225
C	-5.87855	-2.73701	-2.617
H	-6.47924	-3.06071	-3.46063
C	-5.14035	-1.56045	-2.70544
H	-5.16223	-0.96638	-3.61432
Ag	-1.2867	2.312013	-1.86077
F	-0.23365	4.177732	-2.15908
F	0.624507	5.1865	-0.27395
H	0.243461	4.73449	-1.11587
H	2.195452	-2.86776	-0.81755
F	1.013382	-3.09114	-0.91677
F	3.250003	-2.69109	-0.72638
F	-4.3913	1.174826	-2.08898
H	-6.04713	1.149889	-1.56852
F	-6.93803	1.144414	-1.28335

References

1. Ueoka, R., Bortfeld-Miller, M., Morinaka, B. I., Vorholt, J. A. & Piel, J. Toblerols: Cyclopropanol-Containing Polyketide Modulators of Antibiosis in Methylobacteria. *Angewandte Chemie* **130**, 989–993 (2018).

2. Bell, G. E. *et al.* Synthesis of 2-BMIDA Indoles via Heteroannulation: Applications in Drug Scaffold and Natural Product Synthesis. *Org Lett* **24**, 3024–3027 (2022).

3. Munoz, E. *et al.* Synthesis and biological evaluation of niclosamide PROTACs. *Bioorg Med Chem Lett* **72**, 128870 (2022).

4. Bernar, I., Fiser, B., Blanco-Ania, D., Gómez-Bengoa, E. & Rutjes, F. P. J. T. Pd-Catalyzed Hydroamination of Alkoxyallenes with Azole Heterocycles: Examples and Mechanistic Proposal. *Org Lett* **19**, 4211–4214 (2017).

5. Mathieu, G., Patel, H. & Lebel, H. Convenient Continuous Flow Synthesis of N-Methyl Secondary Amines from Alkyl Mesylates and Epoxides. *Org Process Res Dev* **24**, 2157–2168 (2020).

6. Ayres, J. N. *et al.* Synthesis and reactivity of N-allenyl cyanamides. *Org. Lett.* **20**, 5282–5285 (2018).

7. Tsuzuki, Y. *et al. 4-Deoxy-4-Fluoro-Xyloside Derivatives as Inhibitors of Glycosaminoglycan Biosynthesis*. (2010).

8. Lasányi, D. & Tolnai, G. L. Copper-Catalyzed Ring Opening of [1.1.1]Propellane with Alkynes: Synthesis of Exocyclic Allenic Cyclobutanes. *Org Lett* **21**, 10057–10062 (2019).

9. Schimler, S. D., Hall, D. J. & Debbert, S. L. Anticancer (hexacarbonyldicobalt)propargyl aryl ethers: Synthesis, antiproliferative activity, apoptosis induction, and effect on cellular oxidative stress. *J Inorg Biochem* **119**, 28–37 (2013).

10. Maibunkaew, T., Thongsornkleeb, C., Tummatorn, J., Bunrit, A. & Ruchirawat, S. Practical and metal-free electrophilic aromatic halogenation by interhalogen compounds generated in situ from N-halosuccinimide and catalytic TMSCL. *Synlett* **25**, 1769–1775 (2014).

11. Semleit, N., Kreuzahler, M. & Haberhauer, G. Gold(I)-Catalyzed Allene–Diene–Alkyne Coupling Reaction to Polycycles. *European J Org Chem* **2020**, 6629–6634 (2020).

12. Spielmann, K., Xiang, M., Schwartz, L. A. & Krische, M. J. Direct Conversion of Primary Alcohols to 1,2-Amino Alcohols: Enantioselective Iridium-Catalyzed Carbonyl Reductive Coupling of Phthalimido-Allene via Hydrogen Auto-Transfer. *J Am Chem Soc* **141**, 14136–14141 (2019).

13. Gao, M. *et al.* Ruthenium Carbene-Mediated Construction of Strained Allenes via the Enyne Cross-Metathesis/Cyclopropanation of 1,6-Enynes. *Org Lett* **22**, 1139–1143 (2020).

14. Wen, H., Luo, N., Zhu, Q. & Luo, R. Amide Iridium Complexes As Catalysts for Transfer Hydrogenation Reduction of N-sulfonylimine. *Journal of Organic Chemistry* **86**, 3850–3859 (2021).

15. Wang, X. *et al.* Nitrosoarenes as Nitrogen Source for Generation of Sulfonamides with the Insertion of Sulfur Dioxide under Metal-Free Conditions†. *Chin J Chem* **38**, 1098–1102 (2020).

16. García, L. *et al.* Transition-Metal-Free Stereoselective Borylation of Allenamides. *Chemistry - A European Journal* **24**, 14059–14063 (2018).

17. Morri, A. K., Thummala, Y. & Doddi, V. R. The Dual Role of 1,8-Diazabicyclo[5.4.0]undec-7-ene (DBU) in the Synthesis of Terminal Aryl- and Styryl-Acetylenes via Umpolung Reactivity. *Org Lett* **17**, 4640–4643 (2015).

18. Kandasamy, M., Huang, Y. H., Ganesan, B., Senadi, G. C. & Lin, W. Y. In Situ Generation of Alkynylzinc and Its Subsequent Negishi Reaction in a Flow Reactor. *European J Org Chem* **2019**, 4349–4356 (2019).

19. Feng, Y. S., Xie, C. Q., Qiao, W. L. & Xu, H. J. Palladium-catalyzed trifluoroethylation of terminal alkynes with 1,1,1-trifluoro-2-iodoethane. *Org Lett* **15**, 936–939 (2013).

20. Munoz, S. B. *et al.* Synthesis of 3-substituted isoindolin-1-ones via a tandem desilylation, cross-coupling, hydroamidation sequence under aqueous phase-transfer conditions. *Org Biomol Chem* **14**, 85–92 (2015).

21. Zimin, D. P. *et al.* Gold-Catalyzed Functionalization of Semicarbazides with Terminal Alkynes to Achieve Substituted Semicarbazones. *European J Org Chem* **2019**, 6094–6100 (2019).

22. Eisterhold, A. M. *et al.* Expanded Cyclotetrabenzoins. *Org Lett* **23**, 781–785 (2021).

23. Li, T. *et al.* Design and synthesis of O-GlcNAcase inhibitors via 'click chemistry' and biological evaluations. *Carbohydr Res* **346**, 1083–1092 (2011).

24. Wang, W. *et al.* 3-Silaazetidine: An Unexplored yet Versatile Organosilane Species for Ring Expansion toward Silaazacycles. *J Am Chem Soc* **143**, 11141–11151 (2021).

25. Tang, R. J., Milcent, T. & Crousse, B. Regioselective Halogenation of Arenes and Heterocycles in Hexafluoroisopropanol. *Journal of Organic Chemistry* **83**, 930–938 (2018).

26. Boelke, A., Sadat, S., Lork, E. & Nachtsheim, B. J. Pseudocyclic bis-N-heterocycle-stabilized iodanes - synthesis, characterization and applications. *Chemical Communications* **57**, 7434–7437 (2021).

27. Bhat, A. H., Alavi, S. & Grover, H. K. Tandem Carbenoid C–H Functionalization/Conia-ene Cyclization of *N* -Propargyl Indoles Generates Pyrroloindoles under Cooperative Rh(II)/Zn(II) Catalysis. *Org Lett* **22**, 224–229 (2020).

28. Sousa, S. C. A., Bernardo, J. R., Wolff, M., Machura, B. & Fernandes, A. C. Oxo-rhenium(V) complexes containing heterocyclic ligands as catalysts for the reduction of sulfoxides. *European J Org Chem* **2014**, 1855–1859 (2014).

29. Duffy, B. C., Howard, K. T. & Chisholm, J. D. Alkylation of thiols with trichloroacetimidates under neutral conditions. *Tetrahedron Lett* **56**, 3301–3305 (2015).

30. Canlas, G. M. R. & Gilbertson, S. R. [4+2+2] Cycloaddition catalyzed by a new cationic rhodium-bisphosphine monooxide complex. *Chemical Communications* **50**, 5007–5010 (2014).

31. Longin, O., van de Langemheen, H. & Liskamp, R. M. J. An orthogonally protected CycloTriVeratrylene (CTV) as a highly pre-organized molecular scaffold for subsequent ligation of different cyclic peptides towards protein mimics. *Bioorg Med Chem* **25**, 5008–5015 (2017).

32. Xu, Z. F., An, Y., Chen, Y. & Duan, S. Rhodium-catalysed synthesis of fused pyrimidine derivatives employing N-sulfonyl-1,2,3-triazoles as a 1-aza-[4C] synthon. *Tetrahedron Lett* **60**, 1849–1853 (2019).

33. Yoshida, M., Osafune, K. & Hara, S. Facile synthesis of iodonium salts by reaction of organotrifluoroborates with p-iodotoluene difluoride. *Synthesis (Stuttg)* 1542–1546 (2007) doi:10.1055/s-2007-966031.

34. Yoshida, M., Komata, A. & Hara, S. Stereoselective synthesis of fluoroalkenes via (Z)-2-fluoroalkenyliodonium salts. *Tetrahedron* **62**, 8636–8645 (2006).

35. Cao, S. *et al.* AgN3-Catalyzed Hydroazidation of Terminal Alkynes and Mechanistic Studies. *J Am Chem Soc* **142**, 7083–7091 (2020).

36. Sharma, S., Kumar, M., Vishwakarma, R. A., Verma, M. K. & Singh, P. P. Room Temperature Metal-Catalyzed Oxidative Acylation of Electron-Deficient Heteroarenes with Alkynes, Its Mechanism, and Application Studies. *J Org Chem* **83**, 12420–12431 (2018).

37. Merritt, E. A. & Olofsson, B. Synthesis of a range of iodine(III) compounds directly from iodoarenes. *European J Org Chem* 3690–3694 (2011) doi:10.1002/ejoc.201100360.

38. Dixon, L. I. *et al.* Synthesis and reactivity of aryl(alkynyl)iodonium salts. *European J Org Chem* 2334–2345 (2013) doi:10.1002/ejoc.201300092.

39. Gao, C., Nakao, S. & Blum, S. A. Borylative Heterocyclization without Air-Free Techniques. *J Org Chem* **85**, 10350–10368 (2020).

40. Shi, X., Song, T., Li, Q., Guo, X. & Yang, Y. Mesoporous Graphitic Carbon Nitride Photocatalyzed Switchable Divergent Perfluoroalkylation of Terminal Alkynes. *Org Lett* **24**, 8724–8728 (2022).

41. Ueda, T., Konishi, H. & Manabe, K. Trichlorophenyl formate: highly reactive and easily accessible crystalline CO surrogate for palladium-catalyzed carbonylation of aryl/alkenyl halides and triflates. *Org. Lett.* **14**, 5370–5373 (2012).

42. Pritchard, B. P., Altarawy, D., Didier, B., Gibson, T. D. & Windus, T. L. New basis set exchange. An open, up-to-date resource for the molecular sciences community. *J Chem Inf Model* **59**, 4814–4820 (2019).
